# Predicting the substituent effects in the optical and electrochemical properties of *N,N*′-substituted isoindigos

**DOI:** 10.1007/s43630-021-00071-5

**Published:** 2021-07-05

**Authors:** Ferdinand L. Kiss, Brian P. Corbet, Nadja A. Simeth, Ben L. Feringa, Stefano Crespi

**Affiliations:** 1 Faculty for Science and Engineering, Stratingh Institute for Chemistry, University of Groningen, Nijenborgh 4, 9747 AG Groningen, The Netherlands; 2 Present Address: Department Chemie, Ludwig-Maximilians-Universität München, 81377 Munich, Germany

## Abstract

Isoindigo, the structural isomer of the well-known dye indigo, has seen a major revival recently because of the increasing interest of its use as a potential drug core structure and for the development of organic photovoltaic materials. Highly beneficial for diverse applications are its facile synthesis, straightforward functionalisation and the broad absorption band in the visible range. Moreover, its intrinsic electron deficiency renders isoindigo a promising acceptor structure in bulk heterojunction architectures. Here we present new insights into the substituent effects of *N*-functionalised isoindigos, developing a reliable and fast in silico screening approach of a library of compounds. Using experimental UV–Vis and electrochemical data increased the accuracy of the TD-DFT method employed. This procedure allowed us to accurately predict the optical and electrochemical properties of *N*-functionalised isoindigos and the elucidation of the relationship between substituent effects and electronic properties.

**Supplementary Information:**

The online version contains supplementary material available at 10.1007/s43630-021-00071-5.

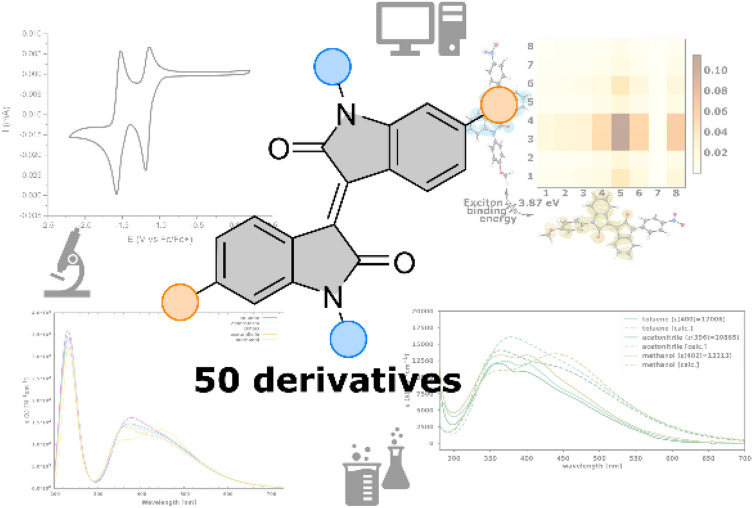

## Introduction

**Fig. 1:**
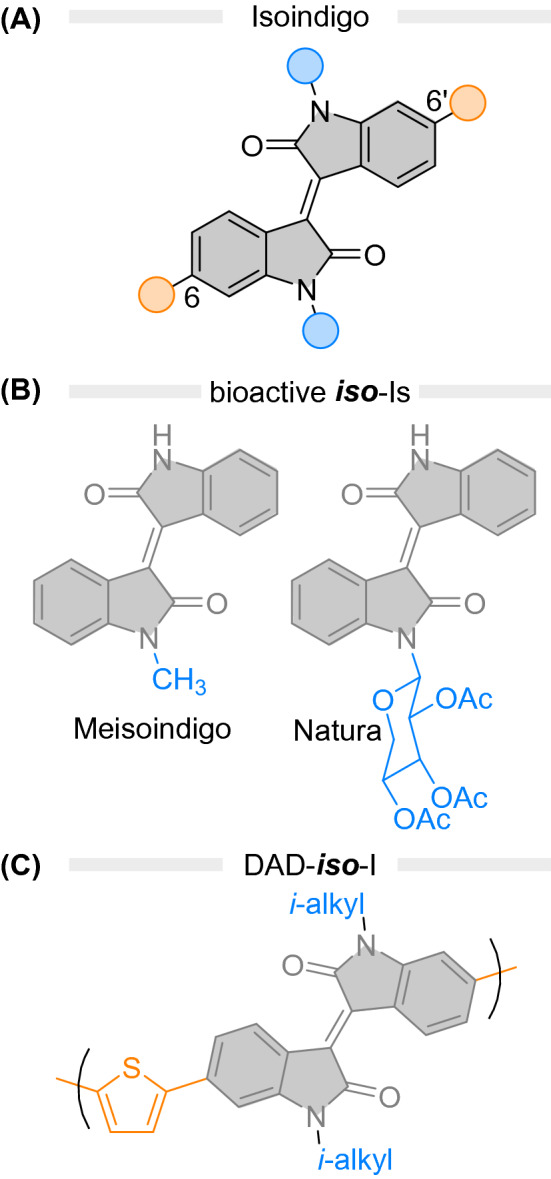
**A** Isoindigo with the 6,6′- and *N*,*N*′-substitution position highlighted. **B** Bioactive *N*-substituted ***iso*-I**s such as Meisoindigo (left) and Natura (right) are used in cancer treatment [4, 24]. **C** In recent OPV-research, the 6,6′-substitution pattern was used as a handle to obtain DAD-materials. The exemplary structure is one of the initial proposed OPV-polymers [17].

Isoindigo was first synthesised in 1842 [1] and initially identified with the name "indin" (***iso*-I**, Fig. 1A) [2]. This structure belongs to the indigoid family of compounds, together with indigo itself, indirubin and the related thioindigo. Similarly to indirubin scaffolds [3], ***iso-*I** became attractive to the pharmaceutical industry due to the properties of some of its derivatives. *N*-substituted ***iso-*I**s have found use in leukaemia treatment under the commercial names Natura and Meisoindigo (Fig. 1B) [3, 4]. Other molecules based on the same motif showed promising activity against different tumour strains under in vitro conditions [3, 5]. Indigo- and thioindigo- compounds [6] are widely employed as dyes in the textile industry [7] and as photosensitisers [8, 9] and are studied regarding their photophysical and photochromic properties [10–14]. In contrast, the optical properties of ***iso*-I** were the subject of a more recent research effort [15–19] as it was realised that ***iso-*I** is highly suitable for organic photovoltaics (OPVs) [17] and optoelectronics [15–19]. The facile synthesis of ***iso***-**I** and its derivatives opens numerous possibilities for various applications. Indeed, several 6,6′-substituted ***iso*-I** derivatives are reported as p-type donor in donor–acceptor–donor (DAD) materials and conjugated polymers due to its electron deficiency [4, 19]. On the other hand, installing substituents at the lactam nitrogen is largely unexplored [4]. A limited selection of *N*-alkylations is reported to overcome solubility issues in ***iso*-I** OPV research [4]. At the same time, aromatic substituents at the nitrogen are rare and only found in a few bio-active ***iso*-I**s [3, 5]. ***iso***-**I** is very stable towards photobleaching [20], a property attributable to the ultrafast intramolecular singlet fission that the molecule undergoes after excitation [21]. This sub-ns event leads to efficient triplet pair separation in thiophene-functionalised derivatives, while in solution it affords radiationless deactivation of the excited state, recovering the starting material at the ground state. Isoindigo absorbs in the visible region of the UV–Vis absorption spectrum (3900 M^−1^ cm^−1^ in DMSO at the absorption maximum at 490 nm [15]) tailing in the near-infrared (NIR) [15, 22, 23]. Consequently, the molecule holds great potential for high conversion efficiency in photochemical processes using visible light.

The introduction of substituents onto the ***iso***-**I** core structure allows tuning its UV–Vis absorption profile [24, 25]. The *π*-system of ***iso-*I**s can be easily extended at the 6,6′-position (Fig. 1A, orange). Substituents at the lactam nitrogens (*N*,*N*′; see Fig. 1, blue), such as alkyl chains, prevent *π*–*π* stacking and lead to increased solubility of ***iso-*I** derivatives [19]. However, to the best of our knowledge, the influence of *N*,*N*′-substituents on the optical and electronic properties of ***iso***-**I** were not systematically studied so far. Functionalisation on the lactam nitrogen is synthetically straightforward and would provide an additional handle for tuning the electronic properties of the core structure. A reliable prediction tool is crucial to efficiently judge the outcome of a synthetic modification and facilitate effective rational design for specific optoelectronic applications.

Computational chemistry provides valuable methods to predict the properties of a compound, supporting the development of new molecules, drugs and materials [26, 27]. To apply quantum mechanical calculations to a library of compounds, the availability of a fast and accurate method is a key requirement. In this context, density functional theory (DFT) is known as the "work-horse" of current theoretical studies in chemistry and physics [28, 29]. However, DFT methods often vary in their performances [30, 31], making the choice of the proper functional for a specific application of uttermost importance [29, 32]. Besides various attempts to categorise functionals by accuracy in a hierarchy as in the Perdew "Jacob's Ladder" [33], extensive benchmarks are needed to identify the functional best suited for a specific use.

Here, we present a library of 50 ***iso-*I** derivatives focusing on the *N*,*N*′-functionalisation pattern. The library was explored computationally, utilising an extensively benchmarked method to obtain insights into the substitution effects on the optical and electronic properties of ***iso-*I**. Additionally, we synthesised a limited subset of selected derivatives to experimentally validate and provide insights into the quality of the computational methods. We further utilised Koopmans’ theorem and parametrisation from experimental values to gain an accurate understanding of the dependencies of both optical and electrochemical properties of ***iso-*I** and its derivatives being part of the library. This methodology will serve as a predictive tool and consequently pave the way for the future rational design of ***iso***-**I**s as chromophore in material sciences and organic photovoltaics.

## Results and discussion

### Design and synthesis

**Fig. 2:**
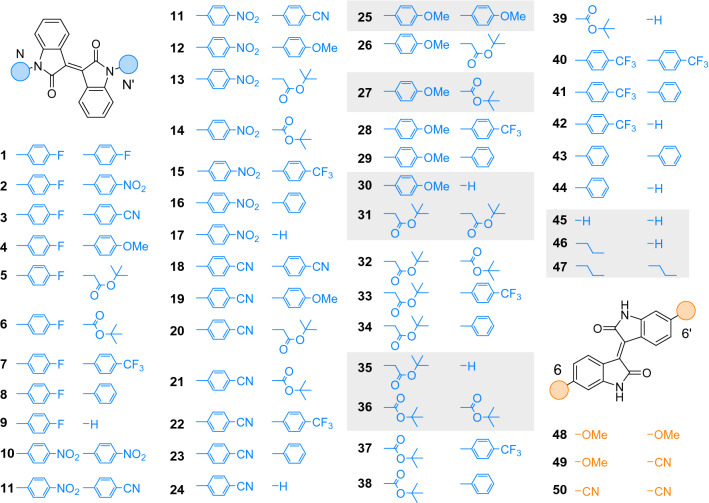
The calculation library consists of permutations of *N*,*N*′-substituents: hydrogen, 4-methoxyphenyl, 4-nitrophenyl, 4-trifluoromethylphenyl, 4-fluorophenyl, *t-*butyl-acetyl and *t*-butoxycarbonyl, as well as 6,6′-permutations of nitrile and methoxy substituents. The derivatives highlighted in the grey boxes have been synthesised in this work

We explored a library of functionalised ***iso***-**I** derivatives employing substituents with varying electronic and steric demand to rationalise their effect on the properties of the chromophore (Fig. 2). We focused on the less explored *N*,*N*′-functionalisation over the 6,6′-substitution of ***iso*-I**. We selected 4-methoxyphenyl as electron-donor and 4-nitrophenyl, 4-trifluoromethylphenyl, 4-fluorobenzyl and benzonitrile as electron-acceptor units, respectively, while the unsubstituted phenyl group serves as a reference. Additionally, *t*-butyl-acetyl and *t*-butyloxycarbonyl were selected for their different steric demand and electronic character. Permutation of the aforementioned *N*,*N*′-substituents resulted in a library of 45 derivatives (Fig. 2, blue, **1**–**45**). *N*,*N*′-propyl and bis-propyl substituted derivatives were included, to serve as a reference for the commonly used alkylated ***iso*-I** derivatives in the OPV research (Fig. 2, blue, **46–47**). For comparison, we included a nitrile group as electron-poor and a methoxy group as electron-rich substituents on the more frequently used 6,6′-position, providing three additional derivatives (Fig. 2, orange, **48–50**). Compounds ***iso***-**I**s **25**, **27**, **30**, **31**, **35**, **36**, **45**, **46** and **47** (Fig. 2, grey boxes) were synthesised to reference our library. A detailed overview of all synthetic steps and procedures is available in the SI.

### Benchmark study

**Fig. 3:**
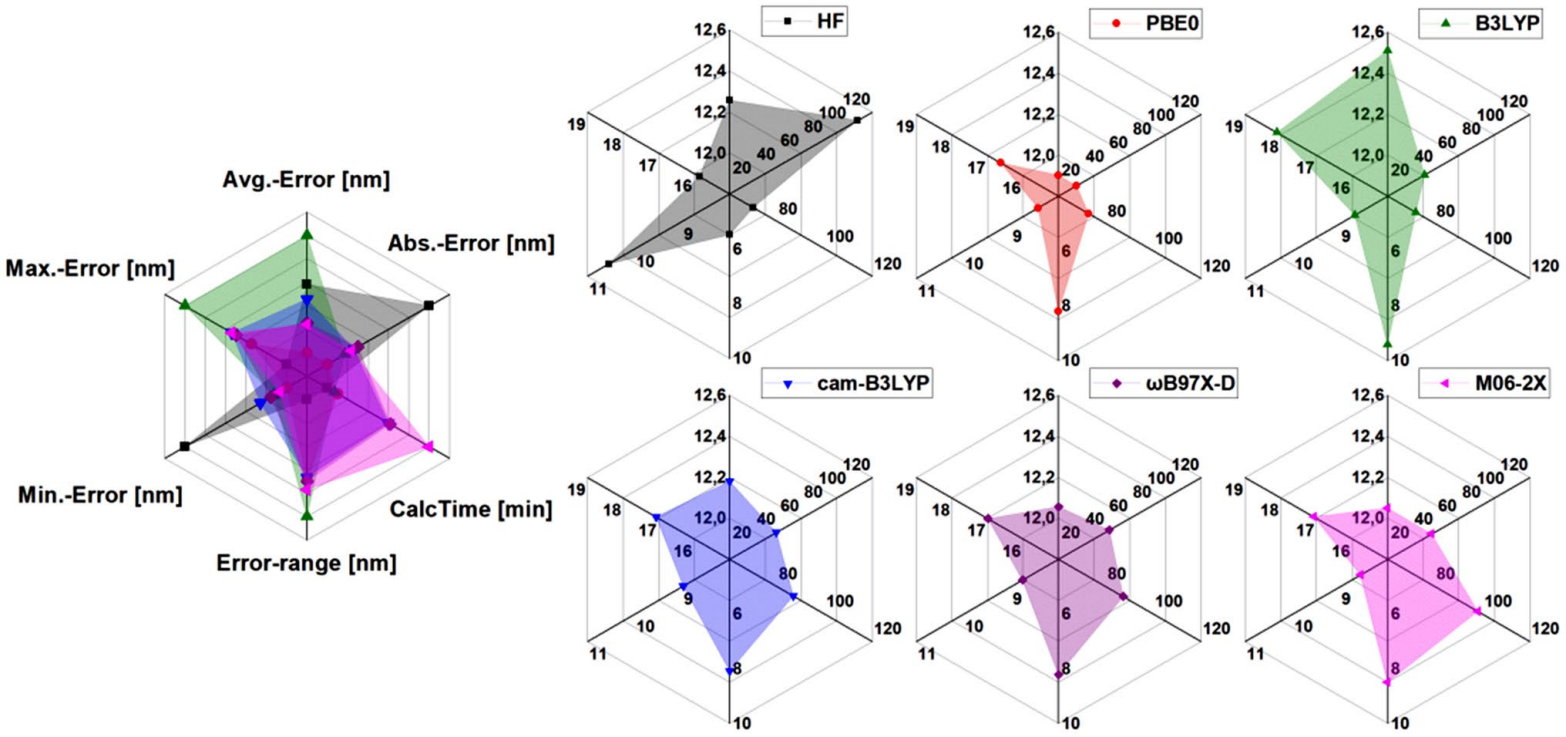
Benchmarks of the error of the change of *λ*_max_ in different solvents (toluene, methanol, chloroform, acetonitrile) relative to DMSO (Avg.-Error) computed at the TD-HF level and at a selected number of TD-DFT functionals with the respective SMD solvent model and 25 computed states versus experimental results. The best fitting functional was chosen based on the minimisation of Avg.-Error, the absolute deviation of *λ*_max_ in the chosen solvents from experimental results (Abs.-Error), the computing wall time (CalcTime), the difference between the highest and lowest relative error (Error-range) and the lowest and highest relative error (Min.-Error and Max.-Error), respectively

We benchmarked several functionals and basis sets to find the best-suited time-dependent (TD) DFT method to analyse the optical properties of a library of different ***iso***-**I**s efficiently. The absorption spectra and the solvatochromism of unfunctionalised ***iso***-**I-45** served as reference for the benchmarking. The solvatochromism [34] of *λ*_max_ in different solvents (toluene, methanol, chloroform, acetonitrile) relative to DMSO was computed at different levels of theory with the respective SMD solvent model [35] and 25 computed states and was compared to the experimental results. The average error of the solvatochromism (defined as “Avg.-Error” in Fig. 3), the average of the absolute deviations of *λ*_max_ in the chosen solvents from experimental results (Abs.-Error), the computing wall time (CalcTime), the difference between the highest and lowest error of the solvatochromism (Error-range), and the lowest and highest error of the solvatochromism (Min.-Error and Max.-Error), were used to select the best suited functional. We aimed for the lowest errors (Avg.-Error, Abs.-Error, Min.-Error and Max.-Error), a uniform description in all solvents (small Error-range) and a computational efficient method (short CalcTime). A selection of the results is given in Fig. 3 and a detailed protocol is presented in the SI. Our study gave the TD-PBE0 [36]/cc-pVDZ [37] level of theory as the best-suited method for the simulations of the UV–Vis spectra of the library. The general applicability regarding excitation energies of TD-PBE0 in intramolecular excited state charge-transfer (CT), as the one observed in ***iso*-I**, was demonstrated earlier [23, 30, 38], even though in some cases TD-PBE0 has shown limitations in describing the CT character of the excitations [38]. In our case, visual inspection of the orbital contribution to the lower excited states confirmed a limited effect of the functional on the nature and order of the states. Therefore, we confidently chose PBE0 to proceed with our analysis. After selection of the functional and basis set, we extended the analysis to our library (see SI).

### Optical properties

Our calculations revealed that all derivatives show the expected *S*_0_ → *S*_1_ *π*–π* vertical excitation (between 480 and 530 nm in methanol). This transition occurs with different probabilities determined by their oscillator strength (*f*). The second vertical transition to the *S*_2_ has, in most cases, a low oscillator strength, making this state a so-called dark state. This finding is in accordance with calculations on ***iso*-I** [24]. Introducing different substituents on the lactam nitrogen influences the position of *λ*_max_ and the oscillator strength of the respective transition. For instance, an ***iso***-**I** with two Boc-groups (***iso*-I-36**) has an unusually high oscillator strength of 0.3019 to the *S*_1_. In contrast, the asymmetrically substituted ***iso*-I**s with only one Boc unit show a bright *π*–π* *S*_0_ → *S*_2_ transition that occurs in ***iso*-I-38** and ***iso*-I-27** with higher probability (*f* = 0.1343 and *f* = 0.1733 for ***iso*-I-38** and ***iso*-I-27** in toluene) than the excitation to the *S*_1_ (*f* = 0.1299 and *f* = 0.1051 for ***iso*-I-38** and ***iso*-I-27** in toluene). Though the *π*–π* transitions in most derivatives were not apparent to be CT from natural transition orbital analysis (see SI), the special case of ***iso*-I-27** showed three consecutive bright and marked CT states. This could be of high interest for optoelectronics, as several excitations and thus a broader spectral range leads to productive charge separation. Moreover, a push–pull configuration like in ***iso-*I-12** (4-methoxyphenyl and 4-nitrophenyl substituents), results in the most bathochromically shifted *λ*_max_ of the library (530 vs. 481 nm of the unsubstituted ***iso*-I-45** and vs. 495 nm of the bis-alkylated reference ***iso*-I-47**, in methanol). All derivatives containing an anisole substituent show an additional aromatic contribution to the frontier orbitals, whereas the frontier orbitals are primarily centred on both the oxindole halves of every other substitution patterns (see SI).

While a push–pull substitution pattern on the nitrogens induced a substantial bathochromic shift of *λ*_max_ (vide supra), the calculations indicated less marked substituent effects at the 6,6′-position. Specifically, ***iso*-I-48** (6,6′-bis-OMe) has the highest oscillator strength of the 6,6′-derivatives, but the lowest *λ*_max_ with *f* = 0.3967 at 467 nm (toluene). By replacing the two electron-donating methoxy moieties by either one or two nitrile substituents, *λ*_max_ increases with simultaneously decreasing *f* over ***iso*-I-49** (*f* = 0.3421 at 478.51 nm in toluene) to ***iso*-I-50** (*f* = 0.2404 at 484.01 nm in toluene). These findings emphasise the challenging balance of absorptivity and electronic transition maximum in the design of new isoindigo chromophores.

**Fig. 4:**
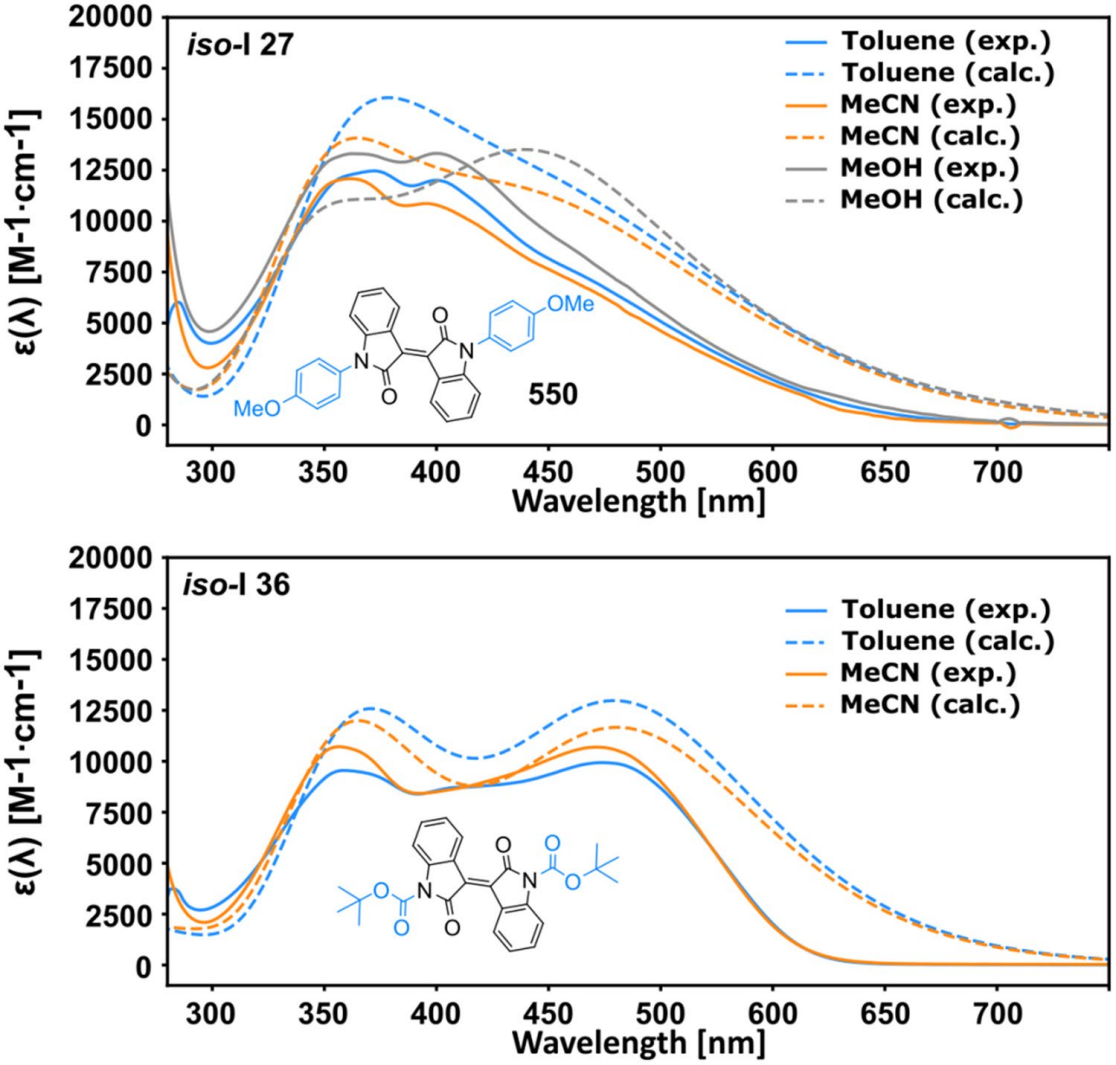
Experimental (solid lines) and calculated spectra (dashed lines) in the respective solvents are in good accordance. Both the vertical excitation energies and the overall shape is met with good accuracy. The *S*_0_ → *S*_1_ transition at ca. 475 nm in ***iso*-I-27** does not correspond to a local maximum in both calculated and experimental spectra. The high attenuation of **iso*-I-36*** was very accurately described by the TD-DFT model, along with the excellent prediction of the vertical excitation energy in toluene, tailing into the NIR

The synthesised ***iso*-I** derivatives were characterised regarding their UV–Vis absorption spectra. As an example, the experimental and predicted spectra of ***iso*-I-27** and ***iso*-I-36** are depicted in Fig. 4 (for all compounds we provided spectra and an excitation analysis in the SI; natural transition orbitals for all synthesised derivatives can be found in the SI). The experimental properties of all ***iso*-I**-derivatives are in good accordance with the calculated results (see Fig. 4) and are summarised in Table 1. In ***iso*-I-27**, the *S*_0_ → *S*_2_ excitation band is used as a reference as the first transition band does not correspond to a local maximum. The experimental spectra confirmed the discussed behaviour of Boc-*N,N′*-substitution based on the theoretical results (vide supra). ***iso*-I-27** shows the increased transition probability to the *S*_2_ and ***iso*-I**-**36** yields the highest attenuation coefficient for the *π*–π* transition, while the remaining derivatives show high and distinct attenuation coefficients at *λ*_max_ with broad absorption bands ranging up to ca. 600 nm.

**Table 1:** Comparison of experimental and theoretical optical and electrochemical properties

***iso*-I**	Solvent	*λ*_max_ [nm]		Attenuation at *λ*_max_		*E*1/2 [V] (*V* vs Fc/Fc^+^)	Exciton binding energy_calc._ [eV]
		Exp	Calc	*f*_calc_	*ε*_exp_ [M^−1^ cm^−1^]		
**25**	Toluene	508	508	0.1422	2882		
	MeCN	503	508	0.1366	2596	− 1.12^a^	4.30
	MeOH	512	526	0.0932	1936		
**27**	Toluene^b^	400	449	0.1733	12,006		
	MeCN^b^	396	451	0.1655	10,865	− 1.01^a^	4.18
	MeOH^b^	402	459	0.2239	13,313		
**30**	Toluene	484	492	0.1748	4962		
	MeCN	485	497	0.1496	5277	− 1.13	4.30
	MeOH	494	504	0.1650	4046		
**31**	Toluene	485	490	0.1999	3985		
	MeCN	482	493	0.1712	3501	− 1.11	4.69
	MeOH	483	497	0.174	2783		
**35**	Toluene	487	486	0.1995	4689		
	MeCN	483	490	0.1783	4773	− 1.15^a^	4.64
	MeOH	482	497	0.1821	2780		
**36**	Toluene	474	487	0.3019	9935		
	MeCN	472	487	0.2727	10,695	− 0.90	4.82
**45**	Toluene	484	482	0.3967	5105		
	MeCN	478	487	0.1884	4208	− 1.16^a^	4.74
	MeOH	481	496	0.1870	4473		
**46**	Toluene	489	496	0.1492	5198		
	MeCN	483	501	0.1286	4440	− 1.19	4.63
	MeOH	485	509	0.1256	4067		
**47**	Toluene	493	501	0.1594	4154		
	MeCN	493	507	0.1361	3784	− 1.20	4.56
	MeOH	495	515	0.1330	3671		

The experimentally obtained *λ*_max_ and the corresponding molar attenuation coefficient (*ε*_exp_) are compared to the calculated *S*_0_ → *S*_1_ transition properties at the TD-PBE0/cc-pVDZ level of theory. The value of *E*_1/2_ obtained by cyclic voltammetry in acetonitrile are referenced versus Fc/Fc^+^. The exciton binding energy was obtained from the analysis of obtained data with the Multiwfn software. A complete list of computed optical and electrochemical data can be found in the SI

^a^Determined from computational results, corrected by the parametrisation scheme described below

^b^*λ*_max_ as *S*_0_–*S*_2_ transition band, since the *S*_0_–*S*_1_ transition does not build up to a local maximum, leading to a formal increased error

**Fig. 5:**
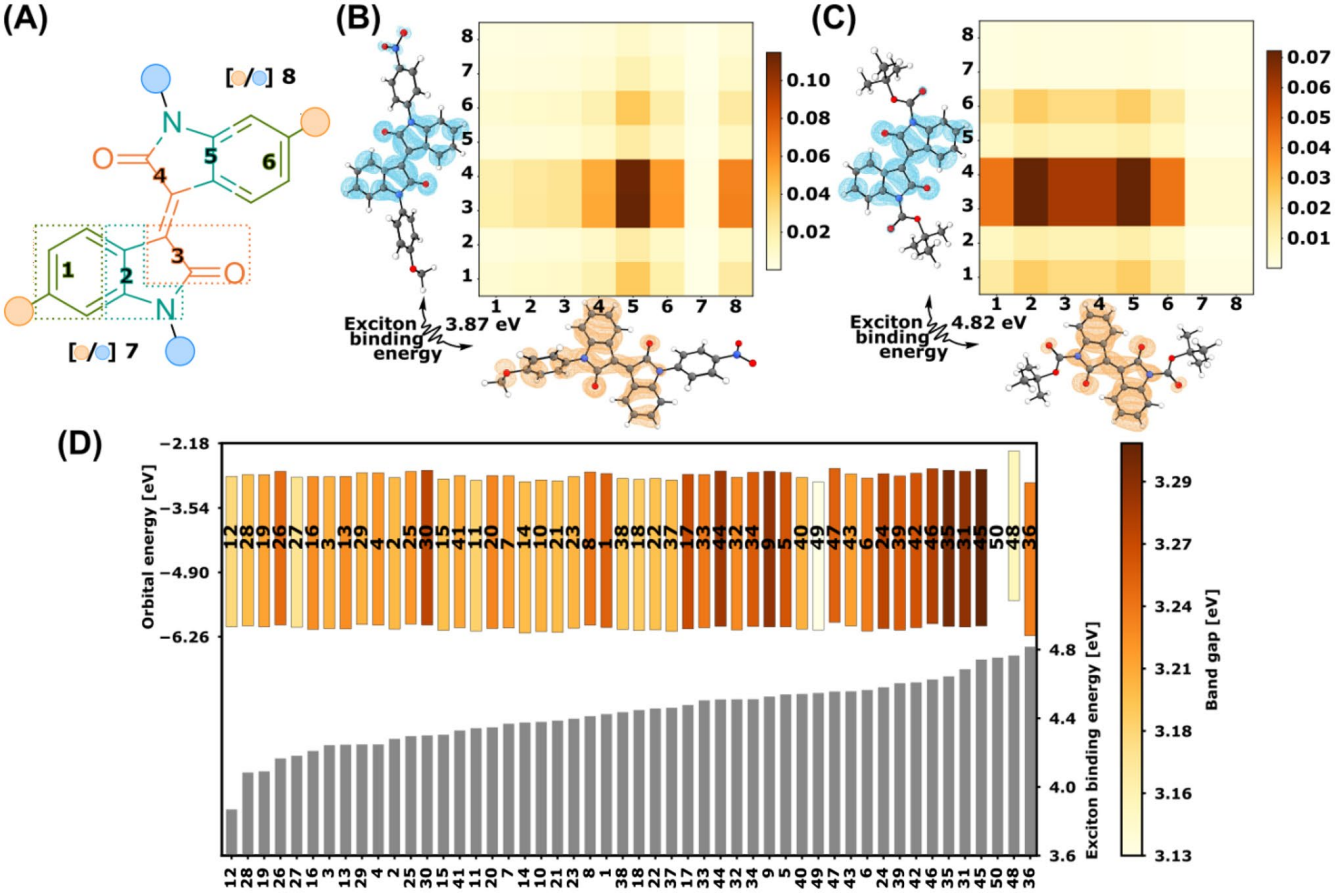
**A** Fragments used to visualise and analyse the transition density matrices (TDMs). **B** An asymmetric TDM can be observed in the strongly mesomeric polarised ***iso*-I-12** with its low exciton binding energy of 3.87 eV. **C** The symmetric inductive effect dominates ***iso*-I-36**, which shows a lower charge transfer with partial local excitation and a high exciton binding energy of 4.82 eV. **D** The orbital energies *E*_HOMO_ (lower end of coloured bars) and *E*_LUMO_ (upper end of coloured bars) mark the lower and upper bound of the band gap (coloured bars). The band gap of the *N*,*N*′-library correlate to the exciton binding energies (grey bars), while the 6,6′-substituted compounds (***iso*-I-48–50**) does not follow this trend (*E*_HOMO_ and *E*_LUMO_ of derivative 50, out of range with − 0.558 and 0.928 eV).

To understand the substituent effects on the nature of the electronic transition, we proceeded to analyse the library using transition density matrices (TDMs). This treatment allows accessing the electron and the hole distribution in the excited state and identifying their delocalisation [39]. In this way, one can visualise electronic excitation processes such as CT [39], which is of direct relevance in optoelectronic materials [24, 40, 41]. For this, we divided each compound into fragments to facilitate the interpretation of the results (Fig. 5A). The choice of the fragments allows us to ascribe the off-diagonal elements to CT and diagonal elements to local excitations. We used the Multiwfn software to analyse the TDMs and the exciton binding energies [42]. Inspecting the TDMs, we can assign four major transition behaviours to the first excited state. Symmetric and asymmetric TDMs, which can be further categorised by the presence of solely inductive or additional mesomeric effects. In symmetric TDMs, both the electron and the hole share a similar distribution around the central double bond, spreading on both oxindole halves of the molecule. On the contrary, asymmetric TDMs have a hole located on one half of the structure. The electron for all S_0_ → S_1_ TDMs is localised on fragments 3 and 4 (Fig. 5A).

The presence of electron-donating and -withdrawing groups interacting with the hole is the origin of the asymmetry in the TDMs. The donation of electron density to the hole via mesomeric effects by an electron donating moiety distorts the hole location. In this case, the TDM can be categorised as mesomerically influenced (Fig. 5B), rather than indirectly affected by only inductive effects (see SI). Vice versa, the inductively dominated TDM in Fig. 5C shows no localisation of the hole on one oxindole-half, without any significant TDM-elements on the substituent. The mesomeric contribution to the TDM is highly dependent on the polarity difference induced by the *N*,*N*′-substituents. The strong polarisation induced by the anisole and nitrophenyl substitution, and the mesomeric effects in ***iso*-I**-**12** lead to a significant hole-density at the *N,N*′-substituents (Fig. 5B). The unpolarised, bis-anisole substituted ***iso*-I**-**25** on the other hand does barely show any hole-density at the position of the substituent (see SI). The findings related to the enhanced resonance between the hole density and the anisole in ***iso*-I**-**12** are in contrast to other examples where the rotation of the phenyl ring prevented an effective overlap, a trend encountered in 6,6′-substituted patterns [19, 24].

The known methodological limitation of the analysis of the excited state character and the excitation process [43, 44] can be observed from the electron and hole-densities in Fig. 5B, C. The CT is not always apparent from electron–hole or natural transition orbital analysis alone. This is due to the poorly defined localisation of the electron density around electron-donating and -withdrawing groups [38]. The TDM analysis, however, aided the identification of the CT and the substitution effects.

Next, we focused on the exciton binding energies, which represent the Coulomb attraction between the exciton quasiparticles (electron and hole). It is a measure for the separability of the exciton in free charges and is directly related to the generation of an effective current in optoelectronics [41]. The strong polarisation and the mesomeric effects in ***iso*-I**-**12** (Fig. 5B) significantly lower the exciton binding energy to 3.87 eV, compared to 4.82 eV in the symmetric ***iso*-I**-**36** (see Fig. 5B, C, along with Table 1). Analysing the HOMO and LUMO energies (*E*_HOMO_ and *E*_LUMO_) provides further information on the effects of the *N,N*′-substitution on the electronic structure properties (see Fig. 5D). The variation of *E*_HOMO_ and *E*_LUMO_ in the *N,N*′-substituent library of 0.245 and 0.299 eV is more marginal than the one found in the 6,6′-substituted compounds (5.55 and 3.92 eV).

We further investigated the band gaps (*E*_HOMO_–*E*_LUMO_) of the *N,N*′-library varying by a range of 0.136 eV, with a maximum of 3.309 eV in the unsubstituted ***iso*-I-45** and a minimum of 3.173 eV in ***iso*-I-26**. Investigation of the band gap and the exciton binding energy reveals evidence of a direct correlation between the two. The band gap increases with rising exciton binding energy (see Fig. 5D, all the compounds apart from ***iso*-I-48–50**, which belong to the 6,6′-substitution pattern). While some derivatives are outliers in this trend (e.g. ***iso*-I**-**26**, **30**, **40**), this finding indicates that the exciton binding energy correlates to the band gap of the *N,N*′*-*library, specifically. The 6,6′-substituted ***iso*-I** derivatives ***iso*-I-48–50** are outliers in this series. ***iso*-I**-**49** shows the second smallest band gap of the library (3.13 eV) while being positioned in the upper third of the library regarding the exciton binding energy. Additionally, ***iso*-I**-**50** shows the lowest band gap in the series (0.371 eV), but is characterised by the third-highest exciton binding energy of 4.75 eV. These examples, combined with the above-mentioned variation in the *E*_HOMO_ and *E*_LUMO_, emphasise further that 6,6′*-*substitution patterns cannot be categorised following the same trend observed for *N,N*′-substituted ***iso*-I** derivatives.

### Electrochemical properties

**Fig. 6:**
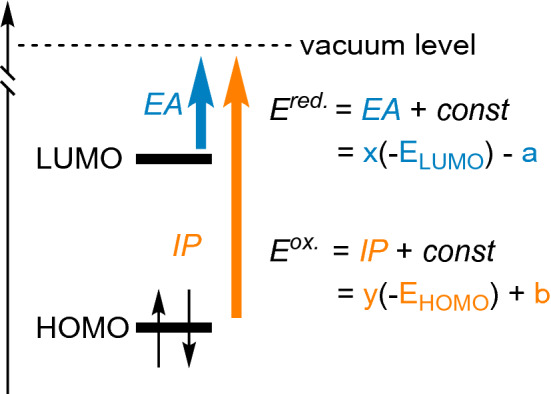
*E*_HOMO_ and *E*_LUMO_ can be approximated to electron affinities (EA) and ionisation potential (IP). The reduction and oxidation potentials can be obtained from the EA and IP

According to Koopmans’ theorem [45], *E*_HOMO_ and *E*_LUMO_ can be approximated to the ionisation potential (IP) and electron affinity (EA), respectively (Fig. 6).

**Fig. 7:**
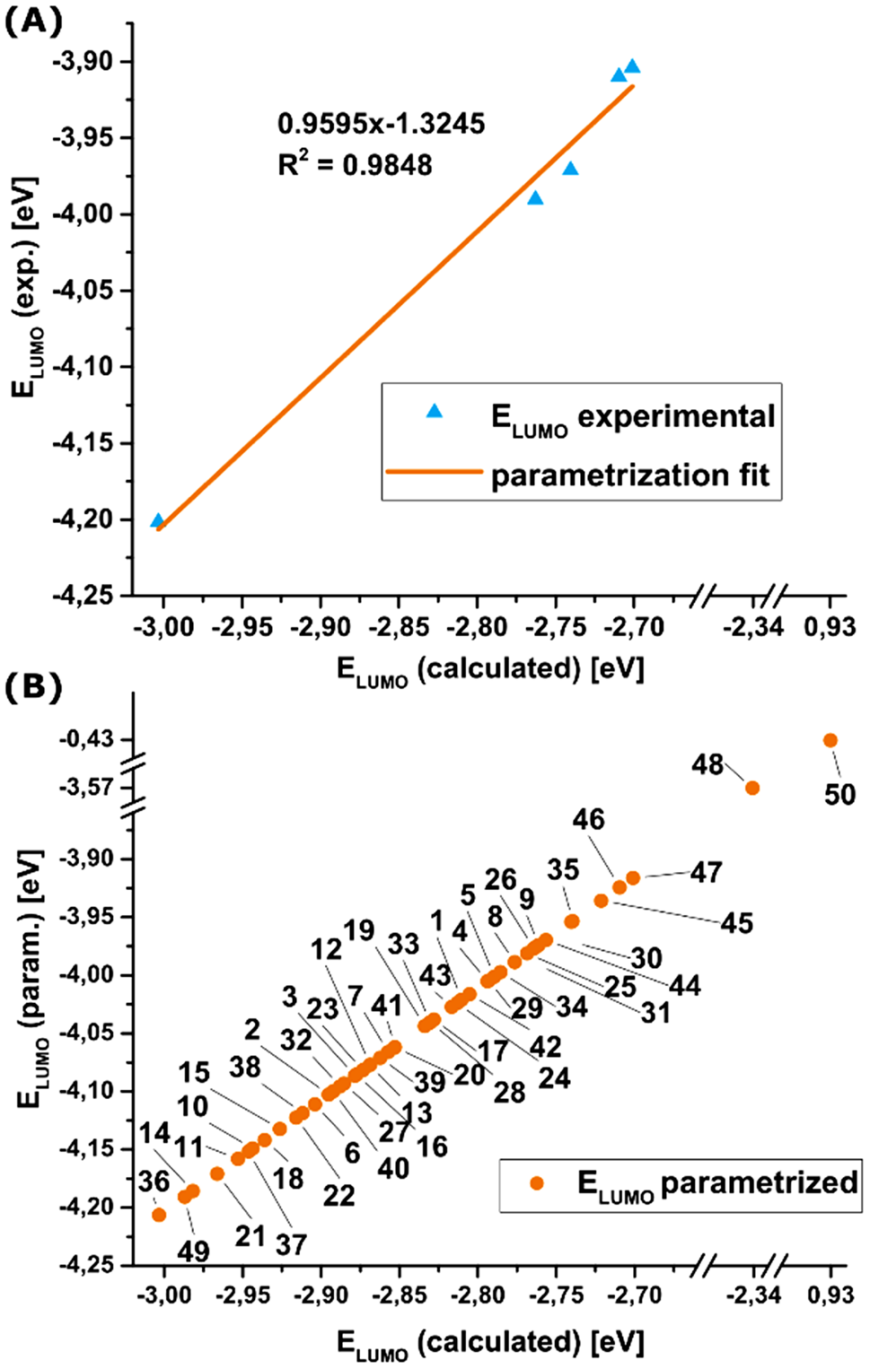
**A** Parametrisation of the calculated *E*_LUMO_s with an experimental subset using a previously described method. **B** Application of the parametrisation to the library. While the *N,N*′-library spans a very local energy range, the 6,6′-library shows more pronounced changes in the *E*_LUMO_ and thus its reduction potentials. The 6,6′-substitution pattern does not follow the same trend as the *N,N*′-substitution pattern regarding the *E*_LUMO_ (see discussion in the text). Thus, we did not further analyse the 6,6′-substitution patterns with the method mentioned above, but we included the parametrised values only for a general overview.

In our study, we decided to utilise the linear dependency of the EA and the reduction potential combined with Koopmans’ theorem to apply a parameterisation method on the computed values. We derived the *E*_LUMO_s from experimental reduction potentials ($E_{{{\text{LUMO}}}}^{{{\text{exp}}.}}$) of a small sub-library of compounds that we use to parametrise the complete dataset of *E*_LUMO_s obtained computationally ($E_{{{\text{LUMO}}}}^{{{\text{calc}}.}}$). This approach is extensively described in the literature [24, 46–50]. We derived $E_{{{\text{LUMO}}}}^{{{\text{exp}}.}}$s from the reduction half-wave potential (*E*_1/2_) versus Fc/Fc^+^, obtained by cyclic voltammetry in acetonitrile, by applying Eq. (1), using the value of 5.1 eV vs. vacuum for ferrocene [47]. The experimental values were plotted against the theoretical ones and the parameters of the relation between $E_{{{\text{LUMO}}}}^{{{\text{exp}}.}}$ and $E_{{{\text{LUMO}}}}^{{{\text{calc}}.}}$ were obtained by linear regression (Fig. 7). Applying Eq. (1), we inferred the parametrisation coefficients (cf. x and a in Fig. 6) to correct the calculated results via Eq. (2) to better analyse the reduction potentials in the library.

1 $$E_{{{\text{LUMO}}}}^{{{\text{exp}}{\text{.}}}} = - \left( {E_{{1/2}}^{{{\text{red}}{\text{.}}}} + 5.1} \right)\;~\left[ {{\text{eV}}} \right],$$

2 $$E_{{1/2}}^{{{\text{red}}{\text{.}}}} = - (0.9595~E_{{{\text{LUMO}}}}^{{{\text{calc}}{\text{.}}}} - 1.3245) - 5.1)~\;\left[ {V~{\text{vs~Fc}}/Fc^{ + } } \right].$$

A correlation between *N,N*′-substitution pattern and the *E*_LUMO_ is observed. The *N,N′*-substituted ***iso*-I** derivatives show more stable LUMOs (lower energy relative to the vacuum level) with electron-withdrawing groups and more destabilised ones by introducing electron-donating groups. Contrary to the considerations taken concerning the TDMs and the exciton binding energy, we do not observe a significant difference from inductive or mesomeric effects. Applying Koopmans’ theorem, these findings were used to obtain an accurate prediction of the reduction potentials. Electron-withdrawing groups increase (***iso-*I-36** *E*_1/2_(param./exp.) = − 0.89 V/− 0.90 V), while weakly electron-donating groups decrease the reductions potential (***iso-*I-47** *E*_1/2_(param./exp.) = − 1.20 V/− 1.18 V). While the electron-donating bis-anisole substituted ***iso*-I**-**25** shows a higher reduction potential (*E*_1/2_(param.) = − 1.12 V) and thus seems to oppose the trend, the push–pull configurations support the findings, as their reduction potentials range is in between the pull–pull and push–push configurations (e.g. *E*_1/2_(param.) = − 1.02 V, − 1.06 V and − 1.06 V for ***iso-*I-12**, ***iso-*I-19** and ***iso-*I-28**).

As the reduction potential is directly linked to the open-circuit voltage of photovoltaics [41], our method could aid the design of enhanced photovoltaic materials.

## Conclusion

In this work, we conducted a detailed investigation into the substitution effects on the *N,N*′-site of ***iso-*I**, providing an alternative for optical and electronic tuning to the already well-studied 6,6′-site. To achieve a comprehensive study of a broad range of substituents, a combination of computational and experimental methods on a library of molecules was applied and a subset of these molecules was synthesised to allow for parametrisation.

TD-PBE0/cc-pVDZ emerged as the best-suited method to simulate the experimental properties of isoindigo, reaching a good agreement between the simulated and calculated ones. Based on the computational data, we discovered trends relating the optical properties to the substitution patterns. Notably, derivatives ***iso*-I-12** and **36** showed the highest *λ*_max_ and *ε*(*λ*_max_)_,_ respectively. The otherwise typical dark *S*_2_ state could be turned into a bright one by asymmetric substitution with one Boc and one other substituent tested.

The analysis of the excitation behaviour by TDMs elucidated the difference between symmetric and asymmetric hole distributions and inductive and resonance contributions to the hole-density. In mesomeric push–pull TDMs we observed a significant contribution of anisole moieties to the hole-density, in contrast to related 6,6′-***iso*-Is**.

The analysis of the exciton binding energy showed that strongly polarised derivatives have lower exciton binding energies, with ***iso-*I-12** having the lowest exciton Coulomb interaction of 3.87 eV, and the unpolarised ***iso-*I-36** showing the highest (4.82 eV). We discovered a relation in the *N,N*′*-*library between the band gap and the exciton binding energy. The lower the band gap, the lower the Coulomb attraction between the electron and the hole. However, this trend does not hold for the 6,6′-substitution patterns.

Finally, a parametrization method for E_LUMO_ was employed to obtain accurate predictions for the reduction potentials of the whole library. We can conclude from the observed trend in *E*_LUMO_ that electron-withdrawing (Boc) groups give rise to low *E*_LUMO_s, while the propyl substituted ***iso-*Is** have the highest *E*_LUMO_s of the *N,N*′*-*library.

The method here reported allows to obtain the reduction potentials for a library of 47 derivatives, utilising a limited subset of experimental data in combination with computational available data. We could enhance the prediction by discovering trends [51] towards the accurate calculation of electrochemical properties. Furthermore, our data did elucidate a relationship between electron-donating and -withdrawing groups to decrease and increase the reduction potential in the *N,N*′-substitution patterns. This approach shows potential to be useful for rational design and library screening of similar compounds.

In conclusion, we successfully elucidated several *N,N*′-substitution effects in isoindigo, which shows potential for more extensive use in material sciences. Taking ***iso-*I-27** as an example, the three consecutive CT states, in combination with one of the lowest exciton binding energies of 4.18 eV, and a low band gap could enable broad spectral operating OPV-materials. We expect that this study will foster more detailed research on the electronic structure of substituted isoindigos. Additionally, we see major potential in the use of *N,N*′-substitution for optical and electronic tuning in cases where the 6,6′-site is already occupied [52].

## Supplementary Information

Below is the link to the electronic supplementary material.

Supplementary file1Supporting Information containing synthetic procedures, characterisation of novel compounds, a full set of spectroscopic and electrochemical studies, details regarding DFT calculations is available free of charge (DOCX 44221 KB)

Supplementary file2 (ZIP 5602 KB)
