# Supplementary material for: Predicting the substituent effects in the optical and electrochemical properties of N,N′-substituted isoindigos
Source: Photochem Photobiol Sci. 2021 Jul 5;20(7):927–38. doi: 10.1007/s43630-021-00071-5 (PMC8550769; doi:10.1007/s43630-021-00071-5)
Supplement: Supplementary file 1 — Supplementary file1Supporting Information containing synthetic procedures, characterisation of novel compounds, a full set of spectroscopic and electrochemical studies, details regarding DFT calculations is available free of charge (DOCX 44221 KB) [file 43630_2021_71_MOESM1_ESM.docx]

**Supporting information**

**Predicting the Substituent Effects in the Optical and Electrochemical Properties of N,N'-substituted Isoindigos**

Ferdinand L. Kiss,^[a,b]^ Brian P. Corbet,^[a]^ Nadja A. Simeth,^[a]^ Ben L. Feringa^[a],*^ and Stefano Crespi^[a],*^

^[a]^ Stratingh Institute for Chemistry, Faculty for Science and Engineering, University of Groningen, Nijenborgh 4, 9747 AG Groningen, The Netherlands.

^[b]^Current address: Department Chemie, Ludwig-Maximilians-Universität München, D-81377 München, Germany.

*s.crespi@rug.nl, b.l.feringa@rug.nl

[Experimental Section 2](#_Toc70590428)

[1. NMR and HRMS spectra 14](#_Toc70590429)

[2.TD-DFT Library 36](#_Toc70590430)

[2.1 Benchmarks 36](#_Toc70590431)

[2.2 Geometries 38](#_Toc70590432)

[2.3 Frontier Orbitals 125](#_Toc70590433)

[2.4 Vertical excitation properties 148](#_Toc70590434)

[2.5 Comparison experimental and theoretical spectra 163](#_Toc70590435)

[2.6 Simulated spectra 169](#_Toc70590436)

[2.7 Transition density matrix analysis 169](#_Toc70590437)

[2.8 Koopmans theorem parametrization 169](#_Toc70590438)

[3. Electrochemistry 171](#_Toc70590439)

[References 172](#_Toc70590440)

# Experimental Section

**Chemicals**

Reagents were purchased from Sigma-Aldrich, Alfa Aesar, TCI, Boom and Combi-Blocks, and used without further purification. Dry solvents were collected from a Pure Solve MD5 solvent dispenser from Demaco or by drying them for 48 h over 3 Å molecular sieves.

**General methods**

All reactions were performed on magnetic stirrers. For dry or oxygen-free reactions, standard Schlenk technique was used employing nitrogen as inert gas, if not otherwise stated. Extraction was conducted by liquid-liquid extraction with stated solvents with at least three times the reaction volume.

**Chromatography**

Flash chromatography was performed on silica gel (Screening devices B.V.) with a particle size of 40−64 μM and pore size of 60 Å or on Buchi EcoFlex silica columns (4 -25 g prepacked columns, 60 Å) using a Buchi Reveleris® X2 system. TLC analysis was conducted on TLC aluminum plates coated with a silica gel matrix (Supelco, silica gel 60) with detection by UV (254 nm or 366 nm) or suitable staining.

**NMR spectroscopy**

^1^H NMR and ^13^C NMR were recorded in DMSO-*d6* and Chloroform-*d* with the chemical shift (δ) relative to the solvent peak on an Agilent Technologies 400-MR (400/54 Premium Shielded) spectrometer (400 MHz). All spectra were measured at room temperature (22–24 °C). The spectra were analysed using *MestReNova* from *Mestrelab research S.L.*

**Mass spectrometry**

High resolution mass spectra were recorded on a Thermofisher LTQ Orbitrap XL with eluent MeOH (0.1% TFA) and flow rate of 0.15 mL min^-1^ in positive (ACPI/ESI) mode.

**UV-vis analysis**

For a typical experiment, a stirred solution (2 mL) of a compound was prepared. For all experiments, the temperature was maintained at stated temperatures using a Quantum Northwest TC1 temperature controller. Electronic absorption spectra were measured using an Agilent 8453 UV-Vis spectrometer. Raw data was processed using Agilent UV-Vis ChemStation B.02.01 SP1, Spectragryph 1.2, and Origin 2018.

**Electrochemistry**

Cyclic voltammograms (CVs) were recorded using a three-electrode setup on a VSP-300 potentiostat/galvanostat (Biologic Science Instruments) using an Ag/AgCl wire as pseudo-reference electrode (RE), freshly polished glassy carbon (3 mm diameter) disc working electrode (WE) and a platinum wire as counter electrode (CE). All CVs were recorded at room temperature, a scan speed 0.1 V/s under an argon atmosphere in acetonitrile, degassed by bubbling argon (>2 min prior to measurement) and tetrabutylammonium hexafluorophosphate (0.1 M) as supporting electrolyte. All CVs were referenced to added ferrocene as internal reference.

**Melting point analysis**

Melting point ranges were determined on a Stuart analogue capillary melting point SMP11 apparatus.

**Procedures**

*(E)-Isoindigo (****iso*-I-45***)*

***iso*-I-45** was synthesized following a literature procedure [1]. Isatin (414 mg, 2.81 mmol, 1.03 equiv.) and 2-oxindole (364 mg, 2.73 mmol, 1.00 equiv.) were dissolved in glacial acetic acid (4.0 mL) and 4 drops of conc. HCl were added. The solution was stirred at 120 °C for 16 h and slowly cooled to room temperature. The precipitate was filtered off and rinsed with water and pentane. The solids were dried *in vacuo* yielding the title compound [(556 mg, 2.10 mmol, 77%)](https://mbook.housing.rug.nl/ELN/101041) as a dark violet powder.

**^1^H NMR** (400 MHz, DMSO-*d*_6_) δ = 10.88 (s, 1H), 9.06 (dd, *J* = 8.1, 1.2 Hz, 1H), 7.34 (td, *J* = 7.6, 1.2 Hz, 1H), 6.96 (td, *J* = 7.8, 1.2 Hz, 1H), 6.84 (d, *J* = 7.5 Hz, 1H).

**HR-MS** (ESI), [M+H]^+^, for C_16_H_11_N_2_O_2_^+^ m/z calcd. 263.08150, found 263.08193.

**R_f_** (SiO_2_, Petroleum ether 40-60:EtOAc = 50:50): 0.68.

**Melting Point** >250 °C.

The analytical data are in agreement with published data [2].

*Di-tert-butyl 2,2'-(2,2'-dioxo-[3,3'-biindolinylidene]-1,1'-diyl)(E)-diacetate (****iso*-I-31***)*

A 50-mL crimp top vial was charged with isoindigo (***iso*-I-45**) (72 mg, 0.27 mmol, 1.00 equiv.), Cs_2_CO_3_ (222 mg, 0.68 mmol, 2.50 equiv.), DMF (360 µL) and *tert*-butyl 2-bromoacetate (0.24 mL, 1.64 mmol, 6.00 equiv.). The vial was sealed and the mixture was stirred at room temperature for 15 h. The resulting mixture was diluted with EtOAc, washed twice with H_2_O and the aqueous layer was extracted twice with methylene chloride. The combined organic layers were dried over Na_2_SO_4_ and subsequently concentrated *in vacuo* yielding a red solid. Flash column chromatography (SiO_2_; EtOAc:Petroleum ether 40-60; 1:3) and drying *in vacuo* afforded the title compound as red solid (70 mg, 0.14 mmol, 53%).

**^1^H NMR** (400 MHz, Chloroform-*d*) δ = 9.20 (d, *J* = 8.0 Hz, 2H), 7.35 (t, *J* = 7.7 Hz, 2H), 7.07 (t, *J* = 7.8 Hz, 2H), 6.68 (d, *J* = 7.8 Hz, 2H), 4.46 (s, 4H), 1.46 (s, 18H).

**^13^C NMR** (101 MHz, Chloroform-*d*) δ = 166.7, 148.6, 144.3, 132.6, 130.3, 122.8, 121.8, 107.9, 82.9, 77.5, 77.16, 76.8, 42.3, 28.2.

**HR-MS** (ESI), [M+Na]^+^, for C_28_H_30_N_2_O_6_Na^+^, m/z calcd. 513.19961, found 513.19834.

**Melting Point** >250 °C.

**R_f_** (SiO_2_; EtOAc:Petroleumether 40-60; 50:50): 0.86.

*Di-tert-butyl (E)-2,2'-dioxo-[3,3'-biindolinylidene]-1,1'-dicarboxylate (****iso*-I-36***)*

To a solution of di-*tert*-butyl dicarbonate (410 mg, 1.88 mmol, 1.95 equiv.) in 1 mL of THF (1 mL) at ambient temperature was added isoindigo (***iso*-I-45**) (255 mg, 0.96 mmol, 1.00 equiv.) in THF (17 mL) dropwise over 30 min *via* a dropping funnel. DMAP (23.5 mg, 0.193 mmol, 0.200 equiv.) was added and the mixture was stirred at room temperature for 18 h. The volatiles were evaporated at 75 °C and the solid residue was diluted in EtOAc and washed with 1 M NH_4_Cl solution twice. The aqueous layer was extracted once with methylene chloride. Drying over Na_2_SO_4_, evaporation of the solvent and drying at high vacuum gave the title compound as red solid (376 mg, 0.81 mmol, 84%).

**^1^H NMR** (400 MHz, Chloroform-*d*) δ = 8.96 (dd, *J* = 8.2, 1.3 Hz, 2H), 7.81 (dt, *J* = 8.3, 0.7 Hz, 2H), 7.43 (ddd, *J* = 8.5, 7.5, 1.3 Hz, 2H), 7.17 (ddd, *J* = 8.4, 7.5, 1.2 Hz, 2H), 1.68 (s, 18H).

**Melting Point** >250 °C.

**R_f_** (SiO_2_; EtOAc:Petroleumether 40-60; 1:1): 0.94.

The analytical data are in agreement with published data.

*1-(4-methoxyphenyl)indolin-2-one (****3****)*

Compound **3** was synthesized following a literature procedure [3]. A crimp top vial was charged under nitrogen counterflow with CuI (29 mg, 0.15 mmol, 0.05 equiv.), oxindole (488 mg, 3.67 mmol, 1.20 equiv.), 4-iodoanisol (715 mg, 3.06 mmol, 1.00 equiv.), K_2_CO_3_ (844 mg, 6.11 mmol, 2.00 equiv.). The reaction vessel was sealed and flushed with nitrogen three times. Dry 1,4-dioxane (3.0 mL) and DMEDA (0.05 mL, 0.46 mmol, 0.15 equiv.) were added and the reaction mixture was stirred for 22 h at 110 °C. The reaction mixture cooled to room temperature under continuous stirring for 3 h. The crude mixture was filtered through a silica plug and washed with EtOAc. The filtrate was concentrated *in vacuo* and was purified by flash column chromatography (SiO_2_; EtOAc: Petroleum ether 40-60; 2:8 to 4:8). Evaporation of the solvents and drying at high vacuum resulted the title compound as a colourless solid (85 mg, 0.354 mmol, 12%).

**^1^H NMR** (400 MHz Chloroform-*d*) δ = 7.34 – 7.28 (m, 3H), 7.20 (td, *J* = 7.8, 1.1 Hz, 1H), 7.09 – 7.02 (m, 3H), 6.73 (d, *J* = 7.9 Hz, 1H), 3.86 (s, 3H), 3.70 (s, 2H).

**^13^C NMR** (101 MHz, Chloroform-*d*) δ = 174.9, 159.3, 145.8, 128.1, 127.9, 127.2, 124.7, 124.4, 122.8, 115.1, 109.4, 55.7, 36.1.

**R_f_** (SiO_2_; EtOAc:Petroleum ether 40-60; 50:50): 0.61.

The analytical data are in agreement with published data [3].

*(E)-1-(4-methoxyphenyl)-[3,3'-biindolinylidene]-2,2'-dione (****iso*-I-30***) and* (*E)-1,1'-bis(4-methoxyphenyl)-[3,3'-biindolinylidene]-2,2'-dione (****iso*-I-25***)*

Isatin (22 mg, 0.15 mmol, 1.4 equiv.) and **3** (25 mg, 0.1 mmol, 1.0 equiv.) were dissolved in glacial acetic acid (0.2 mL) and 2 drops of conc. HCl were added. The solution was stirred at 120 °C for 2 h turning dark red. The reaction mixture was diluted with EtOAc and washed with water three times. The organic phase was dried over Na_2_SO_4_ and subsequently *in vacuo*. Purification by flash column chromatography (SiO_2_; EtOAc: Petroleum ether 40-60; 1:3) yielded both ***iso*-I-30** (17 mg, 0.05 mmol, 43%) and ***iso*-I-25** (3 mg, 0.01 mmol, 4 %) as red solids.

***iso*-I-30**:

**^1^H NMR** (400 MHz, Chloroform-*d*) δ = 9.18 (d, *J* = 4.9 Hz, 1H), 9.16 (d, *J* = 5.2 Hz, 1H), 7.54 – 7.52 (m, 2H), 7.38 – 7.27 (m, 3H), 7.13 – 7.04 (m, 3H), 7.00 (td, *J* = 7.9, 7.5, 1.2 Hz, 1H), 6.81 (d, *J* = 8.2 Hz, 1H), 6.68 (d, *J* = 8.3 Hz, 1H), 3.88 (s, 3H).

**^13^C NMR** (101 MHz, Chloroform-*d*) δ = 132.8, 132.6, 130.6, 130.0, 128.6, 122.9, 122.6, 115.2, 109.3, 109.2, 77.5, 77.2, 76.8, 55.7.

**HR-MS** (ESI), [M+H]^+^, for C_23_H_17_N_2_O_3_^+^, m/z calcd. 369.12337, found 369.12306.

**R_f_** (SiO_2_; EtOAc:Petroleum ether 40-60; 1:1): 0.70.

***iso*-I-25:**

**^1^H NMR** (400 MHz, Chloroform-*d*) δ = 9.22 (d, *J* = 8.1 Hz, 2H), 7.41 – 7.32 (m, 4H), 7.29 (dd, *J* = 7.7, 1.2 Hz, 2H), 7.10 – 7.06 (m, 4H), 7.03 (td, *J* = 7.9, 1.2 Hz, 2H), 6.69 (d, *J* = 7.6 Hz, 2H), 3.88 (s, 6H).

**HR-MS** (ESI), [M+H]^+^, for C_30_H_23_N_2_O_4_^+^, m/z calcd. 475.16523, found 475.16380.

**R_f_** (SiO_2_; EtOAc:Petroelum ether 40-60; 1:1): 0.92.

Due to the limited amount of material, further analysis was not performed.

*Tert-butyl 2-(2,3-dioxoindolin-1-yl)acetate (****1****)*

A crimp top vial was charged with isatin (505 mg, 3.43 mmol, 1.00 equiv.), Cs_2_CO_3_ (2.35 g, 7.21 mmol, 2.10 equiv.), DMF (2.0 mL) and *tert*-butyl bromoacetate (2.0 mL, 13.7 mmol, 4.00 equiv.). The vial was sealed and the mixture was stirred at room temperature for 15 h. The resulting mixture was diluted with EtOAc, washed H_2_O three times and the aqueous layer was extracted once with EtOAc. The combined organic layers were dried over Na_2_SO_4_ and the filtrate was concentrated yielding an orange oil. Flash column chromatography (SiO_2_; EtOAc:Petroleum ether 40-60; 1:1) gave the title compound (685 mg, 2.62 mmol, 76%) as a dark orange crystalline solid.

**^1^H NMR** (400 MHz, Chloroform-*d*) δ = 7.64 (ddd, *J* = 7.4, 1.4, 0.6 Hz, 1H), 7.59 (td, *J* = 7.8, 1.3 Hz, 1H), 7.15 (td, *J* = 7.6, 0.8 Hz, 1H), 6.77 (d, *J* = 8.0 Hz, 1H), 4.39 (s, 2H), 1.46 (s, 9H).

**^13^C NMR** (101 MHz, Chloroform-*d*) δ = 182.8, 165.9, 158.2, 150.7, 138.5, 125.7, 124.2, 117.8, 110.3, 83.6, 77.5, 77.2, 76.8, 42.2, 28.1.

**HR-MS** (ESI), [M+Na]^+^, for C_14_H_15_NO_4_Na^+^, m/z calcd. 284.08933, found 284.08993.

The analytical data are in agreement with published data [4].

*Tert*-butyl (*E*)-2-(2,2'-dioxo-[3,3'-biindolinylidene]-1-yl)acetate (***iso*-I-35**)

Compound **1** (44 mg, 0.17 mmol, 1.00 equiv.) and [2-oxindole (22 mg, 0.17 mmol, 1.00 equiv.)](https://mbook.housing.rug.nl/ELN/125219) were dissolved in [glacial acetic acid (1.0 mL)](https://mbook.housing.rug.nl/ELN/125222) and 2 drops of HCl were added. The mixture was stirred at reflux for 4.5 h and 16 h at room temperature. The reaction mixture was filtered and washed with an excess of water and pentane and carefully with EtOAc. The residue was dried *in vacuo* and gave the title compound as red solid.

**^1^H NMR** (400 MHz, DMSO-*d*_6_) δ = 10.94 (s, 1H), 9.15 – 9.08 (m, 1H), 9.02 (d, *J* = 8.0 Hz, 1H), 7.41 (td, *J* = 7.7, 1.2 Hz, 1H), 7.36 (td, *J* = 7.6, 1.2 Hz, 1H), 7.10 – 7.02 (m, 2H), 6.97 (td, *J* = 7.8, 1.2 Hz, 1H), 6.85 (dd, *J* = 7.9, 1.1 Hz, 1H), 4.58 (s, 2H), 3.32 (s, 9H).

**^13^C NMR** (101 MHz, DMSO-*d*_6_) δ = 169.2, 168.8, 167.4, 144.4, 144.1, 134.2, 133.1, 132.5, 131.6, 129.4, 129.1, 121.9, 121.6, 121.2, 120.8, 109.7, 108.7, 41.2, 40.2, 40.0, 39.9, 39.7, 39.5, 39.3, 39.1, 38.9.

**Melting Point** >250 °C.

No yield was measured.

*Tert-*butyl (*E*)-1'-(4-methoxyphenyl)-2,2'-dioxo-[3,3'-biindolinylidene]-1-carboxylate (***iso*-I-27**)

Di-*tert*-butyl dicarbonate (4 mg, 0.02 mmol, 5.00 equiv.) and ***iso*-I-30** (2 mg, 4.00 µmol, 1.00 equiv.) were dissolved in 1 mL THF. One crumble of DMAP was added and the mixture was stirred at room temperature for 4 h. The crude mixture was subjected to drying *in vacuo* at 75 °C. The solid was diluted in EtOAc and washed with 1 M NH_4_Cl solution. The aqueous layer was extracted once with methylene chloride. The combined organic layers were dried over Na_2_SO_4_, the solvent was evaporated and the residue was dried *in vacuo* to obtain the title compound.

**^1^H NMR** (400 MHz, Chloroform-*d*) δ = 9.20 (dd, *J* = 8.2, 1.3 Hz, 1H), 9.00 (d, *J* = 7.9 Hz, 1H), 7.81 (d, *J* = 8.1 Hz, 1H), 7.41 (td, *J* = 8.4, 8.0, 1.3 Hz, 1H), 7.36 – 7.31 (m, 2H), 7.28 (td, *J* = 7.7, 1.2 Hz, 1H), 7.12 (t, *J* = 7.8 Hz, 1H), 7.09 – 7.04 (m, 3H), 6.67 (d, *J* = 7.8 Hz, 1H), 3.87 (s, 3H), 1.69 (s, 9H).

**HR-MS** (ESI), [M-H_2_+Na]^+^, for C_28_H_24_N_2_O_5_Na^+^, m/z calcd. 491.15774, found 491.15671.

**R_f_** (SiO_2_; EtOAc: Petrolum ether; 1:3): 0.56.

A yield could not be determined unequivocally due to the low reaction scale. A ^13^C-NMR was not obtained due to the small amount of material.

*1-Propylindoline-2,3-dione (****10****)*

Isatin (2.80 g, 20.0 mmol, 1.00 equiv.), Cs_2_CO_3_ (966 mg, 2.96 mmol, 0.16 equiv.) and 1-bromopropyl (6.9 mL, 76.1 mmol, 4.00 equiv.) were dissolved in DMF (4.0 mL) in a crimp top vial. The vial was sealed and the mixture was stirred at room temperature for 18 h. The resulting mixture was diluted with EtOAc, washed three times with water and the aqueous layer was extracted once with EtOAc. The combined organic layers were dried over Na2SO4 and concentrated *in vacuo* yielding an orange solid. Purification by flash column chromatography (SiO_2_, EtOAc:Petroleum ether 40-60, 1:3 to 1:1) yielded the title compound as an orange solid [(825 mg, 4.36 mmol, 23%)](https://mbook.housing.rug.nl/ELN/127235).

**^1^H NMR** (400 MHz, Chloroform-*d*) δ = 7.64 – 7.53 (m, 2H), 7.11 (td, *J* = 7.6, 0.8 Hz, 1H), 6.90 (dd, *J* = 7.9, 1.0 Hz, 1H), 3.74 – 3.65 (m, 2H), 1.75 (h, *J* = 7.4 Hz, 2H), 1.00 (t, *J* = 7.4 Hz, 3H).

**R_f_** (SiO_2_; EtOAc:Petroleum ether 40-60; 1:2): 0.50.

The analytical data are in agreement with published data [5].

# 1. NMR and HRMS spectra

(*E*)-Isoindigo (***iso*-I-45**)


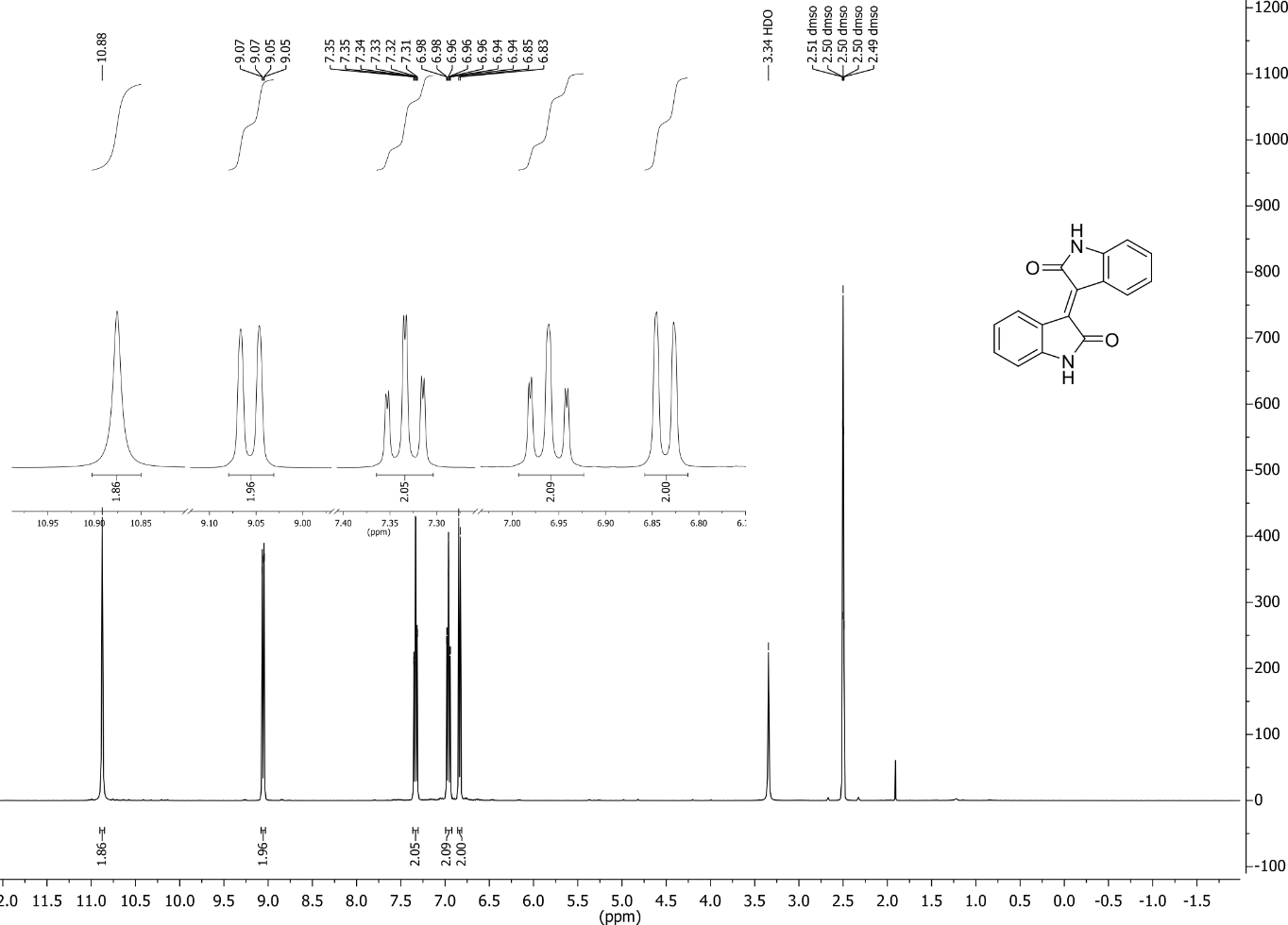


Figure 1: ^1^H-NMR(400 MHz) in DMSO-d6.

Figure 2: HRMS (ESI).

Di-tert-butyl 2,2'-(2,2'-dioxo-[3,3'-biindolinylidene]-1,1'-diyl)(*E*)-diacetate (***iso*-I-31**)


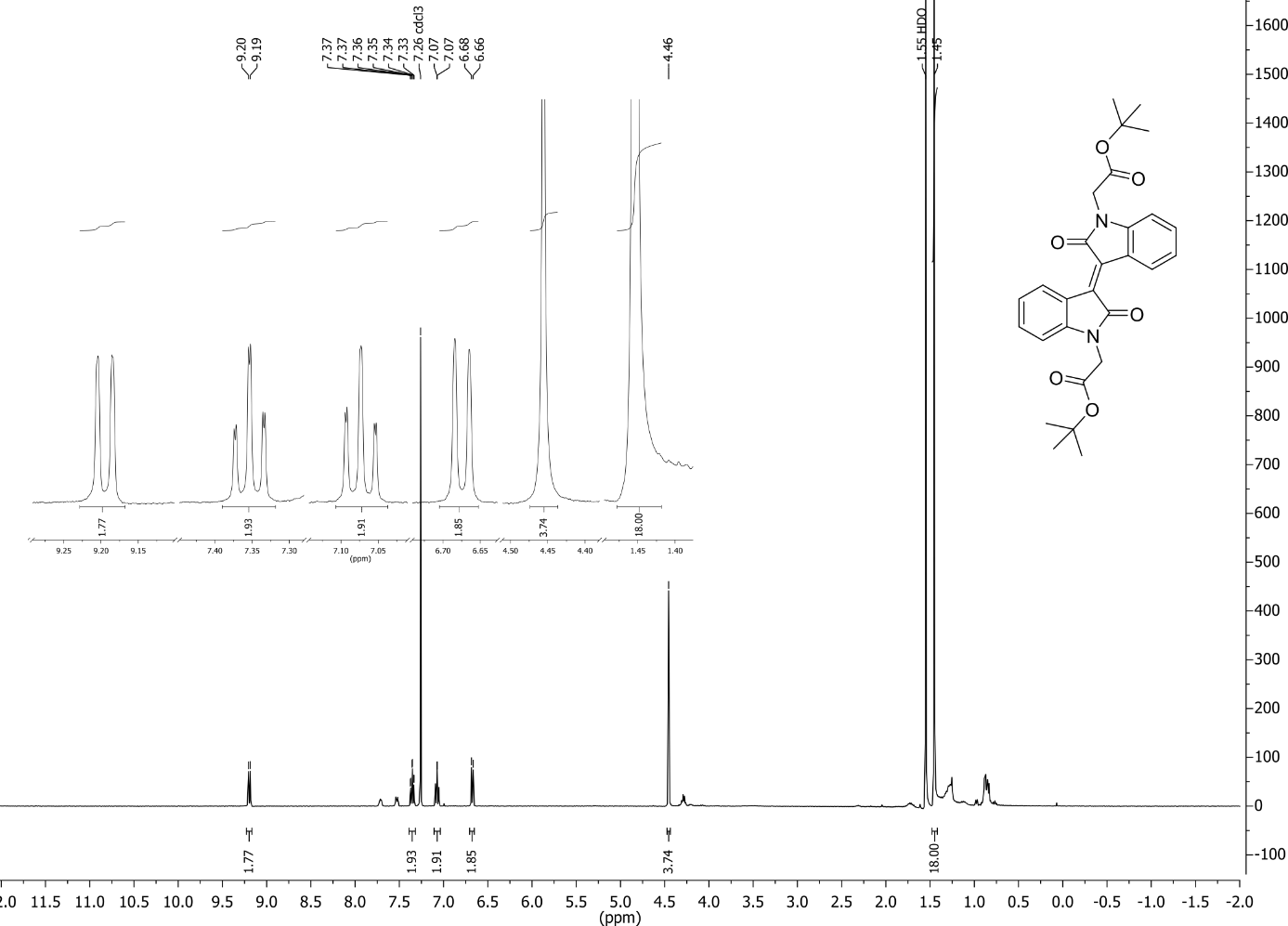


Figure 3: ^1^H-NMR(400 MHz) in Chloroform-d.


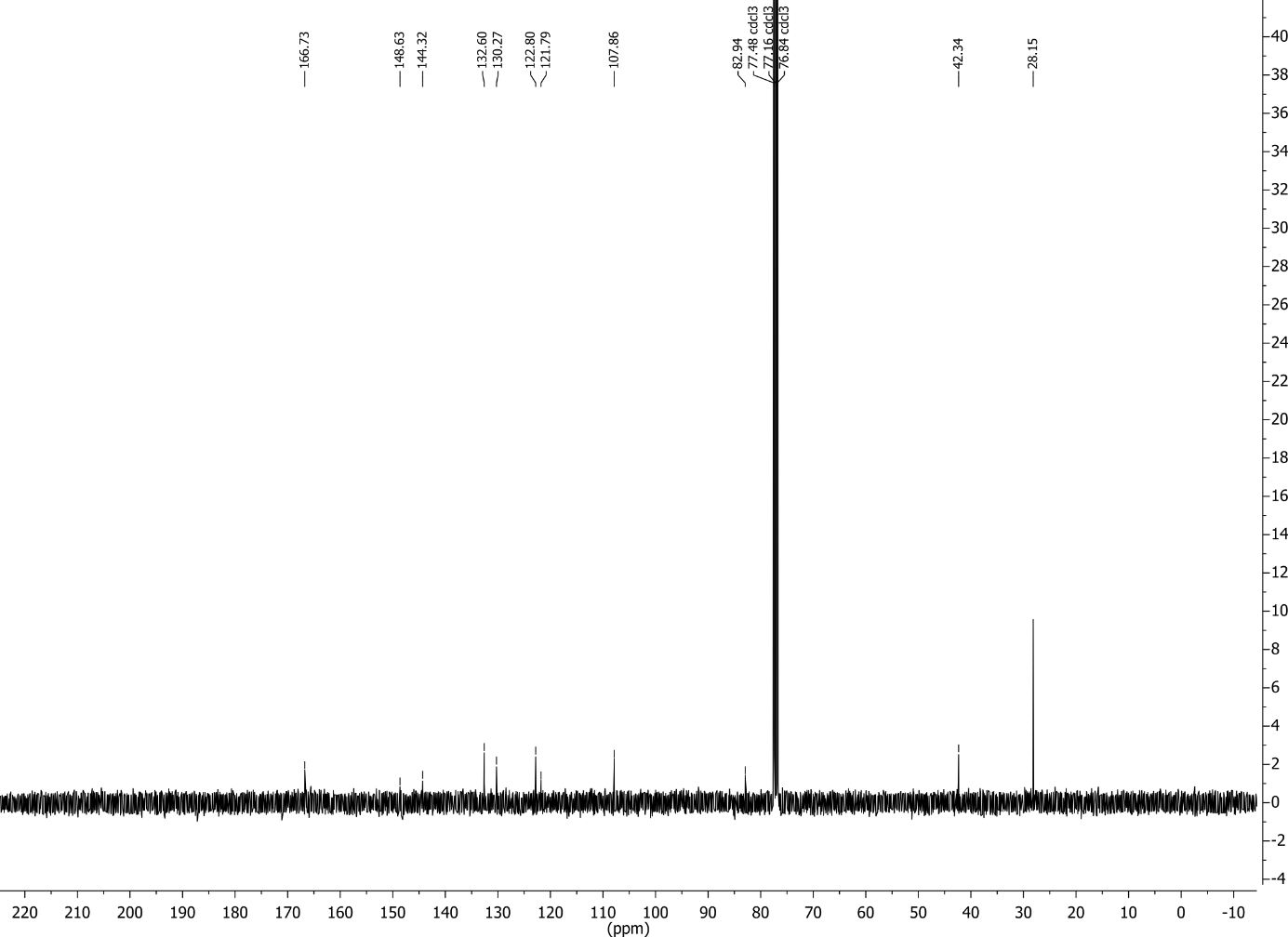


Figure 4: ^13^C-NMR(101 MHz) in Chloroform-d.

Figure 5: HRMS (ESI).

di-tert-butyl (E)-2,2'-dioxo-[3,3'-biindolinylidene]-1,1'-dicarboxylate (***iso*-I-36**)


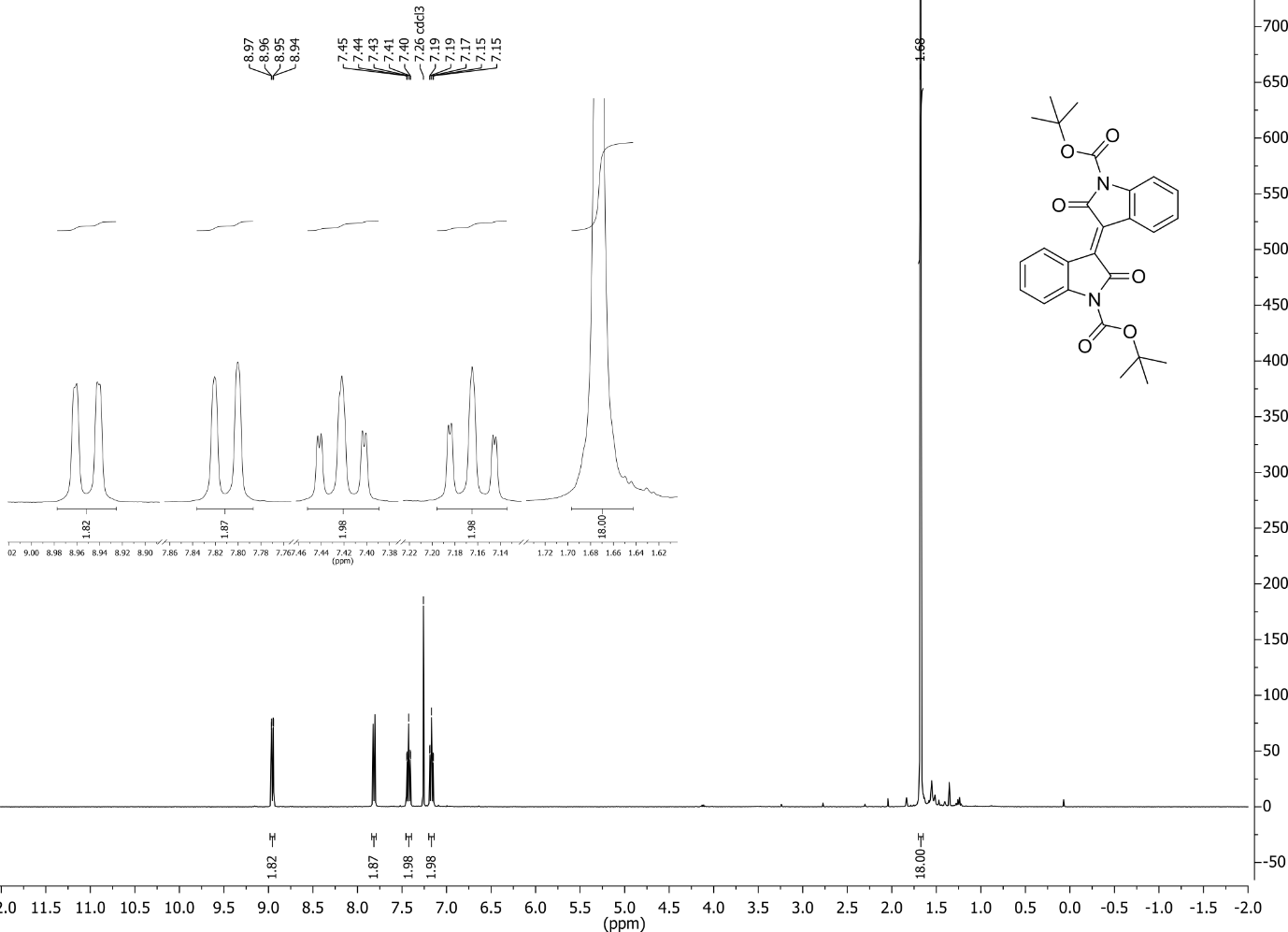


Figure 6: ^1^H-NMR(400 MHz) in Chloroform-d.

1-(4-methoxyphenyl)indolin-2-one (**3**)


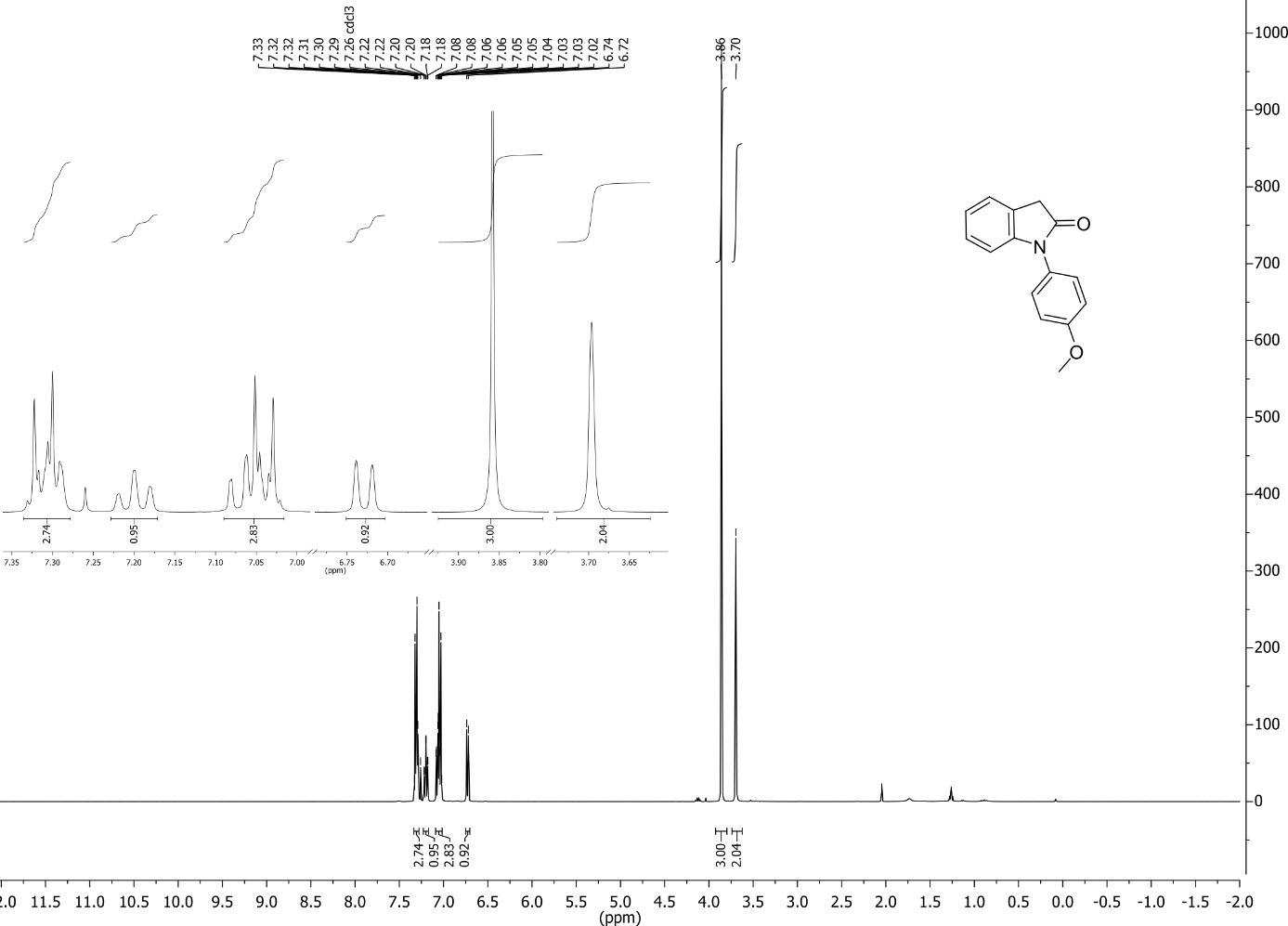


Figure 7: ^1^H-NMR(400 MHz) in Chloroform-d.


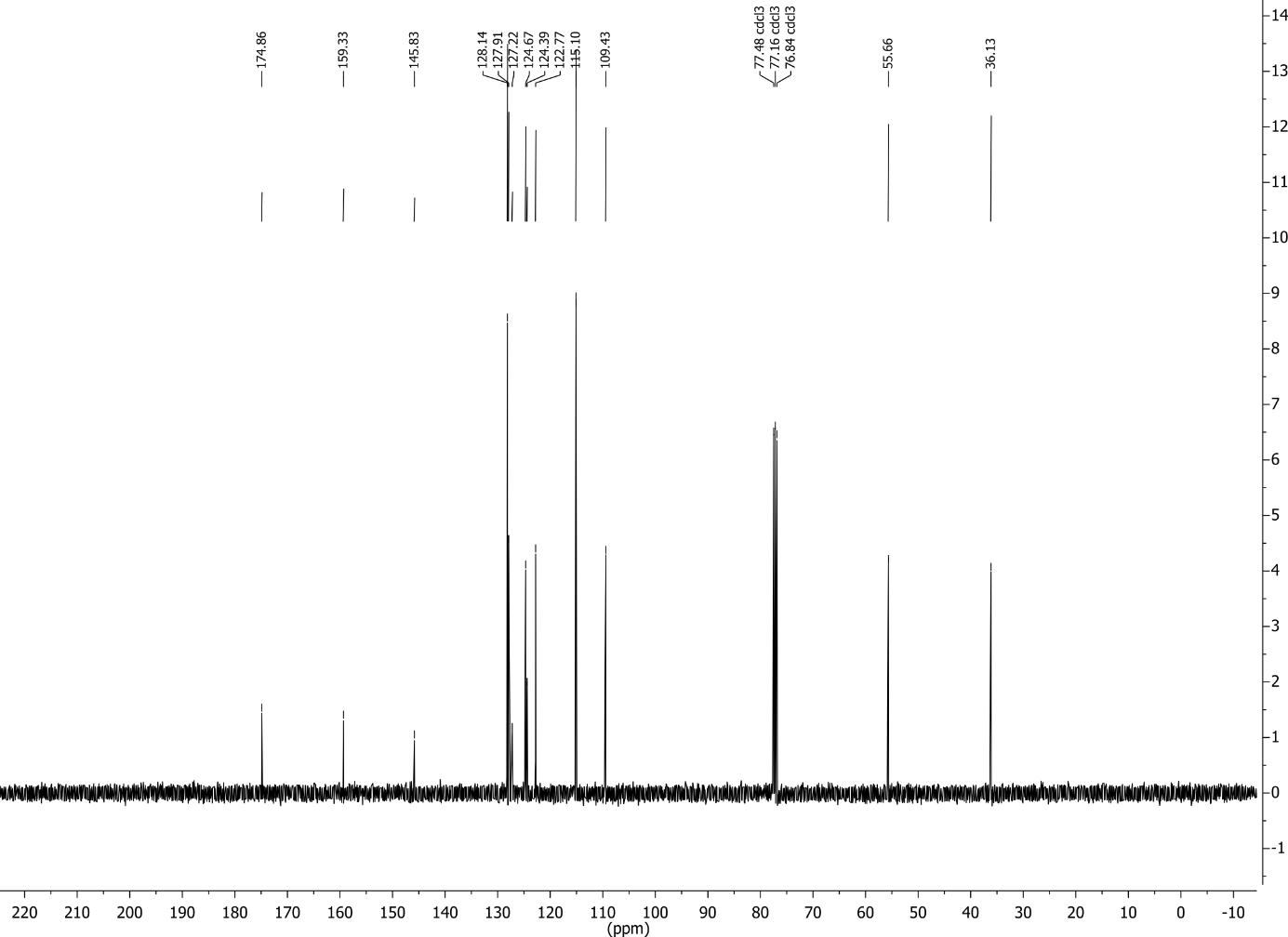


Figure 8: ^13^C-NMR(101 MHz) in Chloroform-d.

(E)-1-(4-methoxyphenyl)-[3,3'-biindolinylidene]-2,2'-dione (***iso*-I-30**)


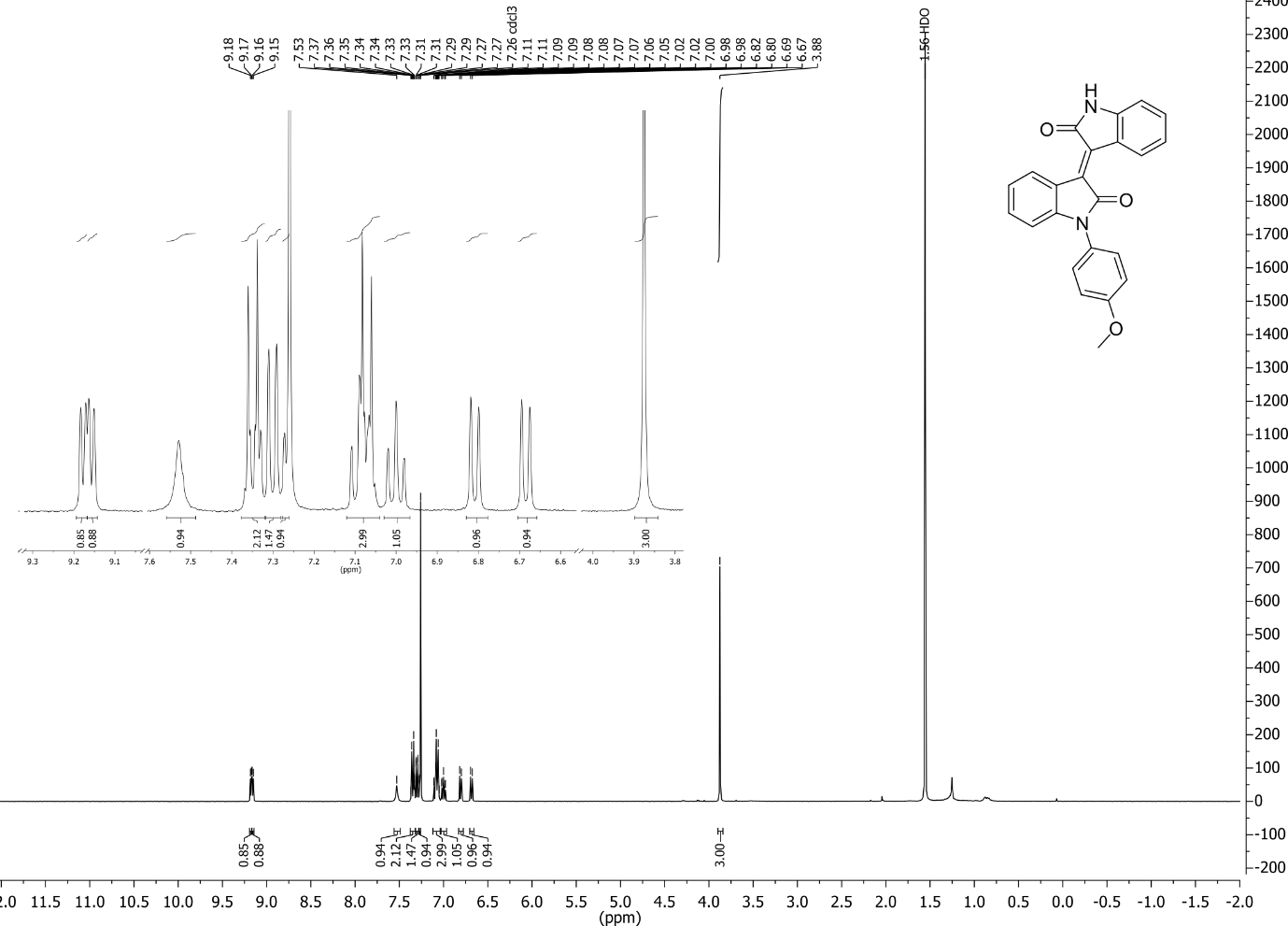


Figure 9: ^1^H-NMR(400 MHz) in Chloroform-d.


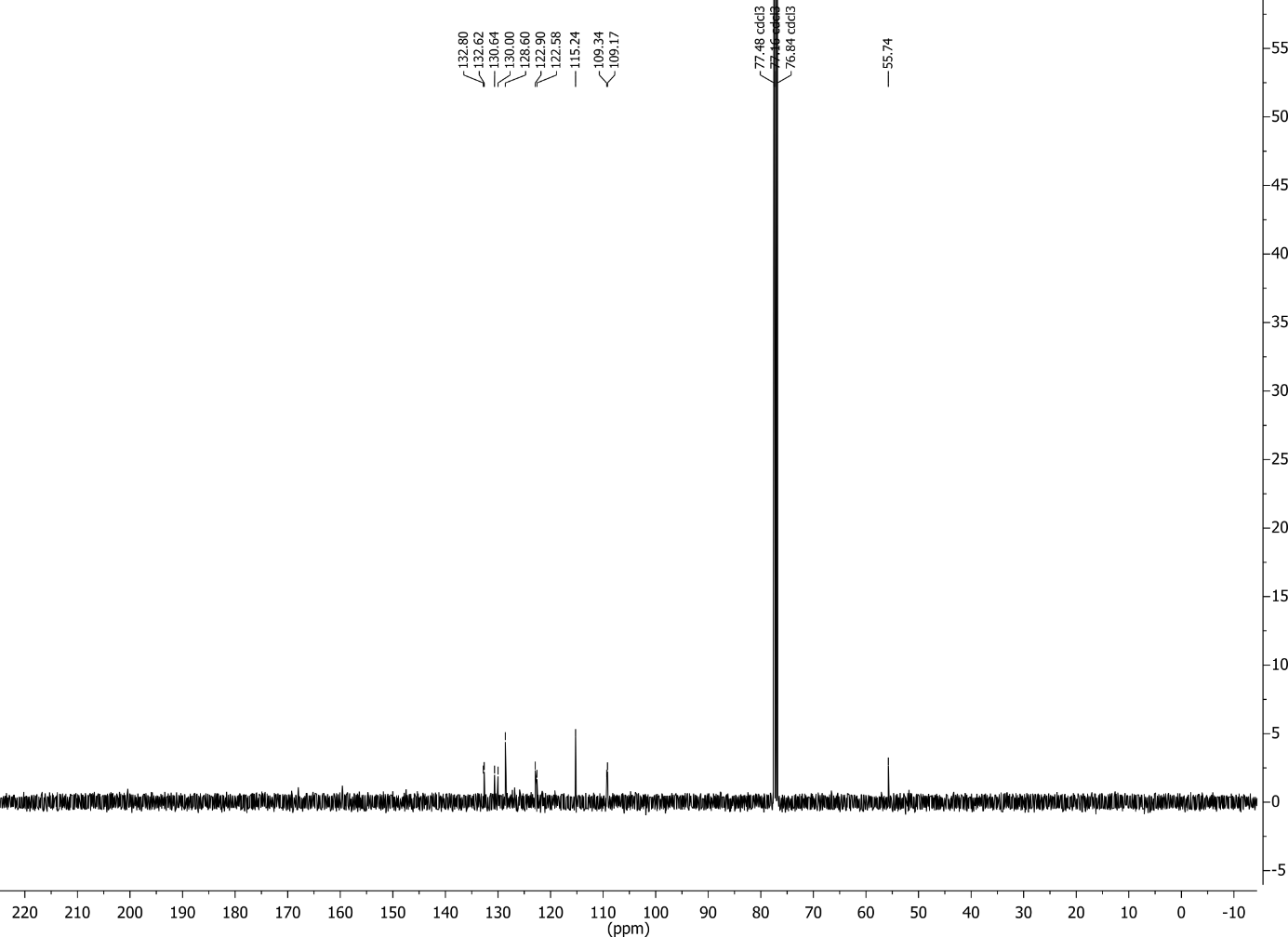


Figure 10: ^13^C-NMR(101 MHz) in Chloroform-d.


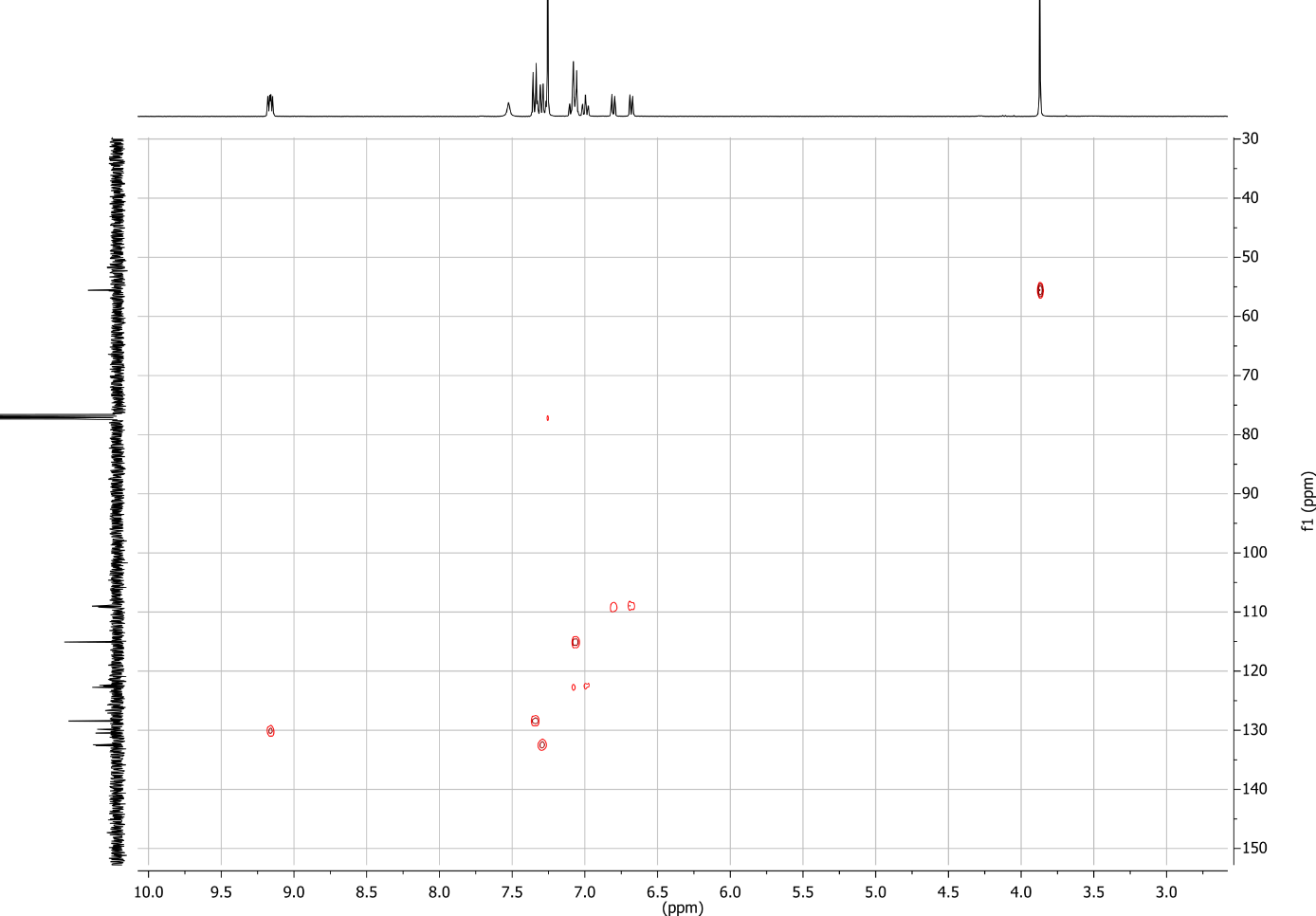


Figure 11: HSQC in Chloroform-d.

Figure 12: HRMS (ESI).

(E)-1,1'-bis(4-methoxyphenyl)-[3,3'-biindolinylidene]-2,2'-dione (***iso*-I-25**)


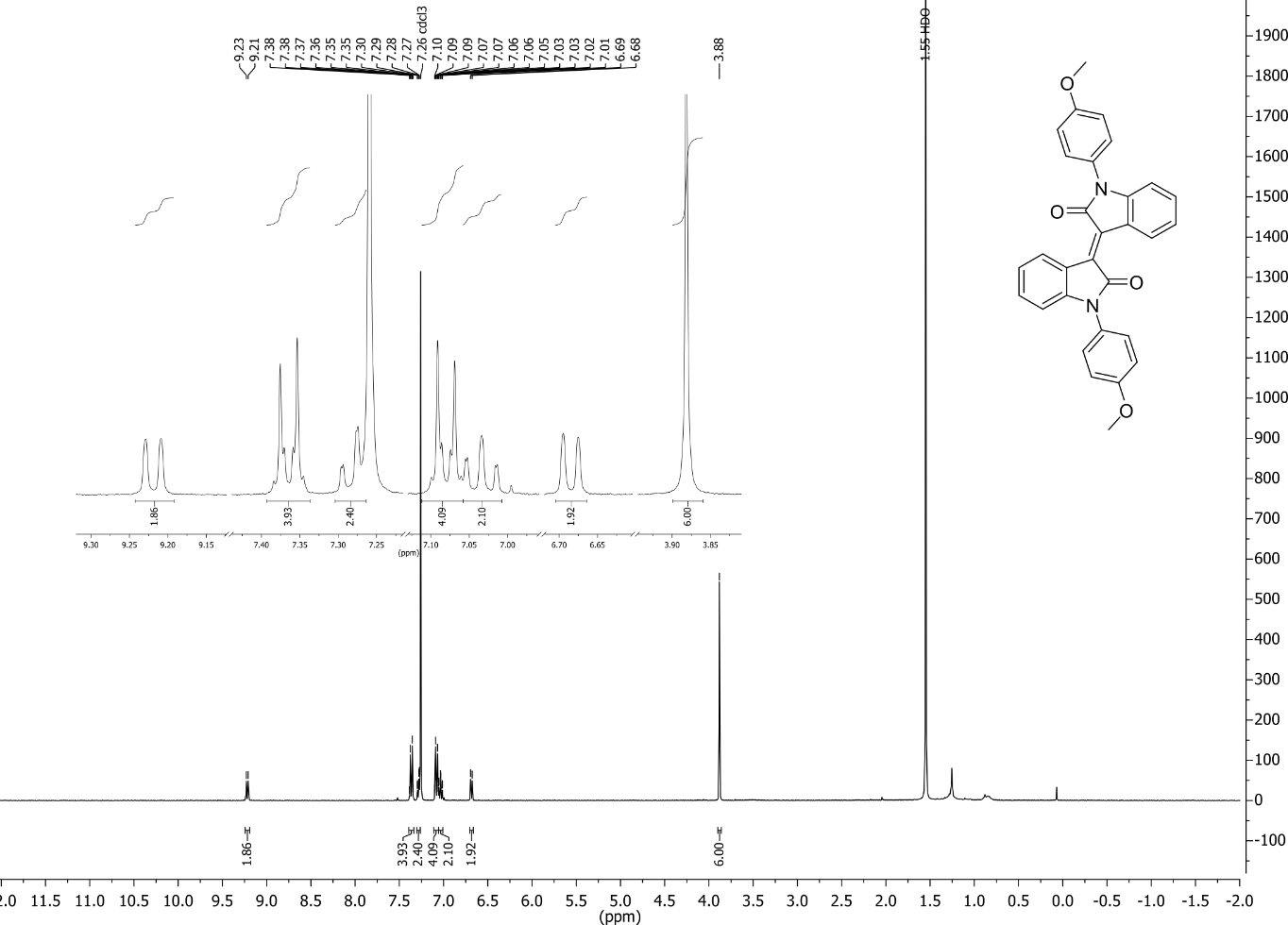


Figure 13: ^1^H-NMR(400 MHz) in Chloroform-d.


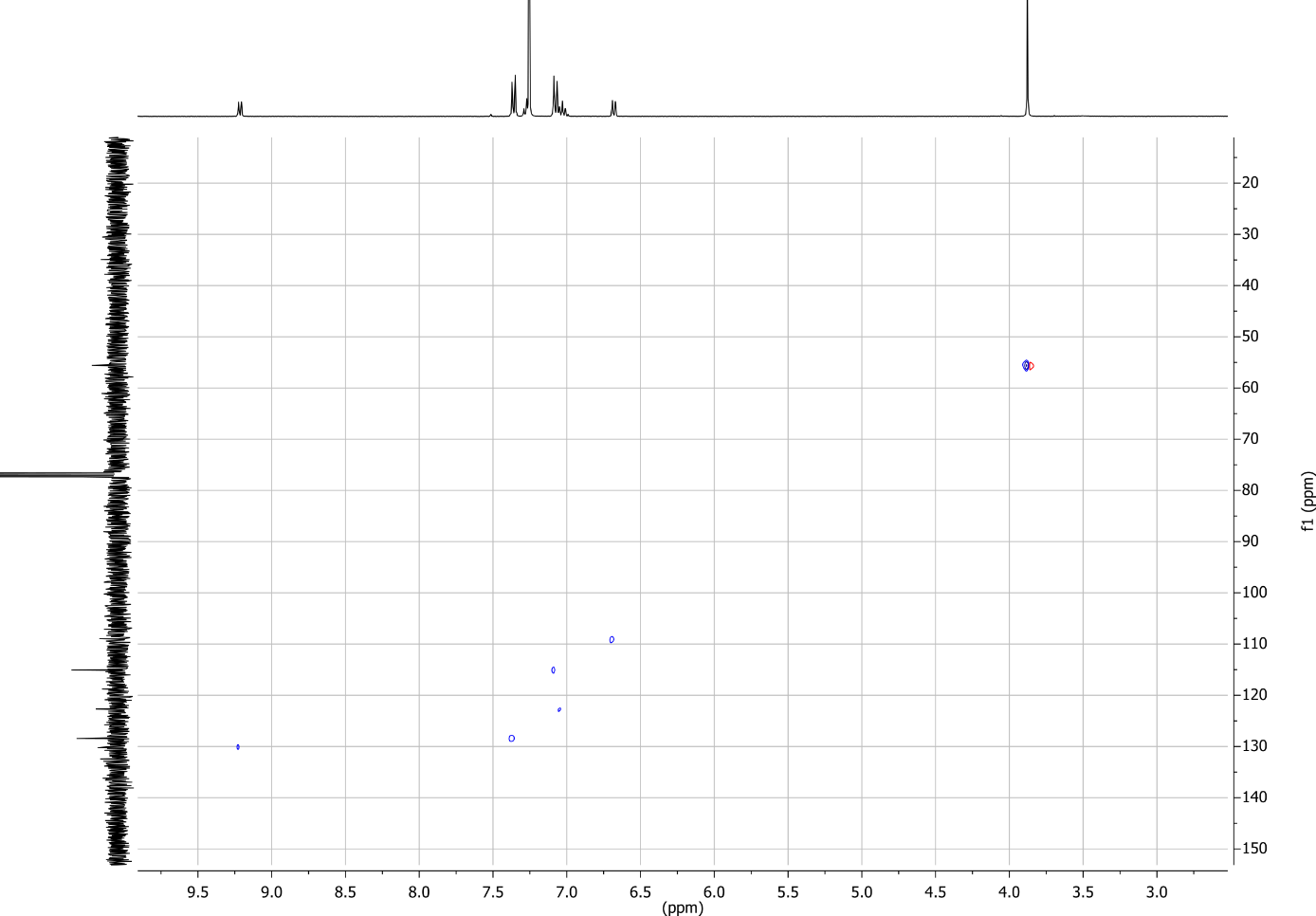


Figure 14: HSQC in Chloroform-d.

Figure 15: HRMS (ESI).

tert-butyl 2-(2,3-dioxoindolin-1-yl)acetate (**1**)


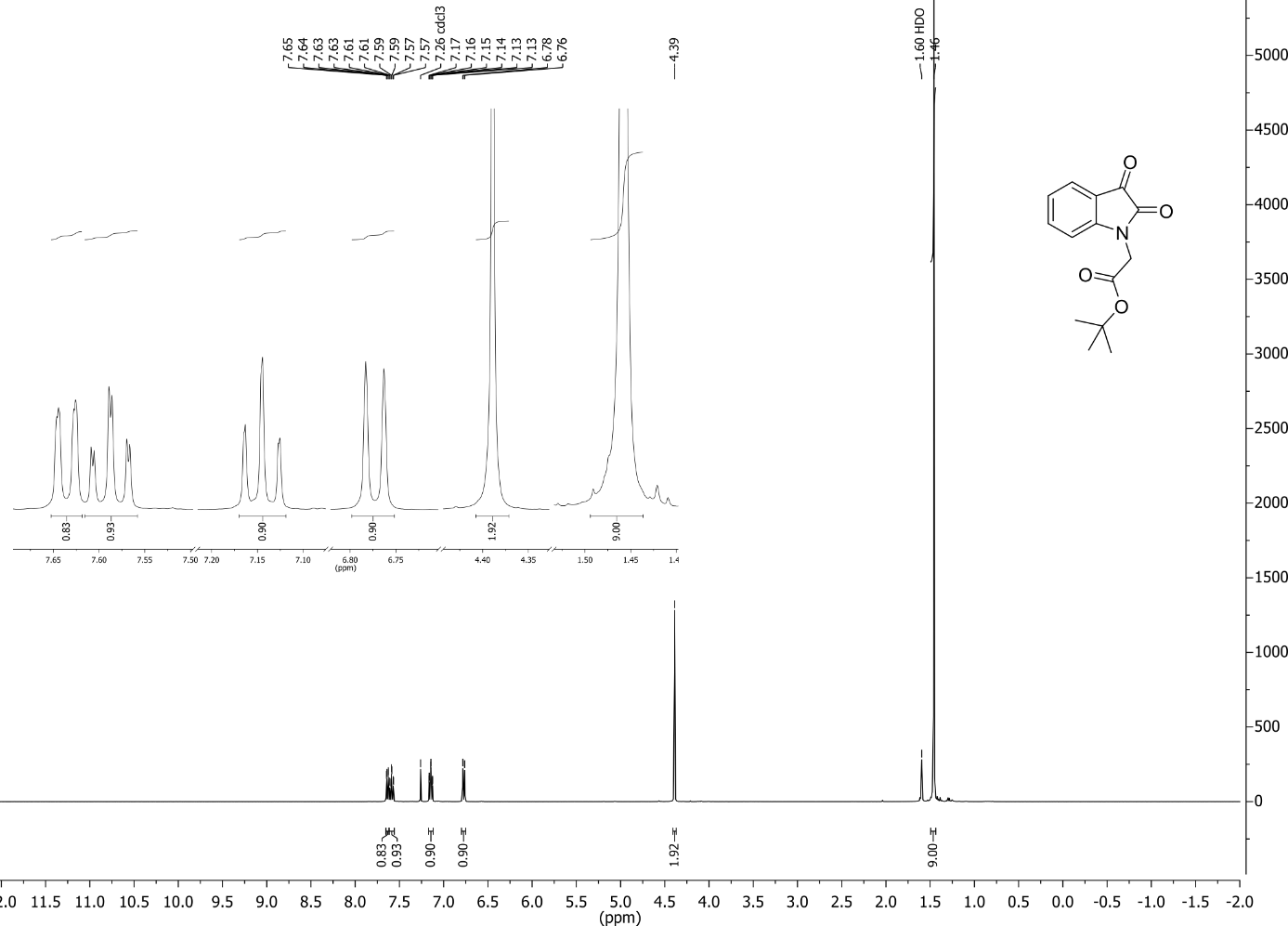


Figure 16: ^1^H-NMR(400 MHz) in Chloroform-d.


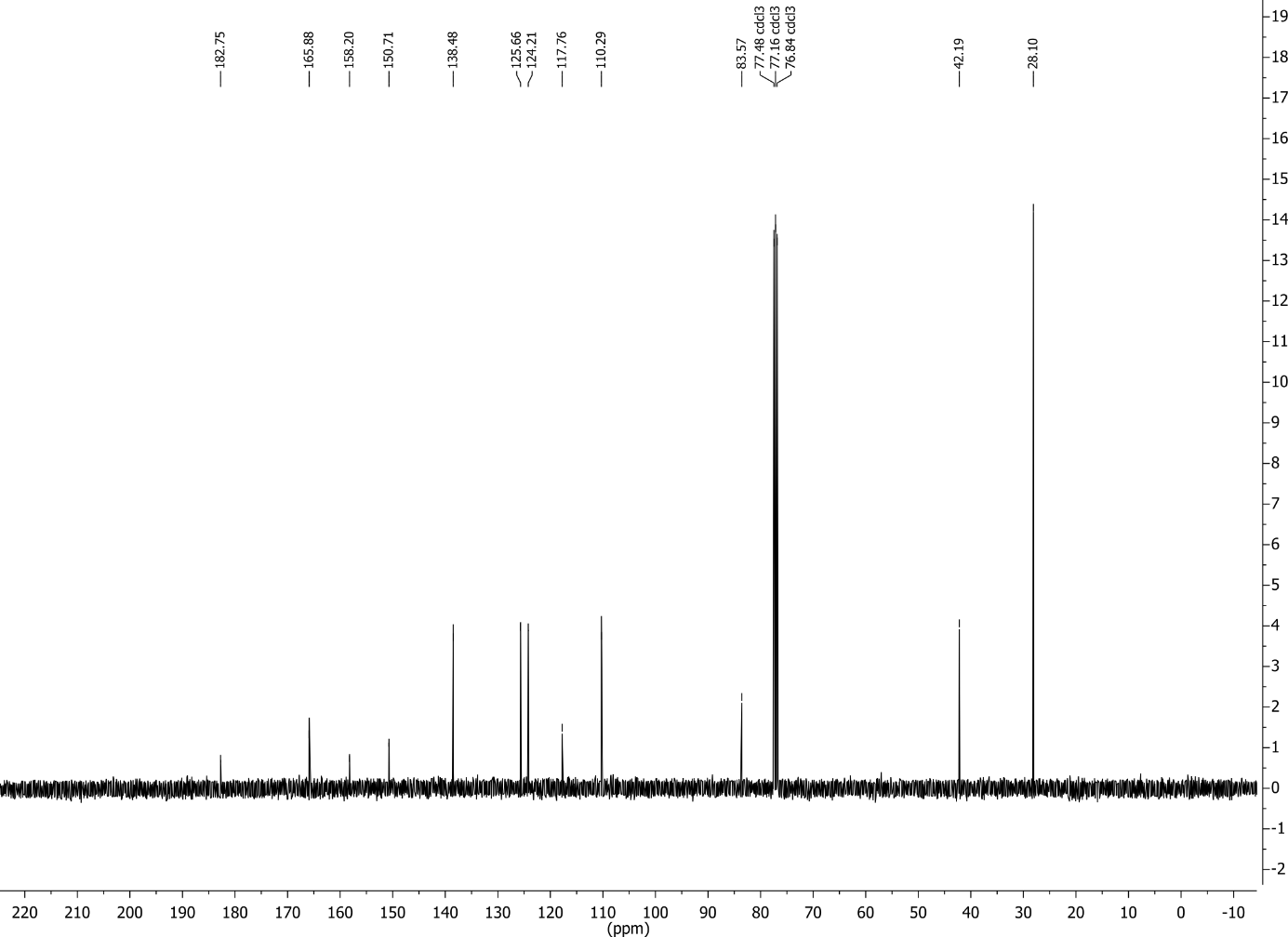


Figure 17: ^13^C-NMR(400 MHz) in Chloroform-d.

Figure 18: HRMS (ESI)

*tert*-butyl (*E*)-2-(2,2'-dioxo-[3,3'-biindolinylidene]-1-yl)acetate (***iso*-I-35**)


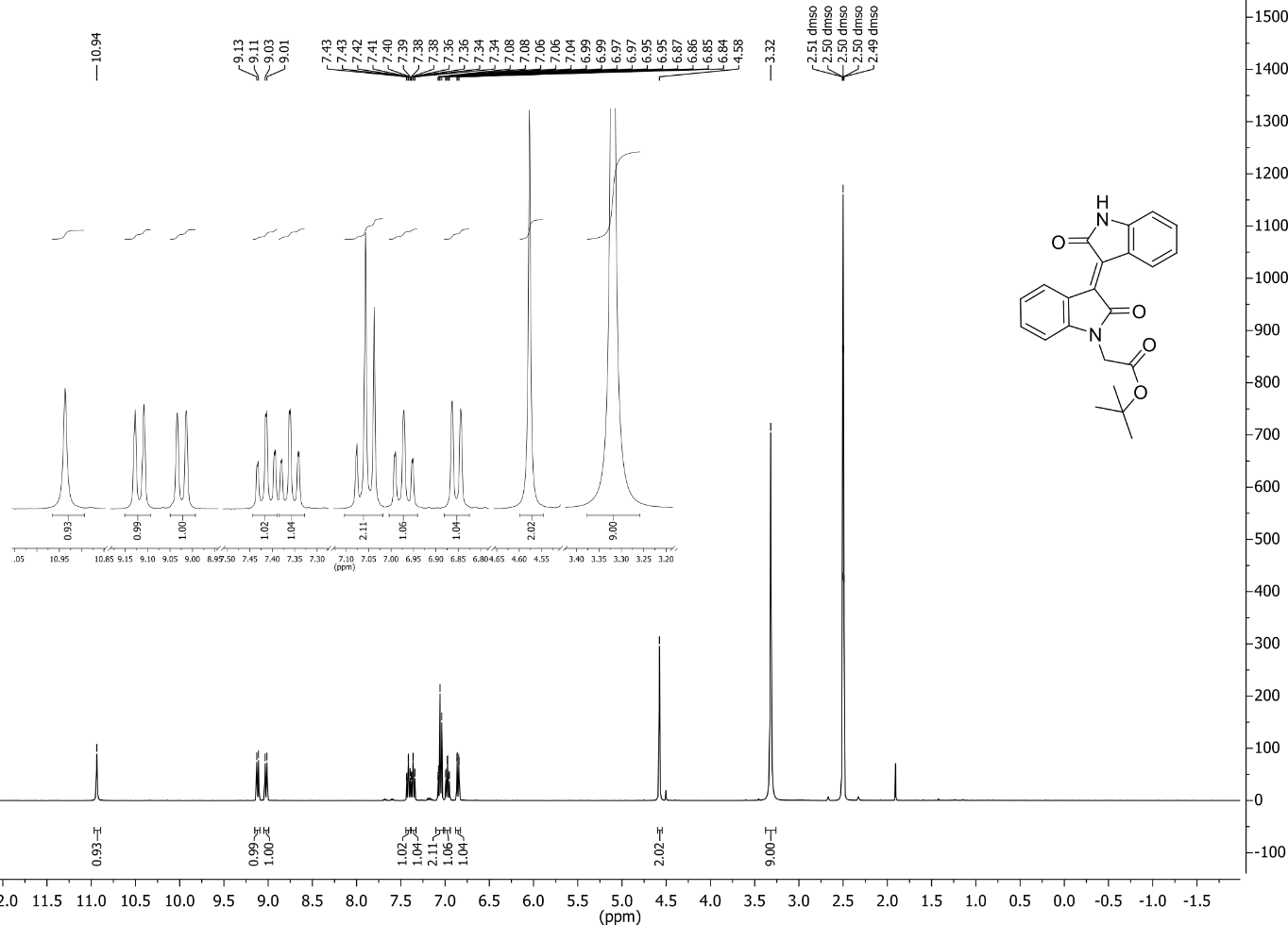


Figure 19: ^1^H-NMR(400 MHz) in DMSO-d6.


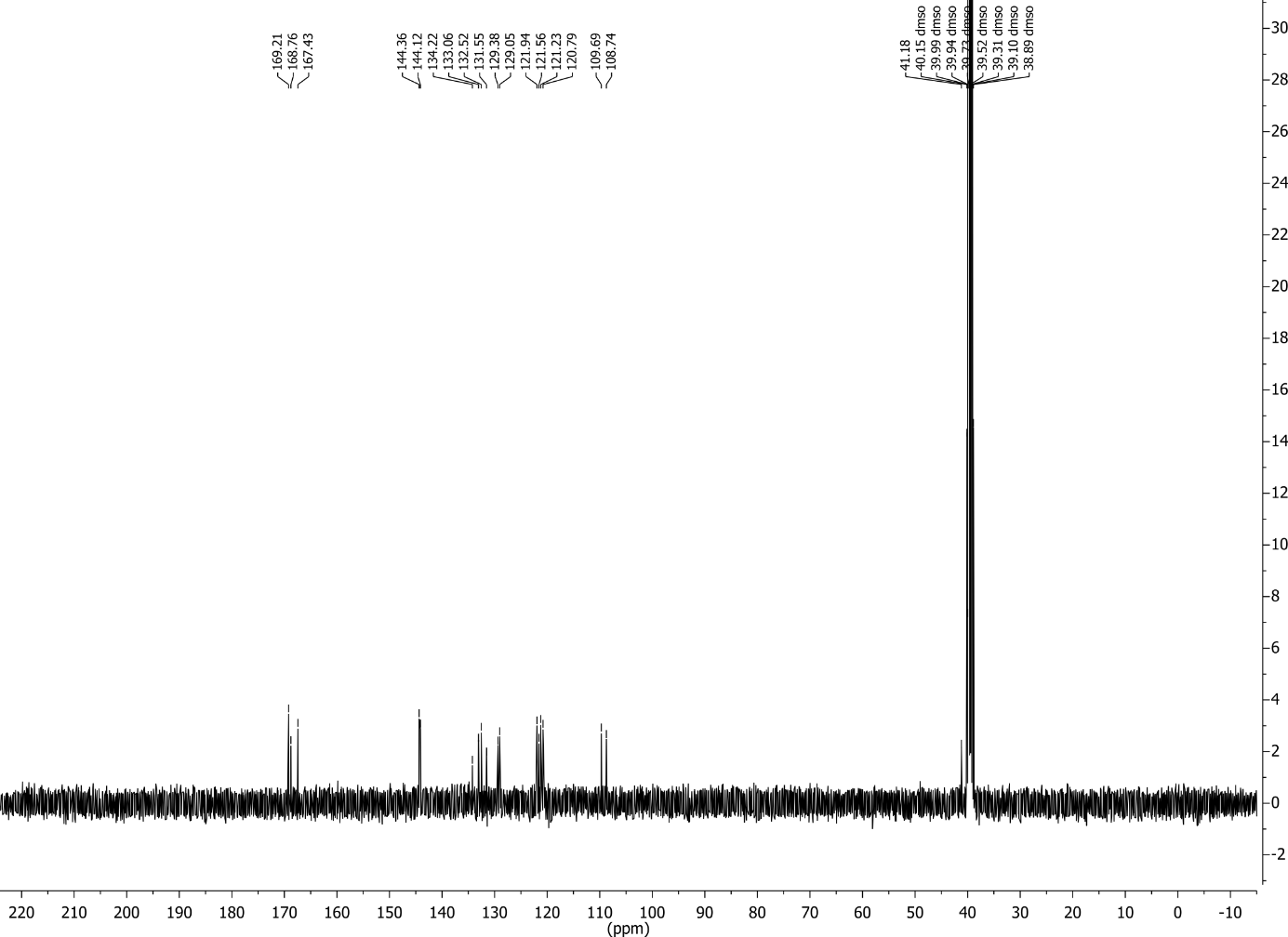


Figure 20: ^13^C-NMR(101 MHz) in DMSO-d6.

*tert*-butyl (*E*)-1'-(4-methoxyphenyl)-2,2'-dioxo-[3,3'-biindolinylidene]-1-carboxylate (***iso*-I-27**)


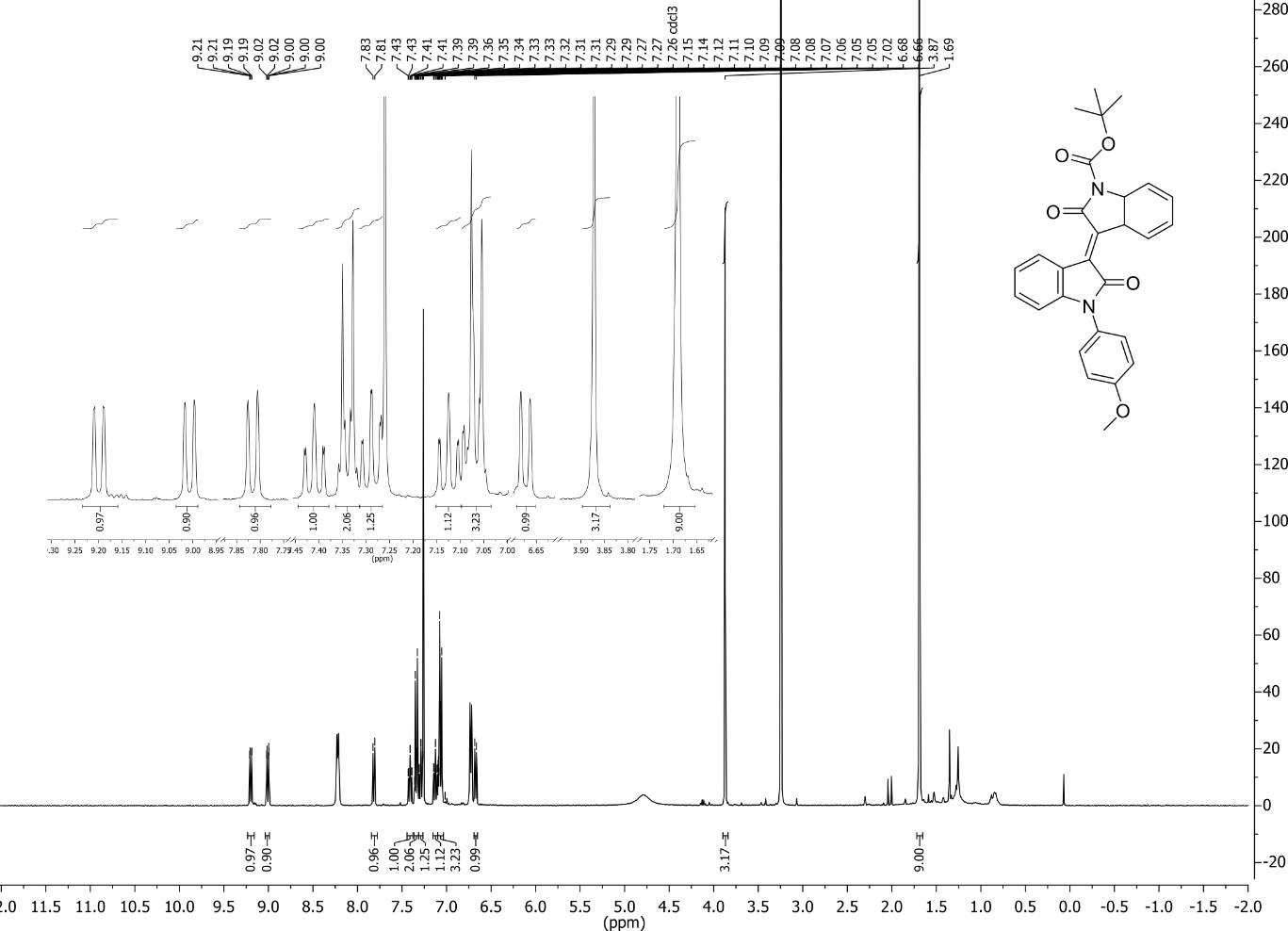


Figure 21: ^1^H-NMR(400 MHz) in Chloroform-d.

Figure 22: HRMS (ESI).

1-propylindoline-2,3-dione (**10**)


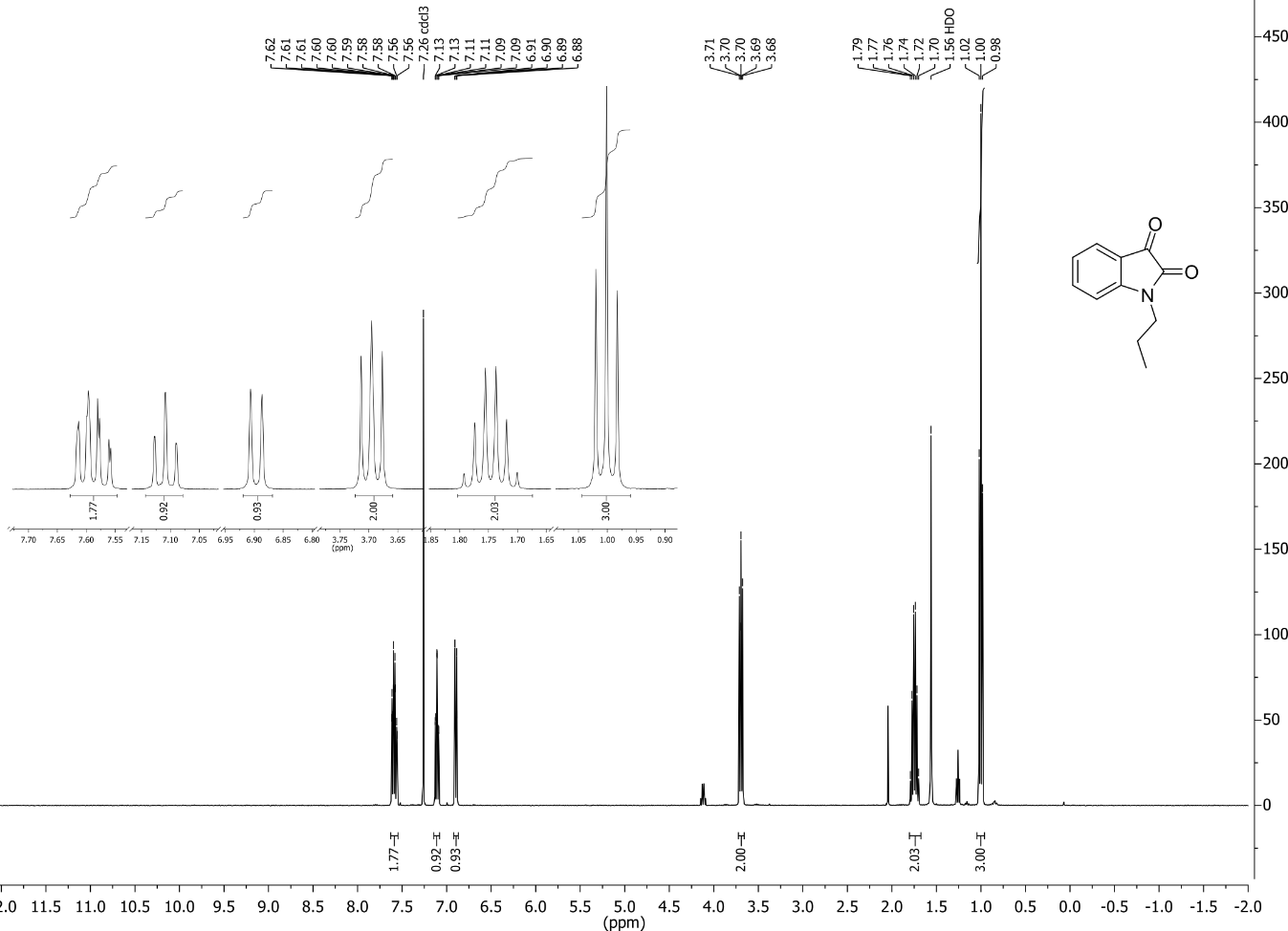


Figure 23: ^1^H-NMR(400 MHz) in Chloroform-d.

(*E*)-1-(4-(trifluoromethyl)phenyl)-[3,3'-biindolinylidene]-2,2'-dione (***iso*-I-42**)

Figure 24: HRMS (ESI).

# 2.TD-DFT Library

We prepared our library by combining the substituents in the Markush-editor of ChemAxon[6] and exporting them as a library of all non-identical permutations of substitution. A script from Ishikawa transformed the library into 3D-geometries [7]. It utilizes the OpenBabel [8], Balloon [9] and MOPAC2012 [10] softwares. OpenBabel adds explicit hydrogen atoms, Balloon transforms the 2D drawing into a 3D model and the MOPAC2012 package performs a preliminary molecular mechanics geometry optimization on a set of 100 conformers and a single-point calculation at the PM7 level. After sorting the energies, we proceeded with the molecular geometry characterized by the lowest energy at the PM7 level.

Subsequently, we optimized the obtained geometries in the gas phase and different solvents, namely DMSO, acetonitrile, chloroform, toluene and methanol, at the ωB97X-D [11]/def2-TZVP [12] level of theory using the corresponding solvation models based on electron density (SMD models) [13]. Vibrational frequency analysis confirmed that the geometries where minima. Imaginary vibrational frequencies that corresponded to the rotation of *t*-butyl groups were ignored if the optimization criteria of the Gaussian16 software were met.

After completion of the optimizations, we simulated the UV-Vis spectra of all molecules using TD-DFT calculations at the PBE0/cc-pVDZ level of theory with the respective SMD-model over 25 states.

## 2.1 Benchmarks

We chose the chromism in different solvents (solvatochromism), namely DMSO, acetonitrile, chloroform, toluene and methanol, as a reference. Calculations at the time-dependent DFT (TD-DFT) level of theory at a geometry optimized at the ωB97X-D/def2-TZVP level of theory with the respective solvation model based on electron density (SMD model) obtained the respective spectra. Comparison of experimental data and calculated results of the solvatochromism relative to DMSO and the absolute deviation from the lowest energy absorption maximum resulted in a comparable set of data. The results are summarized in Table 1 and identify the PBE0 functional [14] as best suited for our use. The low calculation time combined with the low error of 11.90 nm is unmet by the other functionals. Considering not only relative error but, the absolute deviation from the calculated spectra, the PBE0 functional shows to be highly accurate with an average 15.18 nm error. Similarly, benchmarking of the basis set obtained the PBE0/cc-pVDZ [15] level of theory as best suited for the TD-DFT calculation of the library.

The calculations were performed with the Gaussian16 software [16] on the Peregrine HPC cluster on compute nodes with two Intel Xeon E5 2680v3 or two Intel Xeon E5 2680v4 CPUs in a shared memory architecture connected by a 56 Gbps infiniband network. Eight cores and 8 Gb memory were used each job. The calculation wall-time is referenced here as T_calc_.

Table 1: TD-DFT Benchmarks of Isoindigo

|  | HF | B3LYP | cam-B3LYP | ωB97X-D | M06-2X | PBE0 |
| --- | --- | --- | --- | --- | --- | --- |
|  |  |  |  |  |  | def2-TZVP |
| Error_rel_ | 12.26 | 12.51 | 12.18 | 12.06 | 12.05 | 11.90 |
| Error_abs_ | 107.87 | 31.26 | 39.38 | 42.87 | 37.11 | 15.18 |
| T_calc_ [min] | 69.90 | 71.88 | 86.79 | 87.29 | 98.08 | 72.76 |
| ΔError_min/max_ | 5.48 | 9.42 | 8.09 | 8.22 | 8.50 | 8.20 |
| Min-Error_rel_ | 10.55 | 8.69 | 8.98 | 8.76 | 8.57 | 8.43 |
| Max-Error_rel_ | 15.85 | 18.11 | 17.07 | 16.98 | 17.07 | 16.63 |
|  | PBE0 | | | | | |
|  | def2-TZVPP | aug-cc-pVDZ | cc-pVDZ | cc-pVTZ | 6-311+g(2d.p) | 6-311++g(2d.p) |
| Error_rel_ | -12.10 | -12.27 | -11.59 | -12.13 | -12.33 | -12.33 |
| Error_abs_ | 15.53 | 18.18 | 6.37 | 13.33 | 18.87 | 18.89 |
| ΔError_min/max_ | 9.61 | 9.23 | 8.81 | 9.09 | 9.25 | 9.25 |
| Min-Error_rel_ | -8.02 | -8.45 | -7.7 | -8.34 | -8.53 | -8.53 |
| Max-Error_rel_ | -17.63 | -17.68 | -16.51 | -17.43 | -17.78 | -17.78 |

## 2.2 Geometries

| **iso-I** 1 |  | gasphase |  |  | toluene |  |  | DMSO |  |  | methanol |  |  | acetonitrile |  |  | chloroform |  |
| --- | --- | --- | --- | --- | --- | --- | --- | --- | --- | --- | --- | --- | --- | --- | --- | --- | --- | --- |
|  | x | y | z | x | y | z | x | y | z | x | y | z | x | y | z | x | y | z |
| C | -0.501228 | 2.919669 | -0.507697 | -0.504285 | 2.894199 | -0.576208 | -0.509094 | 2.863641 | -0.668586 | -0.510544 | 2.864877 | -0.760564 | -0.505443 | 2.869747 | -0.662741 | -0.504182 | 2.889442 | -0.618303 |
| C | -1.279371 | 4.068976 | -0.545228 | -1.284788 | 4.040388 | -0.646838 | -1.292482 | 4.005679 | -0.775226 | -1.291622 | 4.009305 | -0.852504 | -1.288051 | 4.012515 | -0.769500 | -1.283946 | 4.036219 | -0.692097 |
| C | -2.649881 | 4.007105 | -0.351154 | -2.657468 | 3.979311 | -0.460860 | -2.666528 | 3.946552 | -0.585923 | -2.659069 | 3.957536 | -0.617725 | -2.662638 | 3.953854 | -0.583417 | -2.655731 | 3.977773 | -0.493433 |
| C | -3.281778 | 2.788088 | -0.142144 | -3.288236 | 2.764204 | -0.226288 | -3.295191 | 2.737936 | -0.312658 | -3.286576 | 2.753963 | -0.318370 | -3.292619 | 2.745077 | -0.312783 | -3.286555 | 2.765102 | -0.244609 |
| C | -2.498072 | 1.653928 | -0.124461 | -2.500024 | 1.634151 | -0.171898 | -2.502330 | 1.613334 | -0.219787 | -2.496706 | 1.627091 | -0.242210 | -2.500671 | 1.619898 | -0.219610 | -2.498832 | 1.634733 | -0.187430 |
| C | -1.102003 | 1.686622 | -0.275968 | -1.103586 | 1.666506 | -0.314844 | -1.106707 | 1.645710 | -0.362124 | -1.106144 | 1.655434 | -0.420352 | -1.104589 | 1.651601 | -0.359441 | -1.103590 | 1.665628 | -0.339348 |
| N | -2.932096 | 0.338316 | 0.035694 | -2.932087 | 0.321116 | 0.005436 | -2.929869 | 0.304368 | -0.011595 | -2.918838 | 0.314620 | -0.017743 | -2.929133 | 0.310586 | -0.012068 | -2.929555 | 0.321765 | -0.001587 |
| C | -1.870251 | -0.538854 | -0.025287 | -1.874298 | -0.556437 | -0.048908 | -1.874879 | -0.571931 | -0.052825 | -1.866364 | -0.555573 | -0.081498 | -1.874015 | -0.564867 | -0.048363 | -1.873781 | -0.554643 | -0.062012 |
| C | -0.620656 | 0.298670 | -0.228481 | -0.622772 | 0.279811 | -0.247036 | -0.624993 | 0.262790 | -0.264699 | -0.625241 | 0.274887 | -0.326086 | -0.623907 | 0.268111 | -0.262394 | -0.623732 | 0.279697 | -0.267599 |
| C | 0.623977 | -0.239623 | -0.285360 | 0.624930 | -0.251877 | -0.282678 | 0.625467 | -0.259002 | -0.283262 | 0.624016 | -0.246576 | -0.355089 | 0.625224 | -0.256888 | -0.283796 | 0.623932 | -0.250539 | -0.299579 |
| O | -1.978651 | -1.731130 | 0.124862 | -1.982862 | -1.749553 | 0.106467 | -1.978875 | -1.764044 | 0.129310 | -1.960297 | -1.750913 | 0.116778 | -1.977573 | -1.756497 | 0.139897 | -1.979524 | -1.749185 | 0.097250 |
| C | 1.089725 | -1.623410 | -0.458066 | 1.100360 | -1.630467 | -0.462351 | 1.109533 | -1.632284 | -0.465162 | 1.103532 | -1.614938 | -0.564034 | 1.105201 | -1.631668 | -0.467131 | 1.100046 | -1.627417 | -0.484185 |
| C | 2.489947 | -1.611946 | -0.358302 | 2.499426 | -1.610697 | -0.348086 | 2.506824 | -1.604150 | -0.337947 | 2.496752 | -1.598438 | -0.407771 | 2.502468 | -1.608122 | -0.338222 | 2.497655 | -1.608601 | -0.355876 |
| N | 2.941810 | -0.315932 | -0.109307 | 2.939022 | -0.314878 | -0.080148 | 2.934508 | -0.307519 | -0.058954 | 2.922579 | -0.307421 | -0.084690 | 2.933612 | -0.312485 | -0.057473 | 2.933984 | -0.313887 | -0.076210 |
| C | 1.889345 | 0.575806 | -0.096372 | 1.882109 | 0.566842 | -0.056810 | 1.873942 | 0.564445 | -0.029108 | 1.867652 | 0.563683 | -0.063656 | 1.875992 | 0.562429 | -0.031043 | 1.878317 | 0.565650 | -0.055120 |
| C | 0.470545 | -2.822011 | -0.797117 | 0.492012 | -2.829268 | -0.819358 | 0.510325 | -2.829891 | -0.840902 | 0.503651 | -2.790113 | -1.002817 | 0.502573 | -2.826419 | -0.847034 | 0.494371 | -2.821941 | -0.859943 |
| C | 1.237611 | -3.962949 | -0.989552 | 1.267710 | -3.964532 | -1.011513 | 1.294429 | -3.960347 | -1.031292 | 1.284762 | -3.918419 | -1.214517 | 1.283392 | -3.959059 | -1.038776 | 1.271791 | -3.956571 | -1.051368 |
| C | 2.616721 | -3.923784 | -0.858828 | 2.645398 | -3.918334 | -0.861830 | 2.671173 | -3.907775 | -0.861656 | 2.657449 | -3.881297 | -1.009616 | 2.660157 | -3.911282 | -0.866704 | 2.648183 | -3.912528 | -0.883141 |
| C | 3.265637 | -2.736233 | -0.547510 | 3.284213 | -2.729328 | -0.534331 | 3.300761 | -2.717270 | -0.519867 | 3.288216 | -2.708069 | -0.612992 | 3.293142 | -2.723366 | -0.521522 | 3.284482 | -2.726061 | -0.540168 |
| O | 2.019121 | 1.759203 | 0.100652 | 1.996264 | 1.745757 | 0.181043 | 1.971699 | 1.739289 | 0.244336 | 1.960439 | 1.736368 | 0.240966 | 1.977733 | 1.738306 | 0.238479 | 1.987265 | 1.743311 | 0.199175 |
| C | -4.278440 | -0.072690 | 0.203356 | -4.280493 | -0.079241 | 0.200915 | -4.278962 | -0.085827 | 0.208227 | -4.258640 | -0.084559 | 0.240964 | -4.277785 | -0.082069 | 0.207788 | -4.276459 | -0.079025 | 0.208174 |
| C | -4.655878 | -0.761265 | 1.346946 | -4.685473 | -0.560885 | 1.436854 | -4.718408 | -0.343595 | 1.498162 | -4.614850 | -0.509674 | 1.512232 | -4.714556 | -0.348668 | 1.497010 | -4.669221 | -0.550374 | 1.452198 |
| C | -5.967519 | -1.172283 | 1.508946 | -5.998654 | -0.953678 | 1.631449 | -6.033625 | -0.718066 | 1.718512 | -5.917895 | -0.902853 | 1.768677 | -6.028601 | -0.727901 | 1.717132 | -5.980149 | -0.944636 | 1.660936 |
| C | -6.884533 | -0.872564 | 0.520789 | -6.883100 | -0.841971 | 0.577293 | -6.878095 | -0.815339 | 0.630774 | -6.834769 | -0.846949 | 0.738249 | -6.874582 | -0.821072 | 0.630018 | -6.871982 | -0.844124 | 0.611945 |
| C | -6.532040 | -0.187646 | -0.625387 | -6.504859 | -0.361627 | -0.660532 | -6.465762 | -0.558973 | -0.661369 | -6.504861 | -0.422904 | -0.533319 | -6.464718 | -0.556438 | -0.661417 | -6.506237 | -0.374272 | -0.633550 |
| C | -5.213688 | 0.204179 | -0.784141 | -5.185617 | 0.015749 | -0.846421 | -5.146168 | -0.192362 | -0.869106 | -5.196067 | -0.043509 | -0.780843 | -5.146208 | -0.185434 | -0.869077 | -5.189305 | 0.005009 | -0.833448 |
| F | -8.155909 | -1.260631 | 0.677238 | -8.158768 | -1.214764 | 0.762259 | -8.157468 | -1.174495 | 0.839052 | -8.102863 | -1.221457 | 0.983511 | -8.152692 | -1.184709 | 0.838107 | -8.146771 | -1.218851 | 0.810766 |
| C | 4.287465 | 0.052941 | 0.135733 | 4.284998 | 0.061307 | 0.164022 | 4.280003 | 0.072805 | 0.188753 | 4.260498 | 0.064582 | 0.213699 | 4.279705 | 0.066907 | 0.189544 | 4.279369 | 0.061714 | 0.177315 |
| C | 4.904465 | 1.008144 | -0.659165 | 4.927335 | 0.936078 | -0.700048 | 4.951805 | 0.865721 | -0.730221 | 4.929729 | 0.950240 | -0.618432 | 4.948618 | 0.865293 | -0.727014 | 4.939394 | 0.903692 | -0.705946 |
| C | 6.216350 | 1.374371 | -0.413145 | 6.238922 | 1.309047 | -0.461176 | 6.263853 | 1.240295 | -0.492295 | 6.232318 | 1.320744 | -0.328438 | 6.260570 | 1.240991 | -0.489686 | 6.250271 | 1.275847 | -0.459858 |
| C | 6.894391 | 0.763233 | 0.623007 | 6.885024 | 0.781908 | 0.638864 | 6.874075 | 0.794663 | 0.662783 | 6.836242 | 0.778155 | 0.787722 | 6.873553 | 0.791039 | 0.662465 | 6.874519 | 0.779910 | 0.666993 |
| C | 6.301716 | -0.191310 | 1.425801 | 6.268975 | -0.093718 | 1.510345 | 6.229675 | -0.001145 | 1.588375 | 6.193198 | -0.109637 | 1.626652 | 6.231825 | -0.009859 | 1.585764 | 6.241010 | -0.063341 | 1.557708 |
| C | 4.983931 | -0.537678 | 1.181396 | 4.952143 | -0.447366 | 1.269742 | 4.913713 | -0.358421 | 1.345559 | 4.886048 | -0.461838 | 1.335025 | 4.915813 | -0.368082 | 1.343743 | 4.925488 | -0.417018 | 1.308502 |
| F | 8.166197 | 1.108223 | 0.858350 | 8.159324 | 1.133972 | 0.870120 | 8.150659 | 1.149804 | 0.895635 | 8.103964 | 1.128454 | 1.069277 | 8.149933 | 1.147199 | 0.894790 | 8.149050 | 1.131752 | 0.906442 |
| H | 0.564686 | 2.984692 | -0.635074 | 0.561927 | 2.959791 | -0.704545 | 0.556194 | 2.926777 | -0.811051 | 0.548758 | 2.920191 | -0.949563 | 0.560322 | 2.932007 | -0.802372 | 0.560732 | 2.952515 | -0.761457 |
| H | -0.801894 | 5.024301 | -0.717715 | -0.808997 | 4.992723 | -0.841175 | -0.820848 | 4.952399 | -1.005575 | -0.824470 | 4.951672 | -1.108439 | -0.815332 | 4.959318 | -0.997177 | -0.809458 | 4.986419 | -0.900412 |
| H | -3.240083 | 4.914646 | -0.366246 | -3.249720 | 4.884816 | -0.504656 | -3.261282 | 4.848422 | -0.661918 | -3.251626 | 4.861846 | -0.681493 | -3.256755 | 4.856120 | -0.659482 | -3.247510 | 4.883530 | -0.540237 |
| H | -4.351706 | 2.728137 | 0.001283 | -4.360276 | 2.703145 | -0.096366 | -4.368266 | 2.677170 | -0.188200 | -4.355881 | 2.702041 | -0.162197 | -4.365889 | 2.684736 | -0.190171 | -4.358033 | 2.706997 | -0.108036 |
| H | -0.599640 | -2.869960 | -0.893601 | -0.576788 | -2.881839 | -0.931570 | -0.556717 | -2.885375 | -0.973065 | -0.558774 | -2.830496 | -1.177722 | -0.564359 | -2.878083 | -0.982217 | -0.572888 | -2.872094 | -0.990317 |
| H | 4.343160 | -2.688552 | -0.471919 | 4.360469 | -2.678206 | -0.441162 | 4.375675 | -2.662130 | -0.410600 | 4.361379 | -2.664445 | -0.483394 | 4.368050 | -2.671698 | -0.410937 | 4.359691 | -2.676579 | -0.432896 |
| H | 0.746227 | -4.892816 | -1.243033 | 0.785128 | -4.895486 | -1.278871 | 0.821698 | -4.892152 | -1.314154 | 0.814360 | -4.835426 | -1.545537 | 0.808153 | -4.888610 | -1.324741 | 0.792507 | -4.885119 | -1.333261 |
| H | 3.200160 | -4.823145 | -1.010300 | 3.235556 | -4.813905 | -1.011125 | 3.267609 | -4.799828 | -1.007995 | 3.251375 | -4.771689 | -1.175346 | 3.253949 | -4.804900 | -1.014130 | 3.239306 | -4.807796 | -1.031043 |
| H | -3.916701 | -0.985038 | 2.103441 | -3.969447 | -0.632616 | 2.244666 | -4.030480 | -0.251506 | 2.328738 | -3.872650 | -0.534222 | 2.299778 | -4.025411 | -0.260046 | 2.326935 | -3.946691 | -0.612186 | 2.255353 |
| H | -4.906412 | 0.727213 | -1.680432 | -4.855024 | 0.385384 | -1.808536 | -4.787820 | 0.013080 | -1.869724 | -4.901928 | 0.286565 | -1.769037 | -4.789704 | 0.025894 | -1.869105 | -4.867980 | 0.367816 | -1.801363 |
| H | -6.283161 | -1.715665 | 2.388821 | -6.337893 | -1.337931 | 2.583798 | -6.401181 | -0.927419 | 2.714284 | -6.221301 | -1.241760 | 2.750388 | -6.394134 | -0.944299 | 2.712129 | -6.310454 | -1.320448 | 2.619970 |
| H | -7.277932 | 0.019690 | -1.380238 | -7.229713 | -0.295911 | -1.460637 | -7.162852 | -0.648476 | -1.483911 | -7.255873 | -0.397531 | -1.311868 | -7.162735 | -0.643533 | -1.483419 | -7.237365 | -0.316579 | -1.428782 |
| H | 4.351531 | 1.472494 | -1.463574 | 4.397686 | 1.328864 | -1.557442 | 4.448401 | 1.189170 | -1.631968 | 4.431849 | 1.350100 | -1.492305 | 4.442232 | 1.192476 | -1.625783 | 4.425311 | 1.269996 | -1.584735 |
| H | 4.490335 | -1.270498 | 1.806431 | 4.438262 | -1.121174 | 1.943198 | 4.377121 | -0.975304 | 2.055130 | 4.351384 | -1.148436 | 1.978985 | 4.381223 | -0.988475 | 2.051721 | 4.397522 | -1.067352 | 1.994175 |
| H | 6.715054 | 2.120827 | -1.015817 | 6.758734 | 1.993999 | -1.117265 | 6.808307 | 1.860636 | -1.191773 | 6.774239 | 2.012427 | -0.959776 | 6.802800 | 1.865662 | -1.187012 | 6.784498 | 1.935192 | -1.130715 |
| H | 6.863102 | -0.640833 | 2.233213 | 6.809930 | -0.478270 | 2.364371 | 6.746537 | -0.326311 | 2.481480 | 6.703618 | -0.508756 | 2.493168 | 6.750694 | -0.337765 | 2.476689 | 6.766610 | -0.424523 | 2.431514 |

| **iso-I 2** |  | gasphase |  |  | toluene |  |  | DMSO |  |  | methanol |  |  | acetonitrile |  |  | chloroform |  |
| --- | --- | --- | --- | --- | --- | --- | --- | --- | --- | --- | --- | --- | --- | --- | --- | --- | --- | --- |
|  | x | y | z | x | y | z | x | y | z | x | y | z | x | y | z | x | y | z |
| C | 0.935631 | -2.801657 | -0.922641 | 0.928370 | -2.799925 | -0.863120 | 0.935311 | -2.774882 | -0.929951 | 0.938841 | -2.760879 | -1.025100 | 0.932505 | -2.776285 | -0.931575 | 0.920539 | -2.784138 | -0.895261 |
| C | 1.678743 | -3.968239 | -1.037629 | 1.670302 | -3.965893 | -0.992250 | 1.679875 | -3.937487 | -1.077856 | 1.684175 | -3.921299 | -1.183909 | 1.675995 | -3.939709 | -1.079763 | 1.659673 | -3.951095 | -1.034116 |
| C | 3.043482 | -3.968460 | -0.795731 | 3.038233 | -3.966816 | -0.764492 | 3.046357 | -3.942182 | -0.831956 | 3.046256 | -3.933155 | -0.914463 | 3.042733 | -3.945574 | -0.834616 | 3.027229 | -3.958408 | -0.799530 |
| C | 3.703281 | -2.797051 | -0.447729 | 3.702195 | -2.796254 | -0.421122 | 3.706753 | -2.778984 | -0.456213 | 3.703421 | -2.779130 | -0.504611 | 3.704423 | -2.782768 | -0.459210 | 3.693880 | -2.793273 | -0.441797 |
| C | 2.951486 | -1.646753 | -0.336280 | 2.950988 | -1.646369 | -0.299120 | 2.952158 | -1.632723 | -0.317694 | 2.947454 | -1.636701 | -0.352606 | 2.950914 | -1.635834 | -0.320128 | 2.945027 | -1.642710 | -0.310898 |
| C | 1.563972 | -1.617973 | -0.549541 | 1.560571 | -1.617723 | -0.491867 | 1.563904 | -1.603961 | -0.519857 | 1.563477 | -1.602921 | -0.575189 | 1.562527 | -1.605833 | -0.521794 | 1.555528 | -1.609065 | -0.506491 |
| N | 3.422031 | -0.361675 | -0.065777 | 3.423255 | -0.362824 | -0.028280 | 3.417279 | -0.352891 | -0.019571 | 3.406983 | -0.357737 | -0.026653 | 3.417297 | -0.355700 | -0.022697 | 3.418938 | -0.361556 | -0.029013 |
| C | 2.400863 | 0.561141 | -0.148949 | 2.401374 | 0.556966 | -0.078817 | 2.394066 | 0.562806 | -0.054449 | 2.386454 | 0.552512 | -0.067564 | 2.394988 | 0.560243 | -0.059307 | 2.400168 | 0.559100 | -0.074382 |
| C | 1.129689 | -0.220721 | -0.431064 | 1.128055 | -0.221158 | -0.361290 | 1.128395 | -0.212800 | -0.367931 | 1.128396 | -0.215030 | -0.409803 | 1.128483 | -0.213902 | -0.370458 | 1.126967 | -0.212979 | -0.365701 |
| C | -0.097239 | 0.357522 | -0.468851 | -0.098759 | 0.356729 | -0.398709 | -0.100707 | 0.355622 | -0.407091 | -0.099486 | 0.353720 | -0.450928 | -0.099910 | 0.356409 | -0.408014 | -0.098982 | 0.365217 | -0.399106 |
| O | 2.545824 | 1.742761 | 0.045001 | 2.543641 | 1.735291 | 0.144540 | 2.523131 | 1.735136 | 0.215667 | 2.507086 | 1.725041 | 0.226685 | 2.525205 | 1.733628 | 0.207561 | 2.541506 | 1.736297 | 0.164335 |
| C | -0.521482 | 1.760429 | -0.566039 | -0.520904 | 1.758082 | -0.516565 | -0.524514 | 1.751752 | -0.543811 | -0.520993 | 1.747884 | -0.595407 | -0.522989 | 1.752979 | -0.544895 | -0.519931 | 1.765353 | -0.523583 |
| C | -1.920091 | 1.790535 | -0.457374 | -1.920581 | 1.789899 | -0.422998 | -1.923420 | 1.781971 | -0.448724 | -1.917525 | 1.781805 | -0.483304 | -1.922014 | 1.783868 | -0.449608 | -1.919472 | 1.797971 | -0.430191 |
| N | -2.412277 | 0.493962 | -0.273660 | -2.414097 | 0.495837 | -0.227761 | -2.412767 | 0.489751 | -0.233823 | -2.404717 | 0.488468 | -0.251999 | -2.411956 | 0.491536 | -0.234049 | -2.412641 | 0.504514 | -0.228209 |
| C | -1.380301 | -0.430413 | -0.321220 | -1.383061 | -0.426614 | -0.239578 | -1.381100 | -0.428332 | -0.224188 | -1.375991 | -0.425139 | -0.248403 | -1.380519 | -0.426092 | -0.223048 | -1.382252 | -0.415642 | -0.226830 |
| C | 0.137614 | 2.950754 | -0.854504 | 0.141956 | 2.943728 | -0.815656 | 0.139018 | 2.928796 | -0.873424 | 0.142110 | 2.915734 | -0.956103 | 0.141082 | 2.929912 | -0.874583 | 0.144489 | 2.947646 | -0.832914 |
| C | -0.589897 | 4.124415 | -0.991976 | -0.582434 | 4.116628 | -0.973877 | -0.586168 | 4.098787 | -1.050432 | -0.582777 | 4.085215 | -1.136305 | -0.583688 | 4.100372 | -1.051496 | -0.579160 | 4.120431 | -0.997365 |
| C | -1.969340 | 4.125087 | -0.861411 | -1.963372 | 4.120175 | -0.852133 | -1.967992 | 4.103761 | -0.918247 | -1.962398 | 4.095772 | -0.981441 | -1.965608 | 4.105881 | -0.919420 | -1.960584 | 4.125600 | -0.872419 |
| C | -2.657186 | 2.947315 | -0.600827 | -2.655066 | 2.946334 | -0.583721 | -2.659707 | 2.935292 | -0.625800 | -2.654156 | 2.932962 | -0.665534 | -2.657804 | 2.937460 | -0.626974 | -2.653558 | 2.953855 | -0.596563 |
| O | -1.541929 | -1.617148 | -0.184536 | -1.541865 | -1.609676 | -0.062016 | -1.528520 | -1.606833 | -0.000332 | -1.517612 | -1.605014 | -0.006353 | -1.527751 | -1.604456 | 0.003151 | -1.537019 | -1.596619 | -0.024396 |
| C | 4.755408 | -0.030393 | 0.281973 | 4.769369 | -0.030392 | 0.274462 | 4.762445 | -0.025454 | 0.295377 | 4.744391 | -0.029304 | 0.321007 | 4.762139 | -0.027552 | 0.294462 | 4.765437 | -0.034552 | 0.280870 |
| C | 5.343724 | -0.619664 | 1.392699 | 5.355251 | -0.519773 | 1.433392 | 5.325599 | -0.495134 | 1.473451 | 5.306017 | -0.563163 | 1.472069 | 5.324239 | -0.498714 | 1.472620 | 5.340232 | -0.522025 | 1.446209 |
| C | 6.648018 | -0.309902 | 1.737510 | 6.671224 | -0.209119 | 1.730962 | 6.640881 | -0.190041 | 1.782088 | 6.612475 | -0.253535 | 1.811284 | 6.638693 | -0.192221 | 1.783957 | 6.656071 | -0.216625 | 1.750945 |
| C | 7.335833 | 0.607269 | 0.967427 | 7.368349 | 0.604498 | 0.860402 | 7.355931 | 0.594279 | 0.899767 | 7.319245 | 0.600401 | 0.988656 | 7.353921 | 0.595392 | 0.904468 | 7.362133 | 0.588533 | 0.879840 |
| C | 6.765427 | 1.216215 | -0.132989 | 6.803857 | 1.110904 | -0.293151 | 6.816486 | 1.077960 | -0.275092 | 6.779339 | 1.149768 | -0.156698 | 6.815298 | 1.081131 | -0.270096 | 6.809639 | 1.092223 | -0.280603 |
| C | 5.466462 | 0.886446 | -0.478531 | 5.492033 | 0.781113 | -0.587651 | 5.504490 | 0.754843 | -0.579301 | 5.476799 | 0.821691 | -0.493918 | 5.504183 | 0.756492 | -0.577167 | 5.497537 | 0.768030 | -0.581912 |
| F | 8.594530 | 0.916698 | 1.299509 | 8.641996 | 0.914821 | 1.146407 | 8.632241 | 0.899115 | 1.196444 | 8.586631 | 0.909198 | 1.316339 | 8.629255 | 0.901612 | 1.203808 | 8.637221 | 0.893863 | 1.172982 |
| C | -3.751608 | 0.150873 | 0.002808 | -3.761309 | 0.153780 | 0.017328 | -3.759582 | 0.151096 | 0.021214 | -3.747907 | 0.150699 | 0.025953 | -3.758774 | 0.152137 | 0.021951 | -3.759843 | 0.161236 | 0.018216 |
| C | -4.372495 | -0.866676 | -0.712488 | -4.375372 | -0.833119 | -0.744680 | -4.378162 | -0.833768 | -0.739604 | -4.373723 | -0.843520 | -0.716459 | -4.377052 | -0.833515 | -0.738380 | -4.370381 | -0.826345 | -0.745818 |
| C | -5.682203 | -1.205601 | -0.432404 | -5.691414 | -1.173130 | -0.499230 | -5.692147 | -1.174239 | -0.484801 | -5.682861 | -1.184430 | -0.439347 | -5.691037 | -1.174454 | -0.483241 | -5.683891 | -1.173988 | -0.497525 |
| C | -6.357143 | -0.513443 | 0.556235 | -6.377939 | -0.509949 | 0.502118 | -6.369366 | -0.511364 | 0.524095 | -6.345601 | -0.510924 | 0.572389 | -6.368162 | -0.511298 | 0.525681 | -6.369664 | -0.515473 | 0.508072 |
| C | -5.756169 | 0.499309 | 1.281061 | -5.783497 | 0.474047 | 1.271783 | -5.770433 | 0.474300 | 1.289178 | -5.739026 | 0.484541 | 1.319200 | -5.769375 | 0.474561 | 1.290786 | -5.779380 | 0.472123 | 1.276926 |
| C | -4.442731 | 0.825007 | 1.004819 | -4.463566 | 0.800106 | 1.029050 | -4.452355 | 0.800517 | 1.036771 | -4.425805 | 0.810353 | 1.044648 | -4.451405 | 0.801321 | 1.037861 | -4.461575 | 0.804898 | 1.031612 |
| N | -7.755061 | -0.865920 | 0.848009 | -7.780478 | -0.861816 | 0.756491 | -7.765993 | -0.865322 | 0.790289 | -7.733940 | -0.863146 | 0.861845 | -7.764896 | -0.864775 | 0.791921 | -7.766397 | -0.877308 | 0.768898 |
| O | -8.325859 | -0.231786 | 1.709053 | -8.367118 | -0.256642 | 1.629375 | -8.354209 | -0.256730 | 1.661896 | -8.320855 | -0.235975 | 1.721530 | -8.349848 | -0.263803 | 1.670612 | -8.362432 | -0.264692 | 1.630954 |
| O | -8.251959 | -1.767924 | 0.209295 | -8.277155 | -1.737180 | 0.079099 | -8.267899 | -1.750257 | 0.126133 | -8.242821 | -1.768035 | 0.230281 | -8.271009 | -1.741616 | 0.120779 | -8.256584 | -1.770742 | 0.109542 |
| H | -0.124110 | -2.818166 | -1.105325 | -0.133530 | -2.816875 | -1.035405 | -0.123822 | -2.786494 | -1.123854 | -0.114965 | -2.763820 | -1.250286 | -0.126761 | -2.786582 | -1.125316 | -0.140370 | -2.795651 | -1.076987 |
| H | 1.180876 | -4.886762 | -1.317966 | 1.169367 | -4.883666 | -1.270921 | 1.185094 | -4.848986 | -1.387660 | 1.194618 | -4.824858 | -1.523908 | 1.180229 | -4.850757 | -1.389256 | 1.157905 | -4.864336 | -1.326483 |
| H | 3.608218 | -4.887504 | -0.887768 | 3.601580 | -4.886185 | -0.864033 | 3.611583 | -4.858962 | -0.945427 | 3.612026 | -4.848204 | -1.039112 | 3.607125 | -4.862804 | -0.948482 | 3.588214 | -4.878514 | -0.906279 |
| H | 4.771983 | -2.782082 | -0.284750 | 4.772491 | -2.783290 | -0.266493 | 4.775551 | -2.770113 | -0.289270 | 4.770140 | -2.775643 | -0.324681 | 4.773287 | -2.774661 | -0.292919 | 4.763763 | -2.785682 | -0.282989 |
| H | 1.208475 | 2.964729 | -0.953919 | 1.213713 | 2.955635 | -0.909541 | 1.210303 | 2.936800 | -0.982178 | 1.210445 | 2.917203 | -1.096720 | 1.212347 | 2.937103 | -0.983517 | 1.216137 | 2.957266 | -0.932968 |
| H | -3.735885 | 2.936905 | -0.532335 | -3.734316 | 2.940734 | -0.520640 | -3.738707 | 2.932369 | -0.555252 | -3.732349 | 2.933340 | -0.582666 | -3.736736 | 2.934768 | -0.556154 | -3.732738 | 2.950175 | -0.530876 |
| H | -0.068207 | 5.047817 | -1.204999 | -0.058020 | 5.036912 | -1.195231 | -0.064908 | 5.014945 | -1.296568 | -0.063787 | 4.996159 | -1.405553 | -0.061992 | 5.016319 | -1.297416 | -0.054853 | 5.039014 | -1.226688 |
| H | -2.523405 | 5.048154 | -0.974382 | -2.515331 | 5.043017 | -0.978580 | -2.520715 | 5.024430 | -1.058005 | -2.514368 | 5.016328 | -1.125233 | -2.517998 | 5.026743 | -1.059129 | -2.511930 | 5.048340 | -1.003067 |
| H | 4.776563 | -1.322284 | 1.989361 | 4.778679 | -1.144301 | 2.103552 | 4.735010 | -1.101280 | 2.148427 | 4.722430 | -1.222047 | 2.102275 | 4.733553 | -1.107177 | 2.145378 | 4.756583 | -1.141196 | 2.115284 |
| H | 4.996962 | 1.349567 | -1.335017 | 5.024506 | 1.158240 | -1.487260 | 5.055964 | 1.108832 | -1.498279 | 5.028174 | 1.227523 | -1.391326 | 5.056140 | 1.112649 | -1.495529 | 5.039013 | 1.141701 | -1.487849 |
| H | 7.126520 | -0.758783 | 2.596920 | 7.150179 | -0.579684 | 2.627117 | 7.103220 | -0.546763 | 2.692816 | 7.074016 | -0.659728 | 2.701549 | 7.100227 | -0.550095 | 2.694628 | 7.126826 | -0.586137 | 2.652055 |
| H | 7.336450 | 1.932558 | -0.707287 | 7.386158 | 1.746455 | -0.946424 | 7.414837 | 1.687532 | -0.939083 | 7.370128 | 1.813536 | -0.773977 | 7.413590 | 1.693599 | -0.931442 | 7.399598 | 1.720302 | -0.934512 |
| H | -3.826417 | -1.393431 | -1.480657 | -3.823226 | -1.332596 | -1.527377 | -3.835258 | -1.327002 | -1.533181 | -3.840178 | -1.342495 | -1.513339 | -3.833801 | -1.326775 | -1.531741 | -3.818251 | -1.319069 | -1.533310 |
| H | -3.944779 | 1.598165 | 1.573939 | -3.972027 | 1.551764 | 1.631690 | -3.958306 | 1.555137 | 1.633665 | -3.924237 | 1.571336 | 1.627048 | -3.957439 | 1.556463 | 1.634102 | -3.972585 | 1.558736 | 1.633624 |
| H | -6.185422 | -1.991907 | -0.975130 | -6.185456 | -1.937088 | -1.080965 | -6.186741 | -1.936094 | -1.068947 | -6.183731 | -1.953125 | -1.009278 | -6.185545 | -1.936859 | -1.066769 | -6.173745 | -1.939069 | -1.081536 |
| H | -6.310553 | 1.008714 | 2.055129 | -6.342612 | 0.964289 | 2.054896 | -6.319028 | 0.969729 | 2.076497 | -6.276307 | 0.986935 | 2.109992 | -6.318147 | 0.969673 | 2.078169 | -6.336872 | 0.960473 | 2.062443 |

| **iso-I** 3 |  | gasphase |  |  | toluene |  |  | DMSO |  |  | methanol |  |  | acetonitrile |  |  | chloroform |  |
| --- | --- | --- | --- | --- | --- | --- | --- | --- | --- | --- | --- | --- | --- | --- | --- | --- | --- | --- |
| Element | x | y | z | x | y | z | x | y | z | x | y | z | x | y | z | x | y | z |
| C | 0.642765 | -2.828883 | -0.893868 | 0.635634 | -2.827089 | -0.836474 | 0.645771 | -2.805366 | -0.904598 | 0.647214 | -2.787153 | -1.004470 | 0.641217 | -2.801862 | -0.915986 | 0.630326 | -2.816704 | -0.855664 |
| C | 1.405530 | -3.981806 | -1.018130 | 1.397191 | -3.979254 | -0.975445 | 1.411444 | -3.953023 | -1.061664 | 1.412089 | -3.934011 | -1.169190 | 1.404620 | -3.951231 | -1.072719 | 1.390117 | -3.969523 | -1.001710 |
| C | 2.773669 | -3.956257 | -0.797787 | 2.768139 | -3.954498 | -0.768246 | 2.781214 | -3.929683 | -0.836093 | 2.777605 | -3.919378 | -0.917817 | 2.774120 | -3.931217 | -0.844157 | 2.761116 | -3.948949 | -0.789350 |
| C | 3.416934 | -2.771977 | -0.462638 | 3.415212 | -2.771309 | -0.436025 | 3.423398 | -2.752746 | -0.471626 | 3.418379 | -2.751568 | -0.521239 | 3.418411 | -2.755911 | -0.477416 | 3.410222 | -2.769331 | -0.447059 |
| C | 2.645706 | -1.635548 | -0.341665 | 2.644555 | -1.635490 | -0.303970 | 2.647891 | -1.621755 | -0.323469 | 2.643096 | -1.622938 | -0.363534 | 2.645184 | -1.623274 | -0.329623 | 2.640919 | -1.633262 | -0.308651 |
| C | 1.254805 | -1.632937 | -0.533130 | 1.251183 | -1.632871 | -0.476017 | 1.256619 | -1.621292 | -0.505658 | 1.255946 | -1.616098 | -0.566926 | 1.254214 | -1.619749 | -0.514136 | 1.248077 | -1.627655 | -0.482028 |
| N | 3.096536 | -0.341359 | -0.081126 | 3.096783 | -0.343226 | -0.040665 | 3.091323 | -0.332484 | -0.033048 | 3.082966 | -0.334093 | -0.049860 | 3.090823 | -0.334234 | -0.038837 | 3.093925 | -0.342159 | -0.038387 |
| C | 2.057107 | 0.562214 | -0.149896 | 2.057163 | 0.557269 | -0.075700 | 2.049283 | 0.562271 | -0.053373 | 2.045351 | 0.557170 | -0.081741 | 2.050989 | 0.562386 | -0.064105 | 2.056512 | 0.558041 | -0.069468 |
| C | 0.796389 | -0.243632 | -0.409906 | 0.794575 | -0.244393 | -0.339202 | 0.795250 | -0.238911 | -0.348947 | 0.796762 | -0.236121 | -0.399233 | 0.795557 | -0.236330 | -0.357569 | 0.794187 | -0.239619 | -0.338337 |
| C | -0.441661 | 0.311392 | -0.427635 | -0.443198 | 0.310511 | -0.358280 | -0.445343 | 0.304845 | -0.371602 | -0.443079 | 0.307294 | -0.417341 | -0.444291 | 0.309310 | -0.376326 | -0.443090 | 0.314734 | -0.353854 |
| O | 2.183649 | 1.746813 | 0.038881 | 2.181059 | 1.738057 | 0.145779 | 2.158539 | 1.736980 | 0.215180 | 2.149318 | 1.733800 | 0.202563 | 2.162609 | 1.738477 | 0.199227 | 2.178919 | 1.738505 | 0.163586 |
| C | -0.893652 | 1.706266 | -0.516977 | -0.892730 | 1.704141 | -0.468040 | -0.897889 | 1.692774 | -0.501325 | -0.895813 | 1.692567 | -0.554345 | -0.896897 | 1.697384 | -0.505377 | -0.892251 | 1.707011 | -0.471313 |
| C | -2.290555 | 1.709505 | -0.384267 | -2.291272 | 1.709283 | -0.353908 | -2.295144 | 1.695819 | -0.381035 | -2.289749 | 1.697697 | -0.408722 | -2.294106 | 1.700455 | -0.382515 | -2.290338 | 1.712673 | -0.353250 |
| N | -2.754812 | 0.404298 | -0.193020 | -2.757513 | 0.406564 | -0.153675 | -2.755539 | 0.394897 | -0.158799 | -2.744314 | 0.395877 | -0.164024 | -2.754040 | 0.399270 | -0.158223 | -2.755060 | 0.410673 | -0.144311 |
| C | -1.707844 | -0.500437 | -0.258570 | -1.711211 | -0.496488 | -0.181230 | -1.708307 | -0.503493 | -0.168918 | -1.699965 | -0.496611 | -0.185303 | -1.706561 | -0.498120 | -0.168202 | -1.709691 | -0.490044 | -0.161682 |
| C | -0.262452 | 2.909451 | -0.814400 | -0.256888 | 2.902941 | -0.773323 | -0.263516 | 2.882625 | -0.841978 | -0.266059 | 2.873732 | -0.930715 | -0.262902 | 2.887289 | -0.847163 | -0.256592 | 2.902466 | -0.790326 |
| C | -1.014743 | 4.069181 | -0.937051 | -1.005861 | 4.062251 | -0.917469 | -1.014557 | 4.038574 | -1.003616 | -1.019462 | 4.027979 | -1.092676 | -1.014311 | 4.043380 | -1.007699 | -1.005903 | 4.061324 | -0.939053 |
| C | -2.391450 | 4.043307 | -0.781997 | -2.384632 | 4.039419 | -0.774506 | -2.393455 | 4.016906 | -0.844564 | -2.394716 | 4.010195 | -0.903102 | -2.393083 | 4.021632 | -0.846677 | -2.384575 | 4.039892 | -0.787680 |
| C | -3.052069 | 2.852186 | -0.511636 | -3.049985 | 2.852015 | -0.499096 | -3.056975 | 2.834913 | -0.541161 | -3.054492 | 2.833346 | -0.570676 | -3.056170 | 2.839463 | -0.541974 | -3.049756 | 2.854466 | -0.501793 |
| O | -1.845298 | -1.690287 | -0.120910 | -1.845989 | -1.682804 | -0.003809 | -1.830365 | -1.685504 | 0.053363 | -1.813348 | -1.680078 | 0.056991 | -1.827138 | -1.679763 | 0.058317 | -1.840396 | -1.674563 | 0.039060 |
| C | 4.428174 | 0.014987 | 0.247671 | 4.440577 | 0.014170 | 0.243304 | 4.433600 | 0.021859 | 0.264811 | 4.417483 | 0.018605 | 0.285178 | 4.432496 | 0.018735 | 0.264874 | 4.438905 | 0.012435 | 0.247111 |
| C | 5.043078 | -0.563428 | 1.349693 | 5.050494 | -0.461212 | 1.395672 | 5.021066 | -0.435411 | 1.435970 | 4.995159 | -0.496737 | 1.436839 | 5.014443 | -0.439868 | 1.438446 | 5.041273 | -0.453225 | 1.407456 |
| C | 6.345937 | -0.229254 | 1.676607 | 6.364437 | -0.125821 | 1.674922 | 6.333752 | -0.103559 | 1.727620 | 6.298727 | -0.163930 | 1.764816 | 6.326028 | -0.108874 | 1.736507 | 6.355921 | -0.120441 | 1.687917 |
| C | 7.005443 | 0.701072 | 0.897680 | 7.035110 | 0.697808 | 0.793011 | 7.021615 | 0.694284 | 0.835786 | 6.986575 | 0.693895 | 0.930311 | 7.018372 | 0.689897 | 0.848733 | 7.032778 | 0.689289 | 0.797985 |
| C | 6.408270 | 1.299638 | -0.194205 | 6.446317 | 1.190641 | -0.354202 | 6.457642 | 1.166020 | -0.332340 | 6.430595 | 1.225071 | -0.215920 | 6.459722 | 1.163492 | -0.321372 | 6.452466 | 1.171608 | -0.357898 |
| C | 5.111044 | 0.945555 | -0.521968 | 5.136915 | 0.836304 | -0.630338 | 5.148641 | 0.816316 | -0.619580 | 5.131055 | 0.873979 | -0.541675 | 5.151802 | 0.814492 | -0.615122 | 5.141991 | 0.820290 | -0.634699 |
| F | 8.262624 | 1.034193 | 1.212393 | 8.306653 | 1.031897 | 1.061232 | 8.295210 | 1.024957 | 1.116065 | 8.251103 | 1.025225 | 1.246801 | 8.290761 | 1.019755 | 1.135231 | 8.306531 | 1.020884 | 1.067541 |
| C | -4.084503 | 0.036681 | 0.103385 | -4.096696 | 0.040210 | 0.106672 | -4.093845 | 0.032828 | 0.116190 | -4.078204 | 0.032304 | 0.136233 | -4.091637 | 0.035011 | 0.118891 | -4.095224 | 0.043015 | 0.113394 |
| C | -4.764858 | 0.680645 | 1.131050 | -4.790722 | 0.656691 | 1.141341 | -4.770510 | 0.649922 | 1.161519 | -4.737293 | 0.652679 | 1.190242 | -4.768681 | 0.651231 | 1.164554 | -4.787265 | 0.648940 | 1.155178 |
| C | -6.067494 | 0.329917 | 1.425411 | -6.101263 | 0.305933 | 1.398300 | -6.079194 | 0.302829 | 1.432794 | -6.040470 | 0.303119 | 1.484370 | -6.076403 | 0.301043 | 1.437451 | -6.097365 | 0.293901 | 1.409420 |
| C | -6.693184 | -0.682225 | 0.701563 | -6.716426 | -0.677195 | 0.626369 | -6.704158 | -0.674889 | 0.661329 | -6.675077 | -0.679391 | 0.727062 | -6.699827 | -0.679001 | 0.667449 | -6.710490 | -0.681705 | 0.626006 |
| C | -6.004520 | -1.336687 | -0.316975 | -6.015606 | -1.303089 | -0.402309 | -6.022446 | -1.299087 | -0.381628 | -6.009834 | -1.306914 | -0.324184 | -6.017448 | -1.302627 | -0.375615 | -6.012172 | -1.295968 | -0.411721 |
| C | -4.706177 | -0.974852 | -0.618105 | -4.709320 | -0.939773 | -0.664035 | -4.717715 | -0.938866 | -0.656068 | -4.710755 | -0.944364 | -0.621991 | -4.713783 | -0.939293 | -0.651735 | -4.706307 | -0.927971 | -0.669859 |
| C | -8.043742 | -1.050498 | 1.006241 | -8.074494 | -1.046028 | 0.891935 | -8.058596 | -1.040736 | 0.942362 | -8.023637 | -1.046755 | 1.031289 | -8.053382 | -1.047928 | 0.950117 | -8.067009 | -1.055805 | 0.889722 |
| N | -9.127368 | -1.343594 | 1.249708 | -9.164480 | -1.339982 | 1.103821 | -9.146485 | -1.334331 | 1.167779 | -9.107295 | -1.341488 | 1.275123 | -9.140444 | -1.343915 | 1.176685 | -9.156148 | -1.354682 | 1.100552 |
| H | -0.419295 | -2.865205 | -1.059616 | -0.428282 | -2.863731 | -0.992389 | -0.415761 | -2.838338 | -1.082356 | -0.409340 | -2.810469 | -1.215234 | -0.420040 | -2.831837 | -1.096681 | -0.432947 | -2.849525 | -1.019845 |
| H | 0.920390 | -4.910072 | -1.288640 | 0.909316 | -4.906608 | -1.245481 | 0.930682 | -4.874908 | -1.362707 | 0.935106 | -4.847834 | -1.499506 | 0.922411 | -4.871645 | -1.375873 | 0.901813 | -4.893749 | -1.282149 |
| H | 3.353880 | -4.864912 | -0.896760 | 3.346918 | -4.863409 | -0.875145 | 3.363131 | -4.835064 | -0.956603 | 3.358707 | -4.824209 | -1.046546 | 3.354205 | -4.837793 | -0.964395 | 3.338368 | -4.858318 | -0.901371 |
| H | 4.487629 | -2.736763 | -0.316562 | 4.487248 | -2.738032 | -0.297269 | 4.494098 | -2.721995 | -0.320215 | 4.487139 | -2.727110 | -0.355543 | 4.488885 | -2.727525 | -0.324185 | 4.482124 | -2.739774 | -0.305319 |
| H | 0.806180 | 2.944223 | -0.931855 | 0.813050 | 2.935626 | -0.882335 | 0.805101 | 2.911729 | -0.971326 | 0.798318 | 2.897399 | -1.097103 | 0.805544 | 2.915891 | -0.978413 | 0.812675 | 2.933334 | -0.909778 |
| H | -4.128963 | 2.820367 | -0.423185 | -4.127709 | 2.825041 | -0.417488 | -4.134077 | 2.810213 | -0.448241 | -4.129938 | 2.810516 | -0.458649 | -4.133047 | 2.814539 | -0.446991 | -4.127087 | 2.828994 | -0.413493 |
| H | -0.514615 | 5.002773 | -1.157363 | -0.502337 | 4.992937 | -1.143809 | -0.515839 | 4.964887 | -1.258315 | -0.526228 | 4.949450 | -1.374256 | -0.515950 | 4.969681 | -1.263061 | -0.503687 | 4.990253 | -1.176032 |
| H | -2.964808 | 4.955958 | -0.883096 | -2.955550 | 4.952236 | -0.889203 | -2.966083 | 4.927213 | -0.971557 | -2.968606 | 4.919520 | -1.031903 | -2.965961 | 4.931873 | -0.972917 | -2.955586 | 4.952443 | -0.904910 |
| H | 4.497701 | -1.276904 | 1.953763 | 4.494149 | -1.094252 | 2.074933 | 4.451310 | -1.052735 | 2.118714 | 4.426134 | -1.159513 | 2.076246 | 4.441328 | -1.057642 | 2.117922 | 4.479908 | -1.076789 | 2.091414 |
| H | 4.620988 | 1.400265 | -1.371431 | 4.650886 | 1.202374 | -1.524708 | 4.681507 | 1.160566 | -1.532990 | 4.670233 | 1.265404 | -1.439318 | 4.688609 | 1.160816 | -1.529726 | 4.662016 | 1.176736 | -1.536512 |
| H | 6.844686 | -0.669423 | 2.529013 | 6.861891 | -0.484874 | 2.565683 | 6.814770 | -0.449951 | 2.632664 | 6.772390 | -0.555285 | 2.655379 | 6.802753 | -0.456377 | 2.643380 | 6.848021 | -0.472282 | 2.584706 |
| H | 6.957541 | 2.027012 | -0.775845 | 7.008187 | 1.834878 | -1.016813 | 7.035072 | 1.786997 | -1.004247 | 7.006815 | 1.892836 | -0.842593 | 7.040322 | 1.785486 | -0.989571 | 7.019894 | 1.804376 | -1.027067 |
| H | -4.266748 | 1.450860 | 1.704416 | -4.299499 | 1.405255 | 1.748498 | -4.270519 | 1.396755 | 1.763699 | -4.228937 | 1.403525 | 1.780481 | -4.269888 | 1.399929 | 1.765363 | -4.296208 | 1.392142 | 1.769111 |
| H | -4.168573 | -1.479387 | -1.407275 | -4.161866 | -1.416193 | -1.464600 | -4.184801 | -1.406443 | -1.472062 | -4.188985 | -1.413423 | -1.444939 | -4.179864 | -1.406511 | -1.467365 | -4.159117 | -1.392037 | -1.478446 |
| H | -6.599783 | 0.828490 | 2.223974 | -6.645730 | 0.782124 | 2.202443 | -6.612921 | 0.778504 | 2.244467 | -6.562699 | 0.780569 | 2.302611 | -6.610523 | 0.776047 | 2.249252 | -6.642028 | 0.760704 | 2.219000 |
| H | -6.492814 | -2.124102 | -0.874744 | -6.497823 | -2.066426 | -0.997806 | -6.516259 | -2.054234 | -0.978209 | -6.511856 | -2.066225 | -0.908676 | -6.509906 | -2.059775 | -0.970761 | -6.494833 | -2.052120 | -1.016083 |

| **iso-I** 4 |  | gasphase |  |  | toluene |  |  | DMSO |  |  | methanol |  |  | acetonitrile |  |  | chloroform |  |
| --- | --- | --- | --- | --- | --- | --- | --- | --- | --- | --- | --- | --- | --- | --- | --- | --- | --- | --- |
| Element | x | y | z | x | y | z | x | y | z | x | y | z | x | y | z | x | y | z |
| C | 0.685444 | -2.867406 | -0.595971 | 0.714903 | -2.822406 | -0.727001 | 0.725003 | -2.811519 | -0.761170 | 0.722523 | -2.781564 | -0.937211 | -0.721065 | 2.811950 | -0.766296 | 0.712451 | -2.817063 | -0.749306 |
| C | 1.418707 | -4.051915 | -0.683755 | 1.455013 | -3.989207 | -0.864205 | 1.469795 | -3.973596 | -0.915819 | 1.464035 | -3.945108 | -1.093323 | -1.464138 | 3.975087 | -0.922411 | 1.452333 | -3.983766 | -0.891284 |
| C | 2.805605 | -4.047323 | -0.552220 | 2.831726 | -3.981940 | -0.699720 | 2.847055 | -3.963962 | -0.742203 | 2.833239 | -3.951339 | -0.863121 | -2.841512 | 3.967696 | -0.748634 | 2.828673 | -3.978987 | -0.717363 |
| C | 3.494920 | -2.852780 | -0.336627 | 3.505489 | -2.801859 | -0.412386 | 3.516582 | -2.786519 | -0.432836 | 3.500797 | -2.788408 | -0.497488 | -3.512807 | 2.791503 | -0.437500 | 3.502515 | -2.801541 | -0.417875 |
| C | 2.752964 | -1.683804 | -0.246594 | 2.755812 | -1.651675 | -0.281563 | 2.761342 | -1.641515 | -0.286968 | 2.748390 | -1.643116 | -0.348183 | -2.759284 | 1.645571 | -0.289730 | 2.752630 | -1.651978 | -0.283086 |
| C | 1.345263 | -1.655152 | -0.364035 | 1.358254 | -1.630384 | -0.411742 | 1.364327 | -1.625044 | -0.417971 | 1.358502 | -1.614472 | -0.529436 | -1.362239 | 1.626891 | -0.421085 | 1.355867 | -1.629029 | -0.418839 |
| N | 3.248148 | -0.387793 | -0.086049 | 3.234800 | -0.361292 | -0.060283 | 3.232981 | -0.352547 | -0.045786 | 3.216041 | -0.356938 | -0.065397 | -3.232600 | 0.356939 | -0.046994 | 3.230759 | -0.362727 | -0.051757 |
| C | 2.222829 | 0.541349 | -0.147590 | 2.207239 | 0.555032 | -0.084351 | 2.202875 | 0.556149 | -0.045322 | 2.193575 | 0.552292 | -0.096303 | -2.203749 | -0.552564 | -0.047479 | 2.205115 | 0.552044 | -0.070409 |
| C | 0.925715 | -0.239320 | -0.291657 | 0.926361 | -0.230834 | -0.290213 | 0.927455 | -0.231031 | -0.277002 | 0.925940 | -0.223419 | -0.376009 | -0.927390 | 0.232150 | -0.279199 | 0.925274 | -0.230441 | -0.290186 |
| C | -0.305186 | 0.358218 | -0.288574 | -0.303645 | 0.341262 | -0.285152 | -0.304928 | 0.332185 | -0.275370 | -0.304701 | 0.340737 | -0.376609 | 0.303932 | -0.333512 | -0.276264 | -0.303934 | 0.341812 | -0.287367 |
| O | 2.402994 | 1.732553 | -0.053683 | 2.359393 | 1.738585 | 0.104926 | 2.341303 | 1.734951 | 0.191561 | 2.325370 | 1.732258 | 0.162664 | -2.343695 | -1.731684 | 0.188864 | 2.354049 | 1.734632 | 0.137290 |
| C | -0.725352 | 1.772944 | -0.362733 | -0.737331 | 1.739737 | -0.405142 | -0.740953 | 1.726528 | -0.413154 | -0.736093 | 1.733162 | -0.518730 | 0.738724 | -1.728468 | -0.413650 | -0.735289 | 1.739901 | -0.413441 |
| C | -2.132806 | 1.798983 | -0.238621 | -2.134577 | 1.757050 | -0.268321 | -2.137670 | 1.741773 | -0.277780 | -2.127194 | 1.758340 | -0.345463 | 2.135466 | -1.744929 | -0.277504 | -2.131981 | 1.759830 | -0.274200 |
| N | -2.627020 | 0.504892 | -0.072004 | -2.611549 | 0.468076 | -0.043136 | -2.610043 | 0.454168 | -0.036717 | -2.596938 | 0.470462 | -0.078294 | 2.608661 | -0.457331 | -0.035262 | -2.609355 | 0.471625 | -0.042146 |
| C | -1.604023 | -0.423084 | -0.133222 | -1.585107 | -0.445833 | -0.067294 | -1.582182 | -0.454630 | -0.041502 | -1.575524 | -0.437355 | -0.111245 | 1.581526 | 0.451446 | -0.040737 | -1.584937 | -0.441436 | -0.062537 |
| C | -0.068536 | 2.986391 | -0.598405 | -0.098394 | 2.934335 | -0.720522 | -0.103184 | 2.914260 | -0.755183 | -0.098770 | 2.905353 | -0.909522 | 0.099986 | -2.915670 | -0.756333 | -0.094533 | 2.930034 | -0.742047 |
| C | -0.804411 | 4.169439 | -0.682514 | -0.842459 | 4.099395 | -0.851126 | -0.849113 | 4.076494 | -0.903749 | -0.840593 | 4.070049 | -1.056249 | 0.845140 | -4.078580 | -0.904976 | -0.836948 | 4.095812 | -0.878584 |
| C | -2.190766 | 4.162129 | -0.542187 | -2.218453 | 4.087999 | -0.678615 | -2.225805 | 4.065855 | -0.724373 | -2.211029 | 4.072609 | -0.832675 | 2.221923 | -4.069030 | -0.725256 | -2.212917 | 4.088054 | -0.700022 |
| C | -2.877260 | 2.966769 | -0.322811 | -2.887946 | 2.905673 | -0.390797 | -2.893760 | 2.887209 | -0.416236 | -2.879623 | 2.904946 | -0.484247 | 2.890756 | -2.890822 | -0.416412 | -2.884099 | 2.908719 | -0.402152 |
| O | -1.779637 | -1.614600 | -0.034191 | -1.731217 | -1.629367 | 0.127371 | -1.719013 | -1.634954 | 0.190140 | -1.707328 | -1.620696 | 0.134374 | 1.718734 | 1.631993 | 0.191710 | -1.729898 | -1.624805 | 0.144795 |
| C | 4.603746 | -0.042196 | 0.143090 | 4.590228 | -0.020122 | 0.182723 | 4.590099 | -0.011229 | 0.194705 | 4.561781 | -0.021694 | 0.241265 | -4.589436 | 0.014889 | 0.195149 | 4.587224 | -0.022221 | 0.191458 |
| C | 5.255589 | 0.852155 | -0.708645 | 5.274545 | 0.783707 | -0.717205 | 5.288376 | 0.737377 | -0.741527 | 5.275069 | 0.809320 | -0.610394 | -5.283811 | -0.746008 | -0.734292 | 5.278486 | 0.756369 | -0.725315 |
| C | 6.582124 | 1.199522 | -0.474223 | 6.595742 | 1.122239 | -0.479716 | 6.611627 | 1.074778 | -0.510107 | 6.585507 | 1.143833 | -0.311962 | -6.606244 | -1.085919 | -0.501107 | 6.600286 | 1.095167 | -0.489487 |
| C | 7.248047 | 0.633254 | 0.606329 | 7.208544 | 0.632130 | 0.655909 | 7.205911 | 0.637064 | 0.656228 | 7.152337 | 0.620947 | 0.832740 | -7.203727 | -0.638483 | 0.660085 | 7.203327 | 0.629825 | 0.661684 |
| C | 6.618150 | -0.260030 | 1.464657 | 6.550524 | -0.173416 | 1.563577 | 6.535066 | -0.115363 | 1.599103 | 6.465037 | -0.212809 | 1.691710 | -6.536568 | 0.125664 | 1.596363 | 6.538636 | -0.151032 | 1.585922 |
| C | 5.285247 | -0.586818 | 1.234573 | 5.224927 | -0.492663 | 1.323010 | 5.208420 | -0.435286 | 1.362468 | 5.150830 | -0.529000 | 1.390810 | -5.210570 | 0.447856 | 1.358304 | 5.212859 | -0.471306 | 1.346049 |
| F | 8.521621 | 0.956023 | 0.826219 | 8.491844 | 0.950823 | 0.886564 | 8.493108 | 0.956194 | 0.883010 | 8.427262 | 0.936361 | 1.122897 | -8.490085 | -0.959843 | 0.888431 | 8.488092 | 0.949508 | 0.891871 |
| C | -3.987070 | 0.164340 | 0.150126 | -3.971094 | 0.131159 | 0.193574 | -3.970254 | 0.119057 | 0.203627 | -3.947747 | 0.135604 | 0.212197 | 3.968872 | -0.120948 | 0.205712 | -3.968949 | 0.134470 | 0.197349 |
| C | -4.658508 | -0.677054 | -0.730860 | -4.686658 | -0.583728 | -0.748043 | -4.705064 | -0.542692 | -0.762738 | -4.678936 | -0.632061 | -0.675772 | 4.705211 | 0.534629 | -0.764009 | -4.686831 | -0.574359 | -0.747707 |
| C | -5.990707 | -1.023163 | -0.506833 | -6.014725 | -0.916332 | -0.521950 | -6.034417 | -0.871687 | -0.535977 | -5.996574 | -0.967316 | -0.397168 | 6.034237 | 0.865248 | -0.537224 | -6.013971 | -0.910106 | -0.519535 |
| C | -6.667105 | -0.508712 | 0.604858 | -6.633951 | -0.514643 | 0.657591 | -6.634022 | -0.519551 | 0.670820 | -6.585372 | -0.513714 | 0.779074 | 6.631422 | 0.521243 | 0.673027 | -6.628543 | -0.517644 | 0.665954 |
| C | -5.989413 | 0.342367 | 1.490773 | -5.910807 | 0.212179 | 1.604278 | -5.890239 | 0.155056 | 1.641591 | -5.847002 | 0.266011 | 1.670306 | 5.886229 | -0.146323 | 1.647592 | -5.902912 | 0.203385 | 1.615749 |
| C | -4.660174 | 0.664930 | 1.270847 | -4.588218 | 0.525303 | 1.375660 | -4.566999 | 0.466959 | 1.410260 | -4.534287 | 0.582650 | 1.390807 | 4.563253 | -0.460034 | 1.416173 | -4.581179 | 0.520305 | 1.384704 |
| O | -7.953975 | -0.773894 | 0.903778 | -7.918665 | -0.779014 | 0.971909 | -7.916325 | -0.785076 | 0.987762 | -7.861319 | -0.776702 | 1.141079 | 7.913585 | 0.788697 | 0.990697 | -7.911894 | -0.786350 | 0.983289 |
| C | -8.688151 | -1.625781 | 0.066790 | -8.695093 | -1.523509 | 0.057816 | -8.715384 | -1.470104 | 0.038887 | -8.662047 | -1.562444 | 0.272512 | 8.717430 | 1.464676 | 0.039200 | -8.695197 | -1.521003 | 0.062651 |
| H | -0.395016 | -2.883143 | -0.689578 | -0.354063 | -2.844652 | -0.847688 | -0.343017 | -2.834023 | -0.895377 | -0.337356 | -2.788527 | -1.131335 | 0.346954 | 2.832422 | -0.901362 | -0.355601 | -2.837018 | -0.881442 |
| H | 0.891469 | -4.991669 | -0.857185 | 0.944881 | -4.914260 | -1.099122 | 0.965676 | -4.896258 | -1.173386 | 0.965262 | -4.855556 | -1.400025 | -0.958622 | 4.896574 | -1.181354 | 0.943307 | -4.906501 | -1.138074 |
| H | 3.365177 | -4.982480 | -0.625115 | 3.393648 | -4.901616 | -0.804912 | 3.412493 | -4.880121 | -0.859725 | 3.395950 | -4.868732 | -0.984421 | -3.405651 | 4.884468 | -0.867492 | 3.390196 | -4.898630 | -0.825703 |
| H | 4.582123 | -2.833468 | -0.253778 | 4.581675 | -2.781823 | -0.307037 | 4.592414 | -2.765834 | -0.320887 | 4.572304 | -2.780736 | -0.348705 | -4.588627 | 2.772501 | -0.325618 | 4.578197 | -2.783786 | -0.305588 |
| H | 1.011241 | 3.004963 | -0.698250 | 0.969846 | 2.960340 | -0.846519 | 0.964292 | 2.938336 | -0.893469 | 0.962103 | 2.915819 | -1.098004 | -0.967501 | -2.938633 | -0.895326 | 0.973097 | 2.952805 | -0.877091 |
| H | -3.963662 | 2.943518 | -0.230420 | -3.963203 | 2.880366 | -0.276353 | -3.969047 | 2.864023 | -0.298775 | -3.951761 | 2.893433 | -0.339663 | 3.965985 | -2.868198 | -0.298574 | -3.959208 | 2.886988 | -0.284313 |
| H | -0.280039 | 5.110210 | -0.859069 | -0.336144 | 5.026526 | -1.086138 | -0.346362 | 5.000144 | -1.160468 | -0.340999 | 4.984393 | -1.349773 | 0.341715 | -5.001733 | -1.162083 | -0.330355 | 5.020274 | -1.123952 |
| H | -2.751857 | 5.096769 | -0.610638 | -2.782799 | 5.006989 | -0.777123 | -2.791660 | 4.982513 | -0.836191 | -2.773523 | 4.991240 | -0.945551 | 2.787110 | -4.986058 | -0.837290 | -2.775919 | 5.007466 | -0.802927 |
| H | 4.715809 | 1.284683 | -1.551225 | 4.770010 | 1.148372 | -1.601731 | 4.796731 | 1.055781 | -1.651488 | 4.805631 | 1.194845 | -1.506116 | -4.789266 | -1.072367 | -1.639876 | 4.780025 | 1.100084 | -1.622047 |
| H | 4.765054 | -1.270617 | 1.908052 | 4.678321 | -1.110954 | 2.023462 | 4.651336 | -1.017201 | 2.085631 | 4.582021 | -1.172506 | 2.049814 | -4.656370 | 1.038442 | 2.076603 | 4.660522 | -1.071764 | 2.057530 |
| H | 7.109467 | 1.900349 | -1.122724 | 7.148210 | 1.752340 | -1.163515 | 7.176577 | 1.660398 | -1.223124 | 7.161687 | 1.792543 | -0.958379 | -7.168127 | -1.680972 | -1.208700 | 7.158894 | 1.705428 | -1.186425 |
| H | 7.170045 | -0.677220 | 2.307949 | 7.066673 | -0.530710 | 2.444388 | 7.040158 | -0.436010 | 2.500537 | 6.947392 | -0.598344 | 2.580155 | -7.043863 | 0.453150 | 2.494082 | 7.048775 | -0.489973 | 2.477618 |
| H | -4.132271 | -1.077828 | -1.598056 | -4.203101 | -0.890803 | -1.666417 | -4.238101 | -0.806876 | -1.703413 | -4.219321 | -0.975334 | -1.594243 | 4.239448 | 0.792752 | -1.706985 | -4.206821 | -0.873186 | -1.670979 |
| H | -4.130113 | 1.312650 | 1.972277 | -4.023708 | 1.079093 | 2.115432 | -3.988931 | 0.985250 | 2.165434 | -3.958363 | 1.181045 | 2.085956 | 3.983736 | -0.972929 | 2.173870 | -4.015031 | 1.070570 | 2.125996 |
| H | -6.487590 | -1.691030 | -1.210076 | -6.549965 | -1.481661 | -1.271043 | -6.586068 | -1.392901 | -1.305156 | -6.547113 | -1.571889 | -1.103570 | 6.587784 | 1.381420 | -1.308401 | -6.552061 | -1.469885 | -1.270875 |
| H | -6.529520 | 0.727396 | 2.356913 | -6.403205 | 0.513260 | 2.519662 | -6.365492 | 0.423026 | 2.576652 | -6.316972 | 0.610249 | 2.583011 | 6.360613 | -0.407062 | 2.585160 | -6.391908 | 0.498527 | 2.535217 |
| H | -9.693178 | -1.701130 | 0.498974 | -9.680273 | -1.623119 | 0.508936 | -9.697163 | -1.579422 | 0.494838 | -9.633423 | -1.645499 | 0.755314 | 9.697822 | 1.574081 | 0.497865 | -9.678224 | -1.622368 | 0.517826 |
| H | -8.770071 | -1.222612 | -0.957716 | -8.792232 | -1.005705 | -0.901274 | -8.810677 | -0.898574 | -0.888425 | -8.781135 | -1.078610 | -0.700400 | 8.813078 | 0.885693 | -0.883351 | -8.793129 | -0.991562 | -0.889468 |
| H | -8.243904 | -2.635232 | 0.019037 | -8.275087 | -2.520413 | -0.106250 | -8.309164 | -2.460805 | -0.182699 | -8.237674 | -2.560846 | 0.138896 | 8.313729 | 2.454480 | -0.190476 | -8.276214 | -2.516021 | -0.112479 |

| **iso-I** 5 |  | gasphase |  |  | toluene |  |  | DMSO |  |  | methanol |  |  | acetonitrile |  |  | chloroform |  |
| --- | --- | --- | --- | --- | --- | --- | --- | --- | --- | --- | --- | --- | --- | --- | --- | --- | --- | --- |
| Element | x | y | z | x | y | z | x | y | z | x | y | z | x | y | z | x | y | z |
| C | -0.903393 | 2.896953 | 0.170583 | -0.922951 | 2.892377 | 0.196441 | -0.919769 | 2.888115 | 0.239981 | -0.830368 | 2.839292 | 0.342764 | -0.899307 | 2.882251 | 0.241818 | -0.910618 | 2.888143 | 0.238396 |
| C | -1.655492 | 4.049973 | 0.351375 | -1.678536 | 4.039971 | 0.396147 | -1.677852 | 4.034358 | 0.441535 | -1.559619 | 4.002351 | 0.552267 | -1.650997 | 4.033584 | 0.439348 | -1.664226 | 4.038048 | 0.434361 |
| C | -3.038577 | 3.994329 | 0.417211 | -3.062596 | 3.979087 | 0.454367 | -3.063996 | 3.972108 | 0.481063 | -2.947638 | 3.978492 | 0.562428 | -3.037658 | 3.979798 | 0.475553 | -3.049843 | 3.981792 | 0.475152 |
| C | -3.704982 | 2.779709 | 0.319454 | -3.725594 | 2.764554 | 0.333333 | -3.726027 | 2.758340 | 0.342722 | -3.642449 | 2.787171 | 0.389185 | -3.706725 | 2.769617 | 0.337667 | -3.716462 | 2.769997 | 0.342476 |
| C | -2.944544 | 1.643769 | 0.137939 | -2.960383 | 1.634176 | 0.136566 | -2.957365 | 1.630064 | 0.146279 | -2.902230 | 1.643071 | 0.182415 | -2.944551 | 1.636331 | 0.144753 | -2.952878 | 1.637802 | 0.150067 |
| C | -1.544448 | 1.669787 | 0.041098 | -1.560436 | 1.666296 | 0.041598 | -1.556972 | 1.664591 | 0.063835 | -1.501296 | 1.641559 | 0.123232 | -1.543587 | 1.662159 | 0.065914 | -1.552146 | 1.666085 | 0.068427 |
| N | -3.403307 | 0.327116 | 0.085343 | -3.414505 | 0.318012 | 0.057174 | -3.408337 | 0.314653 | 0.051350 | -3.383457 | 0.336401 | 0.062249 | -3.403244 | 0.322939 | 0.050913 | -3.409171 | 0.322308 | 0.064134 |
| C | -2.346042 | -0.557750 | 0.016519 | -2.358556 | -0.560731 | -0.039555 | -2.354476 | -0.560235 | -0.054308 | -2.351011 | -0.556744 | -0.032991 | -2.354181 | -0.557675 | -0.049041 | -2.355997 | -0.557084 | -0.025099 |
| C | -1.083135 | 0.277990 | -0.057848 | -1.095867 | 0.278061 | -0.079439 | -1.091314 | 0.279333 | -0.064198 | -1.071371 | 0.248482 | -0.014962 | -1.086274 | 0.273552 | -0.058973 | -1.091443 | 0.277591 | -0.052886 |
| C | 0.149081 | -0.257814 | -0.242909 | 0.141019 | -0.250311 | -0.250932 | 0.146959 | -0.245346 | -0.227245 | 0.152633 | -0.305472 | -0.179252 | 0.148212 | -0.260523 | -0.221205 | 0.143487 | -0.253682 | -0.224555 |
| O | -2.479673 | -1.755756 | -0.037767 | -2.487793 | -1.757168 | -0.146946 | -2.479057 | -1.754939 | -0.201781 | -2.497326 | -1.750891 | -0.204429 | -2.484858 | -1.752687 | -0.191546 | -2.485176 | -1.754337 | -0.141043 |
| C | 0.654257 | -1.634584 | -0.133024 | 0.650687 | -1.623041 | -0.122524 | 0.650875 | -1.616446 | -0.084215 | 0.624676 | -1.682648 | -0.015040 | 0.643038 | -1.635274 | -0.079001 | 0.647273 | -1.626933 | -0.090554 |
| C | 2.007040 | -1.614232 | -0.510163 | 2.011351 | -1.595614 | -0.468521 | 2.004010 | -1.603256 | -0.457185 | 1.968252 | -1.712629 | -0.416026 | 1.995206 | -1.631506 | -0.456042 | 2.001956 | -1.610023 | -0.458235 |
| N | 2.374198 | -0.332180 | -0.894121 | 2.379859 | -0.312927 | -0.849512 | 2.373166 | -0.326982 | -0.861281 | 2.354746 | -0.452217 | -0.866831 | 2.371735 | -0.357560 | -0.862496 | 2.371191 | -0.331669 | -0.856555 |
| C | 1.343832 | 0.554695 | -0.709682 | 1.337489 | 0.564226 | -0.708844 | 1.343005 | 0.558866 | -0.702662 | 1.354178 | 0.458631 | -0.691251 | 1.349121 | 0.535267 | -0.696819 | 1.338593 | 0.552071 | -0.698703 |
| C | 0.127712 | -2.832985 | 0.334471 | 0.122455 | -2.819778 | 0.347312 | 0.125092 | -2.794353 | 0.433958 | 0.081627 | -2.825326 | 0.560144 | 0.110893 | -2.809382 | 0.441810 | 0.120776 | -2.812922 | 0.408102 |
| C | 0.932222 | -3.964557 | 0.385144 | 0.933063 | -3.945274 | 0.426993 | 0.929342 | -3.923778 | 0.523521 | 0.860250 | -3.970348 | 0.669604 | 0.908083 | -3.944116 | 0.529942 | 0.926859 | -3.941696 | 0.489591 |
| C | 2.257195 | -3.916463 | -0.016457 | 2.266623 | -3.891379 | 0.052575 | 2.254460 | -3.888477 | 0.111742 | 2.176072 | -3.980704 | 0.227051 | 2.232444 | -3.917808 | 0.114623 | 2.254300 | -3.899835 | 0.089463 |
| C | 2.817711 | -2.727446 | -0.465876 | 2.829265 | -2.702686 | -0.395495 | 2.816400 | -2.715472 | -0.378041 | 2.756496 | -2.839012 | -0.313811 | 2.800666 | -2.748705 | -0.377781 | 2.815842 | -2.720293 | -0.384481 |
| O | 1.430444 | 1.733347 | -0.963230 | 1.409061 | 1.738318 | -0.993287 | 1.415600 | 1.734796 | -0.987237 | 1.445760 | 1.632283 | -0.996404 | 1.430485 | 1.712380 | -0.975323 | 1.411217 | 1.727613 | -0.982846 |
| C | -4.762579 | -0.070374 | 0.070514 | -4.775635 | -0.080662 | 0.049575 | -4.771166 | -0.082473 | 0.033378 | -4.753667 | -0.032506 | 0.007101 | -4.767941 | -0.068561 | 0.031737 | -4.771447 | -0.074464 | 0.040199 |
| C | -5.242801 | -0.949270 | 1.030866 | -5.269786 | -0.879270 | 1.070618 | -5.290037 | -0.814556 | 1.091378 | -5.291025 | -0.833988 | 1.003850 | -5.288528 | -0.805558 | 1.085735 | -5.280030 | -0.863248 | 1.061876 |
| C | -6.569056 | -1.344673 | 1.009647 | -6.596655 | -1.274188 | 1.062248 | -6.618255 | -1.207025 | 1.075770 | -6.624850 | -1.203027 | 0.949626 | -6.618199 | -1.193491 | 1.068736 | -6.607170 | -1.257994 | 1.037394 |
| C | -7.398446 | -0.838752 | 0.028398 | -7.406689 | -0.844504 | 0.030482 | -7.398358 | -0.839695 | -0.001992 | -7.391295 | -0.742967 | -0.101988 | -7.397995 | -0.816985 | -0.006283 | -7.399963 | -0.837086 | -0.011207 |
| C | -6.943648 | 0.038448 | -0.936184 | -6.939821 | -0.045408 | -0.993764 | -6.907708 | -0.105179 | -1.062585 | -6.880937 | 0.061131 | -1.101173 | -6.905424 | -0.078029 | -1.063090 | -6.918858 | -0.047389 | -1.036154 |
| C | -5.611365 | 0.413906 | -0.915673 | -5.607207 | 0.330125 | -0.983038 | -5.574523 | 0.269762 | -1.041960 | -5.542400 | 0.411580 | -1.044743 | -5.570780 | 0.292163 | -1.041340 | -5.586279 | 0.328365 | -1.008759 |
| F | -8.684231 | -1.211854 | 0.010031 | -8.696144 | -1.217589 | 0.022420 | -8.691141 | -1.211989 | -0.018781 | -8.689250 | -1.091962 | -0.155124 | -8.692087 | -1.184631 | -0.024260 | -8.690730 | -1.210599 | -0.035470 |
| C | 3.682302 | 0.075123 | -1.292555 | 3.686816 | 0.081215 | -1.269508 | 3.683246 | 0.050701 | -1.293563 | 3.665217 | -0.103308 | -1.324323 | 3.683504 | 0.014026 | -1.295942 | 3.679576 | 0.050908 | -1.286894 |
| C | 4.640614 | 0.200557 | -0.113806 | 4.657210 | 0.230535 | -0.104347 | 4.650696 | 0.226294 | -0.130848 | 4.625962 | 0.166213 | -0.178219 | 4.648018 | 0.202171 | -0.133236 | 4.650467 | 0.216930 | -0.125734 |
| O | 5.875336 | 0.371311 | -0.572569 | 5.890886 | 0.335469 | -0.576315 | 5.882114 | 0.328016 | -0.598465 | 5.841508 | 0.341526 | -0.650689 | 5.875738 | 0.336492 | -0.600838 | 5.881224 | 0.326582 | -0.597152 |
| O | 4.298540 | 0.160242 | 1.033923 | 4.319971 | 0.262300 | 1.047035 | 4.307568 | 0.278920 | 1.021288 | 4.286543 | 0.215814 | 0.979335 | 4.306073 | 0.234723 | 1.020310 | 4.312998 | 0.255429 | 1.027357 |
| C | 7.015797 | 0.568365 | 0.323800 | 7.051877 | 0.540440 | 0.297236 | 7.047420 | 0.542446 | 0.272316 | 7.005346 | 0.644808 | 0.203939 | 7.038073 | 0.571956 | 0.268832 | 7.045253 | 0.551686 | 0.271071 |
| C | 7.206552 | -0.664303 | 1.197534 | 7.212399 | -0.651657 | 1.229240 | 7.206185 | -0.635917 | 1.220397 | 7.254265 | -0.512312 | 1.157705 | 7.225490 | -0.608163 | 1.209848 | 7.218104 | -0.626916 | 1.217334 |
| C | 6.818393 | 1.834154 | 1.146918 | 6.908219 | 1.852666 | 1.053790 | 6.909173 | 1.866348 | 1.007284 | 6.786106 | 1.965698 | 0.923174 | 6.870247 | 1.888710 | 1.010939 | 6.892347 | 1.873349 | 1.008282 |
| C | 8.181067 | 0.730223 | -0.640209 | 8.207698 | 0.612305 | -0.687505 | 8.202673 | 0.595305 | -0.712914 | 8.134432 | 0.762409 | -0.805358 | 8.188581 | 0.656549 | -0.720156 | 8.195836 | 0.618518 | -0.719740 |
| H | 0.169454 | 2.954304 | 0.116303 | 0.150349 | 2.954421 | 0.148831 | 0.154751 | 2.949739 | 0.210192 | 0.246676 | 2.869418 | 0.351045 | 0.175707 | 2.937085 | 0.215548 | 0.163955 | 2.945883 | 0.208507 |
| H | -1.149436 | 5.001918 | 0.441638 | -1.175630 | 4.991794 | 0.506489 | -1.177581 | 4.985820 | 0.568803 | -1.035931 | 4.936151 | 0.712136 | -1.145212 | 4.982144 | 0.566318 | -1.159486 | 4.987645 | 0.556350 |
| H | -3.610338 | 4.902944 | 0.557792 | -3.637303 | 4.884064 | 0.607365 | -3.640796 | 4.875937 | 0.633817 | -3.501279 | 4.895332 | 0.723423 | -3.609389 | 4.887311 | 0.625385 | -3.622927 | 4.888448 | 0.624765 |
| H | -4.781700 | 2.722759 | 0.399970 | -4.803054 | 2.703925 | 0.405214 | -4.804741 | 2.698310 | 0.398717 | -4.723138 | 2.757873 | 0.429485 | -4.785856 | 2.716192 | 0.391264 | -4.795051 | 2.713799 | 0.401923 |
| H | -0.902255 | -2.888831 | 0.639447 | -0.913205 | -2.880161 | 0.633154 | -0.901491 | -2.837770 | 0.755996 | -0.933757 | -2.828783 | 0.921178 | -0.914912 | -2.845456 | 0.767535 | -0.908672 | -2.862734 | 0.718983 |
| H | 3.859928 | -2.676553 | -0.751859 | 3.877193 | -2.646003 | -0.659679 | 3.857493 | -2.674878 | -0.671088 | 3.793266 | -2.832588 | -0.624005 | 3.841409 | -2.713678 | -0.672548 | 3.859150 | -2.672134 | -0.668057 |
| H | 3.600804 | 1.055803 | -1.763727 | 3.607939 | 1.050915 | -1.763304 | 3.613736 | 1.003880 | -1.819021 | 3.601019 | 0.801263 | -1.930241 | 3.618020 | 0.959829 | -1.834898 | 3.607959 | 1.010273 | -1.801154 |
| H | 4.097052 | -0.614928 | -2.028803 | 4.087863 | -0.629398 | -1.993180 | 4.078314 | -0.686660 | -1.992292 | 4.069519 | -0.893435 | -1.956735 | 4.080939 | -0.732974 | -1.983009 | 4.076010 | -0.677102 | -1.995182 |
| H | 0.511998 | -4.894914 | 0.743277 | 0.511991 | -4.875307 | 0.785877 | 0.513490 | -4.840865 | 0.920715 | 0.433750 | -4.862337 | 1.110289 | 0.487366 | -4.858035 | 0.929264 | 0.508586 | -4.864097 | 0.871171 |
| H | 2.868632 | -4.808781 | 0.028904 | 2.882920 | -4.779261 | 0.119033 | 2.865900 | -4.779465 | 0.183615 | 2.768043 | -4.883034 | 0.318183 | 2.838346 | -4.812585 | 0.186065 | 2.867116 | -4.790131 | 0.157082 |
| H | -4.572809 | -1.332023 | 1.787806 | -4.612941 | -1.195672 | 1.869544 | -4.655175 | -1.076544 | 1.927595 | -4.666462 | -1.169169 | 1.821730 | -4.653575 | -1.075356 | 1.919413 | -4.635819 | -1.171291 | 1.874589 |
| H | -5.225220 | 1.086431 | -1.670595 | -5.209086 | 0.944286 | -1.780482 | -5.156613 | 0.838898 | -1.862521 | -5.109747 | 1.032254 | -1.819039 | -5.151441 | 0.864245 | -1.859081 | -5.176472 | 0.936377 | -1.805090 |
| H | -6.962974 | -2.033302 | 1.744330 | -7.003167 | -1.901267 | 1.844136 | -7.045563 | -1.781070 | 1.887173 | -7.066547 | -1.830174 | 1.712712 | -7.046853 | -1.771117 | 1.876862 | -7.025328 | -1.877160 | 1.819675 |
| H | -7.622072 | 0.405927 | -1.693724 | -7.607018 | 0.263763 | -1.786922 | -7.554520 | 0.159307 | -1.888491 | -7.517038 | 0.397062 | -1.909382 | -7.551880 | 0.193151 | -1.887090 | -7.573670 | 0.255521 | -1.842164 |
| H | 8.134984 | -0.562204 | 1.761558 | 8.156123 | -0.558232 | 1.769353 | 8.155794 | -0.537341 | 1.749138 | 8.207047 | -0.350133 | 1.664603 | 8.174297 | -0.491558 | 1.736284 | 8.166706 | -0.520871 | 1.746457 |
| H | 7.282897 | -1.559761 | 0.578321 | 7.240404 | -1.581720 | 0.658147 | 7.222958 | -1.574614 | 0.663104 | 7.316629 | -1.452895 | 0.607126 | 7.260861 | -1.542683 | 0.646495 | 7.244504 | -1.564193 | 0.658022 |
| H | 6.384295 | -0.785562 | 1.898873 | 6.403606 | -0.706014 | 1.954808 | 6.404379 | -0.673423 | 1.955147 | 6.474933 | -0.594082 | 1.913209 | 6.426480 | -0.666845 | 1.946305 | 6.417031 | -0.674557 | 1.952109 |
| H | 7.733394 | 2.044049 | 1.703032 | 7.841268 | 2.065495 | 1.578432 | 7.844469 | 2.079803 | 1.527594 | 7.716798 | 2.255795 | 1.413776 | 7.801350 | 2.121251 | 1.530524 | 7.825159 | 2.099740 | 1.527615 |
| H | 6.615562 | 2.683845 | 0.493135 | 6.714493 | 2.673724 | 0.361097 | 6.720135 | 2.677453 | 0.301388 | 6.518113 | 2.747846 | 0.210414 | 6.660517 | 2.697963 | 0.308836 | 6.690969 | 2.682102 | 0.303300 |
| H | 5.998806 | 1.726230 | 1.853467 | 6.104135 | 1.810087 | 1.785271 | 6.105558 | 1.838626 | 1.740549 | 6.008235 | 1.893962 | 1.681100 | 6.068905 | 1.838063 | 1.745556 | 6.090290 | 1.835022 | 1.742612 |
| H | 8.022785 | 1.589128 | -1.293167 | 8.071426 | 1.441283 | -1.383603 | 8.069843 | 1.415034 | -1.420990 | 7.932677 | 1.563793 | -1.518271 | 8.033601 | 1.476662 | -1.423241 | 8.049881 | 1.437262 | -1.426154 |
| H | 8.294645 | -0.160628 | -1.258944 | 8.285908 | -0.312996 | -1.260373 | 8.279344 | -0.340365 | -1.269359 | 8.260998 | -0.172897 | -1.353425 | 8.284079 | -0.274471 | -1.281448 | 8.278379 | -0.314898 | -1.278992 |
| H | 9.104642 | 0.884480 | -0.081231 | 9.143843 | 0.764444 | -0.148809 | 9.136686 | 0.753513 | -0.172326 | 9.066405 | 0.987911 | -0.285728 | 9.120506 | 0.833461 | -0.181808 | 9.132206 | 0.785398 | -0.185851 |

| **iso-I** 6 |  | gasphase |  |  | toluene |  |  | DMSO |  |  | methanol |  |  | acetonitrile |  |  | chloroform |  |
| --- | --- | --- | --- | --- | --- | --- | --- | --- | --- | --- | --- | --- | --- | --- | --- | --- | --- | --- |
| Element | x | y | z | x | y | z | x | y | z | x | y | z | x | y | z | x | y | z |
| C | -0.261885 | -2.690341 | -0.455116 | -0.274220 | -2.673134 | -0.527570 | -0.286641 | -2.651136 | -0.605885 | -0.338806 | -2.653185 | -0.932151 | -0.299173 | -2.649238 | -0.691592 | -0.292541 | -2.663315 | -0.661995 |
| C | -0.928154 | -3.898987 | -0.599663 | -0.944585 | -3.877751 | -0.686297 | -0.962622 | -3.846935 | -0.806456 | -1.033680 | -3.842842 | -1.101302 | -0.980183 | -3.843744 | -0.884023 | -0.972023 | -3.860507 | -0.840618 |
| C | -2.308715 | -3.967429 | -0.493580 | -2.325553 | -3.943401 | -0.572560 | -2.346033 | -3.908560 | -0.700527 | -2.402994 | -3.904142 | -0.877513 | -2.361303 | -3.904992 | -0.749696 | -2.352446 | -3.921699 | -0.708868 |
| C | -3.059752 | -2.821193 | -0.271968 | -3.072562 | -2.798893 | -0.328134 | -3.089518 | -2.769120 | -0.420838 | -3.118917 | -2.769851 | -0.513636 | -3.098301 | -2.766023 | -0.450349 | -3.090707 | -2.779837 | -0.425351 |
| C | -2.384207 | -1.624753 | -0.150391 | -2.391575 | -1.607419 | -0.188518 | -2.401414 | -1.588040 | -0.235232 | -2.413883 | -1.595384 | -0.361954 | -2.405526 | -1.586359 | -0.273673 | -2.400220 | -1.596934 | -0.263697 |
| C | -0.984544 | -1.528016 | -0.206428 | -0.992426 | -1.514194 | -0.251876 | -1.002179 | -1.500818 | -0.290014 | -1.023666 | -1.513087 | -0.525571 | -1.007452 | -1.500235 | -0.354556 | -1.001865 | -1.509556 | -0.344297 |
| N | -2.944453 | -0.360919 | 0.031051 | -2.946120 | -0.343698 | 0.006114 | -2.949034 | -0.327590 | -0.006740 | -2.931865 | -0.332070 | -0.068138 | -2.947330 | -0.325456 | -0.032054 | -2.944387 | -0.334438 | -0.033751 |
| C | -1.964593 | 0.608669 | 0.072652 | -1.967651 | 0.623132 | 0.038436 | -1.970058 | 0.631487 | 0.053969 | -1.945660 | 0.614451 | -0.072491 | -1.966606 | 0.632262 | 0.010663 | -1.962900 | 0.624792 | 0.005126 |
| C | -0.636413 | -0.105386 | -0.080510 | -0.640605 | -0.094324 | -0.117020 | -0.646205 | -0.087824 | -0.121223 | -0.645627 | -0.110786 | -0.348676 | -0.646609 | -0.088763 | -0.183933 | -0.642309 | -0.095524 | -0.185286 |
| C | 0.560458 | 0.535079 | -0.064404 | 0.560336 | 0.536845 | -0.087840 | 0.558088 | 0.532523 | -0.082617 | 0.564706 | 0.497646 | -0.325588 | 0.559341 | 0.528656 | -0.144234 | 0.561478 | 0.528148 | -0.151958 |
| O | -2.189089 | 1.778569 | 0.265921 | -2.187349 | 1.795844 | 0.228931 | -2.180011 | 1.800961 | 0.285960 | -2.125315 | 1.785993 | 0.194580 | -2.171645 | 1.802485 | 0.244799 | -2.170803 | 1.795075 | 0.231281 |
| C | 0.934934 | 1.941776 | -0.230489 | 0.945008 | 1.940530 | -0.254559 | 0.949120 | 1.932326 | -0.255519 | 0.950257 | 1.899027 | -0.462641 | 0.952012 | 1.929587 | -0.300497 | 0.948134 | 1.931312 | -0.305885 |
| C | 2.333900 | 2.032990 | -0.158108 | 2.342355 | 2.024306 | -0.153793 | 2.346106 | 2.008156 | -0.152647 | 2.333300 | 1.992794 | -0.262638 | 2.347130 | 2.005825 | -0.176593 | 2.342878 | 2.014876 | -0.180923 |
| N | 2.886865 | 0.756496 | 0.061954 | 2.881865 | 0.747047 | 0.084672 | 2.875959 | 0.730990 | 0.101490 | 2.860092 | 0.712434 | 0.008280 | 2.874680 | 0.726074 | 0.073996 | 2.878004 | 0.735711 | 0.062469 |
| C | 1.861179 | -0.193012 | 0.185929 | 1.852536 | -0.196324 | 0.188308 | 1.843330 | -0.205518 | 0.205299 | 1.840082 | -0.243290 | -0.026412 | 1.841562 | -0.214065 | 0.143015 | 1.847891 | -0.210143 | 0.128204 |
| C | 0.213189 | 3.092717 | -0.536785 | 0.237227 | 3.091071 | -0.592032 | 0.248091 | 3.079012 | -0.618074 | 0.257532 | 3.028687 | -0.887317 | 0.254318 | 3.077678 | -0.665643 | 0.244414 | 3.078515 | -0.662327 |
| C | 0.882431 | 4.289414 | -0.733478 | 0.916516 | 4.282755 | -0.786485 | 0.933939 | 4.264721 | -0.829573 | 0.936912 | 4.225639 | -1.045510 | 0.941548 | 4.265949 | -0.857821 | 0.925235 | 4.271217 | -0.846102 |
| C | 2.263316 | 4.353246 | -0.631897 | 2.295702 | 4.340216 | -0.655406 | 2.314366 | 4.316118 | -0.696284 | 2.302011 | 4.299423 | -0.804507 | 2.319971 | 4.317921 | -0.703499 | 2.302257 | 4.330350 | -0.688326 |
| C | 3.012229 | 3.220382 | -0.346705 | 3.031193 | 3.205624 | -0.342801 | 3.042477 | 3.181680 | -0.364043 | 3.024867 | 3.176734 | -0.422833 | 3.045061 | 3.181828 | -0.369412 | 3.033685 | 3.196797 | -0.361031 |
| O | 2.014546 | -1.326020 | 0.547488 | 1.995361 | -1.329181 | 0.558688 | 1.976419 | -1.335743 | 0.592429 | 1.969905 | -1.404563 | 0.267868 | 1.970350 | -1.353791 | 0.502693 | 1.982738 | -1.352122 | 0.476024 |
| C | -4.330488 | -0.078571 | 0.127266 | -4.333036 | -0.066651 | 0.131692 | -4.336531 | -0.056674 | 0.140888 | -4.298027 | -0.047943 | 0.206556 | -4.330689 | -0.053346 | 0.151788 | -4.326892 | -0.058598 | 0.146858 |
| C | -5.172809 | -0.384952 | -0.932452 | -5.171035 | -0.240685 | -0.960575 | -5.151250 | -0.025873 | -0.981417 | -5.225202 | -0.073840 | -0.824905 | -5.170475 | 0.006812 | -0.950755 | -5.180584 | -0.077166 | -0.946507 |
| C | -6.528721 | -0.119505 | -0.844601 | -6.526731 | 0.015666 | -0.843559 | -6.506864 | 0.223922 | -0.845324 | -6.558560 | 0.192594 | -0.561482 | -6.521911 | 0.257632 | -0.777727 | -6.531068 | 0.177976 | -0.775194 |
| C | -7.013199 | 0.471145 | 0.305854 | -7.009528 | 0.458628 | 0.371593 | -7.007591 | 0.445050 | 0.421851 | -6.923380 | 0.490627 | 0.736059 | -6.993105 | 0.449454 | 0.505654 | -6.989699 | 0.457982 | 0.496552 |
| C | -6.191058 | 0.798542 | 1.366123 | -6.193690 | 0.648985 | 1.468892 | -6.216529 | 0.422953 | 1.553005 | -6.017613 | 0.528947 | 1.777083 | -6.176581 | 0.398034 | 1.617805 | -6.158372 | 0.488889 | 1.598378 |
| C | -4.839806 | 0.513632 | 1.273647 | -4.842294 | 0.375919 | 1.343467 | -4.864144 | 0.163148 | 1.404491 | -4.688973 | 0.249672 | 1.503615 | -4.828714 | 0.138211 | 1.432129 | -4.812019 | 0.221553 | 1.415661 |
| F | -8.321918 | 0.737083 | 0.394531 | -8.321395 | 0.714603 | 0.490025 | -8.322496 | 0.692075 | 0.560401 | -8.215790 | 0.755128 | 0.996886 | -8.303660 | 0.696626 | 0.679996 | -8.297754 | 0.711037 | 0.668418 |
| C | 4.260808 | 0.510655 | 0.233623 | 4.250260 | 0.495319 | 0.301129 | 4.240548 | 0.478861 | 0.353793 | 4.191296 | 0.480221 | 0.398793 | 4.232101 | 0.476681 | 0.362679 | 4.238413 | 0.488885 | 0.329623 |
| O | 4.545499 | -0.730701 | -0.090050 | 4.553704 | -0.722272 | -0.081057 | 4.563998 | -0.719496 | -0.062179 | 4.564789 | -0.730945 | 0.082289 | 4.567044 | -0.726763 | -0.027955 | 4.561018 | -0.728684 | -0.030355 |
| O | 5.025323 | 1.364792 | 0.594265 | 4.990423 | 1.333503 | 0.742375 | 4.955578 | 1.307697 | 0.852858 | 4.851214 | 1.339020 | 0.929176 | 4.934617 | 1.311834 | 0.869622 | 4.957788 | 1.333986 | 0.794131 |
| C | 5.857452 | -1.318758 | 0.183883 | 5.863655 | -1.323587 | 0.200178 | 5.882758 | -1.316687 | 0.214708 | 5.863290 | -1.300155 | 0.503618 | 5.879947 | -1.318488 | 0.286398 | 5.868134 | -1.321133 | 0.294557 |
| C | 6.160172 | -1.233871 | 1.673264 | 6.112445 | -1.335621 | 1.700492 | 6.119129 | -1.372102 | 1.714902 | 5.960658 | -1.285812 | 2.019552 | 6.080966 | -1.356522 | 1.792525 | 6.074378 | -1.315573 | 1.800977 |
| C | 5.661909 | -2.764989 | -0.244085 | 5.695010 | -2.738372 | -0.328808 | 5.738695 | -2.715472 | -0.358913 | 5.772246 | -2.723175 | -0.018665 | 5.748990 | -2.724095 | -0.274532 | 5.720083 | -2.741885 | -0.223608 |
| C | 6.921698 | -0.638365 | -0.665402 | 6.949502 | -0.584312 | -0.566417 | 6.963290 | -0.541044 | -0.520346 | 6.994842 | -0.545006 | -0.172173 | 6.976144 | -0.549694 | -0.433022 | 6.968854 | -0.583733 | -0.451446 |
| H | 0.811839 | -2.662794 | -0.514436 | 0.798956 | -2.646864 | -0.602946 | 0.786906 | -2.625976 | -0.687564 | 0.722648 | -2.622166 | -1.116283 | 0.772415 | -2.623556 | -0.796697 | 0.779615 | -2.638981 | -0.758242 |
| H | -0.356588 | -4.798719 | -0.784533 | -0.377377 | -4.776037 | -0.892094 | -0.400392 | -4.740824 | -1.044083 | -0.498141 | -4.731793 | -1.408852 | -0.423707 | -4.736774 | -1.137792 | -0.413324 | -4.756359 | -1.078558 |
| H | -2.811760 | -4.921335 | -0.589027 | -2.832070 | -4.894037 | -0.683158 | -2.856859 | -4.851943 | -0.848638 | -2.927374 | -4.843540 | -1.002058 | -2.875694 | -4.847383 | -0.891563 | -2.865432 | -4.866680 | -0.837630 |
| H | -4.137768 | -2.861525 | -0.199483 | -4.151094 | -2.836747 | -0.257039 | -4.169353 | -2.802188 | -0.362446 | -4.190218 | -2.804615 | -0.366769 | -4.176802 | -2.798019 | -0.370774 | -4.168867 | -2.812640 | -0.343157 |
| H | -0.859918 | 3.053796 | -0.599553 | -0.834139 | 3.056467 | -0.684225 | -0.822799 | 3.046553 | -0.722845 | -0.798274 | 2.974913 | -1.094940 | -0.814788 | 3.044014 | -0.788098 | -0.824695 | 3.041410 | -0.781359 |
| H | 4.086005 | 3.262654 | -0.263984 | 4.104161 | 3.245052 | -0.243808 | 4.116994 | 3.218349 | -0.276398 | 4.090996 | 3.232942 | -0.268789 | 4.118282 | 3.218855 | -0.267256 | 4.105411 | 3.239085 | -0.249375 |
| H | 0.314958 | 5.182120 | -0.960923 | 0.359345 | 5.175878 | -1.037677 | 0.383810 | 5.156526 | -1.101144 | 0.396538 | 5.107369 | -1.365302 | 0.394455 | 5.158986 | -1.131413 | 0.372592 | 5.162981 | -1.112191 |
| H | 2.773001 | 5.297304 | -0.777389 | 2.814101 | 5.279621 | -0.801346 | 2.839018 | 5.249035 | -0.861088 | 2.821988 | 5.240905 | -0.931018 | 2.845713 | 5.252640 | -0.853876 | 2.822426 | 5.269807 | -0.827978 |
| H | -4.763319 | -0.831491 | -1.829284 | -4.760009 | -0.576965 | -1.903750 | -4.723760 | -0.199043 | -1.960756 | -4.904199 | -0.303950 | -1.832844 | -4.766056 | -0.144324 | -1.943408 | -4.786713 | -0.291512 | -1.931732 |
| H | -4.173064 | 0.761711 | 2.087702 | -4.177901 | 0.511680 | 2.186273 | -4.215905 | 0.131530 | 2.270607 | -3.953821 | 0.263993 | 2.298042 | -4.160634 | 0.084681 | 2.282014 | -4.133491 | 0.233455 | 2.258608 |
| H | -7.203836 | -0.352054 | -1.656556 | -7.200906 | -0.113817 | -1.679330 | -7.164387 | 0.251591 | -1.704016 | -7.302423 | 0.176525 | -1.347106 | -7.198773 | 0.308144 | -1.620206 | -7.218094 | 0.168914 | -1.610734 |
| H | -6.609545 | 1.265492 | 2.246974 | -6.614159 | 1.000875 | 2.401149 | -6.653275 | 0.600947 | 2.526697 | -6.348350 | 0.767357 | 2.779290 | -6.590364 | 0.553977 | 2.605183 | -6.561554 | 0.715079 | 2.576344 |
| H | 7.066824 | -1.802682 | 1.884297 | 7.008431 | -1.923227 | 1.906867 | 7.019646 | -1.958056 | 1.905138 | 6.838890 | -1.861347 | 2.316279 | 6.976298 | -1.940798 | 2.010357 | 6.966387 | -1.898165 | 2.036646 |
| H | 5.341571 | -1.668105 | 2.248987 | 5.273110 | -1.800113 | 2.220807 | 5.281599 | -1.861271 | 2.215656 | 5.079381 | -1.753919 | 2.461830 | 5.231245 | -1.839644 | 2.278329 | 5.222536 | -1.778049 | 2.302699 |
| H | 6.315736 | -0.205412 | 1.992115 | 6.264128 | -0.332543 | 2.094301 | 6.259902 | -0.380530 | 2.140996 | 6.065103 | -0.277014 | 2.415012 | 6.212324 | -0.360119 | 2.210240 | 6.212159 | -0.307882 | 2.188431 |
| H | 4.857489 | -3.224747 | 0.329989 | 4.885101 | -3.249519 | 0.192556 | 4.937211 | -3.258387 | 0.143953 | 4.928744 | -3.246274 | 0.434743 | 4.936559 | -3.261078 | 0.217094 | 4.900590 | -3.252581 | 0.283578 |
| H | 6.581789 | -3.326647 | -0.077732 | 6.617628 | -3.300076 | -0.177404 | 6.671318 | -3.262915 | -0.217932 | 6.688497 | -3.260282 | 0.228605 | 6.678411 | -3.269418 | -0.106878 | 6.642071 | -3.295556 | -0.042078 |
| H | 5.407306 | -2.819527 | -1.303002 | 5.468677 | -2.726597 | -1.395960 | 5.519734 | -2.674282 | -1.427103 | 5.648654 | -2.728632 | -1.102902 | 5.553297 | -2.694459 | -1.347583 | 5.521702 | -2.742171 | -1.296521 |
| H | 6.636886 | -0.659860 | -1.718441 | 6.697986 | -0.529373 | -1.627129 | 6.720684 | -0.459493 | -1.581521 | 6.834872 | -0.504866 | -1.251133 | 6.756555 | -0.480841 | -1.500041 | 6.745733 | -0.544341 | -1.519247 |
| H | 7.073193 | 0.394051 | -0.358962 | 7.094921 | 0.423707 | -0.184119 | 7.095006 | 0.456953 | -0.107208 | 7.094952 | 0.468708 | 0.211246 | 7.097217 | 0.453102 | -0.028225 | 7.098417 | 0.429906 | -0.077720 |
| H | 7.863362 | -1.178854 | -0.556618 | 7.888820 | -1.131375 | -0.468822 | 7.906875 | -1.081335 | -0.427551 | 7.929263 | -1.076836 | 0.014814 | 7.917836 | -1.087848 | -0.312836 | 7.907918 | -1.124126 | -0.320312 |

| **iso-I** 7 |  | gasphase |  |  | toluene |  |  | DMSO |  |  | methanol |  |  | acetonitrile |  |  | chloroform |  |
| --- | --- | --- | --- | --- | --- | --- | --- | --- | --- | --- | --- | --- | --- | --- | --- | --- | --- | --- |
| Element | x | y | z | x | y | z | x | y | z | x | y | z | x | y | z | x | y | z |
| C | 1.239580 | -2.772667 | -0.959867 | 1.237784 | -2.767062 | -0.931512 | 1.237372 | -2.750086 | -0.940012 | 1.251035 | -2.723015 | -1.102459 | 1.238400 | -2.752583 | -0.953693 | 1.231746 | -2.756496 | -0.945844 |
| C | 1.968802 | -3.946495 | -1.090998 | 1.967510 | -3.938821 | -1.078067 | 1.968039 | -3.918743 | -1.108954 | 1.984352 | -3.888856 | -1.277285 | 1.969181 | -3.921887 | -1.118941 | 1.959540 | -3.928728 | -1.100662 |
| C | 3.333220 | -3.966938 | -0.848631 | 3.333548 | -3.960042 | -0.840386 | 3.336143 | -3.941926 | -0.873693 | 3.343510 | -3.922245 | -0.995164 | 3.336291 | -3.945794 | -0.877440 | 3.325874 | -3.954234 | -0.860646 |
| C | 4.006433 | -2.808580 | -0.483115 | 4.007890 | -2.804172 | -0.468510 | 4.011663 | -2.791204 | -0.486696 | 4.009876 | -2.784462 | -0.555812 | 4.010797 | -2.795156 | -0.487680 | 4.002658 | -2.802105 | -0.480408 |
| C | 3.268234 | -1.651037 | -0.355431 | 3.268693 | -1.648366 | -0.329095 | 3.270634 | -1.638752 | -0.327090 | 3.265713 | -1.636398 | -0.388037 | 3.269732 | -1.642256 | -0.331529 | 3.264977 | -1.646181 | -0.333606 |
| C | 1.881572 | -1.601918 | -0.569959 | 1.880480 | -1.599876 | -0.532422 | 1.881511 | -1.591323 | -0.519646 | 1.884598 | -1.581850 | -0.623308 | 1.881423 | -1.594018 | -0.530557 | 1.876986 | -1.594534 | -0.535464 |
| N | 3.753911 | -0.376017 | -0.065006 | 3.752880 | -0.375646 | -0.029401 | 3.752186 | -0.368851 | -0.013467 | 3.736635 | -0.369597 | -0.032002 | 3.750587 | -0.371912 | -0.016319 | 3.750551 | -0.374984 | -0.027672 |
| C | 2.743526 | 0.560342 | -0.134273 | 2.741657 | 0.557001 | -0.072278 | 2.738920 | 0.558389 | -0.026517 | 2.726824 | 0.553239 | -0.064715 | 2.738097 | 0.555373 | -0.036553 | 2.741405 | 0.557061 | -0.062759 |
| C | 1.464170 | -0.200488 | -0.432025 | 1.462841 | -0.200586 | -0.379781 | 1.463104 | -0.196527 | -0.346933 | 1.463910 | -0.192189 | -0.434828 | 1.463133 | -0.198707 | -0.360159 | 1.462400 | -0.195817 | -0.374740 |
| C | 0.244939 | 0.393835 | -0.469104 | 0.242971 | 0.391375 | -0.418421 | 0.241645 | 0.388457 | -0.382315 | 0.243059 | 0.391180 | -0.476880 | 0.242294 | 0.387779 | -0.396867 | 0.243195 | 0.396233 | -0.409198 |
| O | 2.903741 | 1.736665 | 0.079950 | 2.896268 | 1.729354 | 0.174135 | 2.883465 | 1.724397 | 0.263266 | 2.858027 | 1.718089 | 0.254911 | 2.882163 | 1.722728 | 0.249615 | 2.894553 | 1.728420 | 0.197347 |
| C | -0.161483 | 1.803260 | -0.548687 | -0.162322 | 1.799057 | -0.517056 | -0.164243 | 1.791882 | -0.505028 | -0.160889 | 1.792781 | -0.599395 | -0.162499 | 1.791952 | -0.516893 | -0.161226 | 1.802901 | -0.515559 |
| C | -1.561538 | 1.846061 | -0.459588 | -1.563013 | 1.842404 | -0.440554 | -1.564741 | 1.833962 | -0.433965 | -1.558618 | 1.838069 | -0.503569 | -1.562827 | 1.835351 | -0.441685 | -1.561786 | 1.846648 | -0.439008 |
| N | -2.070290 | 0.554111 | -0.303199 | -2.071729 | 0.552300 | -0.271909 | -2.070483 | 0.546022 | -0.245056 | -2.061614 | 0.547415 | -0.302253 | -2.069363 | 0.547094 | -0.253587 | -2.069742 | 0.556985 | -0.262623 |
| C | -1.051367 | -0.380522 | -0.346862 | -1.053613 | -0.380134 | -0.284826 | -1.052392 | -0.381724 | -0.222698 | -1.046391 | -0.375963 | -0.301732 | -1.052137 | -0.381204 | -0.237862 | -1.052418 | -0.372954 | -0.262291 |
| C | 0.513716 | 2.992395 | -0.803062 | 0.515581 | 2.983878 | -0.783892 | 0.515041 | 2.969287 | -0.799601 | 0.517909 | 2.961575 | -0.925933 | 0.517201 | 2.969132 | -0.812269 | 0.517521 | 2.984908 | -0.793333 |
| C | -0.200029 | 4.176161 | -0.927364 | -0.195997 | 4.166659 | -0.927695 | -0.196766 | 4.149255 | -0.966828 | -0.193586 | 4.142163 | -1.088260 | -0.193970 | 4.150193 | -0.975749 | -0.193843 | 4.167289 | -0.943920 |
| C | -1.581142 | 4.188658 | -0.815348 | -1.578350 | 4.181011 | -0.822000 | -1.580477 | 4.164888 | -0.857302 | -1.574635 | 4.163330 | -0.947298 | -1.577425 | 4.167084 | -0.861932 | -1.576638 | 4.182723 | -0.834710 |
| C | -2.284770 | 3.013301 | -0.587853 | -2.284699 | 3.008978 | -0.585286 | -2.287620 | 2.997517 | -0.598638 | -2.281681 | 3.000828 | -0.664960 | -2.285006 | 2.999862 | -0.602760 | -2.283629 | 3.012389 | -0.589645 |
| O | -1.227799 | -1.567504 | -0.228142 | -1.226052 | -1.564816 | -0.128579 | -1.216243 | -1.560879 | -0.009696 | -1.203360 | -1.559865 | -0.083834 | -1.216412 | -1.561620 | -0.030591 | -1.221312 | -1.556215 | -0.081648 |
| C | 5.089636 | -0.066322 | 0.292321 | 5.098932 | -0.064864 | 0.294214 | 5.102840 | -0.060724 | 0.297041 | 5.072485 | -0.065422 | 0.342142 | 5.099594 | -0.062311 | 0.301328 | 5.098151 | -0.067376 | 0.295862 |
| C | 5.812637 | 0.857673 | -0.448138 | 5.836135 | 0.766380 | -0.536251 | 5.846296 | 0.727348 | -0.569474 | 5.824703 | 0.801278 | -0.437493 | 5.846285 | 0.728006 | -0.560646 | 5.841759 | 0.747901 | -0.544934 |
| C | 7.114145 | 1.166731 | -0.092972 | 7.148160 | 1.075314 | -0.220743 | 7.163536 | 1.032404 | -0.269392 | 7.125706 | 1.105740 | -0.073101 | 7.161424 | 1.035402 | -0.253153 | 7.155101 | 1.053570 | -0.229992 |
| C | 7.675131 | 0.530277 | 0.996644 | 7.698420 | 0.528585 | 0.921185 | 7.706616 | 0.523222 | 0.892952 | 7.644201 | 0.517465 | 1.062832 | 7.699212 | 0.526495 | 0.911947 | 7.697296 | 0.518923 | 0.921399 |
| C | 6.975516 | -0.394326 | 1.746829 | 6.986792 | -0.305281 | 1.760253 | 6.990273 | -0.269417 | 1.766738 | 6.917531 | -0.352768 | 1.850270 | 6.979396 | -0.267886 | 1.781565 | 6.979756 | -0.299450 | 1.770554 |
| C | 5.668883 | -0.683295 | 1.392789 | 5.670736 | -0.594767 | 1.442368 | 5.669852 | -0.556348 | 1.462522 | 5.612822 | -0.638515 | 1.484558 | 5.661033 | -0.557070 | 1.470034 | 5.662796 | -0.586116 | 1.452638 |
| F | 8.936416 | 0.819742 | 1.337932 | 8.972366 | 0.818588 | 1.227377 | 8.987934 | 0.810622 | 1.185589 | 8.909998 | 0.803239 | 1.416758 | 8.978363 | 0.816043 | 1.211732 | 8.973593 | 0.806228 | 1.227736 |
| C | -3.423539 | 0.225055 | -0.063255 | -3.430665 | 0.225161 | -0.052097 | -3.432805 | 0.221472 | -0.035848 | -3.418332 | 0.222716 | -0.052851 | -3.431191 | 0.222470 | -0.038998 | -3.429675 | 0.228897 | -0.045464 |
| C | -4.057388 | -0.731989 | -0.841157 | -4.063952 | -0.697138 | -0.870698 | -4.064955 | -0.678079 | -0.880293 | -4.067996 | -0.693516 | -0.865053 | -4.066976 | -0.676969 | -0.881058 | -4.060553 | -0.686832 | -0.873381 |
| C | -5.380206 | -1.055178 | -0.593116 | -5.391156 | -1.020541 | -0.645470 | -5.394810 | -1.000858 | -0.668468 | -5.390058 | -1.018805 | -0.611237 | -5.396102 | -0.999772 | -0.663726 | -5.387688 | -1.013836 | -0.651848 |
| C | -6.071835 | -0.417877 | 0.424158 | -6.083703 | -0.414219 | 0.390094 | -6.086623 | -0.413845 | 0.379590 | -6.055842 | -0.417459 | 0.445196 | -6.083684 | -0.413056 | 0.387421 | -6.080324 | -0.416505 | 0.389423 |
| C | -5.436958 | 0.539924 | 1.203328 | -5.450068 | 0.512448 | 1.208155 | -5.454707 | 0.494075 | 1.220224 | -5.405462 | 0.507237 | 1.253283 | -5.447959 | 0.494332 | 1.225988 | -5.449180 | 0.505223 | 1.215325 |
| C | -4.113194 | 0.854589 | 0.966841 | -4.122370 | 0.825811 | 0.993367 | -4.124261 | 0.806376 | 1.017719 | -4.082754 | 0.822188 | 1.009717 | -4.118200 | 0.806616 | 1.018064 | -4.121416 | 0.822004 | 1.003805 |
| C | -7.523725 | -0.721351 | 0.677664 | -7.536070 | -0.718669 | 0.631186 | -7.539985 | -0.716494 | 0.606993 | -7.500486 | -0.724798 | 0.719921 | -7.536563 | -0.715633 | 0.620289 | -7.532061 | -0.724499 | 0.626437 |
| F | -7.915130 | -1.861604 | 0.098736 | -7.959877 | -1.787589 | -0.052276 | -7.965604 | -1.778407 | -0.087715 | -7.945228 | -1.788200 | 0.039937 | -7.964025 | -1.779345 | -0.070597 | -7.951864 | -1.798028 | -0.053024 |
| F | -8.318034 | 0.253251 | 0.202604 | -8.324380 | 0.311235 | 0.271138 | -8.327260 | 0.317722 | 0.251163 | -8.301626 | 0.307162 | 0.389906 | -8.325270 | 0.317541 | 0.264380 | -8.324421 | 0.300823 | 0.258588 |
| F | -7.788273 | -0.827867 | 1.987694 | -7.790209 | -0.954256 | 1.928111 | -7.808451 | -0.964333 | 1.900289 | -7.725042 | -0.973135 | 2.021504 | -7.800831 | -0.960411 | 1.914997 | -7.792600 | -0.955195 | 1.923606 |
| H | 0.179582 | -2.773508 | -1.142258 | 0.176855 | -2.767759 | -1.111188 | 0.176534 | -2.747650 | -1.124698 | 0.199360 | -2.708765 | -1.337116 | 0.178474 | -2.749068 | -1.143862 | 0.171328 | -2.753556 | -1.131140 |
| H | 1.459772 | -4.854850 | -1.384345 | 1.458298 | -4.845132 | -1.378512 | 1.460751 | -4.820593 | -1.426741 | 1.487713 | -4.779676 | -1.639935 | 1.462786 | -4.823460 | -1.438875 | 1.449578 | -4.831830 | -1.409955 |
| H | 3.887024 | -4.891282 | -0.953568 | 3.887296 | -4.883573 | -0.954381 | 3.890417 | -4.863166 | -1.003927 | 3.899837 | -4.841221 | -1.132836 | 3.890655 | -4.867338 | -1.004988 | 3.877892 | -4.878157 | -0.980511 |
| H | 5.075110 | -2.808705 | -0.319028 | 5.077098 | -2.806492 | -0.305886 | 5.081598 | -2.796090 | -0.326949 | 5.074756 | -2.797024 | -0.365679 | 5.079976 | -2.800407 | -0.323195 | 5.071828 | -2.808024 | -0.316636 |
| H | 1.586117 | 2.997517 | -0.885689 | 1.588654 | 2.987372 | -0.863201 | 1.588199 | 2.970008 | -0.887544 | 1.587908 | 2.955801 | -1.053363 | 1.590061 | 2.968730 | -0.903985 | 1.590494 | 2.987051 | -0.879044 |
| H | -3.364306 | 3.011606 | -0.531189 | -3.364636 | 3.011177 | -0.532596 | -3.367619 | 3.002390 | -0.542819 | -3.360605 | 3.009897 | -0.590857 | -3.364784 | 3.005547 | -0.543681 | -3.363523 | 3.016173 | -0.534218 |
| H | 0.334047 | 5.098238 | -1.114079 | 0.339535 | 5.086283 | -1.123908 | 0.336933 | 5.065157 | -1.185906 | 0.337231 | 5.053683 | -1.331340 | 0.340043 | 5.065779 | -1.195288 | 0.341256 | 5.085454 | -1.148837 |
| H | -2.123878 | 5.119917 | -0.916275 | -2.119727 | 5.111818 | -0.935703 | -2.122081 | 5.093620 | -0.987229 | -2.115461 | 5.092872 | -1.075058 | -2.118515 | 5.096503 | -0.988946 | -2.117805 | 5.113192 | -0.952966 |
| H | 5.350284 | 1.342582 | -1.296418 | 5.379552 | 1.175158 | -1.427570 | 5.394604 | 1.101700 | -1.478770 | 5.392798 | 1.237770 | -1.328660 | 5.398494 | 1.102615 | -1.471757 | 5.391037 | 1.145962 | -1.444346 |
| H | 5.092686 | -1.391248 | 1.974306 | 5.083112 | -1.234117 | 2.088495 | 5.078197 | -1.168339 | 2.131246 | 5.013858 | -1.309206 | 2.087305 | 5.066766 | -1.170212 | 2.135335 | 5.070356 | -1.214842 | 2.104848 |
| H | 7.694180 | 1.888142 | -0.651732 | 7.741488 | 1.725297 | -0.849435 | 7.763052 | 1.647608 | -0.927104 | 7.731606 | 1.781175 | -0.662470 | 7.763345 | 1.652334 | -0.907012 | 7.753839 | 1.690871 | -0.866772 |
| H | 7.446828 | -0.865016 | 2.598546 | 7.454649 | -0.707614 | 2.648530 | 7.455606 | -0.646446 | 2.667701 | 7.362606 | -0.789569 | 2.734383 | 7.440470 | -0.644235 | 2.684986 | 7.442600 | -0.693486 | 2.665344 |
| H | -3.512471 | -1.228356 | -1.630863 | -3.519109 | -1.163841 | -1.679062 | -3.521043 | -1.122016 | -1.702816 | -3.543762 | -1.149470 | -1.694139 | -3.526118 | -1.121498 | -1.705221 | -3.515032 | -1.143753 | -1.687374 |
| H | -3.606408 | 1.583661 | 1.584982 | -3.616432 | 1.531766 | 1.638619 | -3.619464 | 1.501255 | 1.675966 | -3.563550 | 1.529237 | 1.643417 | -3.610546 | 1.501068 | 1.674515 | -3.617072 | 1.525072 | 1.653549 |
| H | -5.871783 | -1.804805 | -1.197225 | -5.881798 | -1.742549 | -1.283329 | -5.884947 | -1.703092 | -1.328649 | -5.893765 | -1.734877 | -1.245952 | -5.889075 | -1.701977 | -1.321817 | -5.876576 | -1.730194 | -1.297539 |
| H | -5.972294 | 1.028794 | 2.006486 | -5.988646 | 0.980408 | 2.022001 | -5.994605 | 0.951955 | 2.039067 | -5.924946 | 0.976109 | 2.079246 | -5.984437 | 0.951561 | 2.047420 | -5.988523 | 0.967941 | 2.031737 |

| **iso-I** 8 |  | gasphase |  |  | toluene |  |  | DMSO |  |  | methanol |  |  | acetonitrile |  |  | chloroform |  |
| --- | --- | --- | --- | --- | --- | --- | --- | --- | --- | --- | --- | --- | --- | --- | --- | --- | --- | --- |
| Element | x | y | z | x | y | z | x | y | z | x | y | z | x | y | z | x | y | z |
| C | 0.278781 | 2.914001 | -0.681424 | 0.277227 | 2.905304 | -0.667825 | 0.277681 | 2.885830 | -0.700966 | 0.274482 | 2.872868 | -0.805530 | -0.278825 | 2.914016 | 0.681983 | 0.276180 | 2.900263 | -0.688353 |
| C | 1.087047 | 4.035975 | -0.805540 | 1.084887 | 4.025306 | -0.811783 | 1.086280 | 4.003499 | -0.860889 | 1.081961 | 3.989485 | -0.975579 | -1.087104 | 4.036033 | 0.805694 | 1.084250 | 4.019705 | -0.837295 |
| C | 2.462159 | 3.941839 | -0.662348 | 2.461624 | 3.931635 | -0.675904 | 2.463571 | 3.911858 | -0.712324 | 2.456773 | 3.905857 | -0.799984 | -2.462177 | 3.941911 | 0.662107 | 2.461145 | 3.927179 | -0.694456 |
| C | 3.065851 | 2.717880 | -0.405118 | 3.066929 | 2.710104 | -0.410354 | 3.068668 | 2.694903 | -0.423710 | 3.062501 | 2.697747 | -0.475659 | -3.065813 | 2.717917 | 0.404912 | 3.066459 | 2.707353 | -0.419176 |
| C | 2.249685 | 1.613221 | -0.282625 | 2.250607 | 1.607220 | -0.271316 | 2.250398 | 1.594845 | -0.271920 | 2.244952 | 1.600143 | -0.311383 | -2.249628 | 1.613231 | 0.282838 | 2.249349 | 1.605637 | -0.275893 |
| C | 0.852058 | 1.678662 | -0.399107 | 0.852104 | 1.673357 | -0.374183 | 0.852816 | 1.661550 | -0.377610 | 0.850162 | 1.661466 | -0.439104 | -0.852028 | 1.678647 | 0.399663 | 0.851339 | 1.671482 | -0.381823 |
| N | 2.652824 | 0.291540 | -0.091588 | 2.653181 | 0.287834 | -0.067403 | 2.649461 | 0.278061 | -0.049279 | 2.641036 | 0.283142 | -0.062911 | -2.652762 | 0.291519 | 0.091976 | 2.650595 | 0.286703 | -0.064235 |
| C | 1.569489 | -0.562113 | -0.133175 | 1.570250 | -0.562443 | -0.076321 | 1.567972 | -0.568660 | -0.039525 | 1.562874 | -0.559224 | -0.063766 | -1.569470 | -0.562151 | 0.133995 | 1.568955 | -0.561296 | -0.067452 |
| C | 0.336706 | 0.305514 | -0.301703 | 0.337378 | 0.302233 | -0.257568 | 0.337791 | 0.294604 | -0.242417 | 0.337560 | 0.297150 | -0.291579 | -0.336691 | 0.305494 | 0.302508 | 0.337267 | 0.301529 | -0.258691 |
| C | -0.924996 | -0.193581 | -0.286833 | -0.924767 | -0.194562 | -0.243373 | -0.925586 | -0.194338 | -0.227439 | -0.924572 | -0.191915 | -0.273900 | 0.925011 | -0.193640 | 0.287606 | -0.924529 | -0.194078 | -0.244552 |
| O | 1.654449 | -1.757009 | 0.011699 | 1.649823 | -1.753616 | 0.108880 | 1.639184 | -1.755651 | 0.185937 | 1.627614 | -1.747827 | 0.181095 | -1.654450 | -1.757094 | -0.010467 | 1.644550 | -1.751832 | 0.133719 |
| C | -1.444209 | -1.562675 | -0.407081 | -1.444062 | -1.561281 | -0.382102 | -1.447095 | -1.555868 | -0.386609 | -1.445086 | -1.549720 | -0.447794 | 1.444234 | -1.562726 | 0.407635 | -1.443515 | -1.559364 | -0.391089 |
| C | -2.836931 | -1.500834 | -0.239556 | -2.838426 | -1.497938 | -0.230610 | -2.840811 | -1.490839 | -0.235642 | -2.834692 | -1.490416 | -0.269939 | 2.836928 | -1.500880 | 0.239863 | -2.837147 | -1.496762 | -0.235431 |
| N | -3.233087 | -0.185782 | 0.004698 | -3.233504 | -0.185187 | 0.024340 | -3.230588 | -0.180948 | 0.039729 | -3.219128 | -0.182214 | 0.037535 | 3.233077 | -0.185782 | -0.004183 | -3.230373 | -0.184956 | 0.029265 |
| C | -2.151353 | 0.668657 | -0.048432 | -2.150867 | 0.664581 | 0.004753 | -2.146814 | 0.663011 | 0.043590 | -2.138525 | 0.657004 | 0.031402 | 2.151346 | 0.668646 | 0.049227 | -2.148748 | 0.662503 | 0.014878 |
| C | -0.881340 | -2.788393 | -0.747267 | -0.879823 | -2.783769 | -0.731257 | -0.883805 | -2.769999 | -0.764646 | -0.885091 | -2.748838 | -0.874396 | 0.881433 | -2.788502 | 0.747714 | -0.879650 | -2.777691 | -0.755252 |
| C | -1.693701 | -3.906316 | -0.878101 | -1.692417 | -3.899067 | -0.882083 | -1.698467 | -3.881581 | -0.934995 | -1.700467 | -3.857859 | -1.055288 | 1.693803 | -3.906470 | 0.878163 | -1.692833 | -3.892152 | -0.912108 |
| C | -3.063149 | -3.816740 | -0.684938 | -3.064010 | -3.809250 | -0.698626 | -3.070637 | -3.792924 | -0.743722 | -3.068493 | -3.777719 | -0.832017 | 3.063215 | -3.816877 | 0.684753 | -3.064189 | -3.804137 | -0.720587 |
| C | -3.657107 | -2.601986 | -0.369053 | -3.659476 | -2.596616 | -0.376736 | -3.664725 | -2.585243 | -0.399033 | -3.659762 | -2.580703 | -0.446081 | 3.657118 | -2.602062 | 0.368998 | -3.659129 | -2.594009 | -0.386695 |
| O | -2.226208 | 1.858153 | 0.138291 | -2.219934 | 1.849461 | 0.229577 | -2.205565 | 1.841278 | 0.313842 | -2.187526 | 1.834331 | 0.328654 | 2.226149 | 1.858166 | -0.137332 | -2.213521 | 1.846106 | 0.256340 |
| C | 3.981176 | -0.136542 | 0.150784 | 3.988063 | -0.137839 | 0.153943 | 3.988037 | -0.143181 | 0.166879 | 3.973633 | -0.139434 | 0.188559 | -3.981052 | -0.136541 | -0.150785 | 3.986332 | -0.138295 | 0.159694 |
| C | 4.574375 | -1.073350 | -0.683264 | 4.598440 | -1.000650 | -0.744735 | 4.628313 | -0.921516 | -0.786431 | 4.603793 | -0.993893 | -0.704495 | -4.574654 | -1.073000 | 0.683361 | 4.610993 | -0.967925 | -0.760253 |
| C | 5.868973 | -1.498589 | -0.440899 | 5.899238 | -1.421867 | -0.527858 | 5.933978 | -1.334656 | -0.578984 | 5.900839 | -1.413922 | -0.460788 | -5.869190 | -1.498252 | 0.440599 | 5.912319 | -1.388362 | -0.542504 |
| C | 6.554618 | -0.964014 | 0.631926 | 6.567466 | -0.954160 | 0.585793 | 6.570048 | -0.941340 | 0.581170 | 6.538739 | -0.951073 | 0.672320 | -6.554290 | -0.964005 | -0.632718 | 6.563372 | -0.951964 | 0.593788 |
| C | 5.985710 | -0.029139 | 1.474120 | 5.983522 | -0.091466 | 1.491549 | 5.957171 | -0.161310 | 1.540915 | 5.935005 | -0.095285 | 1.571595 | -5.985021 | -0.029456 | -1.475006 | 5.965431 | -0.121922 | 1.520726 |
| C | 4.684420 | 0.376426 | 1.232262 | 4.676903 | 0.310585 | 1.272422 | 4.647081 | 0.234557 | 1.328209 | 4.632925 | 0.306687 | 1.325266 | -4.683792 | 0.376107 | -1.232734 | 4.658836 | 0.280004 | 1.299513 |
| F | 7.810148 | -1.366016 | 0.864514 | 7.831574 | -1.353181 | 0.796251 | 7.840829 | -1.333527 | 0.784477 | 7.801126 | -1.349914 | 0.909923 | -7.809830 | -1.365978 | -0.865667 | 7.828957 | -1.350610 | 0.806137 |
| C | -6.512362 | 0.064121 | 1.684575 | -6.521324 | 0.125398 | 1.679460 | -6.509227 | 0.168058 | 1.706693 | -6.467043 | 0.102370 | 1.775225 | 6.511630 | 0.064127 | -1.685488 | -6.505212 | 0.146606 | 1.707220 |
| C | -5.219086 | -0.333906 | 1.389244 | -5.220804 | -0.272284 | 1.414693 | -5.206344 | -0.229648 | 1.449306 | -5.174611 | -0.296598 | 1.472320 | 5.218515 | -0.333985 | -1.389555 | -5.204706 | -0.251015 | 1.440112 |
| C | -4.552984 | 0.231766 | 0.310376 | -4.561605 | 0.232417 | 0.302439 | -4.561301 | 0.235764 | 0.312065 | -4.541094 | 0.231512 | 0.356252 | 4.552828 | 0.231770 | -0.310480 | -4.559077 | 0.231966 | 0.310453 |
| C | -5.170851 | 1.204659 | -0.461410 | -5.191462 | 1.139045 | -0.537220 | -5.205068 | 1.097454 | -0.564090 | -5.183900 | 1.157762 | -0.452185 | 5.170942 | 1.204812 | 0.460922 | -5.200771 | 1.114497 | -0.545984 |
| C | -6.458783 | 1.608755 | -0.150977 | -6.486982 | 1.543272 | -0.258416 | -6.503432 | 1.501184 | -0.293614 | -6.471352 | 1.562026 | -0.134524 | 6.458710 | 1.608983 | 0.149888 | -6.496464 | 1.518536 | -0.265296 |
| C | -7.134024 | 1.037180 | 0.916802 | -7.155062 | 1.034895 | 0.845688 | -7.157488 | 1.035452 | 0.838553 | -7.115228 | 1.033159 | 0.975329 | 7.133538 | 1.037342 | -0.918114 | -7.151313 | 1.033080 | 0.857477 |
| H | -0.788060 | 3.003201 | -0.785073 | -0.790499 | 2.994626 | -0.766748 | -0.789392 | 2.972454 | -0.816314 | -0.789740 | 2.950710 | -0.956198 | 0.787986 | 3.003268 | 0.785952 | -0.791181 | 2.988416 | -0.796433 |
| H | 0.631300 | 4.994621 | -1.014592 | 0.628408 | 4.982076 | -1.029284 | 0.632808 | 4.956198 | -1.102721 | 0.630661 | 4.934354 | -1.250041 | -0.631380 | 4.994695 | 1.014759 | 0.628898 | 4.974850 | -1.064835 |
| H | 3.078119 | 4.826810 | -0.760477 | 3.077179 | 4.815661 | -0.786422 | 3.079534 | 4.794325 | -0.833726 | 3.071641 | 4.787635 | -0.931871 | -3.078154 | 4.826918 | 0.759891 | 3.076768 | 4.810843 | -0.808267 |
| H | 4.139972 | 2.629028 | -0.318986 | 4.141751 | 2.622880 | -0.327648 | 4.143333 | 2.610221 | -0.332578 | 4.136298 | 2.619250 | -0.369658 | -4.139920 | 2.629051 | 0.318498 | 4.141171 | 2.621268 | -0.331969 |
| H | 0.181239 | -2.873559 | -0.890543 | 0.184028 | -2.869685 | -0.867982 | 0.179279 | -2.853172 | -0.914186 | 0.173268 | -2.822690 | -1.063012 | -0.181116 | -2.873713 | 0.891219 | 0.183416 | -2.861733 | -0.902108 |
| H | -4.727419 | -2.516459 | -0.242980 | -4.730882 | -2.512072 | -0.256427 | -4.736093 | -2.503232 | -0.274016 | -4.729262 | -2.504981 | -0.302442 | 4.727415 | -2.516501 | 0.242744 | -4.730217 | -2.511162 | -0.260985 |
| H | -1.246143 | -4.857885 | -1.132337 | -1.244250 | -4.848878 | -1.143042 | -1.254138 | -4.826764 | -1.219508 | -1.261154 | -4.793561 | -1.376574 | 1.246266 | -4.858080 | 1.132314 | -1.246254 | -4.839557 | -1.184900 |
| H | -3.682811 | -4.698408 | -0.789547 | -3.683751 | -4.689496 | -0.815894 | -3.691822 | -4.670284 | -0.875155 | -3.690090 | -4.653167 | -0.974167 | 3.682892 | -4.698581 | 0.789049 | -3.684155 | -4.683751 | -0.842064 |
| H | 4.015788 | -1.477271 | -1.515887 | 4.052140 | -1.346183 | -1.611972 | 4.105632 | -1.203033 | -1.691265 | 4.079640 | -1.330512 | -1.589646 | -4.016434 | -1.476657 | 1.516368 | 4.076845 | -1.286755 | -1.645545 |
| H | 4.208974 | 1.094936 | 1.887243 | 4.188023 | 0.975382 | 1.972868 | 4.135014 | 0.840387 | 2.064874 | 4.128363 | 0.969968 | 2.016284 | -4.208025 | 1.094377 | -1.887756 | 4.158706 | 0.921154 | 2.014075 |
| H | 6.348623 | -2.232159 | -1.074090 | 6.394027 | -2.098768 | -1.211069 | 6.454121 | -1.944487 | -1.305672 | 6.412695 | -2.082943 | -1.139823 | -6.349154 | -2.231558 | 1.073860 | 6.418888 | -2.039523 | -1.242193 |
| H | 6.552129 | 0.359435 | 2.309178 | 6.540697 | 0.245628 | 2.355156 | 6.493339 | 0.122301 | 2.436803 | 6.471504 | 0.240640 | 2.449114 | -6.551059 | 0.358879 | -2.310435 | 6.510929 | 0.191756 | 2.400660 |
| H | -7.030514 | -0.380000 | 2.524711 | -7.036257 | -0.269560 | 2.546000 | -7.015671 | -0.196229 | 2.591681 | -6.965020 | -0.310676 | 2.643531 | 7.029457 | -0.380060 | -2.525798 | -7.010784 | -0.231013 | 2.587030 |
| H | -4.719824 | -1.080606 | 1.993407 | -4.712336 | -0.971014 | 2.067009 | -4.687850 | -0.899772 | 2.123759 | -4.656372 | -1.014833 | 2.095492 | 4.719084 | -1.080825 | -1.993418 | -4.687009 | -0.933250 | 2.102757 |
| H | -8.142242 | 1.352302 | 1.152715 | -8.169114 | 1.349191 | 1.057912 | -8.173732 | 1.347988 | 1.044005 | -8.123260 | 1.346498 | 1.217241 | 8.141632 | 1.352525 | -1.154505 | -8.165358 | 1.346773 | 1.071351 |
| H | -4.637615 | 1.645921 | -1.291945 | -4.664341 | 1.525588 | -1.399328 | -4.689844 | 1.444964 | -1.450440 | -4.676639 | 1.555615 | -1.322025 | 4.638019 | 1.646137 | 1.291631 | -4.683841 | 1.481330 | -1.423218 |
| H | -6.937953 | 2.371911 | -0.750538 | -6.977159 | 2.255001 | -0.910431 | -7.007070 | 2.176273 | -0.974076 | -6.974413 | 2.287591 | -0.761507 | 6.938069 | 2.372259 | 0.749158 | -6.996889 | 2.211217 | -0.930110 |

| **iso-I** 9 |  | gasphase |  |  | toluene |  |  | DMSO |  |  | methanol |  |  | acetonitrile |  |  | chloroform |  |
| --- | --- | --- | --- | --- | --- | --- | --- | --- | --- | --- | --- | --- | --- | --- | --- | --- | --- | --- |
| Element | x | y | z | x | y | z | x | y | z | x | y | z | x | y | z | x | y | z |
| C | 0.563249 | 3.219545 | -0.187255 | 0.563922 | 3.207276 | -0.232361 | 0.570462 | 3.189937 | -0.284204 | 0.583579 | 3.186453 | -0.321819 | 0.574101 | 3.190787 | -0.287069 | 0.571947 | 3.200515 | -0.258295 |
| C | -0.399065 | 4.217504 | -0.266890 | -0.398800 | 4.202142 | -0.342000 | -0.390397 | 4.183322 | -0.425209 | -0.372017 | 4.184969 | -0.458204 | -0.385401 | 4.185272 | -0.431162 | -0.387963 | 4.196612 | -0.383372 |
| C | -1.749002 | 3.910786 | -0.210989 | -1.749661 | 3.893307 | -0.298797 | -1.743263 | 3.875033 | -0.388713 | -1.726436 | 3.885178 | -0.403117 | -1.738902 | 3.878687 | -0.397379 | -1.740425 | 3.890837 | -0.341520 |
| C | -2.171995 | 2.592417 | -0.101171 | -2.172261 | 2.576343 | -0.169985 | -2.168939 | 2.560958 | -0.237427 | -2.160801 | 2.574423 | -0.244014 | -2.166567 | 2.565091 | -0.245915 | -2.167296 | 2.576083 | -0.200761 |
| C | -1.205169 | 1.611519 | -0.039973 | -1.203625 | 1.599811 | -0.074176 | -1.200999 | 1.587056 | -0.108654 | -1.197949 | 1.595999 | -0.121159 | -1.199974 | 1.590284 | -0.114152 | -1.200981 | 1.598752 | -0.089623 |
| C | 0.171061 | 1.891777 | -0.053023 | 0.171918 | 1.882009 | -0.075799 | 0.173655 | 1.869953 | -0.098597 | 0.176742 | 1.872669 | -0.118443 | 0.175080 | 1.871396 | -0.100966 | 0.174652 | 1.878830 | -0.085739 |
| N | -1.412904 | 0.234826 | 0.039418 | -1.409173 | 0.224740 | 0.018179 | -1.405441 | 0.213607 | -0.000104 | -1.403993 | 0.218660 | -0.014554 | -1.406194 | 0.216695 | -0.005791 | -1.407974 | 0.223894 | 0.011113 |
| C | -0.211981 | -0.441753 | 0.059544 | -0.211407 | -0.451091 | 0.044813 | -0.210857 | -0.460941 | 0.041840 | -0.213369 | -0.453106 | 0.018419 | -0.212902 | -0.458511 | 0.040441 | -0.212674 | -0.451581 | 0.047313 |
| C | 0.883944 | 0.606939 | 0.003969 | 0.884510 | 0.598330 | -0.003523 | 0.884039 | 0.588384 | -0.006245 | 0.880910 | 0.590697 | -0.027800 | 0.883464 | 0.588635 | -0.005894 | 0.883684 | 0.595028 | -0.002420 |
| C | 2.204646 | 0.297642 | 0.050962 | 2.205185 | 0.294655 | 0.063457 | 2.203626 | 0.292254 | 0.080610 | 2.197527 | 0.292433 | 0.077176 | 2.202349 | 0.290233 | 0.083945 | 2.203002 | 0.291809 | 0.074438 |
| O | -0.123173 | -1.640790 | 0.161813 | -0.120977 | -1.651102 | 0.151117 | -0.117404 | -1.661138 | 0.168324 | -0.116755 | -1.657615 | 0.149230 | -0.121175 | -1.659019 | 0.169731 | -0.120987 | -1.652036 | 0.166933 |
| C | 2.925528 | -0.979483 | -0.099569 | 2.932331 | -0.976408 | -0.096225 | 2.936167 | -0.970247 | -0.094691 | 2.931221 | -0.962459 | -0.123007 | 2.934689 | -0.972730 | -0.091349 | 2.931109 | -0.975372 | -0.097540 |
| C | 4.290482 | -0.718503 | 0.110049 | 4.291612 | -0.713672 | 0.144556 | 4.288800 | -0.707766 | 0.179232 | 4.272283 | -0.717015 | 0.210193 | 4.288279 | -0.708981 | 0.177084 | 4.287894 | -0.714292 | 0.157365 |
| N | 4.466665 | 0.623527 | 0.399516 | 4.456785 | 0.622498 | 0.464039 | 4.443514 | 0.619768 | 0.536571 | 4.414280 | 0.598674 | 0.625769 | 4.443257 | 0.619479 | 0.532453 | 4.449135 | 0.617512 | 0.499244 |
| C | 3.291215 | 1.320390 | 0.357424 | 3.282047 | 1.313593 | 0.413543 | 3.268882 | 1.304994 | 0.481373 | 3.245932 | 1.282778 | 0.554073 | 3.267511 | 1.302093 | 0.485625 | 3.274405 | 1.304322 | 0.454022 |
| C | 2.565461 | -2.269330 | -0.477291 | 2.584106 | -2.257596 | -0.512497 | 2.599074 | -2.236153 | -0.563851 | 2.606166 | -2.199479 | -0.669861 | 2.596418 | -2.240398 | -0.555440 | 2.586031 | -2.247635 | -0.543067 |
| C | 3.541657 | -3.249661 | -0.599301 | 3.565854 | -3.231862 | -0.637428 | 3.586356 | -3.203638 | -0.699886 | 3.594652 | -3.164451 | -0.812057 | 3.584047 | -3.207574 | -0.693018 | 3.569584 | -3.218907 | -0.678419 |
| C | 4.875864 | -2.964903 | -0.353819 | 4.894374 | -2.947211 | -0.358032 | 4.908917 | -2.923075 | -0.380907 | 4.904273 | -2.905934 | -0.426982 | 4.907891 | -2.925116 | -0.380721 | 4.896114 | -2.937825 | -0.381816 |
| C | 5.270146 | -1.681417 | 0.000339 | 5.277439 | -1.670801 | 0.032270 | 5.280727 | -1.658572 | 0.057158 | 5.265173 | -1.664398 | 0.081890 | 5.280564 | -1.659105 | 0.052900 | 5.275761 | -1.668402 | 0.034943 |
| O | 3.206711 | 2.502977 | 0.587521 | 3.180624 | 2.490910 | 0.672997 | 3.145265 | 2.473146 | 0.780544 | 3.104204 | 2.439528 | 0.905575 | 3.142171 | 2.468484 | 0.792776 | 3.160737 | 2.475570 | 0.743403 |
| C | -2.675484 | -0.409207 | 0.058879 | -2.676684 | -0.414352 | 0.055965 | -2.676278 | -0.420062 | 0.054379 | -2.673200 | -0.418071 | 0.054526 | -2.677255 | -0.417125 | 0.051463 | -2.676833 | -0.413802 | 0.056160 |
| C | -3.550129 | -0.247617 | -1.006695 | -3.476922 | -0.422673 | -1.077575 | -3.431437 | -0.543440 | -1.102801 | -3.483721 | -0.455767 | -1.070732 | -3.424083 | -0.564129 | -1.108438 | -3.466326 | -0.454359 | -1.084197 |
| C | -4.788657 | -0.866179 | -0.992265 | -4.718746 | -1.034364 | -1.046715 | -4.676634 | -1.148470 | -1.055320 | -4.725368 | -1.066093 | -1.007625 | -4.668227 | -1.171438 | -1.057921 | -4.708660 | -1.065269 | -1.046088 |
| C | -5.119724 | -1.657218 | 0.090150 | -5.124239 | -1.639908 | 0.125854 | -5.127488 | -1.623933 | 0.159547 | -5.115381 | -1.636124 | 0.187576 | -5.126444 | -1.624804 | 0.162839 | -5.123318 | -1.635395 | 0.140889 |
| C | -4.259048 | -1.844634 | 1.153733 | -4.342176 | -1.651783 | 1.263460 | -4.393022 | -1.516789 | 1.323475 | -4.323319 | -1.617278 | 1.318036 | -4.400207 | -1.493878 | 1.329681 | -4.352547 | -1.614581 | 1.286040 |
| C | -3.030181 | -1.208105 | 1.136100 | -3.108226 | -1.025218 | 1.223939 | -3.153794 | -0.900831 | 1.264588 | -3.088977 | -0.993178 | 1.246338 | -3.161760 | -0.876375 | 1.267395 | -3.117656 | -0.989642 | 1.238564 |
| F | -6.312910 | -2.263794 | 0.107281 | -6.324022 | -2.239931 | 0.160571 | -6.334114 | -2.216193 | 0.211603 | -6.317719 | -2.234923 | 0.253326 | -6.331888 | -2.219079 | 0.217801 | -6.325046 | -2.234708 | 0.182838 |
| H | 5.340096 | 1.073821 | 0.604013 | 5.329506 | 1.063923 | 0.697228 | 5.315490 | 1.049167 | 0.801826 | 5.271252 | 1.014552 | 0.955942 | 5.313825 | 1.049417 | 0.801881 | 5.319790 | 1.053029 | 0.754959 |
| H | 1.608223 | 3.473188 | -0.208168 | 1.608905 | 3.462852 | -0.250466 | 1.616458 | 3.444024 | -0.305381 | 1.631110 | 3.433880 | -0.370289 | 1.620538 | 3.443742 | -0.306566 | 1.617973 | 3.453595 | -0.279630 |
| H | -0.083763 | 5.247862 | -0.363610 | -0.084465 | 5.231470 | -0.455087 | -0.075548 | 5.210040 | -0.561363 | -0.052764 | 5.208550 | -0.607145 | -0.069021 | 5.211480 | -0.567489 | -0.071376 | 5.224010 | -0.508596 |
| H | -2.486324 | 4.702245 | -0.257783 | -2.487511 | 4.682470 | -0.372933 | -2.479708 | 4.662648 | -0.490059 | -2.458494 | 4.677502 | -0.499873 | -2.474109 | 4.667145 | -0.501033 | -2.476067 | 4.680910 | -0.428059 |
| H | -3.222992 | 2.341107 | -0.065495 | -3.223541 | 2.322590 | -0.151509 | -3.220895 | 2.308168 | -0.231760 | -3.214704 | 2.330283 | -0.229176 | -3.218690 | 2.313000 | -0.242548 | -3.219519 | 2.325374 | -0.186653 |
| H | 1.533377 | -2.512412 | -0.657166 | 1.556911 | -2.499331 | -0.722053 | 1.577780 | -2.471061 | -0.810462 | 1.597169 | -2.414087 | -0.980969 | 1.574087 | -2.476702 | -0.796624 | 1.560939 | -2.484976 | -0.769772 |
| H | 6.310841 | -1.440823 | 0.175159 | 6.313517 | -1.429465 | 0.231961 | 6.311103 | -1.417373 | 0.284179 | 6.287885 | -1.439558 | 0.355610 | 6.311648 | -1.416674 | 0.275311 | 6.309973 | -1.429008 | 0.246139 |
| H | 3.249319 | -4.251202 | -0.885340 | 3.283852 | -4.227853 | -0.952958 | 3.316699 | -4.189230 | -1.057316 | 3.337464 | -4.129796 | -1.229013 | 3.313538 | -4.194434 | -1.046232 | 3.291915 | -4.208916 | -1.016417 |
| H | 5.621208 | -3.744755 | -0.445633 | 5.644489 | -3.722483 | -0.452747 | 5.663768 | -3.692752 | -0.485324 | 5.659694 | -3.674148 | -0.538227 | 5.662908 | -3.694434 | -0.486445 | 5.647723 | -3.710700 | -0.485154 |
| H | -3.258100 | 0.364192 | -1.850358 | -3.125033 | 0.050648 | -1.985220 | -3.044862 | -0.163845 | -2.039943 | -3.143852 | -0.006133 | -1.994958 | -3.031808 | -0.201410 | -2.049866 | -3.107110 | -0.006865 | -2.002050 |
| H | -2.336026 | -1.339536 | 1.954388 | -2.474047 | -1.013833 | 2.100332 | -2.555007 | -0.793099 | 2.159691 | -2.446349 | -0.953775 | 2.116411 | -2.568471 | -0.751034 | 2.163926 | -2.491143 | -0.951993 | 2.120013 |
| H | -5.486849 | -0.751796 | -1.809945 | -5.361382 | -1.053205 | -1.916512 | -5.286115 | -1.255140 | -1.942796 | -5.377935 | -1.106244 | -1.869730 | -5.271273 | -1.297027 | -1.947315 | -5.343771 | -1.108472 | -1.920723 |
| H | -4.554350 | -2.476761 | 1.979758 | -4.698752 | -2.140707 | 2.159909 | -4.787117 | -1.903353 | 2.253868 | -4.670131 | -2.076834 | 2.234091 | -4.799617 | -1.864187 | 2.264399 | -4.717059 | -2.076075 | 2.193944 |

| **iso-I** 10 |  | gasphase |  |  | toluene |  |  | DMSO |  |  | methanol |  |  | acetonitrile |  |  | chloroform |  |
| --- | --- | --- | --- | --- | --- | --- | --- | --- | --- | --- | --- | --- | --- | --- | --- | --- | --- | --- |
| Element | x | y | z | x | y | z | x | y | z | x | y | z | x | y | z | x | y | z |
| C | -0.322139 | 2.973382 | -0.603311 | -0.329944 | 2.963484 | -0.678789 | -0.329928 | 2.928246 | -0.785372 | -0.330195 | 2.918206 | -0.867096 | -0.329963 | 2.934819 | -0.787028 | -0.326641 | 2.944132 | -0.764769 |
| C | -1.021771 | 4.171883 | -0.598635 | -1.031897 | 4.160512 | -0.691323 | -1.029710 | 4.126431 | -0.820637 | -1.028763 | 4.117042 | -0.899326 | -1.030140 | 4.133237 | -0.814553 | -1.025386 | 4.143311 | -0.778269 |
| C | -2.385098 | 4.197596 | -0.354138 | -2.396137 | 4.185677 | -0.446823 | -2.392714 | 4.159421 | -0.559963 | -2.386978 | 4.154286 | -0.614668 | -2.392703 | 4.164740 | -0.551258 | -2.386255 | 4.174455 | -0.511749 |
| C | -3.089871 | 3.019888 | -0.141492 | -3.098759 | 3.009720 | -0.217181 | -3.096486 | 2.991382 | -0.294135 | -3.089986 | 2.989526 | -0.330756 | -3.095687 | 2.994921 | -0.290208 | -3.089544 | 3.002760 | -0.261268 |
| C | -2.385802 | 1.834991 | -0.172835 | -2.390837 | 1.826468 | -0.228918 | -2.389193 | 1.807140 | -0.282365 | -2.384102 | 1.805544 | -0.322642 | -2.388233 | 1.810758 | -0.286184 | -2.384615 | 1.817594 | -0.272822 |
|  |  |  |  |  |  |  |  |  |  |  |  |  |  |  |  |  |  |  |
| C | -0.995994 | 1.779274 | -0.367277 | -1.001192 | 1.772478 | -0.421991 | -1.002108 | 1.748433 | -0.484923 | -1.000749 | 1.744213 | -0.542115 | -1.001354 | 1.752912 | -0.491826 | -0.998072 | 1.758941 | -0.482917 |
| N | -2.897219 | 0.543977 | -0.009896 | -2.897586 | 0.535104 | -0.057732 | -2.893795 | 0.518593 | -0.088918 | -2.886012 | 0.514193 | -0.118203 | -2.892578 | 0.521010 | -0.097510 | -2.891486 | 0.527287 | -0.091384 |
| C | -1.888438 | -0.396922 | -0.112321 | -1.890612 | -0.403852 | -0.163032 | -1.894030 | -0.423297 | -0.206127 | -1.889034 | -0.422392 | -0.240695 | -1.893008 | -0.419630 | -0.219262 | -1.891215 | -0.413849 | -0.215506 |
| C | -0.600190 | 0.365535 | -0.346392 | -0.603515 | 0.360642 | -0.394552 | -0.607329 | 0.338657 | -0.442151 | -0.607595 | 0.335918 | -0.493570 | -0.606509 | 0.342529 | -0.454772 | -0.604144 | 0.347175 | -0.451743 |
| C | 0.606283 | -0.250115 | -0.438894 | 0.606981 | -0.247279 | -0.475164 | 0.607137 | -0.259353 | -0.505950 | 0.606458 | -0.261514 | -0.553254 | 0.607148 | -0.257295 | -0.520459 | 0.607284 | -0.258547 | -0.522013 |
| O | -2.063623 | -1.581536 | 0.024943 | -2.062268 | -1.590237 | -0.024521 | -2.065928 | -1.611717 | -0.067658 | -2.054660 | -1.614079 | -0.086453 | -2.064929 | -1.608789 | -0.084677 | -2.065579 | -1.602263 | -0.085358 |
| C | 0.978294 | -1.656481 | -0.641470 | 0.986344 | -1.647294 | -0.697155 | 0.997278 | -1.650638 | -0.746285 | 0.998000 | -1.649114 | -0.802105 | 0.995348 | -1.650047 | -0.757332 | 0.990892 | -1.655912 | -0.745500 |
| C | 2.376050 | -1.743771 | -0.552010 | 2.382287 | -1.732237 | -0.585010 | 2.391798 | -1.728662 | -0.617979 | 2.389175 | -1.730246 | -0.650968 | 2.388979 | -1.731413 | -0.620517 | 2.385258 | -1.739608 | -0.615908 |
| N | 2.916830 | -0.482028 | -0.283235 | 2.912867 | -0.474302 | -0.281836 | 2.908682 | -0.473517 | -0.283118 | 2.899733 | -0.475448 | -0.294368 | 2.907069 | -0.476069 | -0.286968 | 2.909284 | -0.481837 | -0.298738 |
| C | 1.920589 | 0.479379 | -0.255041 | 1.914238 | 0.481383 | -0.245573 | 1.903283 | 0.470911 | -0.227661 | 1.895453 | 0.463033 | -0.246793 | 1.904273 | 0.471049 | -0.243429 | 1.908740 | 0.469951 | -0.267498 |
| C | 0.275051 | -2.798094 | -1.012959 | 0.292153 | -2.781228 | -1.106644 | 0.312631 | -2.776878 | -1.191171 | 0.319087 | -2.764493 | -1.280947 | 0.310724 | -2.775254 | -1.205302 | 0.301834 | -2.786249 | -1.173201 |
| C | 0.958799 | -3.982673 | -1.245155 | 0.982277 | -3.960188 | -1.348496 | 1.011679 | -3.948317 | -1.445955 | 1.021310 | -3.933415 | -1.537989 | 1.008468 | -3.949131 | -1.453398 | 0.995792 | -3.963653 | -1.413271 |
| C | 2.338235 | -4.042057 | -1.126843 | 2.359814 | -4.019479 | -1.201859 | 2.388604 | -4.003506 | -1.278588 | 2.394477 | -3.993825 | -1.342866 | 2.384127 | -4.007838 | -1.276215 | 2.371965 | -4.022680 | -1.248179 |
| C | 3.069988 | -2.912079 | -0.786957 | 3.083261 | -2.895123 | -0.826755 | 3.102449 | -2.883444 | -0.872049 | 3.103585 | -2.880818 | -0.908122 | 3.098287 | -2.888598 | -0.867279 | 3.090401 | -2.900225 | -0.856667 |
| O | 2.126869 | 1.647366 | -0.040312 | 2.107638 | 1.643335 | 0.015748 | 2.075493 | 1.622419 | 0.095762 | 2.058217 | 1.613769 | 0.098545 | 2.079249 | 1.625458 | 0.068886 | 2.092648 | 1.630108 | 0.013775 |
| C | -4.257593 | 0.204905 | 0.161780 | -4.254828 | 0.198030 | 0.149698 | -4.249178 | 0.190705 | 0.149595 | -4.236841 | 0.185368 | 0.147734 | -4.246654 | 0.190563 | 0.145893 | -4.245835 | 0.194765 | 0.145348 |
| C | -5.205770 | 0.673801 | -0.740982 | -5.214421 | 0.600938 | -0.771629 | -5.217887 | 0.550372 | -0.778971 | -5.223539 | 0.548004 | -0.760400 | -5.220280 | 0.553980 | -0.776171 | -5.220694 | 0.581751 | -0.766343 |
| C | -6.538318 | 0.348084 | -0.576619 | -6.542309 | 0.276761 | -0.570717 | -6.542963 | 0.235620 | -0.546325 | -6.543355 | 0.234933 | -0.500023 | -6.544075 | 0.237753 | -0.537858 | -6.545011 | 0.263484 | -0.534261 |
| C | -6.898383 | -0.460354 | 0.485671 | -6.883659 | -0.462444 | 0.547609 | -6.868774 | -0.449279 | 0.611128 | -6.843848 | -0.451555 | 0.663689 | -6.863250 | -0.452637 | 0.618260 | -6.865357 | -0.452966 | 0.605256 |
| C | -5.967967 | -0.950216 | 1.383534 | -5.940426 | -0.882988 | 1.467988 | -5.915387 | -0.822375 | 1.542518 | -5.872066 | -0.830317 | 1.573805 | -5.904821 | -0.831173 | 1.542502 | -5.906334 | -0.857343 | 1.517152 |
| C | -4.638668 | -0.609769 | 1.220752 | -4.616356 | -0.543712 | 1.266948 | -4.594551 | -0.492167 | 1.308591 | -4.556468 | -0.501109 | 1.312028 | -4.585206 | -0.499233 | 1.303067 | -4.586013 | -0.523955 | 1.284199 |
| N | -8.316465 | -0.812993 | 0.662889 | -8.294032 | -0.813727 | 0.763202 | -8.272771 | -0.792069 | 0.859551 | -8.239646 | -0.788340 | 0.942305 | -8.266079 | -0.794721 | 0.872971 | -8.270246 | -0.795971 | 0.854376 |
| O | -8.602822 | -1.536449 | 1.591449 | -8.568795 | -1.480939 | 1.738027 | -8.537544 | -1.424421 | 1.862270 | -8.488320 | -1.433270 | 1.941361 | -8.525065 | -1.442301 | 1.867065 | -8.530696 | -1.446883 | 1.844803 |
| O | -9.110775 | -0.356997 | -0.130794 | -9.106316 | -0.416082 | -0.045450 | -9.101864 | -0.427085 | 0.050273 | -9.090468 | -0.407957 | 0.162847 | -9.100867 | -0.413638 | 0.077515 | -9.100438 | -0.410199 | 0.057440 |
| C | 4.272453 | -0.206204 | -0.006827 | 4.265479 | -0.200502 | 0.014672 | 4.256730 | -0.197469 | 0.032642 | 4.243043 | -0.198390 | 0.043571 | 4.254488 | -0.199784 | 0.032675 | 4.256372 | -0.205063 | 0.019637 |
| C | 4.949475 | -0.964714 | 0.942747 | 4.919520 | -0.944599 | 0.990724 | 4.901466 | -0.947739 | 1.009383 | 4.875452 | -0.956148 | 1.022268 | 4.893548 | -0.943596 | 1.018154 | 4.901828 | -0.957846 | 0.994701 |
| C | 6.277822 | -0.704964 | 1.218127 | 6.244397 | -0.686805 | 1.283196 | 6.221282 | -0.684428 | 1.319224 | 6.188809 | -0.689572 | 1.354456 | 6.212127 | -0.679140 | 1.332896 | 6.219679 | -0.691693 | 1.310597 |
| C | 6.907228 | 0.326470 | 0.545789 | 6.891788 | 0.327051 | 0.599818 | 6.869139 | 0.341020 | 0.652665 | 6.840991 | 0.344660 | 0.705182 | 6.864789 | 0.339789 | 0.660767 | 6.866830 | 0.338581 | 0.651112 |
| C | 6.247057 | 1.100998 | -0.390218 | 6.253517 | 1.086113 | -0.364693 | 6.239832 | 1.103876 | -0.315883 | 6.223972 | 1.114405 | -0.266111 | 6.241193 | 1.096241 | -0.316673 | 6.237409 | 1.104272 | -0.314575 |
| C | 4.921929 | 0.828114 | -0.670240 | 4.932056 | 0.814199 | -0.661362 | 4.924363 | 0.824770 | -0.631053 | 4.914350 | 0.833191 | -0.601636 | 4.926346 | 0.817157 | -0.635139 | 4.923335 | 0.823905 | -0.634595 |
| N | 8.321400 | 0.609126 | 0.837746 | 8.300066 | 0.605856 | 0.908961 | 8.267406 | 0.627709 | 0.983240 | 8.229568 | 0.633342 | 1.057692 | 8.262162 | 0.626446 | 0.995427 | 8.263902 | 0.629139 | 0.988247 |
| O | 8.877959 | -0.096330 | 1.651093 | 8.846259 | -0.090398 | 1.739089 | 8.827077 | -0.099440 | 1.779600 | 8.775030 | -0.076354 | 1.879640 | 8.809332 | -0.078959 | 1.819164 | 8.809640 | -0.075788 | 1.812206 |
| O | 8.843527 | 1.528952 | 0.246645 | 8.840891 | 1.515737 | 0.316421 | 8.798990 | 1.578326 | 0.444961 | 8.779854 | 1.570395 | 0.513875 | 8.806337 | 1.555296 | 0.432980 | 8.804430 | 1.559378 | 0.426379 |
| H | 0.740621 | 2.970668 | -0.766815 | 0.732316 | 2.961178 | -0.848986 | 0.729061 | 2.917991 | -0.979801 | 0.723821 | 2.903975 | -1.090047 | 0.728746 | 2.925777 | -0.983059 | 0.732238 | 2.937158 | -0.957525 |
| H | -0.488083 | 5.095978 | -0.775562 | -0.501212 | 5.083609 | -0.883857 | -0.502014 | 5.043565 | -1.048608 | -0.504300 | 5.031507 | -1.144912 | -0.502819 | 5.051596 | -1.038358 | -0.496130 | 5.063084 | -0.990584 |
| H | -2.913448 | 5.142129 | -0.331581 | -2.927362 | 5.129089 | -0.440131 | -2.922776 | 5.103600 | -0.574482 | -2.915106 | 5.099637 | -0.626697 | -2.922997 | 5.108848 | -0.559655 | -2.914770 | 5.119504 | -0.506607 |
| H | -4.154338 | 3.035000 | 0.045232 | -4.164698 | 3.025494 | -0.037381 | -4.162152 | 3.013852 | -0.111954 | -4.152819 | 3.015841 | -0.132836 | -4.160952 | 3.015894 | -0.105774 | -4.153454 | 3.023267 | -0.069478 |
| H | -0.795981 | -2.769066 | -1.106365 | -0.776931 | -2.750965 | -1.224510 | -0.754552 | -2.745229 | -1.330573 | -0.743590 | -2.725898 | -1.454298 | -0.755241 | -2.740591 | -1.353276 | -0.765385 | -2.753239 | -1.309689 |
| H | 4.148862 | -2.944797 | -0.729458 | 4.160755 | -2.929072 | -0.745113 | 4.178749 | -2.916219 | -0.773207 | 4.178198 | -2.916239 | -0.792276 | 4.173802 | -2.923551 | -0.761235 | 4.166779 | -2.934918 | -0.760932 |
| H | 0.402889 | -4.868008 | -1.523034 | 0.434203 | -4.841113 | -1.655707 | 0.473954 | -4.825814 | -1.781246 | 0.489818 | -4.804271 | -1.899599 | 0.470699 | -4.825593 | -1.791255 | 0.453034 | -4.842981 | -1.734760 |
| H | 2.857783 | -4.973285 | -1.313057 | 2.884639 | -4.946846 | -1.393910 | 2.920748 | -4.924831 | -1.480386 | 2.928580 | -4.913340 | -1.547984 | 2.915134 | -4.930975 | -1.472580 | 2.899998 | -4.948506 | -1.439456 |
| H | -4.895734 | 1.285282 | -1.577463 | -4.918815 | 1.160025 | -1.649289 | -4.934263 | 1.070777 | -1.683902 | -4.958632 | 1.068964 | -1.670654 | -4.941455 | 1.078841 | -1.679984 | -4.941337 | 1.125119 | -1.659073 |
| H | -3.892236 | -0.980194 | 1.907936 | -3.861202 | -0.854807 | 1.974415 | -3.832405 | -0.758245 | 2.027394 | -3.778013 | -0.770557 | 2.012365 | -3.818193 | -0.769397 | 2.015272 | -3.818486 | -0.819024 | 1.985629 |
| H | -7.292924 | 0.699674 | -1.264431 | -7.302757 | 0.579400 | -1.275168 | -7.307755 | 0.507910 | -1.258423 | -7.322432 | 0.508938 | -1.195964 | -7.313176 | 0.512410 | -1.244401 | -7.316434 | 0.554870 | -1.231570 |
| H | -6.289372 | -1.581791 | 2.198327 | -6.241779 | -1.459141 | 2.330154 | -6.200978 | -1.352279 | 2.439135 | -6.138078 | -1.361946 | 2.475571 | -6.186059 | -1.365381 | 2.438046 | -6.189782 | -1.414925 | 2.397532 |
| H | 4.429821 | -1.752022 | 1.471778 | 4.387279 | -1.718842 | 1.526771 | 4.369184 | -1.731822 | 1.530721 | 4.338688 | -1.747306 | 1.528182 | 4.357700 | -1.723433 | 1.542167 | 4.369388 | -1.744435 | 1.512199 |
| H | 4.386477 | 1.419452 | -1.397981 | 4.416673 | 1.387780 | -1.418005 | 4.417910 | 1.394584 | -1.397051 | 4.415768 | 1.408093 | -1.369489 | 4.423262 | 1.383076 | -1.406230 | 4.414484 | 1.401545 | -1.392816 |
| H | 6.822502 | -1.279982 | 1.952129 | 6.767355 | -1.252751 | 2.039701 | 6.734191 | -1.258057 | 2.076951 | 6.692440 | -1.267471 | 2.115348 | 6.720638 | -1.246856 | 2.098002 | 6.734395 | -1.264649 | 2.067660 |
| H | 6.772747 | 1.899017 | -0.893087 | 6.787735 | 1.870746 | -0.879582 | 6.772037 | 1.893951 | -0.824743 | 6.759084 | 1.911313 | -0.761130 | 6.776666 | 1.882050 | -0.828696 | 6.770211 | 1.900057 | -0.813694 |

| **iso-I** 11 |  | gasphase |  |  | toluene |  |  | DMSO |  |  | methanol |  |  | acetonitrile |  |  | chloroform |  |
| --- | --- | --- | --- | --- | --- | --- | --- | --- | --- | --- | --- | --- | --- | --- | --- | --- | --- | --- |
| Element | x | y | z | x | y | z | x | y | z | x | y | z | x | y | z | x | y | z |
| C | -0.014716 | 2.910575 | -0.976453 | -0.003089 | 2.902293 | -0.891658 | -0.007795 | 2.880814 | -0.971910 | -0.012533 | 2.873705 | -1.067124 | -0.006257 | 2.881018 | -0.992319 | 0.006529 | 2.907005 | -0.876232 |
| C | -0.731928 | 4.089888 | -1.117402 | -0.715633 | 4.079990 | -1.064995 | -0.721623 | 4.055110 | -1.164084 | -0.728486 | 4.047091 | -1.256372 | -0.719030 | 4.057074 | -1.178716 | -0.702594 | 4.085599 | -1.059494 |
| C | -2.107172 | 4.109470 | -0.948283 | -2.094596 | 4.102503 | -0.923377 | -2.100927 | 4.080107 | -1.008409 | -2.104320 | 4.076150 | -1.072025 | -2.097350 | 4.084609 | -1.013852 | -2.083108 | 4.111141 | -0.926720 |
| C | -2.801649 | 2.945611 | -0.645872 | -2.796199 | 2.943412 | -0.619248 | -2.801750 | 2.927780 | -0.676462 | -2.801758 | 2.928418 | -0.715899 | -2.798477 | 2.933069 | -0.678855 | -2.789609 | 2.954481 | -0.623267 |
| C | -2.074596 | 1.782953 | -0.500586 | -2.073382 | 1.781965 | -0.443677 | -2.076561 | 1.769973 | -0.483912 | -2.073762 | 1.773359 | -0.524471 | -2.074389 | 1.773609 | -0.492119 | -2.069869 | 1.792609 | -0.438032 |
| C | -0.679669 | 1.734347 | -0.645959 | -0.675570 | 1.731347 | -0.557308 | -0.680016 | 1.720468 | -0.602740 | -0.680583 | 1.721416 | -0.667512 | -0.678575 | 1.721813 | -0.619321 | -0.671332 | 1.739484 | -0.540158 |
| N | -2.576638 | 0.496301 | -0.278561 | -2.579435 | 0.498839 | -0.211504 | -2.577426 | 0.489197 | -0.229920 | -2.569238 | 0.490926 | -0.253652 | -2.575766 | 0.492671 | -0.237611 | -2.579015 | 0.510675 | -0.204892 |
| C | -1.558232 | -0.440862 | -0.335699 | -1.560309 | -0.435510 | -0.216004 | -1.557175 | -0.441110 | -0.218386 | -1.551408 | -0.434463 | -0.257453 | -1.557118 | -0.439037 | -0.235975 | -1.561360 | -0.422108 | -0.187468 |
| C | -0.269410 | 0.329744 | -0.531956 | -0.269027 | 0.328234 | -0.417971 | -0.271332 | 0.322985 | -0.444629 | -0.271596 | 0.326206 | -0.505948 | -0.271208 | 0.323558 | -0.464423 | -0.268870 | 0.336480 | -0.393488 |
| C | 0.951007 | -0.262976 | -0.510214 | 0.950315 | -0.266167 | -0.397988 | 0.951122 | -0.260206 | -0.420942 | 0.950534 | -0.255705 | -0.482422 | 0.950774 | -0.260812 | -0.439955 | 0.948253 | -0.261000 | -0.375058 |
| O | -1.727334 | -1.622839 | -0.171970 | -1.727360 | -1.611529 | -0.003878 | -1.712215 | -1.611900 | 0.037677 | -1.699009 | -1.607628 | 0.011418 | -1.713124 | -1.611263 | 0.014241 | -1.727927 | -1.594988 | 0.049046 |
| C | 1.365165 | -1.667274 | -0.612302 | 1.361458 | -1.669351 | -0.521894 | 1.366990 | -1.656859 | -0.567295 | 1.366099 | -1.649806 | -0.636260 | 1.365778 | -1.658012 | -0.583525 | 1.353325 | -1.664409 | -0.510844 |
| C | 2.754053 | -1.715530 | -0.416500 | 2.755252 | -1.718282 | -0.364566 | 2.758315 | -1.704397 | -0.395538 | 2.752564 | -1.701419 | -0.436987 | 2.756009 | -1.707343 | -0.402970 | 2.747314 | -1.719228 | -0.359308 |
| N | 3.247117 | -0.429805 | -0.172983 | 3.252724 | -0.435088 | -0.119155 | 3.246876 | -0.424527 | -0.118026 | 3.235344 | -0.421406 | -0.136770 | 3.244363 | -0.427342 | -0.123166 | 3.249270 | -0.439052 | -0.106437 |
| C | 2.232491 | 0.507146 | -0.264892 | 2.235215 | 0.498325 | -0.155617 | 2.227067 | 0.503995 | -0.140691 | 2.219705 | 0.502960 | -0.175221 | 2.226103 | 0.502184 | -0.155853 | 2.235351 | 0.495677 | -0.126233 |
| C | 0.713194 | -2.843296 | -0.968474 | 0.700902 | -2.842181 | -0.873022 | 0.711322 | -2.817952 | -0.962883 | 0.715983 | -2.800578 | -1.068421 | 0.710715 | -2.818041 | -0.983644 | 0.686929 | -2.830686 | -0.873241 |
| C | 1.435668 | -4.022121 | -1.084961 | 1.419882 | -4.019665 | -1.019946 | 1.434127 | -3.990852 | -1.129100 | 1.440289 | -3.972551 | -1.233898 | 1.432888 | -3.992151 | -1.145266 | 1.401248 | -4.009676 | -1.032674 |
| C | 2.803814 | -4.041388 | -0.865455 | 2.793735 | -4.040329 | -0.834510 | 2.806482 | -4.013886 | -0.920587 | 2.807311 | -4.001695 | -0.992633 | 2.803973 | -4.017105 | -0.927943 | 2.775977 | -4.037125 | -0.848938 |
| C | 3.486384 | -2.877732 | -0.536173 | 3.484515 | -2.879510 | -0.512438 | 3.492327 | -2.860842 | -0.560667 | 3.488704 | -2.855287 | -0.602429 | 3.489334 | -2.864867 | -0.563658 | 3.472344 | -2.881679 | -0.518533 |
| O | 2.395564 | 1.689147 | -0.094042 | 2.395252 | 1.674825 | 0.060403 | 2.370536 | 1.673929 | 0.126997 | 2.355415 | 1.675113 | 0.106337 | 2.369837 | 1.673499 | 0.106991 | 2.397072 | 1.669484 | 0.110831 |
| C | -3.913964 | 0.174806 | 0.035142 | -3.928591 | 0.178220 | 0.053493 | -3.923790 | 0.173678 | 0.055170 | -3.909801 | 0.174327 | 0.059911 | -3.920847 | 0.176705 | 0.054321 | -3.930407 | 0.189694 | 0.050643 |
| C | -4.568466 | 0.866680 | 1.049212 | -4.613600 | 0.852620 | 1.058413 | -4.591826 | 0.856274 | 1.065451 | -4.556967 | 0.859762 | 1.081497 | -4.583162 | 0.857246 | 1.069869 | -4.621720 | 0.859540 | 1.053918 |
| C | -5.879138 | 0.562497 | 1.361340 | -5.936040 | 0.549244 | 1.316989 | -5.910246 | 0.554467 | 1.344818 | -5.867616 | 0.554508 | 1.390007 | -5.899579 | 0.553630 | 1.357209 | -5.943626 | 0.548436 | 1.306156 |
| C | -6.513052 | -0.446525 | 0.659840 | -6.549280 | -0.441542 | 0.571250 | -6.533468 | -0.441605 | 0.613392 | -6.501998 | -0.446271 | 0.674094 | -6.526720 | -0.441420 | 0.627535 | -6.547856 | -0.444073 | 0.554756 |
| C | -5.874359 | -1.155817 | -0.340572 | -5.879289 | -1.133585 | -0.421666 | -5.880515 | -1.138351 | -0.388690 | -5.869891 | -1.145001 | -0.340152 | -5.879660 | -1.135815 | -0.380196 | -5.872423 | -1.128440 | -0.440474 |
| C | -4.567414 | -0.838179 | -0.656643 | -4.561259 | -0.815014 | -0.684434 | -4.566647 | -0.820841 | -0.672216 | -4.563318 | -0.824568 | -0.651554 | -4.567441 | -0.817228 | -0.670820 | -4.554893 | -0.802221 | -0.696165 |
| N | -7.908461 | -0.776195 | 0.990244 | -7.954968 | -0.768826 | 0.841972 | -7.930838 | -0.768606 | 0.908393 | -7.887650 | -0.776520 | 1.000412 | -7.921577 | -0.770760 | 0.931826 | -7.948995 | -0.783355 | 0.823290 |
| O | -8.434073 | -1.675998 | 0.372025 | -8.461252 | -1.666255 | 0.201720 | -8.443844 | -1.686602 | 0.299979 | -8.420542 | -1.687321 | 0.397844 | -8.445236 | -1.672870 | 0.309509 | -8.449890 | -1.684398 | 0.182720 |
| O | -8.447338 | -0.126563 | 1.860028 | -8.533836 | -0.122182 | 1.689811 | -8.508564 | -0.105298 | 1.746439 | -8.448242 | -0.126028 | 1.860299 | -8.487385 | -0.125650 | 1.791533 | -8.537154 | -0.145682 | 1.672371 |
| C | 4.574974 | -0.108701 | 0.183258 | 4.595713 | -0.112740 | 0.179671 | 4.586604 | -0.106582 | 0.201167 | 4.568177 | -0.106141 | 0.218179 | 4.582115 | -0.108873 | 0.204669 | 4.596159 | -0.120930 | 0.182238 |
| C | 5.192210 | -0.789313 | 1.226681 | 5.246168 | -0.764095 | 1.220926 | 5.220639 | -0.768683 | 1.245531 | 5.174947 | -0.777815 | 1.272150 | 5.210975 | -0.772785 | 1.251169 | 5.249906 | -0.768582 | 1.223458 |
| C | 6.492304 | -0.484248 | 1.578104 | 6.560398 | -0.456234 | 1.512555 | 6.531357 | -0.464317 | 1.556328 | 6.477402 | -0.475693 | 1.617890 | 6.519615 | -0.467480 | 1.570363 | 6.566805 | -0.462031 | 1.505043 |
| C | 7.177632 | 0.518131 | 0.895901 | 7.221867 | 0.518622 | 0.769040 | 7.200343 | 0.515647 | 0.825887 | 7.163475 | 0.511060 | 0.912902 | 7.191655 | 0.515504 | 0.846547 | 7.224460 | 0.506699 | 0.749821 |
| C | 6.551575 | 1.209260 | -0.138660 | 6.564324 | 1.179658 | -0.266162 | 6.561011 | 1.185190 | -0.215786 | 6.550637 | 1.189926 | -0.138282 | 6.557190 | 1.187263 | -0.196875 | 6.564420 | 1.162839 | -0.287255 |
| C | 5.255773 | 0.893094 | -0.496798 | 5.254371 | 0.859013 | -0.562699 | 5.254469 | 0.867479 | -0.530289 | 5.252481 | 0.874419 | -0.488474 | 5.252827 | 0.868567 | -0.519948 | 5.251729 | 0.843069 | -0.573036 |
| C | 8.525700 | 0.838740 | 1.260060 | 8.583819 | 0.842599 | 1.070201 | 8.557067 | 0.836473 | 1.147026 | 8.511210 | 0.829462 | 1.270665 | 8.546349 | 0.837133 | 1.176412 | 8.588141 | 0.830076 | 1.041124 |
| N | 9.607206 | 1.093492 | 1.551261 | 9.677047 | 1.100463 | 1.310308 | 9.647067 | 1.093725 | 1.404387 | 9.594334 | 1.084708 | 1.557605 | 9.634625 | 1.094862 | 1.440741 | 9.683241 | 1.088169 | 1.273675 |
| H | 1.052866 | 2.910430 | -1.106888 | 1.067106 | 2.900335 | -1.001938 | 1.061258 | 2.873720 | -1.101288 | 1.052415 | 2.860406 | -1.231503 | 1.061822 | 2.871204 | -1.129735 | 1.077481 | 2.901854 | -0.982953 |
| H | -0.206123 | 5.002594 | -1.363585 | -0.183960 | 4.988962 | -1.313803 | -0.193931 | 4.958698 | -1.440858 | -0.205971 | 4.946389 | -1.556108 | -0.191431 | 4.959832 | -1.458282 | -0.167925 | 4.992647 | -1.309642 |
| H | -2.653244 | 5.036957 | -1.063917 | -2.637420 | 5.028984 | -1.061965 | -2.644822 | 5.004015 | -1.160836 | -2.649312 | 4.999557 | -1.223939 | -2.640382 | 5.009744 | -1.161720 | -2.623185 | 5.038260 | -1.072442 |
| H | -3.878066 | 2.949917 | -0.547238 | -3.874289 | 2.952210 | -0.540223 | -3.879312 | 2.939801 | -0.587753 | -3.877803 | 2.942527 | -0.609645 | -3.875377 | 2.946952 | -0.583264 | -3.868242 | 2.966251 | -0.550958 |
| H | -0.348942 | -2.843677 | -1.137787 | -0.365378 | -2.842116 | -1.016646 | -0.352012 | -2.812731 | -1.132939 | -0.341383 | -2.787565 | -1.276006 | -0.351514 | -2.810504 | -1.160923 | -0.379446 | -2.825036 | -1.019200 |
| H | 4.558412 | -2.881325 | -0.397641 | 4.559455 | -2.886279 | -0.397854 | 4.565539 | -2.870810 | -0.428791 | 4.559279 | -2.868921 | -0.450133 | 4.561633 | -2.876258 | -0.425025 | 4.547469 | -2.894101 | -0.405354 |
| H | 0.919636 | -4.934662 | -1.351549 | 0.897152 | -4.930064 | -1.282099 | 0.918865 | -4.894993 | -1.426690 | 0.931394 | -4.870766 | -1.559233 | 0.918287 | -4.895513 | -1.446280 | 0.875120 | -4.915711 | -1.303698 |
| H | 3.354025 | -4.968609 | -0.962205 | 3.341246 | -4.966940 | -0.952281 | 3.357074 | -4.936966 | -1.052606 | 3.358573 | -4.924299 | -1.125683 | 3.354088 | -4.940930 | -1.056564 | 3.319696 | -4.964922 | -0.975568 |
| H | -4.044717 | 1.636686 | 1.599246 | -4.107565 | 1.608586 | 1.643386 | -4.078787 | 1.617700 | 1.637122 | -4.033929 | 1.624790 | 1.639238 | -4.067170 | 1.618340 | 1.639267 | -4.122056 | 1.616643 | 1.642906 |
| H | -4.049497 | -1.377990 | -1.435329 | -4.022214 | -1.335851 | -1.462406 | -4.042528 | -1.338846 | -1.462683 | -4.053173 | -1.343090 | -1.451270 | -4.046973 | -1.334340 | -1.464258 | -4.010507 | -1.314738 | -1.476452 |
| H | -6.406109 | 1.085807 | 2.145260 | -6.482426 | 1.062163 | 2.094539 | -6.440473 | 1.075926 | 2.127933 | -6.382061 | 1.076554 | 2.183274 | -6.425366 | 1.072913 | 2.144734 | -6.493518 | 1.057246 | 2.083988 |
| H | -6.402649 | -1.938417 | -0.864492 | -6.387500 | -1.902531 | -0.984237 | -6.394008 | -1.907326 | -0.946705 | -6.391886 | -1.917277 | -0.885716 | -6.395848 | -1.904555 | -0.936056 | -6.373236 | -1.896684 | -1.010859 |
| H | 4.647697 | -1.551972 | 1.767077 | 4.719319 | -1.506139 | 1.805818 | 4.686992 | -1.517342 | 1.815716 | 4.627235 | -1.531708 | 1.821996 | 4.675116 | -1.523727 | 1.816196 | 4.724906 | -1.506450 | 1.815341 |
| H | 4.765755 | 1.425995 | -1.298351 | 4.739501 | 1.362078 | -1.368740 | 4.753920 | 1.369309 | -1.346491 | 4.771595 | 1.383052 | -1.312729 | 4.755684 | 1.372502 | -1.336927 | 4.733474 | 1.338600 | -1.382016 |
| H | 6.976502 | -1.011104 | 2.389017 | 7.072027 | -0.959606 | 2.321694 | 7.032643 | -0.975274 | 2.367192 | 6.959035 | -0.993490 | 2.436436 | 7.016853 | -0.979968 | 2.382741 | 7.082630 | -0.961465 | 2.314023 |
| H | 7.086246 | 1.988926 | -0.663779 | 7.082523 | 1.936564 | -0.839179 | 7.089102 | 1.941832 | -0.780217 | 7.092616 | 1.952151 | -0.681881 | 7.087405 | 1.946495 | -0.755791 | 7.081697 | 1.913365 | -0.869547 |

| **iso-I** 12 |  | gasphase |  |  | toluene |  |  | DMSO |  |  | methanol |  |  | acetonitrile |  |  | chloroform |  |
| --- | --- | --- | --- | --- | --- | --- | --- | --- | --- | --- | --- | --- | --- | --- | --- | --- | --- | --- |
| Element | x | y | z | x | y | z | x | y | z | x | y | z | x | y | z | x | y | z |
| C | 0.096952 | -2.892693 | -0.872848 | 0.098039 | -2.889604 | -0.813937 | 0.104700 | -2.880576 | -0.855024 | 0.098156 | -2.856853 | -0.994468 | 0.100460 | -2.878821 | -0.873226 | 0.094527 | -2.893318 | -0.835650 |
| C | 0.792968 | -4.085154 | -1.013167 | 0.793043 | -4.079047 | -0.980965 | 0.802311 | -4.065400 | -1.044222 | 0.793788 | -4.043187 | -1.180034 | 0.795928 | -4.066005 | -1.056666 | 0.788533 | -4.082571 | -1.009820 |
| C | 2.171487 | -4.123929 | -0.879478 | 2.173926 | -4.117003 | -0.865029 | 2.184576 | -4.101750 | -0.923352 | 2.171531 | -4.090672 | -1.016237 | 2.177582 | -4.105867 | -0.928581 | 2.169877 | -4.122368 | -0.890805 |
| C | 2.889980 | -2.966123 | -0.612177 | 2.894813 | -2.961706 | -0.593162 | 2.903985 | -2.950617 | -0.629007 | 2.890798 | -2.948863 | -0.685337 | 2.898707 | -2.955743 | -0.633562 | 2.892235 | -2.969590 | -0.610515 |
| C | 2.183924 | -1.790388 | -0.465845 | 2.189315 | -1.788524 | -0.423218 | 2.194978 | -1.782313 | -0.439023 | 2.182914 | -1.780684 | -0.497478 | 2.191827 | -1.785137 | -0.449839 | 2.187369 | -1.797040 | -0.433923 |
| C | 0.787033 | -1.721645 | -0.578423 | 0.790581 | -1.721944 | -0.511627 | 0.796367 | -1.720040 | -0.524540 | 0.788543 | -1.709711 | -0.619144 | 0.793688 | -1.719462 | -0.541194 | 0.788781 | -1.729420 | -0.522337 |
| N | 2.710289 | -0.508310 | -0.275257 | 2.714756 | -0.507694 | -0.223218 | 2.714529 | -0.503000 | -0.217665 | 2.701165 | -0.502313 | -0.250876 | 2.713713 | -0.506023 | -0.230678 | 2.712682 | -0.517130 | -0.225763 |
| C | 1.703260 | 0.444202 | -0.322339 | 1.705831 | 0.439795 | -0.227375 | 1.703385 | 0.437370 | -0.191795 | 1.696163 | 0.437615 | -0.247549 | 1.705054 | 0.436626 | -0.213145 | 1.704662 | 0.428016 | -0.215794 |
| C | 0.400290 | -0.307578 | -0.475603 | 0.403052 | -0.310634 | -0.386490 | 0.404882 | -0.314879 | -0.377840 | 0.401785 | -0.306229 | -0.466121 | 0.405132 | -0.312909 | -0.397544 | 0.402707 | -0.319461 | -0.389903 |
| C | -0.810985 | 0.303110 | -0.438590 | -0.809433 | 0.296641 | -0.347156 | -0.810256 | 0.283180 | -0.340565 | -0.811271 | 0.293683 | -0.429137 | -0.808921 | 0.287370 | -0.357174 | -0.808529 | 0.288855 | -0.355457 |
| O | 1.898584 | 1.625558 | -0.181838 | 1.895057 | 1.617754 | -0.044749 | 1.878787 | 1.609630 | 0.044999 | 1.866711 | 1.611439 | 0.005504 | 1.882996 | 1.610247 | 0.016295 | 1.889342 | 1.603560 | -0.006142 |
| C | -1.207567 | 1.712376 | -0.546268 | -1.208891 | 1.703233 | -0.475218 | -1.211029 | 1.685680 | -0.487888 | -1.208162 | 1.694187 | -0.583898 | -1.209737 | 1.690338 | -0.501533 | -1.202937 | 1.695375 | -0.493123 |
| C | -2.595774 | 1.773314 | -0.343176 | -2.600308 | 1.760108 | -0.295008 | -2.600719 | 1.743722 | -0.301418 | -2.591991 | 1.761513 | -0.367739 | -2.598945 | 1.748536 | -0.310323 | -2.593262 | 1.758490 | -0.309600 |
| N | -3.102163 | 0.500065 | -0.089527 | -3.104104 | 0.488443 | -0.031929 | -3.099575 | 0.475002 | -0.016589 | -3.087738 | 0.493154 | -0.057361 | -3.097071 | 0.479121 | -0.024454 | -3.099675 | 0.489232 | -0.036578 |
| C | -2.106609 | -0.447491 | -0.171251 | -2.103773 | -0.452499 | -0.071619 | -2.099030 | -0.463155 | -0.043376 | -2.092865 | -0.442806 | -0.101791 | -2.096604 | -0.458198 | -0.055730 | -2.103175 | -0.453199 | -0.072298 |
| C | -0.547051 | 2.885090 | -0.898182 | -0.548571 | 2.874508 | -0.832323 | -0.552554 | 2.846191 | -0.880487 | -0.551214 | 2.839938 | -1.018526 | -0.551917 | 2.851448 | -0.894053 | -0.538823 | 2.858905 | -0.867976 |
| C | -1.260109 | 4.071392 | -1.002641 | -1.264948 | 4.056542 | -0.960961 | -1.269882 | 4.026213 | -1.026797 | -1.265171 | 4.021429 | -1.167288 | -1.269490 | 4.031984 | -1.036659 | -1.251078 | 4.042783 | -1.005513 |
| C | -2.626277 | 4.102661 | -0.769807 | -2.634781 | 4.084823 | -0.745283 | -2.638629 | 4.059393 | -0.795423 | -2.627533 | 4.066465 | -0.902202 | -2.637734 | 4.064981 | -0.801806 | -2.620247 | 4.078844 | -0.782132 |
| C | -3.317819 | 2.943703 | -0.442631 | -3.326164 | 2.926484 | -0.415194 | -3.328293 | 2.907819 | -0.436938 | -3.316711 | 2.925553 | -0.508240 | -3.326665 | 2.912880 | -0.442717 | -3.315377 | 2.926430 | -0.437815 |
| O | -2.282150 | -1.627356 | 0.009077 | -2.270875 | -1.627765 | 0.152046 | -2.257613 | -1.634481 | 0.217241 | -2.246029 | -1.615840 | 0.177365 | -2.253818 | -1.630172 | 0.204592 | -2.271377 | -1.627491 | 0.165166 |
| C | 4.056782 | -0.202389 | 0.007879 | 4.070000 | -0.199503 | 0.020629 | 4.070499 | -0.195527 | 0.027999 | 4.050888 | -0.200789 | 0.036645 | 4.069010 | -0.200010 | 0.022479 | 4.067946 | -0.208379 | 0.021059 |
| C | 4.706928 | 0.803025 | -0.698763 | 4.704462 | 0.780742 | -0.733478 | 4.702218 | 0.783814 | -0.729275 | 4.705259 | 0.782992 | -0.694991 | 4.708782 | 0.772225 | -0.737342 | 4.700464 | 0.771450 | -0.735186 |
| C | 6.023902 | 1.106092 | -0.411921 | 6.028878 | 1.087542 | -0.489694 | 6.025652 | 1.093809 | -0.484969 | 6.020979 | 1.089129 | -0.408797 | 6.030613 | 1.082602 | -0.483724 | 6.022209 | 1.085881 | -0.486549 |
| C | 6.677045 | 0.390755 | 0.574945 | 6.703946 | 0.397937 | 0.501600 | 6.699345 | 0.405529 | 0.509200 | 6.661443 | 0.391552 | 0.601010 | 6.693991 | 0.401954 | 0.522690 | 6.694393 | 0.402287 | 0.511464 |
| C | 6.047014 | -0.610341 | 1.291331 | 6.089330 | -0.579788 | 1.263466 | 6.087433 | -0.575082 | 1.270487 | 6.026393 | -0.594116 | 1.337007 | 6.074112 | -0.571814 | 1.286491 | 6.082352 | -0.578231 | 1.272407 |
| C | 4.726660 | -0.900108 | 1.008337 | 4.761051 | -0.871909 | 1.023227 | 4.759726 | -0.869669 | 1.029798 | 4.706855 | -0.884926 | 1.053580 | 4.748411 | -0.867359 | 1.035582 | 4.756542 | -0.877461 | 1.027129 |
| N | 8.082425 | 0.704951 | 0.874089 | 8.115510 | 0.713318 | 0.752733 | 8.106842 | 0.724456 | 0.761787 | 8.056587 | 0.706561 | 0.900362 | 8.098416 | 0.723583 | 0.788505 | 8.099520 | 0.729045 | 0.772675 |
| O | 8.605407 | 1.598222 | 0.243642 | 8.625476 | 1.595035 | 0.093579 | 8.613950 | 1.621708 | 0.118402 | 8.590984 | 1.604332 | 0.279756 | 8.618386 | 1.602587 | 0.130917 | 8.607161 | 1.621479 | 0.125201 |
| O | 8.633994 | 0.050504 | 1.732750 | 8.696376 | 0.073516 | 1.604637 | 8.698538 | 0.075650 | 1.601456 | 8.623400 | 0.057145 | 1.757012 | 8.675478 | 0.095760 | 1.653560 | 8.684888 | 0.090337 | 1.623098 |
| C | -4.452214 | 0.205979 | 0.232771 | -4.464691 | 0.191724 | 0.249656 | -4.460805 | 0.181380 | 0.268403 | -4.438539 | 0.199968 | 0.275203 | -4.456481 | 0.183622 | 0.268908 | -4.459941 | 0.196596 | 0.253297 |
| C | -5.200515 | -0.628397 | -0.575182 | -5.221563 | -0.531132 | -0.652773 | -5.239950 | -0.476399 | -0.665484 | -5.211110 | -0.568214 | -0.576599 | -5.238652 | -0.481388 | -0.657633 | -5.225094 | -0.520033 | -0.647853 |
| C | -6.518443 | -0.922994 | -0.256546 | -6.550858 | -0.823946 | -0.383474 | -6.570624 | -0.764857 | -0.396277 | -6.528880 | -0.863721 | -0.257221 | -6.567348 | -0.771590 | -0.380041 | -6.553460 | -0.810611 | -0.370347 |
| C | -7.096141 | -0.362900 | 0.877585 | -7.129437 | -0.373538 | 0.799185 | -7.126662 | -0.375700 | 0.819976 | -7.075606 | -0.369663 | 0.923086 | -7.117950 | -0.376992 | 0.836844 | -7.121320 | -0.364284 | 0.819407 |
| C | -6.340497 | 0.481994 | 1.690185 | -6.364278 | 0.360958 | 1.706119 | -6.338083 | 0.294668 | 1.757716 | -6.295544 | 0.410853 | 1.777289 | -6.326278 | 0.299275 | 1.767718 | -6.347690 | 0.363954 | 1.724738 |
| C | -5.027525 | 0.754763 | 1.374175 | -5.040826 | 0.634022 | 1.435345 | -5.013815 | 0.566109 | 1.484530 | -4.982981 | 0.687921 | 1.457578 | -5.003910 | 0.572349 | 1.486458 | -5.025258 | 0.635550 | 1.445431 |
| O | -8.369891 | -0.577517 | 1.272390 | -8.411504 | -0.596157 | 1.152864 | -8.406373 | -0.600564 | 1.176201 | -8.347749 | -0.592937 | 1.322914 | -8.396005 | -0.601902 | 1.200652 | -8.401673 | -0.585600 | 1.181461 |
| C | -9.177577 | -1.431614 | 0.496027 | -9.230333 | -1.344046 | 0.279090 | -9.249915 | -1.277591 | 0.260490 | -9.188226 | -1.380025 | 0.493885 | -9.244990 | -1.284799 | 0.294221 | -9.232661 | -1.323538 | 0.306184 |
| H | -0.973788 | -2.876631 | -0.974079 | -0.974158 | -2.874518 | -0.902885 | -0.967358 | -2.863762 | -0.954100 | -0.968831 | -2.829240 | -1.142212 | -0.971018 | -2.859346 | -0.978182 | -0.977524 | -2.875783 | -0.930777 |
| H | 0.246835 | -4.993240 | -1.231041 | 0.245414 | -4.985150 | -1.204775 | 0.258879 | -4.968417 | -1.291042 | 0.253370 | -4.938107 | -1.460648 | 0.251281 | -4.967994 | -1.304489 | 0.240910 | -4.986675 | -1.242290 |
| H | 2.700825 | -5.061144 | -0.994928 | 2.702929 | -5.052222 | -0.998635 | 2.715897 | -5.033423 | -1.072974 | 2.700751 | -5.023784 | -1.164636 | 2.707106 | -5.039314 | -1.073333 | 2.698138 | -5.057386 | -1.029467 |
| H | 3.968427 | -2.985060 | -0.540870 | 3.974153 | -2.982457 | -0.534964 | 3.983281 | -2.972168 | -0.566946 | 3.968050 | -2.978088 | -0.595778 | 3.977602 | -2.979710 | -0.566150 | 3.971480 | -2.992638 | -0.549858 |
| H | 0.514136 | 2.877664 | -1.072684 | 0.515204 | 2.870605 | -0.993482 | 0.508751 | 2.836854 | -1.061903 | 0.503177 | 2.817674 | -1.239777 | 0.508967 | 2.841624 | -1.078310 | 0.523873 | 2.848724 | -1.039130 |
| H | -4.387339 | 2.951347 | -0.283914 | -4.397529 | 2.932725 | -0.266982 | -4.398672 | 2.918982 | -0.279721 | -4.383767 | 2.947068 | -0.330907 | -4.396552 | 2.923965 | -0.282446 | -4.386237 | 2.939393 | -0.285296 |
| H | -0.737269 | 4.980912 | -1.266636 | -0.742305 | 4.965485 | -1.228659 | -0.751559 | 4.929123 | -1.323046 | -0.750517 | 4.915493 | -1.495158 | -0.751729 | 4.935230 | -1.332813 | -0.726742 | 4.946790 | -1.286902 |
| H | -3.166752 | 5.037200 | -0.852043 | -3.177468 | 5.016780 | -0.843177 | -3.181881 | 4.989696 | -0.906511 | -3.168086 | 4.997826 | -1.017848 | -3.181224 | 4.995429 | -0.910364 | -3.159555 | 5.012145 | -0.886543 |
| H | 4.177609 | 1.348512 | -1.465553 | 4.161491 | 1.300940 | -1.509012 | 4.161984 | 1.296100 | -1.512532 | 4.188470 | 1.300954 | -1.490770 | 4.175760 | 1.279027 | -1.529088 | 4.158718 | 1.283999 | -1.517140 |
| H | 4.206573 | -1.663171 | 1.571224 | 4.254441 | -1.617209 | 1.621181 | 4.255945 | -1.619185 | 1.624937 | 4.183615 | -1.637493 | 1.627810 | 4.238187 | -1.611877 | 1.631497 | 4.251329 | -1.624614 | 1.623999 |
| H | 6.549002 | 1.882389 | -0.948263 | 6.538076 | 1.845817 | -1.065735 | 6.530345 | 1.850704 | -1.066947 | 6.543509 | 1.849446 | -0.970438 | 6.541438 | 1.834700 | -1.066625 | 6.528649 | 1.844640 | -1.064640 |
| H | 6.584803 | -1.138594 | 2.064485 | 6.639544 | -1.091213 | 2.039294 | 6.633684 | -1.090542 | 2.046507 | 6.547261 | -1.115561 | 2.126465 | 6.612834 | -1.080669 | 2.072072 | 6.629529 | -1.086715 | 2.052377 |
| H | -4.749367 | -1.063898 | -1.456444 | -4.770044 | -0.875819 | -1.574107 | -4.807013 | -0.769323 | -1.613894 | -4.783723 | -0.942926 | -1.498332 | -4.809452 | -0.778750 | -1.606347 | -4.781835 | -0.860421 | -1.575089 |
| H | -4.436966 | 1.398464 | 2.013816 | -4.444135 | 1.193906 | 2.144613 | -4.401142 | 1.081223 | 2.214155 | -4.374822 | 1.286857 | 2.124165 | -4.388621 | 1.092042 | 2.210569 | -4.422384 | 1.191782 | 2.152487 |
| H | -7.078727 | -1.584636 | -0.900759 | -7.119492 | -1.396438 | -1.101843 | -7.157699 | -1.283617 | -1.140435 | -7.112461 | -1.469695 | -0.935179 | -7.157584 | -1.295747 | -1.117852 | -7.129698 | -1.377434 | -1.087266 |
| H | -6.801216 | 0.903453 | 2.573486 | -6.825146 | 0.700280 | 2.624528 | -6.779743 | 0.591876 | 2.700400 | -6.733082 | 0.787035 | 2.693479 | -6.764112 | 0.599393 | 2.711260 | -6.800234 | 0.701348 | 2.648307 |
| H | -8.761458 | -2.442629 | 0.449778 | -8.840421 | -2.355658 | 0.131946 | -8.877698 | -2.282825 | 0.044704 | -8.789718 | -2.390650 | 0.373429 | -8.872746 | -2.290745 | 0.082223 | -8.848240 | -2.335480 | 0.150227 |
| H | -9.306272 | -1.046214 | -0.520146 | -9.338771 | -0.849439 | -0.690955 | -9.354137 | -0.718441 | -0.673411 | -9.321659 | -0.916395 | -0.486988 | -9.354748 | -0.730696 | -0.641969 | -9.342586 | -0.820078 | -0.658636 |
| H | -10.145177 | -1.465222 | 0.990874 | -10.205621 | -1.405341 | 0.757535 | -10.221598 | -1.351563 | 0.744240 | -10.148317 | -1.429389 | 1.003117 | -10.213144 | -1.356068 | 0.785211 | -10.204479 | -1.381366 | 0.791902 |

| **iso-I** 13 |  | gasphase |  |  | toluene |  |  | DMSO |  |  | methanol |  |  | acetonitrile |  |  | chloroform |  |
| --- | --- | --- | --- | --- | --- | --- | --- | --- | --- | --- | --- | --- | --- | --- | --- | --- | --- | --- |
| Element | x | y | z | x | y | z | x | y | z | x | y | z | x | y | z | x | y | z |
| C | 0.129904 | 3.120699 | -0.038027 | 0.046728 | 3.019605 | -0.118257 | 0.039403 | 3.006156 | -0.062030 | 0.022222 | -2.963854 | -0.017813 | 0.020625 | 2.993147 | -0.070989 | 0.017545 | 3.001232 | -0.122388 |
| C | 0.884051 | 4.261931 | 0.197742 | 0.759705 | 4.188703 | 0.107405 | 0.746250 | 4.173140 | 0.193435 | -0.672016 | -4.141353 | 0.222688 | 0.723333 | 4.165376 | 0.172583 | 0.725035 | 4.172868 | 0.109012 |
| C | 2.256777 | 4.184603 | 0.367903 | 2.134411 | 4.159475 | 0.284128 | 2.123387 | 4.145497 | 0.364328 | -2.052341 | -4.133835 | 0.369664 | 2.101555 | 4.145511 | 0.336389 | 2.101054 | 4.149222 | 0.282283 |
| C | 2.909415 | 2.959679 | 0.317890 | 2.828545 | 2.956888 | 0.254304 | 2.824764 | 2.947932 | 0.303097 | -2.771109 | -2.946383 | 0.302884 | 2.808329 | 2.950650 | 0.280134 | 2.801689 | 2.950197 | 0.245549 |
| C | 2.148810 | 1.835785 | 0.073387 | 2.106982 | 1.804604 | 0.021939 | 2.108097 | 1.798141 | 0.042882 | -2.066709 | -1.787340 | 0.055539 | 2.095781 | 1.795704 | 0.031574 | 2.085041 | 1.795866 | 0.008166 |
| C | 0.760072 | 1.883742 | -0.122384 | 0.719280 | 1.804071 | -0.184446 | 0.720958 | 1.798196 | -0.165189 | -0.678213 | -1.768362 | -0.134782 | 0.707572 | 1.787641 | -0.169539 | 0.697557 | 1.790246 | -0.198468 |
| N | 2.586284 | 0.506948 | 0.062261 | 2.587086 | 0.490993 | 0.030364 | 2.591806 | 0.486362 | 0.018309 | -2.564092 | -0.477311 | 0.037111 | 2.585313 | 0.485591 | 0.015001 | 2.570231 | 0.483772 | 0.011078 |
| C | 1.512062 | -0.358950 | -0.071534 | 1.546338 | -0.410471 | -0.107304 | 1.558647 | -0.412817 | -0.156952 | -1.540203 | 0.429688 | -0.110948 | 1.555144 | -0.419188 | -0.145467 | 1.535577 | -0.419868 | -0.135255 |
| C | 0.280440 | 0.501345 | -0.251696 | 0.285935 | 0.405527 | -0.290466 | 0.293235 | 0.403268 | -0.300940 | -0.267896 | -0.369180 | -0.254955 | 0.285975 | 0.389539 | -0.294114 | 0.271294 | 0.390945 | -0.309096 |
| C | -0.937979 | -0.015691 | -0.549010 | -0.920246 | -0.151900 | -0.561552 | -0.915054 | -0.148692 | -0.565410 | 0.931304 | 0.198591 | -0.522788 | -0.919270 | -0.170408 | -0.556465 | -0.932731 | -0.170584 | -0.577519 |
| O | 1.623415 | -1.558735 | -0.103879 | 1.698528 | -1.606753 | -0.152561 | 1.713959 | -1.605731 | -0.273710 | -1.703521 | 1.626679 | -0.217784 | 1.714856 | -1.613216 | -0.246681 | 1.691598 | -1.616214 | -0.199523 |
| C | -1.464410 | -1.386675 | -0.499689 | -1.416731 | -1.527819 | -0.433650 | -1.410260 | -1.520523 | -0.417437 | 1.417052 | 1.567445 | -0.343667 | -1.407977 | -1.544505 | -0.405891 | -1.425285 | -1.545369 | -0.434217 |
| C | -2.753949 | -1.357033 | -1.054123 | -2.723228 | -1.547970 | -0.947839 | -2.717808 | -1.547495 | -0.926367 | 2.705359 | 1.626401 | -0.893540 | -2.713601 | -1.579954 | -0.919627 | -2.730204 | -1.574909 | -0.950730 |
| N | -3.064734 | -0.071865 | -1.476375 | -3.067003 | -0.291339 | -1.426914 | -3.061113 | -0.296595 | -1.423045 | 3.038224 | 0.394905 | -1.454178 | -3.060592 | -0.332418 | -1.423737 | -3.073387 | -0.325291 | -1.451521 |
| C | -2.074148 | 0.812022 | -1.127415 | -2.075845 | 0.620106 | -1.174937 | -2.068533 | 0.615807 | -1.187859 | 2.070471 | -0.533507 | -1.198344 | -2.075402 | 0.585950 | -1.182906 | -2.087793 | 0.590958 | -1.201119 |
| C | -1.019364 | -2.583794 | 0.049653 | -0.931992 | -2.686277 | 0.162527 | -0.925414 | -2.661508 | 0.211347 | 0.944025 | 2.672235 | 0.354363 | -0.918878 | -2.681540 | 0.227135 | -0.940684 | -2.690985 | 0.186628 |
| C | -1.837161 | -3.705910 | 0.006782 | -1.727693 | -3.824072 | 0.201502 | -1.721435 | -3.797918 | 0.277600 | 1.732723 | 3.811821 | 0.442460 | -1.709362 | -3.821995 | 0.294111 | -1.735629 | -3.828698 | 0.244464 |
| C | -3.094442 | -3.650377 | -0.573393 | -3.003791 | -3.819676 | -0.340573 | -2.998562 | -3.804593 | -0.266860 | 2.990687 | 3.852830 | -0.143913 | -2.985146 | -3.836458 | -0.253514 | -3.011157 | -3.834692 | -0.301174 |
| C | -3.575819 | -2.462419 | -1.109688 | -3.525474 | -2.669133 | -0.919311 | -3.521710 | -2.667479 | -0.870876 | 3.503247 | 2.747768 | -0.813850 | -3.512136 | -2.703581 | -0.862622 | -3.532845 | -2.695304 | -0.902190 |
| O | -2.144459 | 2.001882 | -1.322548 | -2.149584 | 1.790144 | -1.472761 | -2.131453 | 1.781629 | -1.512019 | 2.135272 | -1.695778 | -1.548496 | -2.146039 | 1.752357 | -1.504516 | -2.162059 | 1.759990 | -1.508541 |
| C | 3.928946 | 0.079176 | 0.110727 | 3.944053 | 0.109345 | 0.096050 | 3.951018 | 0.109222 | 0.082818 | -3.928641 | -0.114108 | 0.080186 | 3.946142 | 0.113617 | 0.079367 | 3.929326 | 0.108090 | 0.081808 |
| C | 4.311756 | -0.916147 | 1.002523 | 4.356424 | -0.824735 | 1.038887 | 4.361928 | -0.825365 | 1.025636 | -4.363554 | 0.813723 | 1.018829 | 4.361974 | -0.813024 | 1.028123 | 4.342463 | -0.812342 | 1.037560 |
| C | 5.626581 | -1.338676 | 1.040536 | 5.682950 | -1.205574 | 1.096081 | 5.688858 | -1.204148 | 1.083986 | -5.695280 | 1.177051 | 1.057487 | 5.690686 | -1.185772 | 1.087873 | 5.670693 | -1.185890 | 1.103270 |
| C | 6.544272 | -0.750903 | 0.189509 | 6.580957 | -0.634857 | 0.211919 | 6.585777 | -0.628394 | 0.200787 | -6.570392 | 0.591314 | 0.158923 | 6.584103 | -0.612316 | 0.199411 | 6.567177 | -0.620475 | 0.213595 |
| C | 6.182795 | 0.238114 | -0.706742 | 6.189368 | 0.296762 | -0.732983 | 6.194967 | 0.307152 | -0.741339 | -6.155074 | -0.336484 | -0.780895 | 6.188234 | 0.314294 | -0.749592 | 6.174306 | 0.297594 | -0.744496 |
| C | 4.864254 | 0.647245 | -0.748642 | 4.859242 | 0.663358 | -0.792912 | 4.864151 | 0.671318 | -0.802178 | -4.819697 | -0.684288 | -0.821907 | 4.855752 | 0.672579 | -0.811432 | 4.842524 | 0.656978 | -0.812001 |
| N | 7.946609 | -1.192078 | 0.236605 | 7.993164 | -1.031286 | 0.276743 | 7.995547 | -1.022932 | 0.265155 | -7.982559 | 0.963747 | 0.205387 | 7.995943 | -0.998289 | 0.266169 | 7.979658 | -1.006277 | 0.288948 |
| O | 8.232511 | -2.072224 | 1.018982 | 8.309905 | -1.868921 | 1.095192 | 8.315938 | -1.869352 | 1.075710 | -8.327368 | 1.819938 | 0.995687 | 8.322724 | -1.833294 | 1.085503 | 8.301444 | -1.832281 | 1.118062 |
| O | 8.731700 | -0.647482 | -0.509316 | 8.767001 | -0.498628 | -0.491277 | 8.775273 | -0.484301 | -0.495125 | -8.752359 | 0.400675 | -0.547788 | 8.771905 | -0.464373 | -0.500860 | 8.756423 | -0.479017 | -0.480670 |
| C | -4.361142 | 0.346723 | -1.918162 | -4.356789 | 0.069223 | -1.933182 | -4.347456 | 0.050643 | -1.949634 | 4.321846 | 0.064129 | -2.000015 | -4.347268 | 0.010887 | -1.952880 | -4.370112 | 0.029752 | -1.945959 |
| C | -5.447041 | 0.232410 | -0.852084 | -5.456073 | 0.150169 | -0.879423 | -5.451976 | 0.180214 | -0.908572 | 5.407541 | -0.170641 | -0.961811 | -5.444949 | 0.178468 | -0.910485 | -5.444142 | 0.155741 | -0.872616 |
| O | -4.927382 | 0.319917 | 0.360662 | -4.951079 | 0.304508 | 0.329971 | -4.962170 | 0.276185 | 0.311337 | 4.916108 | -0.260291 | 0.251363 | -4.951896 | 0.263511 | 0.308268 | -4.913528 | 0.297422 | 0.325325 |
| O | -6.606241 | 0.096272 | -1.135417 | -6.619983 | 0.099433 | -1.178907 | -6.613080 | 0.208578 | -1.229112 | 6.569064 | -0.269656 | -1.283413 | -6.605702 | 0.239513 | -1.229144 | -6.616109 | 0.144360 | -1.150369 |
| C | -5.718064 | 0.134640 | 1.576586 | -5.777092 | 0.388923 | 1.537614 | -5.801762 | 0.363547 | 1.512821 | 5.733495 | -0.451718 | 1.461901 | -5.785969 | 0.397013 | 1.509860 | -5.708314 | 0.418370 | 1.552764 |
| C | -6.759964 | 1.237613 | 1.693411 | -6.665411 | 1.621750 | 1.471647 | -6.628222 | 1.638776 | 1.477285 | 6.446541 | -1.791485 | 1.393515 | -6.558213 | 1.705633 | 1.460185 | -6.557826 | 1.678303 | 1.497105 |
| C | -4.671617 | 0.253250 | 2.673888 | -4.739729 | 0.525901 | 2.640194 | -4.777071 | 0.415966 | 2.633313 | 4.685749 | -0.449579 | 2.561981 | -4.757056 | 0.418637 | 2.627874 | -4.639097 | 0.531673 | 2.627067 |
| C | -6.338683 | -1.255716 | 1.572841 | -6.574268 | -0.895932 | 1.704184 | -6.659362 | -0.886525 | 1.627951 | 6.686961 | 0.720600 | 1.623265 | -6.695037 | -0.814984 | 1.638755 | -6.539272 | -0.840064 | 1.749558 |
| H | -0.935337 | 3.192653 | -0.168770 | -1.020088 | 3.055020 | -0.254611 | -1.029471 | 3.038385 | -0.187835 | 1.095541 | -2.979700 | -0.110928 | -1.049095 | 3.019157 | -0.190904 | -1.050593 | 3.031748 | -0.252495 |
| H | 0.387566 | 5.221590 | 0.251199 | 0.230890 | 5.131971 | 0.146477 | 0.213304 | 5.112706 | 0.262516 | -0.126959 | -5.073151 | 0.302035 | 0.186257 | 5.102834 | 0.238004 | 0.191737 | 5.113420 | 0.156123 |
| H | 2.830765 | 5.083194 | 0.554929 | 2.676906 | 5.079741 | 0.460757 | 2.661830 | 5.063817 | 0.563113 | -2.580022 | -5.060619 | 0.558027 | 2.636638 | 5.067756 | 0.525805 | 2.639289 | 5.071364 | 0.462760 |
| H | 3.975183 | 2.890088 | 0.484661 | 3.895832 | 2.927022 | 0.424915 | 3.892969 | 2.921149 | 0.469175 | -3.841422 | -2.935547 | 0.457287 | 3.877436 | 2.929719 | 0.440890 | 3.869559 | 2.925152 | 0.413947 |
| H | -0.042345 | -2.646933 | 0.495049 | 0.058772 | -2.708806 | 0.581768 | 0.062155 | -2.671401 | 0.640947 | -0.023605 | 2.649445 | 0.828743 | 0.067644 | -2.684693 | 0.659197 | 0.047274 | -2.703564 | 0.614082 |
| H | -4.566775 | -2.406965 | -1.541266 | -4.530872 | -2.654017 | -1.319627 | -4.528274 | -2.660023 | -1.268460 | 4.498050 | 2.764617 | -1.239562 | -4.518048 | -2.700652 | -1.261874 | -4.538222 | -2.686296 | -1.303033 |
| H | -4.686850 | -0.228373 | -2.784820 | -4.680414 | -0.632883 | -2.701351 | -4.667608 | -0.685301 | -2.686821 | 4.663729 | 0.850643 | -2.672342 | -4.677263 | -0.742540 | -2.667664 | -4.713362 | -0.696831 | -2.681832 |
| H | -4.285722 | 1.394599 | -2.212584 | -4.273992 | 1.055467 | -2.392299 | -4.262157 | 1.013764 | -2.453895 | 4.229242 | -0.851746 | -2.584757 | -4.258641 | 0.958852 | -2.484683 | -4.292738 | 0.998059 | -2.442262 |
| H | -1.480651 | -4.635314 | 0.430292 | -1.340612 | -4.724626 | 0.659871 | -1.338121 | -4.686923 | 0.761729 | 1.361422 | 4.674915 | 0.979966 | -1.322905 | -4.707749 | 0.781662 | -1.350329 | -4.720165 | 0.722092 |
| H | -3.715385 | -4.536717 | -0.603632 | -3.608226 | -4.717457 | -0.306423 | -3.604241 | -4.700567 | -0.210252 | 3.591857 | 4.750345 | -0.066696 | -3.586759 | -4.735071 | -0.195526 | -3.615607 | -4.731800 | -0.250744 |
| H | 3.578402 | -1.360508 | 1.658677 | 3.639024 | -1.251140 | 1.724914 | 3.646276 | -1.248949 | 1.716016 | -3.663426 | 1.242577 | 1.722102 | 3.648435 | -1.234942 | 1.721644 | 3.625722 | -1.231022 | 1.729483 |
| H | 4.553388 | 1.402639 | -1.457424 | 4.525963 | 1.373302 | -1.537761 | 4.532047 | 1.387404 | -1.541794 | -4.467164 | -1.393432 | -1.558806 | 4.519674 | 1.381871 | -1.555738 | 4.507849 | 1.358074 | -1.564677 |
| H | 5.947721 | -2.109829 | 1.724880 | 6.022439 | -1.929482 | 1.821867 | 6.023628 | -1.927596 | 1.812538 | -6.049359 | 1.893760 | 1.783791 | 6.029300 | -1.902445 | 1.821387 | 6.009950 | -1.898377 | 1.840547 |
| H | 6.924918 | 0.664186 | -1.365417 | 6.912975 | 0.714648 | -1.417058 | 6.914588 | 0.734419 | -1.423886 | -6.858298 | -0.769354 | -1.477034 | 6.905677 | 0.739053 | -1.436058 | 6.895744 | 0.712397 | -1.432884 |
| H | -7.251451 | 1.163674 | 2.664808 | -7.160683 | 1.756911 | 2.434733 | -7.126922 | 1.763098 | 2.440036 | 6.914591 | -1.989432 | 2.359324 | -7.049971 | 1.861651 | 2.421841 | -7.021618 | 1.836869 | 2.472221 |
| H | -7.513452 | 1.154474 | 0.913434 | -7.427924 | 1.525646 | 0.701492 | -7.385918 | 1.605048 | 0.696888 | 7.221507 | -1.802051 | 0.629143 | -7.317674 | 1.694595 | 0.680732 | -7.344339 | 1.599963 | 0.749155 |
| H | -6.283802 | 2.217000 | 1.626344 | -6.067236 | 2.512171 | 1.269744 | -5.985340 | 2.505644 | 1.312667 | 5.733328 | -2.592181 | 1.189130 | -5.878891 | 2.542038 | 1.284860 | -5.936548 | 2.546738 | 1.270362 |
| H | -5.141685 | 0.128973 | 3.650032 | -5.235273 | 0.595612 | 3.609392 | -5.288274 | 0.479009 | 3.594730 | 5.169551 | -0.584729 | 3.530006 | -5.262772 | 0.512894 | 3.589617 | -5.108225 | 0.626067 | 3.607235 |
| H | -4.191273 | 1.231726 | 2.640774 | -4.138216 | 1.424317 | 2.493686 | -4.132033 | 1.289726 | 2.525828 | 3.971242 | -1.261392 | 2.414292 | -4.076820 | 1.263774 | 2.509029 | -4.013753 | 1.409467 | 2.455930 |
| H | -3.906092 | -0.514604 | 2.554707 | -4.074835 | -0.339270 | 2.651700 | -4.155522 | -0.481270 | 2.628693 | 4.143286 | 0.497728 | 2.573324 | -4.173449 | -0.503740 | 2.630676 | -4.002801 | -0.355053 | 2.629939 |
| H | -7.100832 | -1.349424 | 0.802241 | -7.328840 | -1.003534 | 0.927596 | -7.414341 | -0.930889 | 0.845296 | 7.472874 | 0.716148 | 0.870150 | -7.450003 | -0.836985 | 0.855093 | -7.320767 | -0.929022 | 0.997356 |
| H | -5.567778 | -2.010837 | 1.408408 | -5.910027 | -1.761941 | 1.679095 | -6.036582 | -1.781837 | 1.573975 | 6.141376 | 1.664489 | 1.564466 | -6.109745 | -1.735768 | 1.597060 | -5.902597 | -1.726098 | 1.710062 |
| H | -6.800081 | -1.443500 | 2.543443 | -7.075948 | -0.881478 | 2.673228 | -7.164308 | -0.880423 | 2.595344 | 7.155501 | 0.657865 | 2.606821 | -7.200107 | -0.775304 | 2.605266 | -7.010439 | -0.803719 | 2.733230 |

| **iso-I** 14 |  | gasphase |  |  | toluene |  |  | DMSO |  |  | methanol |  |  | acetonitrile |  |  | chloroform |  |
| --- | --- | --- | --- | --- | --- | --- | --- | --- | --- | --- | --- | --- | --- | --- | --- | --- | --- | --- |
| Element | x | y | z | x | y | z | x | y | z | x | y | z | x | y | z | x | y | z |
| C | -0.323230 | -2.659717 | -0.831854 | -0.319775 | -2.664217 | -0.811058 | -0.297019 | -2.669901 | -0.881665 | -0.273439 | -2.651848 | -1.136708 | -0.296820 | -2.673458 | -0.908801 | -0.309781 | -2.664580 | -0.892304 |
| C | 0.302985 | -3.870955 | -1.084911 | 0.306575 | -3.874989 | -1.066057 | 0.337615 | -3.875157 | -1.143979 | 0.375393 | -3.847492 | -1.408035 | 0.338616 | -3.878947 | -1.169065 | 0.321032 | -3.873286 | -1.147455 |
| C | 1.684050 | -3.981421 | -1.032796 | 1.687938 | -3.986029 | -1.006621 | 1.719735 | -3.981502 | -1.061317 | 1.753342 | -3.953613 | -1.271481 | 1.720625 | -3.985146 | -1.083312 | 1.702073 | -3.983868 | -1.066559 |
| C | 2.472563 | -2.877144 | -0.741172 | 2.476619 | -2.883191 | -0.709042 | 2.501402 | -2.880465 | -0.737100 | 2.519748 | -2.861409 | -0.884278 | 2.501345 | -2.883627 | -0.757663 | 2.486467 | -2.882959 | -0.749074 |
| C | 1.834624 | -1.680633 | -0.485171 | 1.837991 | -1.686816 | -0.453772 | 1.853997 | -1.689947 | -0.476325 | 1.858073 | -1.682572 | -0.612883 | 1.853277 | -1.693071 | -0.498658 | 1.843217 | -1.689042 | -0.494237 |
| C | 0.439261 | -1.542566 | -0.507601 | 0.442536 | -1.549487 | -0.477858 | 0.458411 | -1.560548 | -0.516199 | 0.465287 | -1.555992 | -0.703422 | 0.457630 | -1.563533 | -0.542246 | 0.448092 | -1.553778 | -0.536166 |
| N | 2.433042 | -0.436373 | -0.260104 | 2.434118 | -0.442960 | -0.221529 | 2.438671 | -0.445582 | -0.219867 | 2.426081 | -0.440354 | -0.299989 | 2.437771 | -0.448326 | -0.241170 | 2.433785 | -0.444880 | -0.246240 |
| C | 1.472836 | 0.562460 | -0.197576 | 1.475139 | 0.551454 | -0.143367 | 1.474601 | 0.537930 | -0.126139 | 1.456398 | 0.532473 | -0.223926 | 1.473165 | 0.534851 | -0.151436 | 1.472607 | 0.545147 | -0.171777 |
| C | 0.129862 | -0.119668 | -0.314319 | 0.132405 | -0.128854 | -0.277449 | 0.138292 | -0.147810 | -0.300906 | 0.132978 | -0.153094 | -0.462532 | 0.137254 | -0.150390 | -0.328127 | 0.133499 | -0.136695 | -0.329339 |
| C | -1.052868 | 0.535919 | -0.198230 | -1.051231 | 0.525351 | -0.168856 | -1.050061 | 0.495435 | -0.201357 | -1.058431 | 0.483765 | -0.364859 | -1.050323 | 0.494702 | -0.228224 | -1.051579 | 0.513561 | -0.221670 |
| O | 1.730918 | 1.723512 | 0.000085 | 1.728198 | 1.709432 | 0.084385 | 1.708950 | 1.689817 | 0.155621 | 1.669651 | 1.681514 | 0.098911 | 1.706302 | 1.687690 | 0.128188 | 1.715695 | 1.702390 | 0.076028 |
| C | -1.417333 | 1.949869 | -0.283721 | -1.413124 | 1.939321 | -0.268229 | -1.409457 | 1.907729 | -0.312063 | -1.413338 | 1.895567 | -0.455702 | -1.408222 | 1.908047 | -0.333674 | -1.411589 | 1.927372 | -0.318007 |
| C | -2.804681 | 2.055672 | -0.095401 | -2.800425 | 2.047838 | -0.084493 | -2.791745 | 2.021660 | -0.105290 | -2.784180 | 2.017825 | -0.195879 | -2.789400 | 2.023260 | -0.119985 | -2.794528 | 2.039544 | -0.109913 |
| N | -3.358351 | 0.778950 | 0.122466 | -3.353903 | 0.774613 | 0.144691 | -3.341745 | 0.752790 | 0.148984 | -3.330513 | 0.745027 | 0.073902 | -3.340211 | 0.753887 | 0.133074 | -3.346183 | 0.766925 | 0.130121 |
| C | -2.342235 | -0.187745 | 0.124677 | -2.342286 | -0.192512 | 0.155725 | -2.337193 | -0.219649 | 0.136152 | -2.336527 | -0.233225 | -0.021113 | -2.337430 | -0.219980 | 0.108386 | -2.338836 | -0.203684 | 0.112073 |
| C | -0.703012 | 3.100993 | -0.606659 | -0.699049 | 3.084654 | -0.610634 | -0.699743 | 3.039551 | -0.703078 | -0.709885 | 3.016092 | -0.887001 | -0.699002 | 3.040393 | -0.724875 | -0.700953 | 3.067006 | -0.685297 |
| C | -1.365485 | 4.313059 | -0.705020 | -1.360671 | 4.295674 | -0.728843 | -1.360154 | 4.250582 | -0.834351 | -1.365184 | 4.231693 | -0.994405 | -1.358788 | 4.252659 | -0.849426 | -1.361167 | 4.279767 | -0.797373 |
| C | -2.732644 | 4.390751 | -0.489362 | -2.728647 | 4.376992 | -0.516284 | -2.724202 | 4.340845 | -0.593048 | -2.717343 | 4.332912 | -0.696030 | -2.721752 | 4.343820 | -0.601578 | -2.724848 | 4.366344 | -0.557003 |
| C | -3.475583 | 3.258551 | -0.185854 | -3.471350 | 3.248966 | -0.196993 | -3.463087 | 3.221515 | -0.234445 | -3.452001 | 3.220984 | -0.305494 | -3.460274 | 3.224104 | -0.242792 | -3.464647 | 3.241784 | -0.217603 |
| O | -2.480233 | -1.333689 | 0.448032 | -2.484928 | -1.335140 | 0.493366 | -2.480738 | -1.363873 | 0.474491 | -2.478317 | -1.396250 | 0.259859 | -2.481560 | -1.367529 | 0.435635 | -2.480205 | -1.351296 | 0.436964 |
| C | 3.806904 | -0.209139 | -0.039614 | 3.811903 | -0.212241 | -0.021175 | 3.814897 | -0.209933 | -0.008553 | 3.789058 | -0.205915 | -0.012194 | 3.812188 | -0.212764 | -0.017035 | 3.808449 | -0.212970 | -0.022886 |
| C | 4.496110 | -0.993065 | 0.880537 | 4.505795 | -0.960302 | 0.923954 | 4.500483 | -0.947384 | 0.949862 | 4.429047 | -0.974487 | 0.953157 | 4.490092 | -0.954592 | 0.943796 | 4.486963 | -0.962174 | 0.932302 |
| C | 5.842949 | -0.780984 | 1.100903 | 5.855256 | -0.743142 | 1.122016 | 5.847342 | -0.722571 | 1.157424 | 5.761245 | -0.749306 | 1.237352 | 5.834570 | -0.728677 | 1.165443 | 5.832192 | -0.741616 | 1.153768 |
| C | 6.480319 | 0.229212 | 0.404157 | 6.488338 | 0.235436 | 0.376601 | 6.482317 | 0.251418 | 0.406618 | 6.424875 | 0.255550 | 0.554417 | 6.474200 | 0.252459 | 0.427843 | 6.474304 | 0.241576 | 0.421633 |
| C | 5.809287 | 1.028994 | -0.502562 | 5.811638 | 0.998264 | -0.558451 | 5.813043 | 1.000868 | -0.545568 | 5.800822 | 1.035872 | -0.403824 | 5.812729 | 1.006942 | -0.525856 | 5.812833 | 1.004523 | -0.524629 |
| C | 4.464992 | 0.803760 | -0.727791 | 4.464923 | 0.767039 | -0.760292 | 4.469180 | 0.761683 | -0.756244 | 4.471712 | 0.795885 | -0.691423 | 4.471858 | 0.764704 | -0.752654 | 4.470719 | 0.768707 | -0.750617 |
| N | 7.914113 | 0.460655 | 0.636998 | 7.922407 | 0.471734 | 0.585113 | 7.910791 | 0.495360 | 0.624590 | 7.833872 | 0.500347 | 0.855390 | 7.898914 | 0.499732 | 0.663674 | 7.901320 | 0.482348 | 0.656540 |
| O | 8.443904 | 1.363072 | 0.025934 | 8.452210 | 1.350246 | -0.062318 | 8.446989 | 1.370381 | -0.025608 | 8.395697 | 1.413262 | 0.283023 | 8.430586 | 1.406717 | 0.055368 | 8.437886 | 1.377983 | 0.037533 |
| O | 8.479197 | -0.266388 | 1.425340 | 8.500003 | -0.225661 | 1.392778 | 8.489379 | -0.189871 | 1.444142 | 8.383884 | -0.219769 | 1.665131 | 8.479942 | -0.214594 | 1.455866 | 8.474582 | -0.227074 | 1.457694 |
| C | -4.718985 | 0.544742 | 0.396510 | -4.714618 | 0.543327 | 0.427496 | -4.693778 | 0.534581 | 0.487388 | -4.649560 | 0.537009 | 0.516447 | -4.687529 | 0.537761 | 0.490038 | -4.698131 | 0.541894 | 0.456081 |
| O | -5.045559 | -0.682414 | 0.061608 | -5.062032 | -0.657446 | 0.031324 | -5.086553 | -0.637081 | 0.056183 | -5.063017 | -0.660906 | 0.199539 | -5.086843 | -0.635914 | 0.071187 | -5.066662 | -0.654345 | 0.070616 |
| O | -5.438072 | 1.397809 | 0.842068 | -5.412085 | 1.383874 | 0.929133 | -5.342881 | 1.368176 | 1.062809 | -5.266867 | 1.402715 | 1.085255 | -5.328986 | 1.374108 | 1.070344 | -5.372389 | 1.386554 | 0.984139 |
| C | -6.342880 | -1.261756 | 0.417020 | -6.369570 | -1.240097 | 0.362239 | -6.406963 | -1.196596 | 0.397931 | -6.359272 | -1.204136 | 0.660854 | -6.400309 | -1.196386 | 0.437332 | -6.373156 | -1.226585 | 0.434432 |
| C | -6.530889 | -1.217736 | 1.926783 | -6.546345 | -1.282490 | 1.872115 | -6.549986 | -1.296785 | 1.907546 | -6.399156 | -1.212301 | 2.179382 | -6.515720 | -1.291421 | 1.949851 | -6.509546 | -1.272143 | 1.947970 |
| C | -7.456951 | -0.541909 | -0.329318 | -7.475169 | -0.461815 | -0.334125 | -7.501284 | -0.356915 | -0.239864 | -7.495323 | -0.409260 | 0.040483 | -7.506801 | -0.360644 | -0.184996 | -7.486679 | -0.434405 | -0.232081 |
| C | -6.201870 | -2.696602 | -0.066766 | -6.255207 | -2.644847 | -0.206437 | -6.352739 | -2.578426 | -0.229766 | -6.324798 | -2.619953 | 0.113039 | -6.352065 | -2.580181 | -0.187240 | -6.286068 | -2.629828 | -0.141805 |
| H | -1.396484 | -2.594998 | -0.873302 | -1.392976 | -2.598262 | -0.861508 | -1.369372 | -2.602751 | -0.957707 | -1.341228 | -2.579629 | -1.265476 | -1.368999 | -2.606257 | -0.986937 | -1.382017 | -2.596592 | -0.964482 |
| H | -0.298162 | -4.737576 | -1.325324 | -0.293644 | -4.740569 | -1.313767 | -0.253914 | -4.739256 | -1.417672 | -0.201033 | -4.703748 | -1.733767 | -0.252338 | -4.743208 | -1.443450 | -0.274331 | -4.737052 | -1.413218 |
| H | 2.158609 | -4.933433 | -1.233351 | 2.162989 | -4.938178 | -1.206691 | 2.201492 | -4.929399 | -1.266461 | 2.245467 | -4.894146 | -1.486021 | 2.203046 | -4.933029 | -1.286906 | 2.180748 | -4.934301 | -1.266746 |
| H | 3.551070 | -2.949569 | -0.735935 | 3.555113 | -2.958758 | -0.697058 | 3.579844 | -2.954868 | -0.707368 | 3.596559 | -2.934436 | -0.815463 | 3.579688 | -2.957655 | -0.725109 | 3.564734 | -2.959122 | -0.721351 |
| H | 0.360448 | 3.049887 | -0.758464 | 0.364206 | 3.031430 | -0.766021 | 0.357969 | 2.977778 | -0.894551 | 0.335187 | 2.941742 | -1.138782 | 0.357686 | 2.978062 | -0.921929 | 0.358016 | 3.009368 | -0.868838 |
| H | -4.538337 | 3.313404 | -0.015894 | -4.535717 | 3.308349 | -0.035655 | -4.526360 | 3.289651 | -0.065869 | -4.509215 | 3.300185 | -0.106646 | -4.523049 | 3.292137 | -0.070513 | -4.526704 | 3.306453 | -0.042265 |
| H | -0.804219 | 5.206212 | -0.945297 | -0.799235 | 5.184904 | -0.984052 | -0.804189 | 5.131561 | -1.128243 | -0.816936 | 5.106663 | -1.319215 | -0.803147 | 5.133802 | -1.143415 | -0.803231 | 5.165627 | -1.071814 |
| H | -3.236778 | 5.346291 | -0.559114 | -3.233083 | 5.331118 | -0.603319 | -3.228629 | 5.293632 | -0.695118 | -3.218579 | 5.288848 | -0.783611 | -3.225606 | 5.297400 | -0.698853 | -3.228548 | 5.321230 | -0.640871 |
| H | 3.971369 | -1.762480 | 1.430372 | 3.986158 | -1.705662 | 1.510583 | 3.979791 | -1.691748 | 1.536921 | 3.883868 | -1.741591 | 1.486461 | 3.965219 | -1.702913 | 1.521969 | 3.960345 | -1.711191 | 1.507996 |
| H | 3.921283 | 1.415804 | -1.431772 | 3.918764 | 1.345426 | -1.491299 | 3.930054 | 1.322620 | -1.506403 | 3.966446 | 1.379671 | -1.448127 | 3.938933 | 1.328639 | -1.504947 | 3.936815 | 1.344010 | -1.493269 |
| H | 6.396827 | -1.376531 | 1.811286 | 6.408678 | -1.313043 | 1.853556 | 6.391621 | -1.287243 | 1.899849 | 6.270932 | -1.336252 | 1.987198 | 6.373206 | -1.296032 | 1.909966 | 6.372442 | -1.313160 | 1.893894 |
| H | 6.341459 | 1.809422 | -1.025795 | 6.335474 | 1.754580 | -1.123919 | 6.335282 | 1.750823 | -1.121063 | 6.345044 | 1.809328 | -0.925408 | 6.338937 | 1.762654 | -1.090103 | 6.342174 | 1.763505 | -1.081470 |
| H | -5.679132 | -1.681592 | 2.426199 | -5.691492 | -1.772527 | 2.341216 | -5.699665 | -1.829520 | 2.337228 | -5.515222 | -1.711445 | 2.580467 | -5.657093 | -1.822585 | 2.364665 | -5.647931 | -1.773509 | 2.392318 |
| H | -7.428623 | -1.779240 | 2.188955 | -7.441486 | -1.860139 | 2.108048 | -7.455932 | -1.860486 | 2.135605 | -7.281373 | -1.768473 | 2.500248 | -7.417302 | -1.854337 | 2.196279 | -7.404726 | -1.841179 | 2.203849 |
| H | -6.644676 | -0.197419 | 2.286665 | -6.661778 | -0.286432 | 2.294893 | -6.630257 | -0.316879 | 2.374295 | -6.460526 | -0.207522 | 2.593730 | -6.587308 | -0.309580 | 2.414059 | -6.603322 | -0.276435 | 2.377331 |
| H | -8.395504 | -1.074150 | -0.166831 | -8.420209 | -0.991254 | -0.201167 | -8.457649 | -0.863898 | -0.101250 | -8.435411 | -0.921157 | 0.252915 | -8.459390 | -0.870336 | -0.031218 | -8.432368 | -0.957364 | -0.079679 |
| H | -7.571118 | 0.482038 | 0.018706 | -7.580159 | 0.540350 | 0.075822 | -7.565793 | 0.631158 | 0.211464 | -7.555542 | 0.599596 | 0.444547 | -7.566905 | 0.627921 | 0.265964 | -7.573911 | 0.566453 | 0.185864 |
| H | -7.251840 | -0.533986 | -1.400879 | -7.275532 | -0.389099 | -1.404769 | -7.325357 | -0.249310 | -1.311814 | -7.374196 | -0.353650 | -1.042837 | -7.346274 | -0.254597 | -1.259478 | -7.309880 | -0.357989 | -1.306532 |
| H | -7.114638 | -3.251239 | 0.153228 | -7.181544 | -3.191399 | -0.025368 | -7.296210 | -3.095231 | -0.050472 | -7.243888 | -3.138371 | 0.388090 | -7.289546 | -3.100695 | 0.011542 | -7.212792 | -3.167467 | 0.062259 |
| H | -5.363170 | -3.183834 | 0.430382 | -5.433328 | -3.184618 | 0.264967 | -5.544930 | -3.168780 | 0.204926 | -5.477632 | -3.171125 | 0.524384 | -5.532961 | -3.164667 | 0.234119 | -5.457748 | -3.178919 | 0.307598 |
| H | -6.029831 | -2.722481 | -1.143233 | -6.078022 | -2.612082 | -1.282414 | -6.196436 | -2.507483 | -1.307343 | -6.244287 | -2.610309 | -0.975224 | -6.216346 | -2.510911 | -1.267721 | -6.137095 | -2.594477 | -1.222116 |

| **iso-I** 15 |  | gasphase |  |  | toluene |  |  | DMSO |  |  | methanol |  |  | acetonitrile |  |  | chloroform |  |
| --- | --- | --- | --- | --- | --- | --- | --- | --- | --- | --- | --- | --- | --- | --- | --- | --- | --- | --- |
| Element | x | y | z | x | y | z | x | y | z | x | y | z | x | y | z | x | y | z |
| C | 0.638164 | -2.839865 | -1.081952 | 0.634491 | -2.837715 | -1.018997 | 0.638611 | -2.817836 | -1.120816 | 0.640657 | -2.797724 | -1.203820 | 0.632755 | -2.810608 | -1.122165 | 0.621216 | -2.840268 | -0.977849 |
| C | 1.330580 | -4.033117 | -1.229977 | 1.326580 | -4.026830 | -1.197845 | 1.330671 | -4.007506 | -1.298734 | 1.333312 | -3.985844 | -1.388478 | 1.322368 | -4.001671 | -1.301266 | 1.308355 | -4.031119 | -1.166057 |
| C | 2.700970 | -4.088595 | -1.032316 | 2.701284 | -4.080915 | -1.025828 | 2.701638 | -4.068728 | -1.089484 | 2.700272 | -4.053699 | -1.155474 | 2.693050 | -4.066283 | -1.090493 | 2.684702 | -4.089812 | -1.003666 |
| C | 3.415493 | -2.947293 | -0.692909 | 3.419499 | -2.942622 | -0.684084 | 3.417018 | -2.937847 | -0.716842 | 3.413104 | -2.930570 | -0.754050 | 3.410481 | -2.937362 | -0.715251 | 3.409355 | -2.954532 | -0.664385 |
| C | 2.712717 | -1.770590 | -0.540662 | 2.716515 | -1.770031 | -0.502245 | 2.713227 | -1.764657 | -0.539104 | 2.707878 | -1.760624 | -0.568183 | 2.709070 | -1.762895 | -0.536214 | 2.710987 | -1.780502 | -0.474052 |
| C | 1.322911 | -1.686032 | -0.715258 | 1.323339 | -1.687814 | -0.647572 | 1.323993 | -1.678853 | -0.711657 | 1.322443 | -1.670159 | -0.759581 | 1.320167 | -1.673720 | -0.710078 | 1.316786 | -1.694143 | -0.606513 |
| N | 3.239459 | -0.500391 | -0.283239 | 3.242994 | -0.502340 | -0.233871 | 3.234276 | -0.500056 | -0.248629 | 3.224927 | -0.496517 | -0.255248 | 3.232682 | -0.499068 | -0.244636 | 3.242608 | -0.514243 | -0.207826 |
| C | 2.243998 | 0.461615 | -0.344050 | 2.243856 | 0.453864 | -0.246552 | 2.237640 | 0.455419 | -0.264321 | 2.231384 | 0.454629 | -0.276835 | 2.238602 | 0.458438 | -0.264181 | 2.246350 | 0.441790 | -0.202732 |
| C | 0.943047 | -0.273884 | -0.584447 | 0.943165 | -0.278851 | -0.493886 | 0.942921 | -0.274071 | -0.547804 | 0.942408 | -0.268276 | -0.583829 | 0.942052 | -0.267981 | -0.545160 | 0.942421 | -0.284523 | -0.446596 |
| C | -0.263303 | 0.347247 | -0.582678 | -0.263754 | 0.340078 | -0.493312 | -0.265611 | 0.337906 | -0.550063 | -0.265234 | 0.343616 | -0.585066 | -0.265462 | 0.346412 | -0.544479 | -0.261838 | 0.338569 | -0.444565 |
| O | 2.437521 | 1.635775 | -0.152586 | 2.430171 | 1.621886 | -0.007338 | 2.412657 | 1.618848 | 0.011664 | 2.399606 | 1.618784 | 0.018023 | 2.417173 | 1.623067 | 0.005757 | 2.435307 | 1.607940 | 0.050419 |
| C | -0.642205 | 1.762335 | -0.671151 | -0.641710 | 1.752695 | -0.608259 | -0.640969 | 1.748640 | -0.669359 | -0.639885 | 1.752276 | -0.711091 | -0.640207 | 1.757895 | -0.660721 | -0.634777 | 1.751743 | -0.569506 |
| C | -2.035302 | 1.838052 | -0.516311 | -2.037320 | 1.828807 | -0.479678 | -2.032657 | 1.829962 | -0.513350 | -2.028999 | 1.835356 | -0.542260 | -2.032206 | 1.839384 | -0.505587 | -2.031055 | 1.831796 | -0.451603 |
| N | -2.563923 | 0.560927 | -0.311047 | -2.566678 | 0.554529 | -0.259631 | -2.559695 | 0.557831 | -0.280787 | -2.552300 | 0.561331 | -0.294238 | -2.559786 | 0.566580 | -0.275437 | -2.564290 | 0.559603 | -0.228193 |
| C | -1.570000 | -0.396693 | -0.385895 | -1.570464 | -0.399700 | -0.287601 | -1.569308 | -0.398474 | -0.319276 | -1.563778 | -0.388544 | -0.335851 | -1.569480 | -0.389106 | -0.314654 | -1.570734 | -0.394292 | -0.234306 |
| C | 0.045998 | 2.930252 | -0.983348 | 0.051345 | 2.914560 | -0.931839 | 0.050797 | 2.901047 | -1.026599 | 0.049989 | 2.897465 | -1.093336 | 0.052236 | 2.911322 | -1.014182 | 0.063258 | 2.909916 | -0.896642 |
| C | -0.646565 | 4.127159 | -1.097428 | -0.639172 | 4.108822 | -1.079586 | -0.637538 | 4.098084 | -1.166810 | -0.639330 | 4.093347 | -1.237673 | -0.635793 | 4.108974 | -1.152434 | -0.623679 | 4.105284 | -1.055037 |
| C | -2.019968 | 4.172962 | -0.917601 | -2.015911 | 4.156584 | -0.921122 | -2.010439 | 4.154384 | -0.967927 | -2.009715 | 4.152831 | -1.022424 | -2.009048 | 4.164988 | -0.955595 | -2.001764 | 4.157176 | -0.904320 |
| C | -2.737756 | 3.018929 | -0.632300 | -2.738183 | 3.007559 | -0.626882 | -2.732249 | 3.011335 | -0.647972 | -2.730107 | 3.013893 | -0.683083 | -2.731404 | 3.021122 | -0.638955 | -2.728806 | 3.011108 | -0.608917 |
| O | -1.763674 | -1.577394 | -0.237826 | -1.758352 | -1.575890 | -0.091709 | -1.753038 | -1.573360 | -0.100065 | -1.740540 | -1.565826 | -0.098643 | -1.753754 | -1.564568 | -0.097300 | -1.760349 | -1.567785 | -0.015491 |
| C | 4.575888 | -0.215860 | 0.066305 | 4.589588 | -0.216835 | 0.078106 | 4.577071 | -0.219787 | 0.085672 | 4.563934 | -0.218340 | 0.098899 | 4.576256 | -0.220241 | 0.088521 | 4.594710 | -0.227520 | 0.080706 |
| C | 5.265470 | 0.798882 | -0.587035 | 5.265299 | 0.777027 | -0.619786 | 5.269744 | 0.761751 | -0.613009 | 5.261112 | 0.773517 | -0.580337 | 5.268067 | 0.763529 | -0.608222 | 5.255699 | 0.762027 | -0.637285 |
| C | 6.571559 | 1.081013 | -0.235954 | 6.580261 | 1.063831 | -0.309214 | 6.579121 | 1.047675 | -0.279302 | 6.566466 | 1.056536 | -0.230299 | 6.579130 | 1.046136 | -0.277748 | 6.574281 | 1.055628 | -0.349859 |
| C | 7.173497 | 0.335016 | 0.760566 | 7.203746 | 0.339577 | 0.691169 | 7.177255 | 0.332685 | 0.744024 | 7.153680 | 0.327609 | 0.789706 | 7.179503 | 0.325723 | 0.740655 | 7.214328 | 0.340753 | 0.647377 |
| C | 6.503545 | -0.676347 | 1.424158 | 6.547636 | -0.653679 | 1.396079 | 6.504593 | -0.652587 | 1.445650 | 6.475855 | -0.667236 | 1.473311 | 6.507070 | -0.660125 | 1.441914 | 6.573541 | -0.650162 | 1.370130 |
| C | 5.193901 | -0.945121 | 1.077218 | 5.228675 | -0.925618 | 1.089692 | 5.191155 | -0.923288 | 1.115491 | 5.166530 | -0.934931 | 1.126260 | 5.192000 | -0.927405 | 1.115024 | 5.250533 | -0.928146 | 1.086859 |
| N | 8.567703 | 0.626807 | 1.128275 | 8.604861 | 0.634846 | 1.016680 | 8.568036 | 0.629110 | 1.097375 | 8.537813 | 0.617841 | 1.157980 | 8.572963 | 0.615736 | 1.088218 | 8.616721 | 0.644544 | 0.949485 |
| O | 9.074220 | -0.055029 | 1.992965 | 9.142179 | -0.039148 | 1.870669 | 9.092087 | -0.039740 | 1.965799 | 9.060322 | -0.063414 | 2.018018 | 9.102121 | -0.063721 | 1.944875 | 9.177236 | -0.028338 | 1.790117 |
| O | 9.125286 | 1.529988 | 0.543783 | 9.149179 | 1.535552 | 0.413332 | 9.129172 | 1.529386 | 0.505239 | 9.107514 | 1.527749 | 0.588632 | 9.132193 | 1.521296 | 0.502983 | 9.146444 | 1.553003 | 0.343450 |
| C | -3.913533 | 0.265742 | -0.009865 | -3.924673 | 0.261007 | 0.010229 | -3.916262 | 0.270735 | 0.007822 | -3.905978 | 0.271741 | 0.010892 | -3.916509 | 0.276099 | 0.010011 | -3.927061 | 0.267886 | 0.022540 |
| C | -4.601699 | -0.683469 | -0.754456 | -4.601157 | -0.667047 | -0.769952 | -4.625168 | -0.601511 | -0.807900 | -4.615661 | -0.615376 | -0.787588 | -4.618443 | -0.603263 | -0.804423 | -4.594339 | -0.649600 | -0.778146 |
| C | -5.917503 | -0.971425 | -0.448635 | -5.924927 | -0.955071 | -0.498205 | -5.945101 | -0.887464 | -0.516914 | -5.931414 | -0.905139 | -0.481358 | -5.938083 | -0.894325 | -0.516673 | -5.922332 | -0.937538 | -0.527494 |
| C | -6.553198 | -0.308020 | 0.592339 | -6.576230 | -0.309995 | 0.544616 | -6.556194 | -0.293658 | 0.580964 | -6.536250 | -0.300456 | 0.613882 | -6.555881 | -0.298800 | 0.576716 | -6.585043 | -0.301626 | 0.514543 |
| C | -5.866751 | 0.639004 | 1.334569 | -5.901641 | 0.619345 | 1.321060 | -5.851257 | 0.584683 | 1.388477 | -5.829265 | 0.592073 | 1.404081 | -5.857675 | 0.586157 | 1.383065 | -5.919687 | 0.618118 | 1.310258 |
| C | -4.543775 | 0.918910 | 1.039855 | -4.571709 | 0.897936 | 1.059654 | -4.524470 | 0.861856 | 1.104777 | -4.506667 | 0.873473 | 1.105786 | -4.531074 | 0.868524 | 1.102810 | -4.585295 | 0.896409 | 1.069476 |
| C | -8.000832 | -0.608209 | 0.874575 | -8.031925 | -0.597178 | 0.789057 | -7.999767 | -0.604245 | 0.855302 | -7.976788 | -0.613146 | 0.904053 | -7.999174 | -0.615226 | 0.847654 | -8.043138 | -0.592355 | 0.735987 |
| F | -8.803797 | -0.062576 | -0.054321 | -8.819127 | 0.065860 | -0.078638 | -8.811300 | -0.078622 | -0.082741 | -8.798442 | -0.087913 | -0.025354 | -8.810029 | -0.097620 | -0.095507 | -8.818095 | 0.039859 | -0.166544 |
| F | -8.245054 | -1.925918 | 0.868604 | -8.319737 | -1.898932 | 0.645235 | -8.238337 | -1.927298 | 0.859101 | -8.213491 | -1.936225 | 0.910522 | -8.231367 | -1.939393 | 0.856384 | -8.320408 | -1.900379 | 0.621653 |
| F | -8.397249 | -0.135111 | 2.061270 | -8.426749 | -0.239727 | 2.017213 | -8.419958 | -0.131544 | 2.034622 | -8.384508 | -0.140552 | 2.087796 | -8.425677 | -0.139293 | 2.023441 | -8.469449 | -0.205879 | 1.944742 |
| H | -0.426117 | -2.811758 | -1.234512 | -0.432753 | -2.810609 | -1.153227 | -0.424022 | -2.781997 | -1.291592 | -0.416935 | -2.753898 | -1.405633 | -0.429480 | -2.771763 | -1.294687 | -0.446760 | -2.809184 | -1.108143 |
| H | 0.788879 | -4.928345 | -1.504311 | 0.782247 | -4.919925 | -1.475193 | 0.792388 | -4.894726 | -1.606401 | 0.799574 | -4.866191 | -1.722778 | 0.782443 | -4.887099 | -1.611153 | 0.759609 | -4.921650 | -1.443622 |
| H | 3.227756 | -5.026388 | -1.154145 | 3.227944 | -5.015899 | -1.169718 | 3.228248 | -5.004300 | -1.231129 | 3.226966 | -4.988209 | -1.304077 | 3.217844 | -5.002707 | -1.233156 | 3.207538 | -5.026049 | -1.154034 |
| H | 4.489150 | -2.979086 | -0.571832 | 4.495153 | -2.974681 | -0.580915 | 4.489587 | -2.977465 | -0.585450 | 4.483878 | -2.974207 | -0.609285 | 4.482790 | -2.979330 | -0.582951 | 4.485655 | -2.990924 | -0.568934 |
| H | 1.112554 | 2.910174 | -1.120263 | 1.120220 | 2.892802 | -1.053554 | 1.115096 | 2.870787 | -1.187682 | 1.110963 | 2.862296 | -1.278679 | 1.116790 | 2.880998 | -1.173496 | 1.132689 | 2.885065 | -1.015702 |
| H | -3.812901 | 3.043274 | -0.522746 | -3.814831 | 3.035958 | -0.533107 | -3.805941 | 3.047396 | -0.523744 | -3.802764 | 3.052015 | -0.550227 | -3.805195 | 3.056936 | -0.515988 | -3.805966 | 3.043045 | -0.520957 |
| H | -0.102761 | 5.032953 | -1.329698 | -0.092350 | 5.010875 | -1.320700 | -0.094691 | 4.995128 | -1.435506 | -0.099869 | 4.986792 | -1.524691 | -0.092324 | 5.006559 | -1.417992 | -0.073512 | 5.004790 | -1.298829 |
| H | -2.546269 | 5.114365 | -1.010741 | -2.541000 | 5.096162 | -1.038648 | -2.533246 | 5.096442 | -1.077127 | -2.532765 | 5.094248 | -1.136444 | -2.531681 | 5.107276 | -1.063511 | -2.523934 | 5.097541 | -1.029300 |
| H | 4.775803 | 1.367790 | -1.363237 | 4.761002 | 1.323275 | -1.403727 | 4.787299 | 1.294929 | -1.419825 | 4.785733 | 1.315934 | -1.385553 | 4.783191 | 1.300764 | -1.410866 | 4.737832 | 1.298058 | -1.419822 |
| H | 4.642433 | -1.716468 | 1.597440 | 4.689470 | -1.682699 | 1.642860 | 4.639723 | -1.675925 | 1.662647 | 4.609828 | -1.694839 | 1.657902 | 4.640864 | -1.680710 | 1.661491 | 4.722823 | -1.682952 | 1.654042 |
| H | 7.127411 | 1.864123 | -0.729733 | 7.121007 | 1.833020 | -0.840368 | 7.130139 | 1.807113 | -0.814086 | 7.121332 | 1.822937 | -0.751091 | 7.129767 | 1.806580 | -0.811582 | 7.102754 | 1.821751 | -0.897659 |
| H | 7.002750 | -1.228672 | 2.206382 | 7.059036 | -1.192410 | 2.179846 | 6.993591 | -1.189279 | 2.245040 | 6.956824 | -1.213207 | 2.271515 | 6.997685 | -1.199766 | 2.238354 | 7.096562 | -1.183099 | 2.150334 |
| H | -4.101345 | -1.198149 | -1.561817 | -4.089731 | -1.162961 | -1.582788 | -4.144920 | -1.049894 | -1.666717 | -4.138890 | -1.072651 | -1.644156 | -4.132109 | -1.053678 | -1.658804 | -4.073536 | -1.134990 | -1.591864 |
| H | -3.995328 | 1.640978 | 1.630070 | -4.032833 | 1.606455 | 1.674669 | -3.961176 | 1.535093 | 1.737586 | -3.941828 | 1.557547 | 1.725578 | -3.973072 | 1.546681 | 1.734992 | -4.053548 | 1.599001 | 1.697461 |
| H | -6.453012 | -1.714439 | -1.024643 | -6.451221 | -1.681038 | -1.103866 | -6.499241 | -1.567575 | -1.151200 | -6.486379 | -1.596955 | -1.102151 | -6.486757 | -1.580113 | -1.149580 | -6.442348 | -1.654212 | -1.149782 |
| H | -6.355694 | 1.148385 | 2.153058 | -6.403514 | 1.117663 | 2.138957 | -6.322639 | 1.048296 | 2.243937 | -6.295700 | 1.063881 | 2.257863 | -6.334183 | 1.050633 | 2.235198 | -6.430498 | 1.111133 | 2.125848 |

| **iso-I** 17 |  | gasphase |  |  | toluene |  |  | DMSO |  |  | methanol |  |  | acetonitrile |  |  | chloroform |  |
| --- | --- | --- | --- | --- | --- | --- | --- | --- | --- | --- | --- | --- | --- | --- | --- | --- | --- | --- |
| Element | x | y | z | x | y | z | x | y | z | x | y | z | x | y | z | x | y | z |
| C | -1.497715 | 3.248039 | 0.160113 | -1.497815 | 3.238494 | 0.183478 | -1.500307 | 3.222485 | 0.218992 | -1.510090 | 3.215261 | 0.266656 | -1.502584 | 3.227335 | 0.214876 | -1.501593 | 3.234687 | 0.201711 |
| C | -0.679288 | 4.368132 | 0.211360 | -0.680157 | 4.357602 | 0.260373 | -0.684482 | 4.341213 | 0.322371 | -0.698338 | 4.336337 | 0.372749 | -0.686987 | 4.346783 | 0.314803 | -0.685318 | 4.354498 | 0.287595 |
| C | 0.698065 | 4.246664 | 0.130700 | 0.698821 | 4.235780 | 0.195018 | 0.696823 | 4.219878 | 0.267596 | 0.683100 | 4.221414 | 0.301770 | 0.694409 | 4.225786 | 0.260923 | 0.694837 | 4.233732 | 0.227125 |
| C | 1.293998 | 2.996207 | 0.029664 | 1.295859 | 2.986640 | 0.083735 | 1.297070 | 2.973248 | 0.142193 | 1.289525 | 2.978784 | 0.162322 | 1.295035 | 2.978713 | 0.139516 | 1.294243 | 2.985629 | 0.112796 |
| C | 0.469728 | 1.891294 | 0.004266 | 0.470756 | 1.883060 | 0.029619 | 0.472160 | 1.870805 | 0.059286 | 0.468295 | 1.874918 | 0.076768 | 0.470566 | 1.875754 | 0.059820 | 0.469858 | 1.882007 | 0.048371 |
| C | -0.931107 | 1.983907 | 0.034005 | -0.929979 | 1.976504 | 0.043497 | -0.927805 | 1.965238 | 0.058664 | -0.930794 | 1.964910 | 0.082409 | -0.929735 | 1.969439 | 0.058783 | -0.930702 | 1.974854 | 0.055207 |
| N | 0.857081 | 0.549821 | -0.071052 | 0.856403 | 0.542054 | -0.050652 | 0.855745 | 0.530276 | -0.028916 | 0.852980 | 0.531846 | -0.016388 | 0.855005 | 0.534930 | -0.025964 | 0.855328 | 0.540716 | -0.032251 |
| C | -0.251704 | -0.279110 | -0.071308 | -0.249714 | -0.285870 | -0.058929 | -0.247678 | -0.296463 | -0.040557 | -0.246690 | -0.291580 | -0.025493 | -0.248016 | -0.291460 | -0.039464 | -0.248704 | -0.286410 | -0.041275 |
| C | -1.470000 | 0.616777 | -0.007583 | -1.468207 | 0.610259 | -0.008623 | -1.464885 | 0.601419 | -0.004561 | -1.462484 | 0.601967 | 0.013307 | -1.465819 | 0.604789 | -0.002546 | -1.466976 | 0.608968 | 0.000202 |
| C | -2.738961 | 0.135215 | -0.042074 | -2.737917 | 0.133580 | -0.060715 | -2.734979 | 0.133618 | -0.079893 | -2.729578 | 0.132767 | -0.081007 | -2.734855 | 0.133326 | -0.076234 | -2.735603 | 0.133339 | -0.065645 |
| O | -0.183285 | -1.479687 | -0.158425 | -0.182633 | -1.487942 | -0.146170 | -0.184571 | -1.500831 | -0.127053 | -0.187093 | -1.499463 | -0.123393 | -0.184859 | -1.496005 | -0.127509 | -0.183233 | -1.490101 | -0.127130 |
| C | -3.283861 | -1.223414 | 0.123040 | -3.290187 | -1.218487 | 0.123408 | -3.297379 | -1.207669 | 0.129487 | -3.294578 | -1.201650 | 0.146258 | -3.293279 | -1.210508 | 0.129964 | -3.290074 | -1.214985 | 0.129438 |
| C | -4.670725 | -1.148884 | -0.092975 | -4.673154 | -1.141337 | -0.114694 | -4.672572 | -1.129710 | -0.147180 | -4.657689 | -1.136478 | -0.182136 | -4.668173 | -1.136694 | -0.149839 | -4.667617 | -1.141792 | -0.137620 |
| N | -5.023312 | 0.154275 | -0.398748 | -5.014349 | 0.155557 | -0.455658 | -4.997778 | 0.156192 | -0.539886 | -4.969366 | 0.141189 | -0.622848 | -4.996606 | 0.149500 | -0.540386 | -5.002078 | 0.149441 | -0.507592 |
| C | -3.952799 | 1.002700 | -0.356873 | -3.941911 | 0.996431 | -0.421360 | -3.922226 | 0.988887 | -0.507800 | -3.898203 | 0.970416 | -0.574307 | -3.924756 | 0.985874 | -0.499810 | -3.931432 | 0.988732 | -0.464967 |
| C | -2.757411 | -2.448311 | 0.522490 | -2.775363 | -2.434544 | 0.563009 | -2.799796 | -2.406481 | 0.631790 | -2.811626 | -2.376181 | 0.714744 | -2.793114 | -2.408490 | 0.632318 | -2.783283 | -2.420497 | 0.606013 |
| C | -3.594824 | -3.547740 | 0.655918 | -3.619866 | -3.526632 | 0.710942 | -3.653928 | -3.489101 | 0.796555 | -3.668187 | -3.456052 | 0.882863 | -3.644321 | -3.494160 | 0.793351 | -3.630501 | -3.510297 | 0.757563 |
| C | -4.953735 | -3.446396 | 0.400857 | -4.974932 | -3.424635 | 0.431724 | -5.001534 | -3.390308 | 0.473908 | -5.002015 | -3.374413 | 0.502621 | -4.991412 | -3.399416 | 0.466910 | -4.979978 | -3.414308 | 0.446422 |
| C | -5.513883 | -2.232072 | 0.027277 | -5.523955 | -2.217588 | 0.019878 | -5.533563 | -2.196794 | 0.003368 | -5.520988 | -2.199768 | -0.027788 | -5.526140 | -2.206797 | -0.003430 | -5.521249 | -2.215759 | -0.000159 |
| O | -4.024765 | 2.184380 | -0.593400 | -3.996136 | 2.171881 | -0.700948 | -3.948929 | 2.152881 | -0.844154 | -3.904741 | 2.126224 | -0.955231 | -3.955972 | 2.153199 | -0.825720 | -3.973817 | 2.160482 | -0.769467 |
| C | 2.183390 | 0.067022 | -0.066350 | 2.185456 | 0.061823 | -0.056950 | 2.187217 | 0.053861 | -0.047629 | 2.185224 | 0.054889 | -0.044503 | 2.186346 | 0.057148 | -0.045146 | 2.185361 | 0.060967 | -0.047856 |
| C | 3.065841 | 0.478088 | 0.927107 | 3.048585 | 0.407434 | 0.976654 | 3.040887 | 0.357580 | 1.005771 | 3.042953 | 0.353235 | 1.006954 | 3.041192 | 0.359701 | 1.007793 | 3.046163 | 0.382838 | 0.994919 |
| C | 4.366134 | 0.011518 | 0.935025 | 4.349857 | -0.056061 | 0.973557 | 4.343846 | -0.101225 | 0.989075 | 4.344735 | -0.107449 | 0.982374 | 4.343475 | -0.101566 | 0.990560 | 4.347397 | -0.081037 | 0.982445 |
| C | 4.759350 | -0.877573 | -0.048255 | 4.761207 | -0.874950 | -0.062816 | 4.761995 | -0.872739 | -0.080997 | 4.755244 | -0.874453 | -0.094255 | 4.759791 | -0.873232 | -0.080254 | 4.758574 | -0.874944 | -0.073701 |
| C | 3.891908 | -1.311290 | -1.033960 | 3.912527 | -1.240168 | -1.092633 | 3.922305 | -1.192867 | -1.133840 | 3.911472 | -1.189226 | -1.145504 | 3.918988 | -1.192065 | -1.132676 | 3.912121 | -1.215019 | -1.114300 |
| C | 2.596520 | -0.830684 | -1.043456 | 2.616334 | -0.761876 | -1.089236 | 2.625106 | -0.717789 | -1.116311 | 2.615407 | -0.712220 | -1.119442 | 2.622208 | -0.715687 | -1.113982 | 2.616040 | -0.736640 | -1.100468 |
| N | 6.142403 | -1.379735 | -0.042252 | 6.143081 | -1.373686 | -0.067609 | 6.142105 | -1.367768 | -0.099411 | 6.130627 | -1.371074 | -0.121917 | 6.139100 | -1.370023 | -0.100589 | 6.138321 | -1.373497 | -0.089803 |
| O | 6.884077 | -0.970743 | 0.824906 | 6.875326 | -1.021676 | 0.833581 | 6.882331 | -1.029444 | 0.802401 | 6.881557 | -1.037892 | 0.773280 | 6.874911 | -1.052466 | 0.811971 | 6.872590 | -1.046741 | 0.819833 |
| O | 6.456477 | -2.170646 | -0.904780 | 6.477205 | -2.109156 | -0.972378 | 6.477624 | -2.091661 | -1.015307 | 6.463164 | -2.095257 | -1.038907 | 6.479492 | -2.074736 | -1.029199 | 6.476131 | -2.086369 | -1.011979 |
| H | -5.949471 | 0.482683 | -0.603748 | -5.938845 | 0.475123 | -0.688841 | -5.918055 | 0.460226 | -0.815513 | -5.873860 | 0.434927 | -0.957410 | -5.918172 | 0.454514 | -0.811044 | -5.923447 | 0.461752 | -0.766179 |
| H | -2.566952 | 3.357760 | 0.195595 | -2.567550 | 3.348585 | 0.212525 | -2.571026 | 3.330671 | 0.248581 | -2.581002 | 3.318526 | 0.323073 | -2.573329 | 3.335068 | 0.243797 | -2.571835 | 3.343782 | 0.232145 |
| H | -1.129951 | 5.347315 | 0.302835 | -1.131004 | 5.336047 | 0.362012 | -1.135487 | 5.318114 | 0.440596 | -1.151795 | 5.309908 | 0.507886 | -1.138403 | 5.323884 | 0.429489 | -1.136945 | 5.332262 | 0.394021 |
| H | 1.322322 | 5.130872 | 0.150046 | 1.323183 | 5.119442 | 0.236351 | 1.320351 | 5.102873 | 0.332796 | 1.302711 | 5.107074 | 0.368836 | 1.317649 | 5.109154 | 0.323374 | 1.318196 | 5.117812 | 0.276474 |
| H | 2.368491 | 2.895731 | -0.030460 | 2.371509 | 2.886558 | 0.042193 | 2.373656 | 2.874564 | 0.118327 | 2.366529 | 2.885843 | 0.130966 | 2.371572 | 2.880032 | 0.115895 | 2.370441 | 2.886746 | 0.079092 |
| H | -1.703944 | -2.550582 | 0.712231 | -1.725494 | -2.536084 | 0.774588 | -1.757297 | -2.501564 | 0.882886 | -1.782320 | -2.454562 | 1.023197 | -1.751082 | -2.500300 | 0.886872 | -1.738516 | -2.515742 | 0.846326 |
| H | -6.576518 | -2.134776 | -0.153482 | -6.583223 | -2.118304 | -0.179109 | -6.586349 | -2.096818 | -0.227047 | -6.565316 | -2.112664 | -0.298392 | -6.578639 | -2.109964 | -0.236354 | -6.576186 | -2.119768 | -0.222467 |
| H | -3.173846 | -4.497066 | 0.958833 | -3.209003 | -4.470356 | 1.044819 | -3.259705 | -4.421632 | 1.179571 | -3.287828 | -4.372080 | 1.316455 | -3.248227 | -4.425787 | 1.176552 | -3.227461 | -4.446959 | 1.120213 |
| H | -5.588978 | -4.317288 | 0.501242 | -5.616103 | -4.290013 | 0.544010 | -5.650630 | -4.248008 | 0.600706 | -5.652700 | -4.230225 | 0.634264 | -5.638013 | -4.259399 | 0.590770 | -5.623212 | -4.277860 | 0.562118 |
| H | 2.727742 | 1.155715 | 1.699211 | 2.696820 | 1.031795 | 1.786847 | 2.683732 | 0.947078 | 1.839439 | 2.690028 | 0.938908 | 1.845161 | 2.685471 | 0.950427 | 1.841153 | 2.693905 | 0.989367 | 1.818392 |
| H | 1.899654 | -1.155095 | -1.802094 | 1.936291 | -1.026995 | -1.885934 | 1.954034 | -0.940136 | -1.933958 | 1.939148 | -0.929536 | -1.934587 | 1.949563 | -0.937919 | -1.930337 | 1.937997 | -0.979386 | -1.906326 |
| H | 5.068517 | 0.316388 | 1.696462 | 5.034401 | 0.200626 | 1.768209 | 5.018838 | 0.126561 | 1.800645 | 5.022841 | 0.114359 | 1.793156 | 5.019577 | 0.124760 | 1.801580 | 5.028960 | 0.157758 | 1.785391 |
| H | 4.236154 | -2.009133 | -1.782658 | 4.265510 | -1.881489 | -1.886524 | 4.278139 | -1.792897 | -1.958352 | 4.262624 | -1.785669 | -1.974693 | 4.274045 | -1.792690 | -1.956939 | 4.264356 | -1.835917 | -1.924622 |

| **iso-I** 18 |  | gasphase |  |  | toluene |  |  | DMSO |  |  | methanol |  |  | acetonitrile |  |  | chloroform |  |
| --- | --- | --- | --- | --- | --- | --- | --- | --- | --- | --- | --- | --- | --- | --- | --- | --- | --- | --- |
| Element | x | y | z | x | y | z | x | y | z | x | y | z | x | y | z | x | y | z |
| C | 0.407894 | -2.870759 | -0.924274 | 0.396452 | -2.868035 | -0.845759 | 0.407155 | -2.844035 | -0.939184 | 0.410367 | -2.831878 | -1.018127 | 0.405080 | -2.844428 | -0.947802 | 0.410628 | -2.854529 | -0.974895 |
| C | 1.145971 | -4.038586 | -1.053150 | 1.130606 | -4.035130 | -1.000752 | 1.144918 | -4.006954 | -1.110108 | 1.149810 | -3.993139 | -1.192267 | 1.141957 | -4.008235 | -1.117816 | 1.148662 | -4.020856 | -1.118871 |
| C | 2.518228 | -4.036573 | -0.860428 | 2.506955 | -4.035518 | -0.833781 | 2.519968 | -4.009286 | -0.918677 | 2.521500 | -3.999774 | -0.977418 | 2.516750 | -4.012097 | -0.923575 | 2.519870 | -4.023543 | -0.907523 |
| C | 3.189125 | -2.862139 | -0.545957 | 3.184877 | -2.864177 | -0.522697 | 3.193497 | -2.844906 | -0.572221 | 3.192024 | -2.840857 | -0.605860 | 3.190883 | -2.848395 | -0.575163 | 3.189853 | -2.855686 | -0.565744 |
| C | 2.441532 | -1.711103 | -0.413524 | 2.440695 | -1.713482 | -0.366821 | 2.444758 | -1.698584 | -0.402654 | 2.440975 | -1.697937 | -0.431631 | 2.443053 | -1.701358 | -0.406240 | 2.441309 | -1.706140 | -0.421767 |
| C | 1.048626 | -1.684347 | -0.582666 | 1.044372 | -1.685120 | -0.505147 | 1.050865 | -1.672151 | -0.556095 | 1.050300 | -1.668857 | -0.603963 | 1.049420 | -1.673487 | -0.562502 | 1.049970 | -1.677828 | -0.599010 |
| N | 2.919351 | -0.417623 | -0.181007 | 2.922657 | -0.422739 | -0.130253 | 2.918034 | -0.410289 | -0.137863 | 2.909394 | -0.409099 | -0.147217 | 2.917052 | -0.413040 | -0.140296 | 2.914511 | -0.414962 | -0.166404 |
| C | 1.888792 | 0.503602 | -0.254553 | 1.891166 | 0.495825 | -0.154799 | 1.884680 | 0.503449 | -0.152535 | 1.879047 | 0.499217 | -0.169944 | 1.884588 | 0.501192 | -0.158477 | 1.884455 | 0.502293 | -0.221684 |
| C | 0.614931 | -0.286288 | -0.474571 | 0.614234 | -0.287866 | -0.376307 | 0.616101 | -0.281405 | -0.407856 | 0.615867 | -0.280522 | -0.447340 | 0.615763 | -0.282221 | -0.414957 | 0.615579 | -0.283724 | -0.471474 |
| C | -0.614829 | 0.287240 | -0.474529 | -0.614238 | 0.287833 | -0.376312 | -0.616094 | 0.281410 | -0.407831 | -0.615739 | 0.281647 | -0.447255 | -0.615721 | 0.282206 | -0.414790 | -0.615581 | 0.283732 | -0.471481 |
| O | 2.037399 | 1.688120 | -0.087834 | 2.037733 | 1.675255 | 0.054856 | 2.015699 | 1.677472 | 0.103626 | 2.002995 | 1.674898 | 0.102676 | 2.016067 | 1.675763 | 0.096215 | 2.020869 | 1.683359 | -0.007234 |
| C | -1.048884 | 1.685224 | -0.582459 | -1.044364 | 1.685089 | -0.505159 | -1.050861 | 1.672156 | -0.556067 | -1.050596 | 1.669899 | -0.603695 | -1.049382 | 1.673471 | -0.562320 | -1.049974 | 1.677836 | -0.599015 |
| C | -2.441792 | 1.711573 | -0.413175 | -2.440686 | 1.713465 | -0.366833 | -2.444754 | 1.698587 | -0.402618 | -2.441266 | 1.698513 | -0.431139 | -2.443011 | 1.701359 | -0.406009 | -2.441313 | 1.706145 | -0.421775 |
| N | -2.919269 | 0.417919 | -0.180920 | -2.922660 | 0.422728 | -0.130260 | -2.918026 | 0.410295 | -0.137808 | -2.909285 | 0.409458 | -0.147047 | -2.917002 | 0.413070 | -0.139914 | -2.914514 | 0.414965 | -0.166421 |
| C | -1.888493 | -0.503027 | -0.254832 | -1.891179 | -0.495848 | -0.154823 | -1.884662 | -0.503432 | -0.152414 | -1.878688 | -0.498552 | -0.170215 | -1.884477 | -0.501103 | -0.157639 | -1.884460 | -0.502291 | -0.221725 |
| C | -0.408564 | 2.871872 | -0.924053 | -0.396431 | 2.867996 | -0.845772 | -0.407159 | 2.844034 | -0.939185 | -0.411164 | 2.833186 | -1.017917 | -0.405067 | 2.844358 | -0.947822 | -0.410632 | 2.854540 | -0.974889 |
| C | -1.146996 | 4.039505 | -1.052669 | -1.130573 | 4.035099 | -1.000767 | -1.144926 | 4.006948 | -1.110124 | -1.151023 | 3.994233 | -1.191725 | -1.141951 | 4.008144 | -1.117946 | -1.148667 | 4.020868 | -1.118858 |
| C | -2.519218 | 4.037087 | -0.859730 | -2.506922 | 4.035502 | -0.833796 | -2.519974 | 4.009279 | -0.918685 | -2.522657 | 4.000413 | -0.976539 | -2.516739 | 4.012025 | -0.923661 | -2.519876 | 4.023552 | -0.907512 |
| C | -3.189729 | 2.862415 | -0.545340 | -3.184857 | 2.864168 | -0.522709 | -3.193498 | 2.844903 | -0.572208 | -3.192721 | 2.841219 | -0.605035 | -3.190857 | 2.848361 | -0.575097 | -3.189858 | 2.855691 | -0.565742 |
| O | -2.036751 | -1.687653 | -0.088558 | -2.037754 | -1.675268 | 0.054884 | -2.015685 | -1.677467 | 0.103690 | -2.002246 | -1.674428 | 0.101753 | -2.016014 | -1.675749 | 0.096681 | -2.020869 | -1.683353 | -0.007253 |
| C | 4.248273 | -0.075920 | 0.150911 | 4.264525 | -0.080841 | 0.151233 | 4.256158 | -0.072756 | 0.167877 | 4.243343 | -0.071795 | 0.182533 | 4.254367 | -0.074873 | 0.169242 | 4.245369 | -0.074990 | 0.166491 |
| C | 4.901260 | 0.935963 | -0.541440 | 4.900076 | 0.899739 | -0.599628 | 4.905392 | 0.905989 | -0.573941 | 4.901163 | 0.912854 | -0.543179 | 4.903385 | 0.907691 | -0.567998 | 4.907725 | 0.910106 | -0.555033 |
| C | 6.198189 | 1.272234 | -0.206596 | 6.208943 | 1.239035 | -0.319457 | 6.210162 | 1.242356 | -0.271410 | 6.200449 | 1.249587 | -0.217655 | 6.207178 | 1.245253 | -0.261895 | 6.204980 | 1.248487 | -0.223571 |
| C | 6.853299 | 0.591433 | 0.816733 | 6.888780 | 0.588109 | 0.707709 | 6.866333 | 0.587010 | 0.768807 | 6.840630 | 0.588037 | 0.828261 | 6.862686 | 0.587470 | 0.777385 | 6.843784 | 0.590566 | 0.825470 |
| C | 6.195916 | -0.421070 | 1.511300 | 6.250426 | -0.395529 | 1.459840 | 6.215945 | -0.397653 | 1.509722 | 6.180835 | -0.402547 | 1.552690 | 6.212295 | -0.400596 | 1.514022 | 6.179295 | -0.398596 | 1.547516 |
| C | 4.894660 | -0.746341 | 1.183243 | 4.937222 | -0.722196 | 1.184563 | 4.907060 | -0.720854 | 1.210702 | 4.877319 | -0.726129 | 1.231368 | 4.904320 | -0.724972 | 1.211563 | 4.877602 | -0.723839 | 1.220689 |
| C | 8.202483 | 0.933169 | 1.156620 | 8.249738 | 0.931655 | 0.991538 | 8.221119 | 0.927419 | 1.077873 | 8.189583 | 0.928081 | 1.160537 | 8.216700 | 0.928857 | 1.089952 | 8.191907 | 0.932656 | 1.163582 |
| N | 9.284956 | 1.204984 | 1.428282 | 9.342096 | 1.205331 | 1.217696 | 9.309503 | 1.200471 | 1.325591 | 9.273718 | 1.200699 | 1.426921 | 9.304368 | 1.202520 | 1.340374 | 9.274708 | 1.205551 | 1.433912 |
| C | -4.248087 | 0.075789 | 0.150974 | -4.264530 | 0.080843 | 0.151230 | -4.256155 | 0.072759 | 0.167907 | -4.243118 | 0.071646 | 0.182654 | -4.254356 | 0.074902 | 0.169446 | -4.245369 | 0.074989 | 0.166482 |
| C | -4.894592 | 0.745766 | 1.183522 | -4.937213 | 0.722196 | 1.184570 | -4.907094 | 0.720891 | 1.210688 | -4.877245 | 0.725436 | 1.231736 | -4.904554 | 0.725245 | 1.211462 | -4.877593 | 0.723822 | 1.220696 |
| C | -6.195748 | 0.420076 | 1.511556 | -6.250421 | 0.395545 | 1.459849 | -6.215984 | 0.397687 | 1.509684 | -6.180650 | 0.401354 | 1.553005 | -6.212567 | 0.400864 | 1.513751 | -6.179283 | 0.398574 | 1.547529 |
| C | -6.852909 | -0.592407 | 0.816749 | -6.888792 | -0.588075 | 0.707710 | -6.866339 | -0.587014 | 0.768789 | -6.840177 | -0.589186 | 0.828272 | -6.862748 | -0.587448 | 0.777259 | -6.843779 | -0.590577 | 0.825475 |
| C | -6.197677 | -1.272765 | -0.206797 | -6.208969 | -1.238999 | -0.319467 | -6.210131 | -1.242393 | -0.271383 | -6.199843 | -1.250193 | -0.217893 | -6.206991 | -1.245474 | -0.261710 | -6.204984 | -1.248482 | -0.223582 |
| C | -4.900851 | -0.936073 | -0.541616 | -4.900098 | -0.899719 | -0.599640 | -4.905356 | -0.906023 | -0.573890 | -4.900672 | -0.912960 | -0.543359 | -4.903163 | -0.907907 | -0.567652 | -4.907732 | -0.910096 | -0.555051 |
| C | -8.201989 | -0.934578 | 1.156612 | -8.249754 | -0.931604 | 0.991541 | -8.221130 | -0.927425 | 1.077830 | -8.189013 | -0.929749 | 1.160492 | -8.216802 | -0.928838 | 1.089650 | -8.191898 | -0.932672 | 1.163595 |
| N | -9.284379 | -1.206740 | 1.428255 | -9.342115 | -1.205267 | 1.217702 | -9.309518 | -1.200479 | 1.325526 | -9.273055 | -1.202782 | 1.426832 | -9.304501 | -1.202501 | 1.339934 | -9.274696 | -1.205572 | 1.433931 |
| H | -0.657261 | -2.887368 | -1.072456 | -0.671574 | -2.883593 | -0.974731 | -0.658287 | -2.854625 | -1.095526 | -0.650931 | -2.836379 | -1.204866 | -0.660026 | -2.853258 | -1.106921 | -0.652392 | -2.865891 | -1.144580 |
| H | 0.638944 | -4.959405 | -1.308344 | 0.617733 | -4.953463 | -1.254616 | 0.639287 | -4.919660 | -1.397924 | 0.648900 | -4.900760 | -1.503580 | 0.635863 | -4.920192 | -1.407127 | 0.644606 | -4.936205 | -1.400335 |
| H | 3.080598 | -4.955427 | -0.966678 | 3.066295 | -4.954265 | -0.957506 | 3.082143 | -4.924885 | -1.053935 | 3.084509 | -4.914310 | -1.116818 | 3.078268 | -4.928182 | -1.058177 | 3.082147 | -4.941749 | -1.022543 |
| H | 4.263554 | -2.849097 | -0.428198 | 4.261243 | -2.854992 | -0.422467 | 4.268327 | -2.838591 | -0.453923 | 4.265382 | -2.836709 | -0.473963 | 4.265441 | -2.843077 | -0.454738 | 4.262857 | -2.848795 | -0.432705 |
| H | 0.656549 | 2.888829 | -1.072423 | 0.671595 | 2.883540 | -0.974743 | 0.658281 | 2.854621 | -1.095543 | 0.650066 | 2.838073 | -1.204950 | 0.660025 | 2.853155 | -1.107037 | 0.652388 | 2.865905 | -1.144570 |
| H | -4.264143 | 2.849042 | -0.427476 | -4.261223 | 2.854996 | -0.422479 | -4.268328 | 2.838585 | -0.453906 | -4.266057 | 2.836686 | -0.472977 | -4.265413 | 2.843048 | -0.454652 | -4.262862 | 2.848799 | -0.432705 |
| H | -0.640270 | 4.960498 | -1.307835 | -0.617690 | 4.953426 | -1.254632 | -0.639300 | 4.919649 | -1.397962 | -0.650479 | 4.902052 | -1.503051 | -0.635873 | 4.920060 | -1.407411 | -0.644611 | 4.936220 | -1.400313 |
| H | -3.081867 | 4.955793 | -0.965786 | -3.066252 | 4.954254 | -0.957523 | -3.082153 | 4.924874 | -1.053958 | -3.085996 | 4.914783 | -1.115699 | -3.078266 | 4.928088 | -1.058373 | -3.082152 | 4.941759 | -1.022526 |
| H | 4.388992 | 1.460773 | -1.334332 | 4.368366 | 1.395152 | -1.399392 | 4.391914 | 1.397014 | -1.388649 | 4.398887 | 1.407922 | -1.362951 | 4.390012 | 1.401002 | -1.381414 | 4.407790 | 1.408151 | -1.373567 |
| H | 4.371721 | -1.516974 | 1.733533 | 4.428118 | -1.471316 | 1.776102 | 4.387597 | -1.473287 | 1.788926 | 4.349917 | -1.483361 | 1.796182 | 4.385055 | -1.480077 | 1.786425 | 4.347847 | -1.477739 | 1.787514 |
| H | 6.711289 | 2.059800 | -0.741368 | 6.709212 | 2.002782 | -0.899302 | 6.723826 | 2.002729 | -0.844061 | 6.722116 | 2.014895 | -0.776519 | 6.720563 | 2.008528 | -0.830912 | 6.726915 | 2.015306 | -0.779900 |
| H | 6.702618 | -0.939957 | 2.313547 | 6.779081 | -0.891104 | 2.262817 | 6.730246 | -0.897435 | 2.319411 | 6.683803 | -0.906564 | 2.367006 | 6.725827 | -0.902081 | 2.323142 | 6.677602 | -0.899509 | 2.366508 |
| H | -4.371821 | 1.516382 | 1.733994 | -4.428096 | 1.471303 | 1.776114 | -4.387656 | 1.473353 | 1.788896 | -4.350046 | 1.482637 | 1.796782 | -4.385454 | 1.480548 | 1.786212 | -4.347832 | 1.477713 | 1.787528 |
| H | -4.388489 | -1.460540 | -1.334674 | -4.368400 | -1.395130 | -1.399413 | -4.391850 | -1.397074 | -1.388565 | -4.398281 | -1.407602 | -1.363317 | -4.389595 | -1.401407 | -1.380833 | -4.407804 | -1.408129 | -1.373596 |
| H | -6.702541 | 0.938616 | 2.313969 | -6.779065 | 0.891119 | 2.262834 | -6.730313 | 0.897495 | 2.319338 | -6.683734 | 0.904945 | 2.367512 | -6.726293 | 0.902541 | 2.322630 | -6.677581 | 0.899475 | 2.366534 |
| H | -6.710604 | -2.060317 | -0.741756 | -6.709252 | -2.002732 | -0.899318 | -6.723770 | -2.002795 | -0.844018 | -6.721303 | -2.015472 | -0.776989 | -6.720214 | -2.008941 | -0.830618 | -6.726924 | -2.015292 | -0.779917 |

| **iso-I** 19 |  | gasphase |  |  | toluene |  |  | DMSO |  |  | methanol |  |  | acetonitrile |  |  | chloroform |  |
| --- | --- | --- | --- | --- | --- | --- | --- | --- | --- | --- | --- | --- | --- | --- | --- | --- | --- | --- |
| Element | x | y | z | x | y | z | x | y | z | x | y | z | x | y | z | x | y | z |
| C | -0.480493 | 2.922523 | -0.474112 | -0.492296 | 2.913203 | -0.515103 | -0.504310 | 2.889685 | -0.671050 | -0.497429 | 2.872165 | -0.795057 | -0.504221 | 2.895256 | -0.667333 | -0.494412 | 2.901556 | -0.623835 |
| C | -1.193225 | 4.113679 | -0.498906 | -1.208353 | 4.101623 | -0.560373 | -1.221823 | 4.077133 | -0.725070 | -1.211525 | 4.061557 | -0.846991 | -1.222598 | 4.082395 | -0.721297 | -1.209153 | 4.091172 | -0.663960 |
| C | -2.564663 | 4.126122 | -0.305477 | -2.581641 | 4.111183 | -0.373773 | -2.591633 | 4.089231 | -0.502126 | -2.574301 | 4.083071 | -0.583665 | -2.592854 | 4.093381 | -0.500616 | -2.578233 | 4.104658 | -0.442922 |
| C | -3.263162 | 2.941069 | -0.114132 | -3.277459 | 2.926688 | -0.169239 | -3.283341 | 2.909912 | -0.255032 | -3.265682 | 2.910901 | -0.301333 | -3.283963 | 2.913143 | -0.255181 | -3.272302 | 2.923657 | -0.211227 |
| C | -2.545101 | 1.763856 | -0.115345 | -2.554392 | 1.752482 | -0.147050 | -2.557803 | 1.737006 | -0.223562 | -2.543722 | 1.736900 | -0.272339 | -2.557656 | 1.740739 | -0.223454 | -2.550542 | 1.748469 | -0.194924 |
| C | -1.148928 | 1.721619 | -0.260133 | -1.157645 | 1.713887 | -0.283793 | -1.165048 | 1.698910 | -0.389394 | -1.156567 | 1.691814 | -0.470680 | -1.164518 | 1.703397 | -0.387978 | -1.157377 | 1.707598 | -0.360553 |
| N | -3.047930 | 0.468664 | 0.036222 | -3.052521 | 0.456752 | 0.010517 | -3.048052 | 0.441809 | -0.043293 | -3.032383 | 0.441416 | -0.067578 | -3.047586 | 0.445041 | -0.042941 | -3.045604 | 0.452912 | -0.023930 |
| C | -2.025604 | -0.462545 | -0.023572 | -2.031243 | -0.471267 | -0.049265 | -2.032676 | -0.485460 | -0.134676 | -2.023548 | -0.483430 | -0.170268 | -2.031257 | -0.480848 | -0.130253 | -2.029524 | -0.475418 | -0.110631 |
| C | -0.739107 | 0.310050 | -0.218975 | -0.744732 | 0.304405 | -0.233711 | -0.750011 | 0.293643 | -0.332405 | -0.746309 | 0.287973 | -0.404055 | -0.749118 | 0.297888 | -0.330938 | -0.745511 | 0.299548 | -0.310231 |
| C | 0.476354 | -0.292155 | -0.276595 | 0.475049 | -0.289402 | -0.274223 | 0.474756 | -0.286414 | -0.356708 | 0.476170 | -0.294296 | -0.431403 | 0.474775 | -0.284172 | -0.358540 | 0.475363 | -0.290314 | -0.345540 |
| O | -2.198068 | -1.647904 | 0.116475 | -2.200190 | -1.659057 | 0.086110 | -2.195381 | -1.676779 | -0.005883 | -2.179901 | -1.676920 | -0.014132 | -2.192181 | -1.672109 | 0.004030 | -2.195941 | -1.665574 | 0.019707 |
| C | 0.870257 | -1.696088 | -0.457441 | 0.879961 | -1.688040 | -0.467386 | 0.891885 | -1.677169 | -0.562113 | 0.888462 | -1.682698 | -0.646829 | 0.889580 | -1.676137 | -0.562667 | 0.882843 | -1.686396 | -0.541863 |
| C | 2.269463 | -1.755097 | -0.354438 | 2.277775 | -1.739003 | -0.344460 | 2.284704 | -1.723920 | -0.396134 | 2.276862 | -1.739623 | -0.458370 | 2.282401 | -1.725030 | -0.396535 | 2.277361 | -1.739747 | -0.390247 |
| N | 2.787419 | -0.487708 | -0.095920 | 2.782451 | -0.472300 | -0.062422 | 2.771529 | -0.459589 | -0.074972 | 2.762354 | -0.476799 | -0.112834 | 2.771344 | -0.460316 | -0.078066 | 2.775665 | -0.474181 | -0.087878 |
| C | 1.785432 | 0.456115 | -0.078344 | 1.775726 | 0.461956 | -0.035136 | 1.760104 | 0.467144 | -0.059527 | 1.756126 | 0.448105 | -0.108957 | 1.761662 | 0.467556 | -0.066299 | 1.769492 | 0.458935 | -0.077555 |
| C | 0.193138 | -2.860665 | -0.805819 | 0.216215 | -2.852520 | -0.840692 | 0.243430 | -2.832007 | -0.987278 | 0.239112 | -2.820844 | -1.112178 | 0.239369 | -2.830352 | -0.987344 | 0.224157 | -2.843285 | -0.945847 |
| C | 0.902785 | -4.037344 | -1.001744 | 0.936575 | -4.023030 | -1.036443 | 0.973864 | -3.996970 | -1.183173 | 0.965516 | -3.986674 | -1.315784 | 0.967972 | -3.996912 | -1.181542 | 0.946341 | -4.013533 | -1.138627 |
| C | 2.281781 | -4.067933 | -0.863893 | 2.313560 | -4.047106 | -0.872165 | 2.345531 | -4.020629 | -0.969768 | 2.332552 | -4.023390 | -1.075440 | 2.339576 | -4.022779 | -0.967080 | 2.319799 | -4.042055 | -0.942800 |
| C | 2.988511 | -2.916249 | -0.544408 | 3.007847 | -2.894084 | -0.529963 | 3.025156 | -2.872949 | -0.580531 | 3.013893 | -2.887968 | -0.652826 | 3.021031 | -2.875541 | -0.579090 | 3.009389 | -2.894139 | -0.572909 |
| O | 1.972175 | 1.630575 | 0.125930 | 1.947846 | 1.632068 | 0.211112 | 1.911406 | 1.632103 | 0.231897 | 1.903135 | 1.613620 | 0.202924 | 1.915650 | 1.634296 | 0.218245 | 1.934091 | 1.628799 | 0.183388 |
| C | -4.409557 | 0.116650 | 0.163844 | -4.414702 | 0.105708 | 0.155046 | -4.404848 | 0.094873 | 0.163598 | -4.384009 | 0.098830 | 0.185062 | -4.404250 | 0.096133 | 0.163648 | -4.403983 | 0.103885 | 0.164963 |
| C | -4.820986 | -0.690376 | 1.216830 | -4.824556 | -0.629914 | 1.259233 | -4.769531 | -0.577482 | 1.322445 | -4.712522 | -0.558672 | 1.362851 | -4.768547 | -0.575590 | 1.323132 | -4.779007 | -0.606387 | 1.297683 |
| C | -6.151334 | -1.042041 | 1.335200 | -6.153495 | -0.980094 | 1.396513 | -6.090808 | -0.924267 | 1.526054 | -6.027338 | -0.901862 | 1.610618 | -6.089465 | -0.924876 | 1.526078 | -6.102781 | -0.955197 | 1.481442 |
| C | -7.079724 | -0.580109 | 0.405171 | -7.076965 | -0.583837 | 0.431442 | -7.045372 | -0.584213 | 0.569331 | -7.009597 | -0.572320 | 0.678965 | -7.044073 | -0.588019 | 0.568083 | -7.052449 | -0.581265 | 0.532771 |
| C | -6.664692 | 0.225740 | -0.652247 | -6.664439 | 0.153721 | -0.676147 | -6.679196 | 0.093901 | -0.591455 | -6.678868 | 0.091286 | -0.500654 | -6.678168 | 0.089297 | -0.593438 | -6.675146 | 0.131998 | -0.603159 |
| C | -5.331457 | 0.564420 | -0.775324 | -5.332197 | 0.489794 | -0.815330 | -5.353867 | 0.426541 | -0.794718 | -5.360229 | 0.419943 | -0.748790 | -5.353200 | 0.424371 | -0.796075 | -5.347781 | 0.466689 | -0.787841 |
| C | -8.461774 | -0.935609 | 0.533519 | -8.457468 | -0.936766 | 0.576950 | -8.416606 | -0.934505 | 0.782296 | -8.373562 | -0.919235 | 0.936809 | -8.415059 | -0.940834 | 0.780359 | -8.426962 | -0.932504 | 0.726356 |
| N | -9.570432 | -1.218050 | 0.636084 | -9.565153 | -1.217446 | 0.693346 | -9.517627 | -1.215166 | 0.953475 | -9.469131 | -1.197244 | 1.143913 | -9.515677 | -1.223478 | 0.951168 | -9.530275 | -1.212487 | 0.881895 |
| C | 4.155592 | -0.195638 | 0.139810 | 4.151256 | -0.174383 | 0.175847 | 4.131748 | -0.160358 | 0.209233 | 4.114363 | -0.179835 | 0.211411 | 4.131728 | -0.160932 | 0.207327 | 4.137963 | -0.176847 | 0.187044 |
| C | 4.840995 | 0.671346 | -0.689399 | 4.881427 | 0.541516 | -0.753601 | 4.901589 | 0.522962 | -0.713839 | 4.870406 | 0.618185 | -0.627558 | 4.906016 | 0.511427 | -0.720384 | 4.882379 | 0.571311 | -0.705700 |
| C | 6.177037 | 0.963380 | -0.454886 | 6.218359 | 0.835153 | -0.525837 | 6.231245 | 0.816046 | -0.444175 | 6.189119 | 0.916860 | -0.314725 | 6.235593 | 0.804241 | -0.449363 | 6.212273 | 0.866855 | -0.440715 |
| C | 6.836452 | 0.367928 | 0.614792 | 6.830794 | 0.393528 | 0.643072 | 6.795354 | 0.406312 | 0.761552 | 6.753243 | 0.396272 | 0.845838 | 6.794482 | 0.405681 | 0.762465 | 6.802568 | 0.393909 | 0.727646 |
| C | 6.144154 | -0.509630 | 1.448869 | 6.092555 | -0.334720 | 1.577045 | 6.016042 | -0.289498 | 1.688442 | 5.989835 | -0.414427 | 1.686883 | 6.010596 | -0.277829 | 1.694679 | 6.050086 | -0.366868 | 1.623996 |
| C | 4.813093 | -0.779798 | 1.217484 | 4.761891 | -0.609729 | 1.346684 | 4.692985 | -0.566015 | 1.414765 | 4.676443 | -0.694901 | 1.373908 | 4.687731 | -0.554359 | 1.419484 | 4.726073 | -0.643399 | 1.357519 |
| O | 8.134339 | 0.577057 | 0.924937 | 8.122265 | 0.619161 | 0.958495 | 8.074702 | 0.633866 | 1.117298 | 8.027526 | 0.620629 | 1.238246 | 8.073720 | 0.633226 | 1.120246 | 8.086008 | 0.618345 | 1.076791 |
| C | 8.880337 | 1.463032 | 0.123193 | 8.914989 | 1.362762 | 0.057467 | 8.908951 | 1.336628 | 0.212562 | 8.851332 | 1.439553 | 0.423448 | 8.915624 | 1.322878 | 0.212357 | 8.896035 | 1.389762 | 0.210902 |
| H | 0.587957 | 2.929139 | -0.596040 | 0.576315 | 2.922570 | -0.638450 | 0.559861 | 2.895036 | -0.834202 | 0.559876 | 2.869422 | -1.001916 | 0.560254 | 2.901400 | -0.828711 | 0.570491 | 2.907246 | -0.779655 |
| H | -0.662653 | 5.042886 | -0.658349 | -0.680755 | 5.030600 | -0.732573 | -0.702121 | 5.002457 | -0.938142 | -0.695359 | 4.980991 | -1.091825 | -0.703056 | 5.008223 | -0.932466 | -0.685402 | 5.017530 | -0.861161 |
| H | -3.103824 | 5.064895 | -0.305699 | -3.124524 | 5.047856 | -0.392176 | -3.135894 | 5.024996 | -0.531219 | -3.114700 | 5.021163 | -0.611680 | -3.137789 | 5.028733 | -0.529797 | -3.119791 | 5.042253 | -0.458308 |
| H | -4.333788 | 2.944223 | 0.034537 | -4.349945 | 2.928389 | -0.033095 | -4.353789 | 2.914671 | -0.101705 | -4.331943 | 2.922877 | -0.120788 | -4.354570 | 2.916614 | -0.103176 | -4.342006 | 2.929044 | -0.053755 |
| H | -0.877367 | -2.855943 | -0.908225 | -0.852515 | -2.852955 | -0.964046 | -0.819970 | -2.829000 | -1.155489 | -0.818916 | -2.803992 | -1.315058 | -0.823858 | -2.825586 | -1.156955 | -0.841243 | -2.837747 | -1.098752 |
| H | 4.066551 | -2.921274 | -0.461161 | 4.084172 | -2.894600 | -0.422864 | 4.097539 | -2.875293 | -0.437166 | 4.084111 | -2.901010 | -0.494793 | 4.093337 | -2.879147 | -0.435392 | 4.083338 | -2.899692 | -0.443388 |
| H | 0.366754 | -4.940074 | -1.262612 | 0.411012 | -4.926465 | -1.316808 | 0.463178 | -4.895596 | -1.504701 | 0.456633 | -4.874916 | -1.667675 | 0.455875 | -4.894918 | -1.502489 | 0.426312 | -4.912612 | -1.442887 |
| H | 2.819502 | -4.995262 | -1.016154 | 2.859053 | -4.970610 | -1.021433 | 2.898944 | -4.939466 | -1.119672 | 2.882744 | -4.942793 | -1.233780 | 2.891456 | -4.942771 | -1.115412 | 2.866846 | -4.964979 | -1.090547 |
| H | -4.095283 | -1.046281 | 1.933439 | -4.101881 | -0.927752 | 2.005539 | -4.020636 | -0.822775 | 2.062679 | -3.940583 | -0.795956 | 2.082253 | -4.019577 | -0.818883 | 2.063936 | -4.034733 | -0.883399 | 2.031055 |
| H | -4.998918 | 1.171091 | -1.606835 | -4.998592 | 1.045026 | -1.681870 | -5.053238 | 0.940478 | -1.698123 | -5.087010 | 0.921975 | -1.667525 | -5.052787 | 0.937847 | -1.699793 | -5.040022 | 1.005236 | -1.674388 |
| H | -6.477451 | -1.671019 | 2.152248 | -6.479811 | -1.553831 | 2.253367 | -6.385589 | -1.448085 | 2.425350 | -6.295396 | -1.415085 | 2.524399 | -6.383916 | -1.448291 | 2.425703 | -6.403924 | -1.508941 | 2.360460 |
| H | -7.385829 | 0.572334 | -1.379883 | -7.383754 | 0.450122 | -1.427440 | -7.426150 | 0.348576 | -1.331039 | -7.448475 | 0.337382 | -1.219855 | -7.425020 | 0.341331 | -1.334016 | -7.416610 | 0.411288 | -1.339389 |
| H | 4.326517 | 1.134338 | -1.520506 | 4.402707 | 0.880066 | -1.663484 | 4.462113 | 0.832462 | -1.653928 | 4.429280 | 1.013994 | -1.533863 | 4.469915 | 0.812414 | -1.664818 | 4.421580 | 0.932941 | -1.616164 |
| H | 4.272294 | -1.449339 | 1.874390 | 4.185865 | -1.165118 | 2.076307 | 4.087418 | -1.101228 | 2.135818 | 4.080990 | -1.317357 | 2.030353 | 4.078317 | -1.080126 | 2.144189 | 4.139369 | -1.224835 | 2.057870 |
| H | 6.687312 | 1.650839 | -1.113470 | 6.765737 | 1.401831 | -1.265067 | 6.811247 | 1.354666 | -1.179741 | 6.759849 | 1.546039 | -0.982449 | 6.819891 | 1.333850 | -1.188020 | 6.772033 | 1.458645 | -1.150539 |
| H | 6.668327 | -0.958596 | 2.281817 | 6.580203 | -0.667445 | 2.483968 | 6.463948 | -0.602498 | 2.623024 | 6.440923 | -0.811182 | 2.587701 | 6.455027 | -0.581007 | 2.634148 | 6.520153 | -0.725193 | 2.530781 |
| H | 8.936298 | 1.114644 | -0.912761 | 8.997791 | 0.861646 | -0.911741 | 9.014089 | 0.797590 | -0.732994 | 8.980039 | 1.004778 | -0.571166 | 9.022340 | 0.773064 | -0.726692 | 8.998052 | 0.914398 | -0.768917 |
| H | 9.881164 | 1.485358 | 0.547538 | 9.903026 | 1.429520 | 0.508282 | 9.881887 | 1.408760 | 0.694070 | 9.815819 | 1.485308 | 0.924651 | 9.886063 | 1.395356 | 0.698637 | 9.873984 | 1.445611 | 0.684401 |
| H | 8.458037 | 2.472353 | 0.143157 | 8.518705 | 2.372447 | -0.085895 | 8.527564 | 2.342928 | 0.018943 | 8.440815 | 2.448675 | 0.334855 | 8.538902 | 2.328436 | 0.006594 | 8.498057 | 2.401043 | 0.088183 |

| **iso-I** 20 |  | gasphase |  |  | toluene |  |  | DMSO |  |  | methanol |  |  | acetonitrile |  |  | chloroform |  |
| --- | --- | --- | --- | --- | --- | --- | --- | --- | --- | --- | --- | --- | --- | --- | --- | --- | --- | --- |
| Element | x | y | z | x | y | z | x | y | z | x | y | z | x | y | z | x | y | z |
| C | 0.934580 | 3.002431 | -1.224243 | 0.936276 | 2.978271 | -1.261155 | 0.952462 | 2.977876 | -1.258825 | 0.957494 | 2.961977 | -1.368875 | 0.945276 | 2.967512 | -1.285178 | 0.951892 | 2.994120 | -1.253308 |
| C | 1.762638 | 4.115852 | -1.252052 | 1.765054 | 4.090142 | -1.314269 | 1.790182 | 4.082016 | -1.334449 | 1.795687 | 4.066030 | -1.435372 | 1.779180 | 4.074629 | -1.361167 | 1.787315 | 4.101229 | -1.305681 |
| C | 3.098046 | 4.018022 | -0.895954 | 3.100332 | 3.998666 | -0.952902 | 3.127036 | 3.984573 | -0.973884 | 3.123156 | 3.974061 | -1.039163 | 3.115108 | 3.983242 | -0.995067 | 3.122150 | 4.001188 | -0.941731 |
| C | 3.641623 | 2.798601 | -0.514470 | 3.643195 | 2.787547 | -0.544492 | 3.663010 | 2.775896 | -0.548399 | 3.651917 | 2.770573 | -0.589301 | 3.653952 | 2.777793 | -0.563588 | 3.658175 | 2.786596 | -0.533598 |
| C | 2.804640 | 1.702754 | -0.484433 | 2.805367 | 1.692937 | -0.492239 | 2.815987 | 1.689193 | -0.476335 | 2.803675 | 1.685403 | -0.524942 | 2.810676 | 1.688116 | -0.490750 | 2.813566 | 1.697009 | -0.482673 |
| C | 1.444136 | 1.773103 | -0.820279 | 1.444437 | 1.758677 | -0.827071 | 1.453700 | 1.763569 | -0.802063 | 1.449917 | 1.756962 | -0.880071 | 1.449443 | 1.756616 | -0.822069 | 1.453230 | 1.772436 | -0.816849 |
| N | 3.159871 | 0.378561 | -0.210230 | 3.157409 | 0.374633 | -0.188749 | 3.156114 | 0.372440 | -0.154509 | 3.135543 | 0.366499 | -0.190633 | 3.154615 | 0.372396 | -0.165974 | 3.156395 | 0.375051 | -0.182072 |
| C | 2.082940 | -0.468705 | -0.416736 | 2.078167 | -0.472291 | -0.363877 | 2.066282 | -0.461955 | -0.290888 | 2.053146 | -0.464308 | -0.354045 | 2.069872 | -0.466867 | -0.312634 | 2.071524 | -0.461890 | -0.354231 |
| C | 0.902668 | 0.406978 | -0.775989 | 0.901771 | 0.395530 | -0.753734 | 0.901088 | 0.408271 | -0.707967 | 0.897509 | 0.403804 | -0.789297 | 0.901558 | 0.399313 | -0.727474 | 0.903052 | 0.413260 | -0.747980 |
| C | -0.355584 | -0.075572 | -0.934244 | -0.355489 | -0.087943 | -0.912816 | -0.357074 | -0.064821 | -0.877238 | -0.361871 | -0.063254 | -0.957458 | -0.356326 | -0.076723 | -0.891071 | -0.355991 | -0.061009 | -0.913875 |
| O | 2.135605 | -1.661875 | -0.252112 | 2.118691 | -1.659125 | -0.148340 | 2.079171 | -1.638932 | -0.014465 | 2.056219 | -1.645329 | -0.075720 | 2.088920 | -1.646510 | -0.046908 | 2.096776 | -1.648600 | -0.128007 |
| C | -0.881476 | -1.439457 | -1.091968 | -0.873903 | -1.453210 | -1.075897 | -0.873226 | -1.424801 | -1.070849 | -0.882328 | -1.415551 | -1.172357 | -0.873004 | -1.437277 | -1.079631 | -0.878600 | -1.420462 | -1.101898 |
| C | -2.281749 | -1.343056 | -1.149417 | -2.273882 | -1.361977 | -1.140221 | -2.271653 | -1.330414 | -1.153685 | -2.280965 | -1.319170 | -1.223542 | -2.272014 | -1.343358 | -1.155520 | -2.277776 | -1.322331 | -1.167089 |
| N | -2.673675 | -0.015918 | -1.040034 | -2.672182 | -0.037612 | -1.020140 | -2.670006 | -0.007678 | -1.008269 | -2.672900 | 0.006062 | -1.044240 | -2.669922 | -0.019836 | -1.011471 | -2.670875 | 0.002433 | -1.023183 |
| C | -1.589584 | 0.811480 | -0.905649 | -1.593146 | 0.793128 | -0.883427 | -1.592937 | 0.815597 | -0.833996 | -1.591119 | 0.815921 | -0.866567 | -1.592076 | 0.802864 | -0.842323 | -1.589122 | 0.823217 | -0.863915 |
| C | -0.309048 | -2.693785 | -1.271586 | -0.294165 | -2.702268 | -1.267452 | -0.286206 | -2.665701 | -1.291821 | -0.297017 | -2.646414 | -1.445099 | -0.286877 | -2.678818 | -1.300307 | -0.301292 | -2.666361 | -1.320251 |
| C | -1.124754 | -3.801843 | -1.462626 | -1.103184 | -3.812610 | -1.473068 | -1.090535 | -3.771908 | -1.535791 | -1.106126 | -3.747278 | -1.695873 | -1.092539 | -3.785954 | -1.536750 | -1.113867 | -3.770043 | -1.548104 |
| C | -2.504348 | -3.677498 | -1.476870 | -2.484105 | -3.694262 | -1.491201 | -2.473144 | -3.653868 | -1.565130 | -2.489204 | -3.630909 | -1.684675 | -2.475378 | -3.668096 | -1.559245 | -2.495243 | -3.646713 | -1.562715 |
| C | -3.104550 | -2.434000 | -1.324408 | -3.091358 | -2.455149 | -1.329655 | -3.086076 | -2.420338 | -1.380071 | -3.099878 | -2.403021 | -1.456113 | -3.087488 | -2.433809 | -1.374626 | -3.099364 | -2.408931 | -1.377635 |
| O | -1.680007 | 2.006122 | -0.746329 | -1.684899 | 1.987575 | -0.713733 | -1.679087 | 2.003973 | -0.611913 | -1.664383 | 1.999464 | -0.597002 | -1.676764 | 1.991483 | -0.619252 | -1.671998 | 2.013891 | -0.656637 |
| C | 4.411448 | -0.057239 | 0.273136 | 4.415578 | -0.053410 | 0.288538 | 4.420368 | -0.061042 | 0.305469 | 4.383502 | -0.065573 | 0.316530 | 4.416475 | -0.056502 | 0.304962 | 4.411879 | -0.063855 | 0.294518 |
| C | 4.980280 | 0.568151 | 1.377255 | 4.977213 | 0.565126 | 1.399647 | 4.990996 | 0.544103 | 1.418551 | 4.916833 | 0.551205 | 1.441382 | 4.982883 | 0.561862 | 1.413231 | 4.976088 | 0.545249 | 1.409351 |
| C | 6.206934 | 0.150280 | 1.853980 | 6.210013 | 0.155880 | 1.867784 | 6.229039 | 0.128216 | 1.867349 | 6.135859 | 0.134129 | 1.938209 | 6.218500 | 0.150305 | 1.872856 | 6.205298 | 0.123569 | 1.876252 |
| C | 6.865988 | -0.910192 | 1.236677 | 6.878391 | -0.887708 | 1.231264 | 6.887586 | -0.905731 | 1.205017 | 6.810943 | -0.911811 | 1.312312 | 6.879095 | -0.892936 | 1.227012 | 6.864569 | -0.921980 | 1.233082 |
| C | 6.287128 | -1.545401 | 0.140584 | 6.308483 | -1.515708 | 0.125878 | 6.310984 | -1.518010 | 0.093765 | 6.271068 | -1.535060 | 0.189094 | 6.306162 | -1.519287 | 0.121548 | 6.292694 | -1.540138 | 0.122890 |
| C | 5.066312 | -1.116972 | -0.342257 | 5.081336 | -1.094319 | -0.346743 | 5.078464 | -1.089086 | -0.357990 | 5.057650 | -1.105102 | -0.311365 | 5.076083 | -1.094781 | -0.341344 | 5.069167 | -1.105922 | -0.347919 |
| C | 8.137735 | -1.347541 | 1.730393 | 8.156217 | -1.315539 | 1.715578 | 8.168930 | -1.341058 | 1.669231 | 8.071363 | -1.348623 | 1.828416 | 8.158181 | -1.323251 | 1.702639 | 8.137720 | -1.362850 | 1.716317 |
| N | 9.158628 | -1.695801 | 2.125417 | 9.182435 | -1.656293 | 2.103176 | 9.198370 | -1.690170 | 2.041937 | 9.084261 | -1.698849 | 2.243209 | 9.185792 | -1.668057 | 2.084494 | 9.160650 | -1.714732 | 2.103508 |
| C | -4.019882 | 0.450924 | -0.955255 | -4.025208 | 0.416597 | -0.956996 | -4.026963 | 0.441268 | -0.950864 | -4.026802 | 0.457393 | -0.933574 | -4.026224 | 0.431375 | -0.949953 | -4.024332 | 0.456577 | -0.945139 |
| C | -4.653623 | 0.161269 | 0.400437 | -4.661602 | 0.169290 | 0.405208 | -4.672708 | 0.166950 | 0.400139 | -4.625665 | 0.165016 | 0.431555 | -4.667743 | 0.160715 | 0.403348 | -4.656163 | 0.171477 | 0.410277 |
| O | -5.947483 | 0.454471 | 0.346061 | -5.958139 | 0.431200 | 0.331819 | -5.962814 | 0.443122 | 0.329833 | -5.907395 | 0.463551 | 0.424971 | -5.954187 | 0.453404 | 0.342309 | -5.952427 | 0.426697 | 0.349109 |
| O | -4.047788 | -0.254912 | 1.346576 | -4.050955 | -0.191319 | 1.373086 | -4.067938 | -0.225648 | 1.363363 | -3.988942 | -0.266652 | 1.361870 | -4.062829 | -0.244436 | 1.361653 | -4.039778 | -0.211801 | 1.368137 |
| C | -6.823432 | 0.320804 | 1.512134 | -6.846400 | 0.336204 | 1.496371 | -6.869476 | 0.308082 | 1.479490 | -6.780266 | 0.312549 | 1.604845 | -6.855597 | 0.328100 | 1.497621 | -6.841026 | 0.296087 | 1.512371 |
| C | -6.345902 | 1.238204 | 2.629612 | -6.399215 | 1.316601 | 2.570394 | -6.432093 | 1.239382 | 2.599138 | -6.289983 | 1.212510 | 2.727304 | -6.397250 | 1.251327 | 2.615882 | -6.398842 | 1.251140 | 2.610492 |
| C | -8.171326 | 0.780160 | 0.977874 | -8.196003 | 0.741973 | 0.926665 | -8.207555 | 0.748204 | 0.910236 | -8.125176 | 0.788187 | 1.082876 | -8.190455 | 0.788429 | 0.936247 | -8.191825 | 0.709668 | 0.951860 |
| C | -6.885081 | -1.136900 | 1.948319 | -6.884785 | -1.098910 | 2.000590 | -6.927821 | -1.145803 | 1.921091 | -6.846350 | -1.151586 | 2.007841 | -6.931052 | -1.126270 | 1.935971 | -6.871605 | -1.152144 | 1.976951 |
| H | -0.101503 | 3.093799 | -1.498164 | -0.099149 | 3.064078 | -1.540893 | -0.082312 | 3.066007 | -1.542989 | -0.066445 | 3.043456 | -1.694562 | -0.088557 | 3.050601 | -1.574653 | -0.082120 | 3.085544 | -1.538190 |
| H | 1.354779 | 5.070137 | -1.557595 | 1.359041 | 5.037909 | -1.642689 | 1.393037 | 5.027564 | -1.680622 | 1.407907 | 5.006603 | -1.804959 | 1.379987 | 5.017460 | -1.712300 | 1.388260 | 5.051530 | -1.635794 |
| H | 3.732042 | 4.895042 | -0.924754 | 3.734880 | 4.874840 | -0.998801 | 3.768383 | 4.854978 | -1.035311 | 3.764617 | 4.844817 | -1.095196 | 3.753586 | 4.855686 | -1.057225 | 3.761927 | 4.873734 | -0.986252 |
| H | 4.690526 | 2.709436 | -0.268717 | 4.691382 | 2.705132 | -0.292652 | 4.711227 | 2.690644 | -0.296775 | 4.695171 | 2.688803 | -0.316202 | 4.701590 | 2.697138 | -0.308390 | 4.705626 | 2.698829 | -0.280184 |
| H | 0.759494 | -2.810542 | -1.242548 | 0.775352 | -2.814558 | -1.238584 | 0.784398 | -2.775739 | -1.261077 | 0.774967 | -2.753476 | -1.458162 | 0.784007 | -2.787970 | -1.275294 | 0.768462 | -2.782365 | -1.297370 |
| H | -4.181289 | -2.330217 | -1.339610 | -4.168408 | -2.354220 | -1.352410 | -4.162932 | -2.319903 | -1.418716 | -4.177341 | -2.303566 | -1.467723 | -4.164405 | -2.332864 | -1.407682 | -4.176053 | -2.303217 | -1.399374 |
| H | -4.010213 | 1.532182 | -1.098340 | -4.034523 | 1.491541 | -1.141098 | -4.044232 | 1.517752 | -1.122560 | -4.053266 | 1.536106 | -1.090430 | -4.042600 | 1.507609 | -1.123322 | -4.036444 | 1.535967 | -1.100557 |
| H | -4.634898 | 0.015848 | -1.744950 | -4.626572 | -0.058860 | -1.733226 | -4.615996 | -0.029545 | -1.738612 | -4.646062 | -0.002170 | -1.704591 | -4.619820 | -0.040445 | -1.733516 | -4.627239 | 0.000842 | -1.731450 |
| H | -0.670648 | -4.775314 | -1.590805 | -0.644000 | -4.782837 | -1.610117 | -0.629069 | -4.737594 | -1.697676 | -0.648311 | -4.707320 | -1.897047 | -0.631858 | -4.752086 | -1.698092 | -0.658092 | -4.738912 | -1.705896 |
| H | -3.124851 | -4.554505 | -1.610743 | -3.099839 | -4.573005 | -1.636774 | -3.085819 | -4.529075 | -1.742663 | -3.105019 | -4.502759 | -1.867903 | -3.088901 | -4.543865 | -1.730897 | -3.113716 | -4.520583 | -1.725652 |
| H | 4.453729 | 1.376883 | 1.865960 | 4.442803 | 1.361183 | 1.900665 | 4.464109 | 1.336067 | 1.933812 | 4.376106 | 1.351884 | 1.928306 | 4.454682 | 1.360565 | 1.916498 | 4.448324 | 1.343468 | 1.913963 |
| H | 4.613619 | -1.606484 | -1.191798 | 4.636754 | -1.572192 | -1.207776 | 4.629437 | -1.546093 | -1.228687 | 4.635625 | -1.570809 | -1.191299 | 4.629086 | -1.563825 | -1.206493 | 4.622445 | -1.573278 | -1.213928 |
| H | 6.652355 | 0.634032 | 2.712588 | 6.651076 | 0.633872 | 2.731969 | 6.680480 | 0.594766 | 2.732398 | 6.559632 | 0.608468 | 2.813131 | 6.666439 | 0.627348 | 2.733989 | 6.649626 | 0.593518 | 2.743219 |
| H | 6.800458 | -2.370404 | -0.334225 | 6.831143 | -2.326019 | -0.364110 | 6.830923 | -2.317172 | -0.417056 | 6.804317 | -2.343774 | -0.292445 | 6.827090 | -2.326078 | -0.376117 | 6.809941 | -2.351074 | -0.371911 |
| H | -6.246714 | 2.261477 | 2.264058 | -6.304477 | 2.321472 | 2.154738 | -6.318943 | 2.259058 | 2.225763 | -6.165490 | 2.235788 | 2.368161 | -6.274866 | 2.270085 | 2.243014 | -6.307521 | 2.266077 | 2.219088 |
| H | -7.083850 | 1.234525 | 3.433312 | -7.150761 | 1.346624 | 3.361149 | -7.201386 | 1.244406 | 3.373222 | -7.037376 | 1.218859 | 3.522538 | -7.159876 | 1.263758 | 3.396401 | -7.152750 | 1.257861 | 3.399542 |
| H | -5.390462 | 0.911117 | 3.033014 | -5.447830 | 1.025234 | 3.010295 | -5.493818 | 0.919042 | 3.047606 | -5.347476 | 0.866414 | 3.147450 | -5.459048 | 0.917929 | 3.055020 | -5.447428 | 0.952357 | 3.045813 |
| H | -8.491823 | 0.148488 | 0.148629 | -8.496851 | 0.065613 | 0.125069 | -8.503270 | 0.107286 | 0.077919 | -8.454431 | 0.170146 | 0.245728 | -8.498824 | 0.152830 | 0.104459 | -8.488181 | 0.051723 | 0.133230 |
| H | -8.114572 | 1.811372 | 0.627861 | -8.158483 | 1.757301 | 0.529151 | -8.157763 | 1.780040 | 0.558343 | -8.065086 | 1.826406 | 0.751740 | -8.126809 | 1.819995 | 0.585836 | -8.158259 | 1.735402 | 0.581187 |
| H | -8.920987 | 0.722401 | 1.767726 | -8.953792 | 0.706257 | 1.710355 | -8.972703 | 0.683810 | 1.684840 | -8.869669 | 0.720577 | 1.876959 | -8.952199 | 0.733599 | 1.714903 | -8.949037 | 0.649611 | 1.734597 |
| H | -5.930065 | -1.475244 | 2.343446 | -5.931837 | -1.399438 | 2.430783 | -5.986235 | -1.473051 | 2.357584 | -5.902603 | -1.505388 | 2.418835 | -5.991203 | -1.467044 | 2.365924 | -5.918663 | -1.458653 | 2.403480 |
| H | -7.169610 | -1.772484 | 1.107975 | -7.139439 | -1.782469 | 1.188314 | -7.172550 | -1.792126 | 1.075913 | -7.115779 | -1.770812 | 1.150104 | -7.189387 | -1.766222 | 1.090045 | -7.118935 | -1.814418 | 1.144945 |
| H | -7.642449 | -1.245008 | 2.726261 | -7.653996 | -1.186789 | 2.769771 | -7.712844 | -1.255349 | 2.671301 | -7.618130 | -1.270851 | 2.770217 | -7.713153 | -1.226878 | 2.690425 | -7.643582 | -1.263881 | 2.740278 |

| **iso-I** 21 |  | gasphase |  |  | toluene |  |  | DMSO |  |  | methanol |  |  | acetonitrile |  |  | chloroform |  |
| --- | --- | --- | --- | --- | --- | --- | --- | --- | --- | --- | --- | --- | --- | --- | --- | --- | --- | --- |
| Element | x | y | z | x | y | z | x | y | z | x | y | z | x | y | z | x | y | z |
| C | 0.045969 | -2.646037 | -0.778064 | 0.047079 | -2.647337 | -0.752050 | 0.076750 | -2.656445 | -0.843611 | 0.103932 | -2.634960 | -1.075340 | 0.078655 | -2.658275 | -0.866734 | 0.060706 | -2.649391 | -0.829872 |
| C | 0.688909 | -3.851930 | -1.014707 | 0.689010 | -3.853006 | -0.992486 | 0.729572 | -3.855411 | -1.090286 | 0.772495 | -3.823158 | -1.331730 | 0.733094 | -3.857675 | -1.108088 | 0.709165 | -3.851947 | -1.070178 |
| C | 2.070334 | -3.946551 | -0.944419 | 2.070940 | -3.948298 | -0.920289 | 2.111870 | -3.943893 | -0.990631 | 2.149351 | -3.911031 | -1.172708 | 2.114834 | -3.946078 | -1.000157 | 2.090435 | -3.945279 | -0.972986 |
| C | 2.842806 | -2.831373 | -0.651140 | 2.844789 | -2.834453 | -0.624659 | 2.875951 | -2.830888 | -0.665020 | 2.895339 | -2.807450 | -0.777690 | 2.876860 | -2.832316 | -0.671544 | 2.857887 | -2.832745 | -0.654302 |
| C | 2.188278 | -1.640284 | -0.412613 | 2.190769 | -1.643192 | -0.384986 | 2.210703 | -1.646765 | -0.420948 | 2.214269 | -1.636117 | -0.522569 | 2.210089 | -1.647927 | -0.433038 | 2.197286 | -1.645043 | -0.415923 |
| C | 0.791676 | -1.518001 | -0.452683 | 0.793972 | -1.521495 | -0.421412 | 0.813977 | -1.535141 | -0.477549 | 0.821334 | -1.527744 | -0.635252 | 0.813563 | -1.536097 | -0.497843 | 0.800924 | -1.526863 | -0.473357 |
| N | 2.769981 | -0.388690 | -0.187908 | 2.771381 | -0.392017 | -0.155982 | 2.777049 | -0.394518 | -0.164826 | 2.761440 | -0.387179 | -0.202870 | 2.775371 | -0.395120 | -0.175636 | 2.770202 | -0.393586 | -0.167391 |
| C | 1.799532 | 0.599706 | -0.142564 | 1.802377 | 0.592185 | -0.091757 | 1.801116 | 0.577345 | -0.086963 | 1.780516 | 0.572972 | -0.143500 | 1.799332 | 0.576732 | -0.106665 | 1.798357 | 0.584794 | -0.104908 |
| C | 0.464676 | -0.097263 | -0.271932 | 0.467222 | -0.102685 | -0.233132 | 0.474652 | -0.125062 | -0.274369 | 0.468609 | -0.128059 | -0.403771 | 0.473385 | -0.125434 | -0.297091 | 0.468097 | -0.112207 | -0.277287 |
| C | -0.726359 | 0.546325 | -0.175571 | -0.724316 | 0.539652 | -0.141616 | -0.722145 | 0.504850 | -0.192907 | -0.731475 | 0.495783 | -0.331070 | -0.723113 | 0.505725 | -0.216157 | -0.725346 | 0.525830 | -0.191538 |
| O | 2.043225 | 1.764906 | 0.050204 | 2.041438 | 1.754630 | 0.129555 | 2.017925 | 1.733748 | 0.191271 | 1.974703 | 1.725819 | 0.180064 | 2.015271 | 1.734833 | 0.166166 | 2.025582 | 1.745532 | 0.143372 |
| C | -1.104551 | 1.956064 | -0.273918 | -1.100994 | 1.949119 | -0.254728 | -1.095935 | 1.912546 | -0.315623 | -1.100625 | 1.903305 | -0.434245 | -1.096341 | 1.914569 | -0.331528 | -1.098830 | 1.935340 | -0.301675 |
| C | -2.495634 | 2.048013 | -0.107490 | -2.492486 | 2.042205 | -0.095332 | -2.481424 | 2.012292 | -0.124066 | -2.478061 | 2.010647 | -0.204210 | -2.481141 | 2.014443 | -0.134586 | -2.486371 | 2.033714 | -0.118773 |
| N | -3.038941 | 0.766587 | 0.108470 | -3.034708 | 0.763950 | 0.132398 | -3.019941 | 0.738807 | 0.131629 | -3.015335 | 0.732544 | 0.059280 | -3.020029 | 0.739631 | 0.116443 | -3.028471 | 0.756365 | 0.118045 |
| C | -2.012640 | -0.189126 | 0.132118 | -2.011546 | -0.190409 | 0.169714 | -2.004842 | -0.222787 | 0.133487 | -2.007827 | -0.234337 | -0.010573 | -2.005936 | -0.222794 | 0.106361 | -2.010132 | -0.203189 | 0.123942 |
| C | -0.397598 | 3.113257 | -0.591079 | -0.394719 | 3.101477 | -0.589557 | -0.394711 | 3.049793 | -0.706133 | -0.401104 | 3.030430 | -0.854536 | -0.395845 | 3.053808 | -0.718390 | -0.393908 | 3.080610 | -0.662145 |
| C | -1.071342 | 4.317726 | -0.705705 | -1.068532 | 4.304006 | -0.725251 | -1.067132 | 4.252584 | -0.851719 | -1.067730 | 4.238040 | -0.980697 | -1.068260 | 4.257818 | -0.855294 | -1.064793 | 4.285734 | -0.792173 |
| C | -2.442439 | 4.381824 | -0.511978 | -2.440909 | 4.369991 | -0.537634 | -2.434601 | 4.329102 | -0.625324 | -2.427003 | 4.324767 | -0.711687 | -2.435040 | 4.333912 | -0.624039 | -2.433312 | 4.359029 | -0.576266 |
| C | -3.177840 | 3.243140 | -0.214410 | -3.175562 | 3.234629 | -0.225719 | -3.164737 | 3.203729 | -0.267687 | -3.156952 | 3.205804 | -0.332578 | -3.164569 | 3.206842 | -0.269857 | -3.167076 | 3.228317 | -0.244229 |
| O | -2.143886 | -1.334442 | 0.460597 | -2.146172 | -1.331087 | 0.517252 | -2.139968 | -1.367189 | 0.474903 | -2.141517 | -1.397621 | 0.273369 | -2.141593 | -1.371245 | 0.434256 | -2.144840 | -1.350295 | 0.453660 |
| C | 4.140820 | -0.144906 | 0.040349 | 4.147021 | -0.145749 | 0.050161 | 4.148970 | -0.142164 | 0.064140 | 4.121099 | -0.135205 | 0.096443 | 4.145770 | -0.142318 | 0.064112 | 4.142856 | -0.144680 | 0.060680 |
| C | 4.797453 | 0.856806 | -0.663786 | 4.798869 | 0.819954 | -0.706369 | 4.807122 | 0.817950 | -0.693991 | 4.806223 | 0.849971 | -0.602770 | 4.811004 | 0.814996 | -0.691557 | 4.804842 | 0.815671 | -0.693851 |
| C | 6.137163 | 1.097099 | -0.430259 | 6.141774 | 1.065151 | -0.498218 | 6.144109 | 1.075466 | -0.463051 | 6.130207 | 1.105485 | -0.304023 | 6.146145 | 1.072983 | -0.449548 | 6.143493 | 1.066105 | -0.464285 |
| C | 6.831571 | 0.330421 | 0.502517 | 6.838953 | 0.335317 | 0.462172 | 6.822610 | 0.359715 | 0.521571 | 6.766898 | 0.362418 | 0.687925 | 6.815782 | 0.360396 | 0.543530 | 6.822923 | 0.344755 | 0.515290 |
| C | 6.170900 | -0.671392 | 1.209467 | 6.184247 | -0.632965 | 1.220257 | 6.163313 | -0.607534 | 1.277500 | 6.079605 | -0.629138 | 1.385023 | 6.149302 | -0.603918 | 1.297189 | 6.158726 | -0.620062 | 1.269950 |
| C | 4.828201 | -0.900901 | 0.983528 | 4.838349 | -0.865607 | 1.017758 | 4.823141 | -0.851564 | 1.050745 | 4.752221 | -0.871645 | 1.091119 | 4.810948 | -0.848255 | 1.059422 | 4.816963 | -0.857234 | 1.045441 |
| C | 8.224065 | 0.572538 | 0.736529 | 8.234281 | 0.580570 | 0.671040 | 8.209501 | 0.619965 | 0.758152 | 8.140424 | 0.619784 | 0.993281 | 8.200910 | 0.620972 | 0.791356 | 8.212929 | 0.595840 | 0.747734 |
| N | 9.341130 | 0.764240 | 0.923190 | 9.354090 | 0.774899 | 0.837312 | 9.323352 | 0.828969 | 0.948281 | 9.244058 | 0.826150 | 1.238222 | 9.313244 | 0.830078 | 0.990411 | 9.329014 | 0.795646 | 0.933330 |
| C | -4.400771 | 0.519143 | 0.363545 | -4.397101 | 0.517561 | 0.392776 | -4.371982 | 0.508387 | 0.461762 | -4.341994 | 0.510707 | 0.470868 | -4.368186 | 0.509616 | 0.461300 | -4.384041 | 0.517545 | 0.417721 |
| O | -4.709943 | -0.712389 | 0.027774 | -4.722241 | -0.690150 | -0.001529 | -4.750712 | -0.668410 | 0.031953 | -4.731450 | -0.695715 | 0.155727 | -4.751349 | -0.668861 | 0.040806 | -4.730255 | -0.685609 | 0.032853 |
| O | -5.135225 | 1.365944 | 0.795892 | -5.114087 | 1.352359 | 0.876216 | -5.032881 | 1.337402 | 1.030347 | -4.985085 | 1.373225 | 1.015504 | -5.023833 | 1.339904 | 1.034438 | -5.079181 | 1.357490 | 0.925890 |
| C | -6.005997 | -1.303728 | 0.367417 | -6.026887 | -1.288424 | 0.311893 | -6.067669 | -1.239128 | 0.368740 | -6.030829 | -1.252941 | 0.590495 | -6.062252 | -1.241909 | 0.396741 | -6.035325 | -1.273595 | 0.375332 |
| C | -6.214447 | -1.257453 | 1.874445 | -6.228940 | -1.321797 | 1.818820 | -6.216471 | -1.336896 | 1.877962 | -6.111320 | -1.241910 | 2.107406 | -6.188569 | -1.336805 | 1.908404 | -6.202035 | -1.308664 | 1.886117 |
| C | -5.844371 | -2.738404 | -0.110356 | -5.883331 | -2.695816 | -0.243477 | -5.998244 | -2.621814 | -0.255451 | -5.960037 | -2.675337 | 0.063347 | -5.995719 | -2.625567 | -0.226351 | -5.916252 | -2.680282 | -0.186801 |
| C | -7.117166 | -0.596922 | -0.395615 | -7.130963 | -0.530978 | -0.409407 | -7.166613 | -0.410669 | -0.275774 | -7.162019 | -0.484624 | -0.070839 | -7.171877 | -0.417240 | -0.234769 | -7.146701 | -0.503940 | -0.320476 |
| H | -1.027340 | -2.594102 | -0.833124 | -1.026343 | -2.594230 | -0.811478 | -0.995529 | -2.603533 | -0.931687 | -0.962678 | -2.577580 | -1.220440 | -0.993076 | -2.605451 | -0.960834 | -1.011519 | -2.595420 | -0.913281 |
| H | 0.100503 | -4.726949 | -1.256289 | 0.100463 | -4.727086 | -1.238377 | 0.152081 | -4.728795 | -1.364502 | 0.212226 | -4.688101 | -1.662676 | 0.157133 | -4.731386 | -1.384428 | 0.127260 | -4.724705 | -1.336396 |
| H | 2.557846 | -4.894725 | -1.131823 | 2.558093 | -4.896766 | -1.108501 | 2.607588 | -4.887261 | -1.183072 | 2.656650 | -4.846210 | -1.375059 | 2.611753 | -4.889673 | -1.188287 | 2.582578 | -4.891372 | -1.160802 |
| H | 3.921968 | -2.891044 | -0.631126 | 3.923944 | -2.897392 | -0.602019 | 3.954788 | -2.891097 | -0.620649 | 3.971692 | -2.865697 | -0.689787 | 3.955384 | -2.892054 | -0.620381 | 3.936612 | -2.894948 | -0.612354 |
| H | 0.668645 | 3.072634 | -0.725615 | 0.671745 | 3.060016 | -0.725196 | 0.665635 | 2.998615 | -0.886034 | 0.649935 | 2.967035 | -1.083504 | 0.663808 | 3.002995 | -0.902359 | 0.668817 | 3.033166 | -0.825907 |
| H | -4.243627 | 3.287536 | -0.061233 | -4.243181 | 3.282066 | -0.083085 | -4.230508 | 3.261023 | -0.111100 | -4.218909 | 3.273433 | -0.156276 | -4.230042 | 3.263055 | -0.110488 | -4.232633 | 3.282577 | -0.087757 |
| H | -0.515846 | 5.215714 | -0.941415 | -0.513121 | 5.198720 | -0.974445 | -0.517893 | 5.137915 | -1.145195 | -0.522537 | 5.118002 | -1.297071 | -0.519457 | 5.144333 | -1.145969 | -0.511412 | 5.176119 | -1.061163 |
| H | -2.955617 | 5.331541 | -0.594301 | -2.955032 | 5.317597 | -0.638216 | -2.948445 | 5.275576 | -0.738564 | -2.937190 | 5.274537 | -0.813478 | -2.948819 | 5.281152 | -0.730874 | -2.945447 | 5.308132 | -0.673653 |
| H | 4.254776 | 1.448857 | -1.385864 | 4.253546 | 1.377131 | -1.454607 | 4.274760 | 1.356412 | -1.465501 | 4.305192 | 1.409405 | -1.380903 | 4.285435 | 1.351345 | -1.469154 | 4.272346 | 1.362690 | -1.459030 |
| H | 4.305013 | -1.662926 | 1.545400 | 4.318700 | -1.601692 | 1.616357 | 4.297600 | -1.588714 | 1.643019 | 4.203685 | -1.628267 | 1.636397 | 4.279780 | -1.583134 | 1.649486 | 4.288582 | -1.591052 | 1.639309 |
| H | 6.652790 | 1.876244 | -0.974865 | 6.654744 | 1.816569 | -1.083108 | 6.664752 | 1.821443 | -1.048261 | 6.673395 | 1.870887 | -0.841925 | 6.672185 | 1.816909 | -1.032519 | 6.665703 | 1.813197 | -1.046668 |
| H | 6.708873 | -1.257098 | 1.942369 | 6.726811 | -1.189386 | 1.972540 | 6.695515 | -1.154530 | 2.043977 | 6.580119 | -1.196751 | 2.157991 | 6.674488 | -1.148238 | 2.070370 | 6.689518 | -1.169952 | 2.035391 |
| H | -7.109945 | -1.827205 | 2.126375 | -7.120355 | -1.909433 | 2.043963 | -7.119132 | -1.906792 | 2.103616 | -6.992290 | -1.809091 | 2.411994 | -7.087413 | -1.906793 | 2.148456 | -7.093868 | -1.888712 | 2.128620 |
| H | -5.364709 | -1.711357 | 2.386326 | -5.375875 | -1.797002 | 2.306092 | -5.363971 | -1.862069 | 2.312588 | -5.230266 | -1.720527 | 2.538697 | -5.328835 | -1.860535 | 2.330306 | -5.342510 | -1.793612 | 2.352228 |
| H | -6.343118 | -0.237288 | 2.229800 | -6.364574 | -0.324242 | 2.231934 | -6.306041 | -0.356383 | 2.341785 | -6.200866 | -0.233231 | 2.506739 | -6.271602 | -0.355104 | 2.371041 | -6.319114 | -0.311008 | 2.305059 |
| H | -6.754573 | -3.301428 | 0.098830 | -6.804709 | -3.254078 | -0.073119 | -6.937725 | -3.146735 | -0.078783 | -6.878421 | -3.204149 | 0.320341 | -6.929598 | -3.155011 | -0.034305 | -6.839153 | -3.229558 | 0.003106 |
| H | -5.657526 | -2.765431 | -1.184339 | -5.688963 | -2.668983 | -1.316644 | -5.838173 | -2.551976 | -1.332546 | -5.849714 | -2.678991 | -1.022316 | -5.852456 | -2.555791 | -1.305821 | -5.745836 | -2.651854 | -1.264143 |
| H | -5.007721 | -3.216004 | 0.399477 | -5.061692 | -3.219978 | 0.245640 | -5.186931 | -3.203777 | 0.183993 | -5.116281 | -3.207829 | 0.505155 | -5.174258 | -3.201805 | 0.201735 | -5.089480 | -3.213540 | 0.284016 |
| H | -7.244849 | 0.427186 | -0.052677 | -7.256953 | 0.472653 | -0.009088 | -7.241354 | 0.577913 | 0.172883 | -7.249576 | 0.528326 | 0.317596 | -7.244266 | 0.571184 | 0.214728 | -7.258677 | 0.498618 | 0.087179 |
| H | -6.898450 | -0.590803 | -1.464513 | -6.913524 | -0.463410 | -1.476911 | -6.987169 | -0.304167 | -1.347251 | -7.012464 | -0.441449 | -1.151155 | -7.004380 | -0.310774 | -1.308145 | -6.949075 | -0.433301 | -1.391683 |
| H | -8.052827 | -1.137208 | -0.243151 | -8.070766 | -1.072556 | -0.288857 | -8.119078 | -0.925654 | -0.139837 | -8.099339 | -1.008759 | 0.123248 | -8.120778 | -0.935638 | -0.087492 | -8.087166 | -1.040356 | -0.183259 |

| **iso-I** 22 |  | gasphase |  |  | toluene |  |  | DMSO |  |  | methanol |  |  | acetonitrile |  |  | chloroform |  |
| --- | --- | --- | --- | --- | --- | --- | --- | --- | --- | --- | --- | --- | --- | --- | --- | --- | --- | --- |
| Element | x | y | z | x | y | z | x | y | z | x | y | z | x | y | z | x | y | z |
| C | -1.026119 | 2.811260 | -1.010994 | -1.020705 | 2.804275 | -0.978133 | -1.023909 | 2.785407 | -1.009731 | -1.038699 | 2.760847 | -1.188654 | -1.021491 | 2.786420 | -1.016812 | -1.025133 | 2.785515 | -1.080197 |
| C | -1.737623 | 3.994746 | -1.147437 | -1.731371 | 3.985021 | -1.139757 | -1.735574 | 3.962715 | -1.192756 | -1.753355 | 3.938165 | -1.358996 | -1.731970 | 3.964907 | -1.197986 | -1.735313 | 3.967467 | -1.236780 |
| C | -3.105468 | 4.030755 | -0.928784 | -3.102167 | 4.022416 | -0.935108 | -3.106781 | 4.003208 | -0.979879 | -3.113836 | 3.988005 | -1.086277 | -3.102835 | 4.007065 | -0.982480 | -3.101474 | 4.011340 | -0.999223 |
| C | -3.798461 | 2.879298 | -0.579149 | -3.798325 | 2.875249 | -0.577554 | -3.802471 | 2.863280 | -0.597795 | -3.798969 | 2.857084 | -0.659003 | -3.799368 | 2.867700 | -0.599552 | -3.794197 | 2.869862 | -0.616270 |
| C | -3.076901 | 1.712486 | -0.439158 | -3.077016 | 1.711051 | -0.414265 | -3.079354 | 1.702489 | -0.415738 | -3.072327 | 1.697777 | -0.489702 | -3.077445 | 1.705882 | -0.419065 | -3.072928 | 1.704652 | -0.459188 |
| C | -1.688837 | 1.647413 | -0.635406 | -1.686566 | 1.645747 | -0.591903 | -1.689371 | 1.637485 | -0.592703 | -1.691620 | 1.626254 | -0.719831 | -1.687820 | 1.639218 | -0.598745 | -1.686959 | 1.634900 | -0.664685 |
| N | -3.581787 | 0.436018 | -0.172061 | -3.581776 | 0.438067 | -0.132989 | -3.579578 | 0.432924 | -0.113179 | -3.562068 | 0.429183 | -0.154645 | -3.578735 | 0.436079 | -0.117034 | -3.574018 | 0.433421 | -0.160978 |
| C | -2.575324 | -0.512050 | -0.247201 | -2.573272 | -0.506116 | -0.168329 | -2.569372 | -0.506324 | -0.123692 | -2.557865 | -0.507707 | -0.199429 | -2.569835 | -0.504018 | -0.132772 | -2.569368 | -0.511955 | -0.213982 |
| C | -1.288073 | 0.240253 | -0.511122 | -1.286346 | 0.241350 | -0.445722 | -1.287225 | 0.238565 | -0.424866 | -1.287562 | 0.230720 | -0.547551 | -1.287125 | 0.239496 | -0.432843 | -1.287028 | 0.232266 | -0.514851 |
| C | -0.074061 | -0.365382 | -0.533785 | -0.072491 | -0.363482 | -0.470942 | -0.071523 | -0.358580 | -0.450449 | -0.071638 | -0.363548 | -0.578371 | -0.072208 | -0.359533 | -0.457389 | -0.072551 | -0.369502 | -0.538935 |
| O | -2.749831 | -1.688614 | -0.050845 | -2.741108 | -1.676633 | 0.073461 | -2.724467 | -1.669660 | 0.166075 | -2.701101 | -1.673125 | 0.105766 | -2.726166 | -1.668787 | 0.151682 | -2.731640 | -1.682591 | 0.037270 |
| C | 0.321209 | -1.775622 | -0.631683 | 0.319425 | -1.772355 | -0.588507 | 0.320696 | -1.763355 | -0.588641 | 0.319931 | -1.766993 | -0.709443 | 0.318800 | -1.765011 | -0.594365 | 0.319365 | -1.777584 | -0.652334 |
| C | 1.718466 | -1.832894 | -0.510465 | 1.718131 | -1.831865 | -0.488420 | 1.719117 | -1.822316 | -0.493499 | 1.713826 | -1.829565 | -0.576114 | 1.717005 | -1.825472 | -0.495475 | 1.715927 | -1.838424 | -0.530768 |
| N | 2.234928 | -0.549240 | -0.316312 | 2.237306 | -0.550797 | -0.285112 | 2.235652 | -0.543362 | -0.273261 | 2.225316 | -0.547372 | -0.344680 | 2.234313 | -0.546560 | -0.274387 | 2.232241 | -0.556651 | -0.322927 |
| C | 1.226843 | 0.395325 | -0.365655 | 1.229691 | 0.392121 | -0.297325 | 1.227500 | 0.395408 | -0.255569 | 1.221519 | 0.388181 | -0.363625 | 1.227226 | 0.392740 | -0.260381 | 1.226736 | 0.385635 | -0.349341 |
| C | -0.358510 | -2.952804 | -0.927173 | -0.366413 | -2.943784 | -0.892374 | -0.366965 | -2.926500 | -0.918400 | -0.363492 | -2.922728 | -1.070799 | -0.369311 | -2.927689 | -0.925668 | -0.364185 | -2.945842 | -0.972971 |
| C | 0.347214 | -4.140309 | -1.058467 | 0.334882 | -4.130744 | -1.048636 | 0.334032 | -4.111238 | -1.095081 | 0.338544 | -4.109302 | -1.229639 | 0.331042 | -4.113380 | -1.099946 | 0.337643 | -4.134311 | -1.117880 |
| C | 1.725077 | -4.167725 | -0.912497 | 1.715026 | -4.161931 | -0.918346 | 1.715399 | -4.144814 | -0.960672 | 1.714881 | -4.148573 | -1.050531 | 1.712103 | -4.148336 | -0.961808 | 1.715678 | -4.168230 | -0.960264 |
| C | 2.433888 | -3.004109 | -0.644294 | 2.429640 | -3.003115 | -0.644505 | 2.431326 | -2.991056 | -0.667509 | 2.427590 | -2.998546 | -0.733213 | 2.428490 | -2.994984 | -0.667226 | 2.428381 | -3.010810 | -0.673644 |
| O | 1.408468 | 1.578150 | -0.219269 | 1.408540 | 1.571464 | -0.112355 | 1.397959 | 1.569106 | -0.020085 | 1.385615 | 1.568252 | -0.131806 | 1.398376 | 1.567002 | -0.026567 | 1.401534 | 1.565835 | -0.156623 |
| C | -4.910697 | 0.134122 | 0.195214 | -4.920737 | 0.136795 | 0.202100 | -4.921401 | 0.134070 | 0.215189 | -4.888155 | 0.136450 | 0.242208 | -4.919666 | 0.136666 | 0.215411 | -4.904297 | 0.138281 | 0.213987 |
| C | -5.600143 | -0.880718 | -0.456499 | -5.604448 | -0.847399 | -0.500326 | -5.601072 | -0.845564 | -0.497774 | -5.601914 | -0.850446 | -0.425387 | -5.599543 | -0.846380 | -0.492957 | -5.609347 | -0.850016 | -0.461376 |
| C | -6.897271 | -1.178238 | -0.087545 | -6.910644 | -1.147382 | -0.167739 | -6.909831 | -1.144743 | -0.174574 | -6.892168 | -1.146547 | -0.031832 | -6.907139 | -1.146839 | -0.165616 | -6.905857 | -1.145032 | -0.088198 |
| C | -7.516483 | -0.455648 | 0.929490 | -7.539753 | -0.453155 | 0.863420 | -7.539562 | -0.451113 | 0.857169 | -7.467641 | -0.442051 | 1.023607 | -7.535639 | -0.451265 | 0.865750 | -7.501232 | -0.440641 | 0.956214 |
| C | -6.822723 | 0.559723 | 1.583369 | -6.853365 | 0.534339 | 1.566612 | -6.858797 | 0.534430 | 1.568983 | -6.752596 | 0.552118 | 1.688424 | -6.854475 | 0.537318 | 1.573236 | -6.794090 | 0.551995 | 1.631618 |
| C | -5.521358 | 0.846360 | 1.221402 | -5.542966 | 0.821674 | 1.239358 | -5.545928 | 0.820297 | 1.249666 | -5.458129 | 0.835001 | 1.299246 | -5.542710 | 0.824454 | 1.249914 | -5.493463 | 0.834069 | 1.263320 |
| C | -8.865950 | -0.756945 | 1.304609 | -8.897834 | -0.755427 | 1.202253 | -8.898530 | -0.752870 | 1.187204 | -8.806194 | -0.742654 | 1.428623 | -8.893578 | -0.754205 | 1.199977 | -8.848401 | -0.738196 | 1.337930 |
| N | -9.948621 | -0.996148 | 1.604609 | -9.987964 | -0.995604 | 1.472945 | -9.990317 | -0.994876 | 1.451657 | -9.881626 | -0.983916 | 1.754017 | -9.984462 | -0.997019 | 1.467711 | -9.930274 | -0.975309 | 1.643461 |
| C | 3.586811 | -0.237844 | -0.043570 | 3.595664 | -0.242083 | -0.034879 | 3.596159 | -0.238299 | -0.026405 | 3.577409 | -0.241869 | -0.049668 | 3.594662 | -0.240984 | -0.025401 | 3.586617 | -0.247858 | -0.049076 |
| C | 4.246678 | -0.886560 | 0.994053 | 4.262715 | -0.870566 | 1.010183 | 4.258936 | -0.857206 | 1.026461 | 4.198674 | -0.856926 | 1.030188 | 4.255752 | -0.857430 | 1.030014 | 4.226566 | -0.857906 | 1.023367 |
| C | 5.568482 | -0.589753 | 1.261586 | 5.588898 | -0.573690 | 1.255410 | 5.586861 | -0.562176 | 1.267129 | 5.515289 | -0.558243 | 1.320876 | 5.583258 | -0.561132 | 1.272518 | 5.546740 | -0.556945 | 1.294411 |
| C | 6.230511 | 0.369068 | 0.506303 | 6.245308 | 0.363951 | 0.468248 | 6.245289 | 0.362467 | 0.465584 | 6.203328 | 0.364754 | 0.542149 | 6.242715 | 0.362378 | 0.470341 | 6.222565 | 0.365118 | 0.504351 |
| C | 5.568342 | 1.025065 | -0.518331 | 5.577349 | 0.997231 | -0.567242 | 5.582243 | 0.982936 | -0.581583 | 5.580930 | 0.980773 | -0.531737 | 5.581112 | 0.980609 | -0.579285 | 5.582012 | 0.977908 | -0.560524 |
| C | 4.247319 | 0.719618 | -0.797697 | 4.251715 | 0.690506 | -0.823142 | 4.254974 | 0.677444 | -0.832072 | 4.264525 | 0.671878 | -0.832840 | 4.254353 | 0.673871 | -0.831593 | 4.262041 | 0.666514 | -0.841962 |
| C | 7.679430 | 0.653038 | 0.798398 | 7.694908 | 0.652328 | 0.744215 | 7.694214 | 0.649550 | 0.737464 | 7.635941 | 0.663069 | 0.882613 | 7.691607 | 0.650474 | 0.743329 | 7.660311 | 0.666358 | 0.822701 |
| F | 8.113914 | 1.763374 | 0.193532 | 8.143310 | 1.724180 | 0.081615 | 8.152079 | 1.708068 | 0.058506 | 8.150601 | 1.657473 | 0.150223 | 8.147156 | 1.713741 | 0.070130 | 8.158248 | 1.664065 | 0.083794 |
| F | 8.468048 | -0.354144 | 0.386946 | 8.481245 | -0.381278 | 0.391092 | 8.481240 | -0.392308 | 0.404585 | 8.428505 | -0.409043 | 0.691779 | 8.480258 | -0.388082 | 0.403702 | 8.450961 | -0.403413 | 0.615100 |
| F | 7.900333 | 0.802017 | 2.112433 | 7.921802 | 0.872337 | 2.048865 | 7.925031 | 0.892780 | 2.039126 | 7.783081 | 1.013599 | 2.172635 | 7.922729 | 0.886437 | 2.046164 | 7.826181 | 1.012536 | 2.110628 |
| H | 0.036165 | 2.798555 | -1.179016 | 0.043448 | 2.790383 | -1.136928 | 0.038769 | 2.766563 | -1.182658 | 0.013475 | 2.731073 | -1.419754 | 0.040795 | 2.765700 | -1.192237 | 0.034302 | 2.764971 | -1.269949 |
| H | -1.212866 | 4.897767 | -1.429052 | -1.204485 | 4.884802 | -1.428950 | -1.212253 | 4.856596 | -1.506889 | -1.242227 | 4.824241 | -1.712940 | -1.208090 | 4.858185 | -1.512853 | -1.213247 | 4.862584 | -1.548894 |
| H | -3.647123 | 4.961198 | -1.041625 | -3.643123 | 4.951227 | -1.065369 | -3.648686 | 4.929407 | -1.125418 | -3.657473 | 4.914537 | -1.223466 | -3.643860 | 4.933971 | -1.126725 | -3.642076 | 4.941061 | -1.125254 |
| H | -4.870346 | 2.895261 | -0.440562 | -4.871479 | 2.893683 | -0.447757 | -4.875019 | 2.885995 | -0.462108 | -4.865461 | 2.886077 | -0.482120 | -4.871602 | 2.891563 | -0.461969 | -4.864153 | 2.894163 | -0.462410 |
| H | -1.428318 | -2.947061 | -1.037358 | -1.437762 | -2.934894 | -0.991853 | -1.438101 | -2.913664 | -1.028124 | -1.429379 | -2.902520 | -1.228568 | -1.440141 | -2.913554 | -1.038450 | -1.433314 | -2.933584 | -1.097975 |
| H | 3.511637 | -3.013922 | -0.561025 | 3.508292 | -3.018561 | -0.572850 | 3.509999 | -3.009927 | -0.593107 | 3.503839 | -3.021489 | -0.629364 | 3.506894 | -3.014710 | -0.589768 | 3.505562 | -3.029189 | -0.581575 |
| H | -0.189866 | -5.053382 | -1.277548 | -0.206272 | -5.040233 | -1.274168 | -0.205746 | -5.016491 | -1.341560 | -0.195495 | -5.011180 | -1.499951 | -0.209040 | -5.018158 | -1.347434 | -0.200314 | -5.042488 | -1.356742 |
| H | 2.261564 | -5.101945 | -1.018914 | 2.248458 | -5.096000 | -1.042103 | 2.248640 | -5.077207 | -1.098645 | 2.248191 | -5.082757 | -1.175968 | 2.244774 | -5.081317 | -1.097921 | 2.249307 | -5.103554 | -1.074188 |
| H | -5.115750 | -1.438068 | -1.244626 | -5.111753 | -1.375866 | -1.303932 | -5.107755 | -1.366188 | -1.306581 | -5.149384 | -1.379755 | -1.252655 | -5.106789 | -1.368903 | -1.300878 | -5.143235 | -1.384027 | -1.277186 |
| H | -4.970574 | 1.619545 | 1.740007 | -4.997056 | 1.573619 | 1.793378 | -5.003157 | 1.573194 | 1.805497 | -4.887415 | 1.593845 | 1.818047 | -4.999796 | 1.579636 | 1.802439 | -4.930506 | 1.590531 | 1.793768 |
| H | -7.438325 | -1.967846 | -0.590811 | -7.448177 | -1.913757 | -0.709533 | -7.447146 | -1.905570 | -0.724508 | -7.456746 | -1.913982 | -0.544257 | -7.444476 | -1.910283 | -0.711876 | -7.460984 | -1.913919 | -0.608432 |
| H | -7.301554 | 1.111421 | 2.380834 | -7.342713 | 1.063615 | 2.372999 | -7.352631 | 1.064147 | 2.372342 | -7.205458 | 1.090094 | 2.510379 | -7.347093 | 1.068315 | 2.376492 | -7.258769 | 1.089216 | 2.447344 |
| H | 3.718622 | -1.616600 | 1.592993 | 3.738982 | -1.584705 | 1.631742 | 3.733880 | -1.564322 | 1.655087 | 3.650656 | -1.562574 | 1.640751 | 3.729856 | -1.563690 | 1.658865 | 3.687501 | -1.560721 | 1.644843 |
| H | 3.724953 | 1.230154 | -1.593685 | 3.725867 | 1.178552 | -1.631499 | 3.734115 | 1.148208 | -1.654473 | 3.773966 | 1.139261 | -1.676014 | 3.733763 | 1.143310 | -1.654937 | 3.756954 | 1.135319 | -1.675035 |
| H | 6.081458 | -1.093563 | 2.070185 | 6.108745 | -1.062783 | 2.069112 | 6.104609 | -1.046038 | 2.085455 | 6.001023 | -1.037677 | 2.161277 | 6.099883 | -1.042891 | 2.092769 | 6.045935 | -1.031551 | 2.129511 |
| H | 6.081323 | 1.774971 | -1.103817 | 6.085787 | 1.727712 | -1.181069 | 6.092619 | 1.698465 | -1.211472 | 6.113378 | 1.695998 | -1.143397 | 6.092345 | 1.695517 | -1.209181 | 6.105180 | 1.694567 | -1.178300 |

| **iso-I** 23 |  | gasphase |  |  | toluene |  |  | DMSO |  |  | methanol |  |  | acetonitrile |  |  | chloroform |  |
| --- | --- | --- | --- | --- | --- | --- | --- | --- | --- | --- | --- | --- | --- | --- | --- | --- | --- | --- |
| Element | x | y | z | x | y | z | x | y | z | x | y | z | x | y | z | x | y | z |
| C | 0.020883 | 2.957791 | -0.718001 | 0.018520 | 2.947550 | -0.709329 | 0.019204 | 2.926992 | -0.770322 | 0.022479 | 2.923276 | -0.857314 | 0.017208 | 2.933787 | -0.756903 | 0.015128 | 2.947005 | -0.716940 |
| C | 0.795482 | 4.102554 | -0.842656 | 0.790687 | 4.091790 | -0.851361 | 0.791442 | 4.069057 | -0.930489 | 0.797719 | 4.063914 | -1.012436 | 0.789299 | 4.076190 | -0.917258 | 0.786430 | 4.091181 | -0.866722 |
| C | 2.174537 | 4.044744 | -0.720882 | 2.170713 | 4.036962 | -0.730293 | 2.172169 | 4.016772 | -0.795437 | 2.175478 | 4.013364 | -0.848138 | 2.170290 | 4.023900 | -0.784609 | 2.166970 | 4.038447 | -0.741896 |
| C | 2.814763 | 2.835833 | -0.482390 | 2.813787 | 2.831873 | -0.479886 | 2.815969 | 2.817410 | -0.518776 | 2.815564 | 2.816760 | -0.549266 | 2.814543 | 2.824132 | -0.509686 | 2.811614 | 2.835730 | -0.482366 |
| C | 2.031367 | 1.708243 | -0.352274 | 2.032118 | 1.704371 | -0.336746 | 2.033183 | 1.692315 | -0.360436 | 2.029298 | 1.694873 | -0.394351 | 2.032070 | 1.698955 | -0.350806 | 2.030468 | 1.708773 | -0.332521 |
| C | 0.632078 | 1.737065 | -0.452168 | 0.632367 | 1.731536 | -0.428501 | 0.634428 | 1.720030 | -0.455923 | 0.633299 | 1.722429 | -0.513690 | 0.632921 | 1.726266 | -0.444675 | 0.630734 | 1.734614 | -0.423887 |
| N | 2.471031 | 0.390571 | -0.189690 | 2.472268 | 0.389012 | -0.161046 | 2.469783 | 0.378970 | -0.164265 | 2.459710 | 0.378973 | -0.183266 | 2.469249 | 0.385543 | -0.154490 | 2.470618 | 0.394471 | -0.148288 |
| C | 1.402714 | -0.490188 | -0.242873 | 1.405674 | -0.490620 | -0.186168 | 1.403846 | -0.497353 | -0.169520 | 1.395969 | -0.490501 | -0.202589 | 1.403597 | -0.489989 | -0.155175 | 1.405615 | -0.483443 | -0.158197 |
| C | 0.151681 | 0.350890 | -0.372359 | 0.153725 | 0.346842 | -0.329558 | 0.155143 | 0.340271 | -0.336993 | 0.152835 | 0.345196 | -0.392412 | 0.154345 | 0.345871 | -0.325530 | 0.153842 | 0.350884 | -0.315274 |
| C | -1.097648 | -0.177411 | -0.337452 | -1.095965 | -0.179609 | -0.294220 | -1.096351 | -0.176561 | -0.297509 | -1.098363 | -0.170111 | -0.353340 | -1.096130 | -0.174231 | -0.290908 | -1.094850 | -0.176264 | -0.283430 |
| O | 1.517584 | -1.684515 | -0.123582 | 1.517087 | -1.682367 | -0.029734 | 1.504719 | -1.684999 | 0.032786 | 1.488350 | -1.680994 | 0.012869 | 1.504746 | -1.677211 | 0.051569 | 1.514176 | -1.673201 | 0.024207 |
| C | -1.589201 | -1.553449 | -0.474883 | -1.587677 | -1.553908 | -0.444424 | -1.591860 | -1.544715 | -0.468706 | -1.593083 | -1.535422 | -0.534734 | -1.587475 | -1.544504 | -0.461223 | -1.582906 | -1.550534 | -0.441784 |
| C | -2.976454 | -1.527941 | -0.259390 | -2.977232 | -1.526587 | -0.245402 | -2.979552 | -1.516638 | -0.262316 | -2.974123 | -1.515298 | -0.293957 | -2.975745 | -1.519876 | -0.258163 | -2.972645 | -1.526626 | -0.246087 |
| N | -3.394157 | -0.228830 | 0.030265 | -3.394210 | -0.228952 | 0.049057 | -3.388582 | -0.222927 | 0.059118 | -3.376523 | -0.223354 | 0.056452 | -3.389036 | -0.226103 | 0.058951 | -3.391543 | -0.230486 | 0.054157 |
| C | -2.335499 | 0.651809 | -0.039955 | -2.333785 | 0.646908 | 0.008623 | -2.325472 | 0.647174 | 0.037483 | -2.319600 | 0.644770 | 0.012882 | -2.328993 | 0.646613 | 0.035626 | -2.333419 | 0.645254 | 0.022672 |
| C | -1.010233 | -2.756101 | -0.867024 | -1.006813 | -2.754725 | -0.839074 | -1.014470 | -2.734063 | -0.900444 | -1.022368 | -2.709633 | -1.013172 | -1.006140 | -2.733189 | -0.890025 | -0.998601 | -2.746192 | -0.847014 |
| C | -1.800554 | -3.889150 | -0.999179 | -1.797428 | -3.885911 | -0.985864 | -1.808219 | -3.860892 | -1.067156 | -1.816783 | -3.835463 | -1.182015 | -1.796728 | -3.862509 | -1.056301 | -1.787036 | -3.878079 | -1.003115 |
| C | -3.164185 | -3.836560 | -0.756084 | -3.163684 | -3.832597 | -0.753765 | -3.173494 | -3.810527 | -0.819624 | -3.174763 | -3.795674 | -0.894881 | -3.162699 | -3.815433 | -0.811298 | -3.154117 | -3.829232 | -0.770533 |
| C | -3.774929 | -2.644783 | -0.388940 | -3.776540 | -2.641634 | -0.386806 | -3.782632 | -2.626994 | -0.421542 | -3.778356 | -2.623075 | -0.456442 | -3.775751 | -2.632533 | -0.416758 | -3.770205 | -2.641853 | -0.396004 |
| O | -2.428152 | 1.834852 | 0.175415 | -2.421076 | 1.825713 | 0.256070 | -2.398716 | 1.817587 | 0.335365 | -2.386033 | 1.817269 | 0.324286 | -2.407594 | 1.819294 | 0.325037 | -2.420414 | 1.822977 | 0.283698 |
| C | 3.798554 | -0.010685 | 0.069055 | 3.806341 | -0.009230 | 0.076556 | 3.804459 | -0.014324 | 0.084217 | 3.789159 | -0.017341 | 0.094604 | 3.805026 | -0.010297 | 0.086401 | 3.806629 | -0.004530 | 0.082246 |
| C | 4.376915 | -1.030259 | -0.676931 | 4.386700 | -0.994660 | -0.712111 | 4.397123 | -0.985116 | -0.713326 | 4.394194 | -0.992083 | -0.688143 | 4.392113 | -0.978416 | -0.718769 | 4.383194 | -0.980362 | -0.721076 |
| C | 5.673618 | -1.425425 | -0.413277 | 5.688056 | -1.389318 | -0.472167 | 5.698046 | -1.376322 | -0.464218 | 5.688508 | -1.386347 | -0.410134 | 5.693449 | -1.373540 | -0.477460 | 5.684708 | -1.379583 | -0.488826 |
| C | 6.404162 | -0.796665 | 0.592209 | 6.416486 | -0.789491 | 0.552860 | 6.407147 | -0.784018 | 0.579117 | 6.376526 | -0.792610 | 0.646095 | 6.408439 | -0.787711 | 0.565743 | 6.413522 | -0.791879 | 0.543310 |
| C | 5.821871 | 0.223240 | 1.340905 | 5.833884 | 0.199444 | 1.342554 | 5.813555 | 0.193222 | 1.375684 | 5.769464 | 0.188232 | 1.427862 | 5.820439 | 0.187067 | 1.369677 | 5.835377 | 0.189189 | 1.346385 |
| C | 4.520752 | 0.607453 | 1.084003 | 4.528129 | 0.581661 | 1.107279 | 4.508592 | 0.571407 | 1.129422 | 4.471096 | 0.569638 | 1.153212 | 4.514910 | 0.569107 | 1.131201 | 4.529428 | 0.575581 | 1.117922 |
| C | 7.753024 | -1.199478 | 0.858098 | 7.769234 | -1.191410 | 0.796842 | 7.757374 | -1.182795 | 0.835210 | 7.719898 | -1.193699 | 0.930789 | 7.759121 | -1.190822 | 0.814130 | 7.765150 | -1.199510 | 0.781340 |
| N | 8.835343 | -1.520282 | 1.070529 | 8.854919 | -1.511895 | 0.991723 | 8.841656 | -1.502879 | 1.041004 | 8.799154 | -1.515659 | 1.159202 | 8.843619 | -1.514312 | 1.013721 | 8.850285 | -1.525376 | 0.971911 |
| C | -6.664515 | 1.490191 | 0.018375 | -6.696052 | 1.422664 | -0.103022 | -6.702613 | 1.405635 | -0.073714 | -6.675904 | 1.439469 | 0.072049 | -6.711074 | 1.384987 | -0.099965 | -6.706594 | 1.392249 | -0.128135 |
| C | -5.378496 | 1.123733 | -0.342542 | -5.400742 | 1.054800 | -0.429147 | -5.409363 | 1.038944 | -0.412785 | -5.395796 | 1.073843 | -0.314618 | -5.415556 | 1.019706 | -0.432517 | -5.408641 | 1.026798 | -0.448470 |
| C | -4.713174 | 0.150227 | 0.387731 | -4.722673 | 0.151677 | 0.375900 | -4.714944 | 0.154752 | 0.399975 | -4.691960 | 0.150125 | 0.444219 | -4.717898 | 0.150243 | 0.393407 | -4.722840 | 0.147307 | 0.376236 |
| C | -5.329434 | -0.454104 | 1.475118 | -5.333169 | -0.385819 | 1.500432 | -5.304813 | -0.369501 | 1.541628 | -5.257770 | -0.414384 | 1.578658 | -5.306214 | -0.360510 | 1.542039 | -5.326420 | -0.370768 | 1.513509 |
| C | -6.621168 | -0.093436 | 1.820921 | -6.633806 | -0.024467 | 1.812454 | -6.603161 | -0.008744 | 1.867134 | -6.543502 | -0.054110 | 1.950669 | -6.606702 | -0.001279 | 1.861246 | -6.629412 | -0.011698 | 1.820157 |
| C | -7.290288 | 0.880365 | 1.095088 | -7.315792 | 0.881419 | 1.013575 | -7.301835 | 0.880822 | 1.062933 | -7.252199 | 0.874425 | 1.201036 | -7.308879 | 0.873399 | 1.043595 | -7.319710 | 0.871439 | 1.002322 |
| H | -1.049229 | 3.017377 | -0.809222 | -1.052060 | 3.004997 | -0.800915 | -1.050732 | 2.980009 | -0.880603 | -1.044110 | 2.972657 | -1.003203 | -1.052885 | 2.986476 | -0.865427 | -1.055372 | 3.002184 | -0.815259 |
| H | 0.310912 | 5.049810 | -1.038099 | 0.304504 | 5.036083 | -1.058503 | 0.308035 | 5.008762 | -1.164864 | 0.319694 | 5.000749 | -1.268427 | 0.305395 | 5.016071 | -1.149834 | 0.300106 | 5.033453 | -1.083346 |
| H | 2.765382 | 4.946051 | -0.823224 | 2.759860 | 4.938410 | -0.842825 | 2.761411 | 4.916674 | -0.920613 | 2.766469 | 4.912544 | -0.970738 | 2.759323 | 4.923900 | -0.910003 | 2.755271 | 4.939918 | -0.859496 |
| H | 3.892482 | 2.779337 | -0.420175 | 3.891835 | 2.779948 | -0.415663 | 3.893799 | 2.769038 | -0.444990 | 3.892157 | 2.768840 | -0.457236 | 3.892400 | 2.775521 | -0.436999 | 3.889682 | 2.785948 | -0.415053 |
| H | 0.047900 | -2.812925 | -1.051015 | 0.053032 | -2.812396 | -1.015432 | 0.043230 | -2.786659 | -1.095825 | 0.027618 | -2.751224 | -1.252256 | 0.051987 | -2.783277 | -1.083779 | 0.061131 | -2.800101 | -1.027880 |
| H | -4.841906 | -2.588089 | -0.224355 | -4.844931 | -2.585878 | -0.229010 | -4.849964 | -2.575530 | -0.253588 | -4.841857 | -2.578863 | -0.263688 | -4.843487 | -2.583024 | -0.250920 | -4.839036 | -2.589873 | -0.238838 |
| H | -1.341082 | -4.823042 | -1.294138 | -1.336776 | -4.819082 | -1.282420 | -1.353667 | -4.787382 | -1.393695 | -1.369569 | -4.752205 | -1.544330 | -1.339090 | -4.788315 | -1.380413 | -1.324908 | -4.807982 | -1.308110 |
| H | -3.766611 | -4.729884 | -0.862084 | -3.766077 | -4.725113 | -0.868385 | -3.778284 | -4.699545 | -0.949126 | -3.779808 | -4.684000 | -1.028304 | -3.764819 | -4.706332 | -0.940234 | -3.754537 | -4.722281 | -0.891951 |
| H | 3.806388 | -1.515066 | -1.455276 | 3.817887 | -1.450795 | -1.509518 | 3.842887 | -1.427924 | -1.528959 | 3.855018 | -1.435198 | -1.514268 | 3.832703 | -1.415878 | -1.533943 | 3.812733 | -1.423857 | -1.525081 |
| H | 4.056347 | 1.383703 | 1.677142 | 4.061944 | 1.334577 | 1.728687 | 4.032605 | 1.317996 | 1.751146 | 3.983536 | 1.319138 | 1.762486 | 4.043006 | 1.313849 | 1.758217 | 4.065193 | 1.322896 | 1.747623 |
| H | 6.128145 | -2.219160 | -0.990307 | 6.145326 | -2.157004 | -1.081597 | 6.167916 | -2.131098 | -1.080310 | 6.169436 | -2.144494 | -1.013598 | 6.159132 | -2.126351 | -1.099095 | 6.140745 | -2.139702 | -1.108700 |
| H | 6.386535 | 0.701655 | 2.129513 | 6.399608 | 0.655281 | 2.143825 | 6.368337 | 0.644106 | 2.187424 | 6.308911 | 0.639405 | 2.249860 | 6.379908 | 0.632799 | 2.181046 | 6.403508 | 0.635329 | 2.151497 |
| H | -7.181041 | 2.253858 | -0.548551 | -7.224079 | 2.131768 | -0.727783 | -7.245513 | 2.098302 | -0.704533 | -7.226305 | 2.163302 | -0.515929 | -7.256619 | 2.066107 | -0.740968 | -7.241653 | 2.082463 | -0.768101 |
| H | -4.883634 | 1.594796 | -1.180453 | -4.911170 | 1.467199 | -1.301343 | -4.937294 | 1.433374 | -1.303410 | -4.941241 | 1.500265 | -1.199836 | -4.943908 | 1.403952 | -1.327833 | -4.924369 | 1.421431 | -1.332190 |
| H | -8.297176 | 1.166169 | 1.370565 | -8.329815 | 1.167344 | 1.262731 | -8.314254 | 1.164731 | 1.321780 | -8.254685 | 1.157791 | 1.496942 | -8.322875 | 1.156127 | 1.297490 | -8.335610 | 1.155046 | 1.247078 |
| H | -4.793277 | -1.201241 | 2.046246 | -4.787369 | -1.081270 | 2.125506 | -4.747597 | -1.055860 | 2.167003 | -4.692834 | -1.130470 | 2.162448 | -4.746112 | -1.035288 | 2.177330 | -4.774619 | -1.049821 | 2.151389 |
| H | -7.100846 | -0.567301 | 2.667581 | -7.111051 | -0.445036 | 2.688432 | -7.066340 | -0.419287 | 2.755536 | -6.988840 | -0.495527 | 2.833462 | -7.068811 | -0.401188 | 2.755030 | -7.102351 | -0.416898 | 2.705790 |

| **iso-I** 24 |  | gasphase |  |  | toluene |  |  | DMSO |  |  | methanol |  |  | acetonitrile |  |  | chloroform |  |
| --- | --- | --- | --- | --- | --- | --- | --- | --- | --- | --- | --- | --- | --- | --- | --- | --- | --- | --- |
| Element | x | y | z | x | y | z | x | y | z | x | y | z | x | y | z | x | y | z |
| C | -0.976719 | 3.234235 | -0.189342 | -0.978494 | 3.223053 | -0.212662 | -0.981343 | 3.208805 | -0.247165 | -0.990028 | 3.201901 | -0.298437 | -0.982197 | 3.213993 | -0.245341 | -0.982744 | 3.218486 | -0.231117 |
| C | -0.092781 | 4.302762 | -0.254594 | -0.096390 | 4.290680 | -0.308534 | -0.100758 | 4.275919 | -0.365366 | -0.112161 | 4.270775 | -0.418157 | -0.101517 | 4.282047 | -0.356577 | -0.102022 | 4.286711 | -0.336674 |
| C | 1.275166 | 4.099927 | -0.177149 | 1.273386 | 4.087982 | -0.249462 | 1.271064 | 4.073349 | -0.313865 | 1.260204 | 4.072758 | -0.353443 | 1.270314 | 4.079713 | -0.302553 | 1.268867 | 4.085478 | -0.278917 |
| C | 1.795527 | 2.817119 | -0.064430 | 1.795911 | 2.807159 | -0.124504 | 1.796433 | 2.794645 | -0.175807 | 1.790987 | 2.796852 | -0.205494 | 1.795672 | 2.800310 | -0.168875 | 1.793787 | 2.806021 | -0.146570 |
| C | 0.906491 | 1.764026 | -0.024172 | 0.907074 | 1.755336 | -0.050340 | 0.907636 | 1.744329 | -0.076915 | 0.904671 | 1.745833 | -0.104975 | 0.906944 | 1.749340 | -0.076857 | 0.905752 | 1.754248 | -0.061835 |
| C | -0.486401 | 1.939801 | -0.051679 | -0.485751 | 1.931484 | -0.059128 | -0.484494 | 1.921371 | -0.074370 | -0.486534 | 1.920376 | -0.106004 | -0.485422 | 1.925787 | -0.076800 | -0.486958 | 1.929379 | -0.067002 |
| N | 1.213787 | 0.403313 | 0.063313 | 1.213623 | 0.395683 | 0.044861 | 1.212283 | 0.385443 | 0.024730 | 1.208475 | 0.383378 | -0.005068 | 1.211924 | 0.389823 | 0.020073 | 1.212541 | 0.394752 | 0.037316 |
| C | 0.059060 | -0.358466 | 0.075300 | 0.061925 | -0.365604 | 0.068779 | 0.063717 | -0.374910 | 0.050714 | 0.063151 | -0.372391 | 0.013271 | 0.063754 | -0.370367 | 0.042055 | 0.063316 | -0.366099 | 0.062602 |
| C | -1.105133 | 0.607244 | 0.004520 | -1.102938 | 0.599658 | 0.010985 | -1.100296 | 0.591595 | 0.004640 | -1.098175 | 0.591886 | -0.025772 | -1.100870 | 0.595232 | -0.002483 | -1.101845 | 0.597725 | 0.007985 |
| C | -2.400262 | 0.202082 | 0.044556 | -2.398312 | 0.198986 | 0.068631 | -2.395453 | 0.198698 | 0.082656 | -2.390273 | 0.199896 | 0.079325 | -2.395352 | 0.199689 | 0.075611 | -2.396237 | 0.197868 | 0.074491 |
| O | 0.056744 | -1.560082 | 0.176589 | 0.058530 | -1.568322 | 0.173635 | 0.056728 | -1.579751 | 0.155096 | 0.050486 | -1.581428 | 0.120704 | 0.057094 | -1.575845 | 0.142366 | 0.058435 | -1.570129 | 0.169648 |
| C | -3.024870 | -1.123829 | -0.105617 | -3.029812 | -1.119736 | -0.103820 | -3.034441 | -1.110818 | -0.110392 | -3.035332 | -1.100203 | -0.133810 | -3.031603 | -1.112125 | -0.113728 | -3.028915 | -1.117876 | -0.107468 |
| C | -4.404975 | -0.964283 | 0.106720 | -4.407112 | -0.956350 | 0.122939 | -4.404265 | -0.948260 | 0.156433 | -4.389570 | -0.951827 | 0.204378 | -4.400253 | -0.953961 | 0.161644 | -4.403112 | -0.956589 | 0.137357 |
| N | -4.679930 | 0.360929 | 0.396611 | -4.671937 | 0.362095 | 0.448365 | -4.655802 | 0.360209 | 0.528051 | -4.621302 | 0.345629 | 0.636716 | -4.653611 | 0.354694 | 0.532700 | -4.663570 | 0.357971 | 0.483661 |
| C | -3.560652 | 1.143564 | 0.347843 | -3.549664 | 1.135625 | 0.416855 | -3.533025 | 1.127849 | 0.490533 | -3.502900 | 1.108929 | 0.574134 | -3.534503 | 1.126120 | 0.484362 | -3.542266 | 1.128653 | 0.450342 |
| C | -2.571881 | -2.382825 | -0.488751 | -2.586654 | -2.370144 | -0.524653 | -2.605851 | -2.344618 | -0.591259 | -2.626984 | -2.305553 | -0.696013 | -2.602168 | -2.344770 | -0.597183 | -2.590241 | -2.360394 | -0.555795 |
| C | -3.473293 | -3.431916 | -0.610671 | -3.494786 | -3.410801 | -0.666796 | -3.521297 | -3.377637 | -0.745070 | -3.547006 | -3.334466 | -0.848156 | -3.515352 | -3.380986 | -0.744436 | -3.500434 | -3.398858 | -0.703242 |
| C | -4.824137 | -3.246585 | -0.360117 | -4.843273 | -3.223317 | -0.400579 | -4.862467 | -3.194969 | -0.432640 | -4.870469 | -3.171221 | -0.458201 | -4.855055 | -3.202676 | -0.422860 | -4.846035 | -3.215115 | -0.416123 |
| C | -5.311207 | -1.996559 | -0.002324 | -5.320740 | -1.980365 | -0.006879 | -5.325698 | -1.965192 | 0.016614 | -5.315109 | -1.963705 | 0.065932 | -5.319371 | -1.973923 | 0.028758 | -5.318985 | -1.978321 | 0.002826 |
| O | -3.562719 | 2.330060 | 0.571186 | -3.533015 | 2.313619 | 0.690526 | -3.493665 | 2.296685 | 0.808710 | -3.437983 | 2.265987 | 0.946022 | -3.498912 | 2.297742 | 0.794159 | -3.515859 | 2.303220 | 0.746064 |
| C | 2.510719 | -0.156202 | 0.066348 | 2.513753 | -0.160536 | 0.060210 | 2.515264 | -0.166884 | 0.054258 | 2.511999 | -0.170106 | 0.044401 | 2.514469 | -0.164071 | 0.051882 | 2.514153 | -0.160342 | 0.059111 |
| C | 3.409117 | 0.173068 | -0.941963 | 3.388570 | 0.102523 | -0.986831 | 3.377221 | 0.050167 | -1.013004 | 3.387029 | 0.040651 | -1.012938 | 3.380562 | 0.055398 | -1.011637 | 3.381715 | 0.078799 | -0.999346 |
| C | 4.679459 | -0.368863 | -0.940586 | 4.660441 | -0.435609 | -0.974129 | 4.650902 | -0.483812 | -0.985938 | 4.659660 | -0.494647 | -0.965400 | 4.653182 | -0.481442 | -0.982274 | 4.653702 | -0.459336 | -0.981058 |
| C | 5.051258 | -1.259437 | 0.063599 | 5.052148 | -1.253180 | 0.083730 | 5.050719 | -1.247365 | 0.108872 | 5.043404 | -1.252424 | 0.139092 | 5.047664 | -1.250479 | 0.110821 | 5.049412 | -1.251245 | 0.094941 |
| C | 4.143351 | -1.601302 | 1.063156 | 4.169422 | -1.525871 | 1.126630 | 4.182329 | -1.470820 | 1.175808 | 4.161287 | -1.469511 | 1.195682 | 4.175004 | -1.476440 | 1.173914 | 4.174684 | -1.498436 | 1.151103 |
| C | 2.878274 | -1.047156 | 1.066467 | 2.903163 | -0.974547 | 1.115571 | 2.914490 | -0.923465 | 1.147951 | 2.894535 | -0.920759 | 1.147928 | 2.908330 | -0.926113 | 1.143925 | 2.908357 | -0.946915 | 1.133342 |
| C | 6.367354 | -1.825911 | 0.065621 | 6.368722 | -1.817213 | 0.097696 | 6.366955 | -1.808810 | 0.137343 | 6.357989 | -1.815265 | 0.188422 | 6.362745 | -1.815172 | 0.141437 | 6.364870 | -1.816539 | 0.114105 |
| N | 7.423349 | -2.277850 | 0.066966 | 7.425315 | -2.267500 | 0.108519 | 7.423829 | -2.259186 | 0.159704 | 7.413870 | -2.266944 | 0.227873 | 7.418548 | -2.268108 | 0.165537 | 7.420967 | -2.268571 | 0.129010 |
| H | -5.585164 | 0.746112 | 0.596156 | -5.576554 | 0.738391 | 0.674733 | -5.559015 | 0.723232 | 0.788281 | -5.503245 | 0.695701 | 0.977326 | -5.555627 | 0.716760 | 0.798475 | -5.566575 | 0.728580 | 0.729620 |
| H | -2.037622 | 3.407389 | -0.222744 | -2.039928 | 3.396503 | -0.237497 | -2.043674 | 3.380259 | -0.275029 | -2.053064 | 3.369811 | -0.350379 | -2.044502 | 3.384998 | -0.275617 | -2.044678 | 3.390480 | -0.259888 |
| H | -0.484455 | 5.306178 | -0.354745 | -0.489248 | 5.292769 | -0.420120 | -0.493500 | 5.276551 | -0.492669 | -0.506421 | 5.268941 | -0.559398 | -0.494372 | 5.283053 | -0.480417 | -0.495707 | 5.287725 | -0.456368 |
| H | 1.951013 | 4.945036 | -0.208023 | 1.948894 | 4.932310 | -0.306478 | 1.945714 | 4.916894 | -0.391681 | 1.931934 | 4.918573 | -0.432644 | 1.944915 | 4.923774 | -0.374858 | 1.943239 | 4.930213 | -0.344600 |
| H | 2.862267 | 2.652287 | -0.007086 | 2.864015 | 2.643538 | -0.088280 | 2.865306 | 2.631858 | -0.154842 | 2.860499 | 2.637973 | -0.180210 | 2.864455 | 2.637345 | -0.145987 | 2.862393 | 2.643567 | -0.115478 |
| H | -1.525988 | -2.549873 | -0.674513 | -1.543193 | -2.537628 | -0.725739 | -1.569471 | -2.504770 | -0.834207 | -1.606414 | -2.446764 | -1.011243 | -1.566980 | -2.501262 | -0.847652 | -1.549589 | -2.523592 | -0.776550 |
| H | -6.366367 | -1.833714 | 0.175033 | -6.373434 | -1.814569 | 0.182410 | -6.371953 | -1.799700 | 0.238769 | -6.350248 | -1.813042 | 0.343822 | -6.364697 | -1.811661 | 0.257530 | -6.369174 | -1.814151 | 0.206862 |
| H | -3.109168 | -4.408293 | -0.901050 | -3.139179 | -4.381664 | -0.986028 | -3.180272 | -4.337437 | -1.111446 | -3.224784 | -4.274945 | -1.276506 | -3.173601 | -4.339698 | -1.112928 | -3.149848 | -4.364496 | -1.043777 |
| H | -5.510109 | -4.079141 | -0.451552 | -5.535197 | -4.049290 | -0.508533 | -5.560286 | -4.014826 | -0.550839 | -5.571480 | -3.988277 | -0.577326 | -5.550935 | -4.024884 | -0.535952 | -5.539512 | -4.039331 | -0.528677 |
| H | 3.106360 | 0.847494 | -1.731613 | 3.067954 | 0.722412 | -1.813518 | 3.048235 | 0.632403 | -1.863514 | 3.069761 | 0.618252 | -1.871141 | 3.055714 | 0.641821 | -1.860837 | 3.056668 | 0.680212 | -1.837961 |
| H | 2.168255 | -1.308989 | 1.837343 | 2.213161 | -1.175268 | 1.922799 | 2.234781 | -1.078279 | 1.974360 | 2.202799 | -1.070261 | 1.965794 | 2.224994 | -1.083120 | 1.966956 | 2.224006 | -1.124824 | 1.951028 |
| H | 5.382402 | -0.115406 | -1.722408 | 5.346485 | -0.234860 | -1.785822 | 5.329694 | -0.319796 | -1.811855 | 5.350260 | -0.336535 | -1.782812 | 5.335189 | -0.315643 | -1.805177 | 5.335484 | -0.278153 | -1.800963 |
| H | 4.435142 | -2.296594 | 1.838366 | 4.479560 | -2.162244 | 1.944319 | 4.503722 | -2.062124 | 2.022634 | 4.470830 | -2.057007 | 2.049685 | 4.492095 | -2.072144 | 2.019263 | 4.490188 | -2.113844 | 1.982795 |

| **iso-I** 25 |  | gasphase |  |  | toluene |  |  | DMSO |  |  | methanol |  |  | acetonitrile |  |  | chloroform |  |
| --- | --- | --- | --- | --- | --- | --- | --- | --- | --- | --- | --- | --- | --- | --- | --- | --- | --- | --- |
| Element | x | y | z | x | y | z | x | y | z | x | y | z | x | y | z | x | y | z |
| C | 0.435870 | -2.880648 | -0.851154 | 0.422970 | -2.877620 | -0.765502 | 0.426325 | -2.863099 | -0.837973 | 0.440034 | -2.838612 | -1.060030 | 0.420468 | -2.865895 | -0.841517 | 0.416663 | -2.873280 | -0.810915 |
| C | 1.190348 | -4.039646 | -0.976242 | 1.174007 | -4.034912 | -0.923600 | 1.178022 | -4.019758 | -1.000780 | 1.194354 | -3.993993 | -1.216348 | 1.170145 | -4.024711 | -1.000031 | 1.165100 | -4.032687 | -0.967995 |
| C | 2.566697 | -4.013810 | -0.815582 | 2.554479 | -4.010812 | -0.794174 | 2.557277 | -3.999650 | -0.844086 | 2.565087 | -3.982936 | -0.994974 | 2.549050 | -4.007716 | -0.838864 | 2.544581 | -4.014668 | -0.820618 |
| C | 3.225928 | -2.823131 | -0.539627 | 3.220462 | -2.823226 | -0.520395 | 3.221352 | -2.816933 | -0.543375 | 3.221162 | -2.810703 | -0.638284 | 3.214976 | -2.825831 | -0.537978 | 3.212415 | -2.831154 | -0.532114 |
| C | 2.462999 | -1.680789 | -0.418448 | 2.460051 | -1.682574 | -0.369246 | 2.459526 | -1.677175 | -0.390098 | 2.456480 | -1.673439 | -0.489647 | 2.455166 | -1.684248 | -0.388992 | 2.454262 | -1.688784 | -0.382691 |
| C | 1.065200 | -1.677005 | -0.551688 | 1.059304 | -1.677593 | -0.466040 | 1.060731 | -1.671009 | -0.504818 | 1.065037 | -1.662353 | -0.661739 | 1.056660 | -1.674627 | -0.508107 | 1.054766 | -1.678772 | -0.493150 |
| N | 2.929005 | -0.384803 | -0.206025 | 2.930997 | -0.390696 | -0.147768 | 2.926346 | -0.387180 | -0.150585 | 2.911630 | -0.382230 | -0.212384 | 2.924455 | -0.394831 | -0.149639 | 2.926285 | -0.398293 | -0.152212 |
| C | 1.891618 | 0.521391 | -0.245288 | 1.894953 | 0.512587 | -0.131315 | 1.891893 | 0.513801 | -0.135345 | 1.879841 | 0.514082 | -0.238525 | 1.892859 | 0.508431 | -0.140874 | 1.894095 | 0.506107 | -0.143068 |
| C | 0.617626 | -0.280694 | -0.443530 | 0.616746 | -0.282201 | -0.331695 | 0.617422 | -0.279017 | -0.359754 | 0.618288 | -0.275463 | -0.510788 | 0.616586 | -0.281005 | -0.365580 | 0.615975 | -0.283190 | -0.355966 |
| C | -0.617629 | 0.280693 | -0.443541 | -0.616745 | 0.282179 | -0.331691 | -0.617421 | 0.279026 | -0.359752 | -0.618285 | 0.275444 | -0.510777 | -0.616581 | 0.281003 | -0.365557 | -0.615966 | 0.283174 | -0.355928 |
| O | 2.033438 | 1.708622 | -0.080328 | 2.035171 | 1.693965 | 0.081768 | 2.024406 | 1.693362 | 0.103826 | 1.998883 | 1.696711 | 0.017859 | 2.028522 | 1.689167 | 0.092936 | 2.034284 | 1.687886 | 0.077869 |
| C | -1.065203 | 1.677004 | -0.551700 | -1.059298 | 1.677572 | -0.466047 | -1.060732 | 1.671017 | -0.504814 | -1.065030 | 1.662335 | -0.661733 | -1.056656 | 1.674624 | -0.508088 | -1.054756 | 1.678755 | -0.493122 |
| C | -2.463001 | 1.680788 | -0.418464 | -2.460045 | 1.682560 | -0.369253 | -2.459528 | 1.677180 | -0.390092 | -2.456472 | 1.673429 | -0.489640 | -2.455160 | 1.684249 | -0.388962 | -2.454251 | 1.688774 | -0.382654 |
| N | -2.929009 | 0.384802 | -0.206051 | -2.930996 | 0.390686 | -0.147760 | -2.926345 | 0.387184 | -0.150579 | -2.911627 | 0.382226 | -0.212358 | -2.924449 | 0.394837 | -0.149581 | -2.926274 | 0.398291 | -0.152122 |
| C | -1.891626 | -0.521396 | -0.245345 | -1.894955 | -0.512601 | -0.131288 | -1.891890 | -0.513795 | -0.135340 | -1.879837 | -0.514086 | -0.238459 | -1.892845 | -0.508416 | -0.140744 | -1.894075 | -0.506096 | -0.142856 |
| C | -0.435869 | 2.880650 | -0.851151 | -0.422957 | 2.877594 | -0.765509 | -0.426330 | 2.863108 | -0.837972 | -0.440020 | 2.838586 | -1.060039 | -0.420467 | 2.865880 | -0.841547 | -0.416654 | 2.873246 | -0.810946 |
| C | -1.190346 | 4.039650 | -0.976231 | -1.173989 | 4.034889 | -0.923615 | -1.178030 | 4.019766 | -1.000777 | -1.194335 | 3.993968 | -1.216371 | -1.170144 | 4.024691 | -1.000090 | -1.165090 | 4.032650 | -0.968065 |
| C | -2.566695 | 4.013814 | -0.815575 | -2.554461 | 4.010795 | -0.794198 | -2.557285 | 3.999655 | -0.844079 | -2.565069 | 3.982919 | -0.994999 | -2.549047 | 4.007703 | -0.838910 | -2.544570 | 4.014638 | -0.820683 |
| C | -3.225928 | 2.823134 | -0.539631 | -3.220451 | 2.823213 | -0.520414 | -3.221356 | 2.816936 | -0.543367 | -3.221150 | 2.810694 | -0.638295 | -3.214971 | 2.825826 | -0.537985 | -3.212405 | 2.831136 | -0.532133 |
| O | -2.033443 | -1.708622 | -0.080351 | -2.035180 | -1.693973 | 0.081815 | -2.024401 | -1.693358 | 0.103824 | -1.998889 | -1.696716 | 0.017913 | -2.028514 | -1.689163 | 0.093007 | -2.034276 | -1.687890 | 0.077998 |
| C | 4.278293 | -0.028555 | 0.046179 | 4.293351 | -0.039366 | 0.048476 | 4.288164 | -0.038572 | 0.058949 | 4.257649 | -0.031494 | 0.081250 | 4.287163 | -0.047313 | 0.059575 | 4.287930 | -0.048014 | 0.054485 |
| C | 4.935025 | -0.539888 | 1.150107 | 4.952018 | -0.430681 | 1.199311 | 4.922299 | -0.394889 | 1.235268 | 4.855449 | -0.490875 | 1.241112 | 4.909066 | -0.364961 | 1.253534 | 4.935549 | -0.432563 | 1.214247 |
| C | 6.259420 | -0.210290 | 1.402616 | 6.287738 | -0.107554 | 1.393354 | 6.256304 | -0.072762 | 1.443078 | 6.171622 | -0.162197 | 1.532868 | 6.243151 | -0.042439 | 1.461353 | 6.268916 | -0.106007 | 1.419249 |
| C | 6.925859 | 0.659503 | 0.546725 | 6.963878 | 0.630294 | 0.426577 | 6.956198 | 0.624975 | 0.461816 | 6.886124 | 0.648245 | 0.655932 | 6.955049 | 0.615392 | 0.461271 | 6.952840 | 0.627377 | 0.453991 |
| C | 6.257904 | 1.184598 | -0.559002 | 6.294466 | 1.030178 | -0.730572 | 6.311019 | 0.986516 | -0.722879 | 6.278576 | 1.115236 | -0.510107 | 6.322448 | 0.937855 | -0.741358 | 6.294482 | 1.019370 | -0.712587 |
| C | 4.948230 | 0.838449 | -0.809633 | 4.971923 | 0.692121 | -0.919311 | 4.988528 | 0.651057 | -0.923918 | 4.974301 | 0.771540 | -0.798237 | 4.999516 | 0.603147 | -0.941410 | 4.973724 | 0.678515 | -0.911998 |
| O | 8.209804 | 1.051691 | 0.702870 | 8.257052 | 1.002592 | 0.519361 | 8.248944 | 0.989829 | 0.567118 | 8.168021 | 1.032431 | 0.849693 | 8.249247 | 0.977170 | 0.563341 | 8.244620 | 1.002510 | 0.557083 |
| C | 8.928575 | 0.564545 | 1.811166 | 8.982417 | 0.632226 | 1.671987 | 8.948962 | 0.647008 | 1.750458 | 8.834653 | 0.594170 | 2.022916 | 8.940678 | 0.677276 | 1.763552 | 8.959758 | 0.645767 | 1.724023 |
| C | -4.278295 | 0.028555 | 0.046165 | -4.293352 | 0.039365 | 0.048484 | -4.288162 | 0.038572 | 0.058952 | -4.257650 | 0.031497 | 0.081264 | -4.287159 | 0.047315 | 0.059610 | -4.287925 | 0.048014 | 0.054538 |
| C | -4.935012 | 0.539871 | 1.150110 | -4.952026 | 0.430705 | 1.199306 | -4.922299 | 0.394883 | 1.235272 | -4.855465 | 0.490902 | 1.241109 | -4.909112 | 0.365051 | 1.253519 | -4.935608 | 0.432672 | 1.214228 |
| C | -6.259406 | 0.210274 | 1.402629 | -6.287750 | 0.107589 | 1.393345 | -6.256304 | 0.072752 | 1.443080 | -6.171642 | 0.162231 | 1.532855 | -6.243200 | 0.042526 | 1.461315 | -6.268981 | 0.106120 | 1.419195 |
| C | -6.925858 | -0.659501 | 0.546730 | -6.963886 | -0.630273 | 0.426576 | -6.956195 | -0.624983 | 0.461815 | -6.886133 | -0.648229 | 0.655926 | -6.955050 | -0.615400 | 0.461260 | -6.952845 | -0.627374 | 0.453978 |
| C | -6.257919 | -1.184579 | -0.559014 | -6.294467 | -1.030184 | -0.730559 | -6.311014 | -0.986518 | -0.722882 | -6.278570 | -1.115243 | -0.510096 | -6.322398 | -0.937951 | -0.741317 | -6.294423 | -1.019477 | -0.712526 |
| C | -4.948246 | -0.838432 | -0.809655 | -4.971921 | -0.692139 | -0.919294 | -4.988524 | -0.651055 | -0.923918 | -4.974291 | -0.771554 | -0.798216 | -4.999465 | -0.603239 | -0.941348 | -4.973660 | -0.678623 | -0.911905 |
| O | -8.209803 | -1.051686 | 0.702884 | -8.257063 | -1.002561 | 0.519357 | -8.248940 | -0.989839 | 0.567113 | -8.168033 | -1.032411 | 0.849678 | -8.249245 | -0.977190 | 0.563313 | -8.244626 | -1.002515 | 0.557043 |
| C | -8.928559 | -0.564556 | 1.811196 | -8.982436 | -0.632171 | 1.671970 | -8.948961 | -0.647024 | 1.750453 | -8.834681 | -0.594130 | 2.022883 | -8.940720 | -0.677224 | 1.763481 | -8.959828 | -0.645659 | 1.723909 |
| H | -0.632923 | -2.916655 | -0.966786 | -0.648541 | -2.913560 | -0.857172 | -0.643187 | -2.894275 | -0.957619 | -0.621137 | -2.859174 | -1.246337 | -0.648712 | -2.894653 | -0.965097 | -0.653640 | -2.904054 | -0.921142 |
| H | 0.690953 | -4.973146 | -1.199242 | 0.669367 | -4.966013 | -1.146370 | 0.677596 | -4.946563 | -1.250663 | 0.704438 | -4.911673 | -1.515735 | 0.668280 | -4.950655 | -1.250141 | 0.660226 | -4.960272 | -1.205071 |
| H | 3.139720 | -4.927264 | -0.913832 | 3.124532 | -4.923634 | -0.915000 | 3.127894 | -4.911901 | -0.967276 | 3.137615 | -4.894349 | -1.115748 | 3.117849 | -4.921528 | -0.958725 | 3.112499 | -4.929032 | -0.940313 |
| H | 4.301609 | -2.785105 | -0.436465 | 4.298395 | -2.787317 | -0.438452 | 4.298094 | -2.786357 | -0.442317 | 4.293241 | -2.788616 | -0.494808 | 4.291429 | -2.796870 | -0.433423 | 4.289631 | -2.800875 | -0.437825 |
| H | 0.632925 | 2.916658 | -0.966774 | 0.648555 | 2.913529 | -0.857167 | 0.643181 | 2.894286 | -0.957621 | 0.621150 | 2.859139 | -1.246348 | 0.648711 | 2.894629 | -0.965150 | 0.653648 | 2.904011 | -0.921192 |
| H | -4.301610 | 2.785108 | -0.436471 | -4.298383 | 2.787310 | -0.438474 | -4.298098 | 2.786358 | -0.442306 | -4.293228 | 2.788613 | -0.494820 | -4.291424 | 2.796869 | -0.433425 | -4.289620 | 2.800863 | -0.437843 |
| H | -0.690948 | 4.973152 | -1.199219 | -0.669343 | 4.965987 | -1.146385 | -0.677607 | 4.946571 | -1.250662 | -0.704415 | 4.911642 | -1.515770 | -0.668280 | 4.950626 | -1.250238 | -0.660215 | 4.960223 | -1.205185 |
| H | -3.139717 | 4.927270 | -0.913817 | -3.124510 | 4.923618 | -0.915033 | -3.127904 | 4.911905 | -0.967268 | -3.137594 | 4.894334 | -1.115786 | -3.117847 | 4.921511 | -0.958796 | -3.112488 | 4.928997 | -0.940417 |
| H | 4.409132 | -1.205245 | 1.823167 | 4.419356 | -0.994791 | 1.954812 | 4.373354 | -0.932178 | 1.998913 | 4.292839 | -1.113972 | 1.925470 | 4.350303 | -0.871742 | 2.030740 | 4.396874 | -0.993849 | 1.967734 |
| H | 4.430972 | 1.247840 | -1.666653 | 4.454060 | 0.998360 | -1.819031 | 4.490857 | 0.923704 | -1.846207 | 4.505200 | 1.126737 | -1.707240 | 4.510360 | 0.846123 | -1.876516 | 4.464105 | 0.978177 | -1.818929 |
| H | 6.748373 | -0.627588 | 2.270674 | 6.780910 | -0.426826 | 2.300094 | 6.731298 | -0.364744 | 2.368647 | 6.618920 | -0.536287 | 2.442462 | 6.709180 | -0.302634 | 2.400849 | 6.754731 | -0.419448 | 2.332064 |
| H | 6.789545 | 1.863136 | -1.212528 | 6.832388 | 1.604540 | -1.473452 | 6.864756 | 1.526725 | -1.480292 | 6.846966 | 1.743458 | -1.184428 | 6.886571 | 1.447842 | -1.511888 | 6.838433 | 1.589536 | -1.454631 |
| H | 9.014072 | -0.526359 | 1.785336 | 9.045103 | -0.455470 | 1.774444 | 8.992314 | -0.436851 | 1.888025 | 8.903404 | -0.496303 | 2.054389 | 8.978233 | -0.400708 | 1.942316 | 9.022681 | -0.440370 | 1.837229 |
| H | 8.464130 | 0.868200 | 2.754473 | 8.542467 | 1.059822 | 2.578014 | 8.494039 | 1.109314 | 2.630737 | 8.331631 | 0.958380 | 2.922448 | 8.481249 | 1.174736 | 2.621932 | 8.506330 | 1.081999 | 2.618682 |
| H | 9.921676 | 1.002360 | 1.744397 | 9.984887 | 1.034352 | 1.540313 | 9.958636 | 1.032396 | 1.625219 | 9.835816 | 1.016963 | 1.973222 | 9.952350 | 1.054212 | 1.629413 | 9.961919 | 1.050173 | 1.597928 |
| H | -4.409108 | 1.205214 | 1.823175 | -4.419367 | 0.994827 | 1.954800 | -4.373356 | 0.932170 | 1.998919 | -4.292864 | 1.114012 | 1.925462 | -4.350387 | 0.871905 | 2.030706 | -4.396980 | 0.994043 | 1.967685 |
| H | -4.431000 | -1.247810 | -1.666688 | -4.454051 | -0.998398 | -1.819004 | -4.490851 | -0.923698 | -1.846207 | -4.505178 | -1.126770 | -1.707206 | -4.510271 | -0.846283 | -1.876416 | -4.463991 | -0.978370 | -1.818781 |
| H | -6.748346 | 0.627559 | 2.270699 | -6.780927 | 0.426881 | 2.300075 | -6.731299 | 0.364729 | 2.368649 | -6.618951 | 0.536338 | 2.442436 | -6.709268 | 0.302791 | 2.400772 | -6.754846 | 0.419647 | 2.331954 |
| H | -6.789570 | -1.863104 | -1.212546 | -6.832385 | -1.604559 | -1.473432 | -6.864750 | -1.526724 | -1.480297 | -6.846952 | -1.743478 | -1.184412 | -6.886484 | -1.448011 | -1.511827 | -6.838328 | -1.589727 | -1.454539 |
| H | -8.464106 | -0.868229 | 2.754492 | -8.542495 | -1.059751 | 2.578009 | -8.494038 | -1.109332 | 2.630732 | -8.331671 | -0.958324 | 2.922429 | -8.481308 | -1.174612 | 2.621911 | -8.506441 | -1.081793 | 2.618636 |
| H | -9.014051 | 0.526349 | 1.785385 | -9.045118 | 0.455527 | 1.774404 | -8.992315 | 0.436835 | 1.888024 | -8.903433 | 0.496344 | 2.054336 | -8.978304 | 0.400773 | 1.942165 | -9.022770 | 0.440490 | 1.836999 |
| H | -9.921663 | -1.002365 | 1.744431 | -9.984906 | -1.034296 | 1.540295 | -9.958634 | -1.032413 | 1.625212 | -9.835843 | -1.016925 | 1.973184 | -9.952380 | -1.054190 | 1.629337 | -9.961978 | -1.050089 | 1.597802 |

| **iso-I** 26 |  | gasphase |  |  | toluene |  |  | DMSO |  |  | methanol |  |  | acetonitrile |  |  | chloroform |  |
| --- | --- | --- | --- | --- | --- | --- | --- | --- | --- | --- | --- | --- | --- | --- | --- | --- | --- | --- |
| Element | x | y | z | x | y | z | x | y | z | x | y | z | x | y | z | x | y | z |
| C | -0.564209 | 2.939705 | 0.153706 | -0.568538 | 2.936431 | 0.166754 | -0.563632 | 2.932501 | 0.205349 | -0.489152 | 2.882299 | 0.339497 | -0.540249 | 2.919216 | 0.215749 | -0.565279 | 2.938763 | 0.219225 |
| C | -1.305533 | 4.099652 | 0.335913 | -1.308525 | 4.095596 | 0.359501 | -1.304214 | 4.090374 | 0.406749 | -1.206281 | 4.052933 | 0.549915 | -1.273565 | 4.083021 | 0.410589 | -1.306663 | 4.098532 | 0.405305 |
| C | -2.688186 | 4.054710 | 0.420543 | -2.692639 | 4.052067 | 0.434580 | -2.690150 | 4.045702 | 0.471676 | -2.594101 | 4.039594 | 0.585101 | -2.660140 | 4.048405 | 0.470074 | -2.692447 | 4.055944 | 0.460402 |
| C | -3.364770 | 2.844471 | 0.341135 | -3.371189 | 2.843899 | 0.338996 | -3.368980 | 2.838503 | 0.359698 | -3.300466 | 2.851659 | 0.436582 | -3.347112 | 2.845471 | 0.359365 | -3.371460 | 2.848447 | 0.353524 |
| C | -2.614789 | 1.701488 | 0.160361 | -2.621112 | 1.701896 | 0.151544 | -2.617592 | 1.698374 | 0.164373 | -2.572242 | 1.699860 | 0.230052 | -2.602984 | 1.699529 | 0.170542 | -2.619737 | 1.706399 | 0.173319 |
| C | -1.215682 | 1.716031 | 0.043745 | -1.221896 | 1.715589 | 0.038126 | -1.218285 | 1.714524 | 0.056414 | -1.172380 | 1.687093 | 0.145663 | -1.203095 | 1.705304 | 0.067704 | -1.219535 | 1.719996 | 0.075767 |
| N | -3.086441 | 0.390704 | 0.119431 | -3.094427 | 0.393353 | 0.091408 | -3.088628 | 0.389942 | 0.088384 | -3.067044 | 0.397673 | 0.128401 | -3.082955 | 0.393642 | 0.097820 | -3.091474 | 0.396838 | 0.108209 |
| C | -2.041363 | -0.503666 | 0.035532 | -2.055064 | -0.500509 | -0.011322 | -2.050622 | -0.499483 | -0.027862 | -2.045880 | -0.504095 | 0.021226 | -2.050744 | -0.502877 | -0.009148 | -2.051922 | -0.494748 | 0.018465 |
| C | -0.768653 | 0.319341 | -0.054188 | -0.778380 | 0.319386 | -0.073543 | -0.774090 | 0.321222 | -0.065169 | -0.757260 | 0.288722 | 0.012596 | -0.768768 | 0.308168 | -0.048430 | -0.775830 | 0.324663 | -0.033226 |
| C | 0.456919 | -0.229742 | -0.244406 | 0.449299 | -0.228608 | -0.249606 | 0.454795 | -0.223025 | -0.235673 | 0.458964 | -0.278771 | -0.162809 | 0.455591 | -0.246224 | -0.219507 | 0.451202 | -0.222838 | -0.210432 |
| O | -2.186710 | -1.700648 | -0.016662 | -2.202304 | -1.696366 | -0.104481 | -2.193539 | -1.694430 | -0.160286 | -2.205588 | -1.698851 | -0.138543 | -2.200991 | -1.698301 | -0.132835 | -2.195828 | -1.692536 | -0.077961 |
| C | 0.947762 | -1.612471 | -0.132784 | 0.939843 | -1.608298 | -0.112867 | 0.941382 | -1.599992 | -0.083043 | 0.919267 | -1.659656 | 0.006881 | 0.933266 | -1.626382 | -0.065906 | 0.939788 | -1.601278 | -0.068057 |
| C | 2.300072 | -1.607297 | -0.511688 | 2.299177 | -1.603276 | -0.464860 | 2.293203 | -1.607719 | -0.461022 | 2.257536 | -1.706310 | -0.409797 | 2.283468 | -1.644216 | -0.449730 | 2.290828 | -1.604692 | -0.449029 |
| N | 2.680387 | -0.329753 | -0.897470 | 2.684263 | -0.328990 | -0.857178 | 2.677225 | -0.340129 | -0.878133 | 2.651354 | -0.453310 | -0.874588 | 2.673946 | -0.379973 | -0.872791 | 2.671891 | -0.334358 | -0.861596 |
| C | 1.658900 | 0.568313 | -0.715178 | 1.654863 | 0.564177 | -0.719331 | 1.658796 | 0.560144 | -0.724827 | 1.661948 | 0.468936 | -0.694315 | 1.663099 | 0.527350 | -0.713479 | 1.651704 | 0.563611 | -0.701542 |
| C | 0.409322 | -2.804939 | 0.336019 | 0.396689 | -2.794070 | 0.367360 | 0.402615 | -2.766916 | 0.446513 | 0.372215 | -2.792468 | 0.597511 | 0.388809 | -2.788392 | 0.469045 | 0.403700 | -2.776074 | 0.446345 |
| C | 1.201996 | -3.945085 | 0.386555 | 1.191602 | -3.930579 | 0.451810 | 1.192790 | -3.905763 | 0.543257 | 1.140769 | -3.944407 | 0.706397 | 1.171921 | -3.932360 | 0.565351 | 1.196532 | -3.914256 | 0.529775 |
| C | 2.526853 | -3.911792 | -0.016575 | 2.524260 | -3.898689 | 0.071975 | 2.516875 | -3.891012 | 0.127190 | 2.450905 | -3.971436 | 0.248074 | 2.494621 | -3.927307 | 0.144249 | 2.520214 | -3.892788 | 0.116069 |
| C | 3.099130 | -2.729003 | -0.467714 | 3.101772 | -2.721289 | -0.387019 | 3.091776 | -2.729492 | -0.374818 | 3.035894 | -2.839659 | -0.308403 | 3.075153 | -2.770688 | -0.363141 | 3.091652 | -2.724453 | -0.373773 |
| O | 1.758821 | 1.745143 | -0.973467 | 1.743629 | 1.734886 | -1.013633 | 1.745992 | 1.732074 | -1.022043 | 1.762176 | 1.639454 | -1.008977 | 1.757740 | 1.699439 | -1.009007 | 1.737832 | 1.736097 | -0.995186 |
| C | -4.451629 | 0.008082 | 0.149566 | -4.463363 | 0.016207 | 0.133893 | -4.458526 | 0.012976 | 0.124633 | -4.442966 | 0.040632 | 0.114394 | -4.455836 | 0.025409 | 0.125950 | -4.461193 | 0.017895 | 0.131388 |
| C | -4.932728 | -0.797826 | 1.175311 | -4.976460 | -0.624926 | 1.255321 | -4.979222 | -0.606795 | 1.254585 | -4.982253 | -0.686432 | 1.169073 | -4.990874 | -0.579046 | 1.257716 | -4.994175 | -0.601936 | 1.255574 |
| C | -6.258917 | -1.169713 | 1.200014 | -6.305251 | -0.987851 | 1.297418 | -6.309323 | -0.969160 | 1.292210 | -6.316241 | -1.036303 | 1.153488 | -6.323705 | -0.932850 | 1.286686 | -6.323504 | -0.966875 | 1.278392 |
| C | -7.131602 | -0.731548 | 0.204573 | -7.145731 | -0.704389 | 0.220294 | -7.141632 | -0.705036 | 0.202349 | -7.130020 | -0.651248 | 0.087442 | -7.143570 | -0.675300 | 0.185805 | -7.142503 | -0.706301 | 0.178678 |
| C | -6.654140 | 0.077188 | -0.820668 | -6.634786 | -0.057220 | -0.900534 | -6.621272 | -0.077131 | -0.926608 | -6.592251 | 0.083203 | -0.965063 | -6.608867 | -0.063652 | -0.945301 | -6.610977 | -0.080088 | -0.944978 |
| C | -5.312696 | 0.432884 | -0.845120 | -5.292249 | 0.292562 | -0.937491 | -5.278514 | 0.273012 | -0.958340 | -5.245834 | 0.418714 | -0.946649 | -5.263669 | 0.277908 | -0.968110 | -5.268694 | 0.272373 | -0.961973 |
| O | -8.414102 | -1.142299 | 0.319936 | -8.430589 | -1.092351 | 0.355314 | -8.426527 | -1.089413 | 0.331900 | -8.424501 | -1.034402 | 0.163153 | -8.432385 | -1.050252 | 0.307546 | -8.429342 | -1.094208 | 0.295426 |
| C | -9.336117 | -0.742797 | -0.666224 | -9.322448 | -0.845751 | -0.710439 | -9.311266 | -0.846438 | -0.748104 | -9.297366 | -0.679515 | -0.897497 | -9.308947 | -0.811815 | -0.780225 | -9.302935 | -0.868925 | -0.793991 |
| C | 3.991377 | 0.061545 | -1.301402 | 3.995016 | 0.043318 | -1.284471 | 3.991617 | 0.017747 | -1.313968 | 3.960987 | -0.121348 | -1.346678 | 3.988204 | -0.029151 | -1.315651 | 3.981672 | 0.028601 | -1.304061 |
| C | 4.957829 | 0.170723 | -0.127846 | 4.971484 | 0.186645 | -0.123715 | 4.961846 | 0.186630 | -0.152497 | 4.937800 | 0.132986 | -0.210739 | 4.959146 | 0.161193 | -0.158664 | 4.965204 | 0.178937 | -0.151518 |
| O | 6.193923 | 0.315048 | -0.593308 | 6.204935 | 0.273580 | -0.600496 | 6.195280 | 0.262122 | -0.619800 | 6.151223 | 0.288074 | -0.695632 | 6.186905 | 0.275059 | -0.631692 | 6.194460 | 0.260953 | -0.632807 |
| O | 4.621526 | 0.140897 | 1.021821 | 4.639303 | 0.228840 | 1.028749 | 4.619034 | 0.256147 | 0.998855 | 4.611064 | 0.188796 | 0.950166 | 4.621677 | 0.211976 | 0.995543 | 4.637918 | 0.229108 | 1.004054 |
| C | 7.342296 | 0.490437 | 0.296775 | 7.371162 | 0.467232 | 0.268273 | 7.362989 | 0.465273 | 0.250511 | 7.328527 | 0.572421 | 0.146936 | 7.354364 | 0.507878 | 0.231785 | 7.369277 | 0.463370 | 0.226176 |
| C | 7.511078 | -0.743141 | 1.173857 | 7.519021 | -0.722326 | 1.205646 | 7.500831 | -0.706855 | 1.209536 | 7.568540 | -0.588681 | 1.098183 | 7.531776 | -0.663243 | 1.185889 | 7.524719 | -0.717348 | 1.172825 |
| C | 7.176684 | 1.762980 | 1.116592 | 7.247328 | 1.784870 | 1.018970 | 7.245964 | 1.797993 | 0.973161 | 7.138232 | 1.896666 | 0.868231 | 7.203032 | 1.834821 | 0.959143 | 7.249469 | 1.788825 | 0.962737 |
| C | 8.506252 | 0.623889 | -0.673266 | 8.524672 | 0.518875 | -0.720466 | 8.520106 | 0.490076 | -0.733604 | 8.448958 | 0.671543 | -0.873960 | 8.502774 | 0.568449 | -0.761368 | 8.513585 | 0.504991 | -0.773257 |
| H | 0.508064 | 2.988893 | 0.083229 | 0.504631 | 2.985578 | 0.103871 | 0.510723 | 2.981013 | 0.154301 | 0.588038 | 2.904981 | 0.326770 | 0.534718 | 2.960075 | 0.169812 | 0.509344 | 2.986505 | 0.176043 |
| H | -0.791344 | 5.048568 | 0.411418 | -0.793229 | 5.042936 | 0.449871 | -0.790248 | 5.037119 | 0.512986 | -0.673049 | 4.984509 | 0.690040 | -0.753290 | 5.026362 | 0.516185 | -0.792058 | 5.045235 | 0.507270 |
| H | -3.251098 | 4.969006 | 0.560680 | -3.254583 | 4.966336 | 0.580082 | -3.253051 | 4.958561 | 0.622884 | -3.137934 | 4.962420 | 0.745588 | -3.217133 | 4.965676 | 0.616250 | -3.255377 | 4.970544 | 0.600404 |
| H | -4.440936 | 2.793067 | 0.433028 | -4.448573 | 2.794051 | 0.421006 | -4.447322 | 2.790728 | 0.433170 | -4.380592 | 2.829412 | 0.494005 | -4.425980 | 2.805158 | 0.429069 | -4.450053 | 2.800427 | 0.421563 |
| H | -0.621042 | -2.849308 | 0.641628 | -0.638644 | -2.837120 | 0.657533 | -0.623648 | -2.794246 | 0.771251 | -0.638700 | -2.783048 | 0.970665 | -0.636177 | -2.807631 | 0.798654 | -0.623188 | -2.809736 | 0.767679 |
| H | 4.141420 | -2.689152 | -0.755371 | 4.149304 | -2.681265 | -0.656052 | 4.132308 | -2.704642 | -0.671750 | 4.068884 | -2.845884 | -0.631007 | 4.114898 | -2.751967 | -0.663037 | 4.132576 | -2.692172 | -0.668468 |
| H | 3.919958 | 1.044957 | -1.768649 | 3.928321 | 1.010660 | -1.784708 | 3.933991 | 0.969329 | -1.843871 | 3.901856 | 0.785266 | -1.950023 | 3.931580 | 0.911213 | -1.865046 | 3.919427 | 0.989673 | -1.816444 |
| H | 4.393513 | -0.631283 | -2.042106 | 4.383432 | -0.678022 | -2.004471 | 4.376372 | -0.728027 | -2.009455 | 4.347661 | -0.915545 | -1.985041 | 4.374350 | -0.788222 | -1.995832 | 4.360962 | -0.704272 | -2.016700 |
| H | 0.771976 | -4.870552 | 0.745853 | 0.758454 | -4.851778 | 0.819115 | 0.766409 | -4.814000 | 0.949574 | 0.710702 | -4.828674 | 1.159032 | 0.741220 | -4.836678 | 0.975778 | 0.770456 | -4.827822 | 0.923866 |
| H | 3.128834 | -4.810549 | 0.028711 | 3.128110 | -4.794784 | 0.142587 | 3.117262 | -4.788991 | 0.204996 | 3.034961 | -4.878933 | 0.338968 | 3.089529 | -4.828864 | 0.222535 | 3.122512 | -4.790123 | 0.185432 |
| H | -4.256607 | -1.139690 | 1.947084 | -4.325228 | -0.841381 | 2.092295 | -4.336041 | -0.803966 | 2.103075 | -4.352090 | -0.976967 | 2.000379 | -4.356681 | -0.771210 | 2.114081 | -4.359411 | -0.799811 | 2.109960 |
| H | -4.934882 | 1.050272 | -1.650322 | -4.888031 | 0.786528 | -1.812320 | -4.869003 | 0.756293 | -1.836917 | -4.821023 | 0.982748 | -1.767947 | -4.842593 | 0.748709 | -1.847950 | -4.848634 | 0.751695 | -1.837600 |
| H | -6.645613 | -1.801666 | 1.988195 | -6.716193 | -1.492760 | 2.161821 | -6.724762 | -1.455356 | 2.165697 | -6.748416 | -1.605697 | 1.966761 | -6.750981 | -1.407166 | 2.161094 | -6.750294 | -1.454944 | 2.145149 |
| H | -7.305292 | 0.426098 | -1.608765 | -7.262564 | 0.170636 | -1.749803 | -7.243201 | 0.139060 | -1.783387 | -7.201428 | 0.392107 | -1.802302 | -7.221956 | 0.146090 | -1.809998 | -7.223270 | 0.130756 | -1.809841 |
| H | -9.438019 | 0.346421 | -0.702019 | -9.421835 | 0.225363 | -0.911553 | -9.398873 | 0.223039 | -0.958509 | -9.374488 | 0.406033 | -0.999716 | -9.386936 | 0.256185 | -1.001002 | -9.397784 | 0.198058 | -1.015471 |
| H | -10.290279 | -1.181614 | -0.384365 | -10.286732 | -1.238377 | -0.394283 | -10.280024 | -1.230585 | -0.435738 | -10.270752 | -1.086530 | -0.632014 | -10.281944 | -1.186197 | -0.469349 | -10.271912 | -1.257453 | -0.487483 |
| H | -9.048905 | -1.111497 | -1.655827 | -9.008960 | -1.361283 | -1.623254 | -8.989878 | -1.371444 | -1.651865 | -8.967612 | -1.116845 | -1.843429 | -8.985456 | -1.348032 | -1.676500 | -8.969268 | -1.401403 | -1.689210 |
| H | 6.689915 | -0.844529 | 1.879579 | 6.711374 | -0.762949 | 1.933380 | 6.696372 | -0.725068 | 1.942139 | 6.796777 | -0.657324 | 1.862680 | 6.734320 | -0.704850 | 1.925170 | 6.727326 | -0.748427 | 1.912497 |
| H | 7.565012 | -1.642058 | 0.557299 | 7.533802 | -1.655385 | 0.638963 | 7.504402 | -1.650652 | 0.660681 | 7.608666 | -1.530166 | 0.547058 | 7.555603 | -1.604517 | 0.633287 | 7.529262 | -1.655545 | 0.614433 |
| H | 8.444328 | -0.659115 | 1.732916 | 8.465194 | -0.638275 | 1.743029 | 8.450394 | -0.618398 | 1.740140 | 8.529692 | -0.442710 | 1.594082 | 8.483249 | -0.550745 | 1.708382 | 8.478411 | -0.629439 | 1.696111 |
| H | 6.359031 | 1.675234 | 1.828126 | 6.444602 | 1.756399 | 1.752640 | 6.441449 | 1.790335 | 1.705915 | 6.366814 | 1.837541 | 1.633805 | 6.402630 | 1.801353 | 1.695772 | 6.452413 | 1.767836 | 1.703180 |
| H | 6.988351 | 2.614541 | 0.460908 | 7.062676 | 2.605165 | 0.322894 | 7.070841 | 2.605511 | 0.259594 | 6.876068 | 2.683212 | 0.158150 | 7.000858 | 2.638321 | 0.248269 | 7.059330 | 2.600718 | 0.258201 |
| H | 8.099115 | 1.955151 | 1.666845 | 8.184575 | 1.987619 | 1.540138 | 8.184308 | 2.000860 | 1.492213 | 8.078403 | 2.171332 | 1.349562 | 8.137811 | 2.062755 | 1.474138 | 8.190831 | 1.996502 | 1.474435 |
| H | 9.435685 | 0.759802 | -0.119244 | 9.464682 | 0.659977 | -0.185491 | 9.456103 | 0.636528 | -0.193132 | 9.389865 | 0.881478 | -0.363995 | 9.438256 | 0.740631 | -0.227675 | 9.457350 | 0.652144 | -0.246607 |
| H | 8.597338 | -0.271201 | -1.289601 | 8.587729 | -0.409797 | -1.289788 | 8.581199 | -0.451305 | -1.282291 | 8.554326 | -0.265635 | -1.423319 | 8.585979 | -0.369720 | -1.312638 | 8.571853 | -0.430320 | -1.332417 |
| H | 8.363450 | 1.483906 | -1.328338 | 8.397696 | 1.346860 | -1.419519 | 8.402226 | 1.306201 | -1.448475 | 8.253128 | 1.476246 | -1.584781 | 8.355011 | 1.382510 | -1.472990 | 8.379799 | 1.326217 | -1.479240 |

| **iso-I** 27 |  | gasphase |  |  | toluene |  |  | DMSO |  |  | methanol |  |  | acetonitrile |  |  | chloroform |  |
| --- | --- | --- | --- | --- | --- | --- | --- | --- | --- | --- | --- | --- | --- | --- | --- | --- | --- | --- |
| Element | x | y | z | x | y | z | x | y | z | x | y | z | x | y | z | x | y | z |
| C | -0.119078 | -2.683521 | -0.689763 | -0.102838 | -2.677734 | -0.658253 | -0.087121 | -2.677808 | -0.705126 | -0.051965 | -2.671960 | -1.042814 | -0.067501 | -2.685372 | -0.808457 | -0.085897 | -2.681119 | -0.728108 |
| C | 0.513548 | -3.897337 | -0.917133 | 0.537508 | -3.888076 | -0.883604 | 0.558863 | -3.881299 | -0.952717 | 0.608025 | -3.870421 | -1.276879 | 0.585437 | -3.890805 | -1.028083 | 0.562429 | -3.887689 | -0.953064 |
| C | 1.893508 | -4.006784 | -0.833611 | 1.918192 | -3.990416 | -0.790796 | 1.940825 | -3.979673 | -0.854312 | 1.979347 | -3.975830 | -1.083224 | 1.963464 | -3.989419 | -0.883765 | 1.942071 | -3.986652 | -0.835025 |
| C | 2.676099 | -2.898785 | -0.539717 | 2.693978 | -2.878143 | -0.493754 | 2.712123 | -2.870686 | -0.531240 | 2.730319 | -2.878721 | -0.678508 | 2.725214 | -2.878443 | -0.543906 | 2.709613 | -2.874602 | -0.514118 |
| C | 2.032046 | -1.698734 | -0.318920 | 2.041649 | -1.681596 | -0.278785 | 2.053786 | -1.681535 | -0.293874 | 2.058446 | -1.696325 | -0.453259 | 2.060395 | -1.687507 | -0.336004 | 2.049408 | -1.682428 | -0.299944 |
| C | 0.636189 | -1.561143 | -0.366474 | 0.645194 | -1.552241 | -0.328710 | 0.657034 | -1.559073 | -0.345091 | 0.669055 | -1.570479 | -0.594673 | 0.665765 | -1.565308 | -0.430189 | 0.653553 | -1.557713 | -0.372347 |
| N | 2.629301 | -0.462334 | -0.081438 | 2.630628 | -0.442921 | -0.036532 | 2.634288 | -0.444080 | -0.024392 | 2.617015 | -0.460676 | -0.118671 | 2.632329 | -0.447011 | -0.060918 | 2.628921 | -0.442760 | -0.037348 |
| C | 1.681326 | 0.536673 | -0.025372 | 1.679445 | 0.547153 | 0.033274 | 1.677932 | 0.534368 | 0.074749 | 1.657256 | 0.511644 | -0.075188 | 1.674571 | 0.533339 | -0.008259 | 1.673808 | 0.540557 | 0.026757 |
| C | 0.330155 | -0.135968 | -0.180384 | 0.331708 | -0.130074 | -0.137347 | 0.336814 | -0.144207 | -0.131052 | 0.334560 | -0.164881 | -0.366157 | 0.338360 | -0.150330 | -0.230396 | 0.332470 | -0.139350 | -0.174737 |
| C | -0.850706 | 0.528898 | -0.107239 | -0.853269 | 0.527233 | -0.072302 | -0.850782 | 0.505734 | -0.075462 | -0.856192 | 0.478790 | -0.315478 | -0.852010 | 0.495135 | -0.174503 | -0.854636 | 0.513734 | -0.118712 |
| O | 1.939820 | 1.697324 | 0.181843 | 1.927262 | 1.706633 | 0.267028 | 1.909916 | 1.686198 | 0.367280 | 1.866353 | 1.664705 | 0.246991 | 1.901369 | 1.692031 | 0.261084 | 1.909864 | 1.699536 | 0.283084 |
| C | -1.199387 | 1.948623 | -0.204280 | -1.207677 | 1.944428 | -0.186087 | -1.201930 | 1.920393 | -0.206659 | -1.198674 | 1.894415 | -0.411344 | -1.205022 | 1.910711 | -0.285777 | -1.206503 | 1.930543 | -0.231092 |
| C | -2.593345 | 2.065532 | -0.087353 | -2.602809 | 2.054297 | -0.079889 | -2.596006 | 2.032857 | -0.100113 | -2.577338 | 2.024819 | -0.202535 | -2.593644 | 2.025754 | -0.128439 | -2.598837 | 2.044827 | -0.102644 |
| N | -3.167319 | 0.792462 | 0.096668 | -3.169023 | 0.781297 | 0.116368 | -3.161809 | 0.763912 | 0.117190 | -3.142365 | 0.753862 | 0.036434 | -3.155017 | 0.755205 | 0.093140 | -3.165052 | 0.772552 | 0.102330 |
| C | -2.158183 | -0.182649 | 0.151906 | -2.158694 | -0.186366 | 0.187830 | -2.155558 | -0.204751 | 0.189324 | -2.151569 | -0.231223 | -0.030992 | -2.152200 | -0.219830 | 0.102003 | -2.158546 | -0.199651 | 0.142181 |
| C | -0.461010 | 3.096415 | -0.479764 | -0.475196 | 3.091524 | -0.478900 | -0.467881 | 3.055975 | -0.537072 | -0.472565 | 3.012899 | -0.808462 | -0.480314 | 3.045962 | -0.637873 | -0.474992 | 3.071031 | -0.550917 |
| C | -1.108973 | 4.314489 | -0.605104 | -1.129085 | 4.303831 | -0.629008 | -1.119241 | 4.266551 | -0.713501 | -1.115099 | 4.233988 | -0.932054 | -1.134310 | 4.259631 | -0.781149 | -1.126586 | 4.285287 | -0.696926 |
| C | -2.484835 | 4.402396 | -0.462963 | -2.506944 | 4.385464 | -0.497412 | -2.497617 | 4.353807 | -0.576757 | -2.476556 | 4.343153 | -0.683450 | -2.506384 | 4.349897 | -0.590853 | -2.501767 | 4.373333 | -0.537306 |
| C | -3.250361 | 3.273820 | -0.205790 | -3.266419 | 3.255999 | -0.226161 | -3.258214 | 3.231837 | -0.276558 | -3.232369 | 3.233595 | -0.328227 | -3.258903 | 3.227631 | -0.271196 | -3.260704 | 3.248227 | -0.245356 |
| O | -2.325696 | -1.326371 | 0.470644 | -2.323688 | -1.326448 | 0.525515 | -2.319767 | -1.343062 | 0.539193 | -2.314396 | -1.396343 | 0.229790 | -2.313281 | -1.370254 | 0.411870 | -2.322926 | -1.347083 | 0.458510 |
| C | 4.017450 | -0.247751 | 0.114185 | 4.022964 | -0.223088 | 0.140746 | 4.025851 | -0.219793 | 0.158251 | 3.990118 | -0.232947 | 0.170839 | 4.017872 | -0.217624 | 0.160479 | 4.018669 | -0.216602 | 0.158104 |
| C | 4.677884 | -0.886054 | 1.159011 | 4.662218 | -0.708356 | 1.276352 | 4.661089 | -0.695130 | 1.300069 | 4.561115 | -0.795772 | 1.306640 | 4.614365 | -0.660389 | 1.335909 | 4.638762 | -0.669202 | 1.317440 |
| C | 6.027785 | -0.688715 | 1.352076 | 6.015062 | -0.509182 | 1.448033 | 6.013330 | -0.488377 | 1.474778 | 5.893916 | -0.579692 | 1.586786 | 5.959760 | -0.446094 | 1.550612 | 5.989106 | -0.463492 | 1.504303 |
| C | 6.737790 | 0.169470 | 0.512958 | 6.748280 | 0.193095 | 0.490615 | 6.748537 | 0.207961 | 0.512931 | 6.668773 | 0.214186 | 0.740130 | 6.726054 | 0.225263 | 0.595279 | 6.737139 | 0.210380 | 0.537352 |
| C | 6.076590 | 0.819160 | -0.523398 | 6.107858 | 0.685171 | -0.642422 | 6.110683 | 0.687255 | -0.628579 | 6.096153 | 0.782040 | -0.393746 | 6.127455 | 0.671172 | -0.580526 | 6.115313 | 0.669361 | -0.620183 |
| C | 4.720605 | 0.600087 | -0.720134 | 4.747963 | 0.467057 | -0.811994 | 4.751702 | 0.463014 | -0.800216 | 4.757404 | 0.547531 | -0.674392 | 4.775013 | 0.440069 | -0.791561 | 4.757886 | 0.446143 | -0.803768 |
| O | 8.054138 | 0.306539 | 0.785468 | 8.063384 | 0.344366 | 0.748424 | 8.061119 | 0.366583 | 0.772125 | 7.961811 | 0.375573 | 1.100740 | 8.028771 | 0.394684 | 0.896230 | 8.048498 | 0.368903 | 0.810495 |
| C | 8.818312 | 1.172559 | -0.020518 | 8.851266 | 1.065835 | -0.174467 | 8.850108 | 1.077591 | -0.166152 | 8.796600 | 1.178961 | 0.281786 | 8.851953 | 1.079022 | -0.032569 | 8.855874 | 1.050853 | -0.129821 |
| C | -4.539772 | 0.567653 | 0.299711 | -4.541355 | 0.550951 | 0.328396 | -4.532267 | 0.544268 | 0.366763 | -4.477301 | 0.553064 | 0.430653 | -4.512970 | 0.539878 | 0.406784 | -4.532398 | 0.548825 | 0.352877 |
| O | -4.858732 | -0.656642 | -0.056585 | -4.870162 | -0.647991 | -0.090981 | -4.891402 | -0.633938 | -0.076947 | -4.887568 | -0.641795 | 0.097703 | -4.903303 | -0.628843 | -0.034355 | -4.881227 | -0.645649 | -0.057126 |
| O | -5.277276 | 1.423012 | 0.710859 | -5.264282 | 1.389330 | 0.797333 | -5.222637 | 1.380553 | 0.887858 | -5.109594 | 1.421558 | 0.978792 | -5.169867 | 1.372569 | 0.975201 | -5.234609 | 1.391937 | 0.846313 |
| C | -6.171108 | -1.230607 | 0.240984 | -6.190289 | -1.232458 | 0.175584 | -6.224711 | -1.200302 | 0.195105 | -6.200517 | -1.178246 | 0.517253 | -6.228250 | -1.187900 | 0.289264 | -6.204819 | -1.218892 | 0.231954 |
| C | -6.425287 | -1.188031 | 1.741277 | -6.439507 | -1.279873 | 1.675159 | -6.450858 | -1.287753 | 1.695416 | -6.287211 | -1.189173 | 2.033862 | -6.387696 | -1.294825 | 1.797061 | -6.427449 | -1.267514 | 1.735224 |
| C | -6.015064 | -2.665787 | -0.237737 | -6.047669 | -2.635626 | -0.390932 | -6.125838 | -2.587410 | -0.415231 | -6.157297 | -2.593343 | -0.031971 | -6.166557 | -2.566731 | -0.345027 | -6.084392 | -2.621473 | -0.340340 |
| C | -7.249822 | -0.506337 | -0.552000 | -7.262863 | -0.453866 | -0.570582 | -7.288271 | -0.375128 | -0.510184 | -7.312620 | -0.376043 | -0.136236 | -7.314237 | -0.343624 | -0.357363 | -7.280098 | -0.427219 | -0.495321 |
| H | -1.191658 | -2.622073 | -0.748306 | -1.175591 | -2.622127 | -0.726179 | -1.159764 | -2.622145 | -0.785283 | -1.114602 | -2.603984 | -1.210321 | -1.136709 | -2.628113 | -0.925492 | -1.157325 | -2.626170 | -0.820046 |
| H | -0.082544 | -4.767573 | -1.157590 | -0.052342 | -4.761482 | -1.129206 | -0.025215 | -4.751682 | -1.222738 | 0.044246 | -4.730478 | -1.614677 | 0.009976 | -4.762301 | -1.312565 | -0.019585 | -4.760468 | -1.219314 |
| H | 2.371051 | -4.962665 | -1.008246 | 2.401428 | -4.944491 | -0.960885 | 2.428758 | -4.928044 | -1.042679 | 2.477619 | -4.920315 | -1.264181 | 2.456400 | -4.939295 | -1.050194 | 2.431338 | -4.937727 | -1.005196 |
| H | 3.754567 | -2.965508 | -0.499357 | 3.772708 | -2.939906 | -0.444029 | 3.790993 | -2.932083 | -0.478722 | 3.803269 | -2.946455 | -0.557654 | 3.801872 | -2.939513 | -0.457076 | 3.787579 | -2.934452 | -0.445830 |
| H | 0.608994 | 3.036328 | -0.572363 | 0.595653 | 3.036576 | -0.569362 | 0.601851 | 2.995660 | -0.643446 | 0.580706 | 2.932173 | -1.020787 | 0.584003 | 2.983054 | -0.788096 | 0.593195 | 3.011447 | -0.669402 |
| H | -4.320242 | 3.334570 | -0.090587 | -4.338351 | 3.314537 | -0.125147 | -4.331106 | 3.297587 | -0.186678 | -4.295742 | 3.318589 | -0.168392 | -4.327967 | 3.294923 | -0.143118 | -4.331195 | 3.313110 | -0.131672 |
| H | -0.528363 | 5.204666 | -0.808270 | -0.552896 | 5.194042 | -0.845419 | -0.543953 | 5.149684 | -0.960196 | -0.548882 | 5.106966 | -1.230445 | -0.566417 | 5.142624 | -1.044824 | -0.552079 | 5.171357 | -0.934319 |
| H | -2.977546 | 5.362252 | -0.553248 | -3.005375 | 5.340237 | -0.609215 | -2.995214 | 5.305848 | -0.713675 | -2.967791 | 5.303124 | -0.782922 | -3.005795 | 5.304248 | -0.702623 | -2.998753 | 5.329157 | -0.647107 |
| H | 4.124226 | -1.539559 | 1.821311 | 4.090864 | -1.242532 | 2.025233 | 4.090114 | -1.229284 | 2.049550 | 3.957191 | -1.404188 | 1.968495 | 4.018551 | -1.174543 | 2.079988 | 4.056034 | -1.183685 | 2.071457 |
| H | 4.204672 | 1.106123 | -1.524758 | 4.248023 | 0.844738 | -1.694605 | 4.254948 | 0.826587 | -1.691112 | 4.309391 | 0.980570 | -1.560042 | 4.307386 | 0.779030 | -1.707559 | 4.271317 | 0.796650 | -1.705254 |
| H | 6.553800 | -1.180218 | 2.159482 | 6.524469 | -0.881368 | 2.327249 | 6.518158 | -0.855835 | 2.359140 | 6.351754 | -1.013820 | 2.466703 | 6.435567 | -0.787390 | 2.461187 | 6.484470 | -0.811107 | 2.401754 |
| H | 6.600171 | 1.493523 | -1.185088 | 6.650886 | 1.233411 | -1.398557 | 6.655099 | 1.227376 | -1.389733 | 6.675095 | 1.398855 | -1.065926 | 6.697399 | 1.191154 | -1.337010 | 6.670803 | 1.194680 | -1.383590 |
| H | 9.831256 | 1.137299 | 0.373255 | 9.860378 | 1.075624 | 0.232443 | 9.859408 | 1.093564 | 0.239735 | 9.775305 | 1.173762 | 0.756800 | 9.845429 | 1.106662 | 0.409843 | 9.859512 | 1.065204 | 0.289935 |
| H | 8.446930 | 2.200550 | 0.032789 | 8.499245 | 2.096161 | -0.282511 | 8.494498 | 2.104403 | -0.288096 | 8.425802 | 2.205765 | 0.226969 | 8.500776 | 2.101431 | -0.196428 | 8.513353 | 2.078958 | -0.277947 |
| H | 8.826740 | 0.844731 | -1.064674 | 8.866738 | 0.580295 | -1.154956 | 8.861147 | 0.577004 | -1.138169 | 8.880642 | 0.762810 | -0.725441 | 8.897089 | 0.550189 | -0.988446 | 8.875889 | 0.528807 | -1.090801 |
| H | -5.597400 | -1.655275 | 2.276457 | -5.608337 | -1.772224 | 2.182844 | -5.623017 | -1.811874 | 2.176527 | -5.418242 | -1.693009 | 2.460913 | -5.544249 | -1.834102 | 2.232063 | -5.591573 | -1.768055 | 2.227153 |
| H | -6.550688 | -0.167551 | 2.096946 | -6.573615 | -0.284915 | 2.095087 | -6.561481 | -0.303966 | 2.147721 | -6.356976 | -0.185105 | 2.448526 | -6.467037 | -0.316250 | 2.266974 | -6.547258 | -0.272832 | 2.160376 |
| H | -7.335616 | -1.746723 | 1.963274 | -7.345563 | -1.857183 | 1.866133 | -7.365453 | -1.853904 | 1.878371 | -7.181239 | -1.742081 | 2.326425 | -7.299063 | -1.854560 | 2.012868 | -7.334689 | -1.838645 | 1.938691 |
| H | -5.796260 | -2.691072 | -1.305726 | -5.820303 | -2.599441 | -1.457335 | -5.912071 | -2.525348 | -1.483451 | -6.041747 | -2.582264 | -1.117044 | -6.000210 | -2.489116 | -1.420655 | -5.874730 | -2.584489 | -1.410475 |
| H | -6.938617 | -3.217324 | -0.058244 | -6.980632 | -3.184028 | -0.254854 | -7.073602 | -3.110278 | -0.282132 | -7.087966 | -3.106461 | 0.212392 | -7.110810 | -3.085851 | -0.177055 | -7.020713 | -3.160094 | -0.189600 |
| H | -5.200143 | -3.156235 | 0.294569 | -5.247718 | -3.175457 | 0.116683 | -5.338216 | -3.166998 | 0.068210 | -5.327237 | -3.150574 | 0.405234 | -5.361374 | -3.157224 | 0.094399 | -5.282151 | -3.170579 | 0.154114 |
| H | -7.374640 | 0.517821 | -0.208239 | -7.390406 | 0.546269 | -0.162145 | -7.385078 | 0.616019 | -0.071557 | -7.379348 | 0.632479 | 0.267596 | -7.384840 | 0.641056 | 0.100491 | -7.392108 | 0.573152 | -0.082290 |
| H | -8.196581 | -1.035256 | -0.430933 | -8.211875 | -0.986300 | -0.486468 | -8.246846 | -0.888931 | -0.420664 | -8.261586 | -0.883034 | 0.046383 | -8.272115 | -0.852033 | -0.235620 | -8.232315 | -0.951220 | -0.396315 |
| H | -6.998141 | -0.498063 | -1.613621 | -7.010773 | -0.375859 | -1.629722 | -7.053431 | -0.274967 | -1.571524 | -7.157937 | -0.319370 | -1.215224 | -7.122653 | -0.228513 | -1.425805 | -7.043758 | -0.349724 | -1.558186 |

| **iso-I** 28 |  | gasphase |  |  | toluene |  |  | DMSO |  |  | methanol |  |  | acetonitrile |  |  | chloroform |  |
| --- | --- | --- | --- | --- | --- | --- | --- | --- | --- | --- | --- | --- | --- | --- | --- | --- | --- | --- |
| Element | x | y | z | x | y | z | x | y | z | x | y | z | x | y | z | x | y | z |
| C | 0.862099 | -2.857235 | -0.936479 | 0.866486 | -2.845237 | -0.908000 | 0.863712 | -2.819056 | -0.907374 | 0.875888 | -2.803971 | -1.128941 | 0.866683 | -2.827022 | -0.920055 | 0.858622 | -2.835766 | -0.917668 |
| C | 1.565257 | -4.047705 | -1.060754 | 1.573240 | -4.031473 | -1.051714 | 1.569746 | -4.003549 | -1.072239 | 1.581388 | -3.989644 | -1.285475 | 1.574317 | -4.011309 | -1.080987 | 1.562176 | -4.023014 | -1.071190 |
| C | 2.931279 | -4.094331 | -0.830156 | 2.941042 | -4.075942 | -0.826513 | 2.938732 | -4.052736 | -0.845578 | 2.937838 | -4.052872 | -0.995053 | 2.942828 | -4.058799 | -0.850680 | 2.930492 | -4.072906 | -0.845783 |
| C | 3.632479 | -2.946557 | -0.484639 | 3.640154 | -2.929805 | -0.470847 | 3.639758 | -2.912732 | -0.472445 | 3.630035 | -2.926089 | -0.567025 | 3.641783 | -2.917431 | -0.476981 | 3.633469 | -2.931099 | -0.482410 |
| C | 2.920198 | -1.771939 | -0.365330 | 2.923874 | -1.759078 | -0.335594 | 2.923313 | -1.744004 | -0.318389 | 2.913702 | -1.757755 | -0.419104 | 2.923834 | -1.749228 | -0.326215 | 2.919978 | -1.759693 | -0.338009 |
| C | 1.532576 | -1.695827 | -0.566847 | 1.534614 | -1.686636 | -0.525779 | 1.533844 | -1.670040 | -0.501001 | 1.535089 | -1.673087 | -0.660520 | 1.534747 | -1.676581 | -0.513751 | 1.530735 | -1.683107 | -0.524427 |
| N | 3.437093 | -0.507277 | -0.090672 | 3.436276 | -0.496252 | -0.046923 | 3.434552 | -0.484401 | -0.015670 | 3.414861 | -0.499916 | -0.076130 | 3.432899 | -0.488426 | -0.022304 | 3.434923 | -0.498396 | -0.044195 |
| C | 2.448862 | 0.449855 | -0.155491 | 2.444019 | 0.454310 | -0.080109 | 2.442598 | 0.463248 | -0.025493 | 2.429759 | 0.446565 | -0.124138 | 2.439876 | 0.457245 | -0.036210 | 2.445099 | 0.451964 | -0.068703 |
| C | 1.148074 | -0.284274 | -0.437922 | 1.146376 | -0.278487 | -0.378117 | 1.146776 | -0.265260 | -0.333727 | 1.147902 | -0.271904 | -0.487183 | 1.146109 | -0.271708 | -0.349612 | 1.146622 | -0.275241 | -0.368226 |
| C | -0.057304 | 0.337739 | -0.473894 | -0.060635 | 0.339166 | -0.417634 | -0.061889 | 0.345871 | -0.368222 | -0.059529 | 0.338625 | -0.530348 | -0.061864 | 0.340804 | -0.388179 | -0.059439 | 0.343091 | -0.401934 |
| O | 2.634980 | 1.624461 | 0.048286 | 2.620087 | 1.624279 | 0.164399 | 2.613256 | 1.628595 | 0.254351 | 2.589266 | 1.613339 | 0.177198 | 2.607648 | 1.623234 | 0.244335 | 2.621712 | 1.621074 | 0.188300 |
| C | -0.430938 | 1.756316 | -0.563407 | -0.434842 | 1.755441 | -0.524844 | -0.438795 | 1.757189 | -0.498719 | -0.431491 | 1.748755 | -0.657882 | -0.436451 | 1.753192 | -0.516516 | -0.432812 | 1.758019 | -0.518908 |
| C | -1.829260 | 1.832968 | -0.470950 | -1.834124 | 1.830392 | -0.448515 | -1.838479 | 1.828165 | -0.432768 | -1.826544 | 1.828194 | -0.547685 | -1.835737 | 1.826864 | -0.444849 | -1.832100 | 1.833157 | -0.444470 |
| N | -2.367524 | 0.554460 | -0.302887 | -2.370930 | 0.552796 | -0.271638 | -2.370826 | 0.551827 | -0.237890 | -2.357872 | 0.550842 | -0.334686 | -2.369870 | 0.550661 | -0.250693 | -2.368169 | 0.556328 | -0.257651 |
| C | -1.370318 | -0.404445 | -0.341884 | -1.373260 | -0.402249 | -0.279319 | -1.371566 | -0.395962 | -0.204985 | -1.365245 | -0.397064 | -0.341683 | -1.372498 | -0.398896 | -0.227000 | -1.371144 | -0.395691 | -0.248140 |
| C | 0.271117 | 2.927248 | -0.828699 | 0.268753 | 2.923202 | -0.799797 | 0.265002 | 2.919527 | -0.795557 | 0.271590 | 2.898963 | -0.998188 | 0.268447 | 2.914379 | -0.816166 | 0.271741 | 2.922689 | -0.805190 |
| C | -0.415109 | 4.126495 | -0.960852 | -0.416695 | 4.120420 | -0.951810 | -0.422268 | 4.112831 | -0.971310 | -0.413018 | 4.095606 | -1.159272 | -0.417298 | 4.109321 | -0.987798 | -0.413518 | 4.119020 | -0.966919 |
| C | -1.795202 | 4.172578 | -0.845925 | -1.798344 | 4.166192 | -0.846260 | -1.805767 | 4.156869 | -0.868080 | -1.791492 | 4.150931 | -1.003472 | -1.800327 | 4.156134 | -0.877830 | -1.795791 | 4.165601 | -0.860400 |
| C | -2.525374 | 3.015820 | -0.607087 | -2.530093 | 3.011593 | -0.601298 | -2.537117 | 3.005173 | -0.606426 | -2.523218 | 3.007196 | -0.707468 | -2.532850 | 3.005453 | -0.614098 | -2.528270 | 3.013013 | -0.606338 |
| O | -1.575869 | -1.585891 | -0.213535 | -1.573161 | -1.581898 | -0.116507 | -1.560410 | -1.569723 | 0.017545 | -1.549370 | -1.576298 | -0.119285 | -1.563062 | -1.574335 | -0.013221 | -1.566650 | -1.573289 | -0.056470 |
| C | 4.788457 | -0.229298 | 0.239016 | 4.795250 | -0.216442 | 0.257309 | 4.798606 | -0.207063 | 0.271764 | 4.760362 | -0.225957 | 0.291986 | 4.794882 | -0.207712 | 0.273248 | 4.795205 | -0.220032 | 0.258287 |
| C | 5.360706 | -0.808749 | 1.366766 | 5.357457 | -0.701775 | 1.432899 | 5.350034 | -0.614840 | 1.481064 | 5.274682 | -0.744581 | 1.474868 | 5.341183 | -0.617807 | 1.484257 | 5.355529 | -0.697955 | 1.437775 |
| C | 6.674911 | -0.550290 | 1.689858 | 6.678912 | -0.443546 | 1.726961 | 6.676517 | -0.357332 | 1.757105 | 6.581472 | -0.484020 | 1.830326 | 6.665502 | -0.357027 | 1.768154 | 6.677518 | -0.438304 | 1.730315 |
| C | 7.435007 | 0.310780 | 0.898796 | 7.456062 | 0.319547 | 0.854639 | 7.469029 | 0.322036 | 0.829253 | 7.385556 | 0.310661 | 1.012218 | 7.460342 | 0.328239 | 0.846650 | 7.455703 | 0.317337 | 0.851858 |
| C | 6.860650 | 0.901363 | -0.221405 | 6.891662 | 0.812836 | -0.317644 | 6.914815 | 0.733733 | -0.380381 | 6.869221 | 0.834582 | -0.168829 | 6.911127 | 0.743648 | -0.363904 | 6.893073 | 0.802326 | -0.325144 |
| C | 5.541473 | 0.621068 | -0.547721 | 5.564416 | 0.534368 | -0.611325 | 5.581811 | 0.459238 | -0.652643 | 5.557221 | 0.555535 | -0.524611 | 5.580400 | 0.465774 | -0.644304 | 5.565275 | 0.523238 | -0.616787 |
| O | 8.709715 | 0.509813 | 1.299489 | 8.734993 | 0.525616 | 1.229852 | 8.750488 | 0.532893 | 1.187889 | 8.649383 | 0.517330 | 1.446088 | 8.739879 | 0.540938 | 1.212690 | 8.735041 | 0.525185 | 1.225878 |
| C | 9.520768 | 1.380359 | 0.545659 | 9.565218 | 1.301578 | 0.392211 | 9.597023 | 1.220516 | 0.282899 | 9.511420 | 1.321930 | 0.656940 | 9.591496 | 1.233230 | 0.315905 | 9.569722 | 1.291308 | 0.378894 |
| C | -3.726947 | 0.258605 | -0.057006 | -3.736413 | 0.256856 | -0.049881 | -3.739975 | 0.254967 | -0.032719 | -3.718158 | 0.260536 | -0.063314 | -3.738608 | 0.255535 | -0.037743 | -3.735151 | 0.259863 | -0.040234 |
| C | -4.400962 | 0.914680 | 0.967011 | -4.415763 | 0.880683 | 0.990454 | -4.426260 | 0.861831 | 1.011939 | -4.349357 | 0.877518 | 1.009723 | -4.416548 | 0.859829 | 1.014009 | -4.416445 | 0.877825 | 1.001786 |
| C | -5.730890 | 0.632039 | 1.209246 | -5.749828 | 0.597803 | 1.206842 | -5.762957 | 0.574849 | 1.210519 | -5.674006 | 0.593491 | 1.277726 | -5.752481 | 0.574574 | 1.221019 | -5.751217 | 0.592210 | 1.212727 |
| C | -6.388331 | -0.320345 | 0.442205 | -6.403285 | -0.321366 | 0.395509 | -6.406763 | -0.329650 | 0.375034 | -6.360625 | -0.316953 | 0.483141 | -6.403919 | -0.325901 | 0.386843 | -6.400661 | -0.323111 | 0.393544 |
| C | -5.712667 | -0.984198 | -0.568825 | -5.723354 | -0.951205 | -0.634049 | -5.720358 | -0.938382 | -0.663992 | -5.728559 | -0.934889 | -0.583975 | -5.725743 | -0.932251 | -0.659103 | -5.718636 | -0.945670 | -0.639642 |
| C | -4.383575 | -0.693122 | -0.822704 | -4.389272 | -0.658144 | -0.861126 | -4.383983 | -0.640935 | -0.872030 | -4.403835 | -0.640543 | -0.862313 | -4.390175 | -0.636449 | -0.875599 | -4.384059 | -0.649710 | -0.860817 |
| C | -7.845854 | -0.588991 | 0.701466 | -7.859941 | -0.596428 | 0.645427 | -7.865250 | -0.607233 | 0.601555 | -7.801656 | -0.600385 | 0.799669 | -7.861749 | -0.601841 | 0.621856 | -7.858013 | -0.599617 | 0.634723 |
| F | -8.110579 | -0.675065 | 2.013082 | -8.099200 | -0.894466 | 1.932806 | -8.134923 | -0.868185 | 1.892294 | -7.972289 | -0.958420 | 2.084787 | -8.124840 | -0.859413 | 1.914548 | -8.111455 | -0.870069 | 1.926003 |
| F | -8.263485 | -1.726831 | 0.136052 | -8.327064 | -1.614252 | -0.085951 | -8.313226 | -1.650785 | -0.106591 | -8.316827 | -1.583309 | 0.052283 | -8.314148 | -1.646765 | -0.081524 | -8.314685 | -1.635875 | -0.077791 |
| F | -8.620003 | 0.397309 | 0.216926 | -8.623103 | 0.473810 | 0.356401 | -8.633149 | 0.447057 | 0.263174 | -8.578667 | 0.482346 | 0.604997 | -8.630627 | 0.452271 | 0.284758 | -8.625105 | 0.459931 | 0.313728 |
| H | -0.199455 | -2.837952 | -1.108251 | -0.196157 | -2.828051 | -1.076224 | -0.198242 | -2.797310 | -1.084109 | -0.173882 | -2.766788 | -1.369599 | -0.194780 | -2.805937 | -1.099973 | -0.203769 | -2.814252 | -1.089957 |
| H | 1.034344 | -4.948506 | -1.338325 | 1.044510 | -4.930921 | -1.338923 | 1.042127 | -4.897484 | -1.379181 | 1.064901 | -4.872578 | -1.639651 | 1.048304 | -4.906200 | -1.387839 | 1.031529 | -4.918777 | -1.366839 |
| H | 3.463944 | -5.031855 | -0.928105 | 3.476227 | -5.010863 | -0.936858 | 3.473097 | -4.986316 | -0.971424 | 3.471720 | -4.987239 | -1.117391 | 3.478500 | -4.991968 | -0.973854 | 3.463029 | -5.008554 | -0.963284 |
| H | 4.702032 | -2.965655 | -0.327000 | 4.710301 | -2.948543 | -0.315028 | 4.710371 | -2.935932 | -0.318109 | 4.693250 | -2.961206 | -0.369822 | 4.711916 | -2.939343 | -0.319432 | 4.703856 | -2.954668 | -0.327887 |
| H | 1.343260 | 2.905876 | -0.912955 | 1.341728 | 2.902190 | -0.878444 | 1.338354 | 2.897842 | -0.878173 | 1.339699 | 2.866469 | -1.136966 | 1.341352 | 2.890799 | -0.904390 | 1.344734 | 2.900452 | -0.888377 |
| H | -3.604522 | 3.039753 | -0.547929 | -3.609718 | 3.037764 | -0.548841 | -3.617019 | 3.031998 | -0.555167 | -3.600790 | 3.042466 | -0.621999 | -3.612427 | 3.034069 | -0.557977 | -3.607917 | 3.040643 | -0.552974 |
| H | 0.140147 | 5.034244 | -1.156069 | 0.139040 | 5.026617 | -1.154403 | 0.130736 | 5.016785 | -1.192200 | 0.136896 | 4.992776 | -1.413090 | 0.136516 | 5.012256 | -1.210717 | 0.141843 | 5.023553 | -1.178572 |
| H | -2.316299 | 5.115441 | -0.953217 | -2.319219 | 5.107834 | -0.966506 | -2.328201 | 5.095548 | -1.004954 | -2.311194 | 5.092566 | -1.130432 | -2.321474 | 5.095998 | -1.011318 | -2.316531 | 5.106500 | -0.987680 |
| H | 4.766680 | -1.464927 | 1.990300 | 4.751390 | -1.283414 | 2.116221 | 4.734305 | -1.136544 | 2.203397 | 4.647503 | -1.353815 | 2.113935 | 4.723408 | -1.144085 | 2.201460 | 4.749066 | -1.274869 | 2.124891 |
| H | 5.092713 | 1.080623 | -1.417880 | 5.124084 | 0.912729 | -1.524783 | 5.150083 | 0.770324 | -1.595792 | 5.153379 | 0.954058 | -1.446944 | 5.152077 | 0.779935 | -1.587978 | 5.126144 | 0.894111 | -1.534203 |
| H | 7.133068 | -0.995553 | 2.562757 | 7.128623 | -0.816301 | 2.637918 | 7.116768 | -0.672299 | 2.694658 | 6.995594 | -0.883548 | 2.747516 | 7.102309 | -0.673639 | 2.706763 | 7.125968 | -0.805867 | 2.644322 |
| H | 7.424291 | 1.576047 | -0.848957 | 7.469824 | 1.408444 | -1.009063 | 7.505043 | 1.259593 | -1.117020 | 7.472073 | 1.451735 | -0.819206 | 7.503947 | 1.274833 | -1.094578 | 7.472957 | 1.391014 | -1.021171 |
| H | 9.107757 | 2.393493 | 0.523940 | 9.178618 | 2.318423 | 0.275388 | 9.229297 | 2.230712 | 0.083167 | 9.118731 | 2.336655 | 0.552543 | 9.223441 | 2.243680 | 0.118548 | 9.185263 | 2.307493 | 0.253613 |
| H | 10.487913 | 1.398811 | 1.042243 | 10.534842 | 1.344367 | 0.884040 | 10.569091 | 1.282337 | 0.767569 | 10.459395 | 1.355074 | 1.189643 | 10.560084 | 1.293313 | 0.807520 | 10.539132 | 1.334008 | 0.870967 |
| H | 9.649660 | 1.020129 | -0.479708 | 9.684511 | 0.840743 | -0.593113 | 9.698568 | 0.675794 | -0.659784 | 9.666496 | 0.882620 | -0.331942 | 9.698631 | 0.692196 | -0.628191 | 9.684774 | 0.819335 | -0.601158 |
| H | -3.877058 | 1.639041 | 1.576309 | -3.894985 | 1.580669 | 1.630339 | -3.912572 | 1.553548 | 1.666587 | -3.802577 | 1.573036 | 1.632875 | -3.896936 | 1.548320 | 1.667341 | -3.898426 | 1.575768 | 1.646222 |
| H | -3.851190 | -1.210540 | -1.607367 | -3.854406 | -1.142869 | -1.665517 | -3.843958 | -1.102219 | -1.687510 | -3.905549 | -1.109561 | -1.700035 | -3.856476 | -1.096674 | -1.695750 | -3.846887 | -1.126141 | -1.669113 |
| H | -6.253662 | 1.141587 | 2.007823 | -6.278916 | 1.083772 | 2.016483 | -6.298898 | 1.049301 | 2.022559 | -6.167321 | 1.074386 | 2.112882 | -6.282000 | 1.046905 | 2.038470 | -6.282664 | 1.074111 | 2.023314 |
| H | -6.221695 | -1.729773 | -1.163435 | -6.228869 | -1.667647 | -1.266453 | -6.219337 | -1.638201 | -1.320083 | -6.259780 | -1.640355 | -1.207932 | -6.230632 | -1.629188 | -1.313738 | -6.221209 | -1.657494 | -1.279754 |

| **iso-I** 29 |  | gasphase |  |  | toluene |  |  | DMSO |  |  | methanol |  |  | acetonitrile |  |  | chloroform |  |
| --- | --- | --- | --- | --- | --- | --- | --- | --- | --- | --- | --- | --- | --- | --- | --- | --- | --- | --- |
| Element | x | y | z | x | y | z | x | y | z | x | y | z | x | y | z | x | y | z |
| C | 0.156238 | 3.041253 | -0.400422 | 0.140762 | 3.011780 | -0.472412 | 0.111664 | 2.932563 | -0.705519 | 0.145802 | 2.995702 | -0.646608 | 0.112773 | 2.936754 | -0.711775 | 0.133824 | 2.997378 | -0.543398 |
| C | -0.604869 | 4.202195 | -0.433751 | -0.627978 | 4.165979 | -0.544190 | -0.670173 | 4.068825 | -0.868349 | -0.620518 | 4.151137 | -0.726888 | -0.668917 | 4.074323 | -0.867687 | -0.639467 | 4.147553 | -0.634823 |
| C | -1.984578 | 4.152572 | -0.313521 | -2.009458 | 4.109066 | -0.436933 | -2.052829 | 4.001948 | -0.760190 | -1.999762 | 4.102080 | -0.573692 | -2.051319 | 4.008129 | -0.754929 | -2.021347 | 4.087305 | -0.524143 |
| C | -2.641418 | 2.935977 | -0.182768 | -2.659322 | 2.891607 | -0.280348 | -2.689445 | 2.792431 | -0.510910 | -2.651993 | 2.891972 | -0.368481 | -2.687965 | 2.797968 | -0.507808 | -2.667089 | 2.870344 | -0.344425 |
| C | -1.873305 | 1.790921 | -0.166703 | -1.881721 | 1.754170 | -0.222183 | -1.897608 | 1.673283 | -0.357590 | -1.875758 | 1.755026 | -0.301237 | -1.896364 | 1.677793 | -0.361066 | -1.884430 | 1.737767 | -0.264899 |
| C | -0.471027 | 1.809092 | -0.247247 | -0.479374 | 1.780326 | -0.289057 | -0.496489 | 1.714524 | -0.421419 | -0.477121 | 1.777328 | -0.399770 | -0.495333 | 1.718009 | -0.429694 | -0.482567 | 1.768175 | -0.333697 |
| N | -2.333606 | 0.478796 | -0.079739 | -2.333366 | 0.441397 | -0.115830 | -2.330208 | 0.362670 | -0.169108 | -2.322389 | 0.439350 | -0.166649 | -2.329300 | 0.366425 | -0.175647 | -2.330237 | 0.424284 | -0.139380 |
| C | -1.284082 | -0.411917 | -0.121815 | -1.282178 | -0.443049 | -0.143357 | -1.267988 | -0.505700 | -0.144952 | -1.277192 | -0.438361 | -0.216645 | -1.267972 | -0.502304 | -0.159518 | -1.277816 | -0.454899 | -0.161073 |
| C | -0.012654 | 0.412485 | -0.232962 | -0.013900 | 0.387134 | -0.249788 | -0.013655 | 0.334995 | -0.296567 | -0.015252 | 0.387673 | -0.348834 | -0.013139 | 0.337500 | -0.309742 | -0.013267 | 0.377592 | -0.277104 |
| C | 1.225485 | -0.142630 | -0.258779 | 1.229451 | -0.155483 | -0.252624 | 1.238811 | -0.179685 | -0.255408 | 1.228641 | -0.146744 | -0.335646 | 1.238720 | -0.179111 | -0.268520 | 1.231805 | -0.159028 | -0.268272 |
| O | -1.414339 | -1.607438 | -0.020264 | -1.405328 | -1.639801 | -0.030060 | -1.368496 | -1.695829 | 0.053607 | -1.392162 | -1.641472 | -0.083665 | -1.369639 | -1.693995 | 0.031393 | -1.394728 | -1.652534 | -0.032460 |
| C | 1.678804 | -1.524820 | -0.473108 | 1.699944 | -1.530318 | -0.471101 | 1.737837 | -1.548151 | -0.427642 | 1.706693 | -1.508679 | -0.585966 | 1.736653 | -1.548711 | -0.437565 | 1.709091 | -1.531449 | -0.481205 |
| C | 3.074581 | -1.538288 | -0.322721 | 3.094039 | -1.528386 | -0.305717 | 3.128034 | -1.513887 | -0.239636 | 3.089750 | -1.515514 | -0.355250 | 3.125814 | -1.516582 | -0.241212 | 3.100677 | -1.524340 | -0.298357 |
| N | 3.534639 | -0.261194 | -0.001272 | 3.534270 | -0.249334 | 0.033030 | 3.535702 | -0.217820 | 0.073374 | 3.509492 | -0.246473 | 0.052678 | 3.533773 | -0.220285 | 0.071781 | 3.531037 | -0.242881 | 0.045661 |
| C | 2.492978 | 0.644055 | 0.015100 | 2.483370 | 0.640186 | 0.055985 | 2.468148 | 0.647281 | 0.066142 | 2.461464 | 0.633330 | 0.062999 | 2.468425 | 0.646411 | 0.054578 | 2.477861 | 0.640501 | 0.056935 |
| C | 1.055950 | -2.698077 | -0.885876 | 1.095061 | -2.706687 | -0.901087 | 1.160847 | -2.742799 | -0.844905 | 1.120642 | -2.653787 | -1.113846 | 1.160526 | -2.742992 | -0.857587 | 1.113240 | -2.707664 | -0.924191 |
| C | 1.813831 | -3.840844 | -1.102043 | 1.867880 | -3.839812 | -1.115840 | 1.957769 | -3.866783 | -1.017892 | 1.901644 | -3.779141 | -1.340743 | 1.956918 | -3.868563 | -1.024166 | 1.892706 | -3.838220 | -1.130174 |
| C | 3.188035 | -3.827559 | -0.922860 | 3.239952 | -3.812627 | -0.917341 | 3.326111 | -3.809230 | -0.790832 | 3.261786 | -3.767754 | -1.062508 | 3.323922 | -3.813041 | -0.788099 | 3.262255 | -3.807058 | -0.910697 |
| C | 3.841181 | -2.664904 | -0.536339 | 3.875896 | -2.645367 | -0.515308 | 3.934404 | -2.620975 | -0.405847 | 3.880719 | -2.623126 | -0.574515 | 3.931626 | -2.625008 | -0.400801 | 3.889450 | -2.638164 | -0.498294 |
| O | 2.628198 | 1.814541 | 0.275503 | 2.595492 | 1.806103 | 0.352425 | 2.542155 | 1.818045 | 0.364058 | 2.544720 | 1.787359 | 0.434820 | 2.544122 | 1.819409 | 0.344769 | 2.578138 | 1.807113 | 0.361658 |
| C | -3.695184 | 0.090068 | 0.011065 | -3.696374 | 0.053335 | -0.008506 | -3.686309 | -0.032850 | -0.013908 | -3.680342 | 0.047015 | -0.003746 | -3.684790 | -0.030443 | -0.015578 | -3.692088 | 0.032706 | -0.014226 |
| C | -4.571700 | 0.376451 | -1.030248 | -4.526922 | 0.119672 | -1.121507 | -4.330733 | -0.726293 | -1.031694 | -4.540297 | 0.058801 | -1.095562 | -4.334747 | -0.716861 | -1.034830 | -4.492958 | -0.064441 | -1.146296 |
| C | -5.896720 | 0.007587 | -0.945005 | -5.852662 | -0.243689 | -1.019057 | -5.648375 | -1.105245 | -0.882265 | -5.858388 | -0.315209 | -0.937737 | -5.651623 | -1.097131 | -0.880571 | -5.816646 | -0.431589 | -1.026936 |
| C | -6.363242 | -0.673772 | 0.178747 | -6.364779 | -0.691473 | 0.199374 | -6.345215 | -0.783933 | 0.284648 | -6.328231 | -0.714299 | 0.314300 | -6.341403 | -0.784175 | 0.292755 | -6.355734 | -0.713136 | 0.229575 |
| C | -5.485248 | -0.973547 | 1.214581 | -5.532243 | -0.764478 | 1.311985 | -5.701194 | -0.082688 | 1.301006 | -5.466055 | -0.730687 | 1.406595 | -5.691648 | -0.091373 | 1.311227 | -5.552969 | -0.619239 | 1.362962 |
| C | -4.156783 | -0.582983 | 1.125927 | -4.202217 | -0.385325 | 1.200257 | -4.371466 | 0.283301 | 1.145177 | -4.143918 | -0.343557 | 1.239030 | -4.362837 | 0.276071 | 1.150467 | -4.223785 | -0.242513 | 1.231670 |
| O | -7.674198 | -1.001016 | 0.168709 | -7.670154 | -1.030255 | 0.202545 | -7.629201 | -1.188941 | 0.338280 | -7.632061 | -1.066593 | 0.370704 | -7.625817 | -1.188843 | 0.349506 | -7.657258 | -1.066178 | 0.247331 |
| C | -8.198194 | -1.701332 | 1.272544 | -8.239073 | -1.502623 | 1.405275 | -8.380142 | -0.887899 | 1.501834 | -8.165062 | -1.487716 | 1.616370 | -8.372379 | -0.898863 | 1.518835 | -8.256914 | -1.370784 | 1.491975 |
| C | 6.845085 | -0.275857 | 1.635755 | 6.818872 | -0.171274 | 1.721794 | 6.775889 | 0.011999 | 1.833524 | 6.703993 | -0.194600 | 1.907456 | 6.759637 | 0.021712 | 1.857464 | 6.787318 | -0.126784 | 1.787108 |
| C | 5.531763 | -0.595994 | 1.334177 | 5.507328 | -0.503426 | 1.423285 | 5.474308 | -0.354494 | 1.527871 | 5.409256 | -0.524592 | 1.538442 | 5.459651 | -0.345000 | 1.544752 | 5.479870 | -0.464816 | 1.475622 |
| C | 4.875383 | 0.075527 | 0.311299 | 4.873566 | 0.101056 | 0.346418 | 4.864934 | 0.166222 | 0.394831 | 4.833812 | 0.095805 | 0.438619 | 4.861870 | 0.163548 | 0.399976 | 4.866420 | 0.113309 | 0.372939 |
| C | 5.524430 | 1.076129 | -0.397197 | 5.540569 | 1.042538 | -0.423436 | 5.543432 | 1.052857 | -0.428749 | 5.537046 | 1.045282 | -0.288432 | 5.549948 | 1.038456 | -0.428320 | 5.547609 | 1.032367 | -0.411666 |
| C | 6.832819 | 1.401391 | -0.079990 | 6.847139 | 1.380622 | -0.110322 | 6.840084 | 1.425276 | -0.109539 | 6.826403 | 1.379944 | 0.095415 | 6.844982 | 1.411241 | -0.102269 | 6.850066 | 1.376398 | -0.085952 |
| C | 7.497713 | 0.724387 | 0.931179 | 7.489606 | 0.772444 | 0.957982 | 7.458661 | 0.903831 | 1.018262 | 7.412215 | 0.759125 | 1.189705 | 7.452123 | 0.901758 | 1.037307 | 7.472824 | 0.795456 | 1.009411 |
| H | 1.228093 | 3.095810 | -0.470348 | 1.212812 | 3.073323 | -0.539668 | 1.183227 | 2.999892 | -0.788225 | 1.214533 | 3.049787 | -0.772625 | 1.184016 | 3.003353 | -0.798782 | 1.205626 | 3.061657 | -0.619696 |
| H | -0.107355 | 5.156457 | -0.544900 | -0.136539 | 5.120884 | -0.677679 | -0.191571 | 5.016454 | -1.079879 | -0.132580 | 5.099977 | -0.909831 | -0.190279 | 5.022285 | -1.077552 | -0.152363 | 5.101811 | -0.788537 |
| H | -2.561873 | 5.068515 | -0.325902 | -2.592914 | 5.020282 | -0.482127 | -2.647519 | 4.898836 | -0.882386 | -2.580850 | 5.014439 | -0.628247 | -2.645778 | 4.905867 | -0.871799 | -2.608109 | 4.995446 | -0.586642 |
| H | -3.718124 | 2.883394 | -0.098817 | -3.737284 | 2.832016 | -0.213069 | -3.767618 | 2.726349 | -0.450269 | -3.728969 | 2.841225 | -0.277383 | -3.765921 | 2.732034 | -0.443767 | -3.745151 | 2.807462 | -0.278177 |
| H | -0.010925 | -2.724931 | -1.020356 | 0.030282 | -2.743666 | -1.051914 | 0.100366 | -2.800915 | -1.022341 | 0.068637 | -2.672995 | -1.345733 | 0.101300 | -2.799489 | -1.042794 | 0.051139 | -2.746732 | -1.094836 |
| H | 4.915778 | -2.636570 | -0.421444 | 4.948581 | -2.608121 | -0.383493 | 5.003655 | -2.563400 | -0.252604 | 4.945849 | -2.598931 | -0.387379 | 4.999918 | -2.568267 | -0.240778 | 4.960188 | -2.598420 | -0.350911 |
| H | 1.319231 | -4.751423 | -1.413002 | 1.387643 | -4.753730 | -1.440281 | 1.502584 | -4.796951 | -1.333095 | 1.441419 | -4.673078 | -1.741793 | 1.502244 | -4.798243 | -1.341478 | 1.420804 | -4.752958 | -1.464846 |
| H | 3.764736 | -4.727681 | -1.094653 | 3.828379 | -4.705893 | -1.086312 | 3.933448 | -4.695780 | -0.925376 | 3.856112 | -4.655266 | -1.241774 | 3.930683 | -4.700726 | -0.917581 | 3.855682 | -4.698458 | -1.072327 |
| H | -4.206238 | 0.891605 | -1.909624 | -4.126029 | 0.458160 | -2.068790 | -3.793947 | -0.967477 | -1.940701 | -4.172043 | 0.363783 | -2.067327 | -3.802513 | -0.951681 | -1.948180 | -4.071012 | 0.149542 | -2.120456 |
| H | -3.470858 | -0.817469 | 1.928646 | -3.551561 | -0.437961 | 2.063702 | -3.866196 | 0.823558 | 1.936353 | -3.469078 | -0.348968 | 2.086029 | -3.852740 | 0.810050 | 1.942771 | -3.594717 | -0.166467 | 2.109727 |
| H | -6.589964 | 0.225717 | -1.746264 | -6.510404 | -0.197011 | -1.877130 | -6.158632 | -1.648431 | -1.667532 | -6.540276 | -0.309624 | -1.778817 | -6.167323 | -1.635047 | -1.665918 | -6.452155 | -0.511625 | -1.899501 |
| H | -5.817952 | -1.505934 | 2.093536 | -5.903071 | -1.110899 | 2.265666 | -6.216199 | 0.178776 | 2.214176 | -5.807111 | -1.036247 | 2.385307 | -6.202002 | 0.162323 | 2.229168 | -5.945928 | -0.834001 | 2.346145 |
| H | -7.705144 | -2.669531 | 1.402950 | -7.749573 | -2.419379 | 1.747533 | -7.943215 | -1.353501 | 2.389462 | -7.653325 | -2.381442 | 1.982626 | -7.930893 | -1.372566 | 2.399814 | -7.768033 | -2.223298 | 1.971796 |
| H | -8.105246 | -1.121094 | 2.195809 | -8.192646 | -0.746987 | 2.195391 | -8.459175 | 0.191591 | 1.656950 | -8.099175 | -0.692390 | 2.363351 | -8.450271 | 0.179202 | 1.683695 | -8.236667 | -0.509442 | 2.165804 |
| H | -9.251560 | -1.862627 | 1.056316 | -9.281788 | -1.718610 | 1.181613 | -9.373350 | -1.299036 | 1.334059 | -9.211036 | -1.721635 | 1.429931 | -9.365834 | -1.309035 | 1.350511 | -9.290905 | -1.628421 | 1.272385 |
| H | 7.355129 | -0.802536 | 2.432035 | 7.313620 | -0.644659 | 2.560360 | 7.254109 | -0.395961 | 2.715136 | 7.156886 | -0.679389 | 2.763246 | 7.228939 | -0.376622 | 2.748212 | 7.267496 | -0.579318 | 2.645530 |
| H | 5.009197 | -1.363675 | 1.890382 | 4.970859 | -1.228564 | 2.022134 | 4.929510 | -1.042846 | 2.161958 | 4.844589 | -1.260240 | 2.097673 | 4.907163 | -1.024092 | 2.182126 | 4.932224 | -1.174386 | 2.083152 |
| H | 8.521790 | 0.978422 | 1.172396 | 8.512292 | 1.035538 | 1.196763 | 8.473711 | 1.192079 | 1.261559 | 8.421772 | 1.018692 | 1.483380 | 8.465759 | 1.190264 | 1.286115 | 8.492232 | 1.062634 | 1.258086 |
| H | 4.999088 | 1.600191 | -1.183364 | 5.033435 | 1.507539 | -1.258251 | 5.056415 | 1.444523 | -1.312537 | 5.074681 | 1.515474 | -1.147032 | 5.071157 | 1.421135 | -1.320517 | 5.056080 | 1.474143 | -1.268604 |
| H | 7.336055 | 2.186071 | -0.630013 | 7.366083 | 2.119533 | -0.707591 | 7.370409 | 2.119743 | -0.749036 | 7.376419 | 2.123612 | -0.467636 | 7.382808 | 2.096619 | -0.745273 | 7.381141 | 2.097431 | -0.694517 |

| **iso-I** 30 |  | gasphase |  |  | toluene |  |  | DMSO |  |  | methanol |  |  | acetonitrile |  |  | chloroform |  |
| --- | --- | --- | --- | --- | --- | --- | --- | --- | --- | --- | --- | --- | --- | --- | --- | --- | --- | --- |
| Element | x | y | z | x | y | z | x | y | z | x | y | z | x | y | z | x | y | z |
| C | -1.205297 | 3.246809 | -0.115382 | -1.175321 | 3.231648 | -0.167371 | -1.129330 | 3.201532 | -0.328903 | -1.195982 | 3.213643 | -0.252844 | -1.137975 | 3.206822 | -0.326445 | -1.170001 | 3.225024 | -0.211745 |
| C | -0.352403 | 4.339671 | -0.197583 | -0.310628 | 4.312074 | -0.285351 | -0.248475 | 4.258321 | -0.522039 | -0.340586 | 4.299168 | -0.391389 | -0.259847 | 4.268410 | -0.506735 | -0.302875 | 4.302184 | -0.345072 |
| C | 1.023064 | 4.171317 | -0.193174 | 1.063590 | 4.126733 | -0.294580 | 1.123462 | 4.046903 | -0.539705 | 1.036823 | 4.123750 | -0.392066 | 1.113090 | 4.062005 | -0.519331 | 1.071637 | 4.113012 | -0.356177 |
| C | 1.581468 | 2.901198 | -0.131584 | 1.607538 | 2.851555 | -0.210509 | 1.647524 | 2.769037 | -0.388403 | 1.594052 | 2.854636 | -0.287582 | 1.641190 | 2.784516 | -0.376787 | 1.613273 | 2.837228 | -0.259827 |
| C | 0.721677 | 1.825355 | -0.065089 | 0.734663 | 1.789264 | -0.104876 | 0.757834 | 1.731309 | -0.203175 | 0.729108 | 1.789127 | -0.160239 | 0.754237 | 1.742254 | -0.204820 | 0.737523 | 1.779149 | -0.137440 |
| C | -0.675803 | 1.963326 | -0.029648 | -0.660062 | 1.944453 | -0.055202 | -0.631905 | 1.916324 | -0.141765 | -0.663943 | 1.938085 | -0.102527 | -0.636376 | 1.922000 | -0.147178 | -0.656082 | 1.938903 | -0.083024 |
| N | 1.073305 | 0.477828 | -0.030324 | 1.070175 | 0.439226 | -0.054828 | 1.066027 | 0.377907 | -0.094520 | 1.065329 | 0.435555 | -0.105176 | 1.066045 | 0.388374 | -0.108299 | 1.067878 | 0.427802 | -0.080034 |
| C | -0.048201 | -0.320030 | 0.006256 | -0.056652 | -0.345323 | -0.008190 | -0.073901 | -0.379564 | -0.002640 | -0.055307 | -0.344123 | -0.056536 | -0.071589 | -0.373164 | -0.031438 | -0.059647 | -0.351507 | -0.029815 |
| C | -1.249952 | 0.610370 | 0.010386 | -1.248086 | 0.598435 | 0.005998 | -1.243604 | 0.587639 | -0.014701 | -1.242931 | 0.594022 | -0.029763 | -1.244030 | 0.590404 | -0.031934 | -1.246735 | 0.595474 | -0.011177 |
| C | -2.529897 | 0.164699 | 0.080596 | -2.532774 | 0.172776 | 0.101237 | -2.534544 | 0.199753 | 0.123238 | -2.521619 | 0.172688 | 0.112047 | -2.532721 | 0.197330 | 0.112470 | -2.531114 | 0.176703 | 0.104277 |
| O | -0.011906 | -1.524060 | 0.080274 | -0.034272 | -1.550925 | 0.070058 | -0.078136 | -1.581632 | 0.143437 | -0.037354 | -1.556346 | 0.037465 | -0.072331 | -1.577833 | 0.095188 | -0.042622 | -1.558777 | 0.053438 |
| C | -3.119219 | -1.176474 | -0.090814 | -3.146977 | -1.155337 | -0.074630 | -3.184103 | -1.111267 | -0.023338 | -3.146913 | -1.136307 | -0.108686 | -3.178705 | -1.115515 | -0.034329 | -3.154342 | -1.145377 | -0.072037 |
| C | -4.498856 | -1.062894 | 0.151464 | -4.520135 | -1.019182 | 0.191325 | -4.544045 | -0.937719 | 0.283511 | -4.491781 | -1.023959 | 0.276622 | -4.534047 | -0.951140 | 0.296891 | -4.519198 | -1.006231 | 0.231093 |
| N | -4.805158 | 0.245929 | 0.480865 | -4.797473 | 0.289569 | 0.543881 | -4.778359 | 0.378849 | 0.638329 | -4.735061 | 0.258942 | 0.745198 | -4.768495 | 0.362456 | 0.663949 | -4.781536 | 0.299968 | 0.606641 |
| C | -3.707619 | 1.060707 | 0.438608 | -3.687935 | 1.081683 | 0.498968 | -3.652681 | 1.139404 | 0.554820 | -3.634716 | 1.046722 | 0.662998 | -3.649004 | 1.129222 | 0.564525 | -3.671057 | 1.085136 | 0.541850 |
| C | -2.637297 | -2.412831 | -0.508867 | -2.694154 | -2.393093 | -0.520313 | -2.774066 | -2.356609 | -0.489307 | -2.732127 | -2.320456 | -0.709076 | -2.770358 | -2.355078 | -0.517157 | -2.717769 | -2.376152 | -0.552203 |
| C | -3.510582 | -3.485264 | -0.638573 | -3.588386 | -3.447890 | -0.651171 | -3.697389 | -3.389202 | -0.593190 | -3.635298 | -3.365418 | -0.854157 | -3.690124 | -3.391949 | -0.612255 | -3.619588 | -3.425538 | -0.675666 |
| C | -4.861450 | -3.345592 | -0.361145 | -4.932465 | -3.287125 | -0.348864 | -5.027913 | -3.194430 | -0.244798 | -4.948617 | -3.238498 | -0.419148 | -5.015325 | -3.206738 | -0.238830 | -4.954786 | -3.263924 | -0.332841 |
| C | -5.377131 | -2.118481 | 0.034421 | -5.419802 | -2.057053 | 0.072711 | -5.472842 | -1.953612 | 0.192808 | -5.400544 | -2.052091 | 0.145620 | -5.459133 | -1.971153 | 0.215246 | -5.426406 | -2.038665 | 0.120599 |
| O | -3.740373 | 2.238701 | 0.703988 | -3.685555 | 2.254835 | 0.795405 | -3.597025 | 2.312598 | 0.855174 | -3.581802 | 2.197167 | 1.057932 | -3.595649 | 2.302384 | 0.867145 | -3.648640 | 2.255561 | 0.855699 |
| C | 2.399601 | -0.025802 | -0.059584 | 2.396252 | -0.071946 | -0.076859 | 2.382755 | -0.157940 | -0.084717 | 2.392410 | -0.077214 | -0.097370 | 2.383816 | -0.145822 | -0.094261 | 2.393446 | -0.087958 | -0.088976 |
| C | 3.204869 | 0.199214 | -1.171071 | 3.123673 | -0.062191 | -1.261727 | 2.881325 | -0.799370 | -1.212526 | 3.141159 | -0.080017 | -1.268381 | 2.901899 | -0.750475 | -1.233643 | 3.081643 | -0.212113 | -1.290369 |
| C | 4.496078 | -0.281182 | -1.202576 | 4.415651 | -0.541880 | -1.285393 | 4.162106 | -1.310918 | -1.206338 | 4.432481 | -0.564485 | -1.258887 | 4.182208 | -1.263722 | -1.221300 | 4.371869 | -0.698368 | -1.301036 |
| C | 4.997285 | -1.013723 | -0.126959 | 4.996119 | -1.050360 | -0.122680 | 4.967464 | -1.177720 | -0.073146 | 4.985708 | -1.060854 | -0.077748 | 4.966129 | -1.170034 | -0.069221 | 4.989363 | -1.074118 | -0.106850 |
| C | 4.189028 | -1.251542 | 0.979323 | 4.266100 | -1.067101 | 1.061844 | 4.469423 | -0.529966 | 1.054718 | 4.233967 | -1.063861 | 1.093314 | 4.448862 | -0.558550 | 1.070167 | 4.299323 | -0.952874 | 1.095935 |
| C | 2.895891 | -0.749338 | 1.007706 | 2.970081 | -0.571573 | 1.076635 | 3.175563 | -0.027717 | 1.040954 | 2.939134 | -0.564982 | 1.075315 | 3.156170 | -0.053448 | 1.049402 | 3.003639 | -0.455894 | 1.095567 |
| O | 6.269950 | -1.450215 | -0.251999 | 6.261509 | -1.501068 | -0.242789 | 6.205304 | -1.702181 | -0.161922 | 6.255279 | -1.516784 | -0.165767 | 6.204442 | -1.695371 | -0.152350 | 6.249743 | -1.541786 | -0.215581 |
| C | 6.825137 | -2.205725 | 0.799037 | 6.896412 | -2.034568 | 0.899770 | 7.065599 | -1.584973 | 0.958187 | 6.873501 | -2.025155 | 1.005816 | 7.042677 | -1.628528 | 0.988576 | 6.921380 | -1.950329 | 0.960665 |
| H | -5.714787 | 0.598086 | 0.717325 | -5.700972 | 0.646807 | 0.802625 | -5.669186 | 0.749198 | 0.929053 | -5.611541 | 0.582381 | 1.124016 | -5.654740 | 0.727526 | 0.974879 | -5.676621 | 0.654208 | 0.900652 |
| H | -2.270739 | 3.392714 | -0.097360 | -2.239010 | 3.392027 | -0.145223 | -2.190892 | 3.380453 | -0.312492 | -2.262805 | 3.365096 | -0.259269 | -2.200431 | 3.381572 | -0.314584 | -2.233767 | 3.388524 | -0.194022 |
| H | -0.773726 | 5.334426 | -0.256352 | -0.720395 | 5.310634 | -0.364021 | -0.641135 | 5.257921 | -0.657934 | -0.756699 | 5.292748 | -0.498650 | -0.655390 | 5.267649 | -0.636659 | -0.709942 | 5.300954 | -0.436186 |
| H | 1.673916 | 5.035194 | -0.242996 | 1.723875 | 4.981326 | -0.375308 | 1.796963 | 4.882705 | -0.684025 | 1.689550 | 4.982494 | -0.490003 | 1.784186 | 4.901444 | -0.653325 | 1.733556 | 4.965065 | -0.450185 |
| H | 2.652910 | 2.755818 | -0.138007 | 2.676999 | 2.690968 | -0.234089 | 2.713843 | 2.589424 | -0.424915 | 2.665275 | 2.705368 | -0.317576 | 2.708189 | 2.608422 | -0.410196 | 2.682322 | 2.673366 | -0.288895 |
| H | -1.589495 | -2.543578 | -0.713299 | -1.652665 | -2.538937 | -0.747310 | -1.744914 | -2.525232 | -0.756970 | -1.719106 | -2.432981 | -1.058632 | -1.745383 | -2.515716 | -0.805094 | -1.684020 | -2.521188 | -0.814393 |
| H | -6.432996 | -1.990262 | 0.235105 | -6.469685 | -1.911312 | 0.292188 | -6.511147 | -1.779564 | 0.443977 | -6.428941 | -1.929497 | 0.460010 | -6.493825 | -1.804478 | 0.485563 | -6.469485 | -1.891401 | 0.369387 |
| H | -3.123191 | -4.444120 | -0.956322 | -3.224252 | -4.408967 | -0.990060 | -3.370729 | -4.358273 | -0.948230 | -3.307808 | -4.289599 | -1.312961 | -3.364794 | -4.356598 | -0.980244 | -3.269893 | -4.382401 | -1.041132 |
| H | -5.524875 | -4.195527 | -0.459629 | -5.613065 | -4.123505 | -0.448890 | -5.731773 | -4.013803 | -0.324506 | -5.636331 | -4.067400 | -0.533909 | -5.716175 | -4.029290 | -0.311910 | -5.641351 | -4.096161 | -0.427665 |
| H | 2.810642 | 0.754913 | -2.012517 | 2.669007 | 0.324952 | -2.165090 | 2.259875 | -0.894562 | -2.094215 | 2.708219 | 0.301804 | -2.184762 | 2.295668 | -0.816367 | -2.128507 | 2.598436 | 0.074891 | -2.216106 |
| H | 2.263714 | -0.935217 | 1.865421 | 2.399393 | -0.579779 | 1.996369 | 2.783125 | 0.473244 | 1.917438 | 2.351042 | -0.558411 | 1.984647 | 2.747985 | 0.418980 | 1.934441 | 2.462503 | -0.357721 | 2.028303 |
| H | 5.134330 | -0.111891 | -2.059393 | 4.993277 | -0.539850 | -2.200497 | 4.560090 | -1.814291 | -2.078238 | 5.028691 | -0.570398 | -2.162696 | 4.596026 | -1.739286 | -2.101459 | 4.919197 | -0.801279 | -2.229177 |
| H | 4.549350 | -1.821062 | 1.823426 | 4.690859 | -1.458902 | 1.974649 | 5.070514 | -0.414047 | 1.945089 | 4.640526 | -1.443954 | 2.019395 | 5.034373 | -0.473967 | 1.974297 | 4.755187 | -1.238972 | 2.032745 |
| H | 6.264143 | -3.130141 | 0.967080 | 6.363806 | -2.910332 | 1.282696 | 6.652256 | -2.093936 | 1.833165 | 6.332820 | -2.892497 | 1.393359 | 6.606419 | -2.166878 | 1.834251 | 6.395615 | -2.772188 | 1.454734 |
| H | 7.838384 | -2.453006 | 0.491589 | 7.891413 | -2.336299 | 0.579000 | 7.999125 | -2.065096 | 0.672431 | 7.874795 | -2.328412 | 0.707408 | 7.977530 | -2.105831 | 0.702648 | 7.903241 | -2.294055 | 0.642002 |
| H | 6.861684 | -1.630796 | 1.729545 | 6.988833 | -1.287122 | 1.693563 | 7.259625 | -0.537007 | 1.202931 | 6.943586 | -1.257033 | 1.780424 | 7.239554 | -0.592311 | 1.276795 | 7.041818 | -1.118615 | 1.660849 |

| **iso-I** 31 |  | gasphase |  |  | toluene |  |  | DMSO |  |  | methanol |  |  | acetonitrile |  |  | chloroform |  |
| --- | --- | --- | --- | --- | --- | --- | --- | --- | --- | --- | --- | --- | --- | --- | --- | --- | --- | --- |
| Element | x | y | z | x | y | z | x | y | z | x | y | z | x | y | z | x | y | z |
| C | 0.647899 | 2.881320 | -0.534730 | 0.581460 | 2.844767 | -0.247200 | 0.555055 | 2.838212 | -0.196183 | 0.482741 | 2.835862 | -0.039816 | 0.539025 | 2.842357 | -0.156171 | 0.557393 | 2.844710 | -0.182347 |
| C | 1.491941 | 3.992805 | -0.474819 | 1.396192 | 3.961209 | -0.105045 | 1.358434 | 3.962242 | -0.047752 | 1.253671 | 3.984161 | 0.091083 | 1.335078 | 3.973381 | -0.020323 | 1.361380 | 3.970741 | -0.053281 |
| C | 2.869530 | 3.857456 | -0.629481 | 2.769744 | 3.866414 | -0.265915 | 2.731464 | 3.885702 | -0.235177 | 2.617448 | 3.958507 | -0.165802 | 2.706221 | 3.907665 | -0.225595 | 2.732142 | 3.893924 | -0.250455 |
| C | 3.440602 | 2.600319 | -0.838197 | 3.367351 | 2.645794 | -0.555505 | 3.339744 | 2.677403 | -0.554518 | 3.252180 | 2.777651 | -0.534527 | 3.320280 | 2.703417 | -0.550058 | 3.338117 | 2.682381 | -0.561673 |
| C | 2.590577 | 1.506577 | -0.895071 | 2.544717 | 1.548214 | -0.693785 | 2.527014 | 1.571827 | -0.697649 | 2.471234 | 1.648901 | -0.660282 | 2.514958 | 1.591019 | -0.680451 | 2.525670 | 1.575393 | -0.686249 |
| C | 1.188164 | 1.611274 | -0.759299 | 1.148250 | 1.616644 | -0.565538 | 1.132102 | 1.623535 | -0.548263 | 1.084628 | 1.652089 | -0.449429 | 1.121608 | 1.631944 | -0.514122 | 1.132015 | 1.626686 | -0.525622 |
| N | 2.955139 | 0.177118 | -1.070398 | 2.935747 | 0.238692 | -0.937868 | 2.928789 | 0.268855 | -0.961321 | 2.901193 | 0.357390 | -0.956559 | 2.923680 | 0.289854 | -0.945326 | 2.926517 | 0.269985 | -0.941764 |
| C | 1.862714 | -0.655208 | -0.963507 | 1.862643 | -0.612830 | -0.907605 | 1.867856 | -0.593724 | -0.917331 | 1.874710 | -0.535951 | -0.854149 | 1.870524 | -0.580720 | -0.881980 | 1.866390 | -0.593604 | -0.877601 |
| C | 0.640033 | 0.239217 | -0.820648 | 0.631005 | 0.247154 | -0.694272 | 0.628856 | 0.251358 | -0.685327 | 0.621307 | 0.268498 | -0.583869 | 0.627220 | 0.255893 | -0.644121 | 0.628581 | 0.252542 | -0.649069 |
| C | -0.640129 | -0.239948 | -0.820636 | -0.630987 | -0.247002 | -0.694286 | -0.628830 | -0.251797 | -0.685235 | -0.621307 | -0.268540 | -0.583875 | -0.627114 | -0.255713 | -0.644021 | -0.628496 | -0.252978 | -0.649062 |
| O | 1.947931 | -1.860878 | -1.028761 | 1.948602 | -1.803878 | -1.104555 | 1.959342 | -1.785496 | -1.117845 | 1.995762 | -1.730623 | -1.045814 | 1.970402 | -1.773824 | -1.072250 | 1.961562 | -1.787452 | -1.060984 |
| C | -1.188268 | -1.611988 | -0.758934 | -1.148237 | -1.616497 | -0.565617 | -1.132121 | -1.623944 | -0.547996 | -1.084640 | -1.652127 | -0.449424 | -1.121473 | -1.631777 | -0.514067 | -1.132144 | -1.627049 | -0.525522 |
| C | -2.590685 | -1.507317 | -0.894702 | -2.544703 | -1.548055 | -0.693865 | -2.527035 | -1.572195 | -0.697373 | -2.471246 | -1.648929 | -0.660278 | -2.514828 | -1.590862 | -0.680341 | -2.525790 | -1.575533 | -0.686217 |
| N | -2.955239 | -0.177904 | -1.070395 | -2.935728 | -0.238520 | -0.937884 | -2.928768 | -0.269227 | -0.961127 | -2.901195 | -0.357416 | -0.956566 | -2.923582 | -0.289676 | -0.945064 | -2.926430 | -0.270065 | -0.941747 |
| C | -1.862788 | 0.654435 | -0.963939 | -1.862622 | 0.612997 | -0.907580 | -1.867824 | 0.593334 | -0.917067 | -1.874700 | 0.535915 | -0.854182 | -1.870475 | 0.580936 | -0.881445 | -1.866174 | 0.593359 | -0.877526 |
| C | -0.648014 | -2.881970 | -0.533983 | -0.581454 | -2.844637 | -0.247333 | -0.555120 | -2.838620 | -0.195835 | -0.482766 | -2.835900 | -0.039791 | -0.538844 | -2.842224 | -0.156308 | -0.557772 | -2.845153 | -0.182093 |
| C | -1.492064 | -3.993431 | -0.473725 | -1.396192 | -3.961081 | -0.105232 | -1.358543 | -3.962608 | -0.047318 | -1.253706 | -3.984191 | 0.091118 | -1.334869 | -3.973281 | -0.020560 | -1.361964 | -3.971038 | -0.053015 |
| C | -2.869656 | -3.858114 | -0.628386 | -2.769742 | -3.866274 | -0.266101 | -2.731570 | -3.886032 | -0.234747 | -2.617483 | -3.958528 | -0.165771 | -2.706021 | -3.907570 | -0.225775 | -2.732694 | -3.894004 | -0.250307 |
| C | -3.440721 | -2.601031 | -0.837450 | -3.367343 | -2.645638 | -0.555638 | -3.339804 | -2.677734 | -0.554176 | -3.252203 | -2.777670 | -0.534508 | -3.320119 | -2.703299 | -0.550082 | -3.338436 | -2.682375 | -0.561633 |
| O | -1.948017 | 1.860099 | -1.029335 | -1.948576 | 1.804055 | -1.104467 | -1.959244 | 1.785070 | -1.117830 | -1.995745 | 1.730591 | -1.045826 | -1.970344 | 1.774007 | -1.071925 | -1.961135 | 1.787245 | -1.060781 |
| C | 4.293739 | -0.317983 | -1.121118 | 4.281780 | -0.199100 | -1.128102 | 4.282671 | -0.148982 | -1.156909 | 4.260138 | -0.025728 | -1.190361 | 4.277443 | -0.121093 | -1.157878 | 4.276378 | -0.153538 | -1.146285 |
| C | 4.967224 | -0.291322 | 0.247453 | 5.064154 | -0.273987 | 0.177205 | 5.051190 | -0.259104 | 0.153114 | 5.051001 | -0.170949 | 0.099172 | 5.061209 | -0.236852 | 0.142137 | 5.064742 | -0.238952 | 0.153480 |
| O | 6.268919 | -0.513124 | 0.107345 | 6.354149 | -0.409504 | -0.095331 | 6.338901 | -0.400985 | -0.106054 | 6.319344 | -0.382776 | -0.180399 | 6.343305 | -0.394942 | -0.132548 | 6.346669 | -0.411132 | -0.123653 |
| O | 4.376551 | -0.108770 | 1.276865 | 4.557300 | -0.232242 | 1.264153 | 4.529628 | -0.235251 | 1.237187 | 4.554028 | -0.107976 | 1.197622 | 4.554479 | -0.202547 | 1.233302 | 4.564799 | -0.172412 | 1.244529 |
| C | 7.175453 | -0.590962 | 1.247951 | 7.367017 | -0.562911 | 0.954719 | 7.348426 | -0.568698 | 0.949680 | 7.350245 | -0.599207 | 0.852736 | 7.364765 | -0.576024 | 0.909829 | 7.367518 | -0.576600 | 0.919932 |
| C | 6.765620 | -1.746422 | 2.158588 | 7.097667 | -1.828573 | 1.755066 | 7.073958 | -1.843354 | 1.731900 | 7.022885 | -1.849714 | 1.652327 | 7.082500 | -1.847546 | 1.694813 | 7.073047 | -1.823797 | 1.739446 |
| C | 8.526268 | -0.866663 | 0.597262 | 8.658433 | -0.692800 | 0.163345 | 8.643644 | -0.690693 | 0.165029 | 8.608026 | -0.803610 | 0.025862 | 8.646931 | -0.715506 | 0.106369 | 8.645400 | -0.751895 | 0.116024 |
| C | 7.190760 | 0.746285 | 1.985986 | 7.396324 | 0.681323 | 1.830372 | 7.375731 | 0.664988 | 1.838477 | 7.473345 | 0.641849 | 1.721689 | 7.420473 | 0.657577 | 1.797803 | 7.438360 | 0.679640 | 1.775113 |
| C | -4.293825 | 0.317210 | -1.121291 | -4.281760 | 0.199285 | -1.128096 | -4.282628 | 0.148630 | -1.156817 | -4.260138 | 0.025710 | -1.190367 | -4.277343 | 0.121243 | -1.157685 | -4.276222 | 0.153674 | -1.146273 |
| C | -4.967222 | 0.291331 | 0.247341 | -5.064132 | 0.274123 | 0.177215 | -5.051233 | 0.258774 | 0.153154 | -5.050993 | 0.170954 | 0.099169 | -5.061247 | 0.236716 | 0.142270 | -5.064586 | 0.239197 | 0.153485 |
| O | -6.268824 | 0.513680 | 0.107243 | -6.354146 | 0.409463 | -0.095314 | -6.338872 | 0.401132 | -0.106112 | -6.319336 | 0.382785 | -0.180399 | -6.343314 | 0.394864 | -0.132536 | -6.346498 | 0.411461 | -0.123665 |
| O | -4.376552 | 0.108932 | 1.276781 | -4.557288 | 0.232214 | 1.264161 | -4.529714 | 0.235216 | 1.237253 | -4.554009 | 0.108019 | 1.197616 | -4.554609 | 0.202282 | 1.233473 | -4.564676 | 0.172474 | 1.244539 |
| C | -7.175265 | 0.592248 | 1.247878 | -7.367045 | 0.562621 | 0.954742 | -7.348383 | 0.569423 | 0.949542 | -7.350230 | 0.599253 | 0.852737 | -7.364954 | 0.575817 | 0.909697 | -7.367379 | 0.576873 | 0.919899 |
| C | -8.526052 | 0.867994 | 0.597155 | -8.658484 | 0.692307 | 0.163373 | -8.643500 | 0.691914 | 0.164802 | -8.608023 | 0.803591 | 0.025866 | -8.646976 | 0.715327 | 0.106016 | -8.645168 | 0.752664 | 0.115951 |
| C | -6.765096 | 1.748004 | 2.157990 | -7.097940 | 1.828292 | 1.755159 | -7.073380 | 1.844043 | 1.731633 | -7.022886 | 1.849807 | 1.652261 | -7.082916 | 1.847287 | 1.694852 | -7.072676 | 1.823793 | 1.739748 |
| C | -7.190846 | -0.744675 | 1.986492 | -7.396116 | -0.681666 | 1.830327 | -7.376295 | -0.664149 | 1.838478 | -7.473297 | -0.641760 | 1.721755 | -7.420813 | -0.657870 | 1.797545 | -7.438577 | -0.679570 | 1.774755 |
| H | -0.424411 | 2.998802 | -0.419803 | -0.483939 | 2.934581 | -0.123964 | -0.508796 | 2.912293 | -0.047632 | -0.571826 | 2.867245 | 0.178677 | -0.523204 | 2.907471 | 0.007517 | -0.505058 | 2.919390 | -0.025437 |
| H | 1.058988 | 4.979691 | -0.302181 | 0.947005 | 4.915901 | 0.135633 | 0.903472 | 4.907541 | 0.219139 | 0.782022 | 4.907309 | 0.402733 | 0.875966 | 4.915317 | 0.251195 | 0.907176 | 4.918052 | 0.207038 |
| H | 3.514179 | 4.737704 | -0.578907 | 3.389088 | 4.747404 | -0.152293 | 3.342439 | 4.772300 | -0.118685 | 3.202697 | 4.863744 | -0.061000 | 3.311379 | 4.799416 | -0.118705 | 3.343180 | 4.782046 | -0.147126 |
| H | 4.520932 | 2.483777 | -0.937464 | 4.442191 | 2.559568 | -0.648511 | 4.413571 | 2.606435 | -0.670979 | 4.321571 | 2.745841 | -0.697858 | 4.393018 | 2.639815 | -0.679329 | 4.411304 | 2.608841 | -0.681377 |
| H | 4.902024 | 0.241824 | -1.846893 | 4.801442 | 0.452285 | -1.831946 | 4.797523 | 0.537659 | -1.829142 | 4.753212 | 0.700414 | -1.836939 | 4.783045 | 0.572314 | -1.830050 | 4.785419 | 0.516314 | -1.840194 |
| H | 4.253219 | -1.365199 | -1.456269 | 4.256153 | -1.201529 | -1.558232 | 4.277743 | -1.133434 | -1.626465 | 4.274734 | -0.987181 | -1.704731 | 4.271978 | -1.102235 | -1.634276 | 4.262448 | -1.148141 | -1.594102 |
| H | 0.424296 | -2.999431 | -0.419025 | 0.483944 | -2.934461 | -0.124096 | 0.508728 | -2.912737 | -0.047300 | 0.571799 | -2.867290 | 0.178711 | 0.523398 | -2.907342 | 0.007296 | 0.504645 | -2.920015 | -0.025084 |
| H | -4.521054 | -2.484508 | -0.936693 | -4.442183 | -2.559401 | -0.648641 | -4.413629 | -2.606734 | -0.670637 | -4.321594 | -2.745852 | -0.697838 | -4.392861 | -2.639708 | -0.679321 | -4.411604 | -2.608653 | -0.681390 |
| H | -4.902172 | -0.243038 | -1.846675 | -4.801424 | -0.452072 | -1.831967 | -4.797451 | -0.538013 | -1.829070 | -4.753222 | -0.700440 | -1.836928 | -4.782843 | -0.572059 | -1.830039 | -4.785362 | -0.516082 | -1.840201 |
| H | -4.253339 | 1.364218 | -1.457089 | -4.256135 | 1.201730 | -1.558186 | -4.277649 | 1.133073 | -1.626394 | -4.274728 | 0.987152 | -1.704755 | -4.271877 | 1.102477 | -1.633896 | -4.262131 | 1.148284 | -1.594068 |
| H | -1.059117 | -4.980269 | -0.300797 | -0.947009 | -4.915786 | 0.135404 | -0.903615 | -4.907907 | 0.219634 | -0.782066 | -4.907339 | 0.402781 | -0.875725 | -4.915243 | 0.250812 | -0.907943 | -4.918407 | 0.207410 |
| H | -3.514313 | -4.738339 | -0.577522 | -3.389091 | -4.747266 | -0.152521 | -3.342577 | -4.772599 | -0.118192 | -3.202740 | -4.863758 | -0.060960 | -3.311155 | -4.799349 | -0.118980 | -3.343894 | -4.782014 | -0.146961 |
| H | 7.520312 | -1.874563 | 2.948351 | 7.935955 | -2.008907 | 2.430238 | 7.910627 | -2.029219 | 2.407548 | 7.874084 | -2.089015 | 2.292041 | 7.924919 | -2.045448 | 2.359725 | 7.916329 | -2.018566 | 2.404282 |
| H | 5.793222 | -1.556970 | 2.629432 | 6.189873 | -1.743977 | 2.348585 | 6.163995 | -1.765442 | 2.323667 | 6.147677 | -1.713446 | 2.284896 | 6.180920 | -1.757401 | 2.297629 | 6.176995 | -1.706586 | 2.345438 |
| H | 6.707800 | -2.683236 | 1.584667 | 7.007547 | -2.689199 | 1.089637 | 6.987453 | -2.695808 | 1.055290 | 6.850840 | -2.695058 | 0.983453 | 6.975612 | -2.697367 | 1.017905 | 6.949054 | -2.688921 | 1.085347 |
| H | 9.307411 | -0.947258 | 1.366669 | 9.500039 | -0.812612 | 0.846820 | 9.477556 | -0.822045 | 0.855701 | 9.456327 | -0.977586 | 0.688820 | 9.488790 | -0.858261 | 0.784974 | 9.490503 | -0.882928 | 0.793048 |
| H | 8.496226 | -1.807341 | 0.028858 | 8.622513 | -1.561503 | -0.495747 | 8.610527 | -1.551482 | -0.504966 | 8.500472 | -1.667012 | -0.632999 | 8.591511 | -1.575758 | -0.562807 | 8.578929 | -1.630027 | -0.528286 |
| H | 8.796440 | -0.053528 | -0.091849 | 8.831323 | 0.197088 | -0.443925 | 8.823265 | 0.208341 | -0.426996 | 8.818355 | 0.078570 | -0.581378 | 8.829374 | 0.181356 | -0.488087 | 8.833818 | 0.124507 | -0.506273 |
| H | 7.960685 | 0.722389 | 2.771091 | 8.249217 | 0.620625 | 2.508643 | 8.231235 | 0.595093 | 2.512472 | 8.348030 | 0.533673 | 2.365332 | 8.283216 | 0.575415 | 2.461038 | 8.297231 | 0.605611 | 2.444430 |
| H | 7.436424 | 1.563237 | 1.290952 | 7.515095 | 1.576965 | 1.217430 | 7.490909 | 1.567791 | 1.235388 | 7.614521 | 1.528766 | 1.101057 | 7.540989 | 1.557928 | 1.192196 | 7.573455 | 1.561888 | 1.146285 |
| H | 6.219605 | 0.954546 | 2.451466 | 6.489742 | 0.776533 | 2.423988 | 6.471261 | 0.750963 | 2.437292 | 6.599697 | 0.786506 | 2.354537 | 6.524609 | 0.756497 | 2.407503 | 6.541815 | 0.807720 | 2.378221 |
| H | -8.495829 | 1.808431 | 0.028362 | -8.622728 | 1.561051 | -0.495673 | -8.609957 | 1.552612 | -0.505289 | -8.500491 | 1.666959 | -0.633042 | -8.591464 | 1.575644 | -0.563068 | -8.578455 | 1.630947 | -0.528127 |
| H | -9.307131 | 0.949088 | 1.366575 | -9.500114 | 0.811923 | 0.846853 | -9.477393 | 0.823720 | 0.855411 | -9.456320 | 0.977588 | 0.688825 | -9.488964 | 0.857986 | 0.784481 | -9.490286 | 0.883713 | 0.792953 |
| H | -8.796452 | 0.054637 | -0.091605 | -8.831204 | -0.197581 | -0.443944 | -8.823493 | -0.207107 | -0.427131 | -8.818345 | -0.078626 | -0.581324 | -8.829276 | -0.181486 | -0.488557 | -8.833743 | -0.123534 | -0.506585 |
| H | -7.519743 | 1.876719 | 2.947703 | -7.936267 | 2.008431 | 2.430333 | -7.909995 | 2.030360 | 2.407223 | -7.874083 | 2.089125 | 2.291972 | -7.925489 | 2.045096 | 2.359597 | -7.915954 | 2.018584 | 2.404583 |
| H | -6.707020 | 2.684539 | 1.583639 | -7.007976 | 2.688969 | 1.089777 | -6.986460 | 2.696383 | 1.054932 | -6.850866 | 2.695121 | 0.983341 | -6.975922 | 2.697170 | 1.018039 | -6.948446 | 2.689054 | 1.085877 |
| H | -5.792741 | 1.558496 | 2.628897 | -6.190136 | 1.743835 | 2.348683 | -6.163477 | 1.765785 | 2.323449 | -6.147669 | 1.713591 | 2.284827 | -6.181468 | 1.757136 | 2.297860 | -6.176687 | 1.706224 | 2.345767 |
| H | -6.219691 | -0.952987 | 2.451951 | -6.489516 | -0.776738 | 2.423938 | -6.471902 | -0.750460 | 2.437360 | -6.599635 | -0.786371 | 2.354594 | -6.525082 | -0.756824 | 2.407431 | -6.542123 | -0.808006 | 2.377921 |
| H | -7.436819 | -1.561865 | 1.291847 | -7.514719 | -1.577298 | 1.217336 | -7.491840 | -1.566969 | 1.235485 | -7.614469 | -1.528712 | 1.101171 | -7.541174 | -1.558169 | 1.191828 | -7.573808 | -1.561629 | 1.145691 |
| H | -7.960664 | -0.720224 | 2.771685 | -8.249020 | -0.621167 | 2.508603 | -8.231811 | -0.593793 | 2.512411 | -8.347973 | -0.533565 | 2.365406 | -8.283702 | -0.575797 | 2.460602 | -8.297497 | -0.605521 | 2.444008 |

| **iso-I** 32 |  | gasphase |  |  | toluene |  |  | DMSO |  |  | methanol |  |  | acetonitrile |  |  | chloroform |  |
| --- | --- | --- | --- | --- | --- | --- | --- | --- | --- | --- | --- | --- | --- | --- | --- | --- | --- | --- |
| Element | x | y | z | x | y | z | x | y | z | x | y | z | x | y | z | x | y | z |
| C | -0.035762 | -2.485261 | -1.036713 | -0.03143 | -2.457862 | -1.058634 | -0.020059 | -2.458933 | -1.227883 | -0.023969 | -2.469147 | -1.447823 | -0.025320 | -2.466289 | -1.264440 | -0.041839 | -2.486091 | -1.178204 |
| C | 0.690286 | -3.629161 | -1.341346 | 0.700954 | -3.596617 | -1.366702 | 0.713279 | -3.592870 | -1.551972 | 0.708275 | -3.599280 | -1.786478 | 0.705380 | -3.601797 | -1.589680 | 0.682555 | -3.628933 | -1.490957 |
| C | 2.066239 | -3.579744 | -1.498735 | 2.080381 | -3.542761 | -1.496632 | 2.096721 | -3.539335 | -1.655961 | 2.094216 | -3.554580 | -1.854239 | 2.089324 | -3.552362 | -1.689810 | 2.064445 | -3.586359 | -1.606087 |
| C | 2.747680 | -2.375674 | -1.382139 | 2.758832 | -2.339764 | -1.351628 | 2.779007 | -2.343128 | -1.470834 | 2.782874 | -2.369837 | -1.620712 | 2.774896 | -2.358578 | -1.499669 | 2.753965 | -2.391368 | -1.442900 |
| C | 2.010168 | -1.246918 | -1.095829 | 2.014114 | -1.215433 | -1.06335 | 2.032953 | -1.223330 | -1.165238 | 2.038751 | -1.254308 | -1.301184 | 2.031709 | -1.237207 | -1.192902 | 2.017235 | -1.263022 | -1.149511 |
| C | 0.621558 | -1.269076 | -0.889710 | 0.622795 | -1.244473 | -0.878881 | 0.639388 | -1.256118 | -1.000957 | 0.642425 | -1.281705 | -1.166779 | 0.637390 | -1.266007 | -1.032384 | 0.624230 | -1.282428 | -0.976665 |
| N | 2.484417 | 0.054166 | -1.005591 | 2.48291 | 0.086078 | -0.947123 | 2.500201 | 0.076079 | -1.011344 | 2.509443 | 0.042739 | -1.104126 | 2.502440 | 0.061056 | -1.034710 | 2.495261 | 0.035104 | -1.018073 |
| C | 1.466955 | 0.936390 | -0.746173 | 1.458648 | 0.961701 | -0.703739 | 1.473639 | 0.943396 | -0.760407 | 1.483948 | 0.898691 | -0.833051 | 1.477817 | 0.929676 | -0.782209 | 1.476916 | 0.913179 | -0.768598 |
| C | 0.191338 | 0.120278 | -0.663223 | 0.185455 | 0.139659 | -0.642983 | 0.200172 | 0.119440 | -0.742781 | 0.207856 | 0.085974 | -0.876965 | 0.201865 | 0.110330 | -0.771099 | 0.197027 | 0.101435 | -0.731699 |
| C | -1.027292 | 0.659157 | -0.405101 | -1.038557 | 0.666176 | -0.387331 | -1.025184 | 0.635093 | -0.480701 | -1.014560 | 0.607257 | -0.615302 | -1.021414 | 0.630806 | -0.507830 | -1.022308 | 0.635456 | -0.473222 |
| O | 1.643489 | 2.120171 | -0.573716 | 1.621635 | 2.147262 | -0.521538 | 1.626327 | 2.122378 | -0.523626 | 1.634645 | 2.065236 | -0.524435 | 1.633584 | 2.107205 | -0.538505 | 1.644524 | 2.094281 | -0.555226 |
| C | -1.533673 | 2.034196 | -0.445643 | -1.557513 | 2.036071 | -0.433993 | -1.542001 | 2.003362 | -0.527743 | -1.510287 | 1.980646 | -0.646995 | -1.531858 | 2.001359 | -0.549360 | -1.525828 | 2.009648 | -0.519666 |
| C | -2.898792 | 2.007333 | -0.119856 | -2.921454 | 1.997109 | -0.105012 | -2.890942 | 1.971513 | -0.145686 | -2.843567 | 1.972739 | -0.217949 | -2.878832 | 1.976000 | -0.159614 | -2.879753 | 1.990255 | -0.152720 |
| N | -3.302662 | 0.684370 | 0.143883 | -3.309617 | 0.672851 | 0.167907 | -3.268051 | 0.652580 | 0.160550 | -3.229788 | 0.654579 | 0.102951 | -3.261322 | 0.657283 | 0.146793 | -3.275262 | 0.672211 | 0.142725 |
| C | -2.200328 | -0.179737 | 0.046344 | -2.203122 | -0.180482 | 0.070481 | -2.177481 | -0.208915 | 0.009413 | -2.166184 | -0.224724 | -0.120055 | -2.175646 | -0.209557 | -0.018587 | -2.186930 | -0.197494 | 0.004242 |
| C | -0.969683 | 3.249752 | -0.823612 | -1.009066 | 3.252732 | -0.83019 | -1.010892 | 3.205115 | -0.987175 | -0.976075 | 3.164155 | -1.147275 | -0.996018 | 3.200460 | -1.010572 | -0.975181 | 3.211287 | -0.956343 |
| C | -1.752959 | 4.392200 | -0.845306 | -1.804254 | 4.386921 | -0.862403 | -1.806260 | 4.339835 | -1.015898 | -1.751646 | 4.311992 | -1.157612 | -1.784674 | 4.340032 | -1.033160 | -1.757298 | 4.354872 | -0.983050 |
| C | -3.093497 | 4.338250 | -0.497751 | -3.143729 | 4.321893 | -0.510635 | -3.130796 | 4.285180 | -0.604640 | -3.059701 | 4.284219 | -0.693506 | -3.106926 | 4.292466 | -0.613424 | -3.086515 | 4.311463 | -0.588545 |
| C | -3.689020 | 3.139205 | -0.132971 | -3.724466 | 3.119791 | -0.131747 | -3.695932 | 3.093198 | -0.171996 | -3.630734 | 3.106844 | -0.228943 | -3.676847 | 3.103170 | -0.179023 | -3.670463 | 3.122091 | -0.174689 |
| O | -2.200075 | -1.330337 | 0.383129 | -2.191564 | -1.329081 | 0.418194 | -2.160433 | -1.361621 | 0.350152 | -2.162873 | -1.394473 | 0.169949 | -2.162461 | -1.366108 | 0.308273 | -2.182794 | -1.351622 | 0.337140 |
| C | 3.855139 | 0.447187 | -1.060124 | 3.853532 | 0.481986 | -1.02199 | 3.876963 | 0.463892 | -1.035806 | 3.888394 | 0.423402 | -1.050671 | 3.880320 | 0.446578 | -1.044803 | 3.872268 | 0.418254 | -1.053910 |
| C | 4.617915 | 0.090896 | 0.210255 | 4.639216 | 0.115008 | 0.230253 | 4.600946 | 0.121625 | 0.258646 | 4.531303 | 0.085520 | 0.283507 | 4.586005 | 0.114731 | 0.262234 | 4.601488 | 0.097644 | 0.243418 |
| O | 5.911618 | 0.323913 | 0.018399 | 5.930854 | 0.303255 | 0.001619 | 5.897795 | 0.314594 | 0.095605 | 5.818747 | 0.354081 | 0.234367 | 5.882629 | 0.323199 | 0.122850 | 5.894960 | 0.322330 | 0.082356 |
| O | 4.100914 | -0.321921 | 1.208959 | 4.134453 | -0.267086 | 1.249443 | 4.043963 | -0.247666 | 1.259174 | 3.920058 | -0.352512 | 1.227782 | 4.016398 | -0.262842 | 1.252927 | 4.053875 | -0.286878 | 1.241699 |
| C | 6.902395 | 0.120647 | 1.076699 | 6.951352 | 0.088605 | 1.033855 | 6.874835 | 0.109905 | 1.175120 | 6.729345 | 0.164260 | 1.379579 | 6.844580 | 0.123505 | 1.217125 | 6.872035 | 0.139353 | 1.164471 |
| C | 6.938903 | -1.348042 | 1.477642 | 6.955779 | -1.371897 | 1.461027 | 6.865292 | -1.346884 | 1.611076 | 6.779734 | -1.307627 | 1.755544 | 6.845351 | -1.336777 | 1.642153 | 6.903594 | -1.321787 | 1.586720 |
| C | 8.204795 | 0.526978 | 0.404235 | 8.241937 | 0.441174 | 0.312045 | 8.194759 | 0.460344 | 0.509389 | 8.064649 | 0.622269 | 0.818612 | 8.169972 | 0.495331 | 0.573668 | 8.182330 | 0.537053 | 0.504969 |
| C | 6.598561 | 1.033754 | 2.256763 | 6.713329 | 1.033587 | 2.202271 | 6.585598 | 1.063003 | 2.324054 | 6.296960 | 1.054037 | 2.533569 | 6.524410 | 1.065194 | 2.367590 | 6.547417 | 1.072987 | 2.320722 |
| C | -4.599784 | 0.320267 | 0.544591 | -4.600084 | 0.29745 | 0.586545 | -4.530888 | 0.293757 | 0.677289 | -4.461521 | 0.320226 | 0.694245 | -4.523343 | 0.304454 | 0.666300 | -4.550728 | 0.321346 | 0.625286 |
| O | -4.839159 | -0.933190 | 0.229803 | -4.858764 | -0.932834 | 0.211224 | -4.862754 | -0.905708 | 0.271565 | -4.797417 | -0.908042 | 0.403168 | -4.840089 | -0.914114 | 0.308638 | -4.847807 | -0.907423 | 0.280944 |
| O | -5.353618 | 1.098076 | 1.065447 | -5.328306 | 1.056396 | 1.168843 | -5.171644 | 1.048310 | 1.360877 | -5.084220 | 1.118531 | 1.349331 | -5.178837 | 1.078225 | 1.314332 | -5.236519 | 1.101085 | 1.232960 |
| C | -6.027772 | -1.637009 | 0.711782 | -6.052113 | -1.65396 | 0.67065 | -6.052191 | -1.607536 | 0.786630 | -5.970458 | -1.578307 | 1.004485 | -6.031767 | -1.602900 | 0.835885 | -6.039694 | -1.598798 | 0.796604 |
| C | -6.062332 | -1.610048 | 2.233393 | -6.06271 | -1.71802 | 2.190136 | -5.953445 | -1.747966 | 2.296530 | -5.834407 | -1.592153 | 2.517427 | -5.953812 | -1.679793 | 2.351945 | -5.991226 | -1.642649 | 2.315611 |
| C | -5.796847 | -3.051884 | 0.203852 | -5.848376 | -3.037537 | 0.075331 | -5.952376 | -2.964692 | 0.112412 | -5.859450 | -2.983519 | 0.439316 | -5.910810 | -2.986244 | 0.219961 | -5.886551 | -2.992825 | 0.211564 |
| C | -7.279592 | -1.033116 | 0.091066 | -7.303305 | -1.00012 | 0.104568 | -7.311063 | -0.878235 | 0.347439 | -7.243603 | -0.900443 | 0.528066 | -7.289246 | -0.902224 | 0.347691 | -7.296173 | -0.924673 | 0.268598 |
| H | -1.099955 | -2.551713 | -0.895121 | -1.099146 | -2.527099 | -0.944375 | -1.091848 | -2.522690 | -1.144857 | -1.099270 | -2.522293 | -1.402152 | -1.097576 | -2.526422 | -1.184246 | -1.112301 | -2.544208 | -1.080212 |
| H | 0.170540 | -4.572516 | -1.443032 | 0.184414 | -4.538926 | -1.494049 | 0.196646 | -4.529393 | -1.718227 | 0.188754 | -4.525913 | -1.994188 | 0.186201 | -4.536236 | -1.759593 | 0.158621 | -4.564832 | -1.635465 |
| H | 2.618410 | -4.484958 | -1.717295 | 2.63785 | -4.444273 | -1.718052 | 2.654755 | -4.436360 | -1.894337 | 2.650517 | -4.449564 | -2.104227 | 2.645251 | -4.450488 | -1.928905 | 2.615490 | -4.490682 | -1.832583 |
| H | 3.820941 | -2.328570 | -1.510192 | 3.834274 | -2.290845 | -1.461581 | 3.855408 | -2.293765 | -1.571549 | 3.861575 | -2.328003 | -1.695311 | 3.851657 | -2.312092 | -1.596967 | 3.830359 | -2.350842 | -1.546122 |
| H | 4.355919 | 0.000986 | -1.921282 | 4.335209 | 0.042414 | -1.896925 | 4.388530 | -0.006813 | -1.875788 | 4.447620 | -0.059061 | -1.852664 | 4.402713 | -0.033564 | -1.872560 | 4.382120 | -0.068380 | -1.886381 |
| H | 3.888986 | 1.530817 | -1.179951 | 3.888507 | 1.566501 | -1.132585 | 3.931247 | 1.543772 | -1.176451 | 3.964621 | 1.500644 | -1.200232 | 3.938869 | 1.524865 | -1.195682 | 3.927707 | 1.495457 | -1.214250 |
| H | 0.074387 | 3.301318 | -1.076677 | 0.032687 | 3.312231 | -1.09231 | 0.015723 | 3.255350 | -1.307682 | 0.032433 | 3.190765 | -1.525479 | 0.028758 | 3.244505 | -1.338052 | 0.057191 | 3.253814 | -1.257736 |
| H | -4.729744 | 3.089293 | 0.142612 | -4.766242 | 3.064433 | 0.140517 | -4.730840 | 3.048082 | 0.129323 | -4.657799 | 3.084266 | 0.099630 | -4.709634 | 3.063273 | 0.129491 | -4.706426 | 3.084775 | 0.122460 |
| H | -1.305594 | 5.335427 | -1.129903 | -1.368648 | 5.331788 | -1.160344 | -1.387161 | 5.275217 | -1.363883 | -1.332734 | 5.235258 | -1.536801 | -1.362224 | 5.273303 | -1.382728 | -1.321424 | 5.289260 | -1.312365 |
| H | -3.691947 | 5.240453 | -0.508905 | -3.752733 | 5.217006 | -0.53165 | -3.741317 | 5.179279 | -0.628266 | -3.656082 | 5.188055 | -0.706031 | -3.712284 | 5.190190 | -0.631738 | -3.685700 | 5.213311 | -0.606797 |
| H | 7.771897 | -1.510281 | 2.163481 | 7.813439 | -1.551199 | 2.111655 | 7.697749 | -1.515042 | 2.296512 | 7.574632 | -1.454873 | 2.488741 | 7.666663 | -1.499346 | 2.342188 | 7.730114 | -1.472514 | 2.283303 |
| H | 6.017980 | -1.648916 | 1.971511 | 6.050658 | -1.63478 | 2.004144 | 5.940577 | -1.609257 | 2.121096 | 5.843798 | -1.650160 | 2.193055 | 5.914270 | -1.614229 | 2.132431 | 5.980220 | -1.618773 | 2.079810 |
| H | 7.095485 | -1.977063 | 0.599602 | 7.049855 | -2.024227 | 0.590638 | 6.995980 | -2.004483 | 0.749425 | 7.007458 | -1.917053 | 0.878927 | 7.000834 | -1.985322 | 0.777819 | 7.068099 | -1.965860 | 0.720642 |
| H | 8.400198 | -0.100381 | -0.466195 | 8.394701 | -0.21128 | -0.549084 | 8.386454 | -0.197622 | -0.339899 | 8.351623 | 0.012980 | -0.040249 | 8.381069 | -0.153630 | -0.277947 | 8.394217 | -0.104726 | -0.351824 |
| H | 9.032955 | 0.415802 | 1.104980 | 9.088555 | 0.32218 | 0.989397 | 9.007723 | 0.343966 | 1.227166 | 8.835046 | 0.525621 | 1.584471 | 8.973652 | 0.382794 | 1.302390 | 8.999301 | 0.438399 | 1.220896 |
| H | 8.161346 | 1.567334 | 0.080336 | 8.22039 | 1.475282 | -0.03498 | 8.188400 | 1.493707 | 0.158605 | 8.014305 | 1.667254 | 0.507752 | 8.155331 | 1.531426 | 0.231269 | 8.144106 | 1.573043 | 0.164524 |
| H | 7.419812 | 0.975784 | 2.972846 | 7.557408 | 0.968634 | 2.891067 | 7.404727 | 1.006559 | 3.043006 | 7.070796 | 1.030299 | 3.302735 | 7.333725 | 1.015722 | 3.098037 | 7.366091 | 1.041290 | 3.041772 |
| H | 5.678441 | 0.744316 | 2.758873 | 5.805451 | 0.780911 | 2.745818 | 5.659802 | 0.809114 | 2.836525 | 5.361689 | 0.720323 | 2.979258 | 5.594751 | 0.794377 | 2.864206 | 5.629803 | 0.783292 | 2.828490 |
| H | 6.511336 | 2.068818 | 1.922753 | 6.641152 | 2.064614 | 1.851185 | 6.522055 | 2.090746 | 1.961221 | 6.182567 | 2.085973 | 2.196527 | 6.451337 | 2.093830 | 2.009311 | 6.447571 | 2.100118 | 1.964860 |
| H | -6.867251 | -2.258700 | 2.581956 | -6.861226 | -2.388847 | 2.511147 | -6.754878 | -2.404275 | 2.639270 | -6.609065 | -2.240768 | 2.929497 | -6.755733 | -2.327246 | 2.709989 | -6.791615 | -2.291437 | 2.674937 |
| H | -6.239531 | -0.605561 | 2.611818 | -6.237847 | -0.740763 | 2.635632 | -6.057289 | -0.789310 | 2.801309 | -5.954570 | -0.600508 | 2.949867 | -6.070655 | -0.701539 | 2.814547 | -6.127397 | -0.655821 | 2.754099 |
| H | -5.120889 | -1.984671 | 2.637691 | -5.115503 | -2.113942 | 2.560202 | -4.999756 | -2.198972 | 2.576780 | -4.861973 | -1.993844 | 2.808017 | -5.001001 | -2.111974 | 2.663164 | -5.039086 | -2.055457 | 2.653541 |
| H | -5.734223 | -3.062013 | -0.884776 | -5.79062 | -2.984312 | -1.012822 | -5.970724 | -2.858885 | -0.973481 | -5.903793 | -2.965074 | -0.650917 | -5.914083 | -2.924768 | -0.869480 | -5.871915 | -2.953415 | -0.878776 |
| H | -6.623070 | -3.693938 | 0.511241 | -6.686115 | -3.681535 | 0.34586 | -6.799071 | -3.582458 | 0.413829 | -6.686321 | -3.590325 | 0.809701 | -6.756539 | -3.598584 | 0.534776 | -6.725729 | -3.615482 | 0.524080 |
| H | -4.867286 | -3.452529 | 0.608085 | -4.927817 | -3.485576 | 0.450619 | -5.031058 | -3.472612 | 0.400891 | -4.921225 | -3.448576 | 0.746131 | -4.989243 | -3.472651 | 0.542687 | -4.960932 | -3.454940 | 0.556984 |
| H | -7.456474 | -0.024367 | 0.456892 | -7.472236 | -0.01598 | 0.536227 | -7.409838 | 0.087252 | 0.839507 | -7.356750 | 0.097215 | 0.948289 | -7.402582 | 0.081689 | 0.798523 | -7.432124 | 0.065831 | 0.697894 |
| H | -8.139232 | -1.655037 | 0.346046 | -8.166051 | -1.62979 | 0.329032 | -8.177913 | -1.488834 | 0.605580 | -8.097813 | -1.503235 | 0.841004 | -8.155337 | -1.508935 | 0.617207 | -8.161549 | -1.536647 | 0.528890 |
| H | -7.188689 | -1.007514 | -0.995936 | -7.226762 | -0.904043 | -0.980028 | -7.309411 | -0.729484 | -0.733967 | -7.254555 | -0.832819 | -0.561267 | -7.270827 | -0.797974 | -0.738757 | -7.254849 | -0.839339 | -0.818837 |

| **iso-I** 33 |  | gasphase |  |  | toluene |  |  | DMSO |  |  | methanol |  |  | acetonitrile |  |  | chloroform |  |
| --- | --- | --- | --- | --- | --- | --- | --- | --- | --- | --- | --- | --- | --- | --- | --- | --- | --- | --- |
| Element | x | y | z | x | y | z | x | y | z | x | y | z | x | y | z | x | y | z |
| C | 0.909290 | -2.530495 | -1.520379 | 0.893068 | -2.500909 | -1.517530 | 0.887361 | -2.453614 | -1.566497 | 0.899017 | -2.396164 | -1.722068 | 0.877277 | -2.476051 | -1.585469 | 0.881212 | -2.537811 | -1.490703 |
| C | 1.692322 | -3.654804 | -1.750489 | 1.671707 | -3.621903 | -1.776195 | 1.662690 | -3.567081 | -1.865639 | 1.675601 | -3.499332 | -2.052878 | 1.646428 | -3.597369 | -1.871578 | 1.653125 | -3.663959 | -1.748331 |
| C | 3.074801 | -3.583308 | -1.701604 | 3.055281 | -3.554358 | -1.730036 | 3.048419 | -3.499410 | -1.830843 | 3.060973 | -3.436804 | -1.996570 | 3.032428 | -3.539831 | -1.826090 | 3.038124 | -3.600313 | -1.717578 |
| C | 3.712041 | -2.376021 | -1.444281 | 3.697153 | -2.354536 | -1.449691 | 3.695063 | -2.308084 | -1.523958 | 3.707718 | -2.258972 | -1.640093 | 3.685812 | -2.350930 | -1.523045 | 3.687848 | -2.400228 | -1.455047 |
| C | 2.921969 | -1.267636 | -1.231601 | 2.910088 | -1.248504 | -1.210542 | 2.909852 | -1.209253 | -1.243182 | 2.921297 | -1.170960 | -1.327530 | 2.906897 | -1.244169 | -1.256282 | 2.906875 | -1.289714 | -1.215774 |
| C | 1.518076 | -1.313094 | -1.236921 | 1.506371 | -1.292237 | -1.208069 | 1.506682 | -1.256930 | -1.223847 | 1.519153 | -1.218347 | -1.321739 | 1.503311 | -1.281997 | -1.245499 | 1.503313 | -1.329706 | -1.196364 |
| N | 3.356423 | 0.033473 | -1.019700 | 3.347167 | 0.047654 | -0.973687 | 3.347033 | 0.081690 | -0.974724 | 3.354697 | 0.115903 | -1.013254 | 3.350914 | 0.047029 | -0.997046 | 3.350286 | 0.008456 | -0.995324 |
| C | 2.297974 | 0.892331 | -0.875553 | 2.291809 | 0.904986 | -0.816016 | 2.292331 | 0.928919 | -0.778403 | 2.297422 | 0.946208 | -0.788226 | 2.300322 | 0.901915 | -0.813723 | 2.299241 | 0.866154 | -0.825329 |
| C | 1.035392 | 0.057947 | -1.011627 | 1.027770 | 0.075498 | -0.960906 | 1.029751 | 0.102358 | -0.942380 | 1.040901 | 0.128344 | -0.996952 | 1.033656 | 0.081914 | -0.974682 | 1.032244 | 0.041366 | -0.958604 |
| C | -0.210381 | 0.577621 | -0.874132 | -0.218655 | 0.591378 | -0.817718 | -0.217207 | 0.608618 | -0.786670 | -0.206040 | 0.628275 | -0.830472 | -0.210396 | 0.595485 | -0.819179 | -0.210652 | 0.563732 | -0.816666 |
| O | 2.425933 | 2.070122 | -0.635705 | 2.417740 | 2.079612 | -0.553518 | 2.409709 | 2.091981 | -0.458208 | 2.404135 | 2.094807 | -0.403183 | 2.423607 | 2.067611 | -0.504150 | 2.426368 | 2.040125 | -0.553956 |
| C | -0.699938 | 1.963713 | -0.849651 | -0.713347 | 1.974772 | -0.810283 | -0.716260 | 1.987996 | -0.790723 | -0.707022 | 2.004802 | -0.821427 | -0.702774 | 1.977216 | -0.827177 | -0.698377 | 1.948675 | -0.838924 |
| C | -2.076566 | 1.922529 | -0.580281 | -2.092172 | 1.930146 | -0.552516 | -2.092657 | 1.939821 | -0.524153 | -2.079920 | 1.951051 | -0.544048 | -2.077604 | 1.937258 | -0.550945 | -2.074640 | 1.916383 | -0.568079 |
| N | -2.490788 | 0.598901 | -0.412339 | -2.500380 | 0.606905 | -0.370598 | -2.492045 | 0.616707 | -0.324863 | -2.471448 | 0.621911 | -0.347694 | -2.482043 | 0.616633 | -0.342078 | -2.486176 | 0.599020 | -0.352254 |
| C | -1.438030 | -0.273500 | -0.625188 | -1.441183 | -0.260799 | -0.549392 | -1.431077 | -0.245926 | -0.489650 | -1.411021 | -0.229699 | -0.526126 | -1.426646 | -0.251466 | -0.511797 | -1.433721 | -0.275647 | -0.516267 |
| C | -0.129338 | 3.197749 | -1.142439 | -0.145386 | 3.207416 | -1.114214 | -0.154618 | 3.215868 | -1.124206 | -0.151182 | 3.231888 | -1.165904 | -0.136987 | 3.200317 | -1.171423 | -0.127999 | 3.169219 | -1.184482 |
| C | -0.915978 | 4.341200 | -1.128216 | -0.936134 | 4.348050 | -1.118442 | -0.949569 | 4.353891 | -1.137730 | -0.949846 | 4.367103 | -1.170470 | -0.926281 | 4.342449 | -1.185789 | -0.913739 | 4.313327 | -1.210009 |
| C | -2.269154 | 4.270067 | -0.838727 | -2.291416 | 4.274206 | -0.836046 | -2.302454 | 4.278653 | -0.836138 | -2.299305 | 4.287369 | -0.853874 | -2.277591 | 4.275619 | -0.874671 | -2.266345 | 4.253214 | -0.907892 |
| C | -2.872339 | 3.048987 | -0.568436 | -2.892355 | 3.053787 | -0.557173 | -2.897565 | 3.060299 | -0.534561 | -2.889262 | 3.067202 | -0.547741 | -2.876885 | 3.061779 | -0.562363 | -2.870184 | 3.043115 | -0.591281 |
| O | -1.539917 | -1.472260 | -0.543275 | -1.528342 | -1.458163 | -0.421683 | -1.499965 | -1.441291 | -0.318146 | -1.466609 | -1.429812 | -0.350277 | -1.500314 | -1.446312 | -0.336892 | -1.520848 | -1.470068 | -0.351208 |
| C | 4.713745 | 0.441265 | -0.851973 | 4.711490 | 0.448692 | -0.838085 | 4.715521 | 0.472781 | -0.831308 | 4.719359 | 0.497947 | -0.811529 | 4.720152 | 0.433613 | -0.845511 | 4.717767 | 0.404312 | -0.864153 |
| C | 5.280939 | 0.038622 | 0.504235 | 5.308641 | 0.045714 | 0.504254 | 5.306671 | 0.039272 | 0.502765 | 5.259857 | 0.024043 | 0.526920 | 5.290948 | 0.031068 | 0.506670 | 5.305826 | 0.030716 | 0.489125 |
| O | 6.587740 | 0.275064 | 0.515930 | 6.618288 | 0.245053 | 0.473940 | 6.613270 | 0.234306 | 0.484296 | 6.557590 | 0.236138 | 0.580988 | 6.594194 | 0.244853 | 0.511328 | 6.606250 | 0.271941 | 0.480182 |
| O | 4.621499 | -0.407493 | 1.399486 | 4.662308 | -0.368269 | 1.426516 | 4.652258 | -0.393485 | 1.414995 | 4.569740 | -0.459370 | 1.391378 | 4.625276 | -0.398159 | 1.412862 | 4.657698 | -0.402131 | 1.403960 |
| C | 7.410201 | 0.028557 | 1.702008 | 7.478788 | -0.008062 | 1.635429 | 7.477084 | -0.055230 | 1.638389 | 7.383588 | -0.094646 | 1.757939 | 7.440935 | -0.016888 | 1.684830 | 7.460373 | 0.053091 | 1.655795 |
| C | 7.389929 | -1.454720 | 2.046346 | 7.429545 | -1.483958 | 2.002543 | 7.424247 | -1.540055 | 1.962414 | 7.345477 | -1.594144 | 2.004034 | 7.405747 | -1.498986 | 2.024251 | 7.459573 | -1.422653 | 2.025268 |
| C | 8.797163 | 0.456723 | 1.247790 | 8.858500 | 0.372693 | 1.123349 | 8.856990 | 0.333754 | 1.135629 | 8.770812 | 0.343596 | 1.320884 | 8.822987 | 0.389752 | 1.201484 | 8.831423 | 0.482600 | 1.160091 |
| C | 6.930479 | 0.896017 | 2.857839 | 7.067948 | 0.891077 | 2.791981 | 7.073767 | 0.812763 | 2.819925 | 6.917681 | 0.712423 | 2.958626 | 7.001244 | 0.858546 | 2.848105 | 7.000573 | 0.941082 | 2.802071 |
| C | -3.783513 | 0.183645 | -0.021632 | -3.798919 | 0.189110 | 0.003590 | -3.794596 | 0.198275 | 0.039302 | -3.766476 | 0.194537 | 0.037115 | -3.783332 | 0.204627 | 0.034631 | -3.788816 | 0.195085 | 0.026353 |
| C | -4.370479 | 0.734449 | 1.111819 | -4.390579 | 0.716877 | 1.143818 | -4.380819 | 0.697867 | 1.192193 | -4.337651 | 0.691809 | 1.198128 | -4.360713 | 0.715800 | 1.187029 | -4.369660 | 0.726846 | 1.168394 |
| C | -5.635670 | 0.335479 | 1.496611 | -5.660547 | 0.312348 | 1.511230 | -5.659280 | 0.297519 | 1.544077 | -5.607512 | 0.282200 | 1.569548 | -5.637417 | 0.320724 | 1.551660 | -5.646778 | 0.339828 | 1.536185 |
| C | -6.311835 | -0.628817 | 0.761810 | -6.330800 | -0.634300 | 0.749936 | -6.337010 | -0.610946 | 0.747236 | -6.290480 | -0.632656 | 0.784401 | -6.322260 | -0.594372 | 0.768405 | -6.330753 | -0.591208 | 0.770145 |
| C | -5.720823 | -1.187836 | -0.359948 | -5.736242 | -1.167382 | -0.384450 | -5.744935 | -1.118572 | -0.403374 | -5.712052 | -1.138177 | -0.373864 | -5.738897 | -1.113682 | -0.381590 | -5.744932 | -1.129917 | -0.368390 |
| C | -4.458978 | -0.780020 | -0.755610 | -4.472195 | -0.752612 | -0.761626 | -4.475686 | -0.709167 | -0.762407 | -4.451517 | -0.719667 | -0.753053 | -4.471521 | -0.709620 | -0.753557 | -4.475868 | -0.733513 | -0.744554 |
| C | -7.703777 | -1.024412 | 1.174028 | -7.726377 | -1.045635 | 1.127877 | -7.720196 | -1.074572 | 1.103634 | -7.666127 | -1.104172 | 1.161119 | -7.704432 | -1.051619 | 1.138142 | -7.730186 | -1.002205 | 1.132488 |
| F | -8.608485 | -0.108102 | 0.787957 | -8.650204 | -0.249585 | 0.556447 | -8.598295 | -0.842098 | 0.110526 | -8.562074 | -0.863444 | 0.185956 | -8.591621 | -0.811221 | 0.154746 | -8.642791 | -0.419279 | 0.330941 |
| F | -7.816730 | -1.138428 | 2.504901 | -7.934425 | -0.983041 | 2.450382 | -8.206782 | -0.476514 | 2.197049 | -8.134668 | -0.518383 | 2.268927 | -8.176358 | -0.454012 | 2.238252 | -8.057225 | -0.673027 | 2.388277 |
| F | -8.080235 | -2.192282 | 0.641378 | -8.009993 | -2.297053 | 0.743313 | -7.759828 | -2.399934 | 1.336317 | -7.697714 | -2.431654 | 1.380671 | -7.749104 | -2.377337 | 1.367156 | -7.914737 | -2.326466 | 1.013755 |
| H | -0.163122 | -2.607267 | -1.541047 | -0.179947 | -2.575036 | -1.538493 | -0.186660 | -2.524818 | -1.587265 | -0.175225 | -2.461560 | -1.771189 | -0.197167 | -2.538692 | -1.614906 | -0.192709 | -2.607608 | -1.503312 |
| H | 1.209267 | -4.599917 | -1.959704 | 1.185669 | -4.561165 | -2.005567 | 1.175239 | -4.498923 | -2.122165 | 1.189929 | -4.419030 | -2.352931 | 1.153971 | -4.527142 | -2.125962 | 1.161735 | -4.603477 | -1.965683 |
| H | 3.668536 | -4.473268 | -1.867639 | 3.646230 | -4.442097 | -1.917860 | 3.637598 | -4.380469 | -2.053363 | 3.650659 | -4.310873 | -2.244029 | 3.616674 | -4.426821 | -2.037738 | 3.624025 | -4.491573 | -1.905031 |
| H | 4.791505 | -2.312620 | -1.409328 | 4.777196 | -2.293952 | -1.419716 | 4.775539 | -2.246001 | -1.512512 | 4.788155 | -2.200037 | -1.616194 | 4.766509 | -2.296735 | -1.503687 | 4.768378 | -2.342042 | -1.440225 |
| H | 5.344688 | 0.036296 | -1.645134 | 5.316610 | 0.034744 | -1.646087 | 5.314904 | 0.066129 | -1.646497 | 5.347976 | 0.110664 | -1.613976 | 5.328409 | 0.001227 | -1.640631 | 5.322083 | -0.037956 | -1.657260 |
| H | 4.748338 | 1.529303 | -0.921375 | 4.756716 | 1.535971 | -0.912046 | 4.772688 | 1.560218 | -0.887668 | 4.787989 | 1.585970 | -0.836204 | 4.785948 | 1.518720 | -0.929586 | 4.775287 | 1.488231 | -0.968282 |
| H | 0.921136 | 3.268264 | -1.362370 | 0.906261 | 3.279601 | -1.329852 | 0.893266 | 3.287950 | -1.360853 | 0.891524 | 3.307293 | -1.426878 | 0.909585 | 3.265188 | -1.416248 | 0.920737 | 3.230947 | -1.418929 |
| H | -3.933519 | 2.981853 | -0.373430 | -3.954290 | 2.986292 | -0.364997 | -3.957074 | 2.993368 | -0.328158 | -3.947332 | 2.996699 | -0.334718 | -3.935257 | 3.001213 | -0.348524 | -3.930055 | 2.985837 | -0.384230 |
| H | -0.461093 | 5.298122 | -1.346830 | -0.483878 | 5.304767 | -1.344856 | -0.505398 | 5.308915 | -1.387453 | -0.512482 | 5.323355 | -1.427492 | -0.479054 | 5.293803 | -1.443842 | -0.460654 | 5.261599 | -1.468640 |
| H | -2.869769 | 5.170780 | -0.832750 | -2.895193 | 5.173078 | -0.842119 | -2.909266 | 5.175552 | -0.847330 | -2.908675 | 5.182645 | -0.859814 | -2.880010 | 5.175450 | -0.886838 | -2.865826 | 5.154802 | -0.929142 |
| H | 8.109361 | -1.645981 | 2.844061 | 8.179263 | -1.686084 | 2.769528 | 8.181299 | -1.763375 | 2.716108 | 8.084900 | -1.842674 | 2.767270 | 8.149853 | -1.701293 | 2.796509 | 8.207101 | -1.594432 | 2.801714 |
| H | 6.405393 | -1.771786 | 2.382244 | 6.454093 | -1.769401 | 2.390547 | 6.451873 | -1.833459 | 2.353026 | 6.369516 | -1.926560 | 2.353018 | 6.429900 | -1.804096 | 2.397019 | 6.490737 | -1.742921 | 2.403208 |
| H | 7.680157 | -2.049522 | 1.178541 | 7.660852 | -2.101929 | 1.132701 | 7.644798 | -2.133219 | 1.072759 | 7.601396 | -2.136597 | 1.091903 | 7.655083 | -2.097155 | 1.145663 | 7.723491 | -2.032931 | 1.159286 |
| H | 9.511880 | 0.315098 | 2.059166 | 9.599238 | 0.223040 | 1.909913 | 9.594278 | 0.158351 | 1.920016 | 9.485438 | 0.144033 | 2.120185 | 9.549468 | 0.234423 | 2.000022 | 9.565686 | 0.361521 | 1.957528 |
| H | 9.121691 | -0.136497 | 0.392134 | 9.136609 | -0.242518 | 0.266157 | 9.132349 | -0.260633 | 0.262784 | 9.085277 | -0.203351 | 0.430291 | 9.121994 | -0.209126 | 0.339587 | 9.142291 | -0.125397 | 0.308859 |
| H | 8.799962 | 1.509200 | 0.962422 | 8.882893 | 1.420764 | 0.821156 | 8.885678 | 1.390348 | 0.864071 | 8.786262 | 1.412603 | 1.101397 | 8.838077 | 1.443930 | 0.919571 | 8.820894 | 1.530422 | 0.855716 |
[truncated: 192,162 more chars]
