# Supplementary figures and images for: Predicting the substituent effects in the optical and electrochemical properties of N,N′-substituted isoindigos
Source: Photochem Photobiol Sci. 2021 Jul 5;20(7):927–38. doi: 10.1007/s43630-021-00071-5 (PMC8550769; doi:10.1007/s43630-021-00071-5)

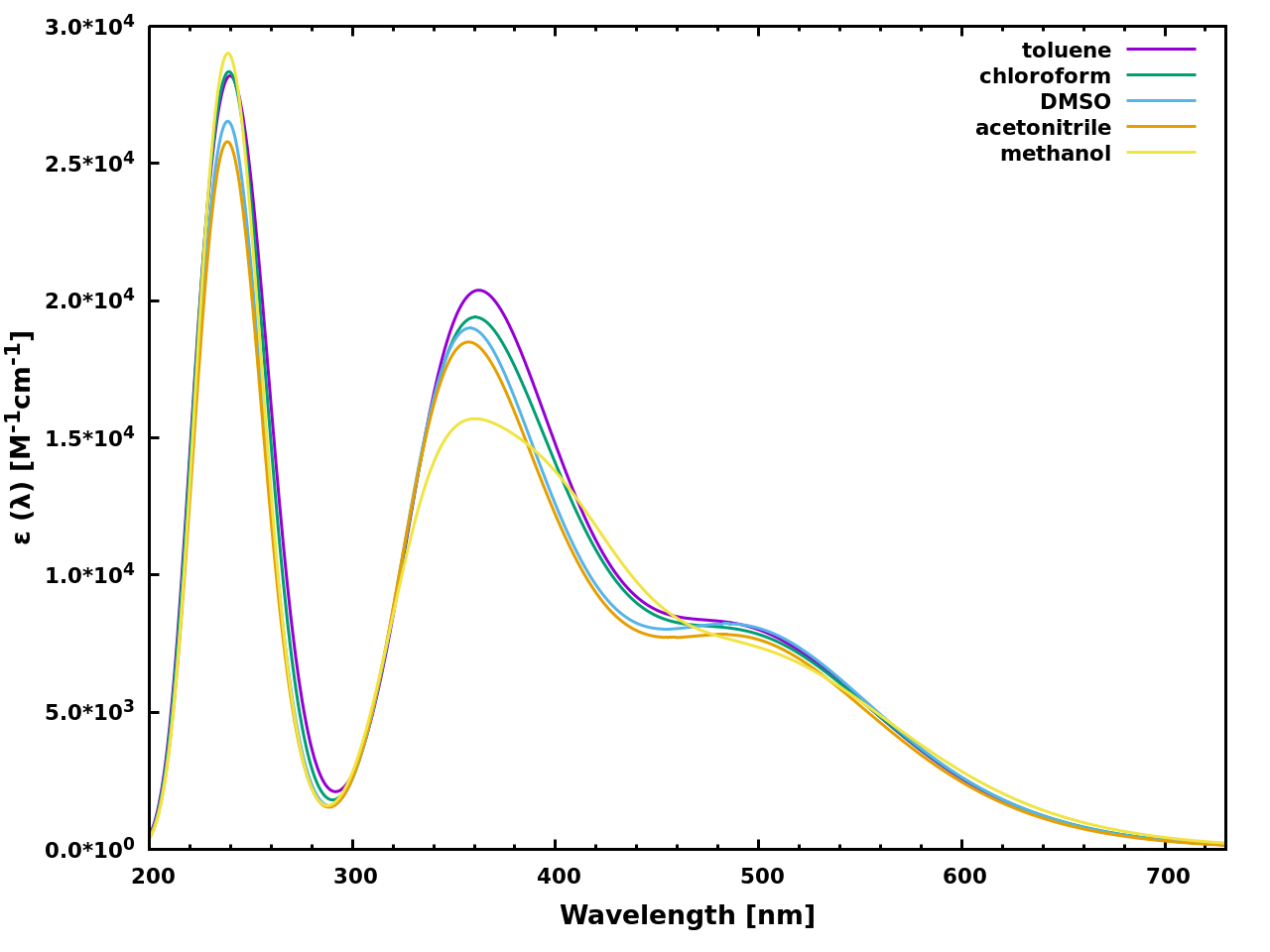

Supplement: Supplementary file 2 — Supplementary file2 (ZIP 5602 KB) [file 43630_2021_71_MOESM2_ESM.zip › simulated_spectra/1.png]

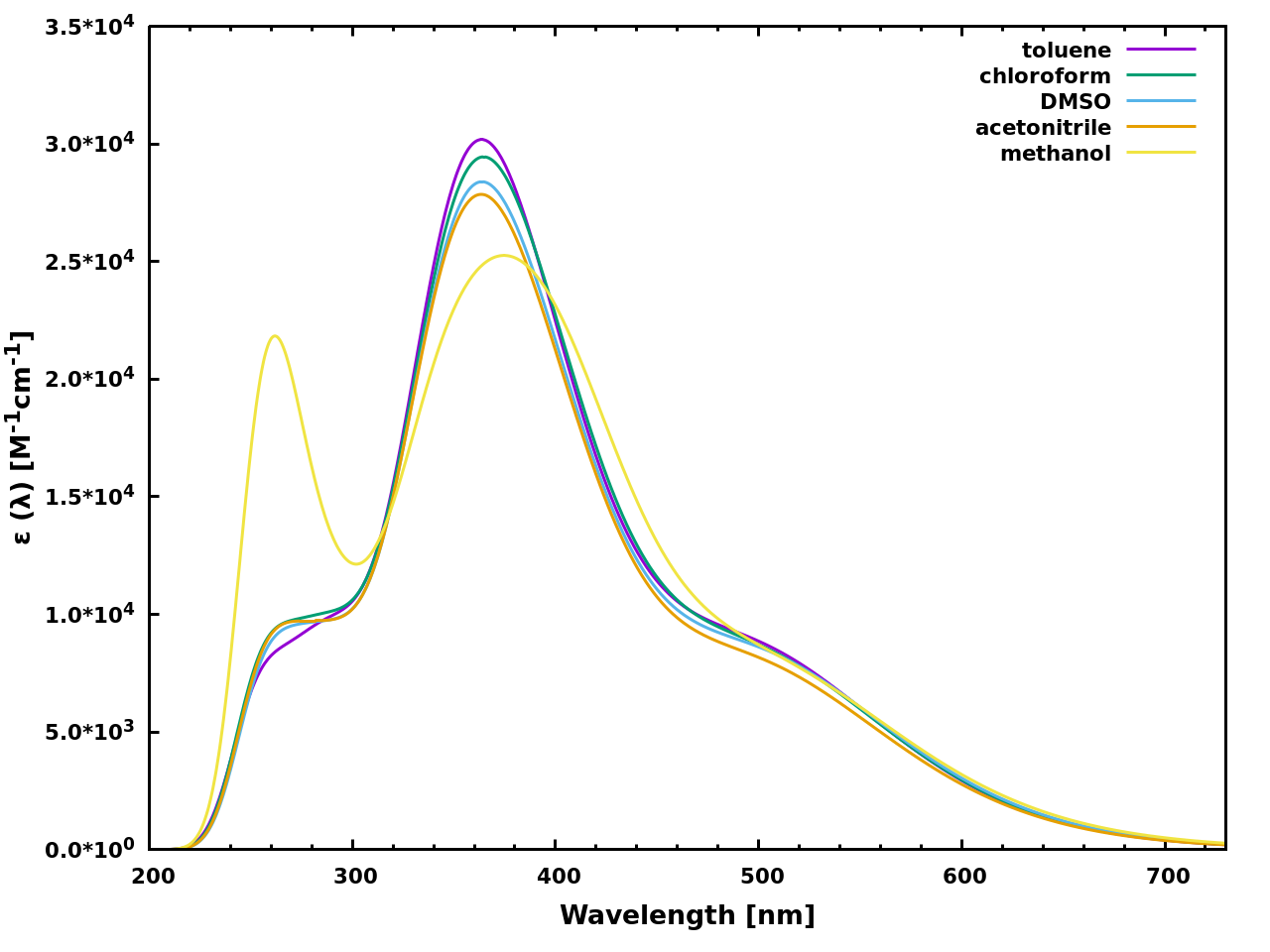

Supplement: Supplementary file 2 — Supplementary file2 (ZIP 5602 KB) [file 43630_2021_71_MOESM2_ESM.zip › simulated_spectra/10.png]

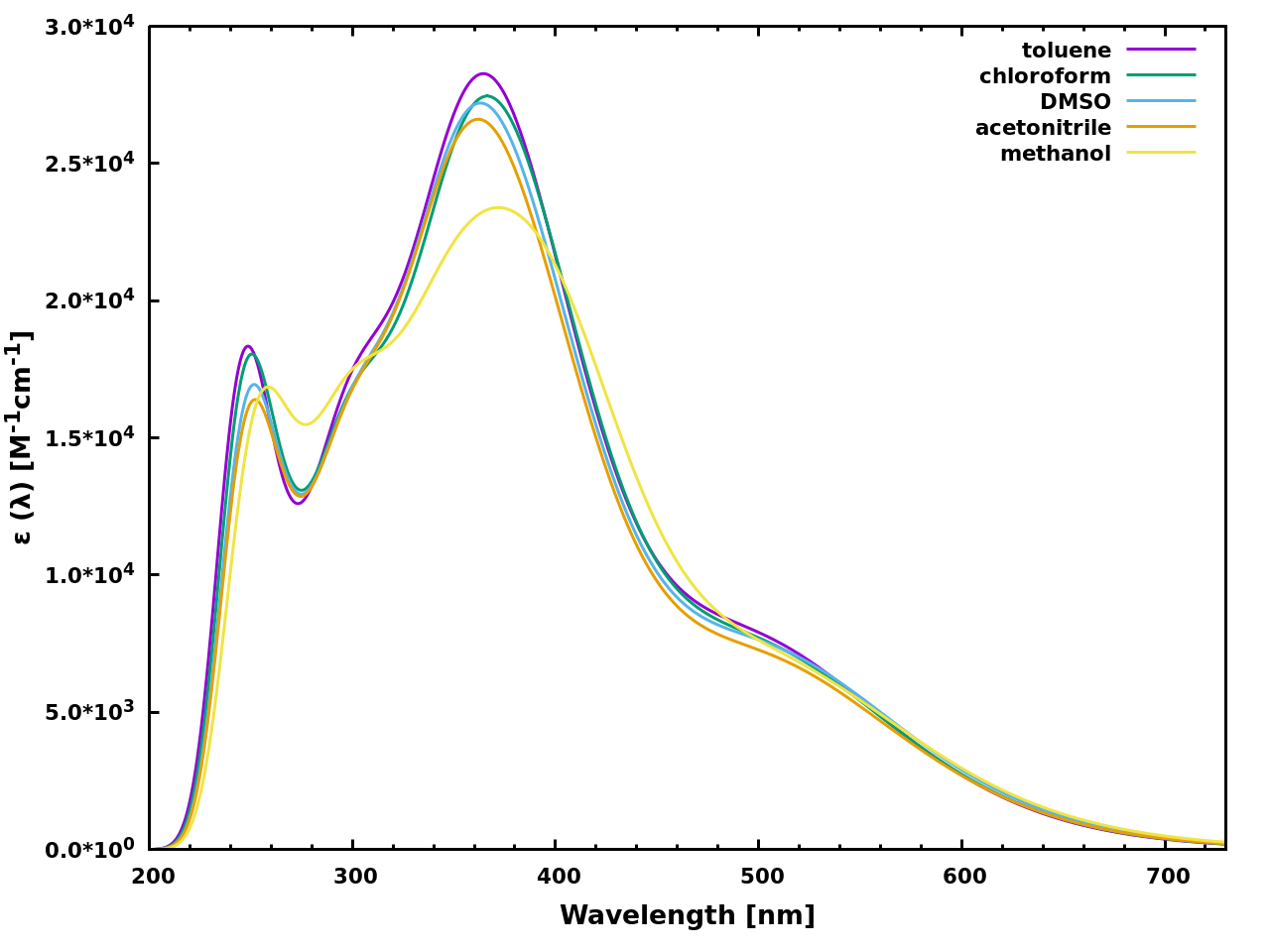

Supplement: Supplementary file 2 — Supplementary file2 (ZIP 5602 KB) [file 43630_2021_71_MOESM2_ESM.zip › simulated_spectra/11.png]

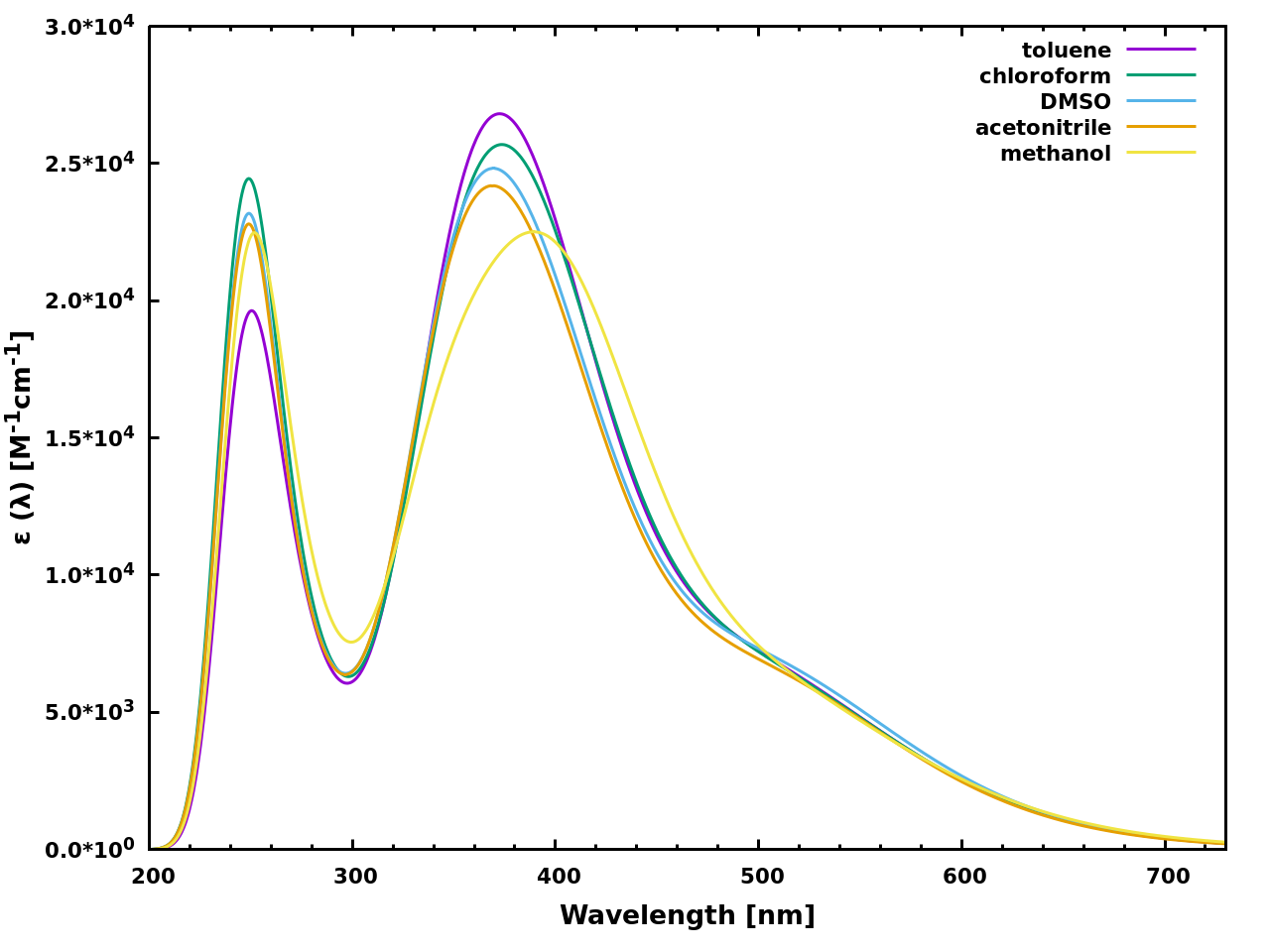

Supplement: Supplementary file 2 — Supplementary file2 (ZIP 5602 KB) [file 43630_2021_71_MOESM2_ESM.zip › simulated_spectra/12.png]

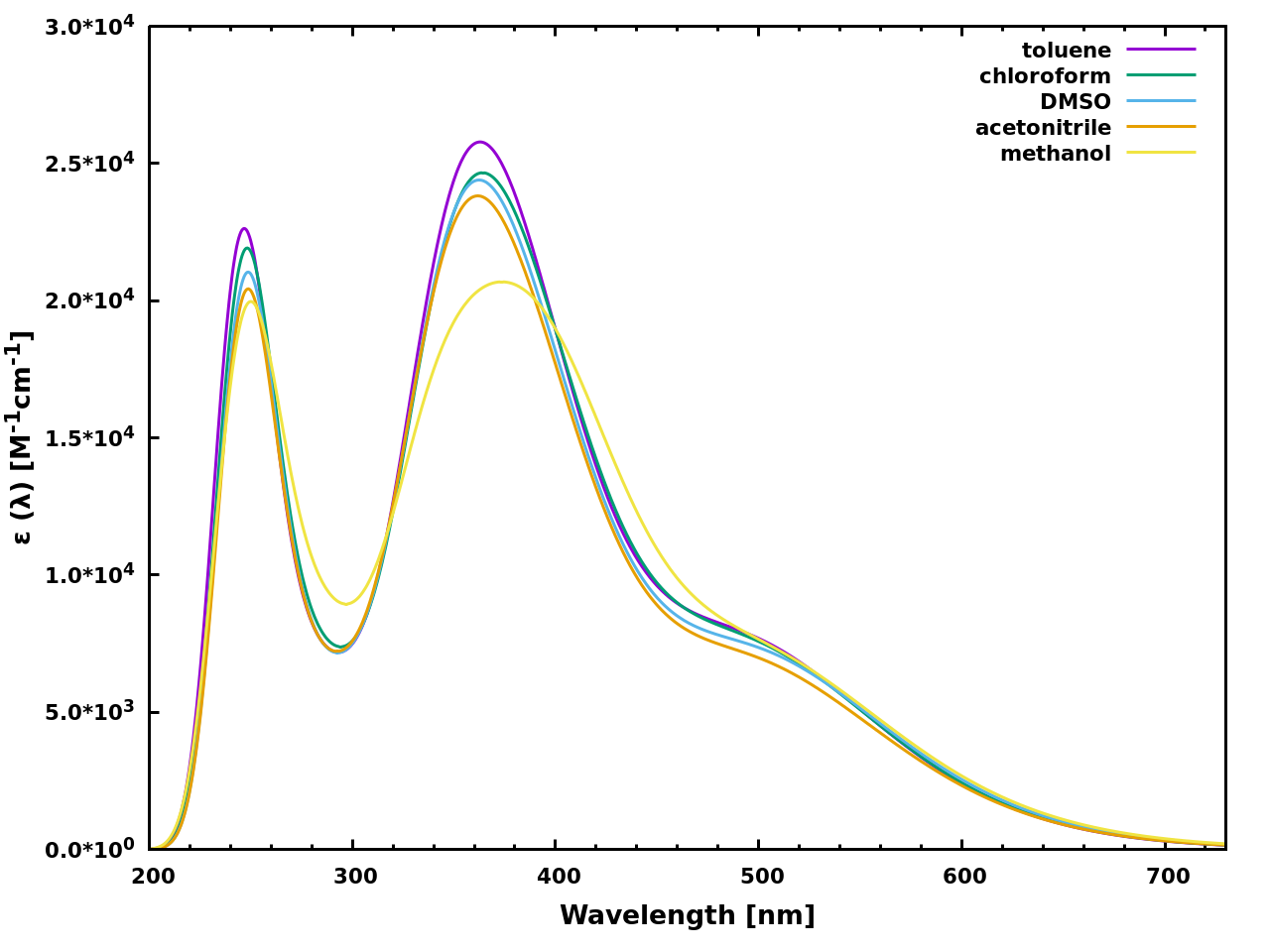

Supplement: Supplementary file 2 — Supplementary file2 (ZIP 5602 KB) [file 43630_2021_71_MOESM2_ESM.zip › simulated_spectra/13.png]

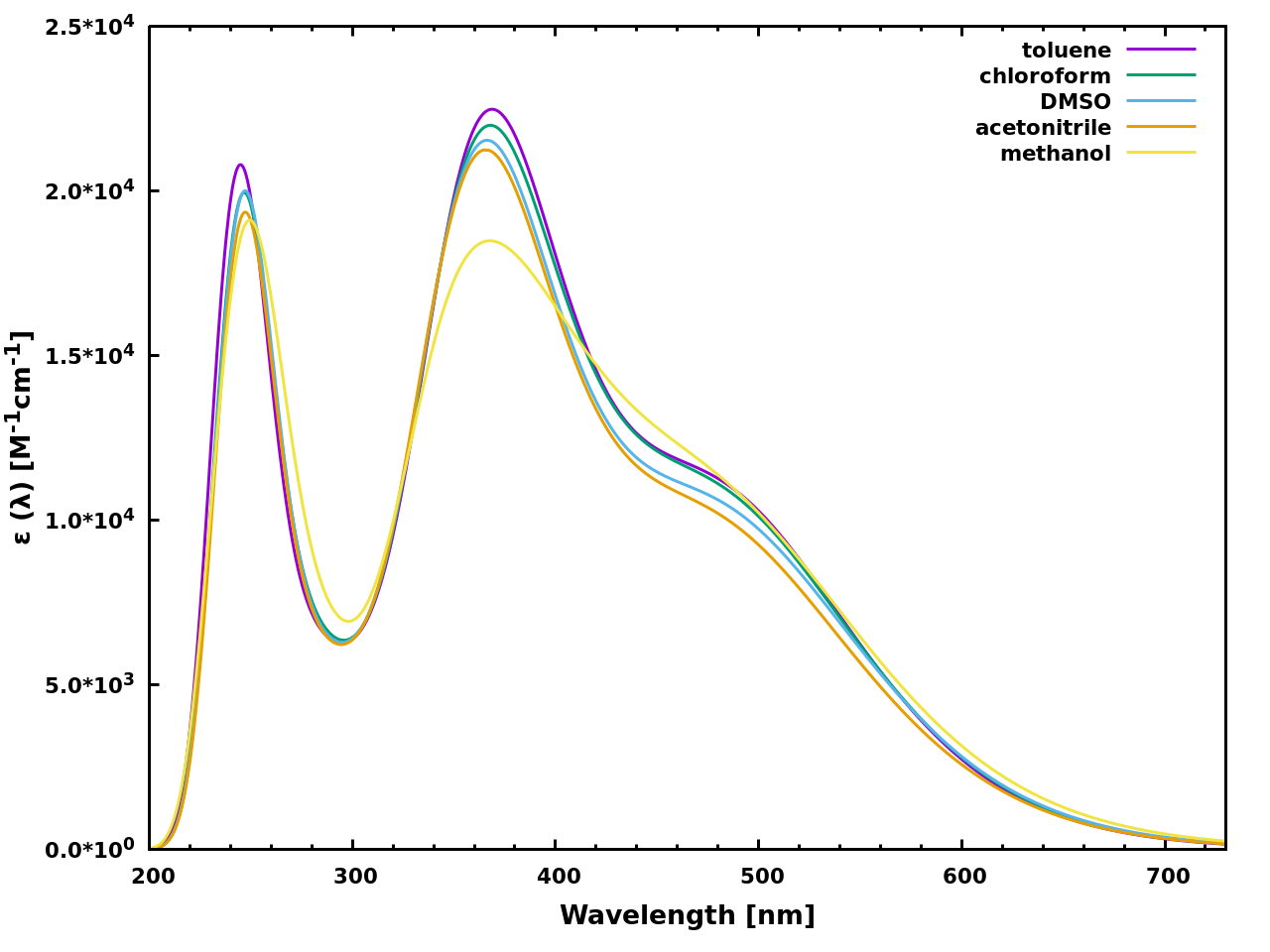

Supplement: Supplementary file 2 — Supplementary file2 (ZIP 5602 KB) [file 43630_2021_71_MOESM2_ESM.zip › simulated_spectra/14.png]

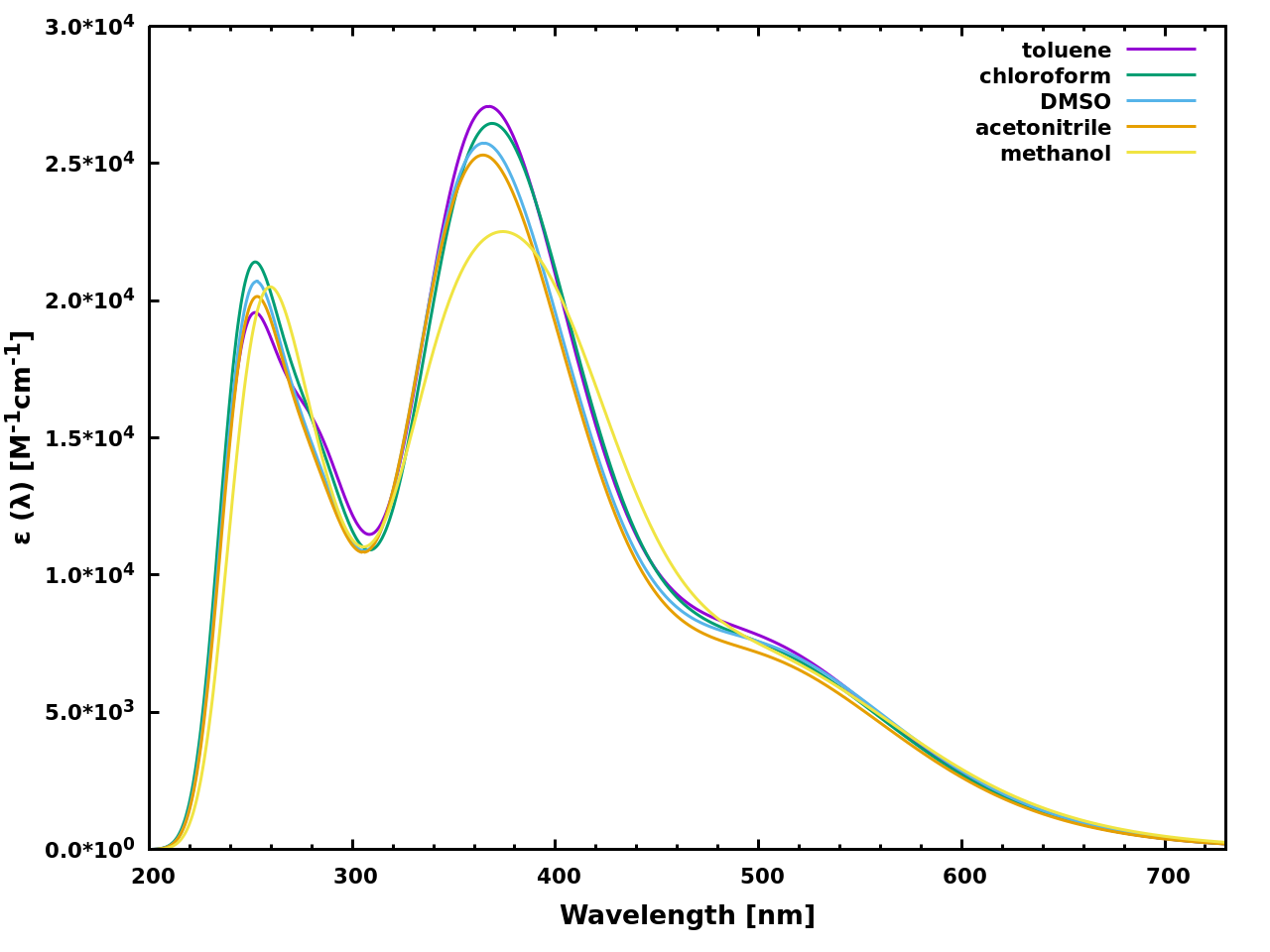

Supplement: Supplementary file 2 — Supplementary file2 (ZIP 5602 KB) [file 43630_2021_71_MOESM2_ESM.zip › simulated_spectra/15.png]

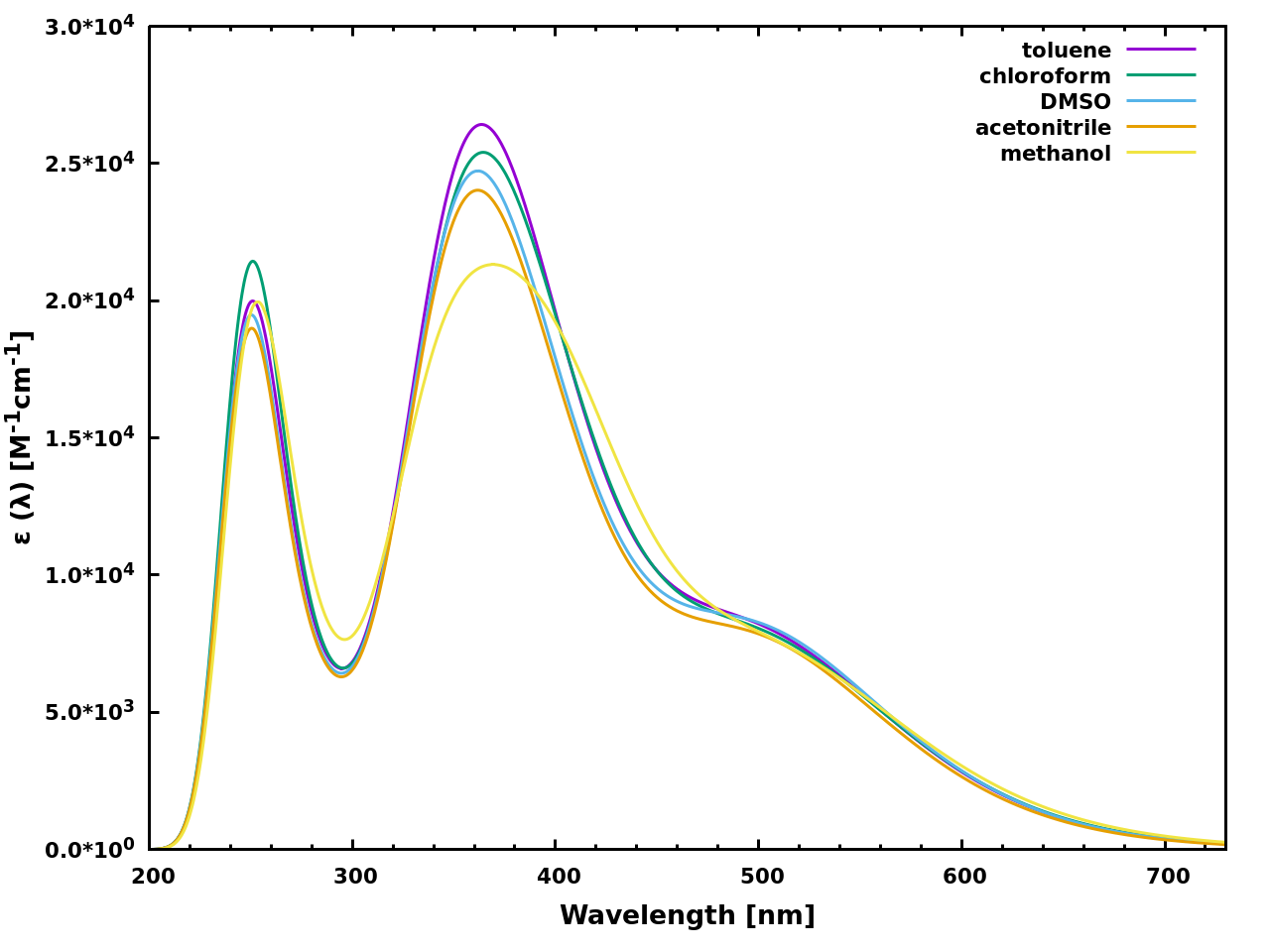

Supplement: Supplementary file 2 — Supplementary file2 (ZIP 5602 KB) [file 43630_2021_71_MOESM2_ESM.zip › simulated_spectra/16.png]

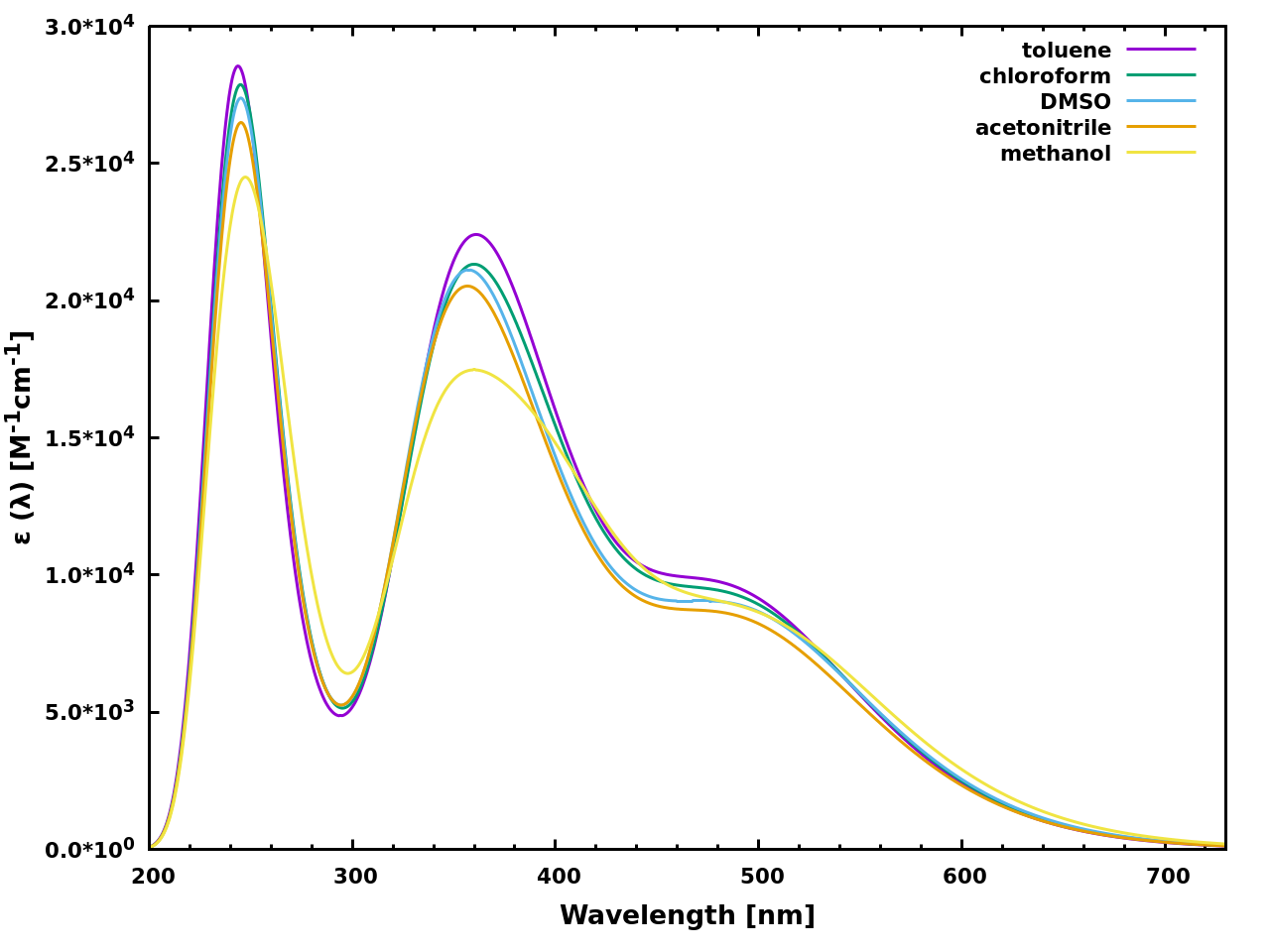

Supplement: Supplementary file 2 — Supplementary file2 (ZIP 5602 KB) [file 43630_2021_71_MOESM2_ESM.zip › simulated_spectra/17.png]

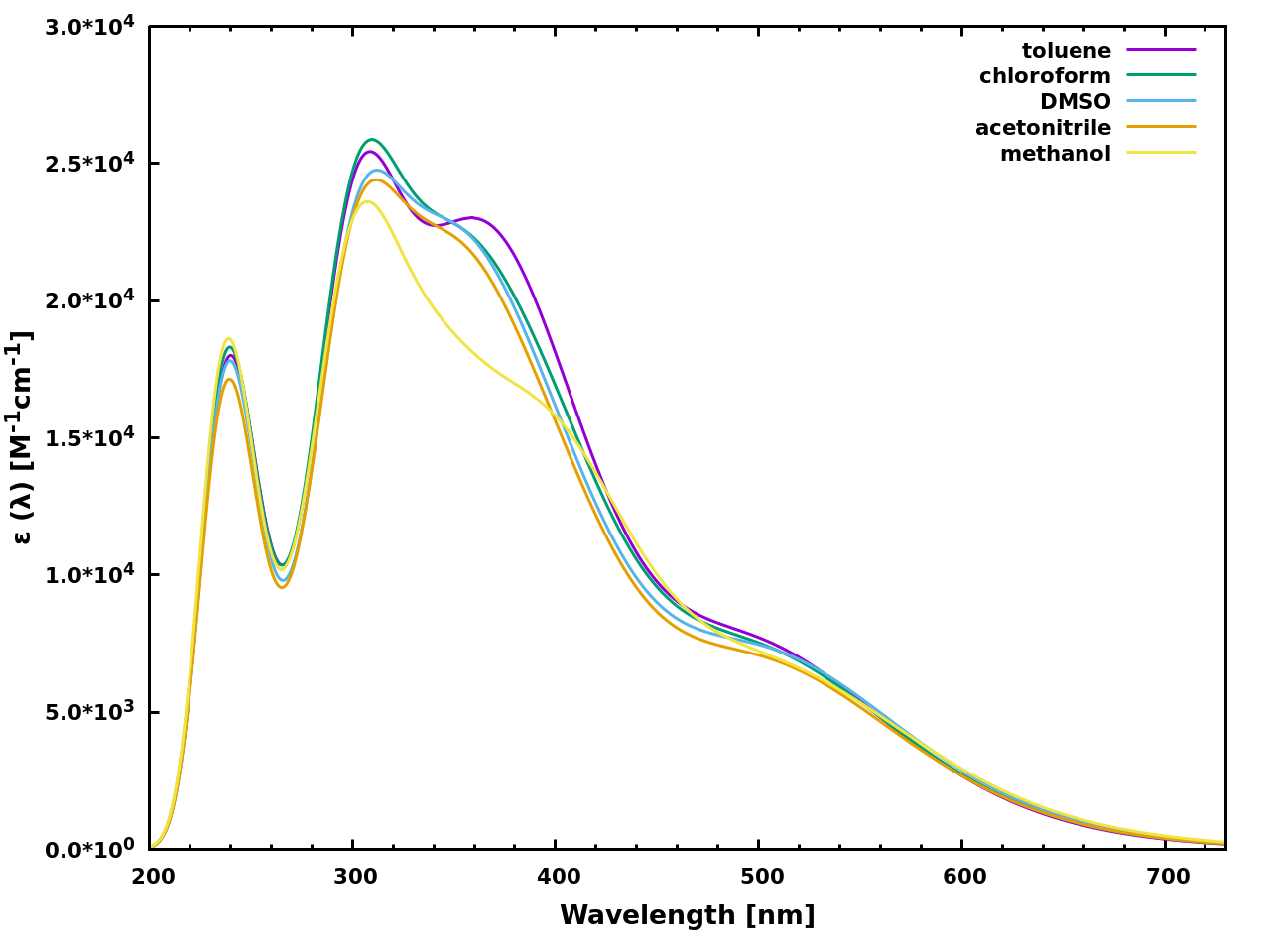

Supplement: Supplementary file 2 — Supplementary file2 (ZIP 5602 KB) [file 43630_2021_71_MOESM2_ESM.zip › simulated_spectra/18.png]

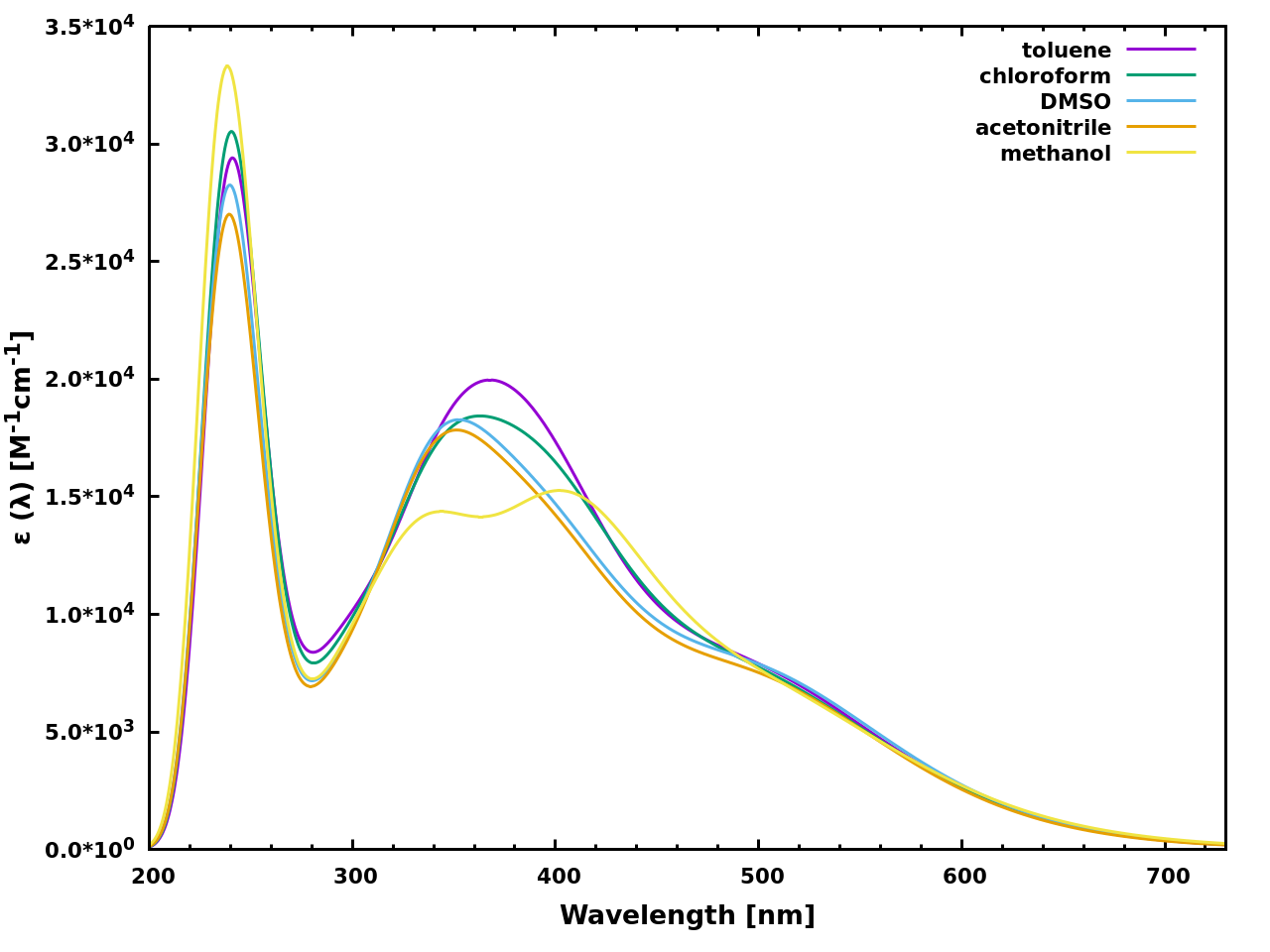

Supplement: Supplementary file 2 — Supplementary file2 (ZIP 5602 KB) [file 43630_2021_71_MOESM2_ESM.zip › simulated_spectra/19.png]

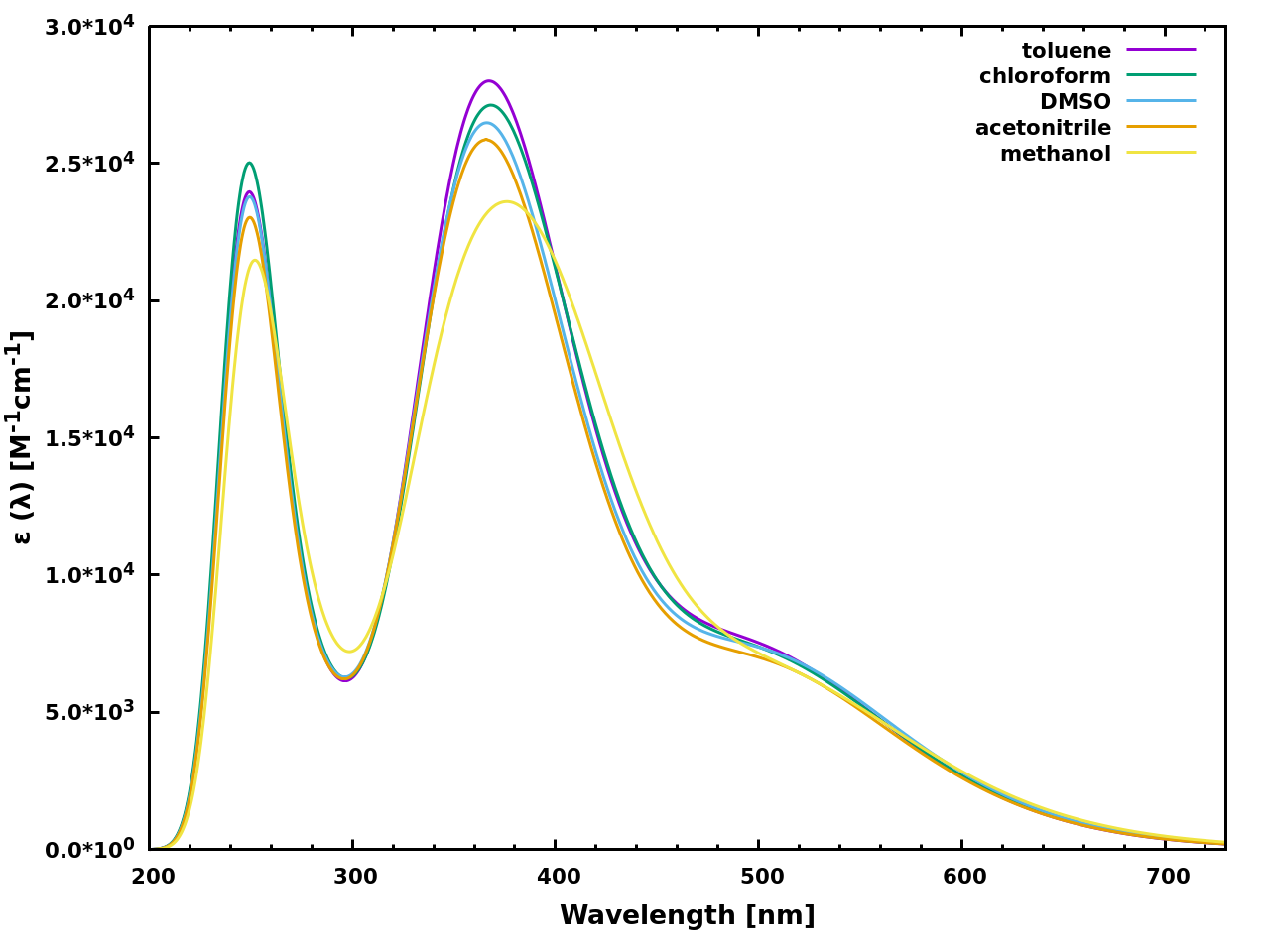

Supplement: Supplementary file 2 — Supplementary file2 (ZIP 5602 KB) [file 43630_2021_71_MOESM2_ESM.zip › simulated_spectra/2.png]

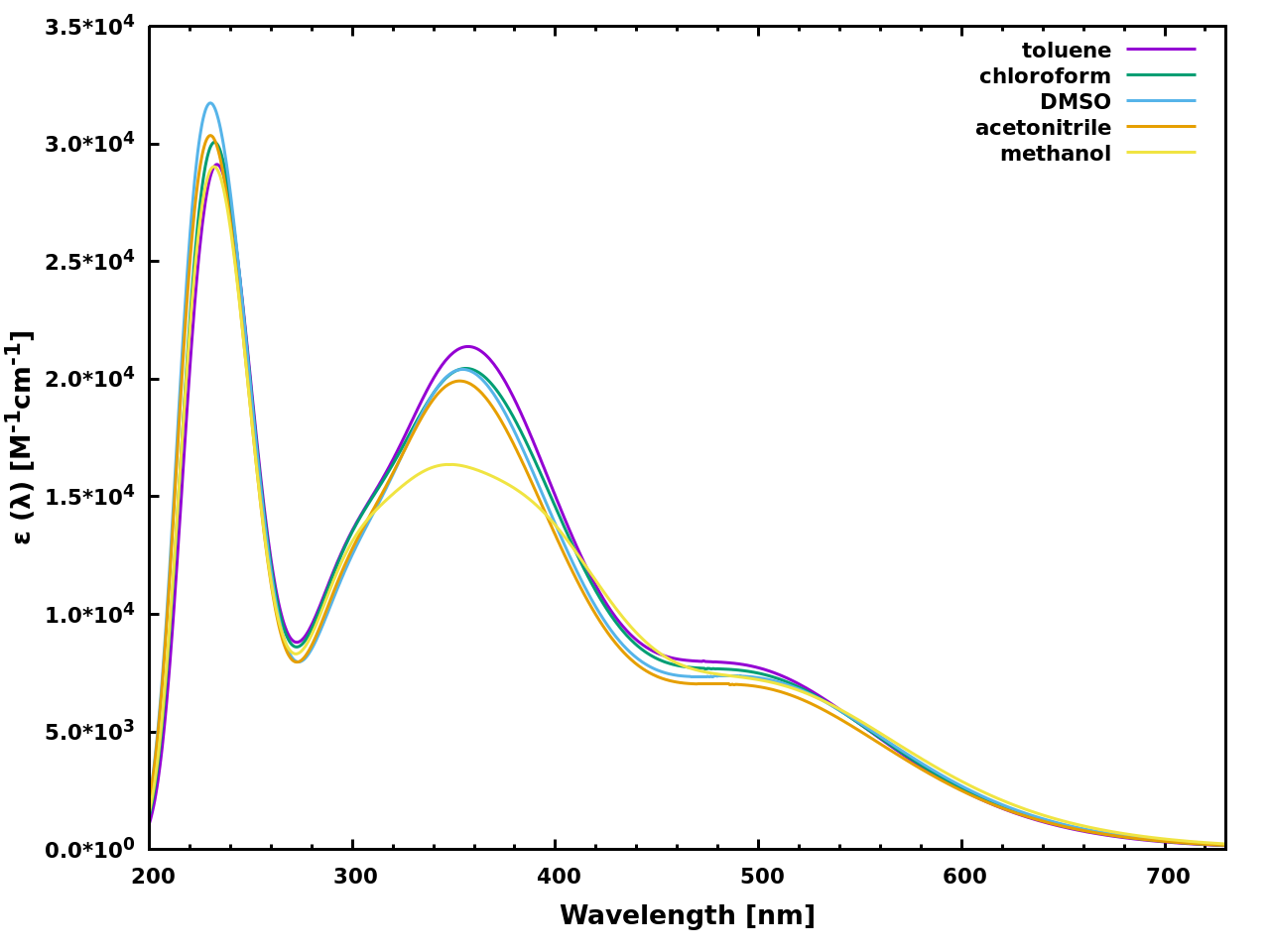

Supplement: Supplementary file 2 — Supplementary file2 (ZIP 5602 KB) [file 43630_2021_71_MOESM2_ESM.zip › simulated_spectra/20.png]

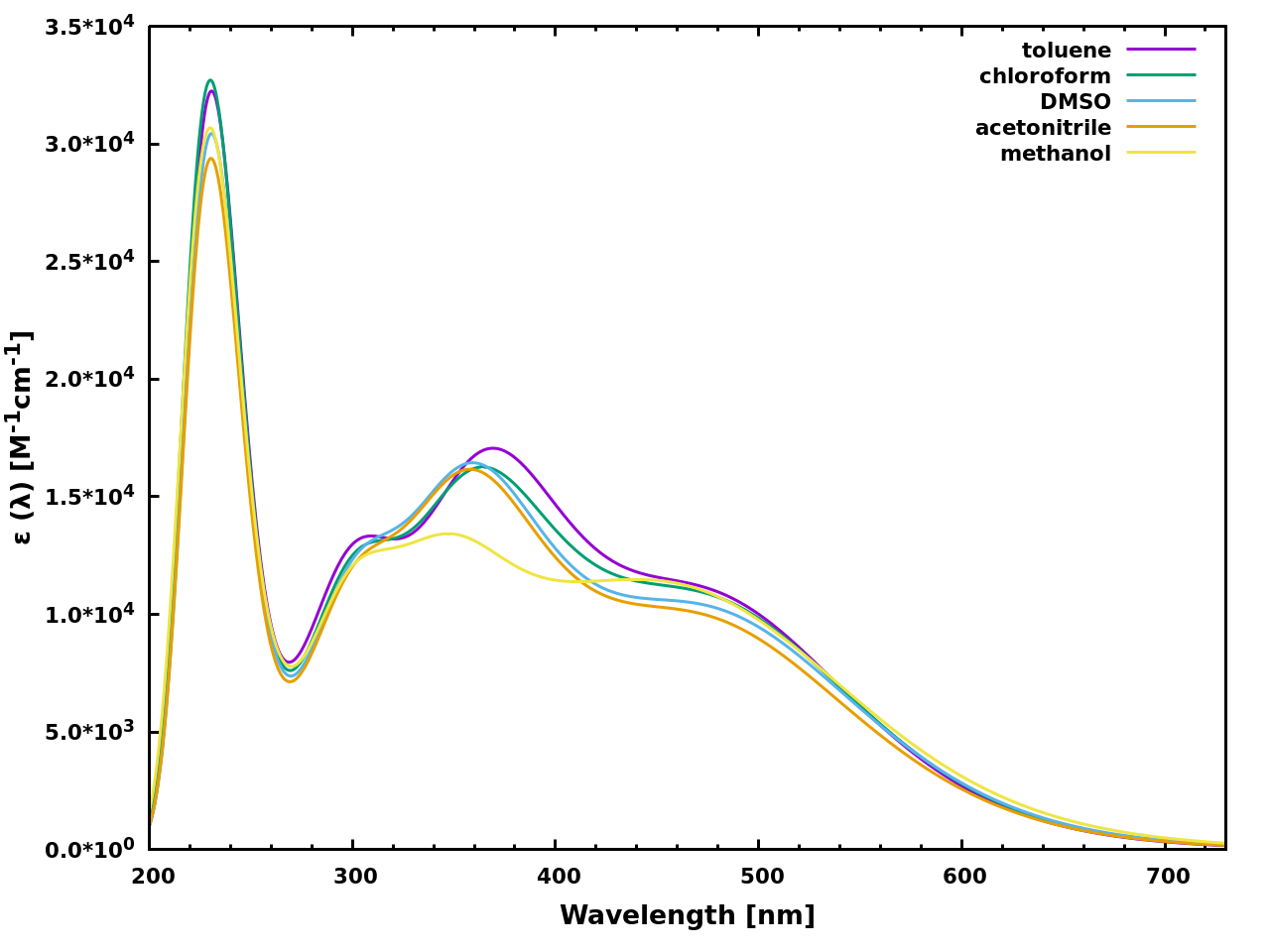

Supplement: Supplementary file 2 — Supplementary file2 (ZIP 5602 KB) [file 43630_2021_71_MOESM2_ESM.zip › simulated_spectra/21.png]

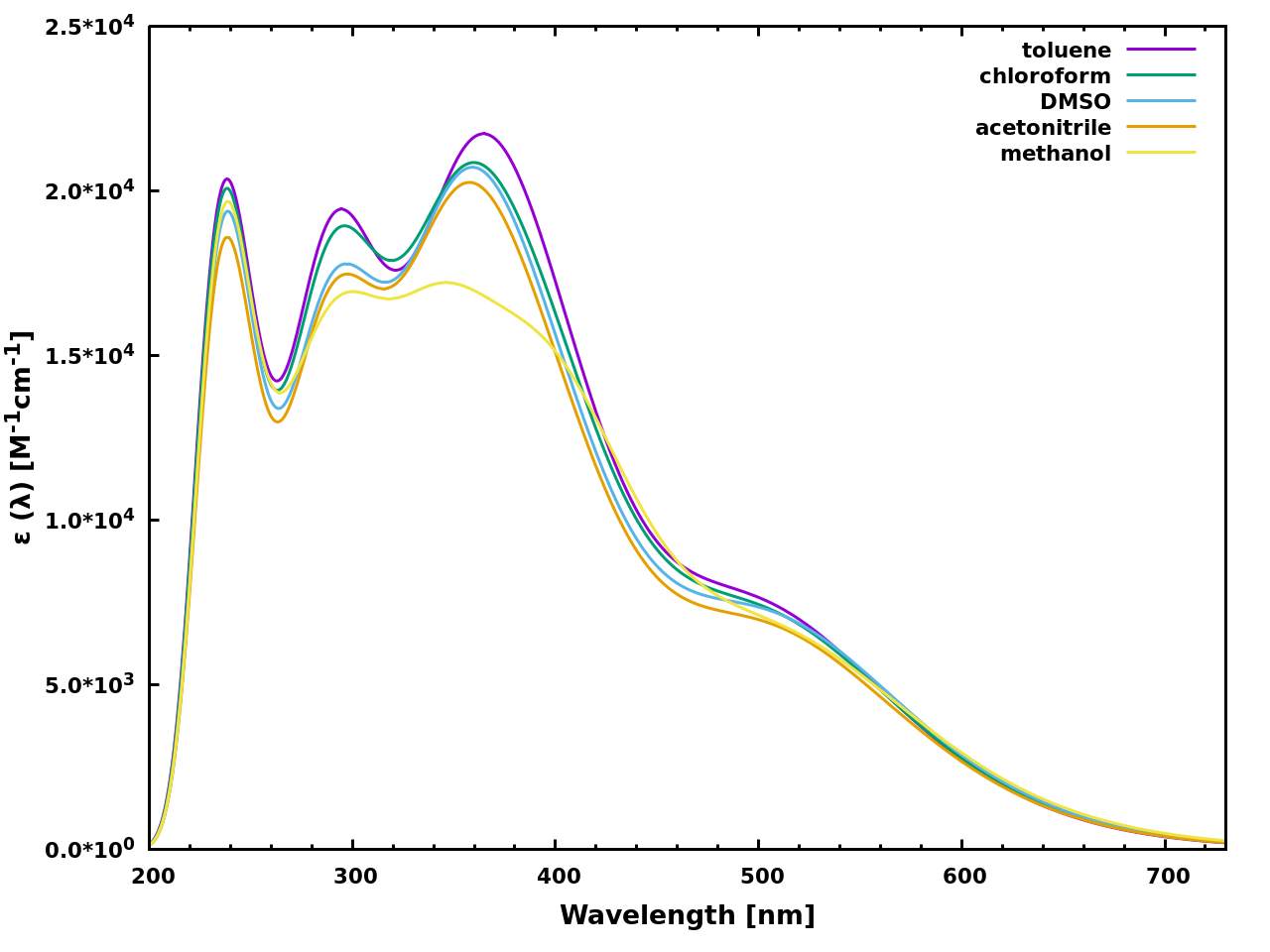

Supplement: Supplementary file 2 — Supplementary file2 (ZIP 5602 KB) [file 43630_2021_71_MOESM2_ESM.zip › simulated_spectra/22.png]

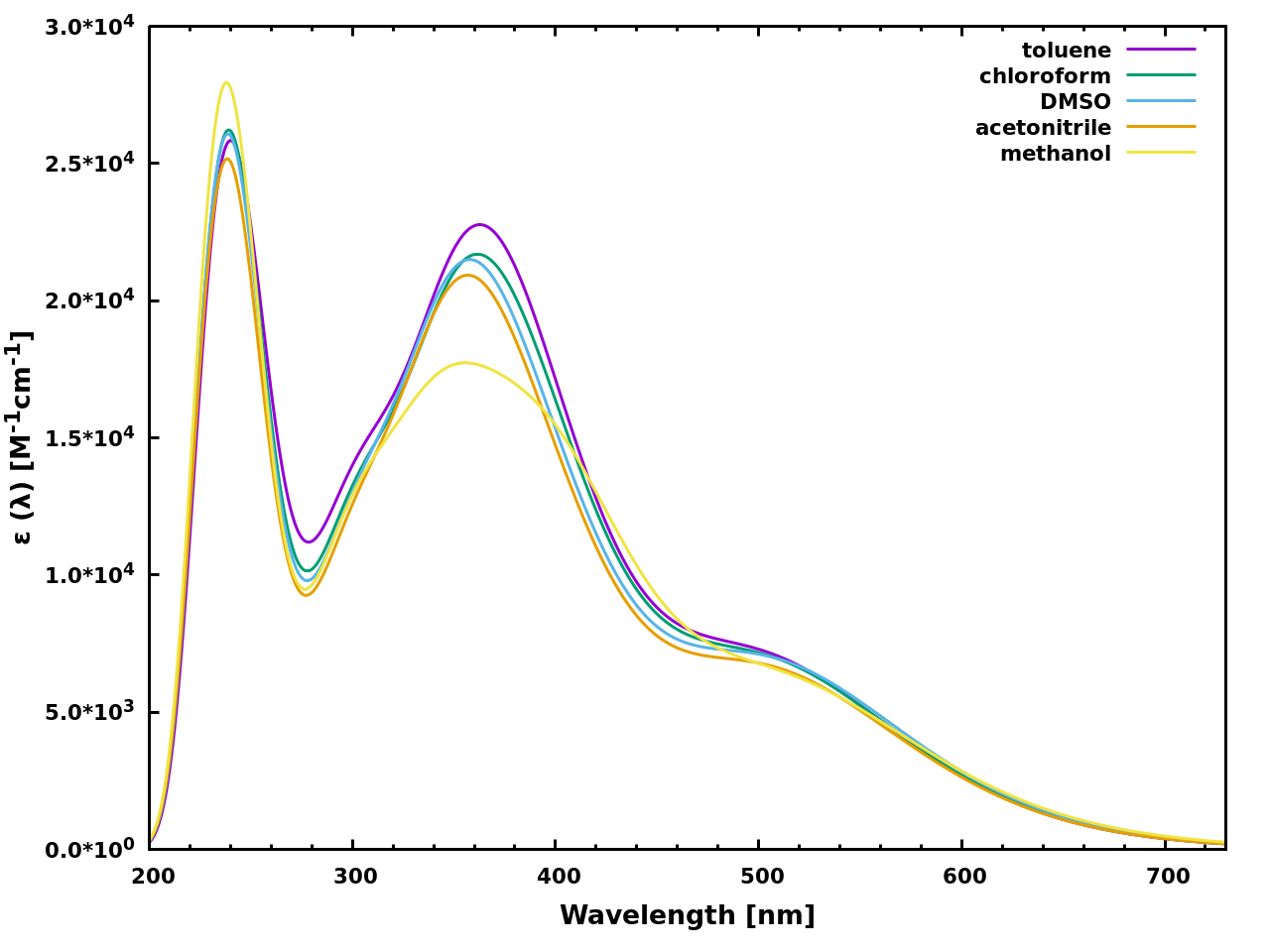

Supplement: Supplementary file 2 — Supplementary file2 (ZIP 5602 KB) [file 43630_2021_71_MOESM2_ESM.zip › simulated_spectra/23.png]

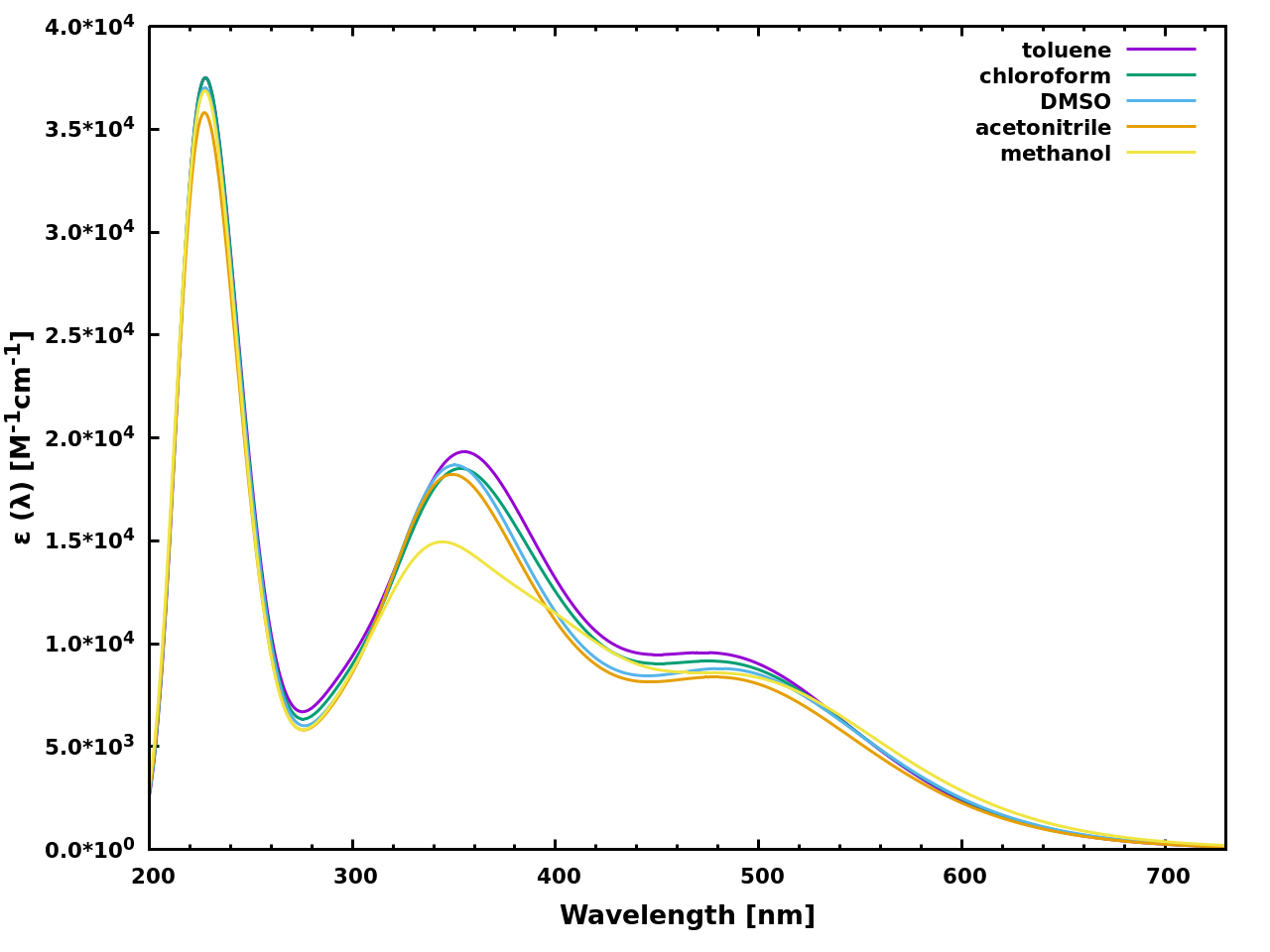

Supplement: Supplementary file 2 — Supplementary file2 (ZIP 5602 KB) [file 43630_2021_71_MOESM2_ESM.zip › simulated_spectra/24.png]

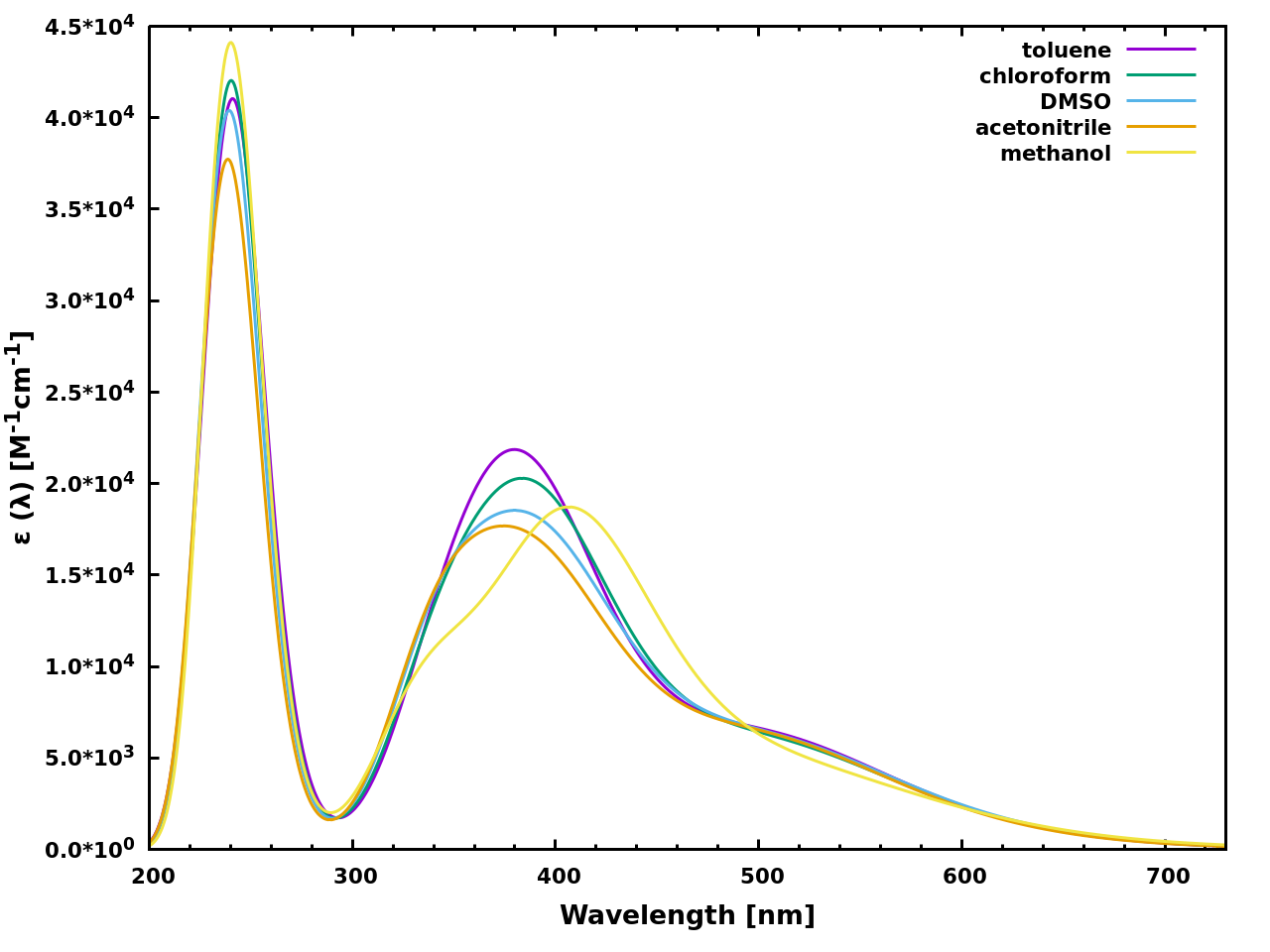

Supplement: Supplementary file 2 — Supplementary file2 (ZIP 5602 KB) [file 43630_2021_71_MOESM2_ESM.zip › simulated_spectra/25.png]

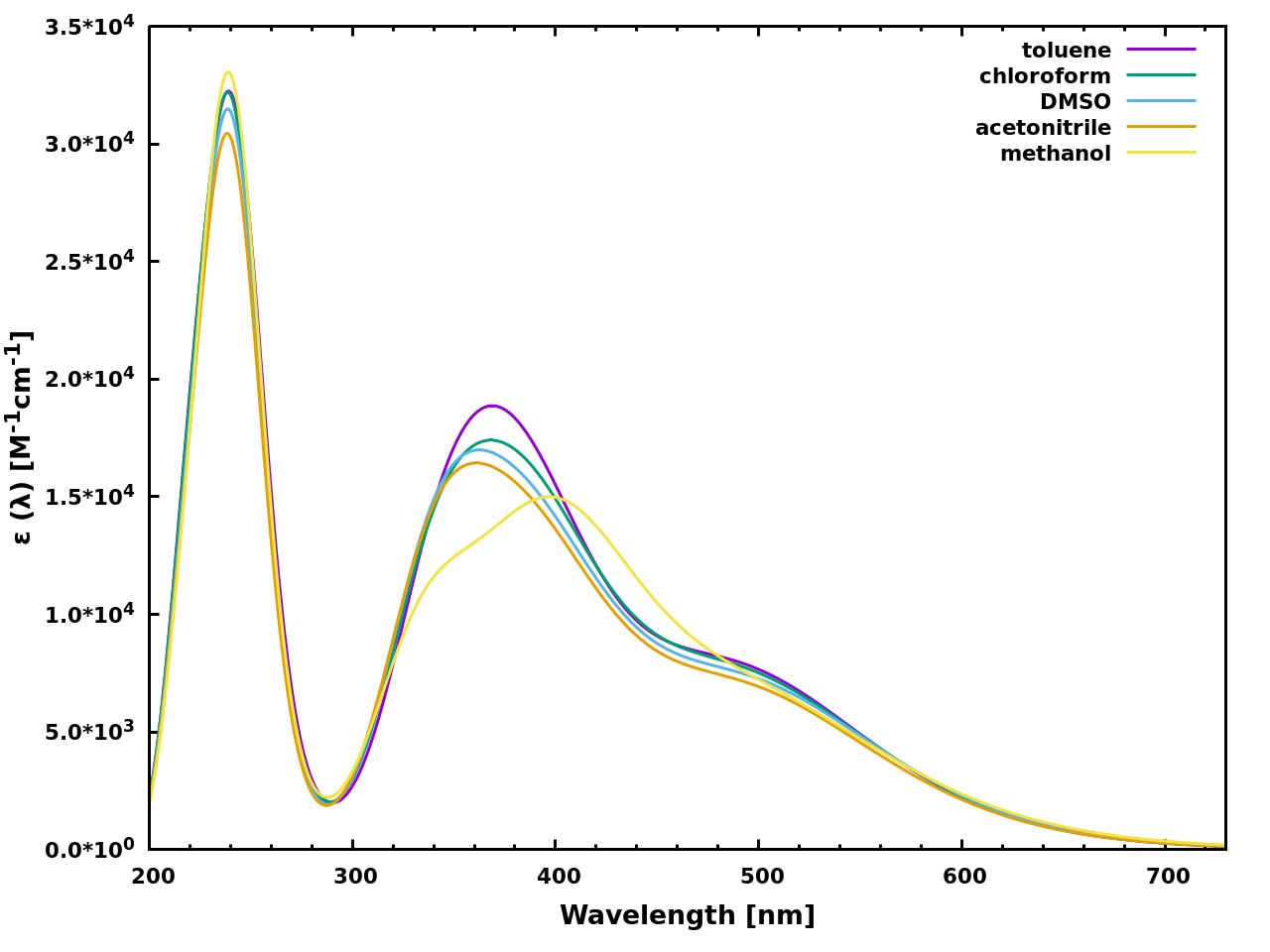

Supplement: Supplementary file 2 — Supplementary file2 (ZIP 5602 KB) [file 43630_2021_71_MOESM2_ESM.zip › simulated_spectra/26.png]

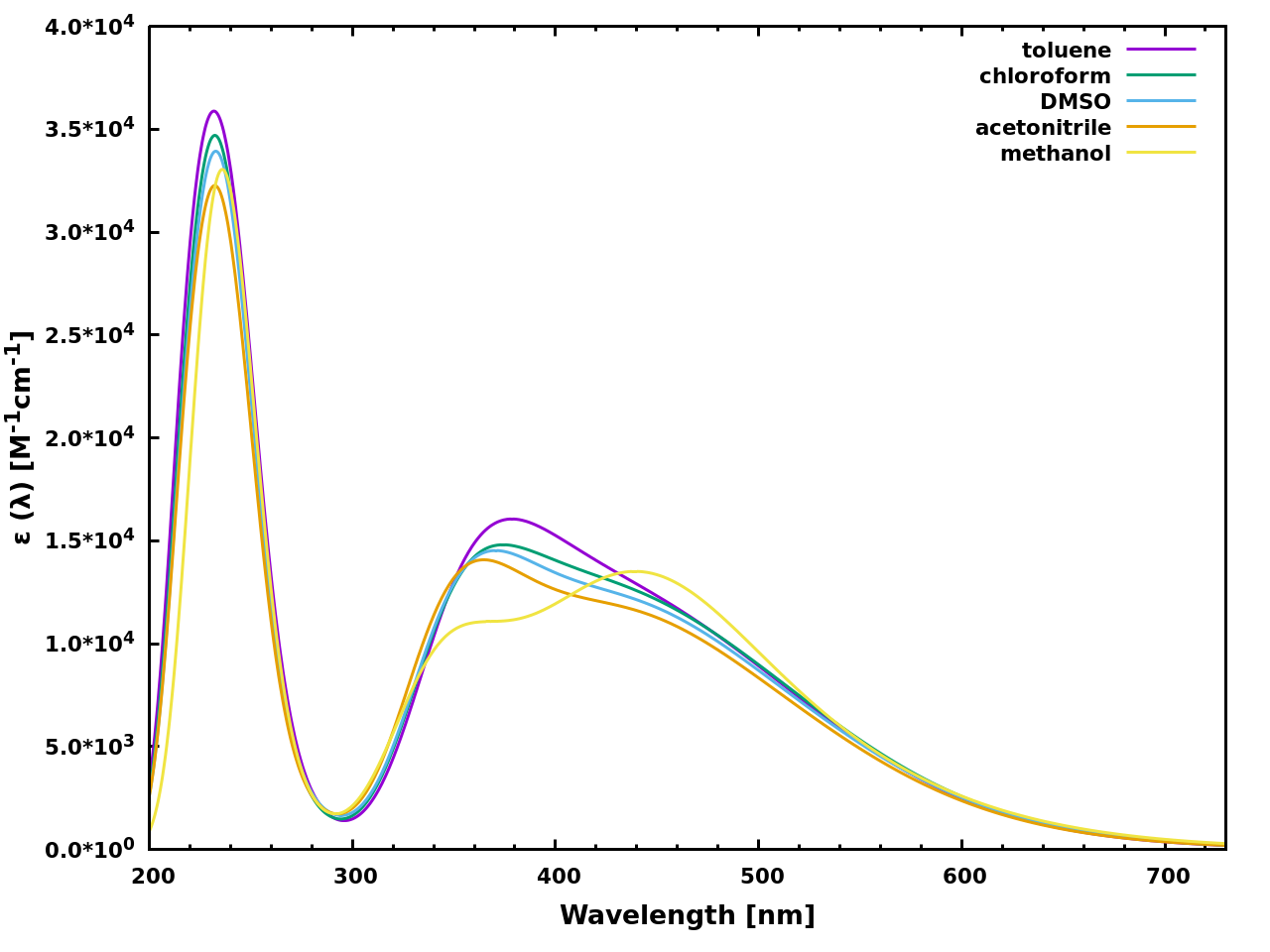

Supplement: Supplementary file 2 — Supplementary file2 (ZIP 5602 KB) [file 43630_2021_71_MOESM2_ESM.zip › simulated_spectra/27.png]

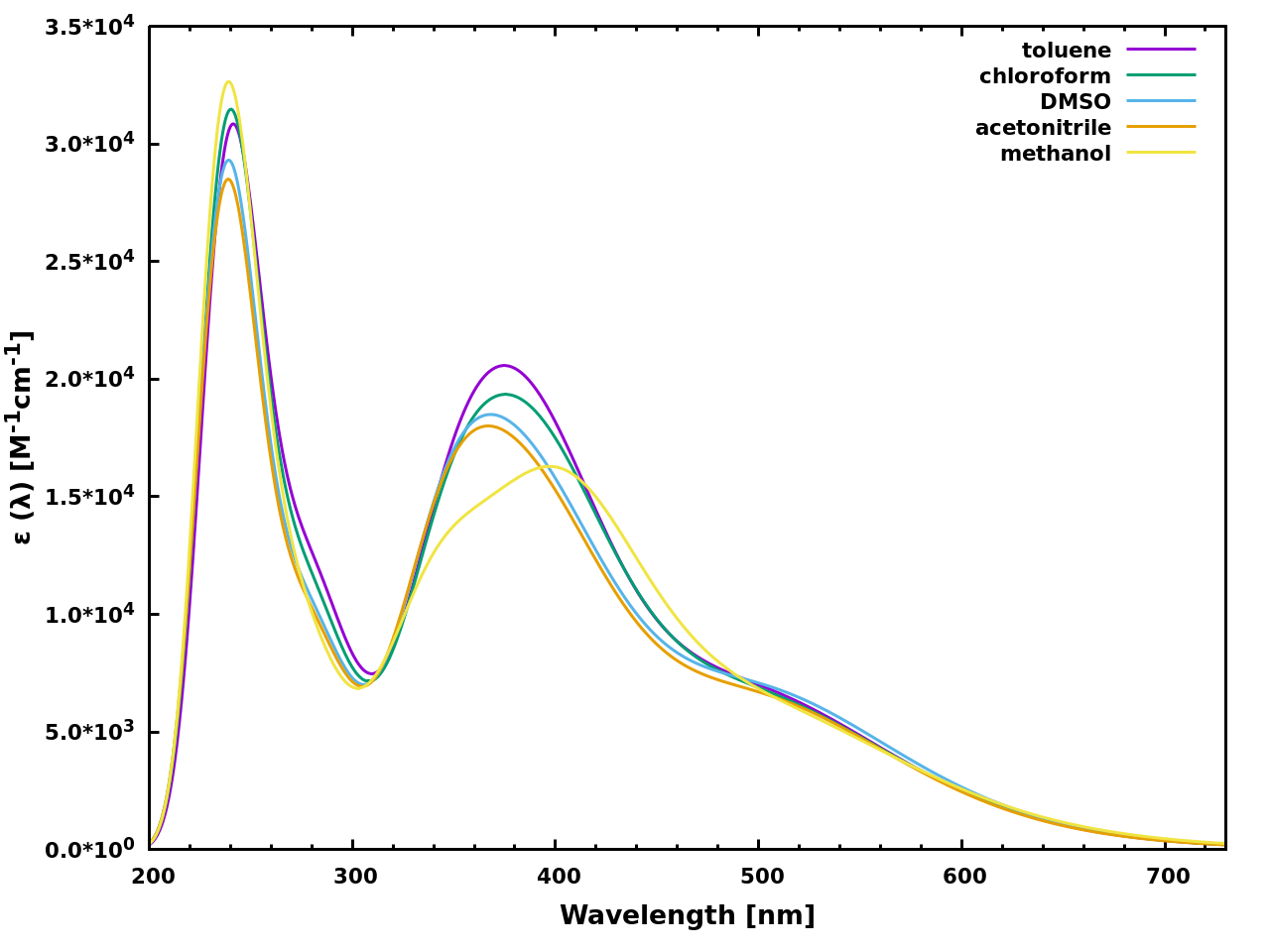

Supplement: Supplementary file 2 — Supplementary file2 (ZIP 5602 KB) [file 43630_2021_71_MOESM2_ESM.zip › simulated_spectra/28.png]

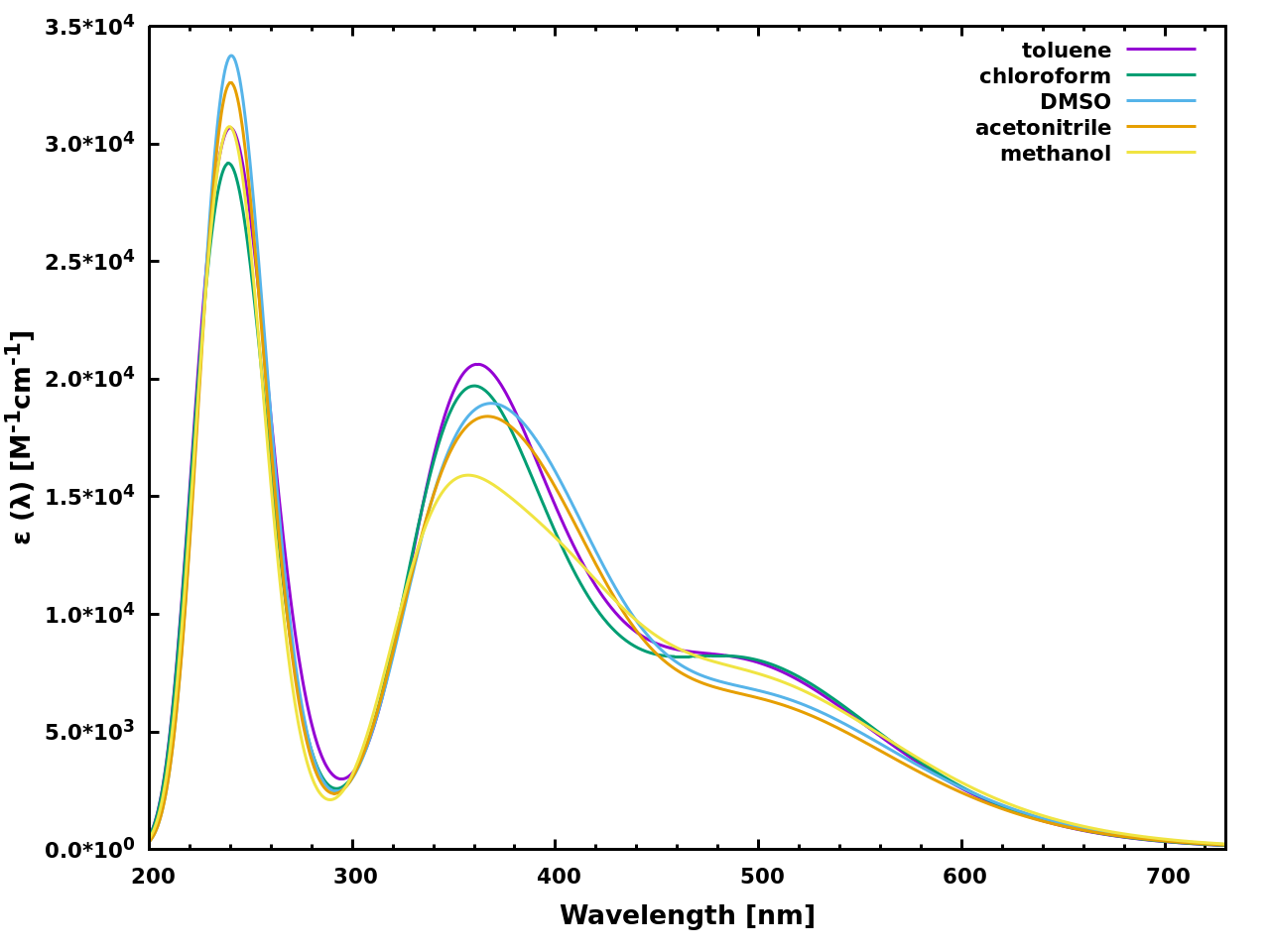

Supplement: Supplementary file 2 — Supplementary file2 (ZIP 5602 KB) [file 43630_2021_71_MOESM2_ESM.zip › simulated_spectra/29.png]

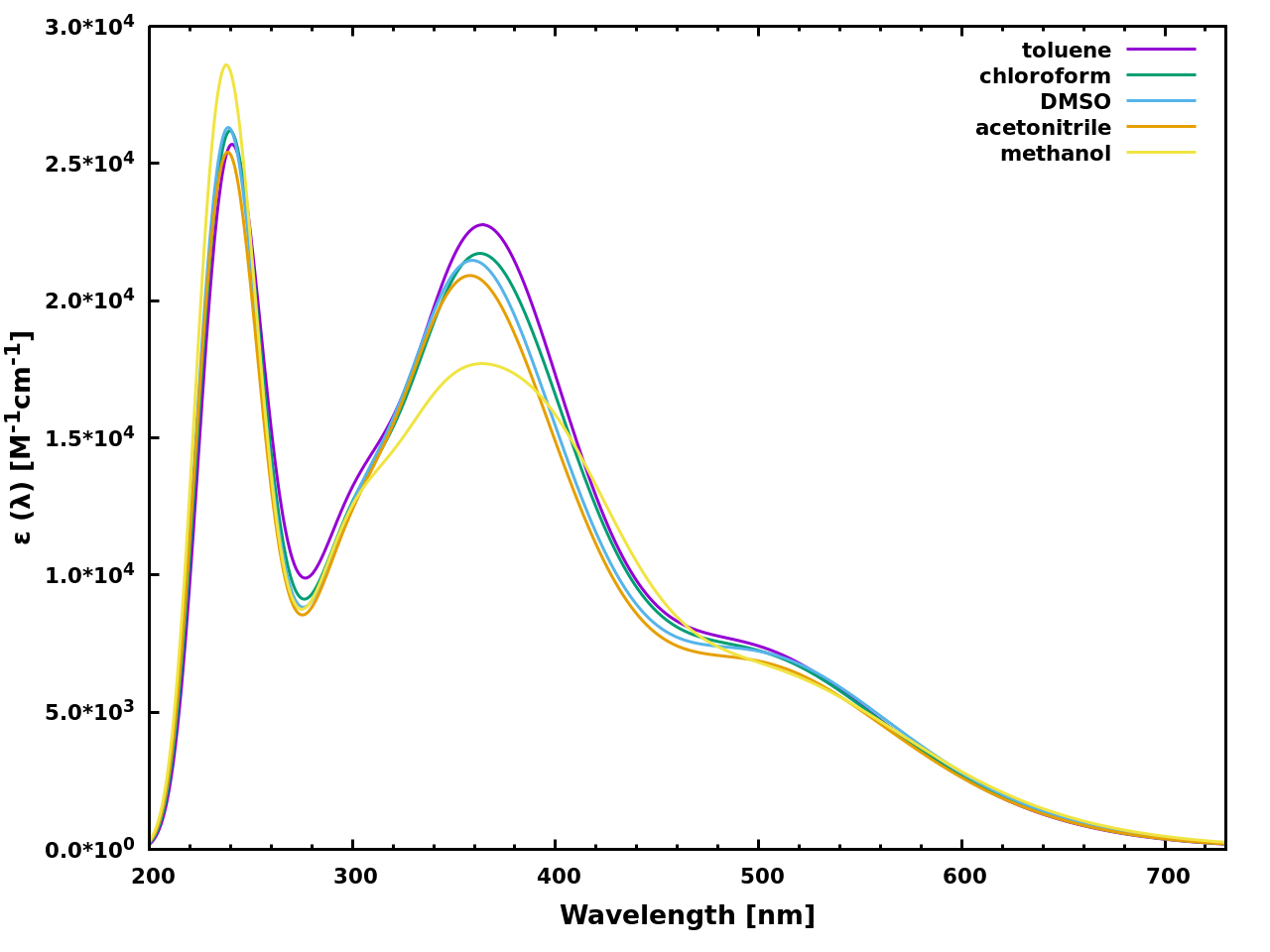

Supplement: Supplementary file 2 — Supplementary file2 (ZIP 5602 KB) [file 43630_2021_71_MOESM2_ESM.zip › simulated_spectra/3.png]

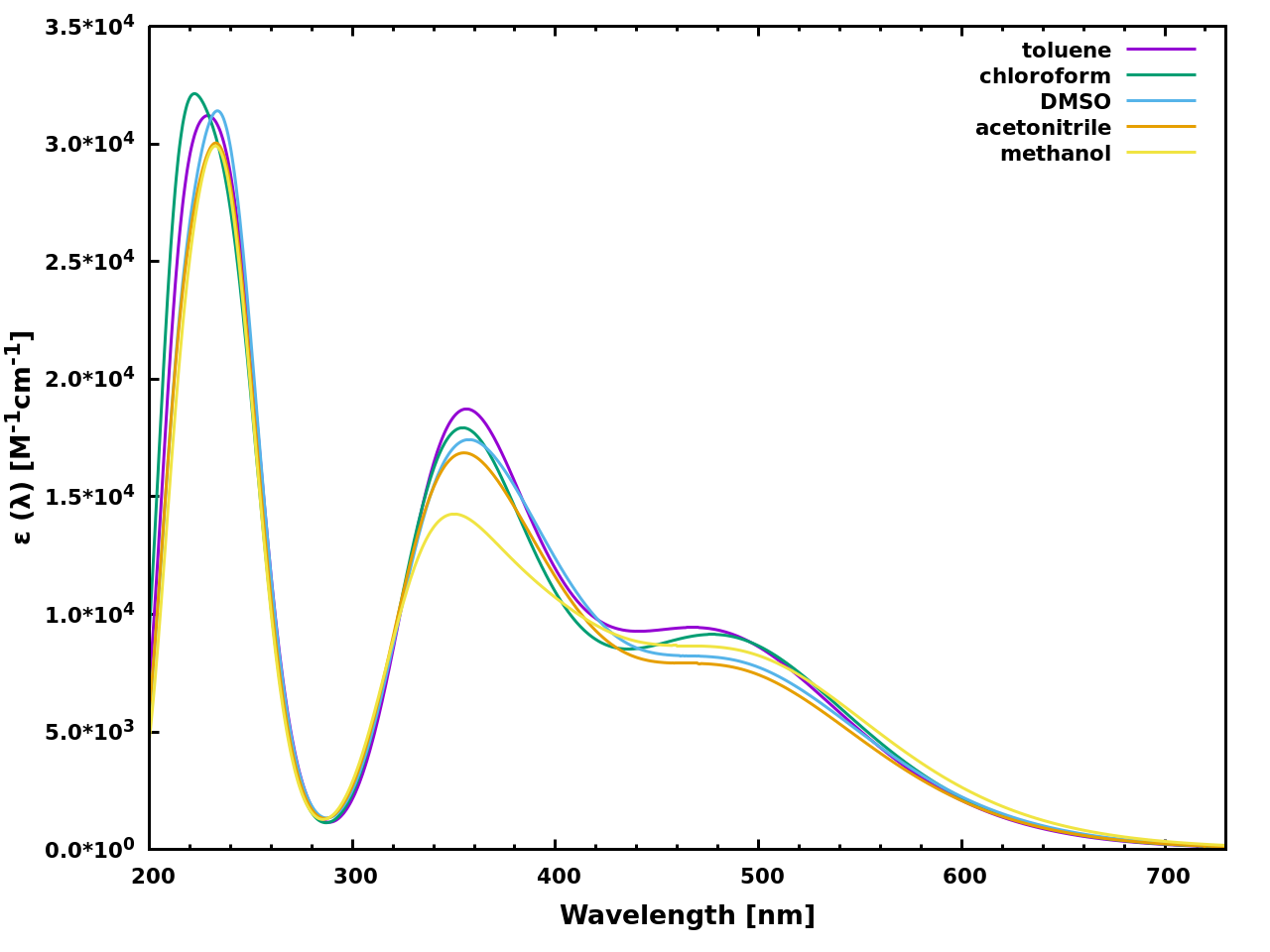

Supplement: Supplementary file 2 — Supplementary file2 (ZIP 5602 KB) [file 43630_2021_71_MOESM2_ESM.zip › simulated_spectra/30.png]

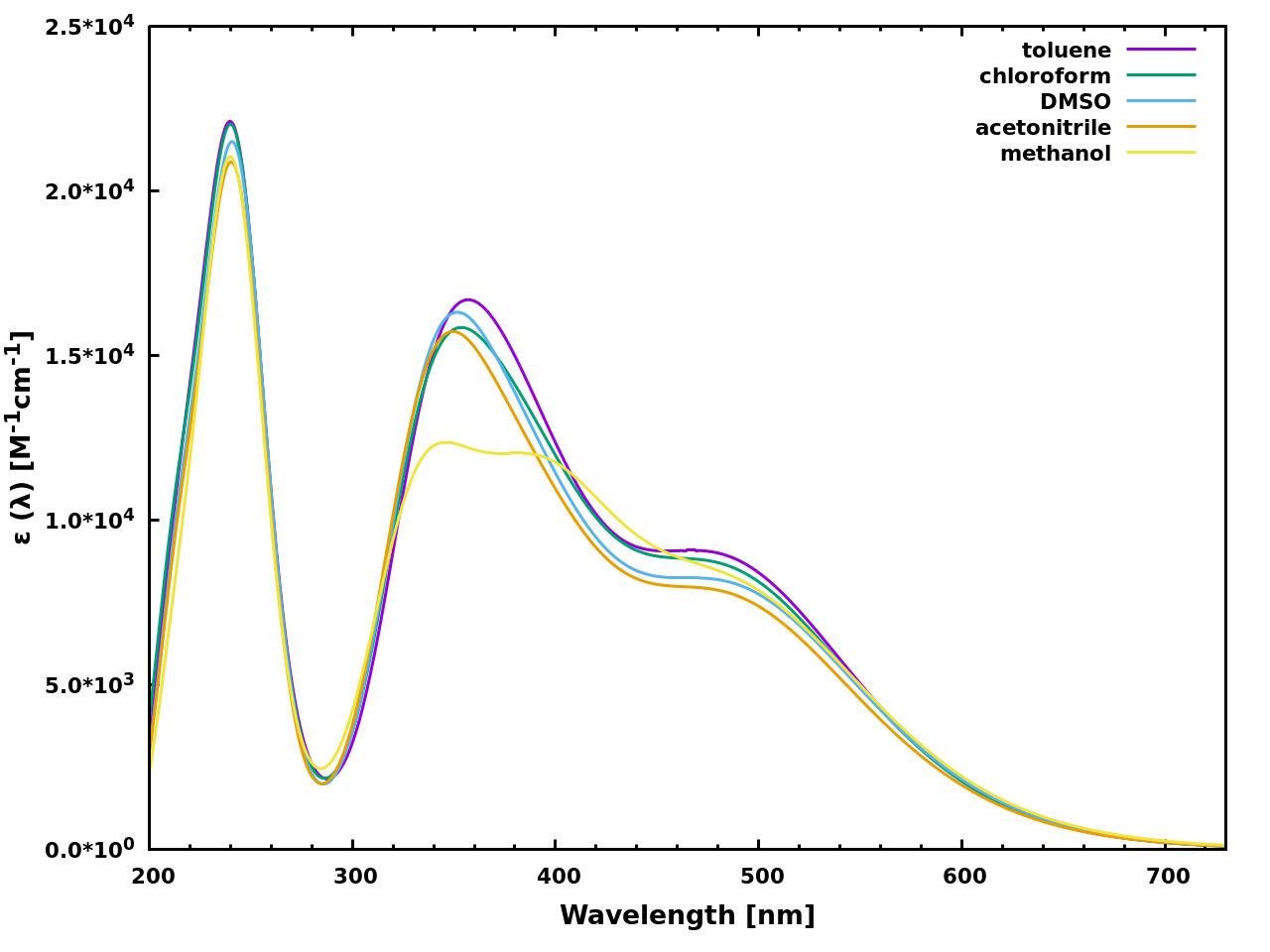

Supplement: Supplementary file 2 — Supplementary file2 (ZIP 5602 KB) [file 43630_2021_71_MOESM2_ESM.zip › simulated_spectra/31.png]

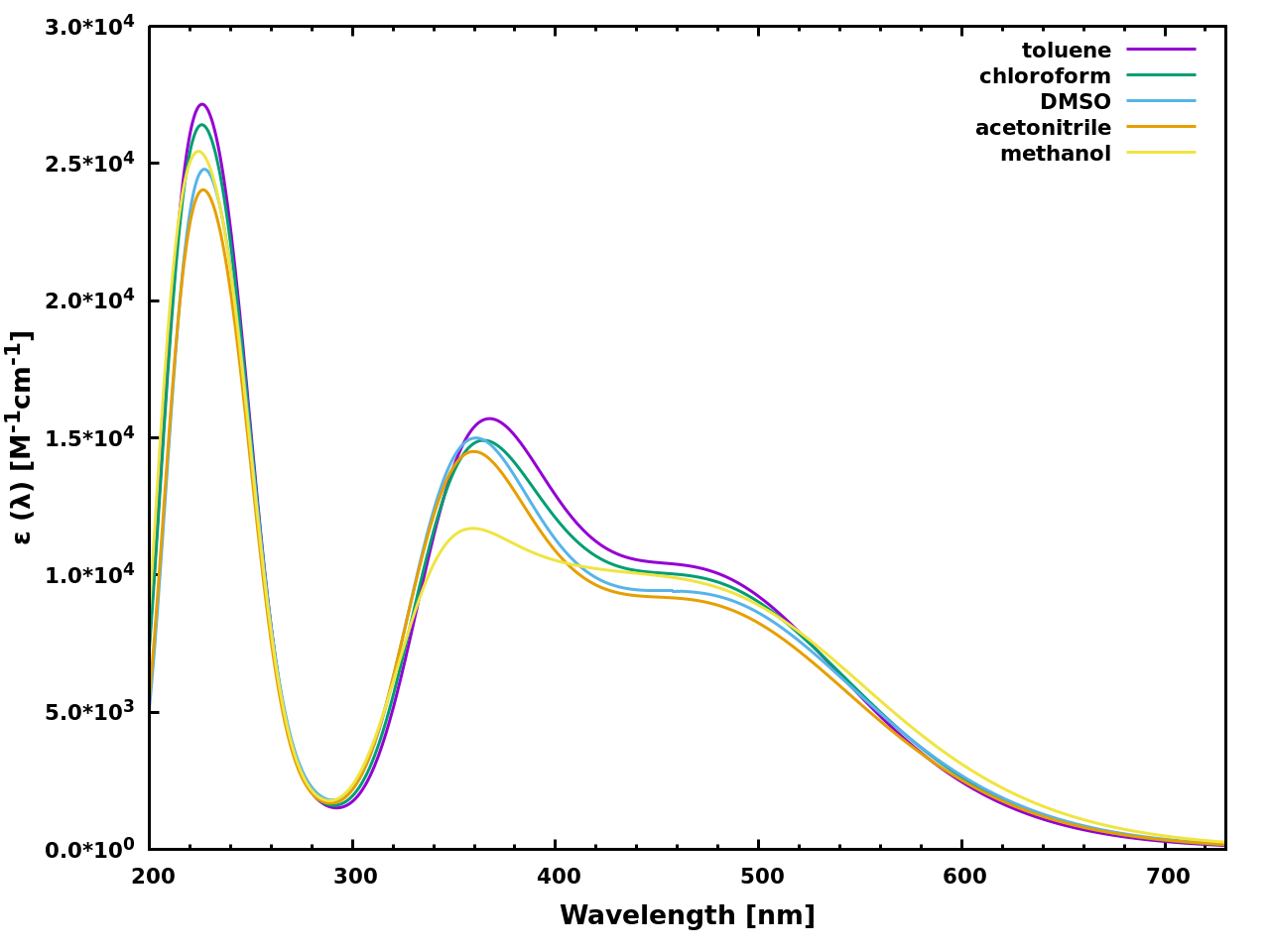

Supplement: Supplementary file 2 — Supplementary file2 (ZIP 5602 KB) [file 43630_2021_71_MOESM2_ESM.zip › simulated_spectra/32.png]

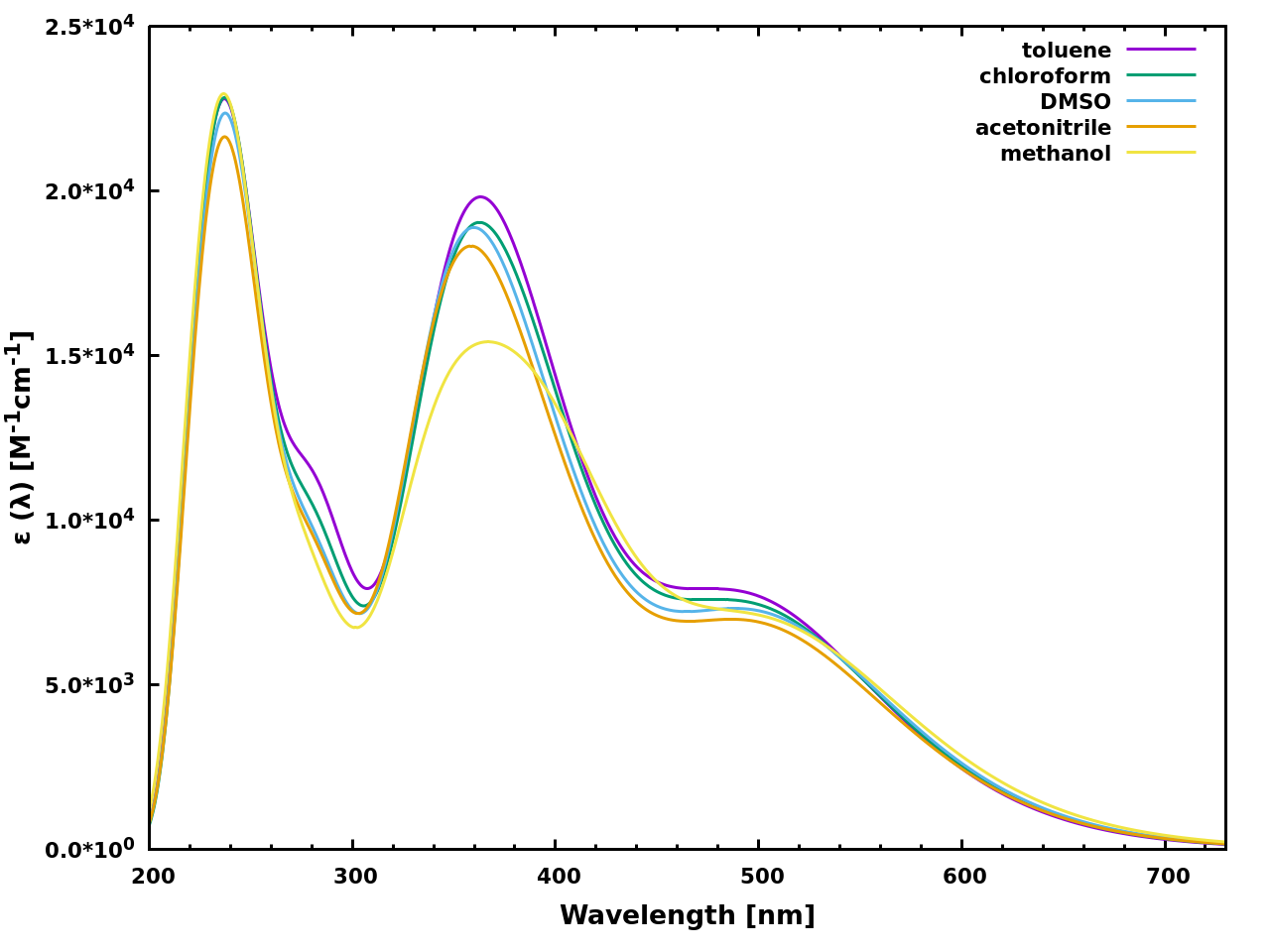

Supplement: Supplementary file 2 — Supplementary file2 (ZIP 5602 KB) [file 43630_2021_71_MOESM2_ESM.zip › simulated_spectra/33.png]

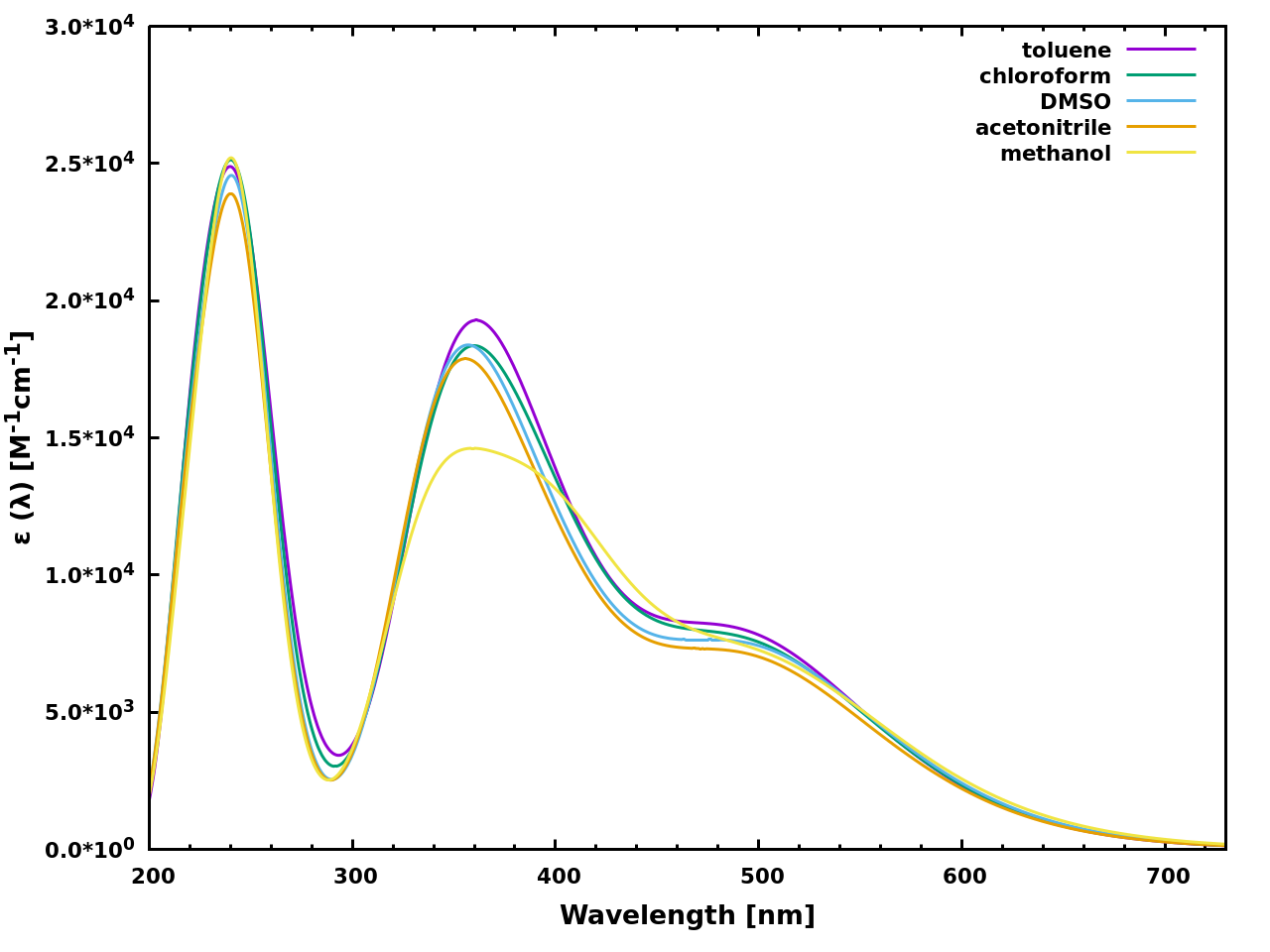

Supplement: Supplementary file 2 — Supplementary file2 (ZIP 5602 KB) [file 43630_2021_71_MOESM2_ESM.zip › simulated_spectra/34.png]

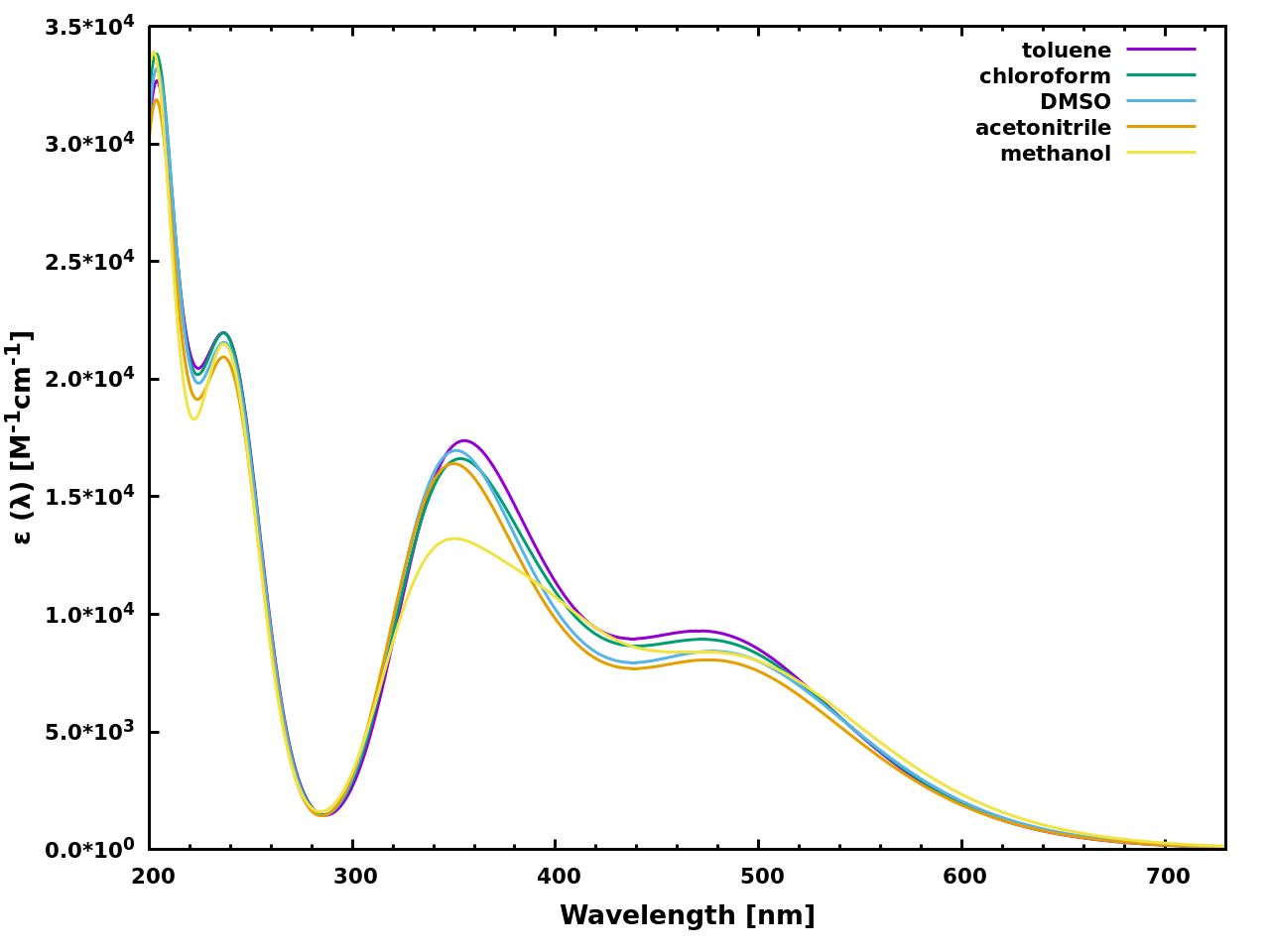

Supplement: Supplementary file 2 — Supplementary file2 (ZIP 5602 KB) [file 43630_2021_71_MOESM2_ESM.zip › simulated_spectra/35.png]

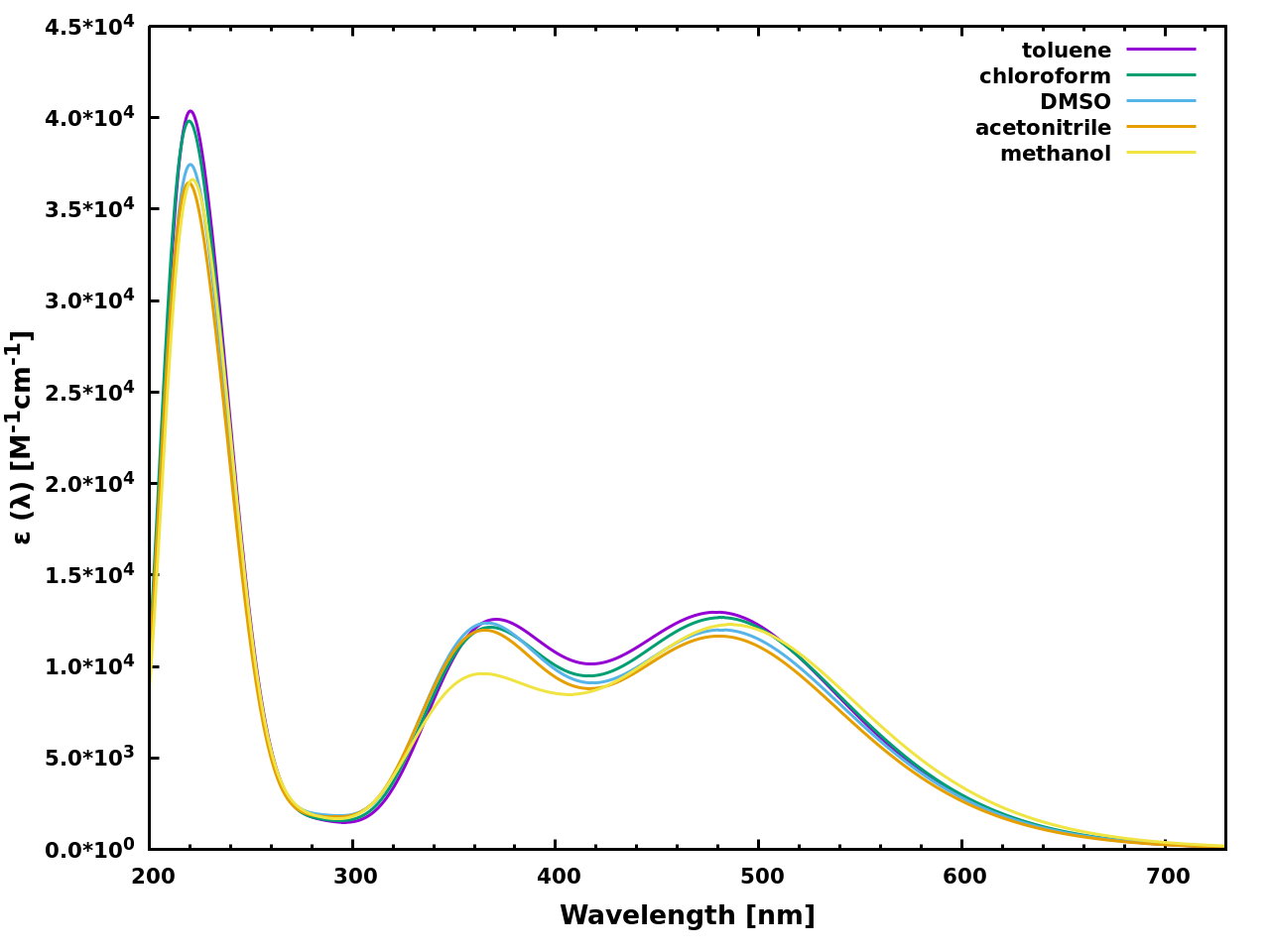

Supplement: Supplementary file 2 — Supplementary file2 (ZIP 5602 KB) [file 43630_2021_71_MOESM2_ESM.zip › simulated_spectra/36.png]

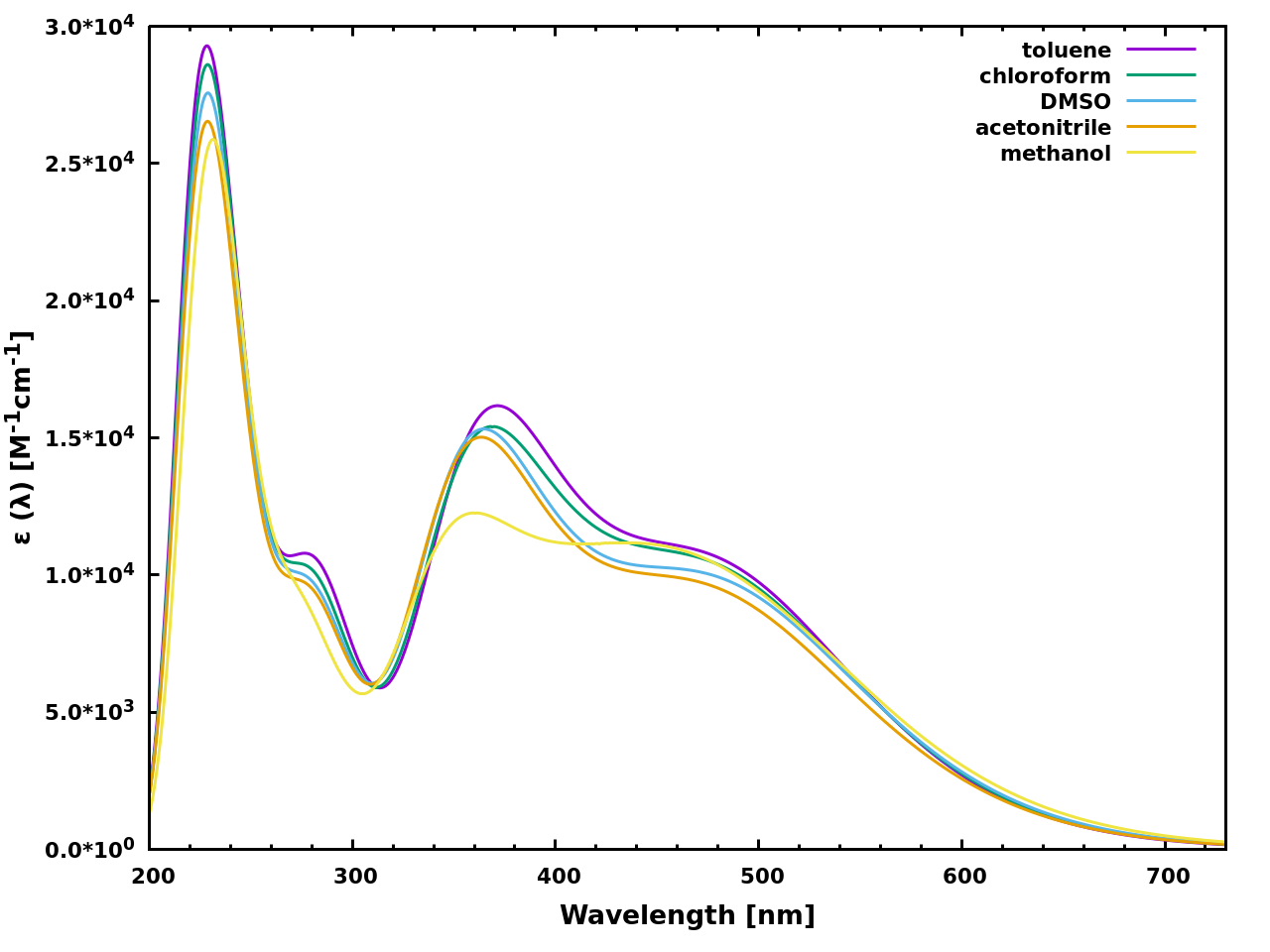

Supplement: Supplementary file 2 — Supplementary file2 (ZIP 5602 KB) [file 43630_2021_71_MOESM2_ESM.zip › simulated_spectra/37.png]

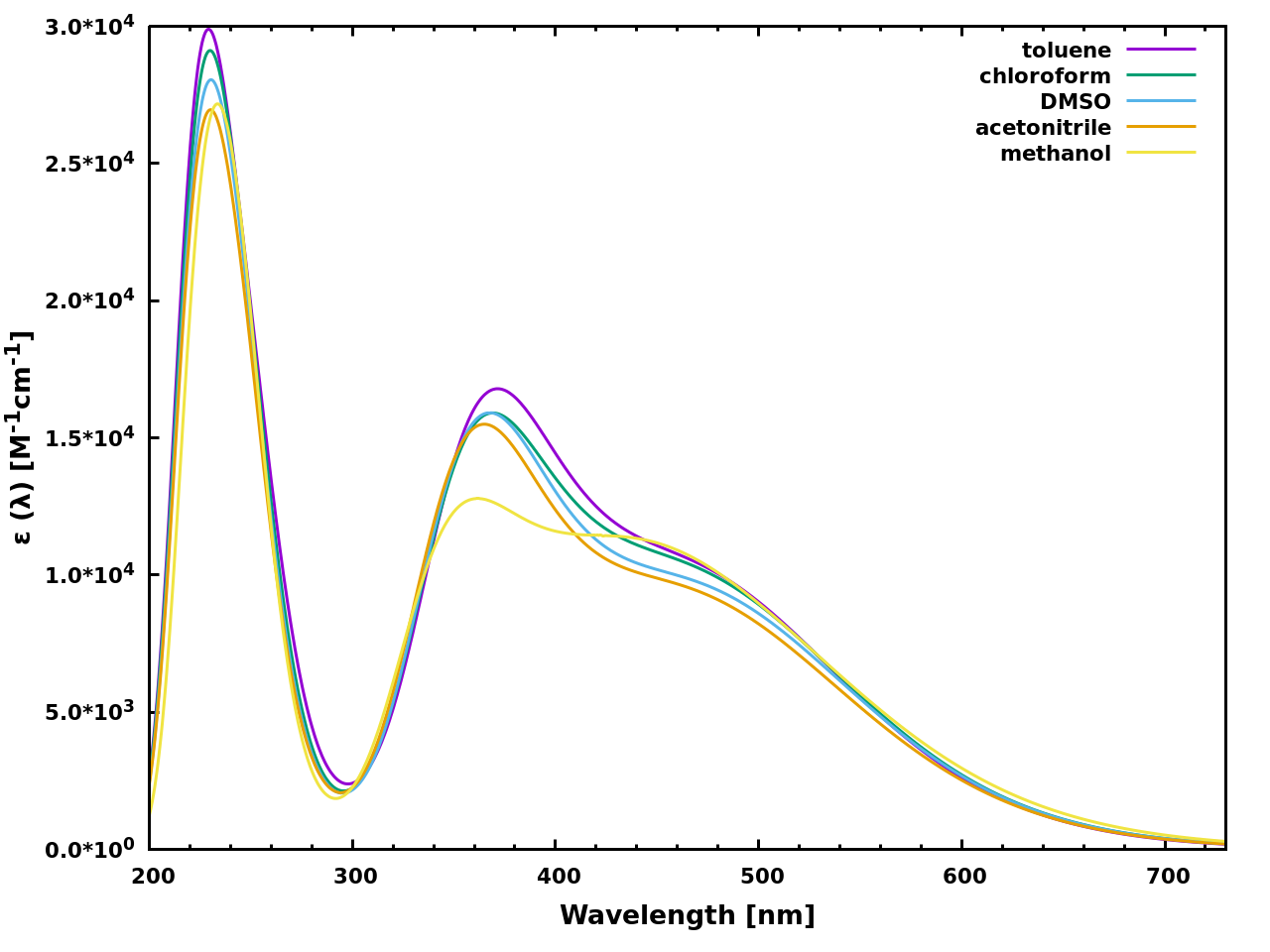

Supplement: Supplementary file 2 — Supplementary file2 (ZIP 5602 KB) [file 43630_2021_71_MOESM2_ESM.zip › simulated_spectra/38.png]

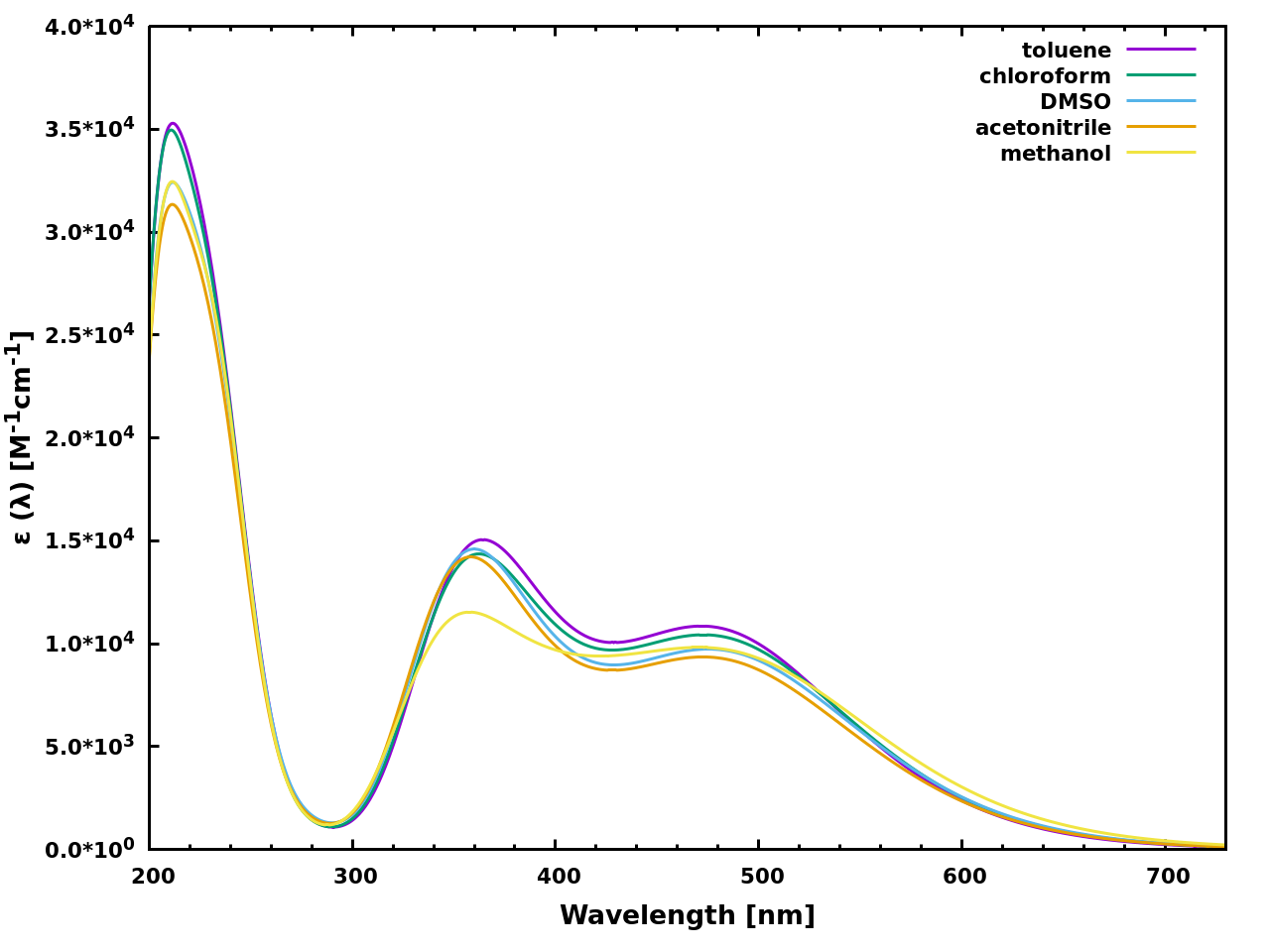

Supplement: Supplementary file 2 — Supplementary file2 (ZIP 5602 KB) [file 43630_2021_71_MOESM2_ESM.zip › simulated_spectra/39.png]

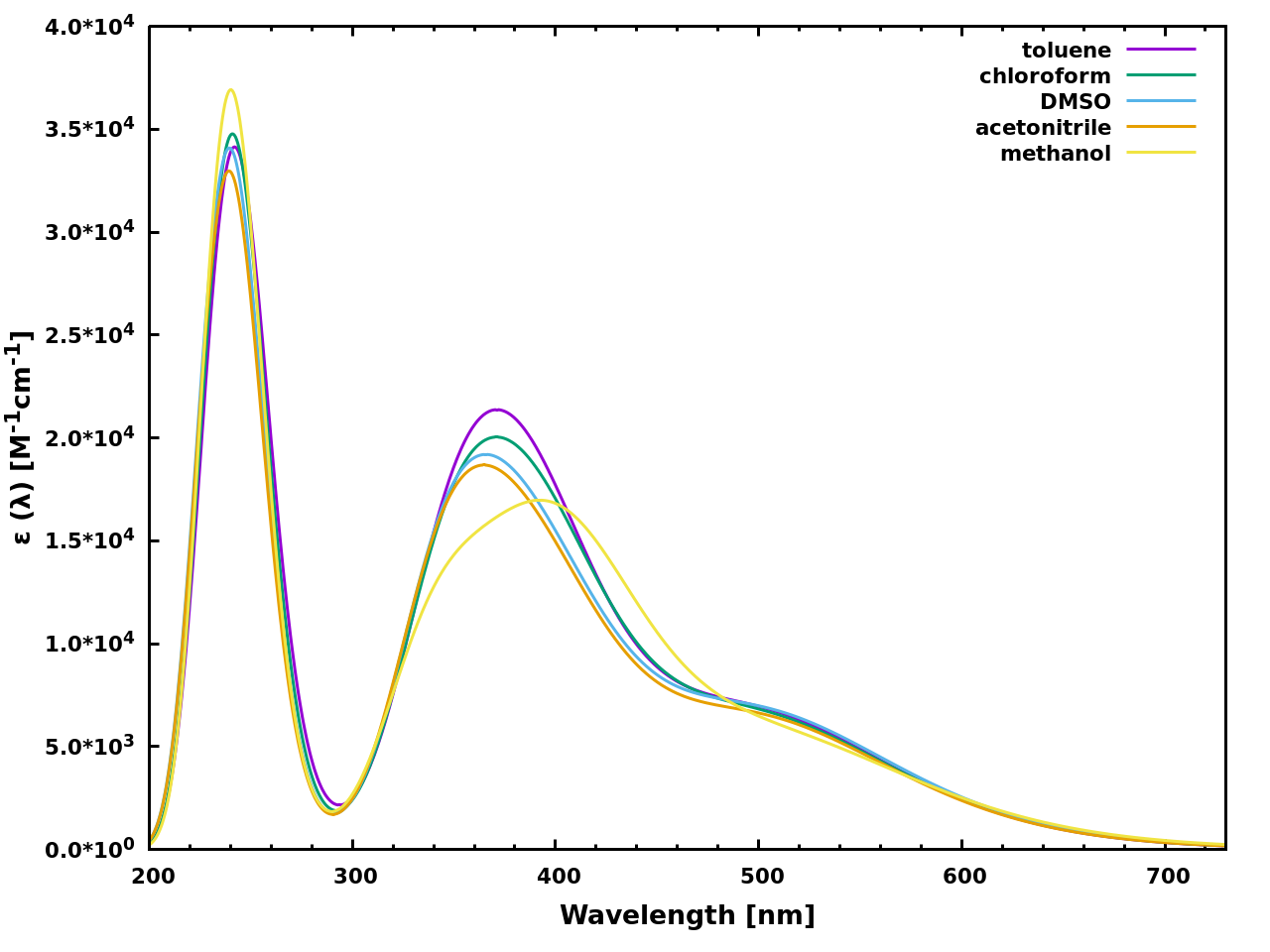

Supplement: Supplementary file 2 — Supplementary file2 (ZIP 5602 KB) [file 43630_2021_71_MOESM2_ESM.zip › simulated_spectra/4.png]

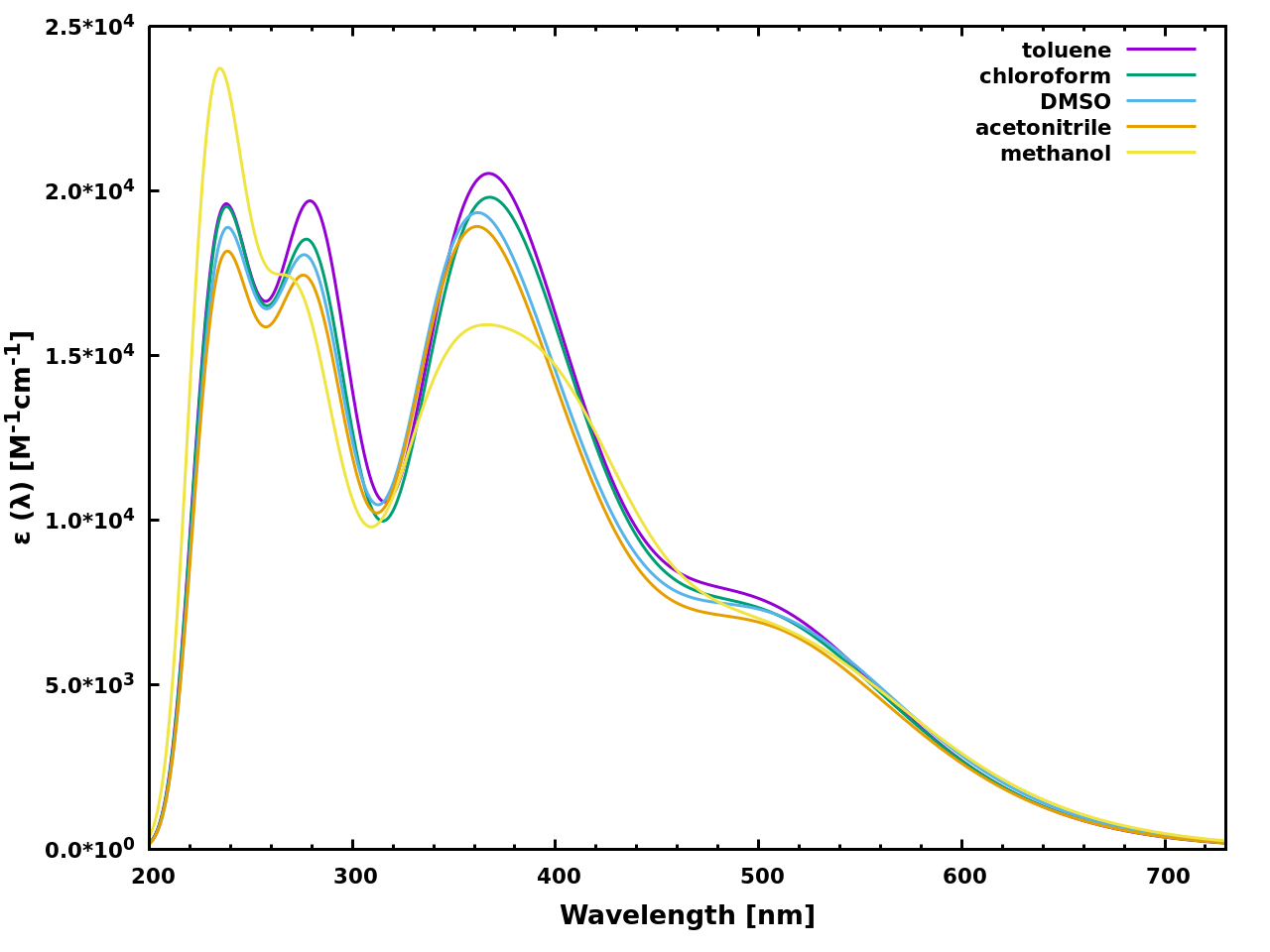

Supplement: Supplementary file 2 — Supplementary file2 (ZIP 5602 KB) [file 43630_2021_71_MOESM2_ESM.zip › simulated_spectra/40.png]

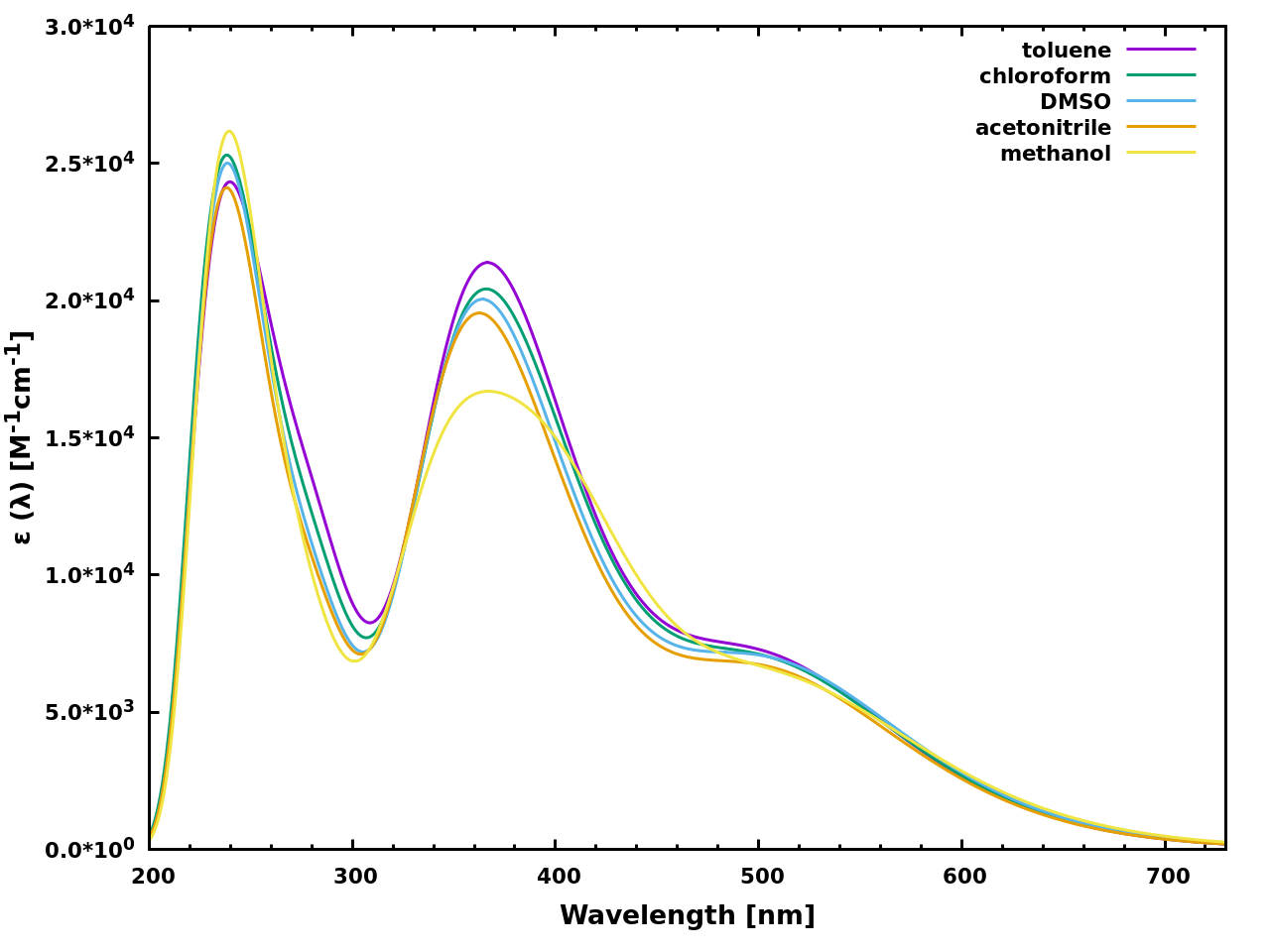

Supplement: Supplementary file 2 — Supplementary file2 (ZIP 5602 KB) [file 43630_2021_71_MOESM2_ESM.zip › simulated_spectra/41.png]

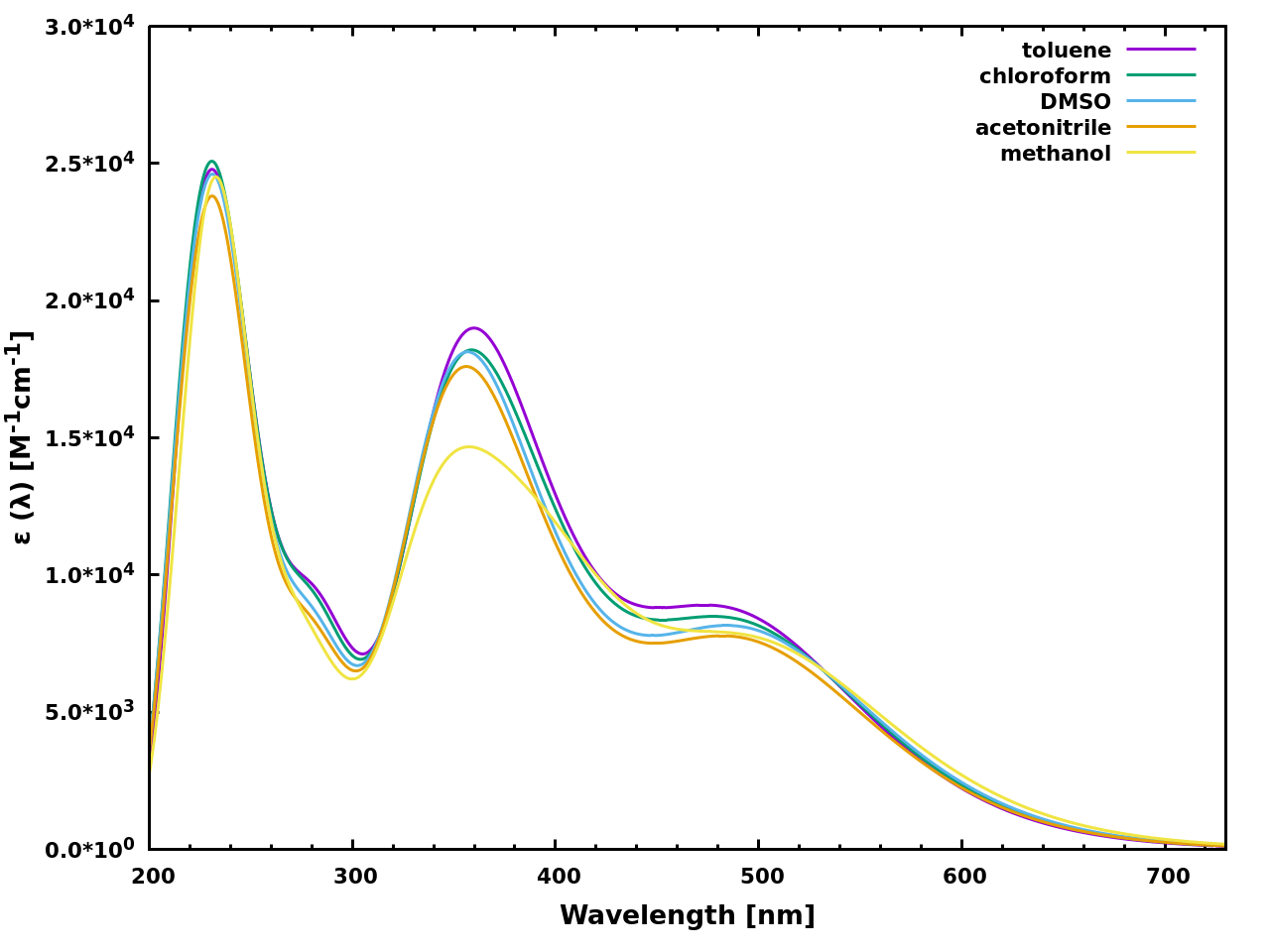

Supplement: Supplementary file 2 — Supplementary file2 (ZIP 5602 KB) [file 43630_2021_71_MOESM2_ESM.zip › simulated_spectra/42.png]

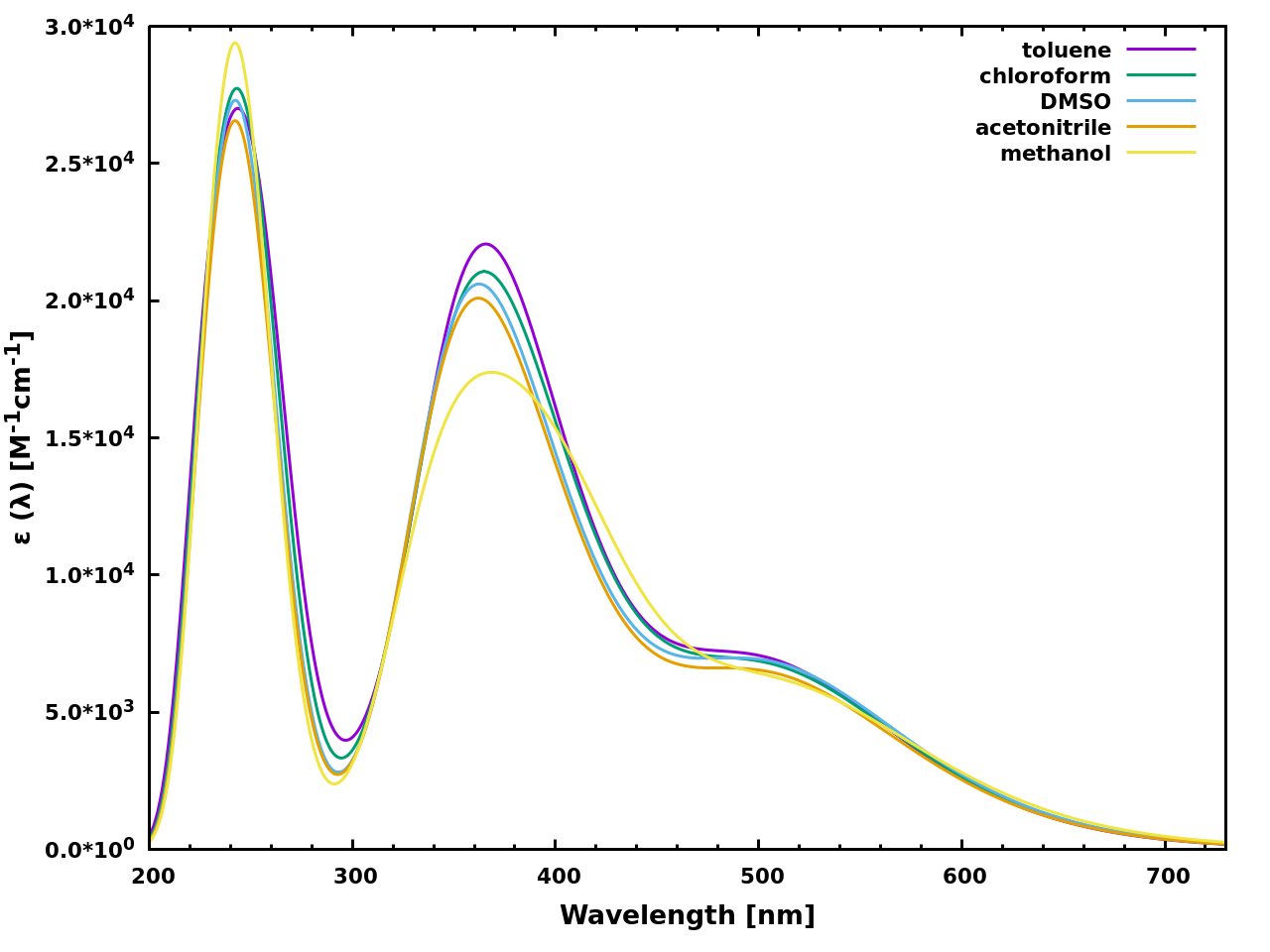

Supplement: Supplementary file 2 — Supplementary file2 (ZIP 5602 KB) [file 43630_2021_71_MOESM2_ESM.zip › simulated_spectra/43.png]

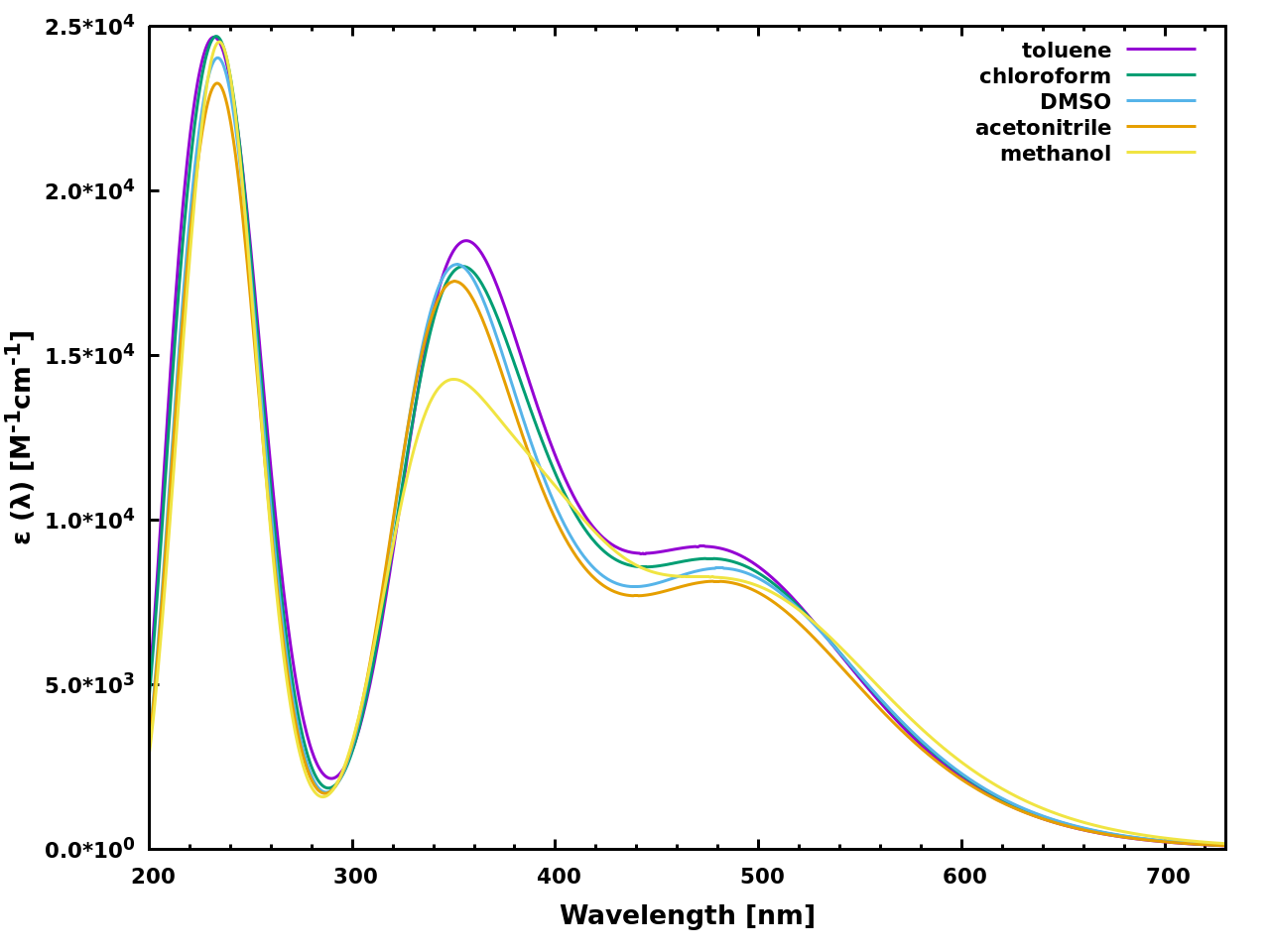

Supplement: Supplementary file 2 — Supplementary file2 (ZIP 5602 KB) [file 43630_2021_71_MOESM2_ESM.zip › simulated_spectra/44.png]

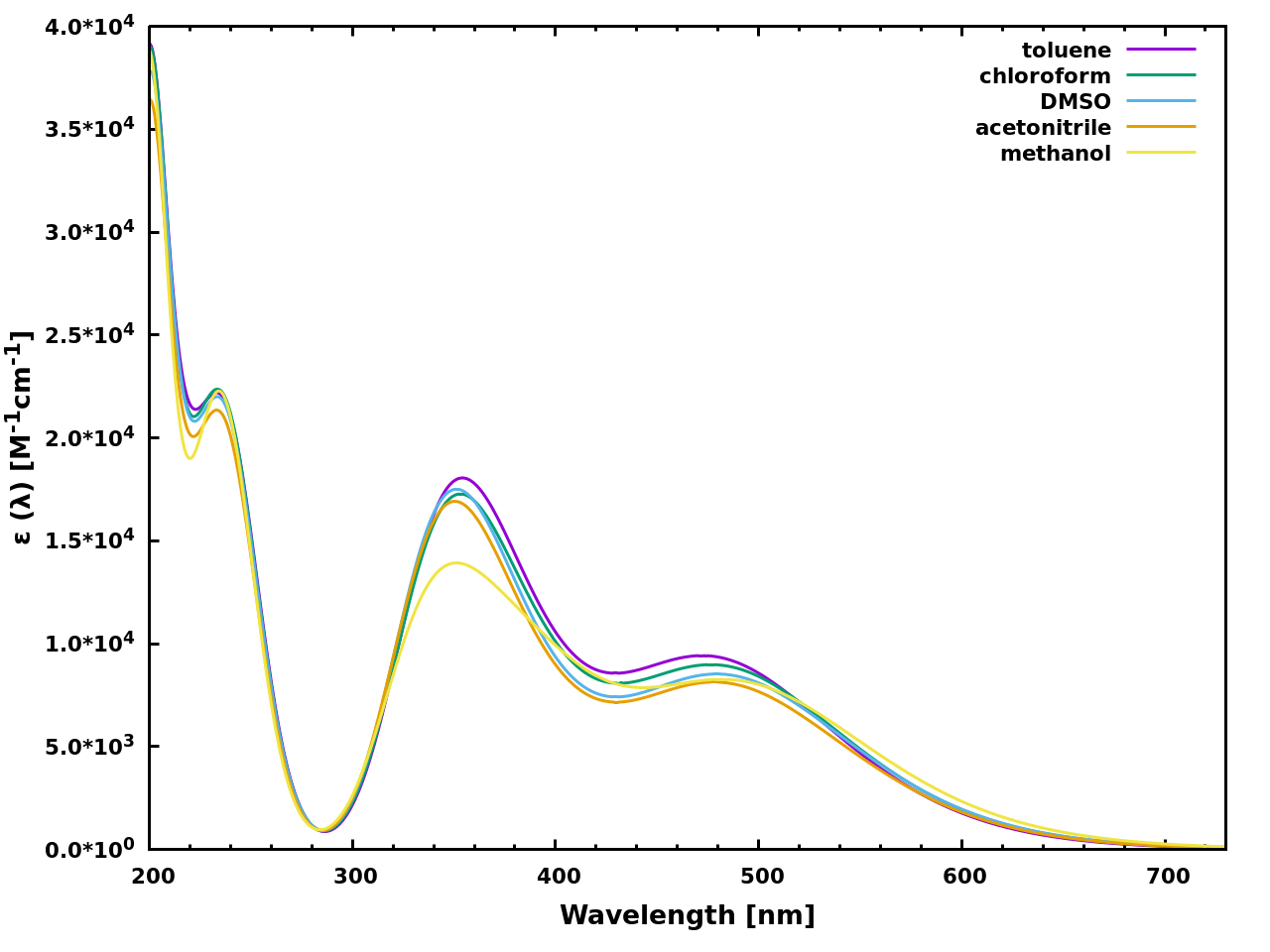

Supplement: Supplementary file 2 — Supplementary file2 (ZIP 5602 KB) [file 43630_2021_71_MOESM2_ESM.zip › simulated_spectra/45.png]

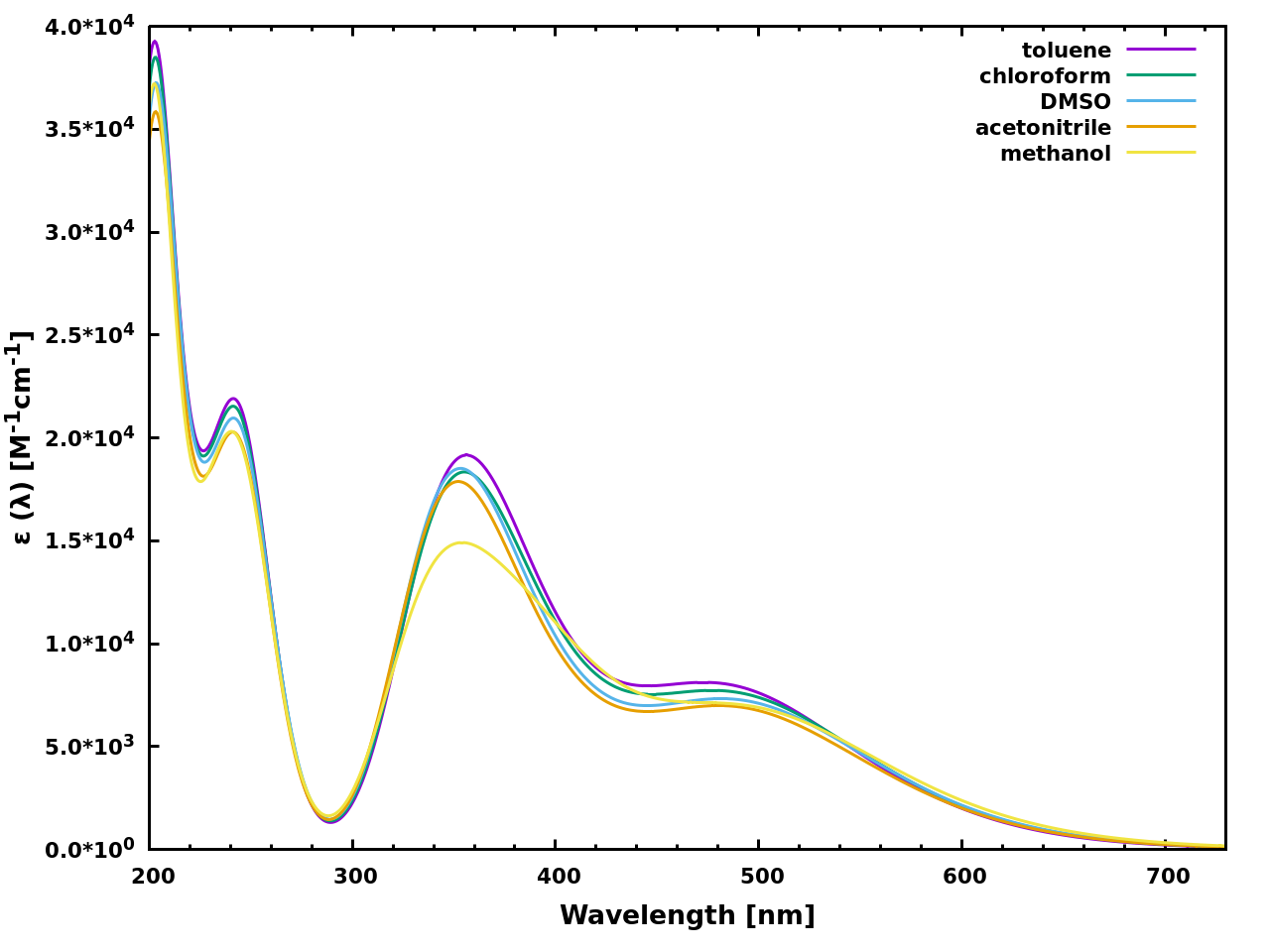

Supplement: Supplementary file 2 — Supplementary file2 (ZIP 5602 KB) [file 43630_2021_71_MOESM2_ESM.zip › simulated_spectra/46.png]

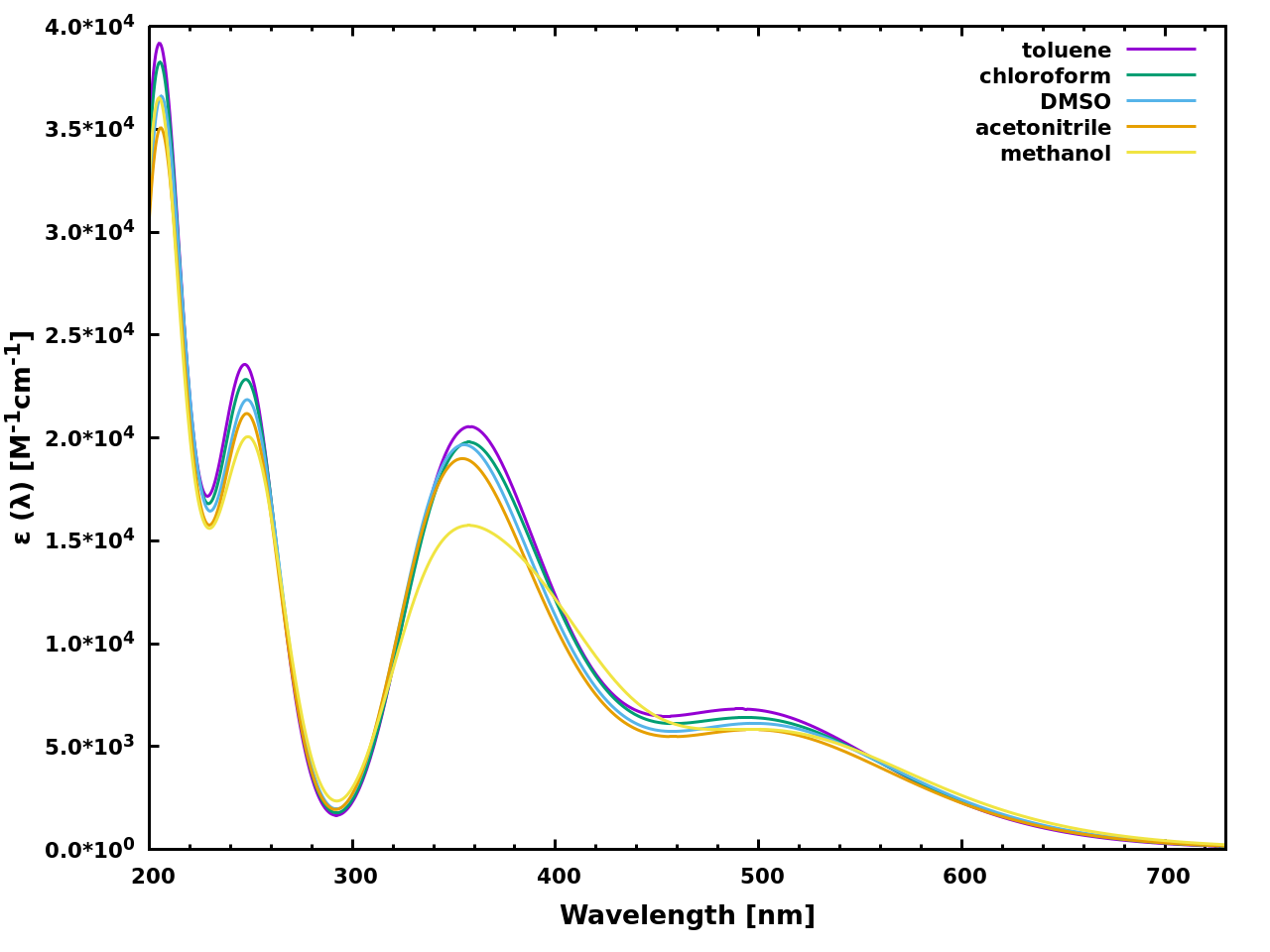

Supplement: Supplementary file 2 — Supplementary file2 (ZIP 5602 KB) [file 43630_2021_71_MOESM2_ESM.zip › simulated_spectra/47.png]

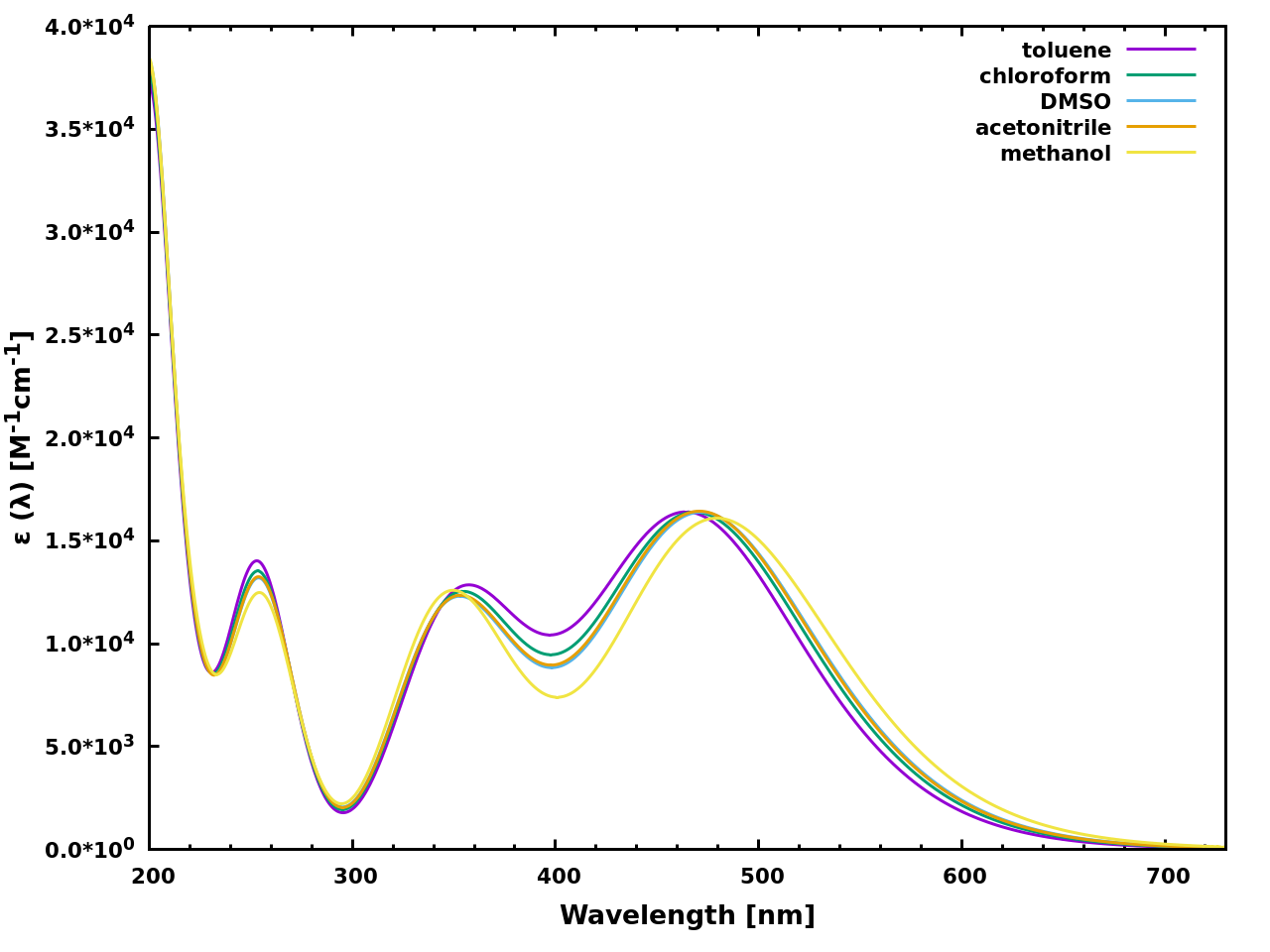

Supplement: Supplementary file 2 — Supplementary file2 (ZIP 5602 KB) [file 43630_2021_71_MOESM2_ESM.zip › simulated_spectra/48.png]

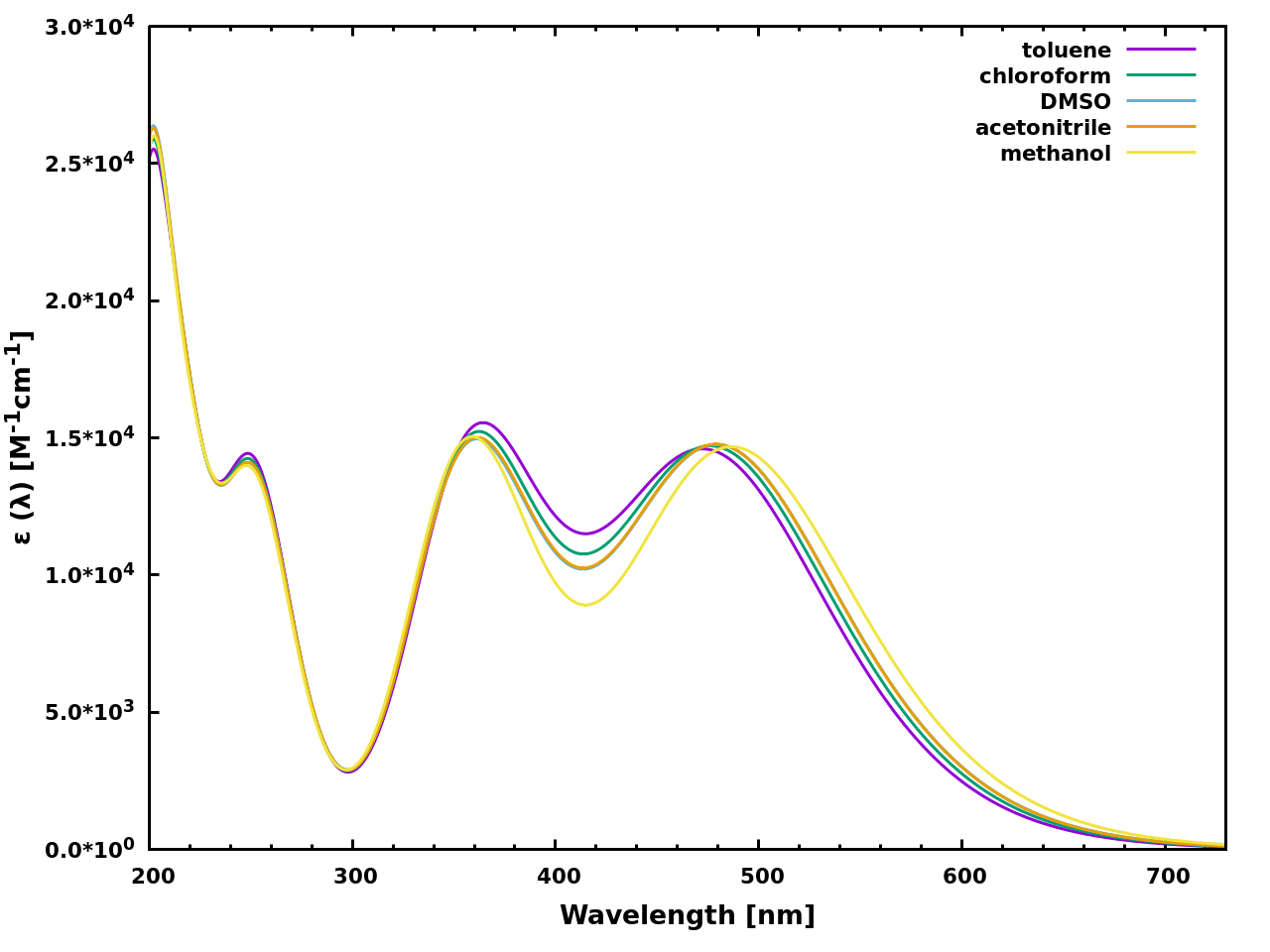

Supplement: Supplementary file 2 — Supplementary file2 (ZIP 5602 KB) [file 43630_2021_71_MOESM2_ESM.zip › simulated_spectra/49.png]

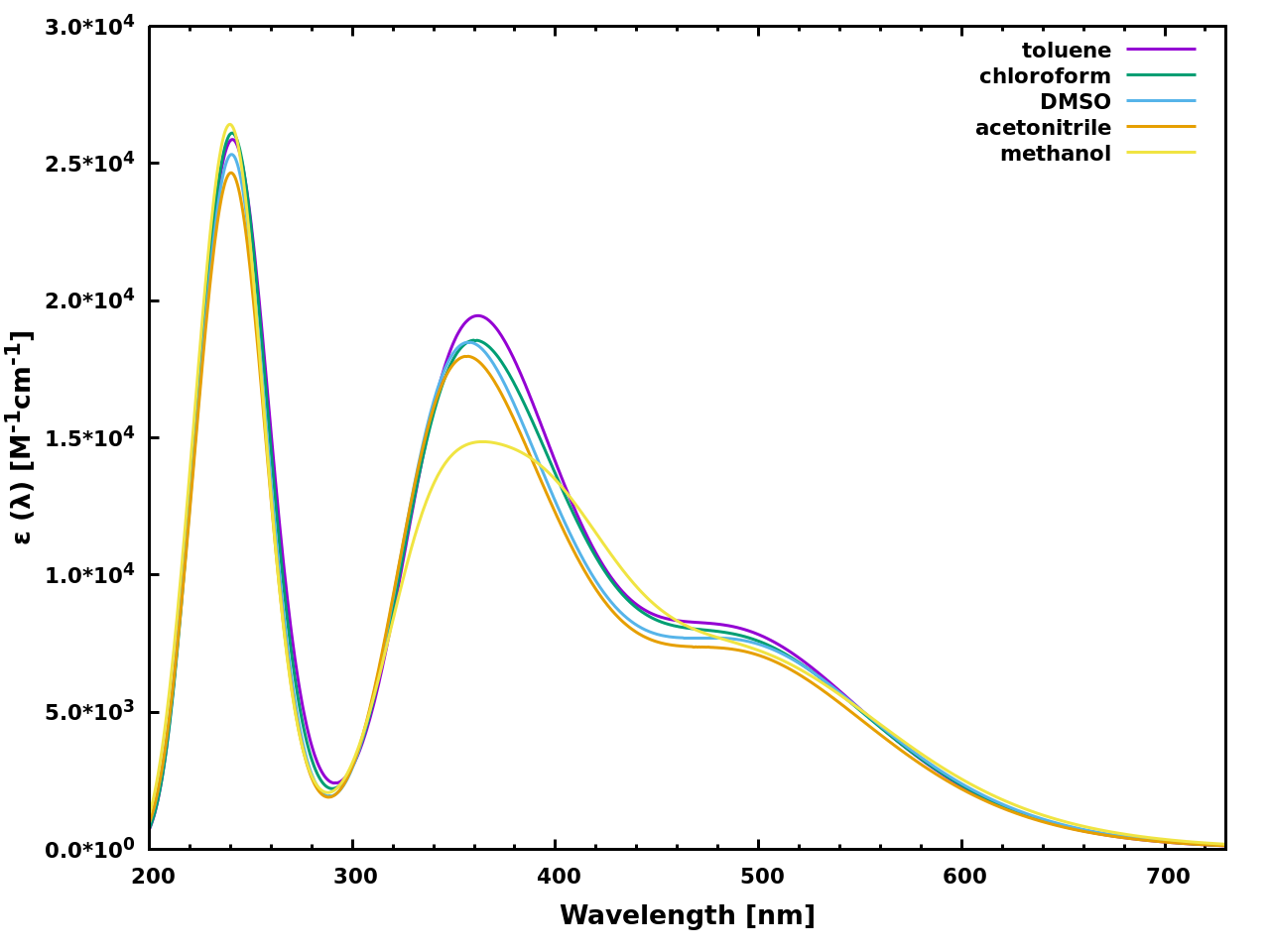

Supplement: Supplementary file 2 — Supplementary file2 (ZIP 5602 KB) [file 43630_2021_71_MOESM2_ESM.zip › simulated_spectra/5.png]

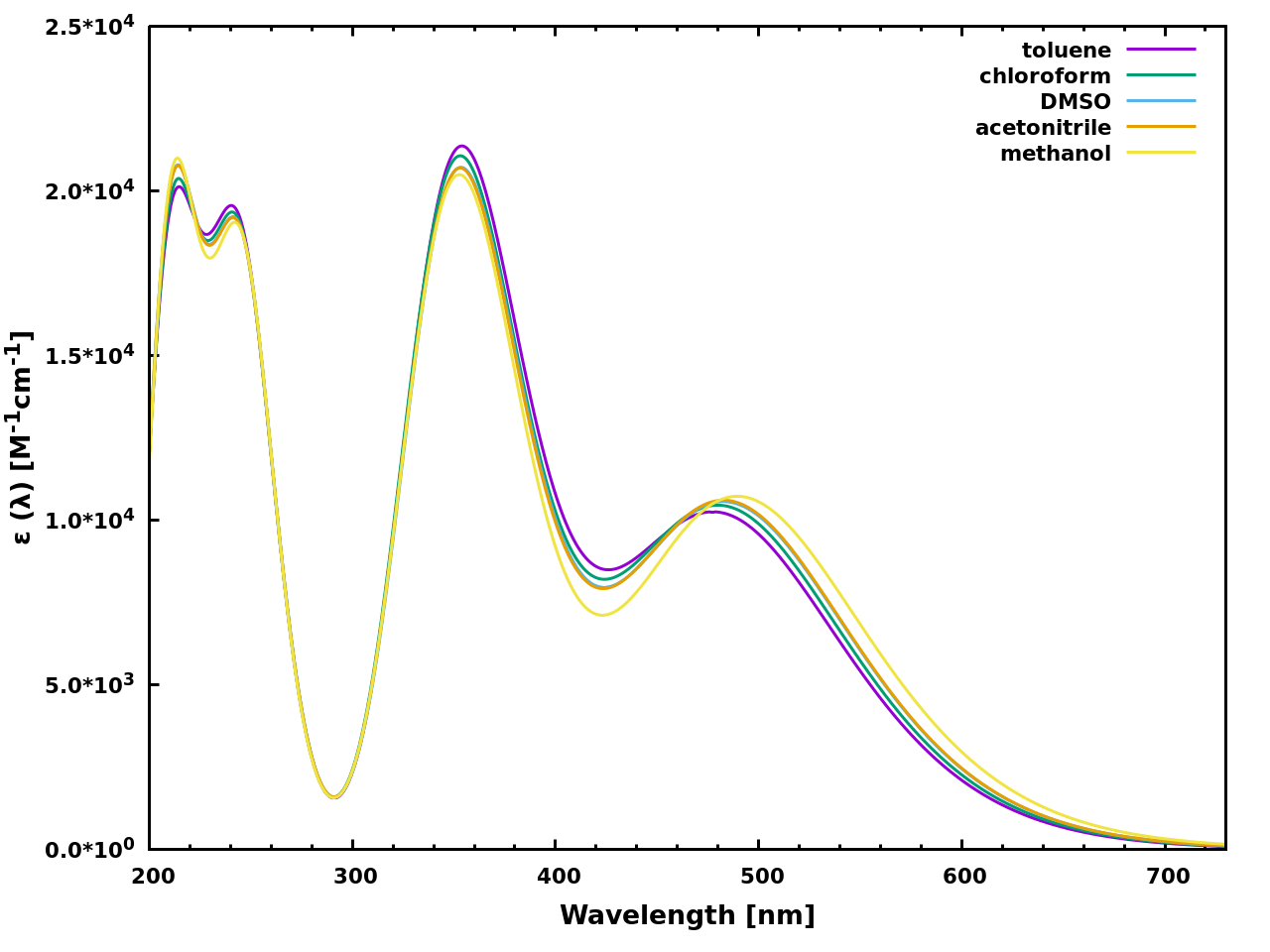

Supplement: Supplementary file 2 — Supplementary file2 (ZIP 5602 KB) [file 43630_2021_71_MOESM2_ESM.zip › simulated_spectra/50.png]

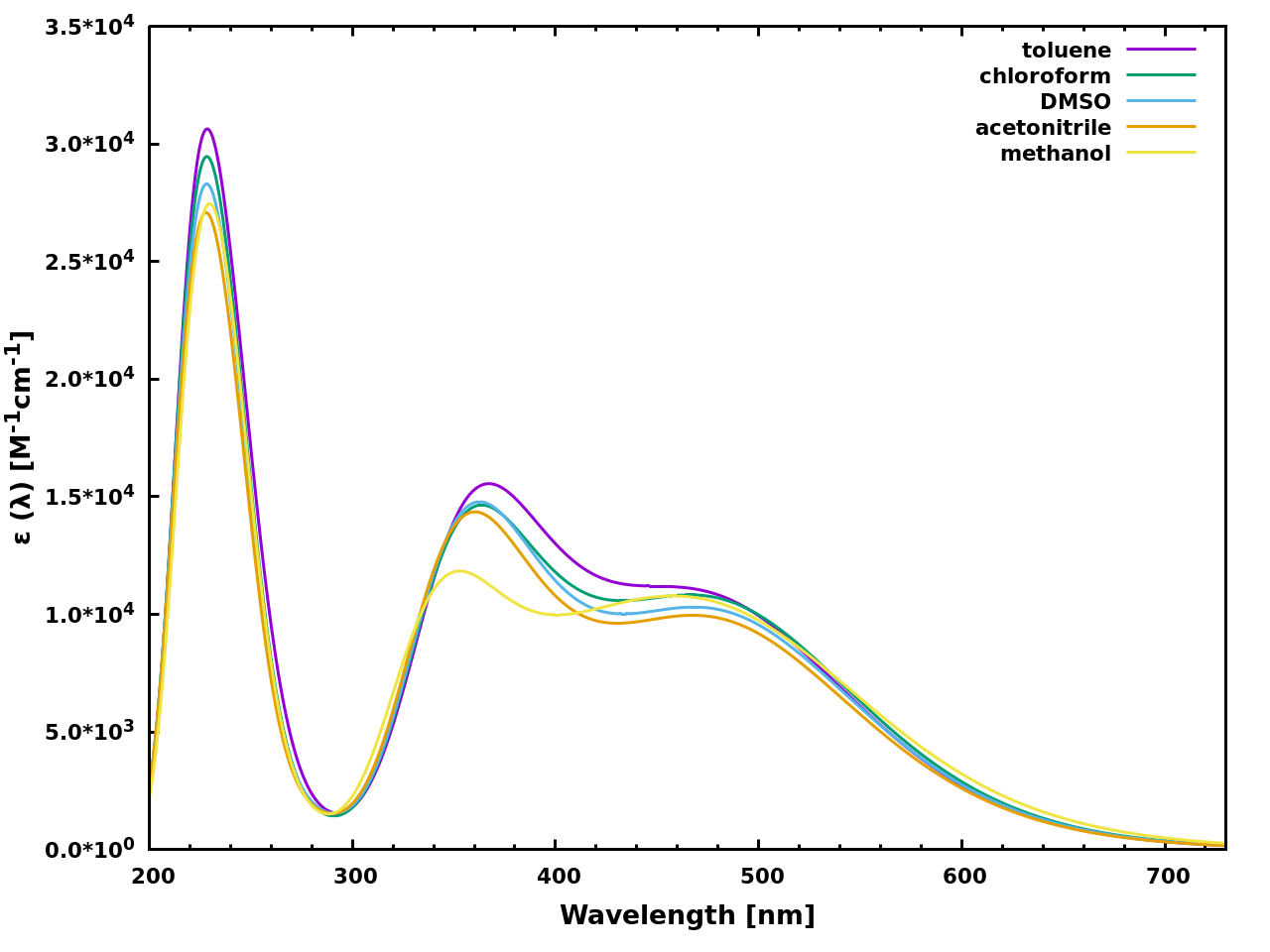

Supplement: Supplementary file 2 — Supplementary file2 (ZIP 5602 KB) [file 43630_2021_71_MOESM2_ESM.zip › simulated_spectra/6.png]

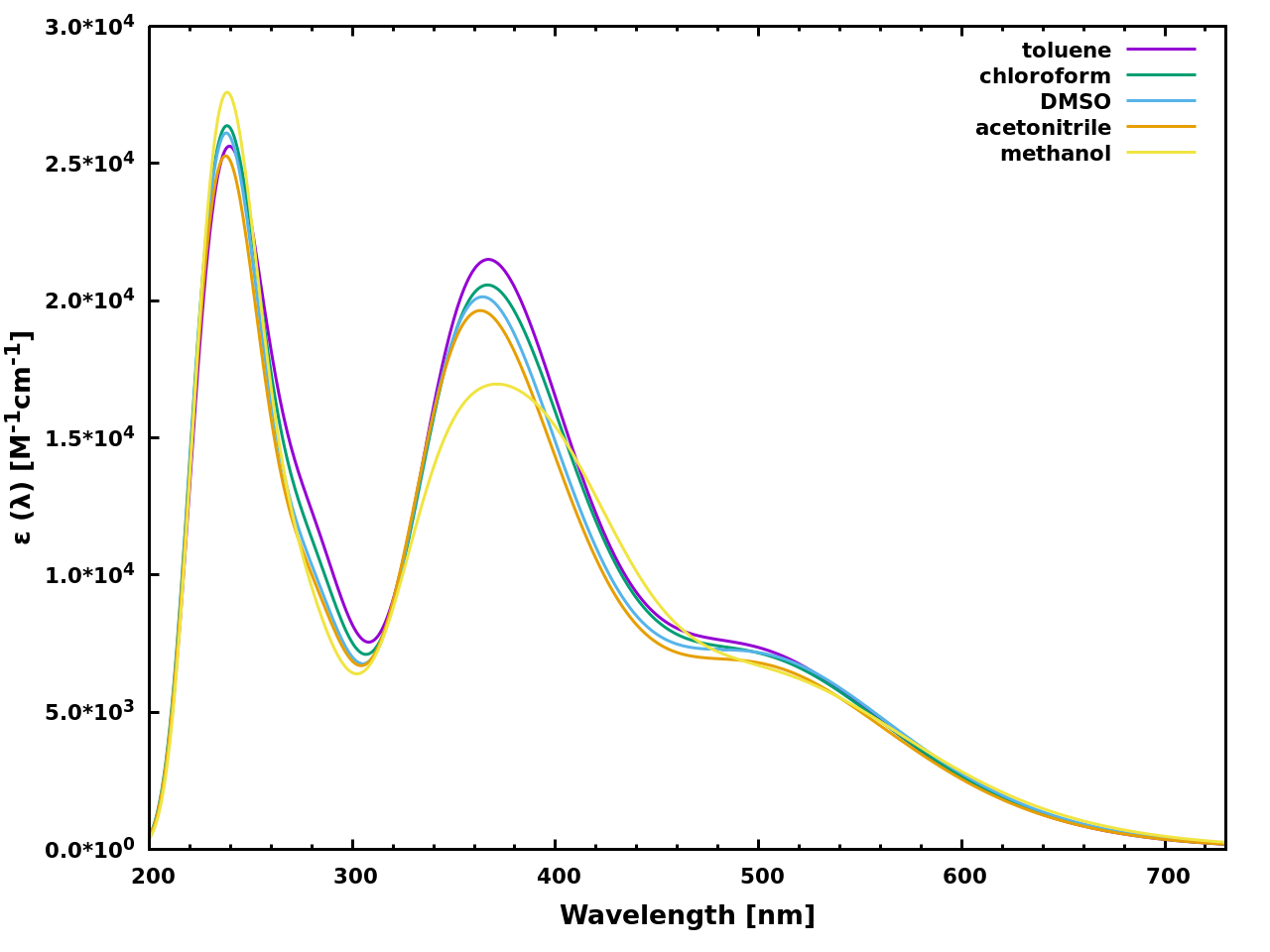

Supplement: Supplementary file 2 — Supplementary file2 (ZIP 5602 KB) [file 43630_2021_71_MOESM2_ESM.zip › simulated_spectra/7.png]

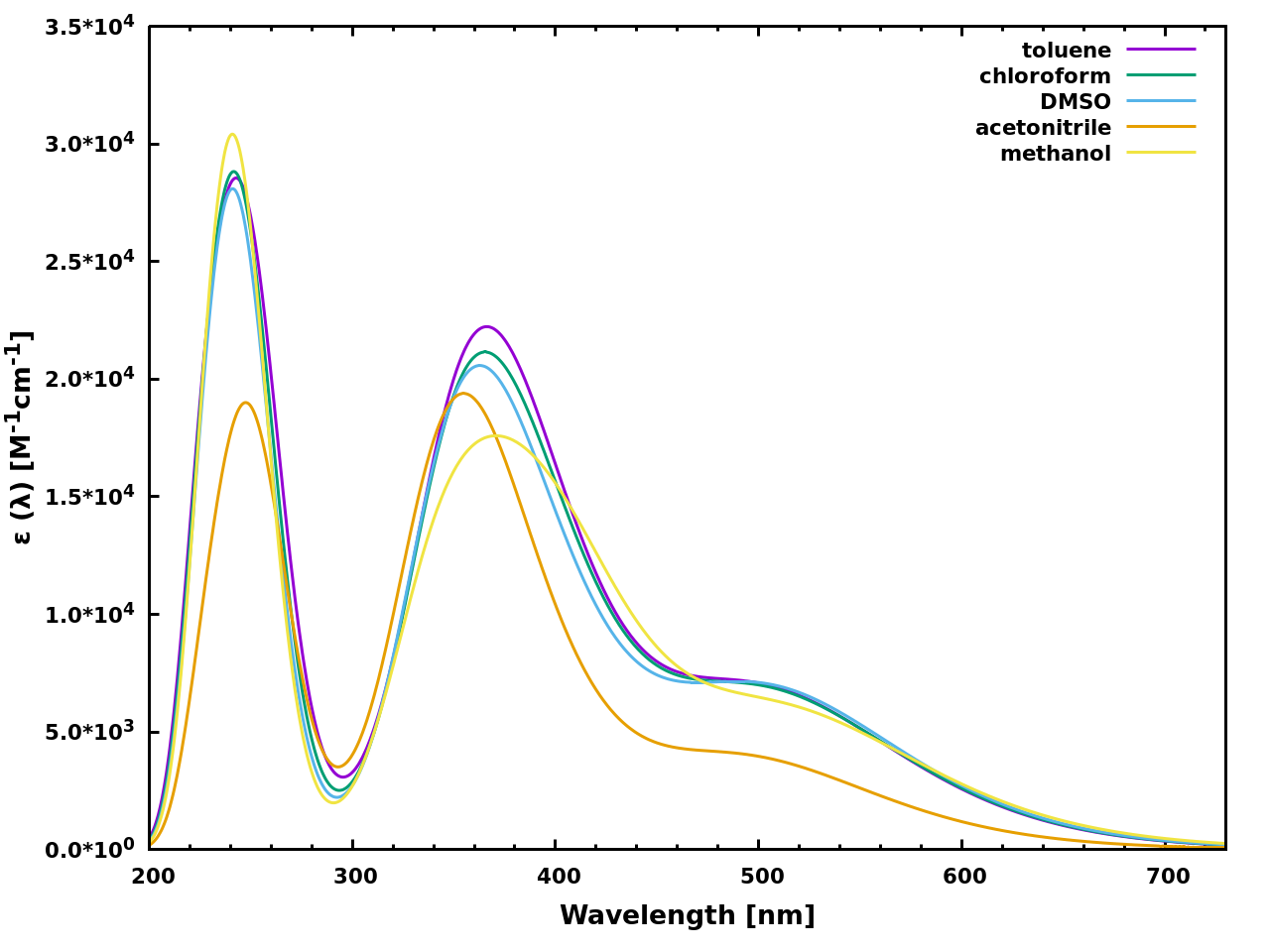

Supplement: Supplementary file 2 — Supplementary file2 (ZIP 5602 KB) [file 43630_2021_71_MOESM2_ESM.zip › simulated_spectra/8.png]

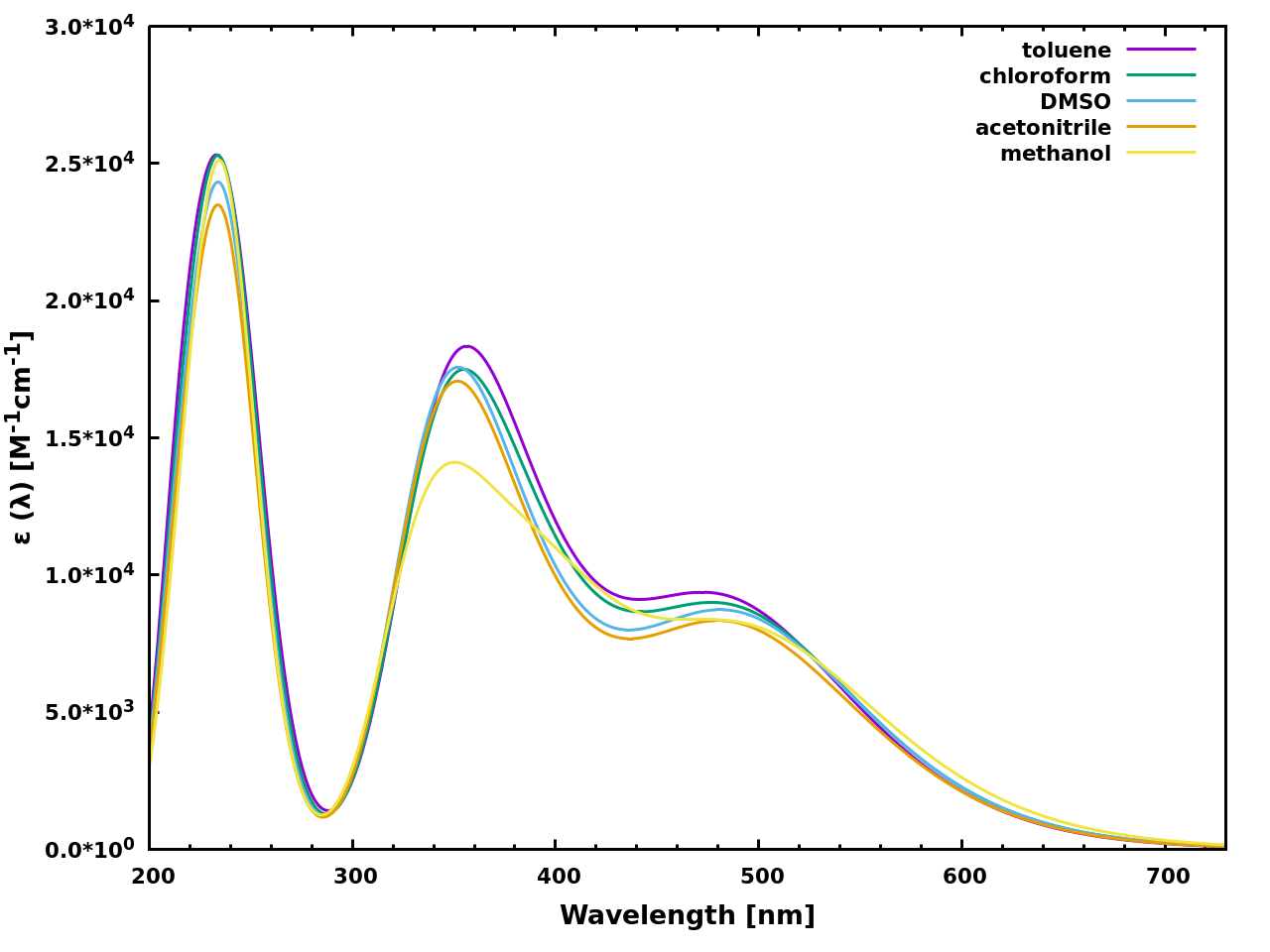

Supplement: Supplementary file 2 — Supplementary file2 (ZIP 5602 KB) [file 43630_2021_71_MOESM2_ESM.zip › simulated_spectra/9.png]
